# Supplementary material for: Rapid increase in erythropoiesis-stimulating agent resistance is a risk factor for poor renal prognosis in patients with chronic kidney disease pre-dialysis: A BRIGHTEN study sub-analysis
Source: PLoS One. 2025 Nov 21;20(11):e0325616. doi: 10.1371/journal.pone.0325616 (PMC12637981; doi:10.1371/journal.pone.0325616)
Supplement: S2 File — (PDF) [file pone.0325616.s002.pdf]

Variable name	Description
random_ID	Anonymized subject identifier randomly assigned to each participant
ADY	Vales represents the number of days from 12 weeks
AVAL	Observed value of ERI-1B at the given study day (ADY)
AGE	Age
CNSR	Censoring indicator flag for time-to-event analysis. 0:censored, 1:event
Event_Day	Study day on which the event occurred. The baseline was defined as 12 weeks after treatment initiation.

Variable name	Description
random_ID	Anonymized subject identifier randomly assigned to each participant
AGE	Age
SEX	Sex
BMI	BMI (kg/m^2) as baseline
PDIAG1_DN	Primary kidney disease - Diabetic nephropathy
PDIAG1_Nephrosclerosis	Primary kidney disease - Nephrosclerosis
PDIAG1_other	Primary kidney disease - Others
PDIAG1_CGN	Primary kidney disease - Glomerulonephritis
CIGARET_Never	Smoking - No smoking history
CIGARET_Ex	Smoking - Smoked in the past but not currently
CIGARET_Current	Smoking - Current smoker
HYPE	Hypertension (140/90<)
MHP01FL	Diabetes
MHP05FL	Ischemic heart disease
MHP06FL	Hospitalization-requiring heart failure
MHP04FL	Stroke
MHP08FL	Peripheral vascular disease
RASPFL	Use of renin-angiotensin system inhibitors
ANTH04FL	Use of Angiotensin II receptor antagonist
ANTH03FL	Use of angiotensin-converting-enzyme inhibitor
IRONFL	Use of Iron supplement as baseline
FED12	Use of Iron supplement until 12 weeks
BPS	Systolic blood pressure (mmHg) at baseline
BPS1	Systolic blood pressure (mmHg) at 12 weeks
BPD	Diastolic blood pressure (mmHg) at baseline
BPD1	Diastolic blood pressure (mmHg) at 12 weeks
BCREAT	Serum Creatinine (mg/dL) at baseline
BCREAT1	Serum Creatinine (mg/dL) at 12 weeks
BEGFRJAP	eGFR (mL/min/1.73m^2) at baseline
BEGFRJAP1	eGFR (mL/min/1.73m^2) at 12 weeks
BHGB	Hb (g/dL) at baseline
BHGB1	Hb (g/dL) at 12 weeks
BALB	Serum Albumin (g/dL) at baseline
BALB1	Serum Albumin (g/dL) at 12 weeks
BIRONC	Serum Iron(μg/dL) at baseline
BIRONC1	Serum Iron(μg/dL) at 12 weeks
BFERRITC	Ferritin(ng/mL) at baseline
BFERRITC1	Ferritin(ng/mL) at 12 weeks
BTSAT	TSAT (%) at baseline
BTSAT1	TSAT (%) at 12 weeks
BCRP	High-sensitivity CRP (ng/mL) at baseline
BCRP1	High-sensitivity CRP (ng/mL) at 12 weeks
BPROBNP	NT-proBNP (pg/mL) at baseline

BPROBNP1	NT-proBNP (pg/mL) at 12 weeks
BPROTCRT	PCR (g/gCr) at baseline
BPROTCRT1	PCR (g/gCr) at 12 weeks
BHGBA1C	HbA1c（NGSP）（%) at baseline
latent_class	latent classes classified by joint latent class model
