# Supplementary material for: Rapid increase in erythropoiesis-stimulating agent resistance is a risk factor for poor renal prognosis in patients with chronic kidney disease pre-dialysis: A BRIGHTEN study sub-analysis
Source: PLoS One. 2025 Nov 21;20(11):e0325616. doi: 10.1371/journal.pone.0325616 (PMC12637981; doi:10.1371/journal.pone.0325616)
Supplement: S3 File — (PDF) [file pone.0325616.s003.pdf]

| random_II | ADY | AVAL | AGE | CNSR | Event_Day |
|---|---|---|---|---|---|
| 1 | 0 | 3.636364 | 83 | 0 | 1135 |
| 1 | 27 | 2.654867 | 83 | 0 | 1135 |
| 1 | 84 | 1.772713 | 83 | 0 | 1135 |
| 1 | 166 | 1.960784 | 83 | 0 | 1135 |
| 1 | 343 | 1.801802 | 83 | 0 | 1135 |
| 1 | 399 | 1.318267 | 83 | 0 | 1135 |
| 2 | 0 | 1.709402 | 70 | 1 | 156 |
| 2 | 21 | 4.093567 | 70 | 1 | 156 |
| 2 | 77 | 2.753196 | 70 | 1 | 156 |
| 3 | 0 | 2.564103 | 79 | 0 | 1030 |
| 3 | 28 | 2.631579 | 79 | 0 | 1030 |
| 3 | 84 | 2 | 79 | 0 | 1030 |
| 3 | 146 | 2.201258 | 79 | 0 | 1030 |
| 3 | 238 | 2.201835 | 79 | 0 | 1030 |
| 3 | 329 | 2.678571 | 79 | 0 | 1030 |
| 3 | 413 | 1.547531 | 79 | 0 | 1030 |
| 3 | 497 | 2.542373 | 79 | 0 | 1030 |
| 3 | 581 | 2.678571 | 79 | 0 | 1030 |
| 4 | 0 | 2.564103 | 88 | 0 | 781 |
| 4 | 28 | 1.181102 | 88 | 0 | 781 |
| 4 | 84 | 0.97561 | 88 | 0 | 781 |
| 4 | 154 | 1.098901 | 88 | 0 | 781 |
| 4 | 252 | 1.212121 | 88 | 0 | 781 |
| 4 | 336 | 1.846154 | 88 | 0 | 781 |
| 4 | 427 | 4.155844 | 88 | 0 | 781 |
| 4 | 504 | 3.571429 | 88 | 0 | 781 |
| 4 | 588 | 3.571429 | 88 | 0 | 781 |
| 5 | 0 | 2.631579 | 77 | 0 | 816 |
| 5 | 28 | 2.068966 | 77 | 0 | 816 |
| 5 | 91 | 2.678571 | 77 | 0 | 816 |
| 5 | 161 | 2.884615 | 77 | 0 | 816 |
| 5 | 252 | 1.339286 | 77 | 0 | 816 |
| 5 | 315 | 1.801802 | 77 | 0 | 816 |
| 5 | 427 | 2.264151 | 77 | 0 | 816 |
| 5 | 518 | 3.157895 | 77 | 0 | 816 |
| 5 | 556 | 1.434426 | 77 | 0 | 816 |
| 6 | 0 | 2.201835 | 46 | 0 | 1048 |
| 6 | 35 | 1.587902 | 46 | 0 | 1048 |

| | | | | | |
|---|---|---|---|---|---|
| 6 | 81 | 2.142857 | 46 | 0 | 1048 |
| 6 | 172 | 2.264151 | 46 | 0 | 1048 |
| 6 | 235 | 2.105263 | 46 | 0 | 1048 |
| 6 | 326 | 3.539823 | 46 | 0 | 1048 |
| 6 | 410 | 2.181818 | 46 | 0 | 1048 |
| 6 | 508 | 2.830189 | 46 | 0 | 1048 |
| 6 | 564 | 2.222222 | 46 | 0 | 1048 |
| 7 | 0 | 4 | 74 | 0 | 1110 |
| 7 | 28 | 6.315789 | 74 | 0 | 1110 |
| 7 | 91 | 4.067797 | 74 | 0 | 1110 |
| 7 | 175 | 2.325581 | 74 | 0 | 1110 |
| 7 | 252 | 0.350877 | 74 | 0 | 1110 |
| 7 | 343 | 0.331538 | 74 | 0 | 1110 |
| 7 | 385 | 1.234568 | 74 | 0 | 1110 |
| 7 | 511 | 0.595238 | 74 | 0 | 1110 |
| 7 | 581 | 2.352941 | 74 | 0 | 1110 |
| 8 | 0 | 0.275482 | 67 | 1 | 169 |
| 8 | 28 | 0.275482 | 67 | 1 | 169 |
| 8 | 84 | 1.190476 | 67 | 1 | 169 |
| 9 | 0 | 3.571429 | 78 | 0 | 764 |
| 10 | 0 | 1.886792 | 68 | 0 | 927 |
| 10 | 63 | 0.880088 | 68 | 0 | 927 |
| 10 | 126 | 0.808081 | 68 | 0 | 927 |
| 10 | 245 | 1.154401 | 68 | 0 | 927 |
| 10 | 294 | 1.284109 | 68 | 0 | 927 |
| 10 | 378 | 26.08696 | 68 | 0 | 927 |
| 10 | 481 | 2.380952 | 68 | 0 | 927 |
| 10 | 567 | 5.825243 | 68 | 0 | 927 |
| 11 | 0 | 5.940594 | 89 | 1 | 577 |
| 11 | 28 | 5.940594 | 89 | 1 | 577 |
| 11 | 84 | 9.69697 | 89 | 1 | 577 |
| 11 | 182 | 12.12121 | 89 | 1 | 577 |
| 11 | 273 | 9.6 | 89 | 1 | 577 |
| 11 | 329 | 10.54945 | 89 | 1 | 577 |
| 11 | 399 | 15 | 89 | 1 | 577 |
| 11 | 483 | 11.16279 | 89 | 1 | 577 |
| 11 | 546 | 23.37662 | 89 | 1 | 577 |
| 12 | 0 | 6.521739 | 54 | 0 | 708 |
| 12 | 28 | 6.896552 | 54 | 0 | 708 |

| | | | | | |
|---|---|---|---|---|---|
| 12 | 84 | 6.666667 | 54 | 0 | 708 |
| 12 | 168 | 12.63158 | 54 | 0 | 708 |
| 12 | 224 | 19.14894 | 54 | 0 | 708 |
| 12 | 315 | 18.36735 | 54 | 0 | 708 |
| 12 | 399 | 18.36735 | 54 | 0 | 708 |
| 12 | 490 | 25.26316 | 54 | 0 | 708 |
| 12 | 567 | 18 | 54 | 0 | 708 |
| 13 | 0 | 2.5 | 67 | 0 | 1065 |
| 13 | 28 | 1.229508 | 67 | 0 | 1065 |
| 13 | 74 | 1.223242 | 67 | 0 | 1065 |
| 13 | 168 | 2 | 67 | 0 | 1065 |
| 13 | 252 | 3.921569 | 67 | 0 | 1065 |
| 13 | 336 | 6.61157 | 67 | 0 | 1065 |
| 13 | 413 | 5.538462 | 67 | 0 | 1065 |
| 13 | 504 | 6.747891 | 67 | 0 | 1065 |
| 13 | 581 | 7.272727 | 67 | 0 | 1065 |
| 14 | 0 | 1.818182 | 83 | 0 | 652 |
| 14 | 35 | 1.869159 | 83 | 0 | 652 |
| 14 | 63 | 3.773585 | 83 | 0 | 652 |
| 14 | 176 | 3.076923 | 83 | 0 | 652 |
| 14 | 239 | 2.285714 | 83 | 0 | 652 |
| 14 | 351 | 2.702703 | 83 | 0 | 652 |
| 14 | 407 | 2.85423 | 83 | 0 | 652 |
| 14 | 504 | 2.727273 | 83 | 0 | 652 |
| 14 | 588 | 2.937063 | 83 | 0 | 652 |
| 15 | 0 | 0.940439 | 77 | 0 | 955 |
| 15 | 24 | 1.000834 | 77 | 0 | 955 |
| 15 | 84 | 0.894188 | 77 | 0 | 955 |
| 15 | 196 | 7.619048 | 77 | 0 | 955 |
| 15 | 287 | 12.76596 | 77 | 0 | 955 |
| 15 | 392 | 8.659794 | 77 | 0 | 955 |
| 15 | 511 | 5.574913 | 77 | 0 | 955 |
| 15 | 609 | 5.574913 | 77 | 0 | 955 |
| 16 | 0 | 7.017544 | 71 | 0 | 1354 |
| 16 | 29 | 2.777778 | 71 | 0 | 1354 |
| 16 | 85 | 5.321508 | 71 | 0 | 1354 |
| 16 | 163 | 3.155819 | 71 | 0 | 1354 |
| 16 | 254 | 5.882353 | 71 | 0 | 1354 |
| 16 | 337 | 6.315789 | 71 | 0 | 1354 |

| | | | | | |
|---|---|---|---|---|---|
| 16 | 421 | 5.940594 | 71 | 0 | 1354 |
| 16 | 505 | 6.25 | 71 | 0 | 1354 |
| 16 | 589 | 5.940594 | 71 | 0 | 1354 |
| 17 | 0 | 2.242991 | 56 | 0 | 792 |
| 17 | 91 | 2.777778 | 56 | 0 | 792 |
| 17 | 189 | 2.4 | 56 | 0 | 792 |
| 17 | 245 | 1.960784 | 56 | 0 | 792 |
| 17 | 343 | 2.330097 | 56 | 0 | 792 |
| 17 | 406 | 2.376238 | 56 | 0 | 792 |
| 17 | 497 | 2.970297 | 56 | 0 | 792 |
| 17 | 587 | 2.753196 | 56 | 0 | 792 |
| 18 | 0 | 1.325758 | 69 | 0 | 761 |
| 18 | 28 | 1.5625 | 69 | 0 | 761 |
| 18 | 84 | 1.680672 | 69 | 0 | 761 |
| 18 | 168 | 1.886792 | 69 | 0 | 761 |
| 18 | 252 | 1.941748 | 69 | 0 | 761 |
| 18 | 336 | 1.785714 | 69 | 0 | 761 |
| 18 | 420 | 1.923077 | 69 | 0 | 761 |
| 18 | 504 | 2.962963 | 69 | 0 | 761 |
| 18 | 588 | 4.444444 | 69 | 0 | 761 |
| 19 | 28 | 5.825243 | 78 | 0 | 124 |
| 19 | 70 | 6.315789 | 78 | 0 | 124 |
| 20 | 0 | 7.619048 | 74 | 1 | 490 |
| 20 | 42 | 14.69388 | 74 | 1 | 490 |
| 20 | 77 | 14.11765 | 74 | 1 | 490 |
| 20 | 147 | 13.09091 | 74 | 1 | 490 |
| 21 | 0 | 3.669725 | 75 | 1 | 91 |
| 22 | 0 | 2.033898 | 67 | 0 | 1212 |
| 22 | 35 | 1.709402 | 67 | 0 | 1212 |
| 22 | 77 | 2.105263 | 67 | 0 | 1212 |
| 22 | 168 | 2.105263 | 67 | 0 | 1212 |
| 22 | 273 | 2.330097 | 67 | 0 | 1212 |
| 22 | 336 | 4.485981 | 67 | 0 | 1212 |
| 22 | 406 | 5.405405 | 67 | 0 | 1212 |
| 22 | 490 | 4.285714 | 67 | 0 | 1212 |
| 22 | 581 | 3.448276 | 67 | 0 | 1212 |
| 23 | 0 | 0.970874 | 86 | 1 | 1108 |
| 23 | 77 | 2.307692 | 86 | 1 | 1108 |
| 23 | 182 | 2.285714 | 86 | 1 | 1108 |

| | | | | | |
|---|---|---|---|---|---|
| 23 | 259 | 5.526316 | 86 | 1 | 1108 |
| 23 | 336 | 10 | 86 | 1 | 1108 |
| 23 | 406 | 17.64706 | 86 | 1 | 1108 |
| 23 | 497 | 15.15789 | 86 | 1 | 1108 |
| 23 | 588 | 18 | 86 | 1 | 1108 |
| 24 | 0 | 6.015038 | 62 | 1 | 866 |
| 24 | 98 | 9.89011 | 62 | 1 | 866 |
| 24 | 175 | 6.728972 | 62 | 1 | 866 |
| 24 | 245 | 11.88119 | 62 | 1 | 866 |
| 24 | 329 | 5.294118 | 62 | 1 | 866 |
| 24 | 413 | 10.49563 | 62 | 1 | 866 |
| 24 | 511 | 9.89011 | 62 | 1 | 866 |
| 24 | 567 | 9.89011 | 62 | 1 | 866 |
| 25 | 0 | 2.515723 | 74 | 1 | 187 |
| 25 | 63 | 10.10526 | 74 | 1 | 187 |
| 26 | 0 | 1.428571 | 78 | 1 | 259 |
| 26 | 91 | 1.666667 | 78 | 1 | 259 |
| 26 | 154 | 0.467836 | 78 | 1 | 259 |
| 26 | 231 | 0.498442 | 78 | 1 | 259 |
| 27 | 0 | 6.015038 | 70 | 0 | 1161 |
| 27 | 21 | 5.620609 | 70 | 0 | 1161 |
| 27 | 84 | 4.719764 | 70 | 0 | 1161 |
| 27 | 147 | 6.451613 | 70 | 0 | 1161 |
| 27 | 259 | 7.54717 | 70 | 0 | 1161 |
| 27 | 322 | 7.619048 | 70 | 0 | 1161 |
| 27 | 385 | 7.476636 | 70 | 0 | 1161 |
| 27 | 511 | 8.333333 | 70 | 0 | 1161 |
| 27 | 567 | 8.737864 | 70 | 0 | 1161 |
| 28 | 0 | 10.28571 | 53 | 0 | 1128 |
| 28 | 84 | 14.25743 | 53 | 0 | 1128 |
| 28 | 147 | 5.454545 | 53 | 0 | 1128 |
| 28 | 224 | 10.90909 | 53 | 0 | 1128 |
| 28 | 315 | 4.181185 | 53 | 0 | 1128 |
| 28 | 427 | 8 | 53 | 0 | 1128 |
| 28 | 483 | 4.013378 | 53 | 0 | 1128 |
| 28 | 546 | 5.82996 | 53 | 0 | 1128 |
| 29 | 21 | 1.020408 | 83 | 1 | 206 |
| 29 | 105 | 1.869159 | 83 | 1 | 206 |
| 29 | 133 | 1.469431 | 83 | 1 | 206 |

| | | | | | |
|---|---|---|---|---|---|
| 30 | 0 | 2.4 | 70 | 1 | 141 |
| 30 | 35 | 1.572739 | 70 | 1 | 141 |
| 30 | 84 | 2.258065 | 70 | 1 | 141 |
| 31 | 0 | 4.571429 | 70 | 0 | 750 |
| 31 | 35 | 4.285714 | 70 | 0 | 750 |
| 31 | 70 | 4.137931 | 70 | 0 | 750 |
| 31 | 161 | 5.309735 | 70 | 0 | 750 |
| 31 | 245 | 4.324324 | 70 | 0 | 750 |
| 31 | 343 | 4.660194 | 70 | 0 | 750 |
| 31 | 413 | 6.315789 | 70 | 0 | 750 |
| 31 | 504 | 5.825243 | 70 | 0 | 750 |
| 31 | 595 | 12.76596 | 70 | 0 | 750 |
| 32 | 0 | 6.521739 | 76 | 0 | 276 |
| 32 | 28 | 6.818182 | 76 | 0 | 276 |
| 32 | 84 | 10.90909 | 76 | 0 | 276 |
| 33 | 0 | 1.188455 | 51 | 0 | 995 |
| 33 | 28 | 0.595829 | 51 | 0 | 995 |
| 33 | 114 | 0.558919 | 51 | 0 | 995 |
| 33 | 161 | 1.111111 | 51 | 0 | 995 |
| 33 | 231 | 0.436364 | 51 | 0 | 995 |
| 33 | 350 | 0.432432 | 51 | 0 | 995 |
| 33 | 406 | 1.376147 | 51 | 0 | 995 |
| 33 | 462 | 1.000834 | 51 | 0 | 995 |
| 33 | 539 | 1.02916 | 51 | 0 | 995 |
| 34 | 0 | 2.123894 | 60 | 0 | 729 |
| 34 | 98 | 0.78637 | 60 | 0 | 729 |
| 34 | 161 | 0.677966 | 60 | 0 | 729 |
| 34 | 231 | 1.428571 | 60 | 0 | 729 |
| 34 | 329 | 1.652893 | 60 | 0 | 729 |
| 34 | 427 | 2.807018 | 60 | 0 | 729 |
| 34 | 497 | 2.909091 | 60 | 0 | 729 |
| 34 | 581 | 2.022756 | 60 | 0 | 729 |
| 35 | 0 | 4.958678 | 69 | 0 | 820 |
| 35 | 28 | 4.83871 | 69 | 0 | 820 |
| 35 | 84 | 5.128205 | 69 | 0 | 820 |
| 35 | 168 | 10.08403 | 69 | 0 | 820 |
| 35 | 252 | 5.172414 | 69 | 0 | 820 |
| 35 | 336 | 5.263158 | 69 | 0 | 820 |
| 35 | 420 | 5.042017 | 69 | 0 | 820 |

| | | | | | |
|---|---|---|---|---|---|
| 35 | 504 | 2.318841 | 69 | 0 | 820 |
| 35 | 567 | 2.469136 | 69 | 0 | 820 |
| 36 | 0 | 6.956522 | 83 | 0 | 653 |
| 36 | 96 | 3.079179 | 83 | 0 | 653 |
| 36 | 185 | 3.880804 | 83 | 0 | 653 |
| 36 | 255 | 5.660377 | 83 | 0 | 653 |
| 36 | 360 | 6.122449 | 83 | 0 | 653 |
| 36 | 430 | 8.490566 | 83 | 0 | 653 |
| 36 | 486 | 8.256881 | 83 | 0 | 653 |
| 36 | 647 | 15.427 | 83 | 0 | 653 |
| 37 | 0 | 5 | 44 | 1 | 203 |
| 37 | 31 | 2.380952 | 44 | 1 | 203 |
| 37 | 91 | 26.08696 | 44 | 1 | 203 |
| 37 | 168 | 9.591778 | 44 | 1 | 203 |
| 38 | 0 | 8.910891 | 78 | 1 | 259 |
| 38 | 28 | 12.37113 | 78 | 1 | 259 |
| 38 | 56 | 20.37599 | 78 | 1 | 259 |
| 38 | 175 | 30.2521 | 78 | 1 | 259 |
| 38 | 257 | 5.660377 | 78 | 1 | 259 |
| 39 | 0 | 4.615385 | 80 | 0 | 1158 |
| 39 | 21 | 5.504587 | 80 | 0 | 1158 |
| 39 | 77 | 4.210526 | 80 | 0 | 1158 |
| 39 | 168 | 6.792453 | 80 | 0 | 1158 |
| 39 | 259 | 8.490566 | 80 | 0 | 1158 |
| 39 | 322 | 7.894737 | 80 | 0 | 1158 |
| 39 | 406 | 4.761905 | 80 | 0 | 1158 |
| 40 | 0 | 3.207331 | 83 | 0 | 1052 |
| 40 | 92 | 5.882353 | 83 | 0 | 1052 |
| 40 | 148 | 4.861111 | 83 | 0 | 1052 |
| 40 | 260 | 5.624372 | 83 | 0 | 1052 |
| 40 | 316 | 4.752475 | 83 | 0 | 1052 |
| 41 | 0 | 1.617251 | 75 | 0 | 764 |
| 41 | 35 | 1.428571 | 75 | 0 | 764 |
| 41 | 98 | 1.617251 | 75 | 0 | 764 |
| 42 | 0 | 2.376238 | 74 | 1 | 101 |
| 42 | 35 | 5.769231 | 74 | 1 | 101 |
| 42 | 91 | 12.96296 | 74 | 1 | 101 |
| 43 | 0 | 2 | 70 | 0 | 862 |
| 43 | 28 | 0.847458 | 70 | 0 | 862 |

| | | | | | |
|---|---|---|---|---|---|
| 43 | 56 | 0.892857 | 70 | 0 | 862 |
| 43 | 168 | 1.071429 | 70 | 0 | 862 |
| 43 | 266 | 2.631579 | 70 | 0 | 862 |
| 43 | 329 | 1.754386 | 70 | 0 | 862 |
| 43 | 413 | 1.428571 | 70 | 0 | 862 |
| 43 | 497 | 1.391304 | 70 | 0 | 862 |
| 43 | 560 | 1.754386 | 70 | 0 | 862 |
| 44 | 0 | 2.727273 | 86 | 0 | 232 |
| 44 | 28 | 2.654867 | 86 | 0 | 232 |
| 44 | 84 | 2.608696 | 86 | 0 | 232 |
| 44 | 175 | 3.809524 | 86 | 0 | 232 |
| 44 | 231 | 2.285714 | 86 | 0 | 232 |
| 45 | 0 | 3.030303 | 93 | 0 | 1044 |
| 45 | 28 | 1.333333 | 93 | 0 | 1044 |
| 45 | 91 | 2.962963 | 93 | 0 | 1044 |
| 45 | 154 | 1.052632 | 93 | 0 | 1044 |
| 45 | 224 | 1.052632 | 93 | 0 | 1044 |
| 45 | 308 | 5.825243 | 93 | 0 | 1044 |
| 45 | 392 | 4.571429 | 93 | 0 | 1044 |
| 45 | 483 | 5.217391 | 93 | 0 | 1044 |
| 45 | 581 | 4.571429 | 93 | 0 | 1044 |
| 46 | 14 | 3.539823 | 47 | 0 | 694 |
| 46 | 84 | 1.639344 | 47 | 0 | 694 |
| 46 | 154 | 1.03426 | 47 | 0 | 694 |
| 46 | 245 | 1.061008 | 47 | 0 | 694 |
| 46 | 336 | 1.017165 | 47 | 0 | 694 |
| 46 | 420 | 1.120448 | 47 | 0 | 694 |
| 46 | 511 | 1.008827 | 47 | 0 | 694 |
| 46 | 574 | 1.481481 | 47 | 0 | 694 |
| 47 | 0 | 3.305785 | 62 | 1 | 648 |
| 47 | 16 | 0.309461 | 62 | 1 | 648 |
| 47 | 92 | 0.369458 | 62 | 1 | 648 |
| 47 | 119 | 0.393553 | 62 | 1 | 648 |
| 47 | 245 | 0.362069 | 62 | 1 | 648 |
| 47 | 364 | 0.338382 | 62 | 1 | 648 |
| 47 | 422 | 0.335249 | 62 | 1 | 648 |
| 47 | 510 | 13.28063 | 62 | 1 | 648 |
| 48 | 0 | 5.263158 | 66 | 0 | 1212 |
| 48 | 35 | 3.448276 | 66 | 0 | 1212 |

| | | | | | |
|---|---|---|---|---|---|
| 48 | 84 | 4.878049 | 66 | 0 | 1212 |
| 48 | 140 | 2.4 | 66 | 0 | 1212 |
| 48 | 244 | 1.754386 | 66 | 0 | 1212 |
| 48 | 328 | 1.251303 | 66 | 0 | 1212 |
| 48 | 450 | 2.239403 | 66 | 0 | 1212 |
| 49 | 28 | 1.327434 | 82 | 0 | 1450 |
| 49 | 84 | 1.320132 | 82 | 0 | 1450 |
| 49 | 168 | 1.980198 | 82 | 0 | 1450 |
| 49 | 252 | 1.930813 | 82 | 0 | 1450 |
| 49 | 329 | 3.773585 | 82 | 0 | 1450 |
| 49 | 413 | 5.429864 | 82 | 0 | 1450 |
| 49 | 504 | 5.882353 | 82 | 0 | 1450 |
| 49 | 588 | 5.825243 | 82 | 0 | 1450 |
| 50 | 0 | 5.228758 | 55 | 0 | 757 |
| 50 | 28 | 9.142857 | 55 | 0 | 757 |
| 50 | 63 | 7.619048 | 55 | 0 | 757 |
| 50 | 133 | 0.558919 | 55 | 0 | 757 |
| 50 | 219 | 0.679117 | 55 | 0 | 757 |
| 50 | 294 | 0.725953 | 55 | 0 | 757 |
| 50 | 399 | 1.924619 | 55 | 0 | 757 |
| 50 | 602 | 1.724138 | 55 | 0 | 757 |
| 51 | 0 | 2.264151 | 68 | 0 | 911 |
| 51 | 56 | 1.886792 | 68 | 0 | 911 |
| 51 | 182 | 1.648352 | 68 | 0 | 911 |
| 51 | 224 | 1.818182 | 68 | 0 | 911 |
| 51 | 350 | 2.020202 | 68 | 0 | 911 |
| 51 | 392 | 2.438316 | 68 | 0 | 911 |
| 51 | 609 | 2.640264 | 68 | 0 | 911 |
| 52 | 0 | 5.890603 | 80 | 0 | 612 |
| 52 | 23 | 5.370844 | 80 | 0 | 612 |
| 53 | 0 | 4.033613 | 60 | 1 | 217 |
| 53 | 35 | 5.217391 | 60 | 1 | 217 |
| 53 | 63 | 5.309735 | 60 | 1 | 217 |
| 53 | 147 | 10.90909 | 60 | 1 | 217 |
| 54 | 0 | 4.6875 | 69 | 1 | 125 |
| 54 | 28 | 4.580153 | 69 | 1 | 125 |
| 55 | 0 | 3.804348 | 67 | 1 | 196 |
| 55 | 24 | 2.526316 | 67 | 1 | 196 |
| 55 | 87 | 5.783133 | 67 | 1 | 196 |

| | | | | | |
|---|---|---|---|---|---|
| 55 | 164 | 6.818182 | 67 | 1 | 196 |
| 56 | 0 | 1.652893 | 94 | 0 | 1029 |
| 56 | 28 | 1.801802 | 94 | 0 | 1029 |
| 56 | 84 | 1.994302 | 94 | 0 | 1029 |
| 56 | 157 | 1.403509 | 94 | 0 | 1029 |
| 56 | 255 | 3.010753 | 94 | 0 | 1029 |
| 56 | 325 | 1.37931 | 94 | 0 | 1029 |
| 56 | 430 | 1.724138 | 94 | 0 | 1029 |
| 56 | 514 | 1.801802 | 94 | 0 | 1029 |
| 56 | 601 | 2.463343 | 94 | 0 | 1029 |
| 57 | 0 | 1.018305 | 78 | 1 | 345 |
| 57 | 28 | 2.702703 | 78 | 1 | 345 |
| 57 | 84 | 3.393939 | 78 | 1 | 345 |
| 57 | 154 | 2.803738 | 78 | 1 | 345 |
| 57 | 238 | 2.727273 | 78 | 1 | 345 |
| 57 | 336 | 9.722222 | 78 | 1 | 345 |
| 58 | 0 | 4.615385 | 71 | 0 | 897 |
| 58 | 35 | 4.528302 | 71 | 0 | 897 |
| 58 | 98 | 2.654867 | 71 | 0 | 897 |
| 58 | 196 | 2.259887 | 71 | 0 | 897 |
| 58 | 256 | 4.615385 | 71 | 0 | 897 |
| 58 | 343 | 2.702703 | 71 | 0 | 897 |
| 58 | 427 | 6 | 71 | 0 | 897 |
| 58 | 518 | 6.315789 | 71 | 0 | 897 |
| 58 | 616 | 10.32258 | 71 | 0 | 897 |
| 59 | 0 | 7.2 | 84 | 0 | 953 |
| 59 | 28 | 4.123711 | 84 | 0 | 953 |
| 59 | 91 | 5.263158 | 84 | 0 | 953 |
| 59 | 147 | 12.5 | 84 | 0 | 953 |
| 59 | 224 | 12 | 84 | 0 | 953 |
| 59 | 294 | 18.75 | 84 | 0 | 953 |
| 59 | 413 | 16.51376 | 84 | 0 | 953 |
| 59 | 483 | 10.81081 | 84 | 0 | 953 |
| 59 | 567 | 21.81818 | 84 | 0 | 953 |
| 60 | 0 | 7.777778 | 69 | 1 | 242 |
| 60 | 42 | 13.72549 | 69 | 1 | 242 |
| 60 | 140 | 11.53846 | 69 | 1 | 242 |
| 61 | 0 | 1.269841 | 66 | 0 | 848 |
| 61 | 21 | 1.639344 | 66 | 0 | 848 |

| | | | | | |
|---|---|---|---|---|---|
| 61 | 77 | 1.785714 | 66 | 0 | 848 |
| 61 | 175 | 1.481481 | 66 | 0 | 848 |
| 61 | 238 | 1.785714 | 66 | 0 | 848 |
| 61 | 336 | 2.330097 | 66 | 0 | 848 |
| 61 | 427 | 5.940594 | 66 | 0 | 848 |
| 62 | 0 | 10.32258 | 77 | 0 | 666 |
| 62 | 30 | 20.16 | 77 | 0 | 666 |
| 62 | 56 | 16.66667 | 77 | 0 | 666 |
| 62 | 140 | 20.68966 | 77 | 0 | 666 |
| 62 | 231 | 15.63275 | 77 | 0 | 666 |
| 62 | 315 | 16.07143 | 77 | 0 | 666 |
| 62 | 381 | 16.66667 | 77 | 0 | 666 |
| 62 | 504 | 16.82243 | 77 | 0 | 666 |
| 62 | 602 | 10.90909 | 77 | 0 | 666 |
| 63 | 0 | 5.128205 | 79 | 0 | 589 |
| 63 | 28 | 5.128205 | 79 | 0 | 589 |
| 64 | 0 | 5.263158 | 49 | 1 | 563 |
| 64 | 28 | 5 | 49 | 1 | 563 |
| 64 | 91 | 7.407407 | 49 | 1 | 563 |
| 64 | 168 | 5.555556 | 49 | 1 | 563 |
| 64 | 252 | 5.714286 | 49 | 1 | 563 |
| 64 | 336 | 4.705882 | 49 | 1 | 563 |
| 64 | 441 | 4.444444 | 49 | 1 | 563 |
| 64 | 504 | 16.32653 | 49 | 1 | 563 |
| 65 | 21 | 9.677419 | 25 | 1 | 869 |
| 65 | 77 | 6.451613 | 25 | 1 | 869 |
| 65 | 161 | 10.25641 | 25 | 1 | 869 |
| 65 | 245 | 12.20339 | 25 | 1 | 869 |
| 65 | 322 | 11.70732 | 25 | 1 | 869 |
| 65 | 413 | 14.51613 | 25 | 1 | 869 |
| 65 | 504 | 7.328244 | 25 | 1 | 869 |
| 65 | 572 | 8.345753 | 25 | 1 | 869 |
| 66 | 0 | 2.884615 | 74 | 1 | 204 |
| 66 | 28 | 2.830189 | 74 | 1 | 204 |
| 66 | 91 | 6.122449 | 74 | 1 | 204 |
| 66 | 161 | 8 | 74 | 1 | 204 |
| 67 | 0 | 2.181818 | 67 | 1 | 384 |
| 67 | 35 | 1.5 | 67 | 1 | 384 |
| 67 | 91 | 2.702703 | 67 | 1 | 384 |

| | | | | | |
|---|---|---|---|---|---|
| 67 | 173 | 8 | 67 | 1 | 384 |
| 67 | 252 | 11.22807 | 67 | 1 | 384 |
| 67 | 343 | 9.195402 | 67 | 1 | 384 |
| 68 | 0 | 2.086957 | 65 | 0 | 1058 |
| 68 | 63 | 2.310231 | 65 | 0 | 1058 |
| 68 | 154 | 2.264151 | 65 | 0 | 1058 |
| 68 | 245 | 2.970297 | 65 | 0 | 1058 |
| 68 | 336 | 2.830189 | 65 | 0 | 1058 |
| 68 | 434 | 3.076923 | 65 | 0 | 1058 |
| 68 | 532 | 2.333333 | 65 | 0 | 1058 |
| 68 | 619 | 2.912621 | 65 | 0 | 1058 |
| 69 | 0 | 5.309735 | 73 | 1 | 420 |
| 69 | 28 | 5.843478 | 73 | 1 | 420 |
| 69 | 88 | 3.966942 | 73 | 1 | 420 |
| 69 | 123 | 4.671858 | 73 | 1 | 420 |
| 69 | 217 | 5.309735 | 73 | 1 | 420 |
| 69 | 315 | 8.256881 | 73 | 1 | 420 |
| 69 | 411 | 36.36364 | 73 | 1 | 420 |
| 70 | 0 | 1.181102 | 74 | 0 | 856 |
| 70 | 56 | 1.179941 | 74 | 0 | 856 |
| 70 | 140 | 1.149425 | 74 | 0 | 856 |
| 70 | 224 | 0.985222 | 74 | 0 | 856 |
| 70 | 322 | 2.242991 | 74 | 0 | 856 |
| 70 | 392 | 3.669725 | 74 | 0 | 856 |
| 70 | 476 | 3.508772 | 74 | 0 | 856 |
| 70 | 560 | 6 | 74 | 0 | 856 |
| 71 | 0 | 1.25 | 66 | 1 | 417 |
| 71 | 28 | 1.229508 | 66 | 1 | 417 |
| 71 | 84 | 1.081081 | 66 | 1 | 417 |
| 71 | 189 | 1.081081 | 66 | 1 | 417 |
| 71 | 247 | 0.611621 | 66 | 1 | 417 |
| 72 | 0 | 0.40678 | 87 | 0 | 655 |
| 72 | 29 | 0.390244 | 87 | 0 | 655 |
| 72 | 112 | 0.4 | 87 | 0 | 655 |
| 72 | 168 | 4.040404 | 87 | 0 | 655 |
| 72 | 290 | 0.474308 | 87 | 0 | 655 |
| 72 | 364 | 1.889339 | 87 | 0 | 655 |
| 72 | 535 | 0.925926 | 87 | 0 | 655 |
| 72 | 591 | 2.564103 | 87 | 0 | 655 |

| | | | | | |
|---|---|---|---|---|---|
| 73 | 0 | 5.517241 | 60 | 1 | 205 |
| 73 | 28 | 12.63158 | 60 | 1 | 205 |
| 73 | 56 | 13.09942 | 60 | 1 | 205 |
| 73 | 147 | 24.74227 | 60 | 1 | 205 |
| 74 | 0 | 1.327434 | 75 | 0 | 596 |
| 74 | 28 | 1.100917 | 75 | 0 | 596 |
| 74 | 63 | 0.952381 | 75 | 0 | 596 |
| 74 | 154 | 4.255319 | 75 | 0 | 596 |
| 74 | 238 | 8.510638 | 75 | 0 | 596 |
| 74 | 322 | 3.571429 | 75 | 0 | 596 |
| 74 | 432 | 12.5 | 75 | 0 | 596 |
| 74 | 509 | 7.476636 | 75 | 0 | 596 |
| 74 | 595 | 7.136788 | 75 | 0 | 596 |
| 75 | 0 | 9.142857 | 59 | 1 | 380 |
| 75 | 35 | 9.6 | 59 | 1 | 380 |
| 75 | 91 | 6.926407 | 59 | 1 | 380 |
| 75 | 175 | 10.6383 | 59 | 1 | 380 |
| 75 | 259 | 17.24138 | 59 | 1 | 380 |
| 75 | 350 | 10.69519 | 59 | 1 | 380 |
| 76 | 0 | 9.090909 | 71 | 1 | 79 |
| 76 | 28 | 12.2449 | 71 | 1 | 79 |
| 76 | 56 | 12 | 71 | 1 | 79 |
| 77 | 0 | 3.168317 | 66 | 0 | 680 |
| 77 | 35 | 2.654867 | 66 | 0 | 680 |
| 77 | 91 | 1.260504 | 66 | 0 | 680 |
| 77 | 182 | 2.727273 | 66 | 0 | 680 |
| 77 | 245 | 2.051282 | 66 | 0 | 680 |
| 77 | 343 | 0.696864 | 66 | 0 | 680 |
| 77 | 406 | 2.777778 | 66 | 0 | 680 |
| 77 | 504 | 3.137255 | 66 | 0 | 680 |
| 77 | 595 | 2.678571 | 66 | 0 | 680 |
| 78 | 0 | 0.265781 | 78 | 1 | 241 |
| 78 | 77 | 0.27907 | 78 | 1 | 241 |
| 78 | 154 | 0.290698 | 78 | 1 | 241 |
| 78 | 238 | 52.33645 | 78 | 1 | 241 |
| 79 | 0 | 2.654867 | 92 | 0 | 1086 |
| 79 | 35 | 9.266409 | 92 | 0 | 1086 |
| 79 | 126 | 8.50059 | 92 | 0 | 1086 |
| 79 | 224 | 6.015038 | 92 | 0 | 1086 |

| | | | | | |
|---|---|---|---|---|---|
| 79 | 315 | 3.183024 | 92 | 0 | 1086 |
| 79 | 406 | 3.034134 | 92 | 0 | 1086 |
| 79 | 504 | 1.633764 | 92 | 0 | 1086 |
| 79 | 595 | 3.729604 | 92 | 0 | 1086 |
| 80 | 0 | 3.478261 | 44 | 0 | 946 |
| 81 | 0 | 10.54945 | 83 | 0 | 470 |
| 81 | 35 | 17.02128 | 83 | 0 | 470 |
| 81 | 84 | 8.602151 | 83 | 0 | 470 |
| 81 | 168 | 14.68531 | 83 | 0 | 470 |
| 81 | 343 | 4 | 83 | 0 | 470 |
| 81 | 420 | 5.769231 | 83 | 0 | 470 |
| 81 | 469 | 6.896552 | 83 | 0 | 470 |
| 82 | 0 | 2.955665 | 75 | 0 | 1051 |
| 82 | 14 | 2.359882 | 75 | 0 | 1051 |
| 82 | 77 | 4.816514 | 75 | 0 | 1051 |
| 82 | 141 | 4.75382 | 75 | 0 | 1051 |
| 82 | 203 | 3.861625 | 75 | 0 | 1051 |
| 82 | 357 | 4.278075 | 75 | 0 | 1051 |
| 82 | 420 | 8.571429 | 75 | 0 | 1051 |
| 82 | 476 | 8.411215 | 75 | 0 | 1051 |
| 82 | 609 | 21.05263 | 75 | 0 | 1051 |
| 83 | 0 | 0.84686 | 83 | 0 | 645 |
| 83 | 28 | 1.5625 | 83 | 0 | 645 |
| 83 | 91 | 2.4 | 83 | 0 | 645 |
| 83 | 175 | 2.040816 | 83 | 0 | 645 |
| 83 | 259 | 4.040404 | 83 | 0 | 645 |
| 83 | 343 | 3.743316 | 83 | 0 | 645 |
| 83 | 427 | 2.020202 | 83 | 0 | 645 |
| 83 | 518 | 3.428571 | 83 | 0 | 645 |
| 83 | 602 | 4.166667 | 83 | 0 | 645 |
| 84 | 0 | 2.201835 | 78 | 0 | 948 |
| 84 | 35 | 2.608696 | 78 | 0 | 948 |
| 84 | 91 | 2.479339 | 78 | 0 | 948 |
| 84 | 203 | 2.439024 | 78 | 0 | 948 |
| 85 | 0 | 5.555556 | 84 | 0 | 1086 |
| 85 | 28 | 3.571429 | 84 | 0 | 1086 |
| 85 | 84 | 1.355932 | 84 | 0 | 1086 |
| 85 | 175 | 1.724138 | 84 | 0 | 1086 |
| 85 | 273 | 1.441441 | 84 | 0 | 1086 |

| | | | | | |
|---|---|---|---|---|---|
| 85 | 357 | 1.785714 | 84 | 0 | 1086 |
| 85 | 413 | 3.773585 | 84 | 0 | 1086 |
| 85 | 504 | 6.956522 | 84 | 0 | 1086 |
| 85 | 595 | 11.53846 | 84 | 0 | 1086 |
| 86 | 0 | 2.5 | 66 | 0 | 575 |
| 86 | 28 | 1.333333 | 66 | 0 | 575 |
| 86 | 98 | 1.322314 | 66 | 0 | 575 |
| 86 | 147 | 1.304348 | 66 | 0 | 575 |
| 86 | 231 | 3.478261 | 66 | 0 | 575 |
| 86 | 329 | 2.807018 | 66 | 0 | 575 |
| 86 | 433 | 2.41744 | 66 | 0 | 575 |
| 86 | 504 | 3.448276 | 66 | 0 | 575 |
| 86 | 574 | 2.424242 | 66 | 0 | 575 |
| 87 | 0 | 2.654867 | 75 | 0 | 1086 |
| 87 | 28 | 1.801802 | 75 | 0 | 1086 |
| 87 | 98 | 2.830189 | 75 | 0 | 1086 |
| 87 | 161 | 2.307692 | 75 | 0 | 1086 |
| 87 | 266 | 2.4 | 75 | 0 | 1086 |
| 87 | 336 | 2.285714 | 75 | 0 | 1086 |
| 87 | 406 | 2.285714 | 75 | 0 | 1086 |
| 87 | 511 | 5.660377 | 75 | 0 | 1086 |
| 87 | 609 | 4.752475 | 75 | 0 | 1086 |
| 88 | 0 | 4.558405 | 57 | 0 | 840 |
| 88 | 27 | 4.022989 | 57 | 0 | 840 |
| 88 | 84 | 3.368421 | 57 | 0 | 840 |
| 88 | 140 | 2.996255 | 57 | 0 | 840 |
| 88 | 266 | 7.228916 | 57 | 0 | 840 |
| 88 | 308 | 21.05263 | 57 | 0 | 840 |
| 88 | 420 | 62.06897 | 57 | 0 | 840 |
| 88 | 490 | 33.96226 | 57 | 0 | 840 |
| 89 | 0 | 2.654867 | 71 | 0 | 1081 |
| 89 | 28 | 1.92 | 71 | 0 | 1081 |
| 89 | 77 | 2.033898 | 71 | 0 | 1081 |
| 89 | 161 | 1.785714 | 71 | 0 | 1081 |
| 89 | 266 | 2.201835 | 71 | 0 | 1081 |
| 89 | 336 | 2.201835 | 71 | 0 | 1081 |
| 89 | 448 | 1.401168 | 71 | 0 | 1081 |
| 89 | 511 | 2.222222 | 71 | 0 | 1081 |
| 89 | 588 | 3.636364 | 71 | 0 | 1081 |

| | | | | | |
|---|---|---|---|---|---|
| 90 | 0 | 0.94451 | 85 | 0 | 819 |
| 90 | 91 | 1.558442 | 85 | 0 | 819 |
| 90 | 189 | 0.635593 | 85 | 0 | 819 |
| 90 | 252 | 1.257862 | 85 | 0 | 819 |
| 90 | 357 | 1.960784 | 85 | 0 | 819 |
| 90 | 420 | 2.242991 | 85 | 0 | 819 |
| 90 | 497 | 8.988764 | 85 | 0 | 819 |
| 90 | 574 | 18.94737 | 85 | 0 | 819 |
| 91 | 0 | 10.94225 | 48 | 0 | 827 |
| 91 | 28 | 17.64706 | 48 | 0 | 827 |
| 91 | 98 | 11.21495 | 48 | 0 | 827 |
| 91 | 140 | 3.296703 | 48 | 0 | 827 |
| 91 | 238 | 5.429864 | 48 | 0 | 827 |
| 91 | 329 | 5.325444 | 48 | 0 | 827 |
| 91 | 420 | 5.940594 | 48 | 0 | 827 |
| 91 | 504 | 5.377147 | 48 | 0 | 827 |
| 91 | 686 | 5.325444 | 48 | 0 | 827 |
| 92 | 0 | 12.76596 | 65 | 0 | 589 |
| 92 | 28 | 18.5567 | 65 | 0 | 589 |
| 92 | 84 | 15.65217 | 65 | 0 | 589 |
| 92 | 168 | 10.34483 | 65 | 0 | 589 |
| 92 | 252 | 5.263158 | 65 | 0 | 589 |
| 92 | 343 | 11.76471 | 65 | 0 | 589 |
| 92 | 434 | 10.16949 | 65 | 0 | 589 |
| 92 | 490 | 7.933884 | 65 | 0 | 589 |
| 92 | 588 | 7.868852 | 65 | 0 | 589 |
| 93 | 0 | 2.222222 | 77 | 0 | 680 |
| 93 | 35 | 6.060606 | 77 | 0 | 680 |
| 93 | 91 | 8.163265 | 77 | 0 | 680 |
| 93 | 168 | 2.328482 | 77 | 0 | 680 |
| 93 | 266 | 2.679426 | 77 | 0 | 680 |
| 94 | 0 | 1.034483 | 74 | 0 | 1348 |
| 94 | 35 | 2.941176 | 74 | 0 | 1348 |
| 94 | 91 | 6.122449 | 74 | 0 | 1348 |
| 94 | 175 | 5 | 74 | 0 | 1348 |
| 94 | 238 | 5.172414 | 74 | 0 | 1348 |
| 94 | 350 | 8.411215 | 74 | 0 | 1348 |
| 94 | 413 | 6.990291 | 74 | 0 | 1348 |
| 94 | 483 | 7.272727 | 74 | 0 | 1348 |

| | | | | | |
|---|---|---|---|---|---|
| 95 | 0 | 2.803738 | 79 | 1 | 274 |
| 95 | 28 | 5.882353 | 79 | 1 | 274 |
| 95 | 84 | 9.142857 | 79 | 1 | 274 |
| 95 | 182 | 9.320388 | 79 | 1 | 274 |
| 95 | 261 | 22.64151 | 79 | 1 | 274 |
| 96 | 0 | 3.358657 | 42 | 0 | 993 |
| 96 | 28 | 6.779661 | 42 | 0 | 993 |
| 96 | 70 | 3.692308 | 42 | 0 | 993 |
| 96 | 161 | 4.285714 | 42 | 0 | 993 |
| 96 | 245 | 5.263158 | 42 | 0 | 993 |
| 96 | 329 | 8.791209 | 42 | 0 | 993 |
| 97 | 0 | 4.786325 | 69 | 0 | 731 |
| 97 | 30 | 5.128205 | 69 | 0 | 731 |
| 97 | 86 | 5.172414 | 69 | 0 | 731 |
| 97 | 170 | 5.128205 | 69 | 0 | 731 |
| 97 | 254 | 5.172414 | 69 | 0 | 731 |
| 97 | 338 | 5.454545 | 69 | 0 | 731 |
| 97 | 422 | 5.309735 | 69 | 0 | 731 |
| 97 | 506 | 5.084746 | 69 | 0 | 731 |
| 97 | 590 | 5.042017 | 69 | 0 | 731 |
| 98 | 0 | 10.71429 | 75 | 1 | 295 |
| 98 | 28 | 5.084746 | 75 | 1 | 295 |
| 98 | 84 | 1.470588 | 75 | 1 | 295 |
| 98 | 161 | 1.319406 | 75 | 1 | 295 |
| 98 | 252 | 4.45269 | 75 | 1 | 295 |
| 99 | 0 | 5.769231 | 58 | 1 | 201 |
| 99 | 28 | 12.5 | 58 | 1 | 201 |
| 99 | 101 | 10.43478 | 58 | 1 | 201 |
| 99 | 136 | 8.971963 | 58 | 1 | 201 |
| 100 | 0 | 2.654867 | 72 | 0 | 974 |
| 100 | 28 | 2.542373 | 72 | 0 | 974 |
| 100 | 91 | 2.678571 | 72 | 0 | 974 |
| 100 | 147 | 2.181818 | 72 | 0 | 974 |
| 100 | 245 | 1.401869 | 72 | 0 | 974 |
| 100 | 336 | 6.060606 | 72 | 0 | 974 |
| 100 | 420 | 4.247788 | 72 | 0 | 974 |
| 100 | 483 | 5.357143 | 72 | 0 | 974 |
| 100 | 567 | 5.217391 | 72 | 0 | 974 |
| 101 | 0 | 9.102402 | 52 | 0 | 785 |

| | | | | | |
|---|---|---|---|---|---|
| 101 | 49 | 5.042017 | 52 | 0 | 785 |
| 101 | 91 | 5.172414 | 52 | 0 | 785 |
| 101 | 175 | 5.405405 | 52 | 0 | 785 |
| 101 | 273 | 4.173913 | 52 | 0 | 785 |
| 101 | 371 | 5.128205 | 52 | 0 | 785 |
| 101 | 413 | 11.11111 | 52 | 0 | 785 |
| 101 | 546 | 8.490566 | 52 | 0 | 785 |
| 101 | 595 | 10.71429 | 52 | 0 | 785 |
| 102 | 0 | 10.34483 | 71 | 1 | 109 |
| 102 | 28 | 5.172414 | 71 | 1 | 109 |
| 102 | 79 | 2.964008 | 71 | 1 | 109 |
| 103 | 0 | 0.854701 | 68 | 0 | 1143 |
| 103 | 28 | 0.701754 | 68 | 0 | 1143 |
| 103 | 91 | 1.769912 | 68 | 0 | 1143 |
| 103 | 147 | 2.153846 | 68 | 0 | 1143 |
| 103 | 256 | 0.884956 | 68 | 0 | 1143 |
| 103 | 340 | 0.903226 | 68 | 0 | 1143 |
| 103 | 424 | 2.803738 | 68 | 0 | 1143 |
| 103 | 508 | 0.900901 | 68 | 0 | 1143 |
| 103 | 588 | 0.28028 | 68 | 0 | 1143 |
| 104 | 0 | 1.010101 | 58 | 1 | 309 |
| 104 | 14 | 0.673401 | 58 | 1 | 309 |
| 104 | 98 | 0.600601 | 58 | 1 | 309 |
| 104 | 205 | 0.606061 | 58 | 1 | 309 |
| 105 | 0 | 0.705467 | 41 | 0 | 1262 |
| 105 | 35 | 1.415929 | 41 | 0 | 1262 |
| 105 | 70 | 1.169591 | 41 | 0 | 1262 |
| 105 | 140 | 0.714286 | 41 | 0 | 1262 |
| 105 | 238 | 1.37931 | 41 | 0 | 1262 |
| 105 | 301 | 1.851852 | 41 | 0 | 1262 |
| 105 | 392 | 1.73913 | 41 | 0 | 1262 |
| 105 | 490 | 1.709402 | 41 | 0 | 1262 |
| 105 | 581 | 1.724138 | 41 | 0 | 1262 |
| 106 | 0 | 2.727273 | 62 | 1 | 261 |
| 106 | 28 | 2.264151 | 62 | 1 | 261 |
| 106 | 84 | 5.504587 | 62 | 1 | 261 |
| 106 | 161 | 5.825243 | 62 | 1 | 261 |
| 106 | 252 | 15.38462 | 62 | 1 | 261 |
| 107 | 0 | 2.242991 | 30 | 1 | 242 |

| | | | | | |
|---|---|---|---|---|---|
| 107 | 35 | 2.857143 | 30 | 1 | 242 |
| 107 | 91 | 5.052632 | 30 | 1 | 242 |
| 107 | 232 | 13.18681 | 30 | 1 | 242 |
| 108 | 0 | 3.883495 | 87 | 0 | 960 |
| 108 | 28 | 5.274725 | 87 | 0 | 960 |
| 108 | 91 | 4.166667 | 87 | 0 | 960 |
| 108 | 147 | 6.336634 | 87 | 0 | 960 |
| 108 | 259 | 4.664723 | 87 | 0 | 960 |
| 108 | 350 | 10.10526 | 87 | 0 | 960 |
| 108 | 434 | 19.35484 | 87 | 0 | 960 |
| 108 | 522 | 21.44681 | 87 | 0 | 960 |
| 108 | 588 | 8.484848 | 87 | 0 | 960 |
| 109 | 0 | 3.061224 | 77 | 1 | 121 |
| 109 | 28 | 5.30303 | 77 | 1 | 121 |
| 109 | 89 | 13.63636 | 77 | 1 | 121 |
| 110 | 0 | 18 | 78 | 0 | 484 |
| 110 | 56 | 6.122449 | 78 | 0 | 484 |
| 110 | 140 | 14.11765 | 78 | 0 | 484 |
| 110 | 210 | 13.45794 | 78 | 0 | 484 |
| 111 | 0 | 2.194357 | 71 | 0 | 1355 |
| 111 | 33 | 5.504587 | 71 | 0 | 1355 |
| 111 | 75 | 2.123894 | 71 | 0 | 1355 |
| 111 | 187 | 1.843318 | 71 | 0 | 1355 |
| 111 | 243 | 9.302326 | 71 | 0 | 1355 |
| 111 | 334 | 4.285714 | 71 | 0 | 1355 |
| 111 | 411 | 5.106383 | 71 | 0 | 1355 |
| 111 | 502 | 7.76699 | 71 | 0 | 1355 |
| 111 | 600 | 4.615385 | 71 | 0 | 1355 |
| 112 | 0 | 5.313093 | 78 | 0 | 648 |
| 112 | 31 | 5.607477 | 78 | 0 | 648 |
| 112 | 84 | 5.714286 | 78 | 0 | 648 |
| 112 | 168 | 8.571429 | 78 | 0 | 648 |
| 112 | 249 | 13.18681 | 78 | 0 | 648 |
| 112 | 336 | 8.411215 | 78 | 0 | 648 |
| 112 | 420 | 7.758621 | 78 | 0 | 648 |
| 112 | 507 | 8.490566 | 78 | 0 | 648 |
| 112 | 591 | 8.333333 | 78 | 0 | 648 |
| 113 | 0 | 6 | 84 | 1 | 285 |
| 113 | 28 | 12.5 | 84 | 1 | 285 |

| | | | | | |
|---|---|---|---|---|---|
| 113 | 77 | 6.349206 | 84 | 1 | 285 |
| 113 | 168 | 12.12121 | 84 | 1 | 285 |
| 113 | 238 | 8.247423 | 84 | 1 | 285 |
| 114 | 0 | 2.330097 | 59 | 0 | 594 |
| 114 | 35 | 4.948454 | 59 | 0 | 594 |
| 114 | 105 | 5.052632 | 59 | 0 | 594 |
| 114 | 168 | 11.65049 | 59 | 0 | 594 |
| 114 | 266 | 13.04348 | 59 | 0 | 594 |
| 114 | 350 | 11.76471 | 59 | 0 | 594 |
| 114 | 420 | 8.495575 | 59 | 0 | 594 |
| 114 | 504 | 11.42857 | 59 | 0 | 594 |
| 114 | 560 | 11.32075 | 59 | 0 | 594 |
| 115 | 0 | 2.930403 | 67 | 0 | 217 |
| 115 | 98 | 7.619048 | 67 | 0 | 217 |
| 115 | 175 | 13.09091 | 67 | 0 | 217 |
| 116 | 0 | 2.376238 | 67 | 1 | 492 |
| 116 | 35 | 2.285714 | 67 | 1 | 492 |
| 116 | 70 | 2.123894 | 67 | 1 | 492 |
| 116 | 182 | 2.222222 | 67 | 1 | 492 |
| 116 | 252 | 4.897959 | 67 | 1 | 492 |
| 116 | 357 | 4.848485 | 67 | 1 | 492 |
| 116 | 427 | 1.401401 | 67 | 1 | 492 |
| 116 | 476 | 3.630078 | 67 | 1 | 492 |
| 117 | 0 | 2.654867 | 91 | 0 | 218 |
| 117 | 56 | 3.018868 | 91 | 0 | 218 |
| 117 | 133 | 1.111111 | 91 | 0 | 218 |
| 117 | 217 | 1.179941 | 91 | 0 | 218 |
| 118 | 0 | 3 | 84 | 0 | 715 |
| 118 | 56 | 3.225806 | 84 | 0 | 715 |
| 118 | 168 | 3.157895 | 84 | 0 | 715 |
| 118 | 280 | 3.225806 | 84 | 0 | 715 |
| 118 | 329 | 3.571429 | 84 | 0 | 715 |
| 118 | 427 | 3.296703 | 84 | 0 | 715 |
| 118 | 525 | 3.463203 | 84 | 0 | 715 |
| 118 | 574 | 4.481793 | 84 | 0 | 715 |
| 119 | 0 | 2.752294 | 35 | 0 | 1212 |
| 119 | 28 | 1.428571 | 35 | 0 | 1212 |
| 119 | 84 | 3.061224 | 35 | 0 | 1212 |
| 119 | 168 | 3 | 35 | 0 | 1212 |

| | | | | | |
|---|---|---|---|---|---|
| 119 | 252 | 2.608696 | 35 | 0 | 1212 |
| 119 | 336 | 2.702703 | 35 | 0 | 1212 |
| 119 | 420 | 3.809524 | 35 | 0 | 1212 |
| 119 | 504 | 3.448276 | 35 | 0 | 1212 |
| 120 | 0 | 3.024302 | 79 | 0 | 288 |
| 121 | 0 | 1.28 | 78 | 0 | 667 |
| 121 | 34 | 1.666667 | 78 | 0 | 667 |
| 121 | 90 | 1.259843 | 78 | 0 | 667 |
| 121 | 188 | 1.367521 | 78 | 0 | 667 |
| 121 | 258 | 1.46789 | 78 | 0 | 667 |
| 121 | 363 | 1.553398 | 78 | 0 | 667 |
| 121 | 440 | 2.962963 | 78 | 0 | 667 |
| 121 | 510 | 2.990654 | 78 | 0 | 667 |
| 121 | 615 | 2.909091 | 78 | 0 | 667 |
| 122 | 0 | 1.149425 | 84 | 0 | 942 |
| 122 | 28 | 2.678571 | 84 | 0 | 942 |
| 122 | 77 | 2.568807 | 84 | 0 | 942 |
| 122 | 189 | 1.904762 | 84 | 0 | 942 |
| 122 | 252 | 3.174603 | 84 | 0 | 942 |
| 122 | 336 | 5.405405 | 84 | 0 | 942 |
| 122 | 420 | 5.357143 | 84 | 0 | 942 |
| 122 | 493 | 5.454545 | 84 | 0 | 942 |
| 122 | 588 | 5.504587 | 84 | 0 | 942 |
| 123 | 0 | 2.586207 | 74 | 0 | 820 |
| 123 | 28 | 5.607477 | 74 | 0 | 820 |
| 123 | 84 | 7.964602 | 74 | 0 | 820 |
| 123 | 147 | 6.315789 | 74 | 0 | 820 |
| 123 | 252 | 13.45794 | 74 | 0 | 820 |
| 123 | 322 | 13.98058 | 74 | 0 | 820 |
| 123 | 395 | 11.43376 | 74 | 0 | 820 |
| 123 | 493 | 4.878049 | 74 | 0 | 820 |
| 123 | 581 | 4.724409 | 74 | 0 | 820 |
| 124 | 0 | 10.9098 | 89 | 1 | 98 |
| 124 | 29 | 2.912621 | 89 | 1 | 98 |
| 124 | 85 | 2.5 | 89 | 1 | 98 |
| 125 | 0 | 1.724138 | 77 | 1 | 235 |
| 125 | 28 | 0.779727 | 77 | 1 | 235 |
| 125 | 91 | 0.854701 | 77 | 1 | 235 |
| 125 | 200 | 0.498798 | 77 | 1 | 235 |

| | | | | | |
|---|---|---|---|---|---|
| 125 | 231 | 5.313093 | 77 | 1 | 235 |
| 126 | 0 | 2.830189 | 79 | 0 | 1065 |
| 126 | 28 | 2.884615 | 79 | 0 | 1065 |
| 126 | 84 | 2.857143 | 79 | 0 | 1065 |
| 126 | 175 | 2.702703 | 79 | 0 | 1065 |
| 126 | 266 | 2.802803 | 79 | 0 | 1065 |
| 126 | 336 | 2.521008 | 79 | 0 | 1065 |
| 126 | 448 | 2.777778 | 79 | 0 | 1065 |
| 126 | 539 | 2.727273 | 79 | 0 | 1065 |
| 126 | 567 | 2.5 | 79 | 0 | 1065 |
| 127 | 0 | 6.060606 | 67 | 0 | 632 |
| 127 | 28 | 5.825243 | 67 | 0 | 632 |
| 127 | 56 | 4.897959 | 67 | 0 | 632 |
| 127 | 126 | 6.521739 | 67 | 0 | 632 |
| 127 | 238 | 6.976744 | 67 | 0 | 632 |
| 127 | 294 | 5.052632 | 67 | 0 | 632 |
| 127 | 371 | 6.25 | 67 | 0 | 632 |
| 127 | 462 | 6.185567 | 67 | 0 | 632 |
| 128 | 0 | 2.586207 | 61 | 0 | 596 |
| 128 | 28 | 2.608696 | 61 | 0 | 596 |
| 128 | 84 | 2.586207 | 61 | 0 | 596 |
| 128 | 175 | 2.777778 | 61 | 0 | 596 |
| 128 | 266 | 2.307692 | 61 | 0 | 596 |
| 128 | 329 | 5.504587 | 61 | 0 | 596 |
| 128 | 455 | 4.528302 | 61 | 0 | 596 |
| 128 | 511 | 6 | 61 | 0 | 596 |
| 128 | 595 | 12.76596 | 61 | 0 | 596 |
| 129 | 0 | 1.153846 | 49 | 0 | 99 |
| 129 | 35 | 1.043478 | 49 | 0 | 99 |
| 129 | 70 | 1.043478 | 49 | 0 | 99 |
| 130 | 0 | 5.128205 | 67 | 0 | 785 |
| 130 | 28 | 5.454545 | 67 | 0 | 785 |
| 130 | 84 | 5.405405 | 67 | 0 | 785 |
| 130 | 168 | 5.405405 | 67 | 0 | 785 |
| 130 | 252 | 5.357143 | 67 | 0 | 785 |
| 130 | 336 | 5.504587 | 67 | 0 | 785 |
| 130 | 420 | 5.607477 | 67 | 0 | 785 |
| 130 | 504 | 6.060606 | 67 | 0 | 785 |
| 130 | 588 | 5.940594 | 67 | 0 | 785 |

| | | | | | |
|---|---|---|---|---|---|
| 131 | 0 | 2.016807 | 72 | 0 | 522 |
| 131 | 35 | 2.142857 | 72 | 0 | 522 |
| 131 | 70 | 2.521008 | 72 | 0 | 522 |
| 131 | 182 | 1.46087 | 72 | 0 | 522 |
| 131 | 280 | 2.040816 | 72 | 0 | 522 |
| 131 | 336 | 5.660377 | 72 | 0 | 522 |
| 131 | 399 | 0.9908 | 72 | 0 | 522 |
| 132 | 0 | 2.962963 | 85 | 1 | 291 |
| 132 | 35 | 3.603604 | 85 | 1 | 291 |
| 132 | 103 | 8.15534 | 85 | 1 | 291 |
| 132 | 168 | 11.32075 | 85 | 1 | 291 |
| 132 | 250 | 17.14286 | 85 | 1 | 291 |
| 133 | 0 | 45 | 68 | 1 | 35 |
| 133 | 27 | 81.81818 | 68 | 1 | 35 |
| 134 | 0 | 2.068966 | 65 | 0 | 725 |
| 134 | 21 | 2.162162 | 65 | 0 | 725 |
| 134 | 91 | 2.264151 | 65 | 0 | 725 |
| 134 | 175 | 5.555556 | 65 | 0 | 725 |
| 134 | 231 | 5.405405 | 65 | 0 | 725 |
| 134 | 315 | 4.173913 | 65 | 0 | 725 |
| 134 | 406 | 5.405405 | 65 | 0 | 725 |
| 134 | 497 | 5.217391 | 65 | 0 | 725 |
| 134 | 553 | 5.825243 | 65 | 0 | 725 |
| 135 | 0 | 5.925926 | 31 | 1 | 67 |
| 135 | 35 | 3.116883 | 31 | 1 | 67 |
| 136 | 0 | 3.883495 | 68 | 1 | 737 |
| 136 | 28 | 3.809524 | 68 | 1 | 737 |
| 136 | 84 | 3.883495 | 68 | 1 | 737 |
| 136 | 168 | 12.2449 | 68 | 1 | 737 |
| 136 | 252 | 10.90909 | 68 | 1 | 737 |
| 136 | 336 | 11.21495 | 68 | 1 | 737 |
| 136 | 420 | 11.65049 | 68 | 1 | 737 |
| 137 | 0 | 5.607477 | 67 | 0 | 1245 |
| 137 | 28 | 3.809524 | 67 | 0 | 1245 |
| 137 | 84 | 7.54717 | 67 | 0 | 1245 |
| 137 | 168 | 7.272727 | 67 | 0 | 1245 |
| 137 | 252 | 9.090909 | 67 | 0 | 1245 |
| 137 | 336 | 10.34483 | 67 | 0 | 1245 |
| 137 | 420 | 10.81081 | 67 | 0 | 1245 |

| | | | | | |
|---|---|---|---|---|---|
| 137 | 504 | 10.16949 | 67 | 0 | 1245 |
| 137 | 588 | 2.290076 | 67 | 0 | 1245 |
| 138 | 0 | 1.785714 | 64 | 0 | 1030 |
| 138 | 38 | 1.851852 | 64 | 0 | 1030 |
| 138 | 164 | 1.769912 | 64 | 0 | 1030 |
| 138 | 262 | 1.315789 | 64 | 0 | 1030 |
| 138 | 359 | 1.25937 | 64 | 0 | 1030 |
| 138 | 423 | 1.193182 | 64 | 0 | 1030 |
| 138 | 472 | 1.081081 | 64 | 0 | 1030 |
| 138 | 542 | 1.034483 | 64 | 0 | 1030 |
| 139 | 0 | 1.259937 | 65 | 1 | 373 |
| 139 | 28 | 2.586207 | 65 | 1 | 373 |
| 139 | 91 | 2.857143 | 65 | 1 | 373 |
| 139 | 147 | 1.818182 | 65 | 1 | 373 |
| 139 | 252 | 3.092784 | 65 | 1 | 373 |
| 139 | 372 | 5.219012 | 65 | 1 | 373 |
| 140 | 0 | 5.405405 | 70 | 0 | 1079 |
| 140 | 28 | 5.405405 | 70 | 0 | 1079 |
| 140 | 84 | 5.405405 | 70 | 0 | 1079 |
| 140 | 175 | 4.485981 | 70 | 0 | 1079 |
| 140 | 231 | 5.555556 | 70 | 0 | 1079 |
| 140 | 315 | 4.173913 | 70 | 0 | 1079 |
| 140 | 427 | 3.539823 | 70 | 0 | 1079 |
| 140 | 511 | 3.703704 | 70 | 0 | 1079 |
| 140 | 595 | 3.636364 | 70 | 0 | 1079 |
| 141 | 0 | 1.190476 | 68 | 1 | 1025 |
| 141 | 91 | 1.058201 | 68 | 1 | 1025 |
| 141 | 189 | 1.020408 | 68 | 1 | 1025 |
| 141 | 238 | 1.029601 | 68 | 1 | 1025 |
| 141 | 329 | 1.234568 | 68 | 1 | 1025 |
| 141 | 448 | 1.223242 | 68 | 1 | 1025 |
| 141 | 539 | 1.048493 | 68 | 1 | 1025 |
| 141 | 595 | 0.980392 | 68 | 1 | 1025 |
| 142 | 0 | 2.362205 | 69 | 1 | 835 |
| 142 | 28 | 1.162791 | 69 | 1 | 835 |
| 142 | 84 | 0.8 | 69 | 1 | 835 |
| 142 | 168 | 2.640264 | 69 | 1 | 835 |
| 142 | 252 | 3.669725 | 69 | 1 | 835 |
| 142 | 336 | 8.421053 | 69 | 1 | 835 |

| | | | | | |
|---|---|---|---|---|---|
| 142 | 497 | 1.680672 | 69 | 1 | 835 |
| 142 | 581 | 3.960396 | 69 | 1 | 835 |
| 143 | 0 | 12.45829 | 70 | 0 | 1023 |
| 143 | 29 | 16 | 70 | 0 | 1023 |
| 143 | 92 | 20 | 70 | 0 | 1023 |
| 143 | 120 | 26.37363 | 70 | 0 | 1023 |
| 143 | 245 | 12.37113 | 70 | 0 | 1023 |
| 143 | 337 | 12 | 70 | 0 | 1023 |
| 143 | 421 | 16.94118 | 70 | 0 | 1023 |
| 143 | 491 | 20.22472 | 70 | 0 | 1023 |
| 143 | 561 | 15.18987 | 70 | 0 | 1023 |
| 144 | 0 | 3.571429 | 74 | 0 | 146 |
| 144 | 28 | 2.339181 | 74 | 0 | 146 |
| 144 | 91 | 3.636364 | 74 | 0 | 146 |
| 145 | 0 | 1.558442 | 73 | 0 | 675 |
| 146 | 0 | 1.376147 | 70 | 0 | 701 |
| 146 | 119 | 1.246106 | 70 | 0 | 701 |
| 146 | 175 | 2.4 | 70 | 0 | 701 |
| 146 | 245 | 1.960784 | 70 | 0 | 701 |
| 146 | 329 | 1.572739 | 70 | 0 | 701 |
| 146 | 427 | 2.803738 | 70 | 0 | 701 |
| 146 | 483 | 2.918187 | 70 | 0 | 701 |
| 147 | 0 | 4.067797 | 81 | 0 | 775 |
| 147 | 35 | 4.8 | 81 | 0 | 775 |
| 147 | 91 | 1.983471 | 81 | 0 | 775 |
| 147 | 154 | 1.801802 | 81 | 0 | 775 |
| 147 | 252 | 2.242991 | 81 | 0 | 775 |
| 147 | 329 | 2.912621 | 81 | 0 | 775 |
| 147 | 406 | 2.12766 | 81 | 0 | 775 |
| 147 | 511 | 3.092784 | 81 | 0 | 775 |
| 147 | 595 | 6.976744 | 81 | 0 | 775 |
| 148 | 0 | 1.977401 | 88 | 0 | 918 |
| 148 | 28 | 1.260504 | 88 | 0 | 918 |
| 148 | 84 | 1.818182 | 88 | 0 | 918 |
| 148 | 140 | 1.709402 | 88 | 0 | 918 |
| 148 | 252 | 0.826446 | 88 | 0 | 918 |
| 148 | 308 | 0.833333 | 88 | 0 | 918 |
| 148 | 420 | 1.801802 | 88 | 0 | 918 |
| 149 | 0 | 1.008403 | 77 | 0 | 976 |

| | | | | | |
|---|---|---|---|---|---|
| 149 | 30 | 2.264151 | 77 | 0 | 976 |
| 149 | 92 | 2.181818 | 77 | 0 | 976 |
| 149 | 184 | 1.886792 | 77 | 0 | 976 |
| 149 | 254 | 2.285714 | 77 | 0 | 976 |
| 149 | 352 | 2.727273 | 77 | 0 | 976 |
| 149 | 415 | 2.201835 | 77 | 0 | 976 |
| 149 | 513 | 2.777778 | 77 | 0 | 976 |
| 149 | 596 | 2.086957 | 77 | 0 | 976 |
| 150 | 0 | 5.263158 | 75 | 0 | 736 |
| 150 | 28 | 3.125 | 75 | 0 | 736 |
| 150 | 70 | 4.713805 | 75 | 0 | 736 |
| 150 | 140 | 6.185567 | 75 | 0 | 736 |
| 150 | 224 | 5.555556 | 75 | 0 | 736 |
| 150 | 294 | 6.185567 | 75 | 0 | 736 |
| 150 | 392 | 6.060606 | 75 | 0 | 736 |
| 150 | 469 | 14.28571 | 75 | 0 | 736 |
| 150 | 609 | 21.23894 | 75 | 0 | 736 |
| 151 | 0 | 3.703704 | 73 | 1 | 1238 |
| 151 | 21 | 2.201835 | 73 | 1 | 1238 |
| 151 | 84 | 5.714286 | 73 | 1 | 1238 |
| 151 | 140 | 7.706422 | 73 | 1 | 1238 |
| 151 | 266 | 5.309735 | 73 | 1 | 1238 |
| 151 | 356 | 3.921569 | 73 | 1 | 1238 |
| 151 | 419 | 9.836066 | 73 | 1 | 1238 |
| 152 | 0 | 3.333333 | 66 | 0 | 827 |
| 152 | 91 | 3.333333 | 66 | 0 | 827 |
| 152 | 189 | 2.666667 | 66 | 0 | 827 |
| 152 | 245 | 1.626016 | 66 | 0 | 827 |
| 152 | 336 | 2.711864 | 66 | 0 | 827 |
| 152 | 420 | 4.958678 | 66 | 0 | 827 |
| 152 | 511 | 4.067797 | 66 | 0 | 827 |
| 152 | 595 | 5.084746 | 66 | 0 | 827 |
| 153 | 0 | 6.545455 | 73 | 0 | 1081 |
| 153 | 30 | 12.41379 | 73 | 0 | 1081 |
| 153 | 65 | 11.52 | 73 | 0 | 1081 |
| 153 | 156 | 10.90909 | 73 | 0 | 1081 |
| 153 | 261 | 3.603604 | 73 | 0 | 1081 |
| 153 | 328 | 13.33333 | 73 | 0 | 1081 |
| 153 | 408 | 11.80328 | 73 | 0 | 1081 |

| | | | | | |
|---|---|---|---|---|---|
| 153 | 492 | 14.28571 | 73 | 0 | 1081 |
| 153 | 548 | 15.12605 | 73 | 0 | 1081 |
| 154 | 0 | 2.34375 | 79 | 0 | 581 |
| 154 | 28 | 1.99241 | 79 | 0 | 581 |
| 154 | 90 | 2.479339 | 79 | 0 | 581 |
| 154 | 167 | 2.222222 | 79 | 0 | 581 |
| 154 | 244 | 2.881152 | 79 | 0 | 581 |
| 154 | 336 | 6.956522 | 79 | 0 | 581 |
| 154 | 406 | 7.619048 | 79 | 0 | 581 |
| 154 | 524 | 5.058717 | 79 | 0 | 581 |
| 154 | 580 | 3.934426 | 79 | 0 | 581 |
| 155 | 0 | 2.352941 | 72 | 0 | 426 |
| 155 | 35 | 3.365385 | 72 | 0 | 426 |
| 155 | 87 | 3.218391 | 72 | 0 | 426 |
| 155 | 182 | 4.067797 | 72 | 0 | 426 |
| 155 | 245 | 2.727273 | 72 | 0 | 426 |
| 155 | 350 | 8.337469 | 72 | 0 | 426 |
| 156 | 0 | 1.754386 | 68 | 1 | 735 |
| 156 | 42 | 1.442308 | 68 | 1 | 735 |
| 156 | 189 | 3.939962 | 68 | 1 | 735 |
| 156 | 273 | 7.560756 | 68 | 1 | 735 |
| 156 | 329 | 4.639175 | 68 | 1 | 735 |
| 156 | 448 | 6.923077 | 68 | 1 | 735 |
| 156 | 513 | 9.545455 | 68 | 1 | 735 |
| 156 | 549 | 8.035714 | 68 | 1 | 735 |
| 157 | 0 | 2.439024 | 59 | 0 | 1018 |
| 157 | 28 | 1.904762 | 59 | 0 | 1018 |
| 157 | 91 | 3.539823 | 59 | 0 | 1018 |
| 157 | 168 | 2.830189 | 59 | 0 | 1018 |
| 157 | 252 | 1.084011 | 59 | 0 | 1018 |
| 157 | 342 | 0.826446 | 59 | 0 | 1018 |
| 157 | 427 | 1.293103 | 59 | 0 | 1018 |
| 157 | 511 | 2.727273 | 59 | 0 | 1018 |
| 157 | 566 | 2.727273 | 59 | 0 | 1018 |
| 158 | 0 | 3.571429 | 95 | 0 | 769 |
| 158 | 28 | 5.363985 | 95 | 0 | 769 |
| 158 | 77 | 4.363636 | 95 | 0 | 769 |
| 158 | 168 | 1.15942 | 95 | 0 | 769 |
| 158 | 252 | 1.960784 | 95 | 0 | 769 |

| | | | | | |
|---|---|---|---|---|---|
| 158 | 308 | 9.375 | 95 | 0 | 769 |
| 158 | 448 | 7.627119 | 95 | 0 | 769 |
| 158 | 511 | 5.714286 | 95 | 0 | 769 |
| 159 | 0 | 3.960396 | 89 | 0 | 233 |
| 159 | 28 | 6.273338 | 89 | 0 | 233 |
| 160 | 0 | 2.325581 | 70 | 0 | 673 |
| 160 | 112 | 1.87291 | 70 | 0 | 673 |
| 160 | 168 | 0.833333 | 70 | 0 | 673 |
| 160 | 224 | 1.415929 | 70 | 0 | 673 |
| 160 | 336 | 0.925926 | 70 | 0 | 673 |
| 160 | 448 | 1.25 | 70 | 0 | 673 |
| 160 | 504 | 1.648352 | 70 | 0 | 673 |
| 160 | 616 | 2.941176 | 70 | 0 | 673 |
| 161 | 0 | 2.380952 | 74 | 0 | 976 |
| 161 | 84 | 6 | 74 | 0 | 976 |
| 161 | 175 | 5.357143 | 74 | 0 | 976 |
| 161 | 245 | 2.279202 | 74 | 0 | 976 |
| 161 | 329 | 8.727273 | 74 | 0 | 976 |
| 161 | 413 | 13.91304 | 74 | 0 | 976 |
| 161 | 525 | 14.41441 | 74 | 0 | 976 |
| 161 | 595 | 4.285714 | 74 | 0 | 976 |
| 162 | 0 | 1.37457 | 77 | 1 | 159 |
| 162 | 42 | 2.727273 | 77 | 1 | 159 |
| 162 | 112 | 3.428571 | 77 | 1 | 159 |
| 163 | 28 | 1.239669 | 78 | 1 | 170 |
| 163 | 84 | 2.912621 | 78 | 1 | 170 |
| 163 | 168 | 5.825243 | 78 | 1 | 170 |
| 164 | 0 | 11.32075 | 60 | 1 | 820 |
| 164 | 28 | 12.2449 | 60 | 1 | 820 |
| 164 | 84 | 37.1134 | 60 | 1 | 820 |
| 164 | 182 | 6.666667 | 60 | 1 | 820 |
| 164 | 245 | 9.142857 | 60 | 1 | 820 |
| 164 | 371 | 8.067227 | 60 | 1 | 820 |
| 164 | 427 | 10.16949 | 60 | 1 | 820 |
| 164 | 511 | 14.11765 | 60 | 1 | 820 |
| 164 | 574 | 17.47573 | 60 | 1 | 820 |
| 165 | 0 | 4.761905 | 47 | 0 | 1030 |
| 165 | 28 | 1.726619 | 47 | 0 | 1030 |
| 166 | 0 | 10.99237 | 57 | 0 | 1124 |

| | | | | | |
|---|---|---|---|---|---|
| 166 | 35 | 11.33858 | 57 | 0 | 1124 |
| 166 | 70 | 9.836066 | 57 | 0 | 1124 |
| 166 | 168 | 4 | 57 | 0 | 1124 |
| 167 | 0 | 3.888889 | 80 | 0 | 967 |
| 167 | 30 | 6.690562 | 80 | 0 | 967 |
| 167 | 57 | 20.45455 | 80 | 0 | 967 |
| 167 | 149 | 20.68966 | 80 | 0 | 967 |
| 167 | 240 | 12 | 80 | 0 | 967 |
| 167 | 296 | 9.230769 | 80 | 0 | 967 |
| 167 | 394 | 3.75 | 80 | 0 | 967 |
| 167 | 506 | 5.217391 | 80 | 0 | 967 |
| 167 | 562 | 4.83871 | 80 | 0 | 967 |
| 168 | 0 | 5.504587 | 73 | 1 | 166 |
| 168 | 28 | 11.42857 | 73 | 1 | 166 |
| 168 | 91 | 11.88119 | 73 | 1 | 166 |
| 169 | 0 | 2.654867 | 76 | 0 | 693 |
| 169 | 84 | 1.320132 | 76 | 0 | 693 |
| 169 | 126 | 0.961538 | 76 | 0 | 693 |
| 169 | 231 | 1.143791 | 76 | 0 | 693 |
| 169 | 349 | 1.2 | 76 | 0 | 693 |
| 169 | 412 | 1.246106 | 76 | 0 | 693 |
| 169 | 538 | 2.564103 | 76 | 0 | 693 |
| 169 | 615 | 2.346041 | 76 | 0 | 693 |
| 170 | 0 | 1.652893 | 74 | 0 | 841 |
| 170 | 28 | 1.612903 | 74 | 0 | 841 |
| 170 | 84 | 0.378788 | 74 | 0 | 841 |
| 170 | 166 | 0.208333 | 74 | 0 | 841 |
| 171 | 0 | 0.328022 | 81 | 0 | 766 |
| 171 | 28 | 0.313761 | 81 | 0 | 766 |
| 171 | 105 | 0.285802 | 81 | 0 | 766 |
| 171 | 258 | 0.307085 | 81 | 0 | 766 |
| 171 | 342 | 0.291576 | 81 | 0 | 766 |
| 171 | 441 | 0.291576 | 81 | 0 | 766 |
| 171 | 476 | 0.310387 | 81 | 0 | 766 |
| 171 | 560 | 0.160367 | 81 | 0 | 766 |
| 172 | 0 | 0.980392 | 60 | 1 | 473 |
| 172 | 42 | 1.926164 | 60 | 1 | 473 |
| 172 | 98 | 1.981132 | 60 | 1 | 473 |
| 172 | 182 | 1.649485 | 60 | 1 | 473 |

| | | | | | |
|---|---|---|---|---|---|
| 172 | 266 | 1.538462 | 60 | 1 | 473 |
| 173 | 0 | 5.925926 | 79 | 0 | 771 |
| 173 | 105 | 9.677419 | 79 | 0 | 771 |
| 173 | 182 | 14.03509 | 79 | 0 | 771 |
| 173 | 259 | 5.228758 | 79 | 0 | 771 |
| 174 | 0 | 4.285714 | 53 | 0 | 691 |
| 174 | 35 | 2.224576 | 53 | 0 | 691 |
| 174 | 99 | 2.5 | 53 | 0 | 691 |
| 174 | 182 | 1.909525 | 53 | 0 | 691 |
| 174 | 238 | 7.660739 | 53 | 0 | 691 |
| 174 | 350 | 5.042017 | 53 | 0 | 691 |
| 174 | 385 | 1.99241 | 53 | 0 | 691 |
| 174 | 504 | 2.644628 | 53 | 0 | 691 |
| 174 | 616 | 5.972272 | 53 | 0 | 691 |
| 175 | 0 | 11.53846 | 86 | 0 | 520 |
| 175 | 28 | 18 | 86 | 0 | 520 |
| 175 | 84 | 16.82243 | 86 | 0 | 520 |
| 175 | 168 | 21.23894 | 86 | 0 | 520 |
| 175 | 238 | 15.38462 | 86 | 0 | 520 |
| 176 | 0 | 5.454545 | 71 | 0 | 610 |
| 176 | 28 | 2.037351 | 71 | 0 | 610 |
| 176 | 98 | 2.196796 | 71 | 0 | 610 |
| 176 | 161 | 5.280528 | 71 | 0 | 610 |
| 176 | 224 | 4.761905 | 71 | 0 | 610 |
| 176 | 343 | 4.285714 | 71 | 0 | 610 |
| 176 | 413 | 5 | 71 | 0 | 610 |
| 176 | 469 | 2.016807 | 71 | 0 | 610 |
| 176 | 609 | 2.201835 | 71 | 0 | 610 |
| 177 | 0 | 18.5567 | 41 | 0 | 953 |
| 177 | 28 | 15.65217 | 41 | 0 | 953 |
| 177 | 63 | 14.54545 | 41 | 0 | 953 |
| 178 | 0 | 2.4 | 57 | 1 | 154 |
| 178 | 91 | 5.217391 | 57 | 1 | 154 |
| 178 | 147 | 6.976744 | 57 | 1 | 154 |
| 179 | 0 | 10.08403 | 65 | 1 | 679 |
| 179 | 28 | 10.08403 | 65 | 1 | 679 |
| 179 | 84 | 10.16949 | 65 | 1 | 679 |
| 179 | 168 | 9.6 | 65 | 1 | 679 |
| 179 | 250 | 10.34483 | 65 | 1 | 679 |

| | | | | | |
|---|---|---|---|---|---|
| 179 | 334 | 10.43478 | 65 | 1 | 679 |
| 179 | 418 | 8.648649 | 65 | 1 | 679 |
| 179 | 509 | 10.71429 | 65 | 1 | 679 |
| 179 | 570 | 15.09434 | 65 | 1 | 679 |
| 180 | 0 | 9.230769 | 69 | 0 | 78 |
| 181 | 0 | 0.781832 | 49 | 0 | 699 |
| 181 | 42 | 5.172414 | 49 | 0 | 699 |
| 181 | 112 | 3.361345 | 49 | 0 | 699 |
| 181 | 168 | 1.088929 | 49 | 0 | 699 |
| 181 | 261 | 3.650587 | 49 | 0 | 699 |
| 181 | 329 | 1.816216 | 49 | 0 | 699 |
| 181 | 413 | 2.105263 | 49 | 0 | 699 |
| 181 | 504 | 0.952381 | 49 | 0 | 699 |
| 181 | 590 | 1.751094 | 49 | 0 | 699 |
| 182 | 0 | 1.190476 | 63 | 1 | 100 |
| 182 | 28 | 1.201201 | 63 | 1 | 100 |
| 182 | 91 | 3.125 | 63 | 1 | 100 |
| 183 | 0 | 3.703704 | 61 | 1 | 418 |
| 183 | 42 | 6.315789 | 61 | 1 | 418 |
| 183 | 98 | 12.12121 | 61 | 1 | 418 |
| 183 | 182 | 8.347826 | 61 | 1 | 418 |
| 183 | 273 | 12.5 | 61 | 1 | 418 |
| 183 | 357 | 10.66667 | 61 | 1 | 418 |
| 184 | 0 | 2.912621 | 73 | 1 | 368 |
| 184 | 28 | 6 | 73 | 1 | 368 |
| 184 | 81 | 10.08101 | 73 | 1 | 368 |
| 184 | 163 | 22.45989 | 73 | 1 | 368 |
| 184 | 274 | 18.33061 | 73 | 1 | 368 |
| 185 | 0 | 2.735043 | 59 | 0 | 1142 |
| 185 | 70 | 3.478261 | 59 | 0 | 1142 |
| 185 | 168 | 3.478261 | 59 | 0 | 1142 |
| 185 | 231 | 8 | 59 | 0 | 1142 |
| 185 | 315 | 3.603604 | 59 | 0 | 1142 |
| 185 | 406 | 3.478261 | 59 | 0 | 1142 |
| 185 | 483 | 3.478261 | 59 | 0 | 1142 |
| 185 | 574 | 3.773585 | 59 | 0 | 1142 |
| 186 | 0 | 11.32075 | 68 | 0 | 938 |
| 186 | 77 | 17.30769 | 68 | 0 | 938 |
| 186 | 189 | 10.71429 | 68 | 0 | 938 |

| | | | | | |
|---|---|---|---|---|---|
| 186 | 254 | 14.58333 | 68 | 0 | 938 |
| 186 | 345 | 16.21622 | 68 | 0 | 938 |
| 186 | 426 | 15.51724 | 68 | 0 | 938 |
| 186 | 527 | 13.33333 | 68 | 0 | 938 |
| 186 | 583 | 14.11765 | 68 | 0 | 938 |
| 187 | 0 | 2.702703 | 66 | 0 | 736 |
| 187 | 91 | 2.777778 | 66 | 0 | 736 |
| 187 | 161 | 2.830189 | 66 | 0 | 736 |
| 187 | 252 | 2.307692 | 66 | 0 | 736 |
| 187 | 329 | 2.040816 | 66 | 0 | 736 |
| 187 | 413 | 5 | 66 | 0 | 736 |
| 187 | 476 | 5.16129 | 66 | 0 | 736 |
| 187 | 560 | 1.416765 | 66 | 0 | 736 |
| 188 | 0 | 4.485981 | 62 | 1 | 564 |
| 188 | 28 | 6.27451 | 62 | 1 | 564 |
| 188 | 63 | 7.207207 | 62 | 1 | 564 |
| 188 | 154 | 7.407407 | 62 | 1 | 564 |
| 188 | 245 | 4.083333 | 62 | 1 | 564 |
| 188 | 341 | 18.7291 | 62 | 1 | 564 |
| 188 | 423 | 5.142857 | 62 | 1 | 564 |
| 188 | 497 | 9.350649 | 62 | 1 | 564 |
| 188 | 563 | 8.602151 | 62 | 1 | 564 |
| 189 | 0 | 4.210526 | 79 | 0 | 943 |
| 189 | 28 | 7.142857 | 79 | 0 | 943 |
| 189 | 84 | 5.245902 | 79 | 0 | 943 |
| 189 | 175 | 5.128205 | 79 | 0 | 943 |
| 189 | 259 | 5.217391 | 79 | 0 | 943 |
| 189 | 350 | 4.444444 | 79 | 0 | 943 |
| 189 | 406 | 5.882353 | 79 | 0 | 943 |
| 189 | 504 | 6.722689 | 79 | 0 | 943 |
| 189 | 560 | 5.357143 | 79 | 0 | 943 |
| 190 | 0 | 1.994302 | 87 | 1 | 181 |
| 190 | 27 | 1.851852 | 87 | 1 | 181 |
| 190 | 55 | 5 | 87 | 1 | 181 |
| 190 | 154 | 33.26733 | 87 | 1 | 181 |
| 191 | 0 | 2.521008 | 79 | 0 | 284 |
| 191 | 28 | 5.217391 | 79 | 0 | 284 |
| 191 | 84 | 5.172414 | 79 | 0 | 284 |
| 191 | 168 | 4.615385 | 79 | 0 | 284 |

| | | | | | |
|---|---|---|---|---|---|
| 191 | 252 | 5.555556 | 79 | 0 | 284 |
| 192 | 0 | 8.659794 | 84 | 0 | 889 |
| 193 | 0 | 9.320388 | 67 | 1 | 499 |
| 193 | 35 | 7.843137 | 67 | 1 | 499 |
| 193 | 77 | 7.33945 | 67 | 1 | 499 |
| 193 | 161 | 5.91133 | 67 | 1 | 499 |
| 193 | 245 | 11.00917 | 67 | 1 | 499 |
| 194 | 0 | 2.222222 | 75 | 1 | 385 |
| 194 | 28 | 2.285714 | 75 | 1 | 385 |
| 194 | 94 | 6.089163 | 75 | 1 | 385 |
| 194 | 189 | 12.76596 | 75 | 1 | 385 |
| 194 | 238 | 9.795918 | 75 | 1 | 385 |
| 194 | 339 | 5.925926 | 75 | 1 | 385 |
| 194 | 377 | 6.787879 | 75 | 1 | 385 |
| 195 | 0 | 2.285714 | 37 | 0 | 954 |
| 195 | 37 | 3.368421 | 37 | 0 | 954 |
| 195 | 102 | 6.593407 | 37 | 0 | 954 |
| 195 | 158 | 7.5 | 37 | 0 | 954 |
| 195 | 242 | 9.230769 | 37 | 0 | 954 |
| 195 | 319 | 8.484848 | 37 | 0 | 954 |
| 195 | 412 | 7.973422 | 37 | 0 | 954 |
| 196 | 0 | 2.12766 | 64 | 0 | 1097 |
| 196 | 28 | 34.28571 | 64 | 0 | 1097 |
| 196 | 91 | 20.40486 | 64 | 0 | 1097 |
| 196 | 161 | 15 | 64 | 0 | 1097 |
| 196 | 252 | 8.944099 | 64 | 0 | 1097 |
| 196 | 308 | 6.4 | 64 | 0 | 1097 |
| 196 | 413 | 11.16279 | 64 | 0 | 1097 |
| 196 | 483 | 9.090909 | 64 | 0 | 1097 |
| 196 | 553 | 13.74046 | 64 | 0 | 1097 |
| 197 | 0 | 6.818182 | 56 | 0 | 586 |
| 197 | 28 | 9.896907 | 56 | 0 | 586 |
| 197 | 84 | 9.230769 | 56 | 0 | 586 |
| 198 | 0 | 5.769231 | 76 | 0 | 750 |
| 198 | 28 | 4.324324 | 76 | 0 | 750 |
| 198 | 93 | 5.263158 | 76 | 0 | 750 |
| 198 | 156 | 3.966942 | 76 | 0 | 750 |
| 198 | 261 | 3.870968 | 76 | 0 | 750 |
| 198 | 331 | 4.83871 | 76 | 0 | 750 |

| | | | | | |
|---|---|---|---|---|---|
| 198 | 415 | 2.5 | 76 | 0 | 750 |
| 198 | 484 | 1.958384 | 76 | 0 | 750 |
| 199 | 0 | 0.555556 | 75 | 0 | 1031 |
| 199 | 98 | 0.833333 | 75 | 0 | 1031 |
| 199 | 189 | 0.292398 | 75 | 0 | 1031 |
| 199 | 280 | 0.333333 | 75 | 0 | 1031 |
| 199 | 343 | 0.3367 | 75 | 0 | 1031 |
| 199 | 420 | 0.555556 | 75 | 0 | 1031 |
| 200 | 0 | 0.619195 | 81 | 0 | 148 |
| 200 | 49 | 2.580645 | 81 | 0 | 148 |
| 200 | 147 | 2.553191 | 81 | 0 | 148 |
| 201 | 0 | 11.53846 | 72 | 0 | 86 |
| 201 | 28 | 10.61947 | 72 | 0 | 86 |
| 202 | 0 | 2.419355 | 73 | 0 | 224 |
| 202 | 28 | 2.884615 | 73 | 0 | 224 |
| 202 | 84 | 4.948454 | 73 | 0 | 224 |
| 202 | 147 | 11.88119 | 73 | 0 | 224 |
| 202 | 217 | 23.07692 | 73 | 0 | 224 |
| 203 | 0 | 0.948617 | 73 | 0 | 764 |
| 203 | 28 | 0.974026 | 73 | 0 | 764 |
| 203 | 63 | 6 | 73 | 0 | 764 |
| 203 | 175 | 2.542373 | 73 | 0 | 764 |
| 203 | 259 | 2.803738 | 73 | 0 | 764 |
| 203 | 343 | 5.454545 | 73 | 0 | 764 |
| 203 | 427 | 12.37113 | 73 | 0 | 764 |
| 203 | 518 | 9.795918 | 73 | 0 | 764 |
| 204 | 0 | 4.958678 | 72 | 1 | 739 |
| 204 | 28 | 3.125 | 72 | 1 | 739 |
| 204 | 84 | 3.333333 | 72 | 1 | 739 |
| 204 | 166 | 3.571429 | 72 | 1 | 739 |
| 204 | 266 | 2.749141 | 72 | 1 | 739 |
| 204 | 320 | 5.384615 | 72 | 1 | 739 |
| 204 | 418 | 7.843137 | 72 | 1 | 739 |
| 204 | 502 | 11.53846 | 72 | 1 | 739 |
| 204 | 586 | 10.61947 | 72 | 1 | 739 |
| 205 | 0 | 2.068966 | 78 | 0 | 704 |
| 205 | 35 | 1.271186 | 78 | 0 | 704 |
| 205 | 91 | 1.15942 | 78 | 0 | 704 |
| 205 | 154 | 0.714286 | 78 | 0 | 704 |

| | | | | | |
|---|---|---|---|---|---|
| 205 | 259 | 1.212121 | 78 | 0 | 704 |
| 205 | 322 | 0.415225 | 78 | 0 | 704 |
| 205 | 408 | 0.395294 | 78 | 0 | 704 |
| 205 | 492 | 1.234568 | 78 | 0 | 704 |
| 205 | 555 | 1.883239 | 78 | 0 | 704 |
| 206 | 0 | 3.636364 | 88 | 0 | 956 |
| 206 | 28 | 2.909091 | 88 | 0 | 956 |
| 206 | 63 | 3.636364 | 88 | 0 | 956 |
| 206 | 175 | 3.773585 | 88 | 0 | 956 |
| 206 | 259 | 3.508772 | 88 | 0 | 956 |
| 206 | 343 | 2.654867 | 88 | 0 | 956 |
| 206 | 427 | 2.678571 | 88 | 0 | 956 |
| 206 | 539 | 2.727273 | 88 | 0 | 956 |
| 206 | 588 | 2.631579 | 88 | 0 | 956 |
| 207 | 0 | 5.172414 | 63 | 1 | 225 |
| 207 | 28 | 4.363636 | 63 | 1 | 225 |
| 207 | 91 | 8.490566 | 63 | 1 | 225 |
| 207 | 175 | 5.471125 | 63 | 1 | 225 |
| 208 | 0 | 9.896907 | 69 | 0 | 907 |
| 208 | 35 | 12.2449 | 69 | 0 | 907 |
| 208 | 91 | 11.76471 | 69 | 0 | 907 |
| 208 | 175 | 11.32075 | 69 | 0 | 907 |
| 208 | 259 | 16.51376 | 69 | 0 | 907 |
| 208 | 350 | 14.25743 | 69 | 0 | 907 |
| 208 | 427 | 10.21277 | 69 | 0 | 907 |
| 208 | 490 | 10.90909 | 69 | 0 | 907 |
| 209 | 0 | 12.44444 | 70 | 1 | 122 |
| 209 | 29 | 6.315789 | 70 | 1 | 122 |
| 209 | 85 | 12.87356 | 70 | 1 | 122 |
| 210 | 0 | 9.756098 | 49 | 0 | 610 |
| 210 | 28 | 1.584158 | 49 | 0 | 610 |
| 210 | 63 | 1.584158 | 49 | 0 | 610 |
| 210 | 147 | 10.61947 | 49 | 0 | 610 |
| 210 | 231 | 11.11111 | 49 | 0 | 610 |
| 210 | 287 | 10.52632 | 49 | 0 | 610 |
| 210 | 399 | 3.230769 | 49 | 0 | 610 |
| 210 | 511 | 10.16949 | 49 | 0 | 610 |
| 210 | 553 | 8.695652 | 49 | 0 | 610 |
| 211 | 0 | 17.96151 | 78 | 0 | 981 |

| | | | | | |
|---|---|---|---|---|---|
| 211 | 28 | 13.11134 | 78 | 0 | 981 |
| 211 | 84 | 19.5122 | 78 | 0 | 981 |
| 211 | 175 | 5.714286 | 78 | 0 | 981 |
| 211 | 245 | 15.65217 | 78 | 0 | 981 |
| 211 | 340 | 12.41074 | 78 | 0 | 981 |
| 211 | 406 | 11.80328 | 78 | 0 | 981 |
| 211 | 497 | 11.25 | 78 | 0 | 981 |
| 211 | 560 | 13.74046 | 78 | 0 | 981 |
| 212 | 0 | 5.172414 | 68 | 0 | 743 |
| 212 | 28 | 2.380952 | 68 | 0 | 743 |
| 212 | 119 | 2.702703 | 68 | 0 | 743 |
| 212 | 175 | 2.777778 | 68 | 0 | 743 |
| 212 | 252 | 3.571429 | 68 | 0 | 743 |
| 212 | 343 | 3.703704 | 68 | 0 | 743 |
| 212 | 427 | 3.603604 | 68 | 0 | 743 |
| 212 | 518 | 3.773585 | 68 | 0 | 743 |
| 212 | 602 | 3.137255 | 68 | 0 | 743 |
| 213 | 0 | 1.983471 | 51 | 0 | 236 |
| 213 | 35 | 2.222222 | 51 | 0 | 236 |
| 213 | 105 | 2.181818 | 51 | 0 | 236 |
| 213 | 140 | 4.363636 | 51 | 0 | 236 |
| 214 | 0 | 2.631579 | 85 | 0 | 575 |
| 214 | 28 | 5.504587 | 85 | 0 | 575 |
| 214 | 91 | 5.555556 | 85 | 0 | 575 |
| 214 | 161 | 5.084746 | 85 | 0 | 575 |
| 214 | 252 | 6.349206 | 85 | 0 | 575 |
| 214 | 350 | 6.233766 | 85 | 0 | 575 |
| 214 | 427 | 7.407407 | 85 | 0 | 575 |
| 214 | 469 | 3.018868 | 85 | 0 | 575 |
| 214 | 574 | 3.047619 | 85 | 0 | 575 |
| 215 | 0 | 0.729404 | 68 | 1 | 39 |
| 215 | 21 | 0.624129 | 68 | 1 | 39 |
| 216 | 0 | 5.357143 | 58 | 1 | 429 |
| 216 | 28 | 4.363636 | 58 | 1 | 429 |
| 216 | 91 | 5.405405 | 58 | 1 | 429 |
| 216 | 154 | 5.454545 | 58 | 1 | 429 |
| 216 | 245 | 10.34483 | 58 | 1 | 429 |
| 216 | 364 | 5.405405 | 58 | 1 | 429 |
| 216 | 392 | 7.30276 | 58 | 1 | 429 |

| | | | | | |
|---|---|---|---|---|---|
| 217 | 0 | 3.298969 | 57 | 1 | 88 |
| 217 | 28 | 8.695652 | 57 | 1 | 88 |
| 217 | 84 | 16.24758 | 57 | 1 | 88 |
| 218 | 0 | 0.778643 | 72 | 0 | 1026 |
| 218 | 37 | 0.740349 | 72 | 0 | 1026 |
| 218 | 93 | 1.401869 | 72 | 0 | 1026 |
| 218 | 198 | 3.204272 | 72 | 0 | 1026 |
| 218 | 294 | 2.727273 | 72 | 0 | 1026 |
| 218 | 350 | 2.702703 | 72 | 0 | 1026 |
| 218 | 406 | 2.608696 | 72 | 0 | 1026 |
| 218 | 518 | 2.654867 | 72 | 0 | 1026 |
| 218 | 574 | 2.564103 | 72 | 0 | 1026 |
| 219 | 0 | 3.361345 | 74 | 1 | 52 |
| 220 | 0 | 1.941748 | 67 | 1 | 457 |
| 220 | 84 | 6 | 67 | 1 | 457 |
| 220 | 177 | 1.843318 | 67 | 1 | 457 |
| 220 | 261 | 26.08696 | 67 | 1 | 457 |
| 220 | 338 | 11.65049 | 67 | 1 | 457 |
| 220 | 422 | 11.76471 | 67 | 1 | 457 |
| 221 | 0 | 1.371429 | 65 | 0 | 1058 |
| 221 | 28 | 2.752294 | 65 | 0 | 1058 |
| 221 | 56 | 2.608696 | 65 | 0 | 1058 |
| 221 | 168 | 2.201835 | 65 | 0 | 1058 |
| 221 | 238 | 2.307692 | 65 | 0 | 1058 |
| 221 | 343 | 3 | 65 | 0 | 1058 |
| 221 | 441 | 2.44898 | 65 | 0 | 1058 |
| 221 | 511 | 2.891566 | 65 | 0 | 1058 |
| 221 | 616 | 3.333333 | 65 | 0 | 1058 |
| 222 | 0 | 2.142857 | 64 | 0 | 792 |
| 222 | 35 | 2.264151 | 64 | 0 | 792 |
| 222 | 105 | 2.105263 | 64 | 0 | 792 |
| 222 | 175 | 2.105263 | 64 | 0 | 792 |
| 222 | 252 | 1.652893 | 64 | 0 | 792 |
| 222 | 343 | 1.440576 | 64 | 0 | 792 |
| 222 | 392 | 1.869159 | 64 | 0 | 792 |
| 222 | 483 | 1.282051 | 64 | 0 | 792 |
| 222 | 595 | 1.293103 | 64 | 0 | 792 |
| 223 | 0 | 1.612903 | 67 | 0 | 1163 |
| 223 | 91 | 0.877193 | 67 | 0 | 1163 |

| | | | | | |
|---|---|---|---|---|---|
| 223 | 147 | 1.709402 | 67 | 0 | 1163 |
| 223 | 273 | 1.742919 | 67 | 0 | 1163 |
| 223 | 336 | 3.508772 | 67 | 0 | 1163 |
| 223 | 406 | 2.339181 | 67 | 0 | 1163 |
| 223 | 511 | 2.970297 | 67 | 0 | 1163 |
| 223 | 567 | 2.941176 | 67 | 0 | 1163 |
| 224 | 0 | 12.74336 | 75 | 1 | 72 |
| 224 | 28 | 69.90291 | 75 | 1 | 72 |
| 225 | 0 | 4.971885 | 62 | 0 | 1338 |
| 225 | 31 | 3.208556 | 62 | 0 | 1338 |
| 225 | 105 | 5.233645 | 62 | 0 | 1338 |
| 225 | 166 | 5.137615 | 62 | 0 | 1338 |
| 225 | 257 | 4.127764 | 62 | 0 | 1338 |
| 225 | 355 | 8.284024 | 62 | 0 | 1338 |
| 225 | 441 | 11.42857 | 62 | 0 | 1338 |
| 225 | 504 | 15.74508 | 62 | 0 | 1338 |
| 225 | 595 | 16.58986 | 62 | 0 | 1338 |
| 226 | 0 | 5.607477 | 70 | 1 | 505 |
| 226 | 28 | 5.504587 | 70 | 1 | 505 |
| 226 | 84 | 9.574468 | 70 | 1 | 505 |
| 226 | 161 | 16.66667 | 70 | 1 | 505 |
| 226 | 321 | 9.333333 | 70 | 1 | 505 |
| 226 | 441 | 21.17647 | 70 | 1 | 505 |
| 227 | 0 | 8.259587 | 50 | 0 | 936 |
| 227 | 62 | 9.343715 | 50 | 0 | 936 |
| 227 | 153 | 1.875 | 50 | 0 | 936 |
| 227 | 245 | 2.308961 | 50 | 0 | 936 |
| 227 | 321 | 2.678571 | 50 | 0 | 936 |
| 227 | 419 | 2.654867 | 50 | 0 | 936 |
| 227 | 504 | 3.846154 | 50 | 0 | 936 |
| 227 | 588 | 3.738318 | 50 | 0 | 936 |
| 228 | 0 | 2.912621 | 75 | 1 | 202 |
| 228 | 28 | 2.359882 | 75 | 1 | 202 |
| 228 | 91 | 5.555556 | 75 | 1 | 202 |
| 229 | 0 | 2.474227 | 59 | 1 | 420 |
| 229 | 28 | 5.660377 | 59 | 1 | 420 |
| 229 | 84 | 4.485981 | 59 | 1 | 420 |
| 229 | 175 | 5.217391 | 59 | 1 | 420 |
| 229 | 238 | 5.607477 | 59 | 1 | 420 |

| | | | | | |
|---|---|---|---|---|---|
| 229 | 322 | 10 | 59 | 1 | 420 |
| 230 | 0 | 3.174603 | 75 | 0 | 660 |
| 230 | 98 | 3.508772 | 75 | 0 | 660 |
| 230 | 149 | 3.361345 | 75 | 0 | 660 |
| 230 | 232 | 2.571166 | 75 | 0 | 660 |
| 230 | 330 | 4.991087 | 75 | 0 | 660 |
| 230 | 408 | 3.703704 | 75 | 0 | 660 |
| 230 | 520 | 2.564103 | 75 | 0 | 660 |
| 230 | 597 | 4.938272 | 75 | 0 | 660 |
| 231 | 0 | 1.975309 | 51 | 0 | 661 |
| 231 | 21 | 1.176471 | 51 | 0 | 661 |
| 231 | 77 | 1.503759 | 51 | 0 | 661 |
| 231 | 161 | 2.272727 | 51 | 0 | 661 |
| 231 | 252 | 2.479339 | 51 | 0 | 661 |
| 231 | 357 | 1.652893 | 51 | 0 | 661 |
| 231 | 399 | 1.106428 | 51 | 0 | 661 |
| 231 | 491 | 1.209677 | 51 | 0 | 661 |
| 231 | 588 | 3.155522 | 51 | 0 | 661 |
| 232 | 0 | 5.940594 | 66 | 1 | 715 |
| 232 | 28 | 5.769231 | 66 | 1 | 715 |
| 232 | 56 | 0.779221 | 66 | 1 | 715 |
| 232 | 280 | 0.996678 | 66 | 1 | 715 |
| 232 | 336 | 9.302326 | 66 | 1 | 715 |
| 232 | 434 | 11.65049 | 66 | 1 | 715 |
| 232 | 462 | 11.76471 | 66 | 1 | 715 |
| 233 | 0 | 2.702703 | 48 | 1 | 511 |
| 233 | 28 | 5.769231 | 48 | 1 | 511 |
| 233 | 84 | 9.708738 | 48 | 1 | 511 |
| 233 | 182 | 10.32258 | 48 | 1 | 511 |
| 233 | 266 | 15.05376 | 48 | 1 | 511 |
| 233 | 345 | 14.84536 | 48 | 1 | 511 |
| 233 | 408 | 20 | 48 | 1 | 511 |
| 233 | 506 | 14.4 | 48 | 1 | 511 |
| 234 | 0 | 9.056604 | 79 | 1 | 232 |
| 234 | 35 | 16.32653 | 79 | 1 | 232 |
| 234 | 84 | 11.76471 | 79 | 1 | 232 |
| 234 | 175 | 28.40237 | 79 | 1 | 232 |
| 235 | 0 | 2.542373 | 71 | 0 | 941 |
| 235 | 28 | 2.803738 | 71 | 0 | 941 |

| | | | | | |
|---|---|---|---|---|---|
| 235 | 84 | 1.853486 | 71 | 0 | 941 |
| 235 | 168 | 3.856749 | 71 | 0 | 941 |
| 235 | 252 | 2.376238 | 71 | 0 | 941 |
| 235 | 322 | 6.25 | 71 | 0 | 941 |
| 235 | 434 | 6.060606 | 71 | 0 | 941 |
| 235 | 497 | 6.990291 | 71 | 0 | 941 |
| 235 | 604 | 9.764603 | 71 | 0 | 941 |
| 236 | 0 | 10.66667 | 75 | 1 | 309 |
| 236 | 21 | 8 | 75 | 1 | 309 |
| 236 | 77 | 4.615385 | 75 | 1 | 309 |
| 236 | 168 | 3.465347 | 75 | 1 | 309 |
| 236 | 269 | 3.394625 | 75 | 1 | 309 |
| 237 | 0 | 2.542373 | 76 | 0 | 687 |
| 237 | 84 | 2.419355 | 76 | 0 | 687 |
| 237 | 147 | 2.586207 | 76 | 0 | 687 |
| 237 | 273 | 2.222222 | 76 | 0 | 687 |
| 237 | 329 | 1.922197 | 76 | 0 | 687 |
| 237 | 392 | 2.542373 | 76 | 0 | 687 |
| 237 | 518 | 2.123894 | 76 | 0 | 687 |
| 237 | 574 | 2.727273 | 76 | 0 | 687 |
| 238 | 72 | 3.125 | 79 | 0 | 792 |
| 238 | 154 | 5.714286 | 79 | 0 | 792 |
| 238 | 252 | 5.309735 | 79 | 0 | 792 |
| 238 | 322 | 3.361345 | 79 | 0 | 792 |
| 238 | 448 | 3.418803 | 79 | 0 | 792 |
| 238 | 490 | 1.538462 | 79 | 0 | 792 |
| 238 | 581 | 3.2 | 79 | 0 | 792 |
| 239 | 0 | 0.454545 | 69 | 0 | 726 |
| 239 | 77 | 0.832178 | 69 | 0 | 726 |
| 239 | 175 | 0.840336 | 69 | 0 | 726 |
| 239 | 259 | 0.793651 | 69 | 0 | 726 |
| 239 | 322 | 0.917431 | 69 | 0 | 726 |
| 239 | 406 | 1.000834 | 69 | 0 | 726 |
| 239 | 462 | 1.080108 | 69 | 0 | 726 |
| 240 | 0 | 2.521008 | 49 | 0 | 1311 |
| 240 | 14 | 3.252033 | 49 | 0 | 1311 |
| 240 | 77 | 4.571429 | 49 | 0 | 1311 |
| 240 | 168 | 3.846154 | 49 | 0 | 1311 |
| 240 | 238 | 5.607477 | 49 | 0 | 1311 |

| | | | | | |
|---|---|---|---|---|---|
| 240 | 329 | 5.660377 | 49 | 0 | 1311 |
| 240 | 420 | 4.705882 | 49 | 0 | 1311 |
| 240 | 483 | 12.63158 | 49 | 0 | 1311 |
| 240 | 574 | 9.411765 | 49 | 0 | 1311 |
| 241 | 0 | 5.128205 | 78 | 0 | 1191 |
| 241 | 28 | 4.671858 | 78 | 0 | 1191 |
| 241 | 85 | 5.414115 | 78 | 0 | 1191 |
| 241 | 197 | 2.542373 | 78 | 0 | 1191 |
| 241 | 253 | 4.90941 | 78 | 0 | 1191 |
| 241 | 365 | 5.185185 | 78 | 0 | 1191 |
| 241 | 421 | 6.040992 | 78 | 0 | 1191 |
| 241 | 504 | 5.405405 | 78 | 0 | 1191 |
| 242 | 0 | 5.042017 | 57 | 0 | 840 |
| 243 | 0 | 8 | 69 | 1 | 310 |
| 243 | 35 | 15.31915 | 69 | 1 | 310 |
| 243 | 133 | 21.96078 | 69 | 1 | 310 |
| 243 | 217 | 17.47573 | 69 | 1 | 310 |
| 244 | 0 | 7.207207 | 88 | 0 | 140 |
| 244 | 69 | 14.11765 | 88 | 0 | 140 |
| 245 | 0 | 6.160616 | 89 | 0 | 651 |
| 245 | 27 | 11.42857 | 89 | 0 | 651 |
| 245 | 83 | 14.6789 | 89 | 0 | 651 |
| 245 | 181 | 7.079646 | 89 | 0 | 651 |
| 245 | 251 | 9.230769 | 89 | 0 | 651 |
| 245 | 335 | 10.90909 | 89 | 0 | 651 |
| 245 | 433 | 17.47573 | 89 | 0 | 651 |
| 245 | 503 | 16.94118 | 89 | 0 | 651 |
| 245 | 601 | 22.01835 | 89 | 0 | 651 |
| 246 | 0 | 5.825243 | 73 | 1 | 34 |
| 246 | 28 | 6 | 73 | 1 | 34 |
| 247 | 0 | 2.752294 | 85 | 0 | 78 |
| 248 | 0 | 2.352941 | 82 | 0 | 645 |
| 248 | 28 | 2.830189 | 82 | 0 | 645 |
| 248 | 84 | 4.040404 | 82 | 0 | 645 |
| 248 | 168 | 6.25 | 82 | 0 | 645 |
| 248 | 231 | 4.528302 | 82 | 0 | 645 |
| 248 | 322 | 5.769231 | 82 | 0 | 645 |
| 248 | 416 | 10.43478 | 82 | 0 | 645 |
| 248 | 490 | 4.173913 | 82 | 0 | 645 |

| | | | | | |
|---|---|---|---|---|---|
| 249 | 0 | 1.495327 | 62 | 1 | 505 |
| 249 | 21 | 3.571429 | 62 | 1 | 505 |
| 249 | 91 | 1.709402 | 62 | 1 | 505 |
| 249 | 133 | 1.73913 | 62 | 1 | 505 |
| 249 | 259 | 1.37931 | 62 | 1 | 505 |
| 249 | 336 | 3.773585 | 62 | 1 | 505 |
| 249 | 427 | 3.636364 | 62 | 1 | 505 |
| 249 | 503 | 2.721088 | 62 | 1 | 505 |
| 250 | 0 | 2.803738 | 74 | 0 | 627 |
| 250 | 28 | 2.181818 | 74 | 0 | 627 |
| 250 | 98 | 2.330097 | 74 | 0 | 627 |
| 250 | 154 | 2.162162 | 74 | 0 | 627 |
| 250 | 255 | 2.4 | 74 | 0 | 627 |
| 250 | 353 | 3.092784 | 74 | 0 | 627 |
| 250 | 381 | 3.125 | 74 | 0 | 627 |
| 250 | 528 | 6.382979 | 74 | 0 | 627 |
| 250 | 563 | 4.948454 | 74 | 0 | 627 |
| 251 | 0 | 1.46789 | 77 | 0 | 666 |
| 251 | 35 | 1.257862 | 77 | 0 | 666 |
| 251 | 77 | 1.294498 | 77 | 0 | 666 |
| 251 | 167 | 3.921569 | 77 | 0 | 666 |
| 251 | 231 | 3.60941 | 77 | 0 | 666 |
| 251 | 343 | 2.469136 | 77 | 0 | 666 |
| 251 | 412 | 1.694915 | 77 | 0 | 666 |
| 251 | 511 | 1.923077 | 77 | 0 | 666 |
| 251 | 588 | 6.122449 | 77 | 0 | 666 |
| 252 | 0 | 5.607477 | 80 | 0 | 1114 |
| 252 | 21 | 7.964602 | 80 | 0 | 1114 |
| 252 | 77 | 5.357143 | 80 | 0 | 1114 |
| 252 | 161 | 11.32075 | 80 | 0 | 1114 |
| 252 | 245 | 10.81081 | 80 | 0 | 1114 |
| 252 | 329 | 10.81081 | 80 | 0 | 1114 |
| 252 | 413 | 10.90909 | 80 | 0 | 1114 |
| 252 | 497 | 10.43478 | 80 | 0 | 1114 |
| 252 | 567 | 7.33945 | 80 | 0 | 1114 |
| 253 | 0 | 1.503759 | 86 | 0 | 855 |
| 253 | 28 | 1.066667 | 86 | 0 | 855 |
| 253 | 91 | 1.359388 | 86 | 0 | 855 |
| 253 | 168 | 1.212121 | 86 | 0 | 855 |

| | | | | | |
|---|---|---|---|---|---|
| 253 | 252 | 2.020202 | 86 | 0 | 855 |
| 253 | 329 | 1.675309 | 86 | 0 | 855 |
| 253 | 420 | 3.783358 | 86 | 0 | 855 |
| 253 | 504 | 3.669725 | 86 | 0 | 855 |
| 253 | 588 | 5.504587 | 86 | 0 | 855 |
| 254 | 0 | 1.724138 | 59 | 1 | 828 |
| 254 | 29 | 2.777778 | 59 | 1 | 828 |
| 254 | 85 | 1.271186 | 59 | 1 | 828 |
| 254 | 169 | 0.782269 | 59 | 1 | 828 |
| 254 | 258 | 2.857143 | 59 | 1 | 828 |
| 254 | 342 | 0.819672 | 59 | 1 | 828 |
| 254 | 421 | 2.803738 | 59 | 1 | 828 |
| 254 | 505 | 2.702703 | 59 | 1 | 828 |
| 254 | 608 | 1.810345 | 59 | 1 | 828 |
| 255 | 0 | 0.368155 | 69 | 0 | 877 |
| 255 | 92 | 0.397219 | 69 | 0 | 877 |
| 255 | 147 | 0.38053 | 69 | 0 | 877 |
| 255 | 238 | 0.387034 | 69 | 0 | 877 |
| 255 | 336 | 0.411664 | 69 | 0 | 877 |
| 255 | 427 | 0.371172 | 69 | 0 | 877 |
| 255 | 525 | 0.397219 | 69 | 0 | 877 |
| 255 | 714 | 4.848485 | 69 | 0 | 877 |
| 256 | 0 | 0.639659 | 71 | 0 | 1037 |
| 256 | 98 | 0.877193 | 71 | 0 | 1037 |
| 256 | 189 | 1.157184 | 71 | 0 | 1037 |
| 256 | 280 | 1.260504 | 71 | 0 | 1037 |
| 256 | 336 | 2.272727 | 71 | 0 | 1037 |
| 256 | 392 | 0.983607 | 71 | 0 | 1037 |
| 256 | 532 | 0.96 | 71 | 0 | 1037 |
| 257 | 0 | 11.65049 | 45 | 0 | 725 |
| 257 | 28 | 11.32075 | 45 | 0 | 725 |
| 257 | 84 | 11.65049 | 45 | 0 | 725 |
| 257 | 171 | 10 | 45 | 0 | 725 |
| 257 | 266 | 9.320388 | 45 | 0 | 725 |
| 257 | 329 | 12 | 45 | 0 | 725 |
| 257 | 420 | 12.37113 | 45 | 0 | 725 |
| 257 | 511 | 11.76471 | 45 | 0 | 725 |
| 257 | 602 | 10.10526 | 45 | 0 | 725 |
| 258 | 0 | 2.542373 | 79 | 0 | 862 |

| | | | | | |
|---|---|---|---|---|---|
| 258 | 28 | 7.407407 | 79 | 0 | 862 |
| 258 | 84 | 5.217391 | 79 | 0 | 862 |
| 258 | 168 | 5.263158 | 79 | 0 | 862 |
| 258 | 252 | 5.084746 | 79 | 0 | 862 |
| 258 | 336 | 5.504587 | 79 | 0 | 862 |
| 258 | 427 | 4.528302 | 79 | 0 | 862 |
| 259 | 0 | 8.888889 | 81 | 0 | 918 |
| 259 | 28 | 8.163265 | 81 | 0 | 918 |
| 259 | 84 | 3.921569 | 81 | 0 | 918 |
| 259 | 168 | 7.920792 | 81 | 0 | 918 |
| 259 | 252 | 3.960396 | 81 | 0 | 918 |
| 259 | 378 | 8.695652 | 81 | 0 | 918 |
| 259 | 420 | 6.757844 | 81 | 0 | 918 |
| 259 | 511 | 6.757844 | 81 | 0 | 918 |
| 259 | 588 | 7.920792 | 81 | 0 | 918 |
| 260 | 0 | 6.451613 | 59 | 0 | 1198 |
| 260 | 28 | 4.848485 | 59 | 0 | 1198 |
| 260 | 91 | 4.848485 | 59 | 0 | 1198 |
| 260 | 161 | 12.76596 | 59 | 0 | 1198 |
| 260 | 245 | 10.90909 | 59 | 0 | 1198 |
| 260 | 329 | 4.40367 | 59 | 0 | 1198 |
| 260 | 420 | 9.056604 | 59 | 0 | 1198 |
| 260 | 490 | 12.37113 | 59 | 0 | 1198 |
| 260 | 574 | 11.65049 | 59 | 0 | 1198 |
| 261 | 0 | 6.060606 | 78 | 0 | 176 |
| 261 | 28 | 16.43836 | 78 | 0 | 176 |
| 261 | 91 | 15.65704 | 78 | 0 | 176 |
| 261 | 146 | 12.92308 | 78 | 0 | 176 |
| 262 | 0 | 11.11111 | 80 | 1 | 401 |
| 262 | 28 | 5.217391 | 80 | 1 | 401 |
| 262 | 84 | 8.333333 | 80 | 1 | 401 |
| 262 | 168 | 9.375 | 80 | 1 | 401 |
| 262 | 270 | 17.24138 | 80 | 1 | 401 |
| 262 | 336 | 16.30435 | 80 | 1 | 401 |
| 263 | 0 | 19.78022 | 81 | 1 | 204 |
| 263 | 28 | 35.64356 | 81 | 1 | 204 |
| 263 | 84 | 39.96828 | 81 | 1 | 204 |
| 263 | 153 | 20.68966 | 81 | 1 | 204 |
| 264 | 0 | 0.987654 | 69 | 0 | 593 |

| | | | | | |
|---|---|---|---|---|---|
| 264 | 63 | 0.820232 | 69 | 0 | 593 |
| 264 | 140 | 0.654664 | 69 | 0 | 593 |
| 264 | 231 | 0.715198 | 69 | 0 | 593 |
| 264 | 312 | 2.303579 | 69 | 0 | 593 |
| 265 | 0 | 5.217391 | 81 | 0 | 515 |
| 265 | 86 | 3.703704 | 81 | 0 | 515 |
| 265 | 147 | 0.714286 | 81 | 0 | 515 |
| 265 | 266 | 0.824742 | 81 | 0 | 515 |
| 265 | 364 | 0.747863 | 81 | 0 | 515 |
| 265 | 436 | 1.560323 | 81 | 0 | 515 |
| 265 | 477 | 1.298701 | 81 | 0 | 515 |
| 266 | 0 | 2.469136 | 76 | 0 | 690 |
| 266 | 28 | 12.63158 | 76 | 0 | 690 |
| 266 | 56 | 2.96244 | 76 | 0 | 690 |
| 266 | 175 | 4.948454 | 76 | 0 | 690 |
| 266 | 238 | 2.580645 | 76 | 0 | 690 |
| 266 | 329 | 2.376238 | 76 | 0 | 690 |
| 266 | 420 | 9.896907 | 76 | 0 | 690 |
| 266 | 490 | 4.705882 | 76 | 0 | 690 |
| 267 | 0 | 2.162162 | 84 | 0 | 696 |
| 267 | 63 | 6.25 | 84 | 0 | 696 |
| 267 | 168 | 4.615385 | 84 | 0 | 696 |
| 267 | 263 | 9.722222 | 84 | 0 | 696 |
| 267 | 353 | 6.375712 | 84 | 0 | 696 |
| 267 | 437 | 2.803738 | 84 | 0 | 696 |
| 267 | 500 | 20.25316 | 84 | 0 | 696 |
| 267 | 581 | 12.35294 | 84 | 0 | 696 |
| 268 | 0 | 2.803738 | 74 | 1 | 263 |
| 268 | 21 | 3.061224 | 74 | 1 | 263 |
| 268 | 140 | 4.528302 | 74 | 1 | 263 |
| 268 | 259 | 22.34043 | 74 | 1 | 263 |
| 269 | 0 | 2.702703 | 60 | 1 | 670 |
| 269 | 28 | 2.702703 | 60 | 1 | 670 |
| 269 | 84 | 5.504587 | 60 | 1 | 670 |
| 269 | 168 | 5.357143 | 60 | 1 | 670 |
| 269 | 252 | 5.405405 | 60 | 1 | 670 |
| 269 | 336 | 5.405405 | 60 | 1 | 670 |
| 269 | 420 | 8.256881 | 60 | 1 | 670 |
| 269 | 502 | 7.433628 | 60 | 1 | 670 |

| | | | | | |
|---|---|---|---|---|---|
| 269 | 588 | 8.411215 | 60 | 1 | 670 |
| 270 | 0 | 5.210918 | 68 | 1 | 361 |
| 270 | 91 | 5.940594 | 68 | 1 | 361 |
| 270 | 175 | 11.88119 | 68 | 1 | 361 |
| 270 | 266 | 13.7931 | 68 | 1 | 361 |
| 270 | 357 | 1.859744 | 68 | 1 | 361 |
| 271 | 0 | 3.883495 | 72 | 0 | 701 |
| 271 | 28 | 3.389831 | 72 | 0 | 701 |
| 271 | 105 | 4.123711 | 72 | 0 | 701 |
| 271 | 266 | 4 | 72 | 0 | 701 |
| 271 | 336 | 3.296703 | 72 | 0 | 701 |
| 271 | 437 | 2.058319 | 72 | 0 | 701 |
| 271 | 504 | 7.079646 | 72 | 0 | 701 |
| 271 | 574 | 3.349282 | 72 | 0 | 701 |
| 272 | 0 | 2.051282 | 70 | 1 | 1094 |
| 272 | 71 | 3.860294 | 70 | 1 | 1094 |
| 272 | 133 | 4.379562 | 70 | 1 | 1094 |
| 272 | 234 | 2.985075 | 70 | 1 | 1094 |
| 272 | 318 | 3.252033 | 70 | 1 | 1094 |
| 272 | 430 | 3.418803 | 70 | 1 | 1094 |
| 272 | 458 | 3.809524 | 70 | 1 | 1094 |
| 272 | 563 | 4 | 70 | 1 | 1094 |
| 273 | 0 | 2.222222 | 86 | 0 | 246 |
| 273 | 189 | 2.5 | 86 | 0 | 246 |
| 273 | 245 | 6 | 86 | 0 | 246 |
| 274 | 0 | 1.401869 | 69 | 0 | 645 |
| 274 | 112 | 2.803738 | 69 | 0 | 645 |
| 274 | 173 | 2.515723 | 69 | 0 | 645 |
| 274 | 238 | 2.039082 | 69 | 0 | 645 |
| 274 | 315 | 1.441441 | 69 | 0 | 645 |
| 274 | 420 | 2.752294 | 69 | 0 | 645 |
| 274 | 488 | 2.521008 | 69 | 0 | 645 |
| 275 | 0 | 3.669725 | 65 | 0 | 967 |
| 275 | 112 | 4.324324 | 65 | 0 | 967 |
| 275 | 203 | 6.349206 | 65 | 0 | 967 |
| 275 | 238 | 3.076923 | 65 | 0 | 967 |
| 275 | 329 | 2.614379 | 65 | 0 | 967 |
| 275 | 455 | 2.259887 | 65 | 0 | 967 |
| 275 | 525 | 2.201835 | 65 | 0 | 967 |

| | | | | | |
|---|---|---|---|---|---|
| 275 | 588 | 4.678363 | 65 | 0 | 967 |
| 276 | 0 | 4.615385 | 68 | 0 | 960 |
| 276 | 29 | 2.4 | 68 | 0 | 960 |
| 276 | 83 | 3.478261 | 68 | 0 | 960 |
| 276 | 167 | 5.882353 | 68 | 0 | 960 |
| 276 | 251 | 4.918033 | 68 | 0 | 960 |
| 276 | 336 | 5.042017 | 68 | 0 | 960 |
| 276 | 425 | 5.217391 | 68 | 0 | 960 |
| 276 | 484 | 11.32075 | 68 | 0 | 960 |
| 276 | 580 | 13.44538 | 68 | 0 | 960 |
| 277 | 0 | 4.8 | 94 | 0 | 1044 |
| 277 | 70 | 10 | 94 | 0 | 1044 |
| 277 | 188 | 4.660194 | 94 | 0 | 1044 |
| 277 | 266 | 6.593407 | 94 | 0 | 1044 |
| 277 | 315 | 5.714286 | 94 | 0 | 1044 |
| 277 | 371 | 9.142857 | 94 | 0 | 1044 |
| 277 | 476 | 7.207207 | 94 | 0 | 1044 |
| 277 | 560 | 11.88119 | 94 | 0 | 1044 |
| 278 | 0 | 8 | 67 | 0 | 1023 |
| 278 | 98 | 9.615385 | 67 | 0 | 1023 |
| 278 | 259 | 15.12605 | 67 | 0 | 1023 |
| 278 | 322 | 14.0625 | 67 | 0 | 1023 |
| 278 | 406 | 4.724409 | 67 | 0 | 1023 |
| 278 | 504 | 2.424242 | 67 | 0 | 1023 |
| 278 | 609 | 2.782609 | 67 | 0 | 1023 |
| 279 | 0 | 5.454545 | 60 | 0 | 541 |
| 279 | 28 | 2.285714 | 60 | 0 | 541 |
| 279 | 98 | 2.44898 | 60 | 0 | 541 |
| 279 | 182 | 8.421053 | 60 | 0 | 541 |
| 279 | 266 | 9.69697 | 60 | 0 | 541 |
| 279 | 343 | 5.797101 | 60 | 0 | 541 |
| 279 | 434 | 18.5567 | 60 | 0 | 541 |
| 279 | 507 | 24.70588 | 60 | 0 | 541 |
| 280 | 0 | 10 | 79 | 0 | 624 |
| 280 | 28 | 2.162162 | 79 | 0 | 624 |
| 280 | 84 | 11.11111 | 79 | 0 | 624 |
| 280 | 147 | 5.172414 | 79 | 0 | 624 |
| 280 | 231 | 10.08403 | 79 | 0 | 624 |
| 280 | 350 | 1.935484 | 79 | 0 | 624 |

| | | | | | |
|---|---|---|---|---|---|
| 280 | 385 | 10.25641 | 79 | 0 | 624 |
| 280 | 511 | 4.958678 | 79 | 0 | 624 |
| 280 | 567 | 9.756098 | 79 | 0 | 624 |
| 281 | 0 | 4.210526 | 77 | 0 | 980 |
| 281 | 63 | 2.086957 | 77 | 0 | 980 |
| 281 | 119 | 1.81308 | 77 | 0 | 980 |
| 281 | 266 | 0.961538 | 77 | 0 | 980 |
| 281 | 294 | 1.617251 | 77 | 0 | 980 |
| 281 | 489 | 2.526316 | 77 | 0 | 980 |
| 281 | 595 | 3.225806 | 77 | 0 | 980 |
| 282 | 0 | 2.571166 | 83 | 0 | 166 |
| 282 | 27 | 2.352941 | 83 | 0 | 166 |
| 282 | 81 | 2.463343 | 83 | 0 | 166 |
| 283 | 0 | 9.312639 | 83 | 0 | 889 |
| 283 | 20 | 9.679278 | 83 | 0 | 889 |
| 283 | 97 | 14.28571 | 83 | 0 | 889 |
| 283 | 160 | 11.90083 | 83 | 0 | 889 |
| 283 | 251 | 9.917355 | 83 | 0 | 889 |
| 283 | 321 | 15.38462 | 83 | 0 | 889 |
| 283 | 405 | 14.4 | 83 | 0 | 889 |
| 283 | 489 | 6.557377 | 83 | 0 | 889 |
| 283 | 552 | 15.25424 | 83 | 0 | 889 |
| 284 | 0 | 2.123894 | 46 | 0 | 697 |
| 284 | 35 | 1.639344 | 46 | 0 | 697 |
| 284 | 77 | 1.724138 | 46 | 0 | 697 |
| 284 | 157 | 1.983471 | 46 | 0 | 697 |
| 284 | 227 | 2.439024 | 46 | 0 | 697 |
| 284 | 325 | 1.016949 | 46 | 0 | 697 |
| 284 | 430 | 2.4 | 46 | 0 | 697 |
| 284 | 486 | 1.889764 | 46 | 0 | 697 |
| 284 | 591 | 2.033898 | 46 | 0 | 697 |
| 285 | 0 | 2.419355 | 77 | 0 | 841 |
| 285 | 28 | 2.459016 | 77 | 0 | 841 |
| 285 | 91 | 0.609756 | 77 | 0 | 841 |
| 285 | 168 | 2.242991 | 77 | 0 | 841 |
| 285 | 245 | 1.304348 | 77 | 0 | 841 |
| 285 | 350 | 1.617251 | 77 | 0 | 841 |
| 286 | 0 | 4.705882 | 67 | 1 | 163 |
| 286 | 35 | 4.528302 | 67 | 1 | 163 |

| | | | | | |
|---|---|---|---|---|---|
| 286 | 91 | 5.607477 | 67 | 1 | 163 |
| 286 | 147 | 6.521739 | 67 | 1 | 163 |
| 287 | 0 | 2.830189 | 75 | 0 | 568 |
| 287 | 91 | 2.884615 | 75 | 0 | 568 |
| 287 | 154 | 2.970297 | 75 | 0 | 568 |
| 287 | 210 | 3 | 75 | 0 | 568 |
| 287 | 336 | 12.5 | 75 | 0 | 568 |
| 287 | 413 | 12 | 75 | 0 | 568 |
| 287 | 476 | 7.777778 | 75 | 0 | 568 |
| 287 | 567 | 16.94118 | 75 | 0 | 568 |
| 288 | 0 | 1.626016 | 52 | 1 | 562 |
| 288 | 28 | 3.773585 | 52 | 1 | 562 |
| 288 | 70 | 5.263158 | 52 | 1 | 562 |
| 288 | 154 | 1.652893 | 52 | 1 | 562 |
| 288 | 210 | 3.478261 | 52 | 1 | 562 |
| 288 | 366 | 9.707242 | 52 | 1 | 562 |
| 288 | 385 | 2.190447 | 52 | 1 | 562 |
| 288 | 539 | 2.349431 | 52 | 1 | 562 |
| 289 | 0 | 2.34375 | 87 | 0 | 876 |
| 289 | 28 | 2.608696 | 87 | 0 | 876 |
| 289 | 91 | 1.694915 | 87 | 0 | 876 |
| 289 | 147 | 1.769912 | 87 | 0 | 876 |
| 289 | 252 | 5.714286 | 87 | 0 | 876 |
| 289 | 343 | 5.217391 | 87 | 0 | 876 |
| 289 | 427 | 2.5 | 87 | 0 | 876 |
| 289 | 483 | 2.123894 | 87 | 0 | 876 |
| 289 | 588 | 3.703704 | 87 | 0 | 876 |
| 290 | 0 | 0.161794 | 51 | 0 | 728 |
| 290 | 35 | 0.160223 | 51 | 0 | 728 |
| 290 | 109 | 0.155688 | 51 | 0 | 728 |
| 290 | 172 | 0.151403 | 51 | 0 | 728 |
| 290 | 256 | 0.154233 | 51 | 0 | 728 |
| 290 | 348 | 0.158682 | 51 | 0 | 728 |
| 290 | 432 | 0.158682 | 51 | 0 | 728 |
| 290 | 537 | 2.970297 | 51 | 0 | 728 |
| 290 | 607 | 3.225806 | 51 | 0 | 728 |
| 291 | 0 | 2.752294 | 79 | 0 | 897 |
| 291 | 42 | 0.631104 | 79 | 0 | 897 |
| 291 | 94 | 0.673995 | 79 | 0 | 897 |

| | | | | | |
|---|---|---|---|---|---|
| 291 | 206 | 5.833333 | 79 | 0 | 897 |
| 291 | 282 | 2.637363 | 79 | 0 | 897 |
| 291 | 352 | 5.474096 | 79 | 0 | 897 |
| 291 | 441 | 6.25 | 79 | 0 | 897 |
| 291 | 497 | 5.405405 | 79 | 0 | 897 |
| 291 | 609 | 5.607477 | 79 | 0 | 897 |
| 292 | 0 | 3.136669 | 69 | 1 | 62 |
| 292 | 26 | 3.883495 | 69 | 1 | 62 |
| 292 | 47 | 4.444444 | 69 | 1 | 62 |
| 293 | 0 | 2.702703 | 38 | 1 | 383 |
| 293 | 28 | 2.912621 | 38 | 1 | 383 |
| 293 | 84 | 2.264151 | 38 | 1 | 383 |
| 293 | 146 | 5.882353 | 38 | 1 | 383 |
| 293 | 258 | 4.528302 | 38 | 1 | 383 |
| 293 | 321 | 11.31313 | 38 | 1 | 383 |
| 294 | 0 | 3 | 57 | 0 | 397 |
| 295 | 0 | 5.217391 | 65 | 0 | 1002 |
| 295 | 105 | 25.97938 | 65 | 0 | 1002 |
| 295 | 172 | 25 | 65 | 0 | 1002 |
| 295 | 259 | 25.26316 | 65 | 0 | 1002 |
| 295 | 326 | 26.37363 | 65 | 0 | 1002 |
| 295 | 427 | 14.6087 | 65 | 0 | 1002 |
| 296 | 0 | 6.504065 | 71 | 1 | 348 |
| 296 | 21 | 4.8 | 71 | 1 | 348 |
| 296 | 161 | 4.948454 | 71 | 1 | 348 |
| 296 | 245 | 5.217391 | 71 | 1 | 348 |
| 296 | 333 | 5.25 | 71 | 1 | 348 |
| 297 | 0 | 1.352657 | 46 | 1 | 191 |
| 297 | 98 | 2.727273 | 46 | 1 | 191 |
| 297 | 161 | 5.607477 | 46 | 1 | 191 |
| 298 | 0 | 4.444444 | 76 | 1 | 840 |
| 298 | 91 | 5.128205 | 76 | 1 | 840 |
| 298 | 154 | 4.8 | 76 | 1 | 840 |
| 298 | 294 | 3.589744 | 76 | 1 | 840 |
| 298 | 357 | 2.916667 | 76 | 1 | 840 |
| 298 | 420 | 5.357143 | 76 | 1 | 840 |
| 298 | 504 | 5.714286 | 76 | 1 | 840 |
| 298 | 559 | 5.506391 | 76 | 1 | 840 |
| 299 | 0 | 6.597938 | 67 | 1 | 549 |

| | | | | | |
|---|---|---|---|---|---|
| 299 | 35 | 6.336634 | 67 | 1 | 549 |
| 299 | 98 | 7.272727 | 67 | 1 | 549 |
| 299 | 168 | 5.405405 | 67 | 1 | 549 |
| 299 | 252 | 8.490566 | 67 | 1 | 549 |
| 299 | 336 | 11.76471 | 67 | 1 | 549 |
| 299 | 420 | 7.668898 | 67 | 1 | 549 |
| 299 | 486 | 11.65049 | 67 | 1 | 549 |
| 299 | 548 | 40 | 67 | 1 | 549 |
| 300 | 0 | 6.605505 | 67 | 0 | 915 |
| 300 | 21 | 5.172414 | 67 | 0 | 915 |
| 300 | 77 | 2.803738 | 67 | 0 | 915 |
| 300 | 165 | 5.825243 | 67 | 0 | 915 |
| 300 | 249 | 4.285714 | 67 | 0 | 915 |
| 300 | 312 | 4.40367 | 67 | 0 | 915 |
| 300 | 410 | 5.309735 | 67 | 0 | 915 |
| 300 | 501 | 5.454545 | 67 | 0 | 915 |
| 300 | 592 | 4.033613 | 67 | 0 | 915 |
| 301 | 0 | 4.242424 | 87 | 0 | 680 |
| 301 | 30 | 3.977273 | 87 | 0 | 680 |
| 301 | 91 | 5.172414 | 87 | 0 | 680 |
| 301 | 147 | 2.362205 | 87 | 0 | 680 |
| 301 | 259 | 5.172414 | 87 | 0 | 680 |
| 301 | 343 | 3.816794 | 87 | 0 | 680 |
| 301 | 427 | 3.389831 | 87 | 0 | 680 |
| 301 | 511 | 3.2 | 87 | 0 | 680 |
| 301 | 567 | 3.539823 | 87 | 0 | 680 |
| 302 | 0 | 1.521739 | 69 | 1 | 265 |
| 302 | 35 | 3.397371 | 69 | 1 | 265 |
| 302 | 126 | 5.714286 | 69 | 1 | 265 |
| 302 | 232 | 6.666667 | 69 | 1 | 265 |
| 303 | 0 | 2.479339 | 80 | 0 | 762 |
| 303 | 28 | 2.809365 | 80 | 0 | 762 |
| 303 | 105 | 5.045045 | 80 | 0 | 762 |
| 303 | 196 | 2.608696 | 80 | 0 | 762 |
| 303 | 285 | 2.681992 | 80 | 0 | 762 |
| 303 | 340 | 2.315964 | 80 | 0 | 762 |
| 303 | 455 | 2.857143 | 80 | 0 | 762 |
| 303 | 511 | 5.454545 | 80 | 0 | 762 |
| 303 | 600 | 4.173913 | 80 | 0 | 762 |

| | | | | | |
|---|---|---|---|---|---|
| 304 | 0 | 5.940594 | 60 | 1 | 206 |
| 304 | 28 | 6 | 60 | 1 | 206 |
| 304 | 56 | 5.825243 | 60 | 1 | 206 |
| 304 | 140 | 4.848485 | 60 | 1 | 206 |
| 304 | 203 | 5.454545 | 60 | 1 | 206 |
| 305 | 0 | 2.912621 | 79 | 1 | 214 |
| 305 | 28 | 6.315789 | 79 | 1 | 214 |
| 305 | 91 | 10.54945 | 79 | 1 | 214 |
| 305 | 182 | 14.81481 | 79 | 1 | 214 |
| 306 | 0 | 3.076923 | 61 | 0 | 890 |
| 306 | 98 | 11.65049 | 61 | 0 | 890 |
| 306 | 154 | 2.103681 | 61 | 0 | 890 |
| 306 | 214 | 3.018868 | 61 | 0 | 890 |
| 306 | 357 | 2.782609 | 61 | 0 | 890 |
| 306 | 420 | 4.637681 | 61 | 0 | 890 |
| 306 | 476 | 2.693603 | 61 | 0 | 890 |
| 306 | 546 | 2.052786 | 61 | 0 | 890 |
| 307 | 0 | 1.333333 | 71 | 0 | 746 |
| 307 | 63 | 1.290323 | 71 | 0 | 746 |
| 307 | 161 | 4.660194 | 71 | 0 | 746 |
| 307 | 254 | 0.968523 | 71 | 0 | 746 |
| 307 | 310 | 1.078167 | 71 | 0 | 746 |
| 307 | 401 | 1.980198 | 71 | 0 | 746 |
| 307 | 485 | 1.693719 | 71 | 0 | 746 |
| 307 | 576 | 1.725377 | 71 | 0 | 746 |
| 308 | 0 | 0.806452 | 78 | 0 | 820 |
| 308 | 28 | 0.892857 | 78 | 0 | 820 |
| 308 | 84 | 2.173913 | 78 | 0 | 820 |
| 308 | 168 | 3.2 | 78 | 0 | 820 |
| 308 | 245 | 1.980198 | 78 | 0 | 820 |
| 308 | 322 | 1.709402 | 78 | 0 | 820 |
| 308 | 434 | 1.886792 | 78 | 0 | 820 |
| 308 | 511 | 0.900901 | 78 | 0 | 820 |
| 309 | 0 | 1.043478 | 73 | 0 | 1052 |
| 309 | 77 | 0.943396 | 73 | 0 | 1052 |
| 309 | 161 | 5.882353 | 73 | 0 | 1052 |
| 309 | 238 | 3.088803 | 73 | 0 | 1052 |
| 309 | 336 | 2.508961 | 73 | 0 | 1052 |
| 309 | 398 | 4.705882 | 73 | 0 | 1052 |

| | | | | | |
|---|---|---|---|---|---|
| 309 | 517 | 4.918033 | 73 | 0 | 1052 |
| 309 | 553 | 2.046784 | 73 | 0 | 1052 |
| 310 | 26 | 3.265306 | 76 | 1 | 100 |
| 310 | 42 | 42.10526 | 76 | 1 | 100 |
| 311 | 0 | 3.061224 | 78 | 1 | 484 |
| 311 | 49 | 3.061224 | 78 | 1 | 484 |
| 311 | 162 | 3.883495 | 78 | 1 | 484 |
| 311 | 254 | 6.722689 | 78 | 1 | 484 |
| 312 | 0 | 2.201835 | 86 | 1 | 406 |
| 312 | 70 | 11.21495 | 86 | 1 | 406 |
| 312 | 161 | 5.042017 | 86 | 1 | 406 |
| 312 | 245 | 13.98058 | 86 | 1 | 406 |
| 312 | 364 | 7 | 86 | 1 | 406 |
| 313 | 0 | 2.752294 | 75 | 0 | 666 |
| 313 | 28 | 2.678571 | 75 | 0 | 666 |
| 313 | 84 | 5.825243 | 75 | 0 | 666 |
| 313 | 168 | 2.542373 | 75 | 0 | 666 |
| 313 | 259 | 6.060606 | 75 | 0 | 666 |
| 313 | 343 | 2.803738 | 75 | 0 | 666 |
| 313 | 427 | 12.63158 | 75 | 0 | 666 |
| 313 | 511 | 11.21495 | 75 | 0 | 666 |
| 313 | 595 | 5.825243 | 75 | 0 | 666 |
| 314 | 0 | 3.389831 | 52 | 0 | 624 |
| 314 | 56 | 5.217391 | 52 | 0 | 624 |
| 314 | 154 | 12.76596 | 52 | 0 | 624 |
| 314 | 217 | 5.673759 | 52 | 0 | 624 |
| 314 | 308 | 11.42857 | 52 | 0 | 624 |
| 314 | 374 | 12.37113 | 52 | 0 | 624 |
| 314 | 525 | 5.940594 | 52 | 0 | 624 |
| 314 | 588 | 9.795918 | 52 | 0 | 624 |
| 315 | 0 | 4.033613 | 70 | 0 | 1086 |
| 315 | 35 | 3.389831 | 70 | 0 | 1086 |
| 315 | 84 | 2.479339 | 70 | 0 | 1086 |
| 315 | 168 | 2.459016 | 70 | 0 | 1086 |
| 315 | 252 | 2.34375 | 70 | 0 | 1086 |
| 315 | 336 | 2.479339 | 70 | 0 | 1086 |
| 315 | 427 | 2.5 | 70 | 0 | 1086 |
| 315 | 511 | 2.654867 | 70 | 0 | 1086 |
| 315 | 595 | 2.439024 | 70 | 0 | 1086 |

| | | | | | |
|---|---|---|---|---|---|
| 316 | 0 | 3.361345 | 71 | 0 | 1086 |
| 316 | 28 | 3.448276 | 71 | 0 | 1086 |
| 316 | 84 | 3.669725 | 71 | 0 | 1086 |
| 316 | 168 | 3.448276 | 71 | 0 | 1086 |
| 316 | 252 | 0.580552 | 71 | 0 | 1086 |
| 316 | 336 | 0.627943 | 71 | 0 | 1086 |
| 316 | 413 | 1.121495 | 71 | 0 | 1086 |
| 317 | 0 | 3.463918 | 51 | 1 | 29 |
| 318 | 0 | 2.830189 | 72 | 1 | 211 |
| 318 | 28 | 2.242991 | 72 | 1 | 211 |
| 318 | 63 | 1.649485 | 72 | 1 | 211 |
| 318 | 168 | 3.368421 | 72 | 1 | 211 |
| 319 | 0 | 2.479339 | 80 | 1 | 511 |
| 319 | 28 | 2.380952 | 80 | 1 | 511 |
| 319 | 84 | 2.654867 | 80 | 1 | 511 |
| 319 | 168 | 2.654867 | 80 | 1 | 511 |
| 319 | 252 | 5.607477 | 80 | 1 | 511 |
| 319 | 329 | 8.187135 | 80 | 1 | 511 |
| 319 | 411 | 7.33945 | 80 | 1 | 511 |
| 319 | 497 | 6.464025 | 80 | 1 | 511 |
| 320 | 0 | 2.702703 | 87 | 0 | 787 |
| 320 | 28 | 1.187111 | 87 | 0 | 787 |
| 320 | 85 | 6.730769 | 87 | 0 | 787 |
| 320 | 161 | 1.651917 | 87 | 0 | 787 |
| 320 | 254 | 2.330097 | 87 | 0 | 787 |
| 320 | 324 | 2.352941 | 87 | 0 | 787 |
| 320 | 422 | 5.106383 | 87 | 0 | 787 |
| 320 | 492 | 10.10526 | 87 | 0 | 787 |
| 320 | 562 | 11.16279 | 87 | 0 | 787 |
| 321 | 0 | 2.616822 | 48 | 0 | 650 |
| 321 | 21 | 2.162162 | 48 | 0 | 650 |
| 321 | 84 | 2.201835 | 48 | 0 | 650 |
| 321 | 119 | 2.285714 | 48 | 0 | 650 |
| 321 | 224 | 2.201835 | 48 | 0 | 650 |
| 321 | 294 | 2.242991 | 48 | 0 | 650 |
| 322 | 0 | 2.439024 | 54 | 0 | 774 |
| 322 | 29 | 1.219512 | 54 | 0 | 774 |
| 322 | 84 | 3.636364 | 54 | 0 | 774 |
| 322 | 169 | 3.361345 | 54 | 0 | 774 |

| | | | | | |
|---|---|---|---|---|---|
| 322 | 252 | 3.508772 | 54 | 0 | 774 |
| 322 | 303 | 2.930403 | 54 | 0 | 774 |
| 322 | 462 | 3.519799 | 54 | 0 | 774 |
| 322 | 546 | 3.2 | 54 | 0 | 774 |
| 322 | 630 | 3.571429 | 54 | 0 | 774 |
| 323 | 0 | 2.264151 | 77 | 0 | 974 |
| 323 | 28 | 2.752294 | 77 | 0 | 974 |
| 323 | 84 | 2.201835 | 77 | 0 | 974 |
| 323 | 182 | 2.068966 | 77 | 0 | 974 |
| 323 | 245 | 2.678571 | 77 | 0 | 974 |
| 323 | 329 | 2.847458 | 77 | 0 | 974 |
| 323 | 413 | 2.752294 | 77 | 0 | 974 |
| 323 | 497 | 2.140673 | 77 | 0 | 974 |
| 323 | 595 | 2.335279 | 77 | 0 | 974 |
| 324 | 0 | 6.185567 | 53 | 0 | 682 |
| 324 | 28 | 6.25 | 53 | 0 | 682 |
| 324 | 91 | 4.705882 | 53 | 0 | 682 |
| 324 | 154 | 6.451613 | 53 | 0 | 682 |
| 324 | 238 | 4.948454 | 53 | 0 | 682 |
| 324 | 343 | 5.052632 | 53 | 0 | 682 |
| 324 | 441 | 5.769231 | 53 | 0 | 682 |
| 324 | 497 | 5.052632 | 53 | 0 | 682 |
| 324 | 567 | 5.217391 | 53 | 0 | 682 |
| 325 | 0 | 2.857143 | 77 | 0 | 638 |
| 325 | 28 | 2.678571 | 77 | 0 | 638 |
| 325 | 84 | 2.830189 | 77 | 0 | 638 |
| 325 | 168 | 3.809524 | 77 | 0 | 638 |
| 325 | 259 | 2.990654 | 77 | 0 | 638 |
| 325 | 322 | 2.909091 | 77 | 0 | 638 |
| 325 | 399 | 3.076923 | 77 | 0 | 638 |
| 325 | 490 | 6.25 | 77 | 0 | 638 |
| 326 | 0 | 12.83422 | 66 | 0 | 5 |
| 327 | 0 | 2.255639 | 70 | 0 | 1324 |
| 327 | 56 | 2.542373 | 70 | 0 | 1324 |
| 327 | 168 | 2.34375 | 70 | 0 | 1324 |
| 327 | 224 | 2.033898 | 70 | 0 | 1324 |
| 327 | 315 | 1.209677 | 70 | 0 | 1324 |
| 327 | 413 | 0.655738 | 70 | 0 | 1324 |
| 327 | 504 | 0.877193 | 70 | 0 | 1324 |

| | | | | | |
|---|---|---|---|---|---|
| 327 | 588 | 0.862069 | 70 | 0 | 1324 |
| 328 | 0 | 1.666667 | 72 | 1 | 519 |
| 328 | 28 | 1.2 | 72 | 1 | 519 |
| 328 | 84 | 2.727273 | 72 | 1 | 519 |
| 328 | 168 | 3.738318 | 72 | 1 | 519 |
| 328 | 245 | 8.333333 | 72 | 1 | 519 |
| 328 | 343 | 3.539823 | 72 | 1 | 519 |
| 329 | 0 | 1.101928 | 83 | 0 | 624 |
| 329 | 28 | 2.678571 | 83 | 0 | 624 |
| 329 | 84 | 2.631579 | 83 | 0 | 624 |
| 329 | 168 | 2.564103 | 83 | 0 | 624 |
| 329 | 252 | 2.631579 | 83 | 0 | 624 |
| 329 | 371 | 1.456311 | 83 | 0 | 624 |
| 329 | 455 | 1.666667 | 83 | 0 | 624 |
| 329 | 567 | 1.73913 | 83 | 0 | 624 |
| 329 | 623 | 2 | 83 | 0 | 624 |
| 330 | 0 | 6.557377 | 69 | 0 | 890 |
| 330 | 21 | 4.067797 | 69 | 0 | 890 |
| 330 | 112 | 5.405405 | 69 | 0 | 890 |
| 330 | 168 | 5.309735 | 69 | 0 | 890 |
| 330 | 252 | 5.882353 | 69 | 0 | 890 |
| 330 | 336 | 5.357143 | 69 | 0 | 890 |
| 330 | 420 | 5.217391 | 69 | 0 | 890 |
| 330 | 511 | 5.217391 | 69 | 0 | 890 |
| 330 | 595 | 6.185567 | 69 | 0 | 890 |
| 331 | 0 | 5.357143 | 79 | 0 | 380 |
| 331 | 28 | 5.084746 | 79 | 0 | 380 |
| 331 | 84 | 4.897959 | 79 | 0 | 380 |
| 331 | 175 | 9.278351 | 79 | 0 | 380 |
| 331 | 252 | 12.12121 | 79 | 0 | 380 |
| 331 | 343 | 9.6 | 79 | 0 | 380 |
| 332 | 0 | 3.846154 | 75 | 1 | 88 |
| 332 | 35 | 4.528302 | 75 | 1 | 88 |
| 333 | 0 | 4.485981 | 56 | 1 | 439 |
| 333 | 35 | 7.54717 | 56 | 1 | 439 |
| 333 | 98 | 5.925926 | 56 | 1 | 439 |
| 333 | 161 | 9.433962 | 56 | 1 | 439 |
| 333 | 245 | 10.81081 | 56 | 1 | 439 |
| 333 | 343 | 21.05263 | 56 | 1 | 439 |

| | | | | | |
|---|---|---|---|---|---|
| 333 | 413 | 14.7541 | 56 | 1 | 439 |
| 334 | 0 | 1.355932 | 66 | 1 | 78 |
| 334 | 35 | 5 | 66 | 1 | 78 |
| 335 | 0 | 2.666667 | 44 | 0 | 554 |
| 335 | 35 | 4.173913 | 44 | 0 | 554 |
| 335 | 70 | 9.320388 | 44 | 0 | 554 |
| 335 | 161 | 10.52632 | 44 | 0 | 554 |
| 335 | 245 | 1.28 | 44 | 0 | 554 |
| 335 | 336 | 5.825243 | 44 | 0 | 554 |
| 335 | 420 | 4.102564 | 44 | 0 | 554 |
| 335 | 490 | 4.210526 | 44 | 0 | 554 |
| 335 | 553 | 5.405405 | 44 | 0 | 554 |
| 336 | 0 | 2.884615 | 76 | 0 | 656 |
| 336 | 28 | 2.857143 | 76 | 0 | 656 |
| 336 | 56 | 1.886792 | 76 | 0 | 656 |
| 336 | 175 | 1.960784 | 76 | 0 | 656 |
| 336 | 217 | 2.666667 | 76 | 0 | 656 |
| 336 | 343 | 4 | 76 | 0 | 656 |
| 336 | 434 | 5.504587 | 76 | 0 | 656 |
| 336 | 490 | 5.825243 | 76 | 0 | 656 |
| 336 | 576 | 5.6 | 76 | 0 | 656 |
| 337 | 0 | 1.212121 | 69 | 1 | 434 |
| 337 | 27 | 3.571429 | 69 | 1 | 434 |
| 337 | 69 | 1.851852 | 69 | 1 | 434 |
| 337 | 153 | 3.738318 | 69 | 1 | 434 |
| 338 | 0 | 7.920792 | 74 | 0 | 462 |
| 338 | 42 | 5.177994 | 74 | 0 | 462 |
| 338 | 105 | 5.177994 | 74 | 0 | 462 |
| 338 | 161 | 5.940594 | 74 | 0 | 462 |
| 338 | 259 | 10.71429 | 74 | 0 | 462 |
| 338 | 343 | 16.21622 | 74 | 0 | 462 |
| 339 | 0 | 4.528302 | 71 | 1 | 268 |
| 339 | 28 | 4.848485 | 71 | 1 | 268 |
| 339 | 63 | 6.521739 | 71 | 1 | 268 |
| 339 | 182 | 9.716599 | 71 | 1 | 268 |
| 339 | 238 | 12.12121 | 71 | 1 | 268 |
| 340 | 0 | 2.941176 | 76 | 0 | 996 |
| 340 | 28 | 0.353982 | 76 | 0 | 996 |
| 340 | 84 | 0.338983 | 76 | 0 | 996 |

| | | | | | |
|---|---|---|---|---|---|
| 340 | 175 | 0.338983 | 76 | 0 | 996 |
| 340 | 238 | 1.025641 | 76 | 0 | 996 |
| 340 | 336 | 2.542373 | 76 | 0 | 996 |
| 340 | 420 | 1.190476 | 76 | 0 | 996 |
| 340 | 518 | 1.157503 | 76 | 0 | 996 |
| 340 | 577 | 1.216862 | 76 | 0 | 996 |
| 341 | 0 | 6.299213 | 70 | 0 | 858 |
| 341 | 70 | 9.090909 | 70 | 0 | 858 |
| 341 | 147 | 3.636364 | 70 | 0 | 858 |
| 341 | 232 | 8.633094 | 70 | 0 | 858 |
| 341 | 308 | 8.273824 | 70 | 0 | 858 |
| 341 | 392 | 4.528302 | 70 | 0 | 858 |
| 341 | 472 | 4.335484 | 70 | 0 | 858 |
| 341 | 562 | 5.16129 | 70 | 0 | 858 |
| 342 | 0 | 5.031447 | 77 | 0 | 459 |
| 342 | 28 | 3.106796 | 77 | 0 | 459 |
| 342 | 91 | 4.040404 | 77 | 0 | 459 |
| 342 | 161 | 2.539683 | 77 | 0 | 459 |
| 342 | 252 | 2.614379 | 77 | 0 | 459 |
| 342 | 329 | 3.333333 | 77 | 0 | 459 |
| 343 | 0 | 2.644628 | 73 | 0 | 848 |
| 343 | 35 | 3.137255 | 73 | 0 | 848 |
| 343 | 98 | 3.883495 | 73 | 0 | 848 |
| 343 | 161 | 3.448276 | 73 | 0 | 848 |
| 343 | 252 | 1.532567 | 73 | 0 | 848 |
| 343 | 343 | 3.636364 | 73 | 0 | 848 |
| 343 | 427 | 3.508772 | 73 | 0 | 848 |
| 343 | 483 | 2.689076 | 73 | 0 | 848 |
| 343 | 602 | 3.389831 | 73 | 0 | 848 |
| 344 | 0 | 2.941176 | 58 | 0 | 520 |
| 344 | 30 | 1.333333 | 58 | 0 | 520 |
| 344 | 91 | 1.333333 | 58 | 0 | 520 |
| 345 | 0 | 6.382979 | 74 | 1 | 1044 |
| 345 | 28 | 4.242424 | 74 | 1 | 1044 |
| 345 | 84 | 1.281465 | 74 | 1 | 1044 |
| 345 | 140 | 1.785714 | 74 | 1 | 1044 |
| 345 | 252 | 1.754386 | 74 | 1 | 1044 |
| 345 | 357 | 2.285714 | 74 | 1 | 1044 |
| 345 | 441 | 2.308802 | 74 | 1 | 1044 |

| | | | | | |
|---|---|---|---|---|---|
| 345 | 504 | 3.092784 | 74 | 1 | 1044 |
| 345 | 560 | 3 | 74 | 1 | 1044 |
| 346 | 0 | 4.210526 | 42 | 0 | 488 |
| 346 | 35 | 2.201835 | 42 | 0 | 488 |
| 346 | 49 | 2.666667 | 42 | 0 | 488 |
| 347 | 0 | 5.128205 | 50 | 1 | 114 |
| 347 | 28 | 4.918033 | 50 | 1 | 114 |
| 347 | 84 | 10.90909 | 50 | 1 | 114 |
| 348 | 0 | 2.162162 | 86 | 0 | 738 |
| 348 | 35 | 0.619469 | 86 | 0 | 738 |
| 348 | 98 | 0.625 | 86 | 0 | 738 |
| 348 | 154 | 2.745098 | 86 | 0 | 738 |
| 348 | 245 | 2.678571 | 86 | 0 | 738 |
| 348 | 336 | 4.166667 | 86 | 0 | 738 |
| 348 | 413 | 4.485981 | 86 | 0 | 738 |
| 348 | 511 | 5.504587 | 86 | 0 | 738 |
| 348 | 588 | 7.142857 | 86 | 0 | 738 |
| 349 | 0 | 9.6 | 87 | 0 | 407 |
| 349 | 28 | 9.6 | 87 | 0 | 407 |
| 349 | 84 | 9.836066 | 87 | 0 | 407 |
| 349 | 175 | 13.67521 | 87 | 0 | 407 |
| 349 | 252 | 10 | 87 | 0 | 407 |
| 349 | 364 | 8.205128 | 87 | 0 | 407 |
| 349 | 406 | 10.90909 | 87 | 0 | 407 |
| 350 | 0 | 5.972272 | 84 | 0 | 73 |
| 350 | 28 | 6.296852 | 84 | 0 | 73 |
| 351 | 0 | 2.068966 | 65 | 0 | 614 |
| 351 | 32 | 5.607477 | 65 | 0 | 614 |
| 351 | 60 | 4.247788 | 65 | 0 | 614 |
| 351 | 151 | 5.172414 | 65 | 0 | 614 |
| 351 | 238 | 10 | 65 | 0 | 614 |
| 351 | 333 | 1.95122 | 65 | 0 | 614 |
| 351 | 405 | 5.217391 | 65 | 0 | 614 |
| 351 | 501 | 3.703704 | 65 | 0 | 614 |
| 352 | 0 | 2.777778 | 87 | 0 | 1051 |
| 352 | 63 | 1.169591 | 87 | 0 | 1051 |
| 352 | 126 | 2.654867 | 87 | 0 | 1051 |
| 352 | 245 | 2.542373 | 87 | 0 | 1051 |
| 352 | 347 | 4.334365 | 87 | 0 | 1051 |

| | | | | | |
|---|---|---|---|---|---|
| 352 | 399 | 0.744417 | 87 | 0 | 1051 |
| 352 | 490 | 2.105263 | 87 | 0 | 1051 |
| 352 | 581 | 2.857143 | 87 | 0 | 1051 |
| 353 | 0 | 5.940594 | 67 | 0 | 970 |
| 353 | 28 | 5.714286 | 67 | 0 | 970 |
| 353 | 84 | 7.843137 | 67 | 0 | 970 |
| 353 | 151 | 5.26151 | 67 | 0 | 970 |
| 353 | 245 | 6 | 67 | 0 | 970 |
| 353 | 329 | 12.63158 | 67 | 0 | 970 |
| 353 | 420 | 15.38462 | 67 | 0 | 970 |
| 353 | 497 | 13.04348 | 67 | 0 | 970 |
| 353 | 588 | 9.056604 | 67 | 0 | 970 |
| 354 | 0 | 5.624372 | 78 | 1 | 75 |
| 354 | 23 | 14.35897 | 78 | 1 | 75 |
| 354 | 70 | 6.451613 | 78 | 1 | 75 |
| 355 | 0 | 2.162162 | 41 | 1 | 15 |
| 356 | 0 | 1.530612 | 59 | 0 | 457 |
| 356 | 105 | 5.177994 | 59 | 0 | 457 |
| 356 | 182 | 13.84615 | 59 | 0 | 457 |
| 356 | 245 | 9.230769 | 59 | 0 | 457 |
| 356 | 308 | 24.74227 | 59 | 0 | 457 |
| 356 | 413 | 28.21948 | 59 | 0 | 457 |
| 357 | 12 | 18.26087 | 56 | 0 | 64 |
| 357 | 63 | 15.25424 | 56 | 0 | 64 |
| 358 | 0 | 3.636364 | 56 | 0 | 212 |
| 358 | 27 | 5.405405 | 56 | 0 | 212 |
| 358 | 83 | 6.185567 | 56 | 0 | 212 |
| 358 | 172 | 5.704584 | 56 | 0 | 212 |
| 359 | 0 | 0.813008 | 71 | 0 | 960 |
| 359 | 28 | 0.757576 | 71 | 0 | 960 |
| 359 | 84 | 0.905172 | 71 | 0 | 960 |
| 359 | 164 | 1.223776 | 71 | 0 | 960 |
| 359 | 252 | 1.287208 | 71 | 0 | 960 |
| 359 | 329 | 1.061008 | 71 | 0 | 960 |
| 359 | 420 | 1.212121 | 71 | 0 | 960 |
| 359 | 504 | 1.212121 | 71 | 0 | 960 |
| 359 | 588 | 1.169591 | 71 | 0 | 960 |
| 360 | 0 | 2.330097 | 77 | 0 | 939 |
| 360 | 35 | 1.648352 | 77 | 0 | 939 |

| | | | | | |
|---|---|---|---|---|---|
| 360 | 84 | 3.030303 | 77 | 0 | 939 |
| 360 | 140 | 2.857143 | 77 | 0 | 939 |
| 360 | 266 | 2.44898 | 77 | 0 | 939 |
| 360 | 336 | 4.615385 | 77 | 0 | 939 |
| 360 | 406 | 9 | 77 | 0 | 939 |
| 360 | 532 | 7.2 | 77 | 0 | 939 |
| 360 | 595 | 8.080808 | 77 | 0 | 939 |
| 361 | 0 | 1.260504 | 57 | 0 | 1114 |
| 361 | 28 | 0.956938 | 57 | 0 | 1114 |
| 361 | 105 | 0.974026 | 57 | 0 | 1114 |
| 361 | 177 | 1.440576 | 57 | 0 | 1114 |
| 361 | 217 | 1.25 | 57 | 0 | 1114 |
| 361 | 329 | 1.293103 | 57 | 0 | 1114 |
| 361 | 385 | 1.327434 | 57 | 0 | 1114 |
| 361 | 497 | 1.351351 | 57 | 0 | 1114 |
| 361 | 609 | 1.339286 | 57 | 0 | 1114 |
| 362 | 0 | 3.305785 | 64 | 0 | 841 |
| 362 | 70 | 2.608696 | 64 | 0 | 841 |
| 362 | 126 | 5.504587 | 64 | 0 | 841 |
| 362 | 259 | 5.91133 | 64 | 0 | 841 |
| 362 | 343 | 3.053435 | 64 | 0 | 841 |
| 362 | 427 | 1.801802 | 64 | 0 | 841 |
| 362 | 511 | 1.724138 | 64 | 0 | 841 |
| 363 | 0 | 2.678571 | 81 | 1 | 71 |
| 363 | 28 | 2.678571 | 81 | 1 | 71 |
| 364 | 0 | 10.10526 | 61 | 1 | 932 |
| 364 | 35 | 6.78925 | 61 | 1 | 932 |
| 364 | 115 | 3.088803 | 61 | 1 | 932 |
| 364 | 169 | 3.088803 | 61 | 1 | 932 |
| 364 | 273 | 8.163265 | 61 | 1 | 932 |
| 364 | 315 | 10.60383 | 61 | 1 | 932 |
| 364 | 409 | 10.18388 | 61 | 1 | 932 |
| 364 | 504 | 7.272727 | 61 | 1 | 932 |
| 364 | 581 | 8.971963 | 61 | 1 | 932 |
| 365 | 0 | 1.804511 | 87 | 0 | 295 |
| 365 | 63 | 0.746269 | 87 | 0 | 295 |
| 365 | 147 | 0.847458 | 87 | 0 | 295 |
| 365 | 238 | 0.900901 | 87 | 0 | 295 |
| 365 | 294 | 0.8 | 87 | 0 | 295 |

| | | | | | |
|---|---|---|---|---|---|
| 366 | 0 | 0.865979 | 25 | 1 | 62 |
| 366 | 40 | 0.902062 | 25 | 1 | 62 |
| 367 | 0 | 6.165138 | 21 | 1 | 38 |
| 368 | 0 | 2.631579 | 58 | 0 | 707 |
| 368 | 63 | 2.654867 | 58 | 0 | 707 |
| 368 | 161 | 2.123894 | 58 | 0 | 707 |
| 368 | 259 | 1.980198 | 58 | 0 | 707 |
| 368 | 329 | 2.181818 | 58 | 0 | 707 |
| 368 | 399 | 2.702703 | 58 | 0 | 707 |
| 368 | 490 | 2.086957 | 58 | 0 | 707 |
| 368 | 581 | 2.753196 | 58 | 0 | 707 |
| 369 | 0 | 2.061856 | 71 | 0 | 449 |
| 369 | 84 | 2.285714 | 71 | 0 | 449 |
| 369 | 154 | 2 | 71 | 0 | 449 |
| 369 | 238 | 2.4 | 71 | 0 | 449 |
| 369 | 308 | 1.290323 | 71 | 0 | 449 |
| 369 | 448 | 1.348315 | 71 | 0 | 449 |
| 370 | 0 | 3.571429 | 82 | 0 | 1012 |
| 370 | 28 | 2.474227 | 82 | 0 | 1012 |
| 370 | 105 | 1.834862 | 82 | 0 | 1012 |
| 370 | 164 | 2.330097 | 82 | 0 | 1012 |
| 370 | 262 | 2.857143 | 82 | 0 | 1012 |
| 370 | 325 | 2.264151 | 82 | 0 | 1012 |
| 370 | 388 | 2.352941 | 82 | 0 | 1012 |
| 370 | 486 | 2.424242 | 82 | 0 | 1012 |
| 370 | 584 | 2.424242 | 82 | 0 | 1012 |
| 371 | 0 | 3.603604 | 67 | 1 | 423 |
| 371 | 28 | 3.773585 | 67 | 1 | 423 |
| 371 | 70 | 5.660377 | 67 | 1 | 423 |
| 371 | 161 | 5.309735 | 67 | 1 | 423 |
| 371 | 224 | 3.636364 | 67 | 1 | 423 |
| 371 | 335 | 8.408408 | 67 | 1 | 423 |
| 371 | 392 | 6.315789 | 67 | 1 | 423 |
| 372 | 0 | 14.28571 | 76 | 1 | 539 |
| 372 | 28 | 6.315789 | 76 | 1 | 539 |
| 372 | 115 | 10.42184 | 76 | 1 | 539 |
| 372 | 206 | 10.90909 | 76 | 1 | 539 |
| 372 | 294 | 5.097087 | 76 | 1 | 539 |
| 372 | 360 | 12.5 | 76 | 1 | 539 |

| | | | | | |
|---|---|---|---|---|---|
| 372 | 451 | 10.54945 | 76 | 1 | 539 |
| 373 | 0 | 5.042017 | 71 | 1 | 474 |
| 373 | 28 | 4.651163 | 71 | 1 | 474 |
| 373 | 83 | 1.840894 | 71 | 1 | 474 |
| 373 | 161 | 5.454545 | 71 | 1 | 474 |
| 373 | 245 | 3.865624 | 71 | 1 | 474 |
| 373 | 342 | 4.660194 | 71 | 1 | 474 |
| 373 | 412 | 6.521739 | 71 | 1 | 474 |
| 374 | 0 | 4.210526 | 72 | 1 | 638 |
| 374 | 35 | 5.504587 | 72 | 1 | 638 |
| 374 | 91 | 5.309735 | 72 | 1 | 638 |
| 374 | 210 | 4.033613 | 72 | 1 | 638 |
| 374 | 245 | 4.40367 | 72 | 1 | 638 |
| 374 | 315 | 3.603604 | 72 | 1 | 638 |
| 374 | 410 | 3.98293 | 72 | 1 | 638 |
| 374 | 497 | 3.636364 | 72 | 1 | 638 |
| 374 | 630 | 4.363636 | 72 | 1 | 638 |
| 375 | 0 | 24.11483 | 70 | 1 | 272 |
| 375 | 42 | 7.894737 | 70 | 1 | 272 |
| 375 | 98 | 17.47573 | 70 | 1 | 272 |
| 375 | 168 | 44.44444 | 70 | 1 | 272 |
| 375 | 252 | 16.82243 | 70 | 1 | 272 |
| 376 | 0 | 3.418803 | 77 | 1 | 656 |
| 376 | 21 | 3.478261 | 77 | 1 | 656 |
| 376 | 42 | 4.324324 | 77 | 1 | 656 |
| 376 | 154 | 2.521008 | 77 | 1 | 656 |
| 376 | 231 | 2.393844 | 77 | 1 | 656 |
| 376 | 332 | 3.988604 | 77 | 1 | 656 |
| 376 | 415 | 4.054054 | 77 | 1 | 656 |
| 376 | 483 | 10.71429 | 77 | 1 | 656 |
| 376 | 548 | 4.505229 | 77 | 1 | 656 |
| 377 | 28 | 8.571429 | 71 | 1 | 449 |
| 377 | 56 | 8.108108 | 71 | 1 | 449 |
| 377 | 147 | 7.826087 | 71 | 1 | 449 |
| 377 | 245 | 9.448819 | 71 | 1 | 449 |
| 377 | 301 | 4.477612 | 71 | 1 | 449 |
| 377 | 385 | 5.042017 | 71 | 1 | 449 |
| 378 | 0 | 3.296703 | 70 | 0 | 1211 |
| 378 | 28 | 3.296703 | 70 | 0 | 1211 |

| | | | | | |
|---|---|---|---|---|---|
| 378 | 84 | 6.896552 | 70 | 0 | 1211 |
| 378 | 168 | 5.940594 | 70 | 0 | 1211 |
| 378 | 252 | 20.86957 | 70 | 0 | 1211 |
| 378 | 334 | 5.357143 | 70 | 0 | 1211 |
| 378 | 418 | 21.81818 | 70 | 0 | 1211 |
| 378 | 502 | 14.98216 | 70 | 0 | 1211 |
| 378 | 586 | 10 | 70 | 0 | 1211 |
| 379 | 0 | 8.93617 | 73 | 1 | 317 |
| 379 | 21 | 12 | 73 | 1 | 317 |
| 379 | 77 | 5.217391 | 73 | 1 | 317 |
| 379 | 175 | 13.63636 | 73 | 1 | 317 |
| 379 | 259 | 14.28571 | 73 | 1 | 317 |
| 380 | 0 | 1.442308 | 85 | 0 | 1003 |
| 380 | 56 | 2.096986 | 85 | 0 | 1003 |
| 380 | 168 | 1.442308 | 85 | 0 | 1003 |
| 380 | 224 | 1.960784 | 85 | 0 | 1003 |
| 380 | 308 | 0.982801 | 85 | 0 | 1003 |
| 380 | 385 | 1.869159 | 85 | 0 | 1003 |
| 380 | 512 | 1.769912 | 85 | 0 | 1003 |
| 380 | 589 | 2.242991 | 85 | 0 | 1003 |
| 381 | 0 | 4.528302 | 48 | 0 | 854 |
| 381 | 28 | 5.940594 | 48 | 0 | 854 |
| 381 | 84 | 4.285714 | 48 | 0 | 854 |
| 381 | 175 | 5.769231 | 48 | 0 | 854 |
| 381 | 259 | 5.660377 | 48 | 0 | 854 |
| 381 | 329 | 2.859088 | 48 | 0 | 854 |
| 381 | 406 | 5.825243 | 48 | 0 | 854 |
| 381 | 504 | 21.42857 | 48 | 0 | 854 |
| 382 | 0 | 3.333333 | 79 | 0 | 169 |
| 382 | 98 | 3.2 | 79 | 0 | 169 |
| 382 | 168 | 4.444444 | 79 | 0 | 169 |
| 383 | 0 | 9.677419 | 72 | 0 | 1380 |
| 383 | 28 | 3.167421 | 72 | 0 | 1380 |
| 383 | 98 | 0.737813 | 72 | 0 | 1380 |
| 383 | 165 | 0.972222 | 72 | 0 | 1380 |
| 383 | 252 | 0.537634 | 72 | 0 | 1380 |
| 383 | 350 | 0.869565 | 72 | 0 | 1380 |
| 383 | 406 | 0.393314 | 72 | 0 | 1380 |
| 383 | 497 | 1.680672 | 72 | 0 | 1380 |

| | | | | | |
|---|---|---|---|---|---|
| 383 | 553 | 1.538462 | 72 | 0 | 1380 |
| 384 | 0 | 14.45783 | 70 | 0 | 91 |
| 384 | 35 | 8.791209 | 70 | 0 | 91 |
| 385 | 0 | 0.21172 | 72 | 0 | 661 |
| 385 | 29 | 0.221344 | 72 | 0 | 661 |
| 385 | 84 | 0.217391 | 72 | 0 | 661 |
| 385 | 195 | 0.209895 | 72 | 0 | 661 |
| 385 | 262 | 0.22755 | 72 | 0 | 661 |
| 385 | 356 | 5.405405 | 72 | 0 | 661 |
| 385 | 456 | 2.880658 | 72 | 0 | 661 |
| 385 | 517 | 2.453271 | 72 | 0 | 661 |
| 385 | 602 | 2.964008 | 72 | 0 | 661 |
| 386 | 0 | 2.419355 | 73 | 0 | 862 |
| 386 | 28 | 2.586207 | 73 | 0 | 862 |
| 386 | 82 | 2.393162 | 73 | 0 | 862 |
| 386 | 167 | 2.521008 | 73 | 0 | 862 |
| 386 | 252 | 1.709402 | 73 | 0 | 862 |
| 386 | 336 | 1.709402 | 73 | 0 | 862 |
| 386 | 420 | 1.652893 | 73 | 0 | 862 |
| 386 | 518 | 1.201201 | 73 | 0 | 862 |
| 386 | 602 | 1.818182 | 73 | 0 | 862 |
| 387 | 0 | 1.886792 | 80 | 0 | 580 |
| 387 | 56 | 1.834862 | 80 | 0 | 580 |
| 387 | 161 | 1.785714 | 80 | 0 | 580 |
| 387 | 217 | 0.961538 | 80 | 0 | 580 |
| 387 | 357 | 1.904762 | 80 | 0 | 580 |
| 387 | 413 | 1.724138 | 80 | 0 | 580 |
| 387 | 523 | 1.851852 | 80 | 0 | 580 |
| 387 | 579 | 1.851852 | 80 | 0 | 580 |
| 388 | 0 | 1.784197 | 63 | 0 | 568 |
| 388 | 86 | 7.76699 | 63 | 0 | 568 |
| 388 | 170 | 8.807339 | 63 | 0 | 568 |
| 388 | 238 | 8.807339 | 63 | 0 | 568 |
| 388 | 315 | 7.476636 | 63 | 0 | 568 |
| 388 | 399 | 7.54717 | 63 | 0 | 568 |
| 388 | 483 | 8.648649 | 63 | 0 | 568 |
| 389 | 0 | 1.788376 | 63 | 0 | 568 |
| 389 | 28 | 4.485981 | 63 | 0 | 568 |
| 389 | 63 | 5.309735 | 63 | 0 | 568 |

| | | | | | |
|---|---|---|---|---|---|
| 389 | 147 | 4.918033 | 63 | 0 | 568 |
| 389 | 203 | 4.761905 | 63 | 0 | 568 |
| 389 | 329 | 8.490566 | 63 | 0 | 568 |
| 389 | 420 | 4.597701 | 63 | 0 | 568 |
| 389 | 483 | 5.217391 | 63 | 0 | 568 |
| 389 | 567 | 8.450704 | 63 | 0 | 568 |
| 390 | 0 | 2.201835 | 54 | 0 | 946 |
| 390 | 28 | 1.327434 | 54 | 0 | 946 |
| 390 | 56 | 2.142857 | 54 | 0 | 946 |
| 390 | 147 | 2.123894 | 54 | 0 | 946 |
| 390 | 238 | 2.068966 | 54 | 0 | 946 |
| 390 | 294 | 0.917431 | 54 | 0 | 946 |
| 390 | 392 | 4.137931 | 54 | 0 | 946 |
| 390 | 518 | 4.615385 | 54 | 0 | 946 |
| 390 | 574 | 5.793103 | 54 | 0 | 946 |
| 391 | 0 | 3.84 | 74 | 0 | 820 |
| 391 | 91 | 1.886792 | 74 | 0 | 820 |
| 391 | 147 | 1.983471 | 74 | 0 | 820 |
| 391 | 245 | 3.902439 | 74 | 0 | 820 |
| 391 | 343 | 2.067183 | 74 | 0 | 820 |
| 391 | 413 | 2.702703 | 74 | 0 | 820 |
| 391 | 504 | 5.405405 | 74 | 0 | 820 |
| 391 | 588 | 9.917355 | 74 | 0 | 820 |
| 392 | 28 | 11.32075 | 75 | 0 | 987 |
| 392 | 84 | 5.263158 | 75 | 0 | 987 |
| 392 | 168 | 4.869565 | 75 | 0 | 987 |
| 392 | 226 | 2.298851 | 75 | 0 | 987 |
| 392 | 345 | 5.555556 | 75 | 0 | 987 |
| 392 | 408 | 5.555556 | 75 | 0 | 987 |
| 392 | 499 | 6.25 | 75 | 0 | 987 |
| 392 | 555 | 13.63636 | 75 | 0 | 987 |
| 393 | 0 | 2.941176 | 54 | 0 | 1273 |
| 393 | 91 | 7.33945 | 54 | 0 | 1273 |
| 393 | 133 | 5.882353 | 54 | 0 | 1273 |
| 393 | 245 | 5.769231 | 54 | 0 | 1273 |
| 393 | 301 | 5.714286 | 54 | 0 | 1273 |
| 393 | 448 | 4.705882 | 54 | 0 | 1273 |
| 393 | 504 | 5.106383 | 54 | 0 | 1273 |
| 393 | 567 | 6.25 | 54 | 0 | 1273 |

| | | | | | |
|---|---|---|---|---|---|
| 394 | 0 | 20.68966 | 82 | 1 | 173 |
| 394 | 14 | 7.5 | 82 | 1 | 173 |
| 394 | 70 | 7.058824 | 82 | 1 | 173 |
| 394 | 168 | 12.37113 | 82 | 1 | 173 |
| 395 | 0 | 2.201835 | 77 | 1 | 364 |
| 395 | 35 | 2.201835 | 77 | 1 | 364 |
| 395 | 70 | 2.4 | 77 | 1 | 364 |
| 395 | 140 | 2.4 | 77 | 1 | 364 |
| 395 | 231 | 4.660194 | 77 | 1 | 364 |
| 395 | 343 | 5.185185 | 77 | 1 | 364 |
| 396 | 0 | 6.185567 | 74 | 0 | 575 |
| 396 | 28 | 5.870021 | 74 | 0 | 575 |
| 396 | 84 | 5.309735 | 74 | 0 | 575 |
| 396 | 196 | 5.405405 | 74 | 0 | 575 |
| 396 | 252 | 5.357143 | 74 | 0 | 575 |
| 396 | 336 | 4.363636 | 74 | 0 | 575 |
| 396 | 427 | 4.444444 | 74 | 0 | 575 |
| 396 | 490 | 5.940594 | 74 | 0 | 575 |
| 396 | 574 | 5.714286 | 74 | 0 | 575 |
| 397 | 0 | 3.589744 | 85 | 0 | 799 |
| 397 | 26 | 3.929825 | 85 | 0 | 799 |
| 397 | 54 | 4.148148 | 85 | 0 | 799 |
| 397 | 182 | 5.280528 | 85 | 0 | 799 |
| 397 | 266 | 4.407713 | 85 | 0 | 799 |
| 397 | 329 | 9.230769 | 85 | 0 | 799 |
| 397 | 420 | 11.11111 | 85 | 0 | 799 |
| 397 | 518 | 9.142857 | 85 | 0 | 799 |
| 397 | 581 | 11.53846 | 85 | 0 | 799 |
| 398 | 0 | 1.415929 | 71 | 0 | 554 |
| 398 | 70 | 0.393314 | 71 | 0 | 554 |
| 398 | 175 | 0.415369 | 71 | 0 | 554 |
| 398 | 252 | 0.411523 | 71 | 0 | 554 |
| 398 | 322 | 0.8329 | 71 | 0 | 554 |
| 398 | 378 | 2.857143 | 71 | 0 | 554 |
| 398 | 483 | 0.909091 | 71 | 0 | 554 |
| 399 | 0 | 1.415929 | 46 | 0 | 624 |
| 399 | 35 | 1.666667 | 46 | 0 | 624 |
| 399 | 98 | 1.333333 | 46 | 0 | 624 |
| 399 | 168 | 1.652893 | 46 | 0 | 624 |

| | | | | | |
|---|---|---|---|---|---|
| 399 | 266 | 1.300813 | 46 | 0 | 624 |
| 400 | 0 | 0.732601 | 64 | 0 | 784 |
| 400 | 56 | 1.351351 | 64 | 0 | 784 |
| 400 | 161 | 1.666667 | 64 | 0 | 784 |
| 400 | 256 | 0.847458 | 64 | 0 | 784 |
| 400 | 340 | 1.388889 | 64 | 0 | 784 |
| 400 | 396 | 1.294299 | 64 | 0 | 784 |
| 400 | 511 | 0.663717 | 64 | 0 | 784 |
| 401 | 0 | 12.2449 | 80 | 1 | 552 |
| 401 | 28 | 15.05376 | 80 | 1 | 552 |
| 401 | 75 | 5.874126 | 80 | 1 | 552 |
| 401 | 154 | 10.90909 | 80 | 1 | 552 |
| 401 | 240 | 6.588235 | 80 | 1 | 552 |
| 401 | 310 | 15.2381 | 80 | 1 | 552 |
| 401 | 422 | 3.389831 | 80 | 1 | 552 |
| 401 | 490 | 7.207207 | 80 | 1 | 552 |
| 401 | 551 | 20.67268 | 80 | 1 | 552 |
| 402 | 0 | 5.274725 | 67 | 0 | 676 |
| 402 | 35 | 6.666667 | 67 | 0 | 676 |
| 402 | 63 | 12.5 | 67 | 0 | 676 |
| 402 | 154 | 7.76699 | 67 | 0 | 676 |
| 402 | 224 | 7.161125 | 67 | 0 | 676 |
| 402 | 343 | 12.63158 | 67 | 0 | 676 |
| 402 | 434 | 17.14286 | 67 | 0 | 676 |
| 402 | 504 | 16.93548 | 67 | 0 | 676 |
| 402 | 605 | 15.82418 | 67 | 0 | 676 |
| 403 | 28 | 1.190476 | 63 | 0 | 241 |
| 403 | 98 | 2.105263 | 63 | 0 | 241 |
| 403 | 189 | 2.285714 | 63 | 0 | 241 |
| 403 | 217 | 2.702703 | 63 | 0 | 241 |
| 404 | 0 | 1.229508 | 72 | 1 | 182 |
| 404 | 28 | 1.769912 | 72 | 1 | 182 |
| 404 | 84 | 4.081633 | 72 | 1 | 182 |
| 404 | 168 | 5.940594 | 72 | 1 | 182 |
| 405 | 0 | 2.644628 | 60 | 1 | 819 |
| 405 | 28 | 3.225806 | 60 | 1 | 819 |
| 405 | 84 | 3.2 | 60 | 1 | 819 |
| 405 | 147 | 2.666667 | 60 | 1 | 819 |
| 405 | 245 | 3.478261 | 60 | 1 | 819 |

| | | | | | |
|---|---|---|---|---|---|
| 405 | 336 | 3.361345 | 60 | 1 | 819 |
| 405 | 434 | 2.689076 | 60 | 1 | 819 |
| 405 | 511 | 3.571429 | 60 | 1 | 819 |
| 405 | 602 | 3.773585 | 60 | 1 | 819 |
| 406 | 0 | 12.2449 | 59 | 0 | 891 |
| 406 | 28 | 10.81081 | 59 | 0 | 891 |
| 406 | 84 | 9.69697 | 59 | 0 | 891 |
| 406 | 162 | 11.32075 | 59 | 0 | 891 |
| 406 | 266 | 15.12605 | 59 | 0 | 891 |
| 406 | 324 | 18.75 | 59 | 0 | 891 |
| 406 | 411 | 11.42857 | 59 | 0 | 891 |
| 406 | 495 | 9.266409 | 59 | 0 | 891 |
| 406 | 589 | 3.809524 | 59 | 0 | 891 |
| 407 | 0 | 1.851852 | 85 | 1 | 358 |
| 407 | 77 | 3.603604 | 85 | 1 | 358 |
| 407 | 182 | 1.346801 | 85 | 1 | 358 |
| 407 | 224 | 8.421053 | 85 | 1 | 358 |
| 407 | 308 | 7.142857 | 85 | 1 | 358 |
| 408 | 0 | 3.030303 | 74 | 0 | 1009 |
| 408 | 28 | 5.825243 | 74 | 0 | 1009 |
| 408 | 84 | 6.273338 | 74 | 0 | 1009 |
| 408 | 147 | 5.940594 | 74 | 0 | 1009 |
| 408 | 238 | 5.825243 | 74 | 0 | 1009 |
| 408 | 329 | 5.825243 | 74 | 0 | 1009 |
| 408 | 413 | 4.363636 | 74 | 0 | 1009 |
| 408 | 483 | 6.122449 | 74 | 0 | 1009 |
| 408 | 567 | 7.407407 | 74 | 0 | 1009 |
| 409 | 0 | 1.889764 | 60 | 1 | 446 |
| 409 | 35 | 1.517067 | 60 | 1 | 446 |
| 409 | 84 | 3.463918 | 60 | 1 | 446 |
| 409 | 175 | 3.034134 | 60 | 1 | 446 |
| 409 | 259 | 5.504587 | 60 | 1 | 446 |
| 409 | 350 | 10.60606 | 60 | 1 | 446 |
| 409 | 413 | 11.78947 | 60 | 1 | 446 |
| 410 | 0 | 1.309942 | 84 | 0 | 582 |
| 410 | 28 | 1.346801 | 84 | 0 | 582 |
| 410 | 91 | 1.827875 | 84 | 0 | 582 |
| 410 | 182 | 3.729604 | 84 | 0 | 582 |
| 410 | 273 | 4.057481 | 84 | 0 | 582 |

| | | | | | |
|---|---|---|---|---|---|
| 410 | 350 | 5.442177 | 84 | 0 | 582 |
| 410 | 413 | 5.555556 | 84 | 0 | 582 |
| 410 | 497 | 11.39241 | 84 | 0 | 582 |
| 410 | 553 | 10 | 84 | 0 | 582 |
| 411 | 0 | 4.958678 | 72 | 0 | 169 |
| 411 | 28 | 5.128205 | 72 | 0 | 169 |
| 411 | 56 | 5.087826 | 72 | 0 | 169 |
| 411 | 168 | 5.882353 | 72 | 0 | 169 |
| 412 | 0 | 10.21277 | 82 | 0 | 447 |
| 412 | 177 | 12.63158 | 82 | 0 | 447 |
| 412 | 240 | 12.90323 | 82 | 0 | 447 |
| 413 | 0 | 1.428571 | 86 | 0 | 1219 |
| 413 | 28 | 1.546392 | 86 | 0 | 1219 |
| 413 | 84 | 3.738318 | 86 | 0 | 1219 |
| 413 | 175 | 2.882883 | 86 | 0 | 1219 |
| 413 | 240 | 2.857143 | 86 | 0 | 1219 |
| 413 | 338 | 3.539823 | 86 | 0 | 1219 |
| 413 | 429 | 1.754386 | 86 | 0 | 1219 |
| 413 | 485 | 2.807018 | 86 | 0 | 1219 |
| 413 | 583 | 2.831858 | 86 | 0 | 1219 |
| 414 | 0 | 9.056604 | 77 | 0 | 681 |
| 414 | 28 | 36.73469 | 77 | 0 | 681 |
| 414 | 63 | 17.30769 | 77 | 0 | 681 |
| 414 | 182 | 3.921569 | 77 | 0 | 681 |
| 414 | 238 | 8.653846 | 77 | 0 | 681 |
| 414 | 350 | 7.741935 | 77 | 0 | 681 |
| 414 | 434 | 30.76923 | 77 | 0 | 681 |
| 414 | 462 | 31.85841 | 77 | 0 | 681 |
| 414 | 553 | 9.266409 | 77 | 0 | 681 |
| 415 | 0 | 1.061947 | 75 | 0 | 792 |
| 415 | 35 | 0.900901 | 75 | 0 | 792 |
| 415 | 77 | 0.934579 | 75 | 0 | 792 |
| 415 | 182 | 1.142857 | 75 | 0 | 792 |
| 415 | 252 | 1.111111 | 75 | 0 | 792 |
| 415 | 329 | 2.352941 | 75 | 0 | 792 |
| 415 | 441 | 1.801802 | 75 | 0 | 792 |
| 415 | 483 | 1.617251 | 75 | 0 | 792 |
| 415 | 574 | 0.869565 | 75 | 0 | 792 |
| 416 | 0 | 12.12121 | 65 | 1 | 800 |

| | | | | | |
|---|---|---|---|---|---|
| 416 | 28 | 8.807339 | 65 | 1 | 800 |
| 416 | 91 | 9.69697 | 65 | 1 | 800 |
| 416 | 182 | 11.88119 | 65 | 1 | 800 |
| 416 | 245 | 19.56522 | 65 | 1 | 800 |
| 416 | 346 | 21.22105 | 65 | 1 | 800 |
| 416 | 395 | 15.14423 | 65 | 1 | 800 |
| 416 | 518 | 21.42857 | 65 | 1 | 800 |
| 416 | 581 | 16 | 65 | 1 | 800 |
| 417 | 0 | 1.595442 | 79 | 0 | 981 |
| 417 | 31 | 1.923077 | 79 | 0 | 981 |
| 417 | 84 | 3.018868 | 79 | 0 | 981 |
| 417 | 175 | 5.405405 | 79 | 0 | 981 |
| 417 | 259 | 5.309735 | 79 | 0 | 981 |
| 417 | 350 | 4.363636 | 79 | 0 | 981 |
| 417 | 434 | 5.405405 | 79 | 0 | 981 |
| 417 | 504 | 4.324324 | 79 | 0 | 981 |
| 417 | 595 | 5.555556 | 79 | 0 | 981 |
| 418 | 0 | 1.101928 | 81 | 0 | 127 |
| 418 | 95 | 3.806503 | 81 | 0 | 127 |
| 419 | 0 | 4.705882 | 74 | 1 | 184 |
| 419 | 28 | 6.185567 | 74 | 1 | 184 |
| 419 | 56 | 6.315789 | 74 | 1 | 184 |
| 420 | 0 | 4.528302 | 52 | 1 | 51 |
| 421 | 0 | 1.148325 | 71 | 0 | 638 |
| 421 | 77 | 1.090909 | 71 | 0 | 638 |
| 421 | 154 | 0.470325 | 71 | 0 | 638 |
| 421 | 266 | 0.377868 | 71 | 0 | 638 |
| 421 | 350 | 0.442105 | 71 | 0 | 638 |
| 421 | 420 | 0.401914 | 71 | 0 | 638 |
| 421 | 518 | 0.451128 | 71 | 0 | 638 |
| 422 | 0 | 15.68627 | 73 | 0 | 647 |
| 422 | 34 | 13.67521 | 73 | 0 | 647 |
| 422 | 97 | 12.12121 | 73 | 0 | 647 |
| 422 | 167 | 2.364865 | 73 | 0 | 647 |
| 422 | 254 | 11.88119 | 73 | 0 | 647 |
| 422 | 338 | 9.160305 | 73 | 0 | 647 |
| 422 | 422 | 7.272727 | 73 | 0 | 647 |
| 422 | 513 | 13.35878 | 73 | 0 | 647 |
| 422 | 569 | 7.94702 | 73 | 0 | 647 |

| | | | | | |
|---|---|---|---|---|---|
| 423 | 0 | 0.616333 | 81 | 0 | 1388 |
| 423 | 35 | 1.530612 | 81 | 0 | 1388 |
| 423 | 80 | 1.149425 | 81 | 0 | 1388 |
| 423 | 203 | 1.376147 | 81 | 0 | 1388 |
| 423 | 245 | 1.851852 | 81 | 0 | 1388 |
| 423 | 336 | 0.909091 | 81 | 0 | 1388 |
| 423 | 392 | 0.695652 | 81 | 0 | 1388 |
| 424 | 0 | 2.777778 | 78 | 0 | 610 |
| 424 | 84 | 3.296703 | 78 | 0 | 610 |
| 424 | 175 | 3.448276 | 78 | 0 | 610 |
| 424 | 217 | 2.5 | 78 | 0 | 610 |
| 424 | 343 | 5.217391 | 78 | 0 | 610 |
| 424 | 413 | 3.389831 | 78 | 0 | 610 |
| 424 | 504 | 2.881152 | 78 | 0 | 610 |
| 424 | 609 | 2.631579 | 78 | 0 | 610 |
| 425 | 0 | 2.5 | 59 | 1 | 107 |
| 425 | 28 | 1.530612 | 59 | 1 | 107 |
| 425 | 77 | 3.53461 | 59 | 1 | 107 |
| 426 | 0 | 5.084746 | 77 | 0 | 1191 |
| 426 | 28 | 4.83871 | 77 | 0 | 1191 |
| 426 | 56 | 3.934426 | 77 | 0 | 1191 |
| 426 | 147 | 2.439024 | 77 | 0 | 1191 |
| 426 | 231 | 2.5 | 77 | 0 | 1191 |
| 426 | 315 | 5.454545 | 77 | 0 | 1191 |
| 426 | 399 | 2.631579 | 77 | 0 | 1191 |
| 426 | 483 | 2.142857 | 77 | 0 | 1191 |
| 426 | 574 | 5.263158 | 77 | 0 | 1191 |
| 427 | 0 | 0.909682 | 66 | 0 | 719 |
| 427 | 27 | 0.862069 | 66 | 0 | 719 |
| 427 | 56 | 0.434783 | 66 | 0 | 719 |
| 427 | 168 | 1.015965 | 66 | 0 | 719 |
| 427 | 263 | 2.020202 | 66 | 0 | 719 |
| 427 | 347 | 1.886792 | 66 | 0 | 719 |
| 427 | 431 | 1.980198 | 66 | 0 | 719 |
| 427 | 508 | 2.242991 | 66 | 0 | 719 |
| 428 | 0 | 16.82243 | 86 | 0 | 162 |
| 428 | 28 | 15.78947 | 86 | 0 | 162 |
| 428 | 91 | 12.13288 | 86 | 0 | 162 |
| 428 | 161 | 9.57265 | 86 | 0 | 162 |

| | | | | | |
|---|---|---|---|---|---|
| 429 | 0 | 1.904762 | 64 | 1 | 829 |
| 429 | 77 | 2.142857 | 64 | 1 | 829 |
| 429 | 133 | 9.411765 | 64 | 1 | 829 |
| 429 | 238 | 3.636364 | 64 | 1 | 829 |
| 429 | 329 | 10.32258 | 64 | 1 | 829 |
| 429 | 427 | 11.32075 | 64 | 1 | 829 |
| 429 | 469 | 14.27358 | 64 | 1 | 829 |
| 429 | 560 | 13.98058 | 64 | 1 | 829 |
| 430 | 0 | 2.242991 | 73 | 0 | 1107 |
| 430 | 28 | 1.851852 | 73 | 0 | 1107 |
| 430 | 70 | 2.830189 | 73 | 0 | 1107 |
| 430 | 167 | 1.960784 | 73 | 0 | 1107 |
| 430 | 252 | 1.941748 | 73 | 0 | 1107 |
| 430 | 294 | 5.660377 | 73 | 0 | 1107 |
| 430 | 391 | 5.607477 | 73 | 0 | 1107 |
| 430 | 475 | 4.324324 | 73 | 0 | 1107 |
| 430 | 594 | 3.960396 | 73 | 0 | 1107 |
| 431 | 0 | 1.260504 | 68 | 0 | 1011 |
| 432 | 0 | 5.357143 | 73 | 0 | 875 |
| 432 | 28 | 5.714286 | 73 | 0 | 875 |
| 432 | 76 | 3.794038 | 73 | 0 | 875 |
| 432 | 168 | 3.030303 | 73 | 0 | 875 |
| 432 | 279 | 1.6 | 73 | 0 | 875 |
| 432 | 337 | 1.682692 | 73 | 0 | 875 |
| 432 | 419 | 1.12782 | 73 | 0 | 875 |
| 432 | 503 | 0.8 | 73 | 0 | 875 |
| 432 | 587 | 0.775194 | 73 | 0 | 875 |
| 433 | 0 | 1.709402 | 83 | 0 | 575 |
| 433 | 28 | 1.666667 | 83 | 0 | 575 |
| 433 | 119 | 1.367521 | 83 | 0 | 575 |
| 433 | 147 | 1.709402 | 83 | 0 | 575 |
| 433 | 238 | 1.904762 | 83 | 0 | 575 |
| 434 | 0 | 0.47619 | 88 | 0 | 695 |
| 434 | 28 | 0.501253 | 88 | 0 | 695 |
| 434 | 91 | 0.565771 | 88 | 0 | 695 |
| 434 | 182 | 0.681818 | 88 | 0 | 695 |
| 434 | 245 | 1.142857 | 88 | 0 | 695 |
| 434 | 315 | 1.052632 | 88 | 0 | 695 |
| 434 | 420 | 1.111111 | 88 | 0 | 695 |

| | | | | | |
|---|---|---|---|---|---|
| 434 | 490 | 0.862069 | 88 | 0 | 695 |
| 434 | 588 | 0.742574 | 88 | 0 | 695 |
| 435 | 0 | 2.93501 | 76 | 0 | 607 |
| 435 | 27 | 2.434077 | 76 | 0 | 607 |
| 435 | 98 | 1.801802 | 76 | 0 | 607 |
| 435 | 189 | 2.912621 | 76 | 0 | 607 |
| 435 | 273 | 5.504587 | 76 | 0 | 607 |
| 435 | 357 | 4.958678 | 76 | 0 | 607 |
| 435 | 438 | 2.459016 | 76 | 0 | 607 |
| 435 | 515 | 2.479339 | 76 | 0 | 607 |
| 435 | 606 | 2.521008 | 76 | 0 | 607 |
| 436 | 0 | 0.913938 | 77 | 1 | 336 |
| 436 | 70 | 3.232323 | 77 | 1 | 336 |
| 436 | 189 | 3.404255 | 77 | 1 | 336 |
| 436 | 252 | 5.186252 | 77 | 1 | 336 |
| 437 | 0 | 4.067797 | 83 | 0 | 946 |
| 437 | 21 | 2.162162 | 83 | 0 | 946 |
| 437 | 56 | 1.709402 | 83 | 0 | 946 |
| 437 | 140 | 6.363636 | 83 | 0 | 946 |
| 437 | 231 | 3.478261 | 83 | 0 | 946 |
| 438 | 0 | 2.105263 | 64 | 1 | 152 |
| 438 | 35 | 3.609023 | 64 | 1 | 152 |
| 438 | 81 | 12.76596 | 64 | 1 | 152 |
| 439 | 0 | 6.315789 | 69 | 0 | 660 |
| 439 | 31 | 3.90108 | 69 | 0 | 660 |
| 439 | 60 | 6.122449 | 69 | 0 | 660 |
| 439 | 179 | 5.504587 | 69 | 0 | 660 |
| 439 | 235 | 4.752475 | 69 | 0 | 660 |
| 439 | 333 | 8.080808 | 69 | 0 | 660 |
| 439 | 389 | 4.8 | 69 | 0 | 660 |
| 439 | 525 | 13.86139 | 69 | 0 | 660 |
| 439 | 574 | 10.52632 | 69 | 0 | 660 |
| 440 | 0 | 2.181818 | 43 | 1 | 416 |
| 440 | 28 | 2.181818 | 43 | 1 | 416 |
| 440 | 63 | 3.137255 | 43 | 1 | 416 |
| 440 | 154 | 4.660194 | 43 | 1 | 416 |
| 440 | 254 | 9.795918 | 43 | 1 | 416 |
| 440 | 315 | 4.320432 | 43 | 1 | 416 |
| 440 | 415 | 4.347826 | 43 | 1 | 416 |

| | | | | | |
|---|---|---|---|---|---|
| 441 | 0 | 5.825243 | 71 | 1 | 659 |
| 441 | 28 | 6.722689 | 71 | 1 | 659 |
| 441 | 77 | 12.2449 | 71 | 1 | 659 |
| 441 | 133 | 11.21495 | 71 | 1 | 659 |
| 441 | 214 | 24.74227 | 71 | 1 | 659 |
| 441 | 291 | 14.15929 | 71 | 1 | 659 |
| 441 | 389 | 3.508772 | 71 | 1 | 659 |
| 441 | 473 | 22.85714 | 71 | 1 | 659 |
| 441 | 564 | 10.52632 | 71 | 1 | 659 |
| 442 | 0 | 6.122449 | 76 | 1 | 842 |
| 442 | 28 | 5.825243 | 76 | 1 | 842 |
| 442 | 84 | 4.8 | 76 | 1 | 842 |
| 442 | 147 | 12.12121 | 76 | 1 | 842 |
| 442 | 266 | 13.33333 | 76 | 1 | 842 |
| 442 | 329 | 16.98113 | 76 | 1 | 842 |
| 442 | 413 | 16.98113 | 76 | 1 | 842 |
| 442 | 497 | 18.36735 | 76 | 1 | 842 |
| 442 | 589 | 36.73469 | 76 | 1 | 842 |
| 443 | 0 | 2.307692 | 77 | 0 | 1044 |
| 443 | 35 | 2.105263 | 77 | 0 | 1044 |
| 443 | 98 | 2.439024 | 77 | 0 | 1044 |
| 443 | 189 | 1.311475 | 77 | 0 | 1044 |
| 443 | 273 | 1.190476 | 77 | 0 | 1044 |
| 443 | 322 | 1.017165 | 77 | 0 | 1044 |
| 443 | 413 | 1.709402 | 77 | 0 | 1044 |
| 443 | 504 | 1.513241 | 77 | 0 | 1044 |
| 443 | 595 | 0.775695 | 77 | 0 | 1044 |
| 444 | 0 | 5.172414 | 72 | 0 | 932 |
| 444 | 28 | 5.504587 | 72 | 0 | 932 |
| 444 | 84 | 4 | 72 | 0 | 932 |
| 444 | 168 | 3.030303 | 72 | 0 | 932 |
| 444 | 252 | 2.631579 | 72 | 0 | 932 |
| 444 | 336 | 2.586207 | 72 | 0 | 932 |
| 444 | 427 | 2.702703 | 72 | 0 | 932 |
| 444 | 525 | 2.222222 | 72 | 0 | 932 |
| 444 | 581 | 2.678571 | 72 | 0 | 932 |
| 445 | 0 | 2.604651 | 81 | 0 | 862 |
| 445 | 77 | 4.958678 | 81 | 0 | 862 |
| 445 | 161 | 1.322314 | 81 | 0 | 862 |

| | | | | | |
|---|---|---|---|---|---|
| 445 | 252 | 1.886792 | 81 | 0 | 862 |
| 445 | 343 | 1.495327 | 81 | 0 | 862 |
| 445 | 413 | 0.679694 | 81 | 0 | 862 |
| 445 | 490 | 1.568627 | 81 | 0 | 862 |
| 445 | 595 | 4.615385 | 81 | 0 | 862 |
| 446 | 0 | 3.198781 | 57 | 0 | 845 |
| 446 | 59 | 2.071006 | 57 | 0 | 845 |
| 446 | 182 | 0.294985 | 57 | 0 | 845 |
| 446 | 266 | 0.326797 | 57 | 0 | 845 |
| 446 | 350 | 0.320513 | 57 | 0 | 845 |
| 446 | 434 | 0.980392 | 57 | 0 | 845 |
| 447 | 0 | 2.631579 | 59 | 1 | 794 |
| 447 | 70 | 0.901578 | 59 | 1 | 794 |
| 447 | 154 | 1.602136 | 59 | 1 | 794 |
| 447 | 252 | 0.795756 | 59 | 1 | 794 |
| 447 | 325 | 1.869159 | 59 | 1 | 794 |
| 448 | 0 | 2.654867 | 63 | 0 | 624 |
| 448 | 28 | 2.181818 | 63 | 0 | 624 |
| 448 | 91 | 2.777778 | 63 | 0 | 624 |
| 448 | 161 | 4.705882 | 63 | 0 | 624 |
| 448 | 266 | 4.571429 | 63 | 0 | 624 |
| 448 | 329 | 6.461538 | 63 | 0 | 624 |
| 448 | 413 | 2.830189 | 63 | 0 | 624 |
| 448 | 518 | 4.301075 | 63 | 0 | 624 |
| 448 | 581 | 7.843137 | 63 | 0 | 624 |
| 449 | 0 | 2.504472 | 78 | 1 | 180 |
| 449 | 28 | 1.577761 | 78 | 1 | 180 |
| 449 | 84 | 5.825243 | 78 | 1 | 180 |
| 449 | 161 | 4.093567 | 78 | 1 | 180 |
| 450 | 0 | 0.946746 | 83 | 0 | 1233 |
| 450 | 28 | 0.92539 | 83 | 0 | 1233 |
| 450 | 84 | 0.961538 | 83 | 0 | 1233 |
| 450 | 168 | 0.956938 | 83 | 0 | 1233 |
| 450 | 245 | 1.118881 | 83 | 0 | 1233 |
| 450 | 336 | 1.201201 | 83 | 0 | 1233 |
| 450 | 420 | 1.834862 | 83 | 0 | 1233 |
| 450 | 504 | 4.23654 | 83 | 0 | 1233 |
| 450 | 581 | 6.060606 | 83 | 0 | 1233 |
| 451 | 0 | 2.325581 | 68 | 0 | 282 |

| | | | | | |
|---|---|---|---|---|---|
| 451 | 84 | 5.698779 | 68 | 0 | 282 |
| 452 | 0 | 0.740741 | 67 | 0 | 1017 |
| 452 | 28 | 0.704225 | 67 | 0 | 1017 |
| 452 | 90 | 1.058201 | 67 | 0 | 1017 |
| 452 | 168 | 1.111111 | 67 | 0 | 1017 |
| 452 | 252 | 1.440576 | 67 | 0 | 1017 |
| 452 | 343 | 0.490196 | 67 | 0 | 1017 |
| 452 | 427 | 0.550964 | 67 | 0 | 1017 |
| 452 | 511 | 1.092896 | 67 | 0 | 1017 |
| 452 | 595 | 1.111111 | 67 | 0 | 1017 |
| 453 | 0 | 1.6 | 86 | 0 | 610 |
| 453 | 28 | 2.330097 | 86 | 0 | 610 |
| 453 | 98 | 2.285714 | 86 | 0 | 610 |
| 453 | 161 | 2.752294 | 86 | 0 | 610 |
| 453 | 259 | 2.4 | 86 | 0 | 610 |
| 453 | 329 | 2.285714 | 86 | 0 | 610 |
| 453 | 392 | 2.376238 | 86 | 0 | 610 |
| 453 | 490 | 2.830189 | 86 | 0 | 610 |
| 453 | 609 | 3.061224 | 86 | 0 | 610 |
| 454 | 0 | 12 | 80 | 0 | 674 |
| 454 | 28 | 4.525862 | 80 | 0 | 674 |
| 454 | 85 | 4.8 | 80 | 0 | 674 |
| 454 | 169 | 5.645161 | 80 | 0 | 674 |
| 454 | 281 | 5.30303 | 80 | 0 | 674 |
| 454 | 341 | 3.937008 | 80 | 0 | 674 |
| 454 | 407 | 5 | 80 | 0 | 674 |
| 454 | 519 | 6.140351 | 80 | 0 | 674 |
| 454 | 603 | 5.555556 | 80 | 0 | 674 |
| 455 | 0 | 2.608696 | 74 | 0 | 152 |
| 455 | 28 | 2.631579 | 74 | 0 | 152 |
| 456 | 0 | 1.616162 | 71 | 1 | 369 |
| 456 | 35 | 1.649485 | 71 | 1 | 369 |
| 456 | 70 | 1.454545 | 71 | 1 | 369 |
| 456 | 168 | 1.923077 | 71 | 1 | 369 |
| 456 | 252 | 2.588997 | 71 | 1 | 369 |
| 456 | 329 | 3.921569 | 71 | 1 | 369 |
| 457 | 0 | 2.542373 | 82 | 1 | 126 |
| 457 | 27 | 2.521008 | 82 | 1 | 126 |
| 457 | 56 | 5.084746 | 82 | 1 | 126 |

| | | | | | |
|---|---|---|---|---|---|
| 458 | 0 | 5.321508 | 63 | 0 | 816 |
| 458 | 21 | 5.236364 | 63 | 0 | 816 |
| 458 | 77 | 8.490566 | 63 | 0 | 816 |
| 458 | 168 | 5.950413 | 63 | 0 | 816 |
| 458 | 269 | 4.744869 | 63 | 0 | 816 |
| 458 | 325 | 10.61947 | 63 | 0 | 816 |
| 458 | 420 | 20.86957 | 63 | 0 | 816 |
| 458 | 511 | 15.70093 | 63 | 0 | 816 |
| 458 | 563 | 14.7541 | 63 | 0 | 816 |
| 459 | 0 | 1.694915 | 62 | 1 | 491 |
| 459 | 41 | 1.886792 | 62 | 1 | 491 |
| 459 | 127 | 1.754386 | 62 | 1 | 491 |
| 459 | 212 | 7.33945 | 62 | 1 | 491 |
| 459 | 336 | 11.93464 | 62 | 1 | 491 |
| 459 | 411 | 11.53846 | 62 | 1 | 491 |
| 459 | 490 | 25.71429 | 62 | 1 | 491 |
| 460 | 0 | 2.896552 | 67 | 1 | 286 |
| 460 | 101 | 2.142857 | 67 | 1 | 286 |
| 460 | 164 | 4.571429 | 67 | 1 | 286 |
| 460 | 234 | 3.296703 | 67 | 1 | 286 |
| 461 | 0 | 17.82178 | 87 | 0 | 898 |
| 461 | 28 | 7.964602 | 87 | 0 | 898 |
| 461 | 84 | 5.454545 | 87 | 0 | 898 |
| 461 | 168 | 5.555556 | 87 | 0 | 898 |
| 461 | 252 | 5.504587 | 87 | 0 | 898 |
| 461 | 336 | 5.309735 | 87 | 0 | 898 |
| 461 | 420 | 5.263158 | 87 | 0 | 898 |
| 461 | 504 | 5.769231 | 87 | 0 | 898 |
| 461 | 588 | 5.714286 | 87 | 0 | 898 |
| 462 | 0 | 2.181818 | 65 | 0 | 1058 |
| 462 | 35 | 4.081633 | 65 | 0 | 1058 |
| 462 | 84 | 2.330097 | 65 | 0 | 1058 |
| 462 | 182 | 4 | 65 | 0 | 1058 |
| 462 | 266 | 4.892966 | 65 | 0 | 1058 |
| 462 | 343 | 3.571429 | 65 | 0 | 1058 |
| 462 | 434 | 3.393939 | 65 | 0 | 1058 |
| 462 | 527 | 4.081633 | 65 | 0 | 1058 |
| 462 | 590 | 4.615385 | 65 | 0 | 1058 |
| 463 | 0 | 2.970297 | 86 | 0 | 438 |

| | | | | | |
|---|---|---|---|---|---|
| 463 | 25 | 5.882353 | 86 | 0 | 438 |
| 463 | 84 | 5.769231 | 86 | 0 | 438 |
| 463 | 175 | 8.648649 | 86 | 0 | 438 |
| 463 | 246 | 22.24184 | 86 | 0 | 438 |
| 464 | 0 | 2.380952 | 65 | 1 | 472 |
| 464 | 28 | 2.016807 | 65 | 1 | 472 |
| 464 | 98 | 1.889764 | 65 | 1 | 472 |
| 464 | 182 | 1.724138 | 65 | 1 | 472 |
| 464 | 245 | 2.970297 | 65 | 1 | 472 |
| 464 | 336 | 2.016807 | 65 | 1 | 472 |
| 464 | 441 | 2.222222 | 65 | 1 | 472 |
| 465 | 0 | 14.11765 | 84 | 0 | 947 |
| 465 | 35 | 5.574913 | 84 | 0 | 947 |
| 465 | 84 | 3.10278 | 84 | 0 | 947 |
| 465 | 175 | 11.21495 | 84 | 0 | 947 |
| 465 | 259 | 5 | 84 | 0 | 947 |
| 465 | 343 | 11.32075 | 84 | 0 | 947 |
| 465 | 427 | 11.53846 | 84 | 0 | 947 |
| 465 | 511 | 5.454545 | 84 | 0 | 947 |
| 465 | 595 | 5.940594 | 84 | 0 | 947 |
| 466 | 0 | 2.830189 | 74 | 0 | 639 |
| 466 | 35 | 1.617874 | 74 | 0 | 639 |
| 466 | 163 | 2.631579 | 74 | 0 | 639 |
| 466 | 219 | 2.930403 | 74 | 0 | 639 |
| 466 | 317 | 2.742857 | 74 | 0 | 639 |
| 466 | 415 | 2.857143 | 74 | 0 | 639 |
| 466 | 464 | 3.692308 | 74 | 0 | 639 |
| 466 | 603 | 3.75 | 74 | 0 | 639 |
| 467 | 0 | 2.608696 | 75 | 1 | 858 |
| 467 | 28 | 5.825243 | 75 | 1 | 858 |
| 467 | 84 | 11.21495 | 75 | 1 | 858 |
| 467 | 168 | 10.71429 | 75 | 1 | 858 |
| 467 | 252 | 16.66667 | 75 | 1 | 858 |
| 467 | 350 | 14.69388 | 75 | 1 | 858 |
| 467 | 426 | 16.55172 | 75 | 1 | 858 |
| 468 | 0 | 6.857143 | 65 | 1 | 432 |
| 468 | 42 | 6 | 65 | 1 | 432 |
| 468 | 98 | 6.25 | 65 | 1 | 432 |
| 468 | 154 | 6.315789 | 65 | 1 | 432 |

| | | | | | |
|---|---|---|---|---|---|
| 468 | 266 | 4.247788 | 65 | 1 | 432 |
| 468 | 336 | 5.825243 | 65 | 1 | 432 |
| 469 | 0 | 11.76471 | 67 | 1 | 195 |
| 469 | 27 | 11.00917 | 67 | 1 | 195 |
| 469 | 83 | 5.217391 | 67 | 1 | 195 |
| 469 | 158 | 11.00917 | 67 | 1 | 195 |
| 470 | 0 | 2.859574 | 74 | 1 | 648 |
| 470 | 28 | 2.028986 | 74 | 1 | 648 |
| 470 | 100 | 2.121212 | 74 | 1 | 648 |
| 470 | 198 | 1.339286 | 74 | 1 | 648 |
| 470 | 233 | 1.577761 | 74 | 1 | 648 |
| 470 | 329 | 1.650457 | 74 | 1 | 648 |
| 470 | 408 | 1.102941 | 74 | 1 | 648 |
| 470 | 527 | 1.166748 | 74 | 1 | 648 |
| 470 | 601 | 5.25 | 74 | 1 | 648 |
| 471 | 0 | 6.25 | 40 | 1 | 184 |
| 471 | 28 | 4.848485 | 40 | 1 | 184 |
| 471 | 63 | 5.882353 | 40 | 1 | 184 |
| 471 | 119 | 12.12121 | 40 | 1 | 184 |
| 472 | 0 | 2.654867 | 86 | 0 | 799 |
| 472 | 91 | 1.95713 | 86 | 0 | 799 |
| 472 | 147 | 2.654867 | 86 | 0 | 799 |
| 472 | 273 | 2.105263 | 86 | 0 | 799 |
| 472 | 392 | 4.897959 | 86 | 0 | 799 |
| 472 | 518 | 5.825243 | 86 | 0 | 799 |
| 472 | 574 | 5.660377 | 86 | 0 | 799 |
| 473 | 28 | 2.608696 | 67 | 0 | 941 |
| 473 | 84 | 2.752294 | 67 | 0 | 941 |
| 473 | 147 | 2.727273 | 67 | 0 | 941 |
| 473 | 231 | 2.654867 | 67 | 0 | 941 |
| 473 | 322 | 2.568807 | 67 | 0 | 941 |
| 473 | 413 | 5.825243 | 67 | 0 | 941 |
| 473 | 504 | 8.648649 | 67 | 0 | 941 |
| 473 | 567 | 11.00917 | 67 | 0 | 941 |
| 474 | 0 | 5.825243 | 79 | 1 | 262 |
| 474 | 28 | 4.660194 | 79 | 1 | 262 |
| 474 | 91 | 6.666667 | 79 | 1 | 262 |
| 474 | 175 | 14.45161 | 79 | 1 | 262 |
| 474 | 250 | 15.05376 | 79 | 1 | 262 |

| | | | | | |
|---|---|---|---|---|---|
| 475 | 0 | 6.25 | 86 | 0 | 1065 |
| 475 | 84 | 16.82243 | 86 | 0 | 1065 |
| 475 | 168 | 3.529412 | 86 | 0 | 1065 |
| 475 | 245 | 9 | 86 | 0 | 1065 |
| 475 | 301 | 7.826087 | 86 | 0 | 1065 |
| 475 | 420 | 7.272727 | 86 | 0 | 1065 |
| 475 | 483 | 6.666667 | 86 | 0 | 1065 |
| 475 | 602 | 7.826087 | 86 | 0 | 1065 |
| 476 | 35 | 5.042017 | 72 | 0 | 1025 |
| 476 | 91 | 2.592593 | 72 | 0 | 1025 |
| 476 | 182 | 2.5 | 72 | 0 | 1025 |
| 476 | 273 | 2.678571 | 72 | 0 | 1025 |
| 476 | 357 | 5.607477 | 72 | 0 | 1025 |
| 476 | 413 | 4.363636 | 72 | 0 | 1025 |
| 476 | 504 | 5.309735 | 72 | 0 | 1025 |
| 476 | 623 | 4.102564 | 72 | 0 | 1025 |
| 477 | 0 | 4.8 | 67 | 1 | 550 |
| 477 | 28 | 2 | 67 | 1 | 550 |
| 477 | 88 | 5.607477 | 67 | 1 | 550 |
| 477 | 179 | 4.285714 | 67 | 1 | 550 |
| 477 | 259 | 5.769231 | 67 | 1 | 550 |
| 477 | 317 | 5.882353 | 67 | 1 | 550 |
| 477 | 408 | 6.153846 | 67 | 1 | 550 |
| 477 | 501 | 22.85714 | 67 | 1 | 550 |
| 478 | 0 | 2.264151 | 69 | 0 | 665 |
| 478 | 27 | 2.830189 | 69 | 0 | 665 |
| 478 | 111 | 2.803738 | 69 | 0 | 665 |
| 478 | 139 | 2.941176 | 69 | 0 | 665 |
| 478 | 251 | 3.168317 | 69 | 0 | 665 |
| 478 | 307 | 5.128205 | 69 | 0 | 665 |
| 478 | 412 | 1.777778 | 69 | 0 | 665 |
| 478 | 475 | 1.777778 | 69 | 0 | 665 |
| 479 | 0 | 11.53846 | 69 | 1 | 319 |
| 479 | 28 | 17.14286 | 69 | 1 | 319 |
| 479 | 84 | 17.64706 | 69 | 1 | 319 |
| 479 | 126 | 18.75 | 69 | 1 | 319 |
| 479 | 252 | 11.21495 | 69 | 1 | 319 |
| 480 | 0 | 5.454545 | 73 | 0 | 876 |
| 480 | 28 | 5.217391 | 73 | 0 | 876 |

| | | | | | |
|---|---|---|---|---|---|
| 480 | 84 | 5.607477 | 73 | 0 | 876 |
| 480 | 168 | 5.660377 | 73 | 0 | 876 |
| 480 | 268 | 5.172414 | 73 | 0 | 876 |
| 480 | 336 | 7.476636 | 73 | 0 | 876 |
| 480 | 413 | 5.607477 | 73 | 0 | 876 |
| 480 | 504 | 8.737864 | 73 | 0 | 876 |
| 480 | 595 | 6.428571 | 73 | 0 | 876 |
| 481 | 0 | 1.454545 | 67 | 0 | 1282 |
| 481 | 70 | 2.105263 | 67 | 0 | 1282 |
| 481 | 175 | 1.544402 | 67 | 0 | 1282 |
| 481 | 273 | 1.602136 | 67 | 0 | 1282 |
| 481 | 350 | 2.222222 | 67 | 0 | 1282 |
| 481 | 406 | 1.320132 | 67 | 0 | 1282 |
| 481 | 525 | 1.5 | 67 | 0 | 1282 |
| 481 | 581 | 1.470588 | 67 | 0 | 1282 |
| 482 | 0 | 12.37113 | 79 | 0 | 565 |
| 482 | 28 | 11.76471 | 79 | 0 | 565 |
| 482 | 84 | 8.888889 | 79 | 0 | 565 |
| 482 | 182 | 11.76471 | 79 | 0 | 565 |
| 482 | 266 | 9.677419 | 79 | 0 | 565 |
| 482 | 322 | 10.43802 | 79 | 0 | 565 |
| 482 | 413 | 10.38961 | 79 | 0 | 565 |
| 482 | 494 | 12.06897 | 79 | 0 | 565 |
| 483 | 0 | 0.720721 | 73 | 0 | 121 |
| 483 | 28 | 0.909091 | 73 | 0 | 121 |
| 483 | 84 | 0.934579 | 73 | 0 | 121 |
| 484 | 0 | 5.405405 | 78 | 1 | 729 |
| 484 | 25 | 5.217391 | 78 | 1 | 729 |
| 484 | 81 | 6.408545 | 78 | 1 | 729 |
| 484 | 186 | 5.714286 | 78 | 1 | 729 |
| 484 | 256 | 5.882353 | 78 | 1 | 729 |
| 484 | 312 | 1.415929 | 78 | 1 | 729 |
| 484 | 375 | 5.263158 | 78 | 1 | 729 |
| 484 | 536 | 9.230769 | 78 | 1 | 729 |
| 484 | 606 | 10 | 78 | 1 | 729 |
| 485 | 0 | 2.830189 | 77 | 0 | 868 |
| 485 | 28 | 2.93501 | 77 | 0 | 868 |
| 485 | 141 | 2.068966 | 77 | 0 | 868 |
| 485 | 238 | 2.564103 | 77 | 0 | 868 |

| | | | | | |
|---|---|---|---|---|---|
| 485 | 294 | 2.654867 | 77 | 0 | 868 |
| 485 | 413 | 2.631579 | 77 | 0 | 868 |
| 485 | 469 | 1.666667 | 77 | 0 | 868 |
| 485 | 588 | 2.439024 | 77 | 0 | 868 |
| 486 | 0 | 7.619048 | 53 | 1 | 672 |
| 486 | 88 | 11.94879 | 53 | 1 | 672 |
| 486 | 144 | 20.09569 | 53 | 1 | 672 |
| 486 | 238 | 23.07692 | 53 | 1 | 672 |
| 486 | 329 | 24.37137 | 53 | 1 | 672 |
| 486 | 410 | 30.56398 | 53 | 1 | 672 |
| 486 | 483 | 13.43642 | 53 | 1 | 672 |
| 486 | 553 | 21.81818 | 53 | 1 | 672 |
| 487 | 0 | 0.674916 | 60 | 1 | 924 |
| 487 | 98 | 0.857143 | 60 | 1 | 924 |
| 487 | 154 | 4.173913 | 60 | 1 | 924 |
| 487 | 252 | 5.217391 | 60 | 1 | 924 |
| 487 | 329 | 4.363636 | 60 | 1 | 924 |
| 487 | 427 | 23.52941 | 60 | 1 | 924 |
| 488 | 0 | 0.535714 | 70 | 0 | 87 |
| 488 | 28 | 0.550459 | 70 | 0 | 87 |
| 488 | 84 | 0.606061 | 70 | 0 | 87 |
| 489 | 0 | 4.411765 | 88 | 0 | 620 |
| 489 | 84 | 6.722689 | 88 | 0 | 620 |
| 489 | 168 | 2.521008 | 88 | 0 | 620 |
| 489 | 238 | 2.564103 | 88 | 0 | 620 |
| 489 | 322 | 2.727273 | 88 | 0 | 620 |
| 489 | 406 | 2.727273 | 88 | 0 | 620 |
| 489 | 497 | 2.631579 | 88 | 0 | 620 |
| 490 | 0 | 2.181818 | 70 | 0 | 890 |
| 490 | 28 | 2.702703 | 70 | 0 | 890 |
| 490 | 84 | 2.123894 | 70 | 0 | 890 |
| 490 | 147 | 2.142857 | 70 | 0 | 890 |
| 490 | 238 | 1.25 | 70 | 0 | 890 |
| 490 | 322 | 1.845748 | 70 | 0 | 890 |
| 490 | 420 | 2.702703 | 70 | 0 | 890 |
| 490 | 504 | 2.631579 | 70 | 0 | 890 |
| 490 | 588 | 2.608696 | 70 | 0 | 890 |
| 491 | 0 | 1.801802 | 75 | 0 | 834 |
| 491 | 91 | 1.558442 | 75 | 0 | 834 |

| | | | | | |
|---|---|---|---|---|---|
| 491 | 168 | 1.834862 | 75 | 0 | 834 |
| 491 | 252 | 1.769912 | 75 | 0 | 834 |
| 491 | 336 | 3.603604 | 75 | 0 | 834 |
| 491 | 378 | 3.478261 | 75 | 0 | 834 |
| 491 | 511 | 1.769912 | 75 | 0 | 834 |
| 491 | 595 | 3.508772 | 75 | 0 | 834 |
| 492 | 0 | 4.948454 | 63 | 1 | 342 |
| 492 | 35 | 4.571429 | 63 | 1 | 342 |
| 492 | 70 | 1.6 | 63 | 1 | 342 |
| 492 | 175 | 4.878757 | 63 | 1 | 342 |
| 492 | 246 | 3.692308 | 63 | 1 | 342 |
| 492 | 337 | 6.440072 | 63 | 1 | 342 |
| 493 | 0 | 12 | 54 | 1 | 612 |
| 493 | 29 | 7.142857 | 54 | 1 | 612 |
| 493 | 112 | 6.666667 | 54 | 1 | 612 |
| 493 | 182 | 7.559055 | 54 | 1 | 612 |
| 494 | 0 | 1.209677 | 65 | 0 | 743 |
| 494 | 28 | 1.229508 | 65 | 0 | 743 |
| 494 | 84 | 2.752294 | 65 | 0 | 743 |
| 494 | 168 | 2.727273 | 65 | 0 | 743 |
| 494 | 259 | 3.883495 | 65 | 0 | 743 |
| 494 | 343 | 4.067797 | 65 | 0 | 743 |
| 494 | 427 | 2.439024 | 65 | 0 | 743 |
| 494 | 511 | 1.147776 | 65 | 0 | 743 |
| 494 | 602 | 1.217039 | 65 | 0 | 743 |
| 495 | 28 | 1.271186 | 65 | 0 | 799 |
| 495 | 84 | 2.608696 | 65 | 0 | 799 |
| 495 | 168 | 0.191296 | 65 | 0 | 799 |
| 495 | 259 | 0.892857 | 65 | 0 | 799 |
| 495 | 339 | 0.172265 | 65 | 0 | 799 |
| 495 | 407 | 0.26296 | 65 | 0 | 799 |
| 495 | 504 | 2.475685 | 65 | 0 | 799 |
| 495 | 616 | 2.654867 | 65 | 0 | 799 |
| 496 | 0 | 3.539823 | 37 | 1 | 309 |
| 496 | 28 | 2.912621 | 37 | 1 | 309 |
| 496 | 84 | 1.851852 | 37 | 1 | 309 |
| 496 | 168 | 1.785714 | 37 | 1 | 309 |
| 496 | 252 | 4.123711 | 37 | 1 | 309 |
| 497 | 0 | 5.899705 | 69 | 0 | 743 |

| | | | | | |
|---|---|---|---|---|---|
| 497 | 28 | 6.837607 | 69 | 0 | 743 |
| 497 | 91 | 8.474576 | 69 | 0 | 743 |
| 497 | 175 | 8.403361 | 69 | 0 | 743 |
| 497 | 259 | 8.547009 | 69 | 0 | 743 |
| 497 | 322 | 7.476636 | 69 | 0 | 743 |
| 497 | 427 | 6.896552 | 69 | 0 | 743 |
| 497 | 518 | 8.849558 | 69 | 0 | 743 |
| 497 | 574 | 8.62069 | 69 | 0 | 743 |
| 498 | 0 | 0.839161 | 78 | 0 | 677 |
| 498 | 21 | 0.886918 | 78 | 0 | 677 |
| 498 | 98 | 1.73913 | 78 | 0 | 677 |
| 499 | 0 | 2.654867 | 72 | 1 | 311 |
| 499 | 28 | 1.212121 | 72 | 1 | 311 |
| 499 | 91 | 0.478469 | 72 | 1 | 311 |
| 499 | 182 | 0.487013 | 72 | 1 | 311 |
| 499 | 245 | 1.473684 | 72 | 1 | 311 |
| 499 | 302 | 3.349282 | 72 | 1 | 311 |
| 500 | 0 | 5.405405 | 69 | 0 | 701 |
| 500 | 28 | 4.485981 | 69 | 0 | 701 |
| 500 | 105 | 4.301075 | 69 | 0 | 701 |
| 500 | 168 | 10.90909 | 69 | 0 | 701 |
| 500 | 266 | 10.54945 | 69 | 0 | 701 |
| 500 | 336 | 6.956522 | 69 | 0 | 701 |
| 500 | 427 | 6.349206 | 69 | 0 | 701 |
| 500 | 511 | 7.476636 | 69 | 0 | 701 |
| 500 | 553 | 7.692308 | 69 | 0 | 701 |
| 501 | 0 | 2.654867 | 78 | 0 | 29 |
| 502 | 0 | 5.405405 | 79 | 1 | 834 |
| 502 | 28 | 2.654867 | 79 | 1 | 834 |
| 502 | 84 | 4.444444 | 79 | 1 | 834 |
| 502 | 168 | 12.5 | 79 | 1 | 834 |
| 502 | 252 | 12.37113 | 79 | 1 | 834 |
| 502 | 336 | 12.5 | 79 | 1 | 834 |
| 502 | 420 | 14.54545 | 79 | 1 | 834 |
| 502 | 511 | 21.95122 | 79 | 1 | 834 |
| 502 | 588 | 20.79208 | 79 | 1 | 834 |
| 503 | 0 | 1.769912 | 51 | 0 | 645 |
| 503 | 28 | 1.754386 | 51 | 0 | 645 |
| 503 | 84 | 1.754386 | 51 | 0 | 645 |

| | | | | | |
|---|---|---|---|---|---|
| 503 | 182 | 1.869159 | 51 | 0 | 645 |
| 503 | 245 | 2.912621 | 51 | 0 | 645 |
| 503 | 336 | 2.807018 | 51 | 0 | 645 |
| 503 | 406 | 2.782609 | 51 | 0 | 645 |
| 503 | 511 | 2.831858 | 51 | 0 | 645 |
| 503 | 567 | 3.921569 | 51 | 0 | 645 |
| 504 | 0 | 11.03448 | 73 | 0 | 778 |
| 504 | 36 | 11.42857 | 73 | 0 | 778 |
| 504 | 140 | 18.18182 | 73 | 0 | 778 |
| 504 | 259 | 11.42857 | 73 | 0 | 778 |
| 504 | 315 | 13.84615 | 73 | 0 | 778 |
| 504 | 440 | 9.090909 | 73 | 0 | 778 |
| 504 | 511 | 11.00917 | 73 | 0 | 778 |
| 504 | 539 | 12.2449 | 73 | 0 | 778 |
| 505 | 28 | 10.83871 | 72 | 0 | 758 |
| 505 | 81 | 16.81682 | 72 | 0 | 758 |
| 505 | 151 | 11.5942 | 72 | 0 | 758 |
| 505 | 242 | 9.375 | 72 | 0 | 758 |
| 505 | 333 | 9.022556 | 72 | 0 | 758 |
| 505 | 424 | 7.092199 | 72 | 0 | 758 |
| 505 | 487 | 4.96124 | 72 | 0 | 758 |
| 505 | 578 | 7.03125 | 72 | 0 | 758 |
| 506 | 0 | 11.42857 | 75 | 0 | 1162 |
| 506 | 26 | 12.12121 | 75 | 0 | 1162 |
| 506 | 82 | 13.18681 | 75 | 0 | 1162 |
| 506 | 174 | 15.84906 | 75 | 0 | 1162 |
| 506 | 265 | 14.15929 | 75 | 0 | 1162 |
| 506 | 432 | 10.45752 | 75 | 0 | 1162 |
| 506 | 481 | 9.677419 | 75 | 0 | 1162 |
| 506 | 565 | 10.25328 | 75 | 0 | 1162 |
| 507 | 0 | 2.105263 | 67 | 1 | 1040 |
| 507 | 70 | 2.142857 | 67 | 1 | 1040 |
| 507 | 168 | 2.941176 | 67 | 1 | 1040 |
| 507 | 247 | 2.142857 | 67 | 1 | 1040 |
| 507 | 322 | 2.162162 | 67 | 1 | 1040 |
| 507 | 392 | 2.330097 | 67 | 1 | 1040 |
| 507 | 490 | 2.777778 | 67 | 1 | 1040 |
| 507 | 553 | 2.222222 | 67 | 1 | 1040 |
| 508 | 0 | 3.428571 | 92 | 0 | 681 |

| | | | | | |
|---|---|---|---|---|---|
| 508 | 77 | 7.792208 | 92 | 0 | 681 |
| 508 | 168 | 8.602151 | 92 | 0 | 681 |
| 508 | 252 | 7.704655 | 92 | 0 | 681 |
| 508 | 343 | 8.695652 | 92 | 0 | 681 |
| 508 | 427 | 9.195402 | 92 | 0 | 681 |
| 508 | 511 | 10 | 92 | 0 | 681 |
| 508 | 588 | 10.78652 | 92 | 0 | 681 |
| 509 | 28 | 12.5 | 65 | 1 | 361 |
| 509 | 56 | 27.90698 | 65 | 1 | 361 |
| 509 | 168 | 25.80645 | 65 | 1 | 361 |
| 509 | 280 | 15.65217 | 65 | 1 | 361 |
| 509 | 322 | 9.756098 | 65 | 1 | 361 |
| 510 | 0 | 8.108108 | 76 | 0 | 715 |
| 510 | 28 | 8.411215 | 76 | 0 | 715 |
| 510 | 56 | 8.108108 | 76 | 0 | 715 |
| 510 | 147 | 10.81081 | 76 | 0 | 715 |
| 510 | 203 | 8.347826 | 76 | 0 | 715 |
| 510 | 294 | 12.97297 | 76 | 0 | 715 |
| 510 | 399 | 10.61947 | 76 | 0 | 715 |
| 510 | 523 | 8.421053 | 76 | 0 | 715 |
| 510 | 567 | 8.571429 | 76 | 0 | 715 |
| 511 | 0 | 13.84615 | 88 | 0 | 284 |
| 511 | 21 | 13.58491 | 88 | 0 | 284 |
| 511 | 56 | 13.09091 | 88 | 0 | 284 |
| 511 | 147 | 16.82243 | 88 | 0 | 284 |
| 511 | 238 | 18 | 88 | 0 | 284 |
| 512 | 0 | 4.081633 | 84 | 0 | 74 |
| 512 | 23 | 8.421053 | 84 | 0 | 74 |
| 512 | 69 | 9.902741 | 84 | 0 | 74 |
| 513 | 0 | 2.93578 | 81 | 0 | 737 |
| 513 | 63 | 3.168317 | 81 | 0 | 737 |
| 513 | 168 | 2.836879 | 81 | 0 | 737 |
| 513 | 238 | 4.166667 | 81 | 0 | 737 |
| 514 | 0 | 2.359882 | 59 | 1 | 471 |
| 514 | 95 | 6.185567 | 59 | 1 | 471 |
| 514 | 179 | 5.940594 | 59 | 1 | 471 |
| 514 | 242 | 5.052632 | 59 | 1 | 471 |
| 514 | 333 | 1.41844 | 59 | 1 | 471 |
| 514 | 431 | 6.818182 | 59 | 1 | 471 |

| | | | | | |
|---|---|---|---|---|---|
| 515 | 0 | 24 | 53 | 0 | 856 |
| 515 | 28 | 13.63636 | 53 | 0 | 856 |
| 515 | 91 | 2.158273 | 53 | 0 | 856 |
| 516 | 0 | 3.539823 | 59 | 0 | 1269 |
| 516 | 28 | 3.448276 | 59 | 0 | 1269 |
| 516 | 56 | 3.508772 | 59 | 0 | 1269 |
| 516 | 168 | 3.508772 | 59 | 0 | 1269 |
| 516 | 253 | 3.771044 | 59 | 0 | 1269 |
| 516 | 364 | 3.389831 | 59 | 0 | 1269 |
| 516 | 420 | 3.603604 | 59 | 0 | 1269 |
| 516 | 476 | 3.603604 | 59 | 0 | 1269 |
| 516 | 560 | 3.669725 | 59 | 0 | 1269 |
| 517 | 0 | 1.344538 | 76 | 1 | 240 |
| 517 | 37 | 1.344538 | 76 | 1 | 240 |
| 517 | 72 | 2.941176 | 76 | 1 | 240 |
| 517 | 179 | 9.278351 | 76 | 1 | 240 |
| 517 | 227 | 16.16162 | 76 | 1 | 240 |
| 518 | 0 | 0.47619 | 80 | 0 | 673 |
| 518 | 28 | 0.519481 | 80 | 0 | 673 |
| 518 | 91 | 1.851852 | 80 | 0 | 673 |
| 518 | 147 | 1.509434 | 80 | 0 | 673 |
| 518 | 252 | 1.941748 | 80 | 0 | 673 |
| 518 | 336 | 0.434783 | 80 | 0 | 673 |
| 518 | 420 | 1.212121 | 80 | 0 | 673 |
| 518 | 525 | 1.834862 | 80 | 0 | 673 |
| 518 | 588 | 2.605459 | 80 | 0 | 673 |
| 519 | 0 | 3.678161 | 49 | 1 | 717 |
| 519 | 97 | 1.742919 | 49 | 1 | 717 |
| 520 | 0 | 2.542373 | 65 | 0 | 977 |
| 520 | 28 | 1.923077 | 65 | 0 | 977 |
| 520 | 84 | 2.197802 | 65 | 0 | 977 |
| 520 | 168 | 10.52632 | 65 | 0 | 977 |
| 520 | 252 | 14.25743 | 65 | 0 | 977 |
| 520 | 336 | 11.76471 | 65 | 0 | 977 |
| 520 | 416 | 11.76471 | 65 | 0 | 977 |
| 520 | 497 | 5.714286 | 65 | 0 | 977 |
| 520 | 574 | 5.660377 | 65 | 0 | 977 |
| 521 | 0 | 2.910603 | 45 | 1 | 616 |
| 521 | 26 | 2.5 | 45 | 1 | 616 |

| | | | | | |
|---|---|---|---|---|---|
| 521 | 84 | 2.702703 | 45 | 1 | 616 |
| 521 | 168 | 2.702703 | 45 | 1 | 616 |
| 521 | 259 | 2.542373 | 45 | 1 | 616 |
| 521 | 322 | 2.608696 | 45 | 1 | 616 |
| 521 | 413 | 3.636364 | 45 | 1 | 616 |
| 521 | 469 | 3.636364 | 45 | 1 | 616 |
| 522 | 0 | 2.410676 | 72 | 0 | 451 |
| 522 | 23 | 4.444444 | 72 | 0 | 451 |
| 522 | 87 | 1.960784 | 72 | 0 | 451 |
| 522 | 164 | 5.544554 | 72 | 0 | 451 |
| 522 | 241 | 4.040404 | 72 | 0 | 451 |
| 523 | 0 | 2.884615 | 75 | 0 | 755 |
| 523 | 28 | 3.689065 | 75 | 0 | 755 |
| 523 | 51 | 2.857143 | 75 | 0 | 755 |
| 524 | 0 | 2.991453 | 91 | 0 | 11 |
| 525 | 0 | 0.116234 | 73 | 0 | 582 |
| 525 | 105 | 0.122574 | 73 | 0 | 582 |
| 525 | 189 | 0.11236 | 73 | 0 | 582 |
| 525 | 231 | 0.11932 | 73 | 0 | 582 |
| 525 | 336 | 0.106167 | 73 | 0 | 582 |
| 525 | 371 | 0.111431 | 73 | 0 | 582 |
| 525 | 486 | 0.106167 | 73 | 0 | 582 |
| 526 | 0 | 5.940594 | 58 | 1 | 162 |
| 526 | 28 | 5.309735 | 58 | 1 | 162 |
| 526 | 84 | 5.309735 | 58 | 1 | 162 |
| 527 | 0 | 0.166667 | 42 | 0 | 1016 |
| 527 | 56 | 0.162602 | 42 | 0 | 1016 |
| 527 | 140 | 0.168067 | 42 | 0 | 1016 |
| 527 | 224 | 0.166667 | 42 | 0 | 1016 |
| 527 | 322 | 0.483092 | 42 | 0 | 1016 |
| 527 | 406 | 0.453686 | 42 | 0 | 1016 |
| 527 | 483 | 0.492207 | 42 | 0 | 1016 |
| 528 | 0 | 10 | 52 | 0 | 932 |
| 528 | 28 | 10 | 52 | 0 | 932 |
| 528 | 84 | 9.677419 | 52 | 0 | 932 |
| 528 | 168 | 9.160305 | 52 | 0 | 932 |
| 528 | 252 | 9.160305 | 52 | 0 | 932 |
| 528 | 336 | 9.302326 | 52 | 0 | 932 |
| 528 | 420 | 9.302326 | 52 | 0 | 932 |

| | | | | | |
|---|---|---|---|---|---|
| 528 | 504 | 9.302326 | 52 | 0 | 932 |
| 528 | 588 | 4.477612 | 52 | 0 | 932 |
| 529 | 0 | 4 | 60 | 1 | 85 |
| 530 | 0 | 2.5 | 79 | 0 | 568 |
| 530 | 28 | 0.786627 | 79 | 0 | 568 |
| 530 | 63 | 1.584158 | 79 | 0 | 568 |
| 530 | 154 | 2.105263 | 79 | 0 | 568 |
| 530 | 217 | 2.086957 | 79 | 0 | 568 |
| 530 | 315 | 2.142857 | 79 | 0 | 568 |
| 530 | 413 | 2.702703 | 79 | 0 | 568 |
| 530 | 504 | 5.618729 | 79 | 0 | 568 |
| 530 | 567 | 5.217391 | 79 | 0 | 568 |
| 531 | 0 | 1.5625 | 88 | 0 | 162 |
| 531 | 28 | 1.546392 | 88 | 0 | 162 |
| 531 | 84 | 3.296703 | 88 | 0 | 162 |
| 531 | 128 | 6.930693 | 88 | 0 | 162 |
| 532 | 0 | 2.016807 | 56 | 0 | 1030 |
| 532 | 35 | 1.73913 | 56 | 0 | 1030 |
| 532 | 77 | 1.157503 | 56 | 0 | 1030 |
| 532 | 161 | 0.754581 | 56 | 0 | 1030 |
| 532 | 245 | 0.997151 | 56 | 0 | 1030 |
| 532 | 349 | 0.862069 | 56 | 0 | 1030 |
| 533 | 0 | 5.940594 | 55 | 1 | 331 |
| 533 | 28 | 5.309735 | 55 | 1 | 331 |
| 533 | 182 | 5.217391 | 55 | 1 | 331 |
| 533 | 266 | 6 | 55 | 1 | 331 |
| 534 | 0 | 1.234568 | 78 | 0 | 750 |
| 534 | 77 | 1.293103 | 78 | 0 | 750 |
| 534 | 182 | 1.767305 | 78 | 0 | 750 |
| 534 | 231 | 2.912621 | 78 | 0 | 750 |
| 534 | 287 | 6.122449 | 78 | 0 | 750 |
| 534 | 406 | 1.360544 | 78 | 0 | 750 |
| 534 | 504 | 6.382979 | 78 | 0 | 750 |
| 534 | 560 | 3.007519 | 78 | 0 | 750 |
| 535 | 0 | 11.65049 | 80 | 0 | 750 |
| 535 | 28 | 5.263158 | 80 | 0 | 750 |
| 535 | 63 | 4.571429 | 80 | 0 | 750 |
| 535 | 147 | 12 | 80 | 0 | 750 |
| 535 | 238 | 9.896907 | 80 | 0 | 750 |

| | | | | | |
|---|---|---|---|---|---|
| 535 | 322 | 14.69388 | 80 | 0 | 750 |
| 535 | 427 | 18.94737 | 80 | 0 | 750 |
| 535 | 490 | 14.54545 | 80 | 0 | 750 |
| 535 | 560 | 14.69388 | 80 | 0 | 750 |
| 536 | 0 | 1.754386 | 80 | 0 | 763 |
| 536 | 28 | 2.941176 | 80 | 0 | 763 |
| 536 | 91 | 4.848485 | 80 | 0 | 763 |
| 536 | 182 | 5.940594 | 80 | 0 | 763 |
| 536 | 245 | 4.752475 | 80 | 0 | 763 |
| 536 | 335 | 1.304348 | 80 | 0 | 763 |
| 536 | 420 | 6 | 80 | 0 | 763 |
| 536 | 504 | 2.586207 | 80 | 0 | 763 |
| 536 | 588 | 6.185567 | 80 | 0 | 763 |
| 537 | 0 | 6.066442 | 50 | 0 | 753 |
| 537 | 31 | 5.733136 | 50 | 0 | 753 |
| 537 | 91 | 11.75373 | 50 | 0 | 753 |
| 537 | 178 | 4.267209 | 50 | 0 | 753 |
| 537 | 248 | 4.651163 | 50 | 0 | 753 |
| 537 | 332 | 5.084746 | 50 | 0 | 753 |
| 537 | 388 | 4.651163 | 50 | 0 | 753 |
| 537 | 507 | 3.809524 | 50 | 0 | 753 |
| 537 | 542 | 3.536842 | 50 | 0 | 753 |
| 538 | 0 | 1.880878 | 55 | 0 | 684 |
| 538 | 77 | 0.820513 | 55 | 0 | 684 |
| 538 | 166 | 0.774194 | 55 | 0 | 684 |
| 538 | 252 | 0.984413 | 55 | 0 | 684 |
| 538 | 329 | 0.85531 | 55 | 0 | 684 |
| 538 | 413 | 1.568627 | 55 | 0 | 684 |
| 538 | 487 | 1.444788 | 55 | 0 | 684 |
| 538 | 590 | 8.888889 | 55 | 0 | 684 |
| 539 | 0 | 1.25 | 54 | 0 | 1051 |
| 539 | 21 | 0.832755 | 54 | 0 | 1051 |
| 539 | 77 | 0.892857 | 54 | 0 | 1051 |
| 539 | 161 | 0.738462 | 54 | 0 | 1051 |
| 539 | 252 | 0.414079 | 54 | 0 | 1051 |
| 539 | 329 | 0.401338 | 54 | 0 | 1051 |
| 539 | 413 | 0.930233 | 54 | 0 | 1051 |
| 539 | 499 | 0.840336 | 54 | 0 | 1051 |
| 539 | 583 | 1.223242 | 54 | 0 | 1051 |

| | | | | | |
|---|---|---|---|---|---|
| 540 | 0 | 7.692308 | 83 | 0 | 547 |
| 541 | 0 | 2.752294 | 61 | 1 | 1059 |
| 541 | 28 | 2.830189 | 61 | 1 | 1059 |
| 541 | 84 | 2.803738 | 61 | 1 | 1059 |
| 541 | 175 | 2.654867 | 61 | 1 | 1059 |
| 541 | 273 | 1.886792 | 61 | 1 | 1059 |
| 541 | 350 | 3.773585 | 61 | 1 | 1059 |
| 541 | 427 | 11.11111 | 61 | 1 | 1059 |
| 541 | 490 | 10.16949 | 61 | 1 | 1059 |
| 542 | 0 | 2.678571 | 55 | 0 | 589 |
| 542 | 77 | 2.884615 | 55 | 0 | 589 |
| 542 | 168 | 3.738318 | 55 | 0 | 589 |
| 542 | 252 | 3.883495 | 55 | 0 | 589 |
| 542 | 336 | 2.807018 | 55 | 0 | 589 |
| 542 | 420 | 3.571429 | 55 | 0 | 589 |
| 542 | 504 | 3.539823 | 55 | 0 | 589 |
| 542 | 588 | 3.636364 | 55 | 0 | 589 |
| 543 | 0 | 4.528302 | 80 | 0 | 476 |
| 543 | 105 | 4.615385 | 80 | 0 | 476 |
| 543 | 163 | 6.213018 | 80 | 0 | 476 |
| 543 | 247 | 4.670559 | 80 | 0 | 476 |
| 543 | 338 | 4.363636 | 80 | 0 | 476 |
| 543 | 429 | 6.666667 | 80 | 0 | 476 |
| 544 | 0 | 12.30769 | 76 | 0 | 1019 |
| 544 | 35 | 14.87603 | 76 | 0 | 1019 |
| 544 | 106 | 10.46512 | 76 | 0 | 1019 |
| 544 | 175 | 15.51724 | 76 | 0 | 1019 |
| 544 | 241 | 11.6129 | 76 | 0 | 1019 |
| 544 | 350 | 6.61157 | 76 | 0 | 1019 |
| 544 | 413 | 12.41379 | 76 | 0 | 1019 |
| 544 | 490 | 10.34483 | 76 | 0 | 1019 |
| 544 | 567 | 11.80328 | 76 | 0 | 1019 |
| 545 | 0 | 0.531679 | 75 | 0 | 618 |
| 545 | 77 | 0.545083 | 75 | 0 | 618 |
| 545 | 140 | 0.438596 | 75 | 0 | 618 |
| 545 | 266 | 0.420168 | 75 | 0 | 618 |
| 545 | 329 | 0.442478 | 75 | 0 | 618 |
| 545 | 385 | 0.434783 | 75 | 0 | 618 |
| 545 | 511 | 6.530612 | 75 | 0 | 618 |

| | | | | | |
|---|---|---|---|---|---|
| 545 | 560 | 6.349206 | 75 | 0 | 618 |
| 546 | 0 | 0.892857 | 89 | 0 | 255 |
| 546 | 28 | 0.943396 | 89 | 0 | 255 |
| 546 | 84 | 0.961538 | 89 | 0 | 255 |
| 546 | 171 | 1.818182 | 89 | 0 | 255 |
| 547 | 0 | 2.201835 | 73 | 0 | 1215 |
| 547 | 35 | 2.201835 | 73 | 0 | 1215 |
| 547 | 70 | 0.733945 | 73 | 0 | 1215 |
| 547 | 175 | 2.830189 | 73 | 0 | 1215 |
| 547 | 244 | 4.948454 | 73 | 0 | 1215 |
| 547 | 420 | 4.705882 | 73 | 0 | 1215 |
| 547 | 623 | 13.58491 | 73 | 0 | 1215 |
| 548 | 0 | 24.70588 | 69 | 0 | 904 |
| 548 | 28 | 16.94118 | 69 | 0 | 904 |
| 548 | 63 | 16.74419 | 69 | 0 | 904 |
| 548 | 168 | 17.56098 | 69 | 0 | 904 |
| 548 | 231 | 13.84615 | 69 | 0 | 904 |
| 549 | 0 | 1.2 | 83 | 0 | 821 |
| 549 | 42 | 1.2 | 83 | 0 | 821 |
| 549 | 105 | 4.166667 | 83 | 0 | 821 |
| 549 | 175 | 4.660194 | 83 | 0 | 821 |
| 549 | 252 | 6.382979 | 83 | 0 | 821 |
| 549 | 343 | 6.382979 | 83 | 0 | 821 |
| 549 | 434 | 5.16129 | 83 | 0 | 821 |
| 549 | 504 | 12.63158 | 83 | 0 | 821 |
| 549 | 595 | 13.18681 | 83 | 0 | 821 |
| 550 | 0 | 12.85714 | 81 | 0 | 1092 |
| 550 | 35 | 6.837607 | 81 | 0 | 1092 |
| 550 | 98 | 8.490566 | 81 | 0 | 1092 |
| 550 | 154 | 2.782609 | 81 | 0 | 1092 |
| 550 | 259 | 9.994051 | 81 | 0 | 1092 |
| 550 | 364 | 6.969027 | 81 | 0 | 1092 |
| 550 | 434 | 18.66667 | 81 | 0 | 1092 |
| 550 | 496 | 8.643457 | 81 | 0 | 1092 |
| 551 | 0 | 4.421053 | 83 | 0 | 948 |
| 551 | 25 | 4.528302 | 83 | 0 | 948 |
| 551 | 88 | 6.122449 | 83 | 0 | 948 |
| 551 | 179 | 4.948454 | 83 | 0 | 948 |
| 551 | 235 | 5.940594 | 83 | 0 | 948 |

| | | | | | |
|---|---|---|---|---|---|
| 552 | 0 | 3.061224 | 36 | 1 | 141 |
| 552 | 19 | 10.27523 | 36 | 1 | 141 |
| 552 | 49 | 4.571429 | 36 | 1 | 141 |
| 553 | 0 | 5.263158 | 80 | 1 | 357 |
| 553 | 28 | 2.542373 | 80 | 1 | 357 |
| 553 | 91 | 2.264151 | 80 | 1 | 357 |
| 553 | 161 | 10.32258 | 80 | 1 | 357 |
| 553 | 238 | 8.4 | 80 | 1 | 357 |
| 553 | 329 | 2.648172 | 80 | 1 | 357 |
| 554 | 0 | 1.481481 | 86 | 0 | 603 |
| 554 | 28 | 1.029601 | 86 | 0 | 603 |
| 554 | 112 | 2.222222 | 86 | 0 | 603 |
| 554 | 189 | 3.738318 | 86 | 0 | 603 |
| 554 | 273 | 1.886792 | 86 | 0 | 603 |
| 554 | 329 | 2.105263 | 86 | 0 | 603 |
| 554 | 413 | 1.923077 | 86 | 0 | 603 |
| 554 | 483 | 1.25 | 86 | 0 | 603 |
| 554 | 602 | 1.367521 | 86 | 0 | 603 |
| 555 | 0 | 5.825243 | 79 | 0 | 214 |
| 555 | 28 | 5.825243 | 79 | 0 | 214 |
| 555 | 84 | 23.52941 | 79 | 0 | 214 |
| 555 | 182 | 13.86139 | 79 | 0 | 214 |
| 556 | 0 | 1.973684 | 85 | 0 | 1317 |
| 556 | 24 | 1.660079 | 85 | 0 | 1317 |
| 556 | 70 | 4.615385 | 85 | 0 | 1317 |
| 556 | 147 | 3.773585 | 85 | 0 | 1317 |
| 556 | 231 | 1.801802 | 85 | 0 | 1317 |
| 557 | 0 | 4.918033 | 69 | 0 | 1142 |
| 557 | 28 | 5.042017 | 69 | 0 | 1142 |
| 557 | 84 | 4.137931 | 69 | 0 | 1142 |
| 557 | 175 | 0.884956 | 69 | 0 | 1142 |
| 557 | 259 | 4.958678 | 69 | 0 | 1142 |
| 557 | 343 | 5.405405 | 69 | 0 | 1142 |
| 557 | 427 | 5.309735 | 69 | 0 | 1142 |
| 557 | 511 | 4.528302 | 69 | 0 | 1142 |
| 557 | 574 | 5.555556 | 69 | 0 | 1142 |
| 558 | 0 | 6.060606 | 56 | 1 | 328 |
| 558 | 28 | 5.274725 | 56 | 1 | 328 |
| 558 | 63 | 12.06897 | 56 | 1 | 328 |

| | | | | | |
|---|---|---|---|---|---|
| 558 | 158 | 13.18681 | 56 | 1 | 328 |
| 558 | 238 | 13.48315 | 56 | 1 | 328 |
| 558 | 301 | 7.920792 | 56 | 1 | 328 |
| 559 | 0 | 10.61947 | 49 | 0 | 1002 |
| 559 | 28 | 8.495575 | 49 | 0 | 1002 |
| 559 | 63 | 8.495575 | 49 | 0 | 1002 |
| 559 | 168 | 8.421053 | 49 | 0 | 1002 |
| 559 | 238 | 8.571429 | 49 | 0 | 1002 |
| 559 | 308 | 8.275862 | 49 | 0 | 1002 |
| 559 | 413 | 6.779661 | 49 | 0 | 1002 |
| 559 | 490 | 8.067227 | 49 | 0 | 1002 |
| 559 | 595 | 7.933884 | 49 | 0 | 1002 |
| 560 | 0 | 1.886792 | 45 | 1 | 343 |
| 560 | 31 | 0.682261 | 45 | 1 | 343 |
| 560 | 66 | 0.670498 | 45 | 1 | 343 |
| 560 | 136 | 0.379867 | 45 | 1 | 343 |
| 560 | 241 | 5.147059 | 45 | 1 | 343 |
| 560 | 336 | 7.317073 | 45 | 1 | 343 |
| 561 | 0 | 2.608696 | 79 | 1 | 398 |
| 561 | 28 | 2.586207 | 79 | 1 | 398 |
| 561 | 84 | 2.678571 | 79 | 1 | 398 |
| 561 | 168 | 1.626016 | 79 | 1 | 398 |
| 561 | 257 | 2.45614 | 79 | 1 | 398 |
| 561 | 322 | 2.222222 | 79 | 1 | 398 |
| 562 | 0 | 1.084011 | 65 | 0 | 596 |
| 562 | 21 | 1.149425 | 65 | 0 | 596 |
| 562 | 42 | 1.73913 | 65 | 0 | 596 |
| 562 | 126 | 3.061224 | 65 | 0 | 596 |
| 562 | 217 | 3.809524 | 65 | 0 | 596 |
| 562 | 301 | 3.571429 | 65 | 0 | 596 |
| 562 | 378 | 1.930813 | 65 | 0 | 596 |
| 562 | 497 | 3.603604 | 65 | 0 | 596 |
| 562 | 595 | 2.631579 | 65 | 0 | 596 |
| 563 | 0 | 4.067797 | 72 | 1 | 194 |
| 563 | 63 | 4.724409 | 72 | 1 | 194 |
| 563 | 147 | 5.946903 | 72 | 1 | 194 |
| 564 | 0 | 16.32653 | 56 | 0 | 403 |
| 564 | 21 | 23.07692 | 56 | 0 | 403 |
| 564 | 80 | 25.35211 | 56 | 0 | 403 |

| | | | | | |
|---|---|---|---|---|---|
| 564 | 140 | 18.18182 | 56 | 0 | 403 |
| 564 | 231 | 21.91781 | 56 | 0 | 403 |
| 564 | 315 | 12.12121 | 56 | 0 | 403 |
| 565 | 0 | 8.419646 | 74 | 0 | 292 |
| 565 | 32 | 7.682927 | 74 | 0 | 292 |
| 565 | 82 | 19.31034 | 74 | 0 | 292 |
| 565 | 167 | 18.8764 | 74 | 0 | 292 |
| 566 | 0 | 2.051282 | 73 | 0 | 804 |
| 566 | 35 | 2.162162 | 73 | 0 | 804 |
| 566 | 70 | 2.330097 | 73 | 0 | 804 |
| 566 | 175 | 4.660194 | 73 | 0 | 804 |
| 566 | 252 | 7.920792 | 73 | 0 | 804 |
| 566 | 336 | 3.571429 | 73 | 0 | 804 |
| 566 | 427 | 6.408545 | 73 | 0 | 804 |
| 566 | 525 | 6.857143 | 73 | 0 | 804 |
| 566 | 623 | 6.593407 | 73 | 0 | 804 |
| 567 | 0 | 1.327434 | 72 | 1 | 611 |
| 567 | 28 | 1.339286 | 72 | 1 | 611 |
| 567 | 91 | 3.636364 | 72 | 1 | 611 |
| 567 | 147 | 1.441441 | 72 | 1 | 611 |
| 567 | 231 | 1.834862 | 72 | 1 | 611 |
| 567 | 315 | 0.900901 | 72 | 1 | 611 |
| 567 | 399 | 13.04348 | 72 | 1 | 611 |
| 567 | 483 | 4.324324 | 72 | 1 | 611 |
| 567 | 574 | 5.504587 | 72 | 1 | 611 |
| 568 | 0 | 1.574803 | 71 | 0 | 1513 |
| 568 | 28 | 2.040816 | 71 | 0 | 1513 |
| 568 | 168 | 6.469003 | 71 | 0 | 1513 |
| 568 | 273 | 5.042017 | 71 | 0 | 1513 |
| 568 | 336 | 5.660377 | 71 | 0 | 1513 |
| 568 | 392 | 5.263158 | 71 | 0 | 1513 |
| 568 | 504 | 6.233766 | 71 | 0 | 1513 |
| 568 | 602 | 5.91133 | 71 | 0 | 1513 |
| 569 | 0 | 1.550388 | 64 | 0 | 28 |
| 569 | 27 | 1.2 | 64 | 0 | 28 |
| 570 | 0 | 0.590551 | 85 | 0 | 554 |
| 570 | 82 | 2.201835 | 85 | 0 | 554 |
| 570 | 161 | 1.075269 | 85 | 0 | 554 |
| 571 | 0 | 10.60606 | 73 | 0 | 751 |

| | | | | | |
|---|---|---|---|---|---|
| 571 | 100 | 9.795918 | 73 | 0 | 751 |
| 571 | 177 | 16.07656 | 73 | 0 | 751 |
| 571 | 275 | 14.84536 | 73 | 0 | 751 |
| 571 | 331 | 18.29401 | 73 | 0 | 751 |
| 571 | 443 | 18.94737 | 73 | 0 | 751 |
| 571 | 499 | 14.4 | 73 | 0 | 751 |
| 571 | 611 | 17.30769 | 73 | 0 | 751 |
| 572 | 0 | 2.479339 | 78 | 0 | 1107 |
| 572 | 21 | 2.564103 | 78 | 0 | 1107 |
| 572 | 98 | 1.869159 | 78 | 0 | 1107 |
| 572 | 182 | 3.603604 | 78 | 0 | 1107 |
| 572 | 238 | 4.485981 | 78 | 0 | 1107 |
| 572 | 336 | 8.971963 | 78 | 0 | 1107 |
| 572 | 440 | 8.903021 | 78 | 0 | 1107 |
| 572 | 630 | 1.680672 | 78 | 0 | 1107 |
| 573 | 0 | 6.25 | 61 | 1 | 67 |
| 573 | 28 | 1.388889 | 61 | 1 | 67 |
| 574 | 0 | 2.727273 | 56 | 0 | 759 |
| 574 | 39 | 2.702703 | 56 | 0 | 759 |
| 574 | 67 | 2.941176 | 56 | 0 | 759 |
| 575 | 0 | 3.145478 | 58 | 0 | 225 |
| 575 | 49 | 3.463203 | 58 | 0 | 225 |
| 575 | 182 | 1.73913 | 58 | 0 | 225 |
| 575 | 224 | 3.571429 | 58 | 0 | 225 |
| 576 | 0 | 0.376648 | 71 | 0 | 1031 |
| 576 | 28 | 0.376648 | 71 | 0 | 1031 |
| 576 | 91 | 0.37037 | 71 | 0 | 1031 |
| 577 | 0 | 2.479339 | 69 | 0 | 1268 |
| 577 | 28 | 1.219512 | 69 | 0 | 1268 |
| 577 | 84 | 1.139601 | 69 | 0 | 1268 |
| 577 | 168 | 1.15942 | 69 | 0 | 1268 |
| 577 | 252 | 1.129944 | 69 | 0 | 1268 |
| 577 | 336 | 1.139601 | 69 | 0 | 1268 |
| 577 | 420 | 1.182557 | 69 | 0 | 1268 |
| 577 | 497 | 0.984615 | 69 | 0 | 1268 |
| 577 | 588 | 1.017165 | 69 | 0 | 1268 |
| 578 | 0 | 2.857143 | 81 | 0 | 560 |
| 578 | 21 | 5.825243 | 81 | 0 | 560 |
| 578 | 112 | 5.454545 | 81 | 0 | 560 |

| | | | | | |
|---|---|---|---|---|---|
| 578 | 259 | 5.128205 | 81 | 0 | 560 |
| 578 | 350 | 5.217391 | 81 | 0 | 560 |
| 578 | 448 | 4.173913 | 81 | 0 | 560 |
| 578 | 559 | 7 | 81 | 0 | 560 |
| 579 | 0 | 13.125 | 87 | 0 | 834 |
| 579 | 29 | 3.188259 | 87 | 0 | 834 |
| 579 | 84 | 2.786378 | 87 | 0 | 834 |
| 579 | 182 | 29.64706 | 87 | 0 | 834 |
| 579 | 245 | 14.4 | 87 | 0 | 834 |
| 579 | 308 | 13.33333 | 87 | 0 | 834 |
| 579 | 427 | 2.962963 | 87 | 0 | 834 |
| 579 | 497 | 3.305785 | 87 | 0 | 834 |
| 579 | 560 | 11.52 | 87 | 0 | 834 |
| 580 | 0 | 13.33333 | 80 | 0 | 140 |
| 580 | 27 | 24.37137 | 80 | 0 | 140 |
| 580 | 78 | 11.53846 | 80 | 0 | 140 |
| 581 | 0 | 5.504587 | 85 | 0 | 1003 |
| 581 | 28 | 5.405405 | 85 | 0 | 1003 |
| 581 | 84 | 8.648649 | 85 | 0 | 1003 |
| 581 | 175 | 10.52632 | 85 | 0 | 1003 |
| 581 | 259 | 10.08403 | 85 | 0 | 1003 |
| 581 | 343 | 8.727273 | 85 | 0 | 1003 |
| 581 | 420 | 10 | 85 | 0 | 1003 |
| 581 | 518 | 11.32075 | 85 | 0 | 1003 |
| 581 | 587 | 11.32075 | 85 | 0 | 1003 |
| 582 | 0 | 10.66667 | 80 | 0 | 820 |
| 582 | 35 | 12.63158 | 80 | 0 | 820 |
| 582 | 77 | 11.76471 | 80 | 0 | 820 |
| 582 | 175 | 7.76699 | 80 | 0 | 820 |
| 582 | 259 | 1.709402 | 80 | 0 | 820 |
| 582 | 343 | 2.285714 | 80 | 0 | 820 |
| 582 | 427 | 1.587302 | 80 | 0 | 820 |
| 582 | 476 | 1.869159 | 80 | 0 | 820 |
| 582 | 609 | 1.851852 | 80 | 0 | 820 |
| 583 | 0 | 3.305785 | 69 | 0 | 393 |
| 583 | 21 | 2.259887 | 69 | 0 | 393 |
| 583 | 70 | 2.168022 | 69 | 0 | 393 |
| 584 | 0 | 6.593407 | 74 | 0 | 648 |
| 584 | 74 | 6.818182 | 74 | 0 | 648 |

| | | | | | |
|---|---|---|---|---|---|
| 584 | 195 | 5.365698 | 74 | 0 | 648 |
| 584 | 244 | 6.722689 | 74 | 0 | 648 |
| 584 | 503 | 4.752475 | 74 | 0 | 648 |
| 584 | 608 | 4.948454 | 74 | 0 | 648 |
| 585 | 0 | 2.330097 | 76 | 1 | 201 |
| 585 | 70 | 6.315789 | 76 | 1 | 201 |
| 585 | 161 | 4.705882 | 76 | 1 | 201 |
| 586 | 0 | 5.357143 | 80 | 1 | 429 |
| 586 | 28 | 7.207207 | 80 | 1 | 429 |
| 586 | 86 | 5.097087 | 80 | 1 | 429 |
| 586 | 168 | 4.660194 | 80 | 1 | 429 |
| 586 | 252 | 11.29032 | 80 | 1 | 429 |
| 586 | 338 | 10.71429 | 80 | 1 | 429 |
| 586 | 423 | 17.5 | 80 | 1 | 429 |
| 587 | 0 | 2.222222 | 51 | 1 | 187 |
| 587 | 28 | 2.086957 | 51 | 1 | 187 |
| 587 | 91 | 2.521008 | 51 | 1 | 187 |
| 587 | 154 | 3.478261 | 51 | 1 | 187 |
| 588 | 0 | 3.883495 | 66 | 1 | 291 |
| 588 | 28 | 8 | 66 | 1 | 291 |
| 588 | 84 | 12.12121 | 66 | 1 | 291 |
| 588 | 168 | 11.00917 | 66 | 1 | 291 |
| 588 | 247 | 22.20753 | 66 | 1 | 291 |
| 589 | 0 | 16 | 72 | 0 | 801 |
| 589 | 98 | 5.925926 | 72 | 0 | 801 |
| 589 | 161 | 18.75 | 72 | 0 | 801 |
| 589 | 240 | 9.056604 | 72 | 0 | 801 |
| 589 | 343 | 8.495575 | 72 | 0 | 801 |
| 589 | 401 | 2.123894 | 72 | 0 | 801 |
| 589 | 492 | 3.703704 | 72 | 0 | 801 |
| 589 | 569 | 5.825243 | 72 | 0 | 801 |
| 590 | 0 | 5.128205 | 65 | 1 | 180 |
| 590 | 28 | 5.172414 | 65 | 1 | 180 |
| 590 | 56 | 5.128205 | 65 | 1 | 180 |
| 591 | 0 | 2.105263 | 82 | 1 | 592 |
| 591 | 35 | 0.862069 | 82 | 1 | 592 |
| 591 | 122 | 0.97527 | 82 | 1 | 592 |
| 591 | 161 | 0.606805 | 82 | 1 | 592 |
| 591 | 217 | 0.629921 | 82 | 1 | 592 |

| | | | | | |
|---|---|---|---|---|---|
| 591 | 373 | 3.106509 | 82 | 1 | 592 |
| 591 | 434 | 0.489939 | 82 | 1 | 592 |
| 591 | 532 | 0.576132 | 82 | 1 | 592 |
| 592 | 0 | 2.564103 | 71 | 1 | 554 |
| 592 | 28 | 2.479339 | 71 | 1 | 554 |
| 592 | 84 | 2.542373 | 71 | 1 | 554 |
| 592 | 168 | 2.654867 | 71 | 1 | 554 |
| 592 | 252 | 2.564103 | 71 | 1 | 554 |
| 592 | 336 | 2.702703 | 71 | 1 | 554 |
| 592 | 420 | 1.801802 | 71 | 1 | 554 |
| 592 | 490 | 10.61947 | 71 | 1 | 554 |
| 593 | 0 | 1.787995 | 81 | 0 | 946 |
| 593 | 27 | 1.73913 | 81 | 0 | 946 |
| 593 | 90 | 2.693603 | 81 | 0 | 946 |
| 593 | 167 | 1.904762 | 81 | 0 | 946 |
| 593 | 223 | 1.834862 | 81 | 0 | 946 |
| 593 | 335 | 1.874791 | 81 | 0 | 946 |
| 593 | 454 | 1.632653 | 81 | 0 | 946 |
| 593 | 522 | 3.240741 | 81 | 0 | 946 |
| 593 | 573 | 6.185567 | 81 | 0 | 946 |
| 594 | 0 | 5.825243 | 65 | 1 | 335 |
| 594 | 70 | 5.263158 | 65 | 1 | 335 |
| 594 | 192 | 5.08167 | 65 | 1 | 335 |
| 594 | 252 | 11.83932 | 65 | 1 | 335 |
| 594 | 334 | 25.26316 | 65 | 1 | 335 |
| 595 | 0 | 3.539823 | 54 | 1 | 672 |
| 595 | 28 | 1.709402 | 54 | 1 | 672 |
| 595 | 84 | 3.428571 | 54 | 1 | 672 |
| 595 | 168 | 11.65049 | 54 | 1 | 672 |
| 595 | 252 | 11.88119 | 54 | 1 | 672 |
| 595 | 336 | 14.69388 | 54 | 1 | 672 |
| 595 | 420 | 12.76596 | 54 | 1 | 672 |
| 595 | 511 | 13.7931 | 54 | 1 | 672 |
| 595 | 588 | 15.65217 | 54 | 1 | 672 |
| 596 | 0 | 2.727273 | 76 | 1 | 484 |
| 596 | 28 | 2.242991 | 76 | 1 | 484 |
| 596 | 91 | 3.508772 | 76 | 1 | 484 |
| 596 | 147 | 2.93578 | 76 | 1 | 484 |
| 596 | 238 | 1.904762 | 76 | 1 | 484 |

| | | | | | |
|---|---|---|---|---|---|
| 596 | 321 | 4 | 76 | 1 | 484 |
| 596 | 406 | 4.633205 | 76 | 1 | 484 |
| 597 | 0 | 0.293938 | 50 | 0 | 1002 |
| 597 | 28 | 0.29914 | 50 | 0 | 1002 |
| 597 | 56 | 0.307298 | 50 | 0 | 1002 |
| 597 | 174 | 0.29914 | 50 | 0 | 1002 |
| 597 | 224 | 0.312989 | 50 | 0 | 1002 |
| 597 | 322 | 0.29914 | 50 | 0 | 1002 |
| 597 | 406 | 2.307692 | 50 | 0 | 1002 |
| 597 | 476 | 3.773585 | 50 | 0 | 1002 |
| 597 | 539 | 3.571429 | 50 | 0 | 1002 |
| 598 | 0 | 0.78125 | 75 | 0 | 794 |
| 598 | 42 | 3.921569 | 75 | 0 | 794 |
| 598 | 84 | 3.846154 | 75 | 0 | 794 |
| 598 | 210 | 7.407407 | 75 | 0 | 794 |
| 598 | 448 | 4.444444 | 75 | 0 | 794 |
| 599 | 0 | 3.883495 | 81 | 0 | 1284 |
| 599 | 35 | 5.825243 | 81 | 0 | 1284 |
| 599 | 126 | 2.830189 | 81 | 0 | 1284 |
| 599 | 210 | 2 | 81 | 0 | 1284 |
| 599 | 315 | 1.206897 | 81 | 0 | 1284 |
| 599 | 378 | 1.48857 | 81 | 0 | 1284 |
| 599 | 490 | 1.428571 | 81 | 0 | 1284 |
| 600 | 0 | 1.981132 | 81 | 0 | 644 |
| 600 | 40 | 11.93182 | 81 | 0 | 644 |
| 600 | 126 | 2.474227 | 81 | 0 | 644 |
| 600 | 189 | 2.777778 | 81 | 0 | 644 |
| 600 | 252 | 7.058824 | 81 | 0 | 644 |
| 600 | 371 | 4.324324 | 81 | 0 | 644 |
| 600 | 427 | 4.193361 | 81 | 0 | 644 |
| 600 | 490 | 3.81558 | 81 | 0 | 644 |
| 600 | 602 | 8.910891 | 81 | 0 | 644 |
| 601 | 0 | 4.494382 | 84 | 0 | 327 |
| 601 | 25 | 1.980198 | 84 | 0 | 327 |
| 601 | 56 | 3.846154 | 84 | 0 | 327 |
| 601 | 140 | 8.602151 | 84 | 0 | 327 |
| 601 | 231 | 1.834862 | 84 | 0 | 327 |
| 602 | 0 | 10.66667 | 85 | 0 | 919 |
| 602 | 35 | 10.78652 | 85 | 0 | 919 |

| | | | | | |
|---|---|---|---|---|---|
| 602 | 70 | 19.35484 | 85 | 0 | 919 |
| 602 | 154 | 18.5567 | 85 | 0 | 919 |
| 602 | 221 | 7.017544 | 85 | 0 | 919 |
| 602 | 350 | 9.756098 | 85 | 0 | 919 |
| 602 | 420 | 6.060606 | 85 | 0 | 919 |
| 602 | 504 | 6.61157 | 85 | 0 | 919 |
| 602 | 546 | 6.779661 | 85 | 0 | 919 |
| 603 | 0 | 0.583495 | 62 | 0 | 868 |
| 603 | 28 | 0.57377 | 62 | 0 | 868 |
| 603 | 91 | 1.576577 | 62 | 0 | 868 |
| 604 | 0 | 0.076453 | 61 | 0 | 639 |
| 604 | 105 | 0.074405 | 61 | 0 | 639 |
| 604 | 175 | 0.075075 | 61 | 0 | 639 |
| 604 | 266 | 0.073099 | 61 | 0 | 639 |
| 604 | 322 | 0.075075 | 61 | 0 | 639 |
| 604 | 504 | 0.080906 | 61 | 0 | 639 |
| 605 | 0 | 1.923077 | 57 | 1 | 385 |
| 605 | 56 | 2.333333 | 57 | 1 | 385 |
| 605 | 154 | 1.824699 | 57 | 1 | 385 |
| 605 | 252 | 2.083333 | 57 | 1 | 385 |
| 605 | 321 | 2.745098 | 57 | 1 | 385 |
| 606 | 0 | 12.2449 | 88 | 0 | 722 |
| 606 | 28 | 5.660377 | 88 | 0 | 722 |
| 606 | 84 | 6.679035 | 88 | 0 | 722 |
| 606 | 161 | 22.5 | 88 | 0 | 722 |
| 606 | 252 | 11.30298 | 88 | 0 | 722 |
| 606 | 336 | 12.76596 | 88 | 0 | 722 |
| 606 | 413 | 9.52381 | 88 | 0 | 722 |
| 606 | 504 | 13.69565 | 88 | 0 | 722 |
| 606 | 588 | 13.47594 | 88 | 0 | 722 |
| 607 | 0 | 0.701754 | 74 | 1 | 392 |
| 607 | 70 | 0.792079 | 74 | 1 | 392 |
| 607 | 154 | 0.821114 | 74 | 1 | 392 |
| 607 | 245 | 3.232323 | 74 | 1 | 392 |
| 608 | 0 | 3.389831 | 85 | 1 | 350 |
| 608 | 28 | 5.084746 | 85 | 1 | 350 |
| 608 | 77 | 5.454545 | 85 | 1 | 350 |
| 608 | 160 | 6.521739 | 85 | 1 | 350 |
| 608 | 246 | 17.02128 | 85 | 1 | 350 |

| | | | | | |
|---|---|---|---|---|---|
| 608 | 331 | 6.499033 | 85 | 1 | 350 |
| 609 | 0 | 2.857143 | 89 | 1 | 221 |
| 609 | 28 | 4.301075 | 89 | 1 | 221 |
| 609 | 77 | 4.210526 | 89 | 1 | 221 |
| 609 | 133 | 1.581921 | 89 | 1 | 221 |
| 610 | 0 | 5.263158 | 61 | 0 | 1023 |
| 610 | 28 | 5.357143 | 61 | 0 | 1023 |
| 610 | 56 | 1.317647 | 61 | 0 | 1023 |
| 610 | 158 | 6.451613 | 61 | 0 | 1023 |
| 610 | 287 | 4.794521 | 61 | 0 | 1023 |
| 611 | 0 | 2.830189 | 76 | 1 | 185 |
| 611 | 28 | 2.222222 | 76 | 1 | 185 |
| 611 | 98 | 4.571429 | 76 | 1 | 185 |
| 611 | 182 | 7.373272 | 76 | 1 | 185 |
| 612 | 0 | 1.294498 | 74 | 0 | 848 |
| 612 | 28 | 1.179941 | 74 | 0 | 848 |
| 612 | 63 | 6.666667 | 74 | 0 | 848 |
| 612 | 161 | 3.669725 | 74 | 0 | 848 |
| 612 | 245 | 3.373946 | 74 | 0 | 848 |
| 612 | 329 | 3.116883 | 74 | 0 | 848 |
| 612 | 455 | 8.737864 | 74 | 0 | 848 |
| 612 | 511 | 12.52174 | 74 | 0 | 848 |
| 612 | 546 | 4.033613 | 74 | 0 | 848 |
| 613 | 0 | 7.017544 | 66 | 1 | 63 |
| 613 | 21 | 4.958678 | 66 | 1 | 63 |
| 613 | 49 | 5.607477 | 66 | 1 | 63 |
| 614 | 0 | 3.26087 | 77 | 1 | 100 |
| 614 | 28 | 3.678161 | 77 | 1 | 100 |
| 615 | 0 | 3.763441 | 65 | 0 | 877 |
| 615 | 119 | 4.958678 | 65 | 0 | 877 |
| 615 | 175 | 4.449153 | 65 | 0 | 877 |
| 615 | 231 | 5.263158 | 65 | 0 | 877 |
| 615 | 343 | 11.00917 | 65 | 0 | 877 |
| 615 | 461 | 7.058824 | 65 | 0 | 877 |
| 615 | 525 | 10.02985 | 65 | 0 | 877 |
| 615 | 574 | 4.477612 | 65 | 0 | 877 |
| 616 | 0 | 1.95122 | 55 | 0 | 1099 |
| 616 | 35 | 2.5 | 55 | 0 | 1099 |
| 616 | 98 | 0.649351 | 55 | 0 | 1099 |

| | | | | | |
|---|---|---|---|---|---|
| 616 | 161 | 1.075269 | 55 | 0 | 1099 |
| 616 | 244 | 2.181818 | 55 | 0 | 1099 |
| 616 | 342 | 2.631579 | 55 | 0 | 1099 |
| 616 | 433 | 2.441151 | 55 | 0 | 1099 |
| 616 | 489 | 2.702703 | 55 | 0 | 1099 |
| 616 | 578 | 11.32075 | 55 | 0 | 1099 |
| 617 | 0 | 2.654867 | 75 | 0 | 890 |
| 617 | 77 | 0.733945 | 75 | 0 | 890 |
| 617 | 182 | 0.740741 | 75 | 0 | 890 |
| 617 | 273 | 0.862689 | 75 | 0 | 890 |
| 617 | 367 | 0.867589 | 75 | 0 | 890 |
| 617 | 413 | 0.685323 | 75 | 0 | 890 |
| 617 | 532 | 0.713012 | 75 | 0 | 890 |
| 617 | 560 | 4.123711 | 75 | 0 | 890 |
| 618 | 0 | 1.196411 | 49 | 0 | 681 |
| 618 | 35 | 1.27186 | 49 | 0 | 681 |
| 618 | 94 | 2.564103 | 49 | 0 | 681 |
| 618 | 175 | 1.801802 | 49 | 0 | 681 |
| 618 | 287 | 5.128205 | 49 | 0 | 681 |
| 618 | 346 | 5.555556 | 49 | 0 | 681 |
| 618 | 430 | 5.172414 | 49 | 0 | 681 |
| 618 | 514 | 5.607477 | 49 | 0 | 681 |
| 618 | 598 | 5.504587 | 49 | 0 | 681 |
| 619 | 0 | 2.201835 | 86 | 0 | 592 |
| 619 | 35 | 2.752294 | 86 | 0 | 592 |
| 619 | 91 | 2.752294 | 86 | 0 | 592 |
| 619 | 144 | 4.210526 | 86 | 0 | 592 |
| 619 | 252 | 6.177606 | 86 | 0 | 592 |
| 619 | 343 | 10.90909 | 86 | 0 | 592 |
| 619 | 419 | 9.183673 | 86 | 0 | 592 |
| 619 | 490 | 13.21101 | 86 | 0 | 592 |
| 620 | 0 | 4.897959 | 78 | 1 | 250 |
| 620 | 35 | 5.052632 | 78 | 1 | 250 |
| 620 | 70 | 13.04348 | 78 | 1 | 250 |
| 620 | 126 | 11.65049 | 78 | 1 | 250 |
| 620 | 211 | 14.47028 | 78 | 1 | 250 |
| 621 | 0 | 5.333333 | 68 | 1 | 194 |
| 621 | 63 | 9.411765 | 68 | 1 | 194 |
| 621 | 147 | 4.772727 | 68 | 1 | 194 |

| | | | | | |
|---|---|---|---|---|---|
| 622 | 0 | 2.588997 | 68 | 0 | 1248 |
| 622 | 42 | 5.040504 | 68 | 0 | 1248 |
| 622 | 91 | 2.380952 | 68 | 0 | 1248 |
| 622 | 140 | 1.923077 | 68 | 0 | 1248 |
| 622 | 252 | 2.240896 | 68 | 0 | 1248 |
| 622 | 350 | 3.703704 | 68 | 0 | 1248 |
| 622 | 413 | 1.980198 | 68 | 0 | 1248 |
| 622 | 525 | 2.061856 | 68 | 0 | 1248 |
| 622 | 581 | 3.409091 | 68 | 0 | 1248 |
| 623 | 0 | 1.553398 | 57 | 0 | 540 |
| 623 | 28 | 5.442177 | 57 | 0 | 540 |
| 623 | 77 | 7.407407 | 57 | 0 | 540 |
| 623 | 161 | 8.333333 | 57 | 0 | 540 |
| 623 | 245 | 16.06426 | 57 | 0 | 540 |
| 623 | 329 | 8.333333 | 57 | 0 | 540 |
| 623 | 413 | 3.278689 | 57 | 0 | 540 |
| 623 | 497 | 5.882353 | 57 | 0 | 540 |
| 624 | 0 | 1.869159 | 88 | 0 | 853 |
| 624 | 70 | 1.941748 | 88 | 0 | 853 |
| 624 | 154 | 1.139601 | 88 | 0 | 853 |
| 624 | 205 | 1.161103 | 88 | 0 | 853 |
| 625 | 0 | 5.318139 | 76 | 0 | 770 |
| 625 | 27 | 4.761905 | 76 | 0 | 770 |
| 625 | 84 | 5.217391 | 76 | 0 | 770 |
| 625 | 196 | 5 | 76 | 0 | 770 |
| 625 | 259 | 5.309735 | 76 | 0 | 770 |
| 625 | 315 | 5 | 76 | 0 | 770 |
| 625 | 427 | 5.363985 | 76 | 0 | 770 |
| 625 | 511 | 4.761905 | 76 | 0 | 770 |
| 625 | 567 | 4.83871 | 76 | 0 | 770 |
| 626 | 0 | 1.339286 | 59 | 0 | 645 |
| 626 | 21 | 0.909091 | 59 | 0 | 645 |
| 626 | 63 | 0.816327 | 59 | 0 | 645 |
| 626 | 161 | 0.970874 | 59 | 0 | 645 |
| 626 | 252 | 2.330097 | 59 | 0 | 645 |
| 626 | 329 | 1.442308 | 59 | 0 | 645 |
| 626 | 406 | 1.176471 | 59 | 0 | 645 |
| 626 | 490 | 0.961538 | 59 | 0 | 645 |
| 626 | 581 | 1.269841 | 59 | 0 | 645 |

| | | | | | |
|---|---|---|---|---|---|
| 627 | 0 | 2.830189 | 75 | 0 | 952 |
| 627 | 56 | 2.970297 | 75 | 0 | 952 |
| 627 | 168 | 2.912621 | 75 | 0 | 952 |
| 627 | 224 | 1.834862 | 75 | 0 | 952 |
| 627 | 364 | 2.752294 | 75 | 0 | 952 |
| 627 | 392 | 1.282051 | 75 | 0 | 952 |
| 627 | 532 | 2.752294 | 75 | 0 | 952 |
| 628 | 0 | 1.785714 | 70 | 0 | 848 |
| 628 | 98 | 5.405405 | 70 | 0 | 848 |
| 628 | 161 | 6.956522 | 70 | 0 | 848 |
| 628 | 273 | 3.204272 | 70 | 0 | 848 |
| 628 | 357 | 7.619048 | 70 | 0 | 848 |
| 628 | 427 | 3.847916 | 70 | 0 | 848 |
| 628 | 490 | 8.888889 | 70 | 0 | 848 |
| 628 | 574 | 6.015038 | 70 | 0 | 848 |
| 629 | 0 | 3.773585 | 48 | 1 | 138 |
| 629 | 28 | 4 | 48 | 1 | 138 |
| 629 | 84 | 11.88119 | 48 | 1 | 138 |
| 630 | 0 | 2.727273 | 74 | 0 | 793 |
| 630 | 28 | 1.724138 | 74 | 0 | 793 |
| 630 | 70 | 2.631579 | 74 | 0 | 793 |
| 630 | 155 | 5.405405 | 74 | 0 | 793 |
| 630 | 238 | 3.571429 | 74 | 0 | 793 |
| 630 | 337 | 8.910891 | 74 | 0 | 793 |
| 630 | 393 | 8.259587 | 74 | 0 | 793 |
| 630 | 491 | 10 | 74 | 0 | 793 |
| 630 | 582 | 8.135593 | 74 | 0 | 793 |
| 631 | 0 | 5.128205 | 69 | 0 | 659 |
| 631 | 28 | 5.454545 | 69 | 0 | 659 |
| 631 | 91 | 5.309735 | 69 | 0 | 659 |
| 631 | 175 | 1.517067 | 69 | 0 | 659 |
| 631 | 252 | 2.702703 | 69 | 0 | 659 |
| 631 | 350 | 1.149425 | 69 | 0 | 659 |
| 631 | 406 | 2.586207 | 69 | 0 | 659 |
| 631 | 546 | 2.752294 | 69 | 0 | 659 |
| 631 | 602 | 2.678571 | 69 | 0 | 659 |
| 632 | 0 | 1.190476 | 51 | 0 | 1062 |
| 632 | 28 | 2.777778 | 51 | 0 | 1062 |
| 632 | 98 | 1.923077 | 51 | 0 | 1062 |

| | | | | | |
|---|---|---|---|---|---|
| 632 | 182 | 2.040816 | 51 | 0 | 1062 |
| 632 | 273 | 3.703704 | 51 | 0 | 1062 |
| 632 | 357 | 4.210526 | 51 | 0 | 1062 |
| 632 | 427 | 11.00917 | 51 | 0 | 1062 |
| 632 | 483 | 4 | 51 | 0 | 1062 |
| 632 | 602 | 12.30769 | 51 | 0 | 1062 |
| 633 | 0 | 4.123711 | 73 | 0 | 1171 |
| 633 | 28 | 3.571429 | 73 | 0 | 1171 |
| 633 | 77 | 3.53461 | 73 | 0 | 1171 |
| 633 | 175 | 3.686636 | 73 | 0 | 1171 |
| 633 | 259 | 13.18681 | 73 | 0 | 1171 |
| 633 | 322 | 6.896552 | 73 | 0 | 1171 |
| 633 | 441 | 3 | 73 | 0 | 1171 |
| 633 | 497 | 3.125 | 73 | 0 | 1171 |
| 633 | 609 | 3.030303 | 73 | 0 | 1171 |
| 634 | 0 | 12.12121 | 84 | 1 | 162 |
| 634 | 35 | 10.34483 | 84 | 1 | 162 |
| 634 | 90 | 7.017544 | 84 | 1 | 162 |
| 635 | 0 | 1.724138 | 75 | 0 | 766 |
| 635 | 42 | 1.801802 | 75 | 0 | 766 |
| 635 | 84 | 1.465201 | 75 | 0 | 766 |
| 635 | 196 | 1.169591 | 75 | 0 | 766 |
| 635 | 259 | 1.179941 | 75 | 0 | 766 |
| 636 | 0 | 1.481481 | 62 | 1 | 246 |
| 636 | 101 | 1.842105 | 62 | 1 | 246 |
| 636 | 157 | 3.333333 | 62 | 1 | 246 |
| 636 | 245 | 2.571166 | 62 | 1 | 246 |
| 637 | 0 | 3.389831 | 68 | 0 | 843 |
| 637 | 42 | 5.504587 | 68 | 0 | 843 |
| 637 | 184 | 4.918033 | 68 | 0 | 843 |
| 637 | 247 | 4.761905 | 68 | 0 | 843 |
| 637 | 329 | 5.454545 | 68 | 0 | 843 |
| 638 | 0 | 4.705882 | 81 | 0 | 1037 |
| 638 | 35 | 3.603604 | 81 | 0 | 1037 |
| 638 | 77 | 4.363636 | 81 | 0 | 1037 |
| 638 | 175 | 5.106383 | 81 | 0 | 1037 |
| 638 | 364 | 1.382488 | 81 | 0 | 1037 |
| 638 | 434 | 4.660194 | 81 | 0 | 1037 |
| 638 | 539 | 9.105691 | 81 | 0 | 1037 |

| | | | | | |
|---|---|---|---|---|---|
| 638 | 574 | 2.965578 | 81 | 0 | 1037 |
| 639 | 0 | 3.030303 | 68 | 0 | 85 |
| 639 | 28 | 1.6 | 68 | 0 | 85 |
| 639 | 84 | 4.166667 | 68 | 0 | 85 |
| 640 | 0 | 4.878049 | 86 | 0 | 703 |
| 640 | 28 | 4.375 | 86 | 0 | 703 |
| 640 | 86 | 4.8 | 86 | 0 | 703 |
| 640 | 170 | 5.217391 | 86 | 0 | 703 |
| 640 | 254 | 5.042017 | 86 | 0 | 703 |
| 640 | 338 | 4.878049 | 86 | 0 | 703 |
| 640 | 422 | 4.83871 | 86 | 0 | 703 |
| 640 | 478 | 4.878049 | 86 | 0 | 703 |
| 640 | 562 | 5.084746 | 86 | 0 | 703 |
| 641 | 0 | 2.752294 | 59 | 1 | 212 |
| 641 | 111 | 2.941176 | 59 | 1 | 212 |
| 641 | 175 | 4.485981 | 59 | 1 | 212 |
| 641 | 211 | 4.296675 | 59 | 1 | 212 |
| 642 | 0 | 6.060606 | 64 | 1 | 904 |
| 642 | 28 | 5.555556 | 64 | 1 | 904 |
| 642 | 84 | 5.607477 | 64 | 1 | 904 |
| 642 | 168 | 11.21495 | 64 | 1 | 904 |
| 642 | 252 | 10.34483 | 64 | 1 | 904 |
| 642 | 339 | 15.14423 | 64 | 1 | 904 |
| 642 | 399 | 12.20339 | 64 | 1 | 904 |
| 642 | 490 | 19.78022 | 64 | 1 | 904 |
| 643 | 28 | 1.290323 | 56 | 0 | 1009 |
| 643 | 63 | 0.550964 | 56 | 0 | 1009 |
| 643 | 147 | 1.37457 | 56 | 0 | 1009 |
| 643 | 210 | 1.785714 | 56 | 0 | 1009 |
| 643 | 287 | 0.990099 | 56 | 0 | 1009 |
| 643 | 371 | 3 | 56 | 0 | 1009 |
| 643 | 483 | 6.593407 | 56 | 0 | 1009 |
| 643 | 539 | 5.607477 | 56 | 0 | 1009 |
| 644 | 0 | 3.571429 | 70 | 0 | 443 |
| 644 | 28 | 3.636364 | 70 | 0 | 443 |
| 644 | 77 | 2.857143 | 70 | 0 | 443 |
| 644 | 182 | 5.940594 | 70 | 0 | 443 |
| 644 | 261 | 11.76471 | 70 | 0 | 443 |
| 644 | 336 | 9.411765 | 70 | 0 | 443 |

| | | | | | |
|---|---|---|---|---|---|
| 645 | 0 | 3.568394 | 88 | 0 | 1097 |
| 645 | 44 | 2.588997 | 88 | 0 | 1097 |
| 645 | 107 | 0.400811 | 88 | 0 | 1097 |
| 645 | 172 | 0.404819 | 88 | 0 | 1097 |
| 645 | 235 | 0.389249 | 88 | 0 | 1097 |
| 645 | 368 | 0.413081 | 88 | 0 | 1097 |
| 645 | 438 | 0.408908 | 88 | 0 | 1097 |
| 645 | 522 | 6.469003 | 88 | 0 | 1097 |
| 645 | 571 | 7.373272 | 88 | 0 | 1097 |
| 646 | 0 | 10.71429 | 82 | 0 | 489 |
| 646 | 28 | 10.25641 | 82 | 0 | 489 |
| 646 | 76 | 2.116402 | 82 | 0 | 489 |
| 646 | 175 | 11.88119 | 82 | 0 | 489 |
| 646 | 259 | 5.714286 | 82 | 0 | 489 |
| 646 | 329 | 3.333333 | 82 | 0 | 489 |
| 646 | 418 | 10.81081 | 82 | 0 | 489 |
| 647 | 28 | 0.75188 | 77 | 0 | 1352 |
| 647 | 84 | 0.877193 | 77 | 0 | 1352 |
| 647 | 168 | 0.840336 | 77 | 0 | 1352 |
| 647 | 252 | 0.819672 | 77 | 0 | 1352 |
| 647 | 336 | 1.310401 | 77 | 0 | 1352 |
| 647 | 413 | 1.089176 | 77 | 0 | 1352 |
| 647 | 504 | 1.232666 | 77 | 0 | 1352 |
| 647 | 581 | 2.020202 | 77 | 0 | 1352 |
| 648 | 0 | 5.357143 | 77 | 1 | 86 |
| 648 | 35 | 5.405405 | 77 | 1 | 86 |
| 648 | 84 | 5.309735 | 77 | 1 | 86 |
| 649 | 0 | 3.361345 | 78 | 0 | 1328 |
| 649 | 28 | 1.652893 | 78 | 0 | 1328 |
| 649 | 84 | 1.724138 | 78 | 0 | 1328 |
| 649 | 168 | 3.703704 | 78 | 0 | 1328 |
| 649 | 252 | 11.65049 | 78 | 0 | 1328 |
| 649 | 336 | 5.309735 | 78 | 0 | 1328 |
| 649 | 420 | 6.315789 | 78 | 0 | 1328 |
| 649 | 504 | 6.185567 | 78 | 0 | 1328 |
| 649 | 588 | 6.521739 | 78 | 0 | 1328 |
| 650 | 0 | 6.25 | 70 | 1 | 525 |
| 650 | 56 | 12 | 70 | 1 | 525 |
| 650 | 168 | 12.79513 | 70 | 1 | 525 |

| | | | | | |
|---|---|---|---|---|---|
| 650 | 280 | 10.66667 | 70 | 1 | 525 |
| 650 | 336 | 12.07764 | 70 | 1 | 525 |
| 650 | 441 | 13.33333 | 70 | 1 | 525 |
| 650 | 497 | 10.61947 | 70 | 1 | 525 |
| 651 | 0 | 2.151639 | 54 | 0 | 593 |
| 651 | 32 | 2.479339 | 54 | 0 | 593 |
| 651 | 116 | 2.479339 | 54 | 0 | 593 |
| 651 | 172 | 2.439024 | 54 | 0 | 593 |
| 651 | 263 | 2.564103 | 54 | 0 | 593 |
| 651 | 354 | 3.539823 | 54 | 0 | 593 |
| 651 | 431 | 2.678571 | 54 | 0 | 593 |
| 651 | 494 | 2.678571 | 54 | 0 | 593 |
| 651 | 578 | 2.752294 | 54 | 0 | 593 |
| 652 | 0 | 1.558442 | 79 | 1 | 413 |
| 652 | 28 | 1.440576 | 79 | 1 | 413 |
| 652 | 84 | 1.025641 | 79 | 1 | 413 |
| 652 | 189 | 2.307692 | 79 | 1 | 413 |
| 652 | 259 | 1.111111 | 79 | 1 | 413 |
| 652 | 329 | 1.286765 | 79 | 1 | 413 |
| 652 | 412 | 6.25 | 79 | 1 | 413 |
| 653 | 0 | 1.626016 | 78 | 1 | 614 |
| 653 | 32 | 1.639344 | 78 | 1 | 614 |
| 653 | 88 | 4.363636 | 78 | 1 | 614 |
| 653 | 144 | 5.172414 | 78 | 1 | 614 |
| 653 | 256 | 3.157895 | 78 | 1 | 614 |
| 653 | 312 | 3.157895 | 78 | 1 | 614 |
| 653 | 403 | 1.960784 | 78 | 1 | 614 |
| 653 | 515 | 5.274725 | 78 | 1 | 614 |
| 653 | 550 | 4.897959 | 78 | 1 | 614 |
| 654 | 0 | 5.555556 | 91 | 0 | 442 |
| 654 | 77 | 1.587302 | 91 | 0 | 442 |
| 654 | 175 | 1.477833 | 91 | 0 | 442 |
| 654 | 238 | 1.344538 | 91 | 0 | 442 |
| 654 | 343 | 1.454545 | 91 | 0 | 442 |
| 654 | 406 | 2.424242 | 91 | 0 | 442 |
| 655 | 0 | 5.607477 | 74 | 0 | 1261 |
| 655 | 28 | 5.769231 | 74 | 0 | 1261 |
| 655 | 84 | 8.080808 | 74 | 0 | 1261 |
| 655 | 168 | 7.407407 | 74 | 0 | 1261 |

| | | | | | |
|---|---|---|---|---|---|
| 655 | 224 | 10.94819 | 74 | 0 | 1261 |
| 655 | 308 | 18 | 74 | 0 | 1261 |
| 655 | 420 | 18.18182 | 74 | 0 | 1261 |
| 655 | 475 | 18.18182 | 74 | 0 | 1261 |
| 655 | 588 | 17.20724 | 74 | 0 | 1261 |
| 656 | 0 | 2.162162 | 54 | 1 | 229 |
| 656 | 28 | 3.921569 | 54 | 1 | 229 |
| 656 | 91 | 5.052632 | 54 | 1 | 229 |
| 656 | 147 | 9.953786 | 54 | 1 | 229 |
| 656 | 217 | 8.718215 | 54 | 1 | 229 |
| 657 | 0 | 5.405405 | 57 | 1 | 104 |
| 657 | 28 | 12.37113 | 57 | 1 | 104 |
| 657 | 84 | 11.21495 | 57 | 1 | 104 |
| 658 | 0 | 2.752294 | 62 | 1 | 89 |
| 658 | 28 | 2.912621 | 62 | 1 | 89 |
| 659 | 0 | 1.818182 | 66 | 0 | 642 |
| 659 | 42 | 1.73913 | 66 | 0 | 642 |
| 659 | 84 | 1.769912 | 66 | 0 | 642 |
| 659 | 168 | 1.834862 | 66 | 0 | 642 |
| 659 | 252 | 1.680672 | 66 | 0 | 642 |
| 660 | 0 | 2.884615 | 61 | 1 | 387 |
| 660 | 35 | 2.991453 | 61 | 1 | 387 |
| 660 | 160 | 3.296703 | 61 | 1 | 387 |
| 660 | 244 | 6.741573 | 61 | 1 | 387 |
| 660 | 335 | 11.88119 | 61 | 1 | 387 |
| 660 | 376 | 12.90323 | 61 | 1 | 387 |
| 661 | 21 | 9.677419 | 74 | 0 | 1359 |
| 661 | 84 | 6.976744 | 74 | 0 | 1359 |
| 661 | 161 | 4.761905 | 74 | 0 | 1359 |
| 661 | 245 | 5.263158 | 74 | 0 | 1359 |
| 661 | 399 | 5.128205 | 74 | 0 | 1359 |
| 661 | 483 | 4.724409 | 74 | 0 | 1359 |
| 661 | 595 | 5 | 74 | 0 | 1359 |
| 662 | 0 | 4.761905 | 73 | 0 | 834 |
| 662 | 72 | 13.71429 | 73 | 0 | 834 |
| 662 | 161 | 14.36307 | 73 | 0 | 834 |
| 662 | 273 | 4.285714 | 73 | 0 | 834 |
| 662 | 357 | 19.56522 | 73 | 0 | 834 |
| 662 | 413 | 7.142857 | 73 | 0 | 834 |

| | | | | | |
|---|---|---|---|---|---|
| 662 | 525 | 11.23746 | 73 | 0 | 834 |
| 662 | 588 | 5.084746 | 73 | 0 | 834 |
| 663 | 0 | 4.580153 | 57 | 0 | 603 |
| 663 | 28 | 4.6875 | 57 | 0 | 603 |
| 663 | 84 | 4.324324 | 57 | 0 | 603 |
| 663 | 175 | 5.454545 | 57 | 0 | 603 |
| 663 | 231 | 5.357143 | 57 | 0 | 603 |
| 663 | 343 | 3.934426 | 57 | 0 | 603 |
| 663 | 434 | 5.309735 | 57 | 0 | 603 |
| 663 | 518 | 5.309735 | 57 | 0 | 603 |
| 663 | 602 | 5.454545 | 57 | 0 | 603 |
| 664 | 0 | 6.122449 | 78 | 0 | 729 |
| 664 | 28 | 9.896907 | 78 | 0 | 729 |
| 664 | 63 | 7.692308 | 78 | 0 | 729 |
| 664 | 135 | 12 | 78 | 0 | 729 |
| 664 | 261 | 10.28466 | 78 | 0 | 729 |
| 664 | 324 | 11.21495 | 78 | 0 | 729 |
| 664 | 415 | 12.30769 | 78 | 0 | 729 |
| 665 | 0 | 20.93023 | 82 | 0 | 55 |
| 665 | 27 | 17.47573 | 82 | 0 | 55 |
| 666 | 0 | 2.474227 | 81 | 0 | 710 |
| 666 | 28 | 2.941176 | 81 | 0 | 710 |
| 666 | 91 | 2.666667 | 81 | 0 | 710 |
| 666 | 161 | 2.474227 | 81 | 0 | 710 |
| 666 | 252 | 3 | 81 | 0 | 710 |
| 666 | 336 | 2.210526 | 81 | 0 | 710 |
| 666 | 406 | 2.526316 | 81 | 0 | 710 |
| 666 | 511 | 2.474227 | 81 | 0 | 710 |
| 666 | 609 | 5.940594 | 81 | 0 | 710 |
| 667 | 0 | 3.265306 | 79 | 0 | 1202 |
| 667 | 35 | 3.137255 | 79 | 0 | 1202 |
| 667 | 70 | 3.333333 | 79 | 0 | 1202 |
| 667 | 182 | 3.883495 | 79 | 0 | 1202 |
| 667 | 238 | 2.857143 | 79 | 0 | 1202 |
| 667 | 350 | 2.970297 | 79 | 0 | 1202 |
| 667 | 413 | 3.191489 | 79 | 0 | 1202 |
| 667 | 518 | 3.265306 | 79 | 0 | 1202 |
| 667 | 609 | 1.963993 | 79 | 0 | 1202 |
| 668 | 0 | 2.222222 | 63 | 0 | 939 |

| | | | | | |
|---|---|---|---|---|---|
| 668 | 35 | 3.030303 | 63 | 0 | 939 |
| 668 | 105 | 3.830369 | 63 | 0 | 939 |
| 668 | 133 | 5.825243 | 63 | 0 | 939 |
| 668 | 273 | 5.769231 | 63 | 0 | 939 |
| 668 | 336 | 4.571429 | 63 | 0 | 939 |
| 668 | 406 | 6.122449 | 63 | 0 | 939 |
| 668 | 504 | 8.823529 | 63 | 0 | 939 |
| 668 | 560 | 8.411215 | 63 | 0 | 939 |
| 669 | 0 | 7.692308 | 64 | 1 | 991 |
| 669 | 84 | 4.705882 | 64 | 1 | 991 |
| 669 | 182 | 5.825243 | 64 | 1 | 991 |
| 669 | 245 | 4.485981 | 64 | 1 | 991 |
| 669 | 315 | 2.941176 | 64 | 1 | 991 |
| 669 | 406 | 4.705882 | 64 | 1 | 991 |
| 669 | 476 | 4.347826 | 64 | 1 | 991 |
| 669 | 560 | 8.988764 | 64 | 1 | 991 |
| 670 | 0 | 5.769231 | 51 | 1 | 46 |
| 670 | 28 | 6 | 51 | 1 | 46 |
| 671 | 0 | 2.521008 | 52 | 0 | 653 |
| 671 | 28 | 2.608696 | 52 | 0 | 653 |
| 671 | 98 | 1.886792 | 52 | 0 | 653 |
| 671 | 189 | 2.162162 | 52 | 0 | 653 |
| 671 | 252 | 2.857143 | 52 | 0 | 653 |
| 671 | 336 | 7.619048 | 52 | 0 | 653 |
| 671 | 427 | 10.71429 | 52 | 0 | 653 |
| 671 | 511 | 10.61947 | 52 | 0 | 653 |
| 672 | 0 | 6.095791 | 75 | 0 | 1101 |
| 672 | 26 | 5.172414 | 75 | 0 | 1101 |
| 672 | 82 | 5.045045 | 75 | 0 | 1101 |
| 672 | 168 | 5.660377 | 75 | 0 | 1101 |
| 672 | 266 | 3.478261 | 75 | 0 | 1101 |
| 672 | 329 | 4.6875 | 75 | 0 | 1101 |
| 672 | 427 | 4.878049 | 75 | 0 | 1101 |
| 672 | 511 | 4.724409 | 75 | 0 | 1101 |
| 672 | 567 | 6.25 | 75 | 0 | 1101 |
| 673 | 0 | 2.752294 | 65 | 0 | 725 |
| 673 | 84 | 5.504587 | 65 | 0 | 725 |
| 673 | 147 | 4.485981 | 65 | 0 | 725 |
| 673 | 210 | 5 | 65 | 0 | 725 |

| | | | | | |
|---|---|---|---|---|---|
| 673 | 322 | 2.807018 | 65 | 0 | 725 |
| 673 | 392 | 1.511063 | 65 | 0 | 725 |
| 673 | 499 | 4.615385 | 65 | 0 | 725 |
| 673 | 569 | 3.934426 | 65 | 0 | 725 |
| 674 | 0 | 5.084746 | 69 | 0 | 746 |
| 674 | 28 | 4.671858 | 69 | 0 | 746 |
| 674 | 94 | 4.485981 | 69 | 0 | 746 |
| 674 | 210 | 4.6875 | 69 | 0 | 746 |
| 674 | 343 | 8.502024 | 69 | 0 | 746 |
| 674 | 430 | 7.430341 | 69 | 0 | 746 |
| 674 | 549 | 7.13073 | 69 | 0 | 746 |
| 674 | 626 | 7.13073 | 69 | 0 | 746 |
| 675 | 0 | 2.586207 | 58 | 0 | 817 |
| 675 | 28 | 2.5 | 58 | 0 | 817 |
| 675 | 84 | 2.564103 | 58 | 0 | 817 |
| 675 | 182 | 2.181818 | 58 | 0 | 817 |
| 675 | 329 | 0.584795 | 58 | 0 | 817 |
| 675 | 413 | 2.061856 | 58 | 0 | 817 |
| 675 | 497 | 3.669725 | 58 | 0 | 817 |
| 676 | 0 | 0.297767 | 67 | 0 | 1093 |
| 676 | 35 | 0.314713 | 67 | 0 | 1093 |
| 676 | 112 | 0.314713 | 67 | 0 | 1093 |
| 676 | 182 | 2.086957 | 67 | 0 | 1093 |
| 676 | 252 | 4.528302 | 67 | 0 | 1093 |
| 677 | 0 | 4.8 | 62 | 1 | 229 |
| 677 | 28 | 4.918033 | 62 | 1 | 229 |
| 677 | 84 | 5.504587 | 62 | 1 | 229 |
| 677 | 173 | 7.142857 | 62 | 1 | 229 |
| 678 | 0 | 2.439024 | 57 | 0 | 1464 |
| 678 | 28 | 1.181102 | 57 | 0 | 1464 |
| 678 | 84 | 0.856793 | 57 | 0 | 1464 |
| 678 | 170 | 1.906708 | 57 | 0 | 1464 |
| 678 | 268 | 1.973221 | 57 | 0 | 1464 |
| 678 | 338 | 4.347826 | 57 | 0 | 1464 |
| 678 | 422 | 6.593407 | 57 | 0 | 1464 |
| 678 | 506 | 6.315789 | 57 | 0 | 1464 |
| 678 | 590 | 6.122449 | 57 | 0 | 1464 |
| 679 | 0 | 2.884615 | 83 | 0 | 876 |
| 679 | 84 | 5.405405 | 83 | 0 | 876 |

| | | | | | |
|---|---|---|---|---|---|
| 680 | 0 | 2.830189 | 87 | 0 | 64 |
| 680 | 28 | 5.504587 | 87 | 0 | 64 |
| 681 | 0 | 4.375 | 41 | 1 | 159 |
| 681 | 32 | 5.357143 | 41 | 1 | 159 |
| 681 | 83 | 5.217391 | 41 | 1 | 159 |
| 682 | 0 | 12.63158 | 72 | 0 | 895 |
| 682 | 28 | 11.32075 | 72 | 0 | 895 |
| 683 | 0 | 1.544402 | 61 | 1 | 576 |
| 683 | 29 | 1.517067 | 61 | 1 | 576 |
| 683 | 84 | 3.478261 | 61 | 1 | 576 |
| 683 | 168 | 5.825243 | 61 | 1 | 576 |
| 684 | 0 | 2.608696 | 76 | 0 | 1103 |
| 684 | 28 | 2.678571 | 76 | 0 | 1103 |
| 684 | 85 | 2.727273 | 76 | 0 | 1103 |
| 684 | 169 | 2.830189 | 76 | 0 | 1103 |
| 684 | 253 | 11.47149 | 76 | 0 | 1103 |
| 684 | 337 | 11.42857 | 76 | 0 | 1103 |
| 684 | 421 | 11.00917 | 76 | 0 | 1103 |
| 684 | 505 | 10.34483 | 76 | 0 | 1103 |
| 684 | 588 | 11.41692 | 76 | 0 | 1103 |
| 685 | 0 | 13.7931 | 79 | 0 | 589 |
| 685 | 27 | 14.73684 | 79 | 0 | 589 |
| 685 | 84 | 14.45783 | 79 | 0 | 589 |
| 685 | 168 | 22.78481 | 79 | 0 | 589 |
| 685 | 287 | 16.9697 | 79 | 0 | 589 |
| 685 | 343 | 25.26316 | 79 | 0 | 589 |
| 685 | 427 | 29.62963 | 79 | 0 | 589 |
| 685 | 497 | 40.38462 | 79 | 0 | 589 |
| 685 | 588 | 48.64865 | 79 | 0 | 589 |
| 686 | 0 | 2.689076 | 77 | 1 | 955 |
| 686 | 35 | 1.724138 | 77 | 1 | 955 |
| 686 | 91 | 2.457757 | 77 | 1 | 955 |
| 686 | 140 | 2.406015 | 77 | 1 | 955 |
| 686 | 238 | 3.234501 | 77 | 1 | 955 |
| 686 | 336 | 4.8 | 77 | 1 | 955 |
| 686 | 413 | 3.852327 | 77 | 1 | 955 |
| 686 | 504 | 4.210526 | 77 | 1 | 955 |
| 686 | 588 | 8.510638 | 77 | 1 | 955 |
| 687 | 0 | 3.738318 | 76 | 1 | 240 |

| | | | | | |
|---|---|---|---|---|---|
| 687 | 28 | 2.515723 | 76 | 1 | 240 |
| 687 | 70 | 3.669725 | 76 | 1 | 240 |
| 687 | 154 | 12.2449 | 76 | 1 | 240 |
| 688 | 0 | 2.521008 | 74 | 0 | 977 |
| 688 | 28 | 2.970297 | 74 | 0 | 977 |
| 688 | 112 | 2.727273 | 74 | 0 | 977 |
| 688 | 175 | 2.264151 | 74 | 0 | 977 |
| 688 | 276 | 2.181818 | 74 | 0 | 977 |
| 688 | 339 | 6.25 | 74 | 0 | 977 |
| 688 | 451 | 5.833333 | 74 | 0 | 977 |
| 688 | 511 | 10.94819 | 74 | 0 | 977 |
| 689 | 0 | 6.481481 | 89 | 0 | 348 |
| 689 | 88 | 5.714286 | 89 | 0 | 348 |
| 689 | 144 | 4.615385 | 89 | 0 | 348 |
| 689 | 235 | 5.660377 | 89 | 0 | 348 |
| 689 | 319 | 5 | 89 | 0 | 348 |
| 690 | 0 | 2.909091 | 77 | 1 | 951 |
| 690 | 28 | 0.52597 | 77 | 1 | 951 |
| 690 | 77 | 0.569801 | 77 | 1 | 951 |
| 690 | 161 | 0.808081 | 77 | 1 | 951 |
| 690 | 224 | 0.740741 | 77 | 1 | 951 |
| 690 | 287 | 0.823045 | 77 | 1 | 951 |
| 690 | 399 | 1.178451 | 77 | 1 | 951 |
| 690 | 504 | 0.846561 | 77 | 1 | 951 |
| 690 | 567 | 0.830737 | 77 | 1 | 951 |
| 691 | 0 | 2.970297 | 66 | 0 | 834 |
| 691 | 28 | 5.825243 | 66 | 0 | 834 |
| 691 | 82 | 5.825243 | 66 | 0 | 834 |
| 691 | 154 | 4.991087 | 66 | 0 | 834 |
| 691 | 287 | 5.172414 | 66 | 0 | 834 |
| 691 | 357 | 4.571429 | 66 | 0 | 834 |
| 691 | 413 | 4.444444 | 66 | 0 | 834 |
| 691 | 525 | 7.476636 | 66 | 0 | 834 |
| 691 | 602 | 3.931204 | 66 | 0 | 834 |
| 692 | 0 | 2.201835 | 69 | 0 | 1187 |
| 692 | 35 | 1.022644 | 69 | 0 | 1187 |
| 692 | 73 | 1.022644 | 69 | 0 | 1187 |
| 692 | 150 | 2.285714 | 69 | 0 | 1187 |
| 692 | 224 | 1.454042 | 69 | 0 | 1187 |

| | | | | | |
|---|---|---|---|---|---|
| 692 | 308 | 3.713528 | 69 | 0 | 1187 |
| 692 | 379 | 3.988604 | 69 | 0 | 1187 |
| 692 | 463 | 2.916667 | 69 | 0 | 1187 |
| 692 | 560 | 2.162162 | 69 | 0 | 1187 |
| 693 | 0 | 2.592593 | 77 | 0 | 814 |
| 693 | 84 | 5.410628 | 77 | 0 | 814 |
| 693 | 178 | 0.795756 | 77 | 0 | 814 |
| 693 | 252 | 0.769231 | 77 | 0 | 814 |
| 693 | 325 | 3.966942 | 77 | 0 | 814 |
| 693 | 415 | 0.629921 | 77 | 0 | 814 |
| 693 | 514 | 0.754717 | 77 | 0 | 814 |
| 693 | 611 | 2.564103 | 77 | 0 | 814 |
| 694 | 0 | 2.295082 | 81 | 0 | 899 |
| 694 | 30 | 2.564103 | 81 | 0 | 899 |
| 694 | 86 | 2.479339 | 81 | 0 | 899 |
| 694 | 199 | 1.207938 | 81 | 0 | 899 |
| 694 | 261 | 2.542373 | 81 | 0 | 899 |
| 694 | 317 | 1.574803 | 81 | 0 | 899 |
| 694 | 436 | 1.355932 | 81 | 0 | 899 |
| 694 | 492 | 1.73913 | 81 | 0 | 899 |
| 694 | 576 | 1.652893 | 81 | 0 | 899 |
| 695 | 0 | 4.95283 | 69 | 0 | 754 |
| 695 | 31 | 3.296703 | 69 | 0 | 754 |
| 695 | 88 | 6.818182 | 69 | 0 | 754 |
| 695 | 159 | 6.859943 | 69 | 0 | 754 |
| 696 | 0 | 5.940594 | 76 | 0 | 286 |
| 696 | 28 | 5.607477 | 76 | 0 | 286 |
| 696 | 84 | 11.53846 | 76 | 0 | 286 |
| 696 | 168 | 10.90909 | 76 | 0 | 286 |
| 696 | 259 | 5.084746 | 76 | 0 | 286 |
| 697 | 0 | 2.727273 | 74 | 0 | 711 |
| 697 | 28 | 2.752294 | 74 | 0 | 711 |
| 697 | 91 | 2.556299 | 74 | 0 | 711 |
| 697 | 157 | 2.242991 | 74 | 0 | 711 |
| 697 | 255 | 3.298969 | 74 | 0 | 711 |
| 697 | 346 | 3.960396 | 74 | 0 | 711 |
| 697 | 409 | 3.232323 | 74 | 0 | 711 |
| 697 | 507 | 5.052632 | 74 | 0 | 711 |
| 697 | 570 | 6.521739 | 74 | 0 | 711 |

| | | | | | |
|---|---|---|---|---|---|
| 698 | 0 | 0.895522 | 68 | 1 | 408 |
| 698 | 98 | 2.608696 | 68 | 1 | 408 |
| 698 | 161 | 1.834862 | 68 | 1 | 408 |
| 698 | 245 | 2.330097 | 68 | 1 | 408 |
| 698 | 308 | 2.307692 | 68 | 1 | 408 |
| 698 | 399 | 12.12121 | 68 | 1 | 408 |
| 699 | 0 | 10.08403 | 83 | 0 | 575 |
| 699 | 29 | 7.004378 | 83 | 0 | 575 |
| 699 | 70 | 5.217391 | 83 | 0 | 575 |
| 699 | 161 | 4.407713 | 83 | 0 | 575 |
| 699 | 259 | 6.05042 | 83 | 0 | 575 |
| 699 | 343 | 11.88119 | 83 | 0 | 575 |
| 699 | 413 | 13.09091 | 83 | 0 | 575 |
| 699 | 490 | 4.878049 | 83 | 0 | 575 |
| 699 | 574 | 13.18681 | 83 | 0 | 575 |
| 700 | 0 | 5.714286 | 73 | 1 | 150 |
| 700 | 28 | 12.37113 | 73 | 1 | 150 |
| 700 | 91 | 16.7916 | 73 | 1 | 150 |
| 701 | 0 | 2.022756 | 66 | 0 | 668 |
| 701 | 49 | 2.005013 | 66 | 0 | 668 |
| 701 | 165 | 8.038278 | 66 | 0 | 668 |
| 702 | 0 | 2.051282 | 77 | 0 | 820 |
| 702 | 35 | 2.884615 | 77 | 0 | 820 |
| 702 | 98 | 2 | 77 | 0 | 820 |
| 702 | 154 | 2.016807 | 77 | 0 | 820 |
| 702 | 245 | 2.564103 | 77 | 0 | 820 |
| 702 | 336 | 0.407747 | 77 | 0 | 820 |
| 702 | 399 | 1.257862 | 77 | 0 | 820 |
| 702 | 518 | 1.456311 | 77 | 0 | 820 |
| 702 | 574 | 2.941176 | 77 | 0 | 820 |
| 703 | 0 | 1.129944 | 70 | 0 | 1082 |
| 703 | 30 | 0.05733 | 70 | 0 | 1082 |
| 703 | 80 | 0.051691 | 70 | 0 | 1082 |
| 703 | 164 | 0.051271 | 70 | 0 | 1082 |
| 703 | 247 | 0.051271 | 70 | 0 | 1082 |
| 703 | 345 | 0.052994 | 70 | 0 | 1082 |
| 703 | 401 | 0.049268 | 70 | 0 | 1082 |
| 703 | 485 | 4.363636 | 70 | 0 | 1082 |
| 703 | 555 | 4.173913 | 70 | 0 | 1082 |

| | | | | | |
|---|---|---|---|---|---|
| 704 | 0 | 16.8 | 45 | 1 | 1309 |
| 704 | 28 | 1.904762 | 45 | 1 | 1309 |
| 704 | 84 | 1.503759 | 45 | 1 | 1309 |
| 704 | 168 | 6.65742 | 45 | 1 | 1309 |
| 704 | 245 | 12.30769 | 45 | 1 | 1309 |
| 704 | 336 | 6.349206 | 45 | 1 | 1309 |
| 704 | 427 | 13.09091 | 45 | 1 | 1309 |
| 704 | 511 | 10.16949 | 45 | 1 | 1309 |
| 704 | 595 | 10.25641 | 45 | 1 | 1309 |
| 705 | 0 | 0.930233 | 67 | 0 | 1086 |
| 705 | 35 | 1.983471 | 67 | 0 | 1086 |
| 706 | 0 | 6.100218 | 88 | 0 | 1265 |
| 706 | 27 | 12.2449 | 88 | 0 | 1265 |
| 706 | 83 | 12.37113 | 88 | 0 | 1265 |
| 706 | 146 | 7.291667 | 88 | 0 | 1265 |
| 706 | 230 | 25.53191 | 88 | 0 | 1265 |
| 706 | 328 | 26.08696 | 88 | 0 | 1265 |
| 706 | 426 | 4.933196 | 88 | 0 | 1265 |
| 707 | 0 | 1.219512 | 83 | 0 | 1086 |
| 707 | 28 | 1.12782 | 83 | 0 | 1086 |
| 707 | 84 | 2.752294 | 83 | 0 | 1086 |
| 707 | 168 | 5.357143 | 83 | 0 | 1086 |
| 707 | 252 | 5.042017 | 83 | 0 | 1086 |
| 707 | 343 | 6.837607 | 83 | 0 | 1086 |
| 707 | 420 | 3.539823 | 83 | 0 | 1086 |
| 707 | 504 | 3.418803 | 83 | 0 | 1086 |
| 707 | 581 | 2.564103 | 83 | 0 | 1086 |
| 708 | 0 | 0.544482 | 48 | 0 | 1111 |
| 709 | 0 | 2.631579 | 53 | 1 | 673 |
| 709 | 28 | 2.162162 | 53 | 1 | 673 |
| 709 | 84 | 1.834862 | 53 | 1 | 673 |
| 709 | 154 | 3.921569 | 53 | 1 | 673 |
| 709 | 252 | 2.564103 | 53 | 1 | 673 |
| 709 | 336 | 15 | 53 | 1 | 673 |
| 709 | 434 | 8.791209 | 53 | 1 | 673 |
| 709 | 490 | 11.11111 | 53 | 1 | 673 |
| 710 | 0 | 5.714286 | 44 | 0 | 967 |
| 710 | 77 | 9.50495 | 44 | 0 | 967 |
| 710 | 182 | 1.709402 | 44 | 0 | 967 |

| | | | | | |
|---|---|---|---|---|---|
| 710 | 252 | 6.122449 | 44 | 0 | 967 |
| 710 | 315 | 2.542373 | 44 | 0 | 967 |
| 710 | 406 | 4.8 | 44 | 0 | 967 |
| 710 | 511 | 5.309735 | 44 | 0 | 967 |
| 710 | 602 | 5.454545 | 44 | 0 | 967 |
| 711 | 0 | 2.424242 | 50 | 0 | 701 |
| 711 | 70 | 1.694915 | 50 | 0 | 701 |
| 711 | 175 | 4.210526 | 50 | 0 | 701 |
| 711 | 231 | 8.807339 | 50 | 0 | 701 |
| 711 | 308 | 6.666667 | 50 | 0 | 701 |
| 711 | 392 | 6.896552 | 50 | 0 | 701 |
| 711 | 518 | 7.207207 | 50 | 0 | 701 |
| 711 | 560 | 11.88119 | 50 | 0 | 701 |
| 712 | 0 | 2.727273 | 68 | 0 | 1296 |
| 712 | 28 | 0.952381 | 68 | 0 | 1296 |
| 712 | 77 | 1.709402 | 68 | 0 | 1296 |
| 712 | 175 | 1.339286 | 68 | 0 | 1296 |
| 712 | 231 | 3.204272 | 68 | 0 | 1296 |
| 712 | 336 | 2.702703 | 68 | 0 | 1296 |
| 712 | 448 | 2.752294 | 68 | 0 | 1296 |
| 712 | 504 | 2.492212 | 68 | 0 | 1296 |
| 712 | 567 | 2.515723 | 68 | 0 | 1296 |
| 713 | 0 | 7.692308 | 58 | 1 | 562 |
| 713 | 28 | 6.976744 | 58 | 1 | 562 |
| 713 | 56 | 13.33333 | 58 | 1 | 562 |
| 713 | 168 | 5.925926 | 58 | 1 | 562 |
| 713 | 245 | 15.78947 | 58 | 1 | 562 |
| 713 | 329 | 10.99237 | 58 | 1 | 562 |
| 713 | 392 | 11.33858 | 58 | 1 | 562 |
| 713 | 490 | 12.41379 | 58 | 1 | 562 |
| 714 | 0 | 1.129944 | 64 | 1 | 611 |
| 714 | 70 | 0.803443 | 64 | 1 | 611 |
| 714 | 154 | 2.285714 | 64 | 1 | 611 |
| 714 | 245 | 5.940594 | 64 | 1 | 611 |
| 714 | 329 | 3.809524 | 64 | 1 | 611 |
| 714 | 413 | 4.897959 | 64 | 1 | 611 |
| 714 | 504 | 20.22472 | 64 | 1 | 611 |
| 714 | 581 | 81.81818 | 64 | 1 | 611 |
| 715 | 0 | 5.825243 | 59 | 1 | 196 |

| | | | | | |
|---|---|---|---|---|---|
| 715 | 28 | 5.714286 | 59 | 1 | 196 |
| 715 | 56 | 4.615385 | 59 | 1 | 196 |
| 715 | 175 | 16.32653 | 59 | 1 | 196 |
| 716 | 0 | 4.324324 | 64 | 0 | 723 |
| 716 | 35 | 2.086957 | 64 | 0 | 723 |
| 716 | 70 | 6.857143 | 64 | 0 | 723 |
| 716 | 133 | 6.923077 | 64 | 0 | 723 |
| 716 | 230 | 6.923077 | 64 | 0 | 723 |
| 716 | 295 | 6 | 64 | 0 | 723 |
| 716 | 386 | 4.593301 | 64 | 0 | 723 |
| 716 | 498 | 16.94118 | 64 | 0 | 723 |
| 716 | 561 | 17.14286 | 64 | 0 | 723 |
| 717 | 0 | 5.042017 | 72 | 1 | 165 |
| 717 | 28 | 3.658537 | 72 | 1 | 165 |
| 718 | 0 | 4.067797 | 66 | 0 | 692 |
| 718 | 35 | 10.71429 | 66 | 0 | 692 |
| 718 | 98 | 6.956522 | 66 | 0 | 692 |
| 718 | 161 | 9.142857 | 66 | 0 | 692 |
| 718 | 252 | 8.571429 | 66 | 0 | 692 |
| 718 | 315 | 10.81081 | 66 | 0 | 692 |
| 719 | 0 | 2.362205 | 78 | 1 | 1049 |
| 719 | 28 | 1.209677 | 78 | 1 | 1049 |
| 719 | 84 | 1.212121 | 78 | 1 | 1049 |
| 719 | 168 | 1.846154 | 78 | 1 | 1049 |
| 719 | 259 | 4.593301 | 78 | 1 | 1049 |
| 719 | 336 | 6.117247 | 78 | 1 | 1049 |
| 719 | 413 | 5.555556 | 78 | 1 | 1049 |
| 719 | 497 | 6.382979 | 78 | 1 | 1049 |
| 719 | 581 | 6.060606 | 78 | 1 | 1049 |
| 720 | 0 | 13.09091 | 86 | 0 | 960 |
| 720 | 35 | 6.837607 | 86 | 0 | 960 |
| 720 | 98 | 7.272727 | 86 | 0 | 960 |
| 720 | 155 | 7.33945 | 86 | 0 | 960 |
| 720 | 218 | 7.258065 | 86 | 0 | 960 |
| 720 | 343 | 3.296703 | 86 | 0 | 960 |
| 720 | 434 | 3.88664 | 86 | 0 | 960 |
| 720 | 532 | 5.811138 | 86 | 0 | 960 |
| 720 | 623 | 5.574913 | 86 | 0 | 960 |
| 721 | 0 | 11.21495 | 74 | 0 | 883 |

| | | | | | |
|---|---|---|---|---|---|
| 721 | 28 | 5 | 74 | 0 | 883 |
| 721 | 119 | 4.615385 | 74 | 0 | 883 |
| 721 | 148 | 7.407407 | 74 | 0 | 883 |
| 721 | 252 | 5.042017 | 74 | 0 | 883 |
| 721 | 343 | 9.89011 | 74 | 0 | 883 |
| 721 | 462 | 17.14286 | 74 | 0 | 883 |
| 722 | 0 | 8.088589 | 68 | 1 | 451 |
| 722 | 40 | 23.68421 | 68 | 1 | 451 |
| 722 | 68 | 6.237624 | 68 | 1 | 451 |
| 722 | 188 | 15.78947 | 68 | 1 | 451 |
| 722 | 267 | 15.78947 | 68 | 1 | 451 |
| 722 | 337 | 9.545455 | 68 | 1 | 451 |
| 722 | 440 | 7.446809 | 68 | 1 | 451 |
| 723 | 0 | 14.54545 | 69 | 0 | 562 |
| 723 | 28 | 20.12579 | 69 | 0 | 562 |
| 723 | 77 | 12.35862 | 69 | 0 | 562 |
| 723 | 162 | 8.75 | 69 | 0 | 562 |
| 723 | 253 | 8.403361 | 69 | 0 | 562 |
| 723 | 337 | 9.096816 | 69 | 0 | 562 |
| 723 | 421 | 8.77193 | 69 | 0 | 562 |
| 724 | 0 | 6.122449 | 40 | 0 | 890 |
| 724 | 63 | 13.63636 | 40 | 0 | 890 |
| 724 | 161 | 12.08054 | 40 | 0 | 890 |
| 724 | 224 | 5.479452 | 40 | 0 | 890 |
| 724 | 315 | 5.673759 | 40 | 0 | 890 |
| 724 | 406 | 6.504065 | 40 | 0 | 890 |
| 724 | 469 | 9.836066 | 40 | 0 | 890 |
| 724 | 546 | 10.58824 | 40 | 0 | 890 |
| 725 | 0 | 6 | 80 | 0 | 218 |
| 725 | 28 | 12.5 | 80 | 0 | 218 |
| 726 | 0 | 1.207938 | 55 | 1 | 411 |
| 726 | 33 | 1.904762 | 55 | 1 | 411 |
| 726 | 75 | 1.904762 | 55 | 1 | 411 |
| 726 | 159 | 2.884615 | 55 | 1 | 411 |
| 726 | 250 | 9.142857 | 55 | 1 | 411 |
| 726 | 320 | 4.324324 | 55 | 1 | 411 |
| 726 | 397 | 8.247423 | 55 | 1 | 411 |
| 727 | 0 | 3.669725 | 76 | 1 | 520 |
| 727 | 28 | 2.807018 | 76 | 1 | 520 |

| | | | | | |
|---|---|---|---|---|---|
| 727 | 84 | 3.478261 | 76 | 1 | 520 |
| 727 | 175 | 5.825243 | 76 | 1 | 520 |
| 727 | 231 | 8.571429 | 76 | 1 | 520 |
| 727 | 322 | 9.056604 | 76 | 1 | 520 |
| 727 | 413 | 15.78947 | 76 | 1 | 520 |
| 728 | 0 | 2.5 | 69 | 0 | 1002 |
| 728 | 56 | 2.033898 | 69 | 0 | 1002 |
| 728 | 154 | 2.752294 | 69 | 0 | 1002 |
| 728 | 245 | 4.918033 | 69 | 0 | 1002 |
| 728 | 329 | 4.210526 | 69 | 0 | 1002 |
| 728 | 441 | 1.983471 | 69 | 0 | 1002 |
| 728 | 546 | 2.033898 | 69 | 0 | 1002 |
| 729 | 0 | 1.093181 | 49 | 1 | 264 |
| 729 | 25 | 1.027397 | 49 | 1 | 264 |
| 729 | 98 | 1.085552 | 49 | 1 | 264 |
| 729 | 175 | 1.955535 | 49 | 1 | 264 |
| 729 | 246 | 1.955535 | 49 | 1 | 264 |
| 730 | 0 | 3.100775 | 49 | 1 | 4 |
| 731 | 0 | 0.646552 | 84 | 0 | 1004 |
| 731 | 29 | 0.663717 | 84 | 0 | 1004 |
| 731 | 73 | 0.614754 | 84 | 0 | 1004 |
| 731 | 184 | 2.125506 | 84 | 0 | 1004 |
| 731 | 254 | 0.965406 | 84 | 0 | 1004 |
| 731 | 338 | 4.255319 | 84 | 0 | 1004 |
| 732 | 16 | 6.451613 | 72 | 1 | 644 |
| 732 | 63 | 5.940594 | 72 | 1 | 644 |
| 732 | 147 | 5.454545 | 72 | 1 | 644 |
| 732 | 238 | 6.185567 | 72 | 1 | 644 |
| 732 | 329 | 10.21277 | 72 | 1 | 644 |
| 732 | 399 | 12.2449 | 72 | 1 | 644 |
| 732 | 490 | 10.66667 | 72 | 1 | 644 |
| 732 | 574 | 14.69388 | 72 | 1 | 644 |
| 733 | 0 | 2.242991 | 80 | 0 | 925 |
| 733 | 35 | 1.442308 | 80 | 0 | 925 |
| 733 | 91 | 0.900901 | 80 | 0 | 925 |
| 733 | 175 | 1.568627 | 80 | 0 | 925 |
| 733 | 280 | 1.538462 | 80 | 0 | 925 |
| 733 | 336 | 3.636364 | 80 | 0 | 925 |
| 733 | 427 | 4.984424 | 80 | 0 | 925 |

| | | | | | |
|---|---|---|---|---|---|
| 733 | 511 | 5.76 | 80 | 0 | 925 |
| 733 | 581 | 6.101695 | 80 | 0 | 925 |
| 734 | 0 | 2.853261 | 50 | 0 | 833 |
| 734 | 24 | 3.502919 | 50 | 0 | 833 |
| 734 | 97 | 4.95137 | 50 | 0 | 833 |
| 734 | 160 | 5.172414 | 50 | 0 | 833 |
| 734 | 251 | 5.263158 | 50 | 0 | 833 |
| 734 | 335 | 3.478261 | 50 | 0 | 833 |
| 734 | 426 | 3.844394 | 50 | 0 | 833 |
| 734 | 482 | 5.172414 | 50 | 0 | 833 |
| 734 | 573 | 5.504587 | 50 | 0 | 833 |
| 735 | 0 | 2.608696 | 77 | 0 | 1016 |
| 735 | 28 | 2.654867 | 77 | 0 | 1016 |
| 735 | 84 | 5.172414 | 77 | 0 | 1016 |
| 735 | 168 | 1.95122 | 77 | 0 | 1016 |
| 735 | 238 | 4.40367 | 77 | 0 | 1016 |
| 735 | 336 | 2.34375 | 77 | 0 | 1016 |
| 735 | 420 | 2.051282 | 77 | 0 | 1016 |
| 735 | 511 | 2.419355 | 77 | 0 | 1016 |
| 735 | 595 | 1.315789 | 77 | 0 | 1016 |
| 736 | 0 | 3.921569 | 85 | 0 | 510 |
| 736 | 28 | 5.882353 | 85 | 0 | 510 |
| 736 | 84 | 15.68627 | 85 | 0 | 510 |
| 736 | 154 | 11.76471 | 85 | 0 | 510 |
| 737 | 0 | 5 | 72 | 0 | 708 |
| 737 | 28 | 1.568627 | 72 | 0 | 708 |
| 737 | 73 | 1.490683 | 72 | 0 | 708 |
| 737 | 203 | 1.694915 | 72 | 0 | 708 |
| 737 | 305 | 2.046784 | 72 | 0 | 708 |
| 737 | 399 | 2.542373 | 72 | 0 | 708 |
| 737 | 497 | 1.012658 | 72 | 0 | 708 |
| 737 | 581 | 1.012658 | 72 | 0 | 708 |
| 738 | 0 | 2.016807 | 67 | 0 | 652 |
| 738 | 35 | 1.269841 | 67 | 0 | 652 |
| 739 | 0 | 2.702703 | 68 | 1 | 458 |
| 739 | 42 | 9.230769 | 68 | 1 | 458 |
| 739 | 182 | 8.888889 | 68 | 1 | 458 |
| 739 | 217 | 9.6 | 68 | 1 | 458 |
| 739 | 357 | 12.2807 | 68 | 1 | 458 |

| | | | | | |
|---|---|---|---|---|---|
| 739 | 441 | 19.14894 | 68 | 1 | 458 |
| 740 | 0 | 3.703704 | 78 | 0 | 946 |
| 740 | 14 | 1.73913 | 78 | 0 | 946 |
| 740 | 70 | 1.869159 | 78 | 0 | 946 |
| 740 | 154 | 3.921569 | 78 | 0 | 946 |
| 740 | 266 | 3.809524 | 78 | 0 | 946 |
| 740 | 350 | 3.921569 | 78 | 0 | 946 |
| 740 | 406 | 1.206637 | 78 | 0 | 946 |
| 740 | 497 | 4 | 78 | 0 | 946 |
| 740 | 581 | 3.333333 | 78 | 0 | 946 |
| 741 | 0 | 3.428571 | 83 | 0 | 855 |
| 741 | 77 | 12.76596 | 83 | 0 | 855 |
| 741 | 161 | 9.320388 | 83 | 0 | 855 |
| 741 | 224 | 12.2449 | 83 | 0 | 855 |
| 741 | 343 | 12.12121 | 83 | 0 | 855 |
| 741 | 427 | 9.142857 | 83 | 0 | 855 |
| 741 | 490 | 10.32258 | 83 | 0 | 855 |
| 741 | 588 | 10.43478 | 83 | 0 | 855 |
| 742 | 0 | 2.608696 | 71 | 1 | 611 |
| 742 | 28 | 5.490196 | 71 | 1 | 611 |
| 742 | 91 | 6.060606 | 71 | 1 | 611 |
| 742 | 182 | 4.363636 | 71 | 1 | 611 |
| 742 | 252 | 6.185567 | 71 | 1 | 611 |
| 742 | 336 | 5.607477 | 71 | 1 | 611 |
| 742 | 420 | 5.714286 | 71 | 1 | 611 |
| 742 | 524 | 25.74713 | 71 | 1 | 611 |
| 742 | 595 | 5.217391 | 71 | 1 | 611 |
| 743 | 0 | 1.851852 | 42 | 0 | 94 |
| 743 | 56 | 2.702703 | 42 | 0 | 94 |
| 744 | 0 | 4.848485 | 81 | 0 | 936 |
| 744 | 35 | 6.060606 | 81 | 0 | 936 |
| 744 | 98 | 4.363636 | 81 | 0 | 936 |
| 744 | 168 | 5.714286 | 81 | 0 | 936 |
| 744 | 252 | 6.185567 | 81 | 0 | 936 |
| 744 | 338 | 11.11111 | 81 | 0 | 936 |
| 744 | 436 | 9.69697 | 81 | 0 | 936 |
| 744 | 505 | 5.177196 | 81 | 0 | 936 |
| 744 | 555 | 22.78481 | 81 | 0 | 936 |
| 745 | 0 | 11.42857 | 42 | 0 | 1126 |

| | | | | | |
|---|---|---|---|---|---|
| 745 | 70 | 11.80328 | 42 | 0 | 1126 |
| 745 | 168 | 13.13869 | 42 | 0 | 1126 |
| 745 | 231 | 12 | 42 | 0 | 1126 |
| 745 | 343 | 12.10084 | 42 | 0 | 1126 |
| 745 | 406 | 14.87603 | 42 | 0 | 1126 |
| 745 | 504 | 11.52 | 42 | 0 | 1126 |
| 745 | 567 | 11.42857 | 42 | 0 | 1126 |
| 746 | 0 | 2.542373 | 78 | 0 | 333 |
| 746 | 28 | 1.339286 | 78 | 0 | 333 |
| 746 | 84 | 4.347826 | 78 | 0 | 333 |
| 746 | 168 | 12.37113 | 78 | 0 | 333 |
| 746 | 252 | 11.21495 | 78 | 0 | 333 |
| 747 | 0 | 4.022989 | 76 | 0 | 659 |
| 747 | 63 | 1.666667 | 76 | 0 | 659 |
| 747 | 147 | 1.869159 | 76 | 0 | 659 |
| 747 | 266 | 3.636364 | 76 | 0 | 659 |
| 747 | 336 | 3.508772 | 76 | 0 | 659 |
| 747 | 395 | 1.851852 | 76 | 0 | 659 |
| 747 | 494 | 4.255319 | 76 | 0 | 659 |
| 747 | 585 | 3.298969 | 76 | 0 | 659 |
| 748 | 0 | 12.63158 | 66 | 1 | 338 |
| 748 | 112 | 5.825243 | 66 | 1 | 338 |
| 748 | 196 | 10.43478 | 66 | 1 | 338 |
| 748 | 281 | 9.343715 | 66 | 1 | 338 |
| 749 | 0 | 12.92308 | 59 | 0 | 903 |
| 749 | 25 | 12.21818 | 59 | 0 | 903 |
| 749 | 90 | 8.888889 | 59 | 0 | 903 |
| 749 | 160 | 10.90909 | 59 | 0 | 903 |
| 749 | 272 | 8.677686 | 59 | 0 | 903 |
| 749 | 384 | 9.917355 | 59 | 0 | 903 |
| 749 | 447 | 10.71429 | 59 | 0 | 903 |
| 749 | 510 | 11.11111 | 59 | 0 | 903 |
| 749 | 566 | 10 | 59 | 0 | 903 |
| 750 | 0 | 4.633205 | 51 | 0 | 556 |
| 750 | 79 | 12 | 51 | 0 | 556 |
| 750 | 191 | 6 | 51 | 0 | 556 |
| 750 | 254 | 7.54717 | 51 | 0 | 556 |
| 750 | 324 | 11.76471 | 51 | 0 | 556 |
| 750 | 408 | 11.21495 | 51 | 0 | 556 |

| | | | | | |
|---|---|---|---|---|---|
| 750 | 492 | 10 | 51 | 0 | 556 |
| 751 | 0 | 2.330097 | 64 | 0 | 982 |
| 751 | 35 | 2.912621 | 64 | 0 | 982 |
| 751 | 63 | 1.648352 | 64 | 0 | 982 |
| 751 | 175 | 7.33945 | 64 | 0 | 982 |
| 751 | 259 | 9.6 | 64 | 0 | 982 |
| 751 | 343 | 5.333333 | 64 | 0 | 982 |
| 751 | 413 | 10.25641 | 64 | 0 | 982 |
| 751 | 512 | 18.18182 | 64 | 0 | 982 |
| 751 | 568 | 16.36364 | 64 | 0 | 982 |
| 752 | 0 | 2.631579 | 71 | 0 | 776 |
| 752 | 28 | 1.587302 | 71 | 0 | 776 |
| 752 | 124 | 1.52381 | 71 | 0 | 776 |
| 752 | 196 | 1.441441 | 71 | 0 | 776 |
| 752 | 266 | 1.111111 | 71 | 0 | 776 |
| 752 | 350 | 1.139601 | 71 | 0 | 776 |
| 752 | 420 | 0.862069 | 71 | 0 | 776 |
| 752 | 532 | 1.363636 | 71 | 0 | 776 |
| 753 | 0 | 2.12766 | 80 | 0 | 1086 |
| 753 | 28 | 2.439024 | 80 | 0 | 1086 |
| 753 | 91 | 2.631579 | 80 | 0 | 1086 |
| 753 | 175 | 2.459016 | 80 | 0 | 1086 |
| 753 | 259 | 2.586207 | 80 | 0 | 1086 |
| 753 | 350 | 1.983471 | 80 | 0 | 1086 |
| 753 | 434 | 2.521008 | 80 | 0 | 1086 |
| 753 | 497 | 3.361345 | 80 | 0 | 1086 |
| 753 | 581 | 2.27704 | 80 | 0 | 1086 |
| 754 | 0 | 5.357143 | 59 | 1 | 79 |
| 754 | 28 | 7.76699 | 59 | 1 | 79 |
| 755 | 0 | 5.263158 | 84 | 1 | 880 |
| 755 | 28 | 4.958678 | 84 | 1 | 880 |
| 755 | 84 | 5.607477 | 84 | 1 | 880 |
| 755 | 171 | 3.603604 | 84 | 1 | 880 |
| 755 | 248 | 9 | 84 | 1 | 880 |
| 755 | 346 | 3.445447 | 84 | 1 | 880 |
| 755 | 423 | 8.562691 | 84 | 1 | 880 |
| 755 | 507 | 14.28571 | 84 | 1 | 880 |
| 755 | 626 | 6.130268 | 84 | 1 | 880 |
| 756 | 0 | 4.660194 | 75 | 1 | 617 |

| | | | | | |
|---|---|---|---|---|---|
| 756 | 35 | 1.517341 | 75 | 1 | 617 |
| 756 | 124 | 1.244998 | 75 | 1 | 617 |
| 756 | 180 | 1.600711 | 75 | 1 | 617 |
| 756 | 292 | 14.4 | 75 | 1 | 617 |
| 756 | 362 | 34.63918 | 75 | 1 | 617 |
| 756 | 432 | 20.22472 | 75 | 1 | 617 |
| 756 | 516 | 19.35484 | 75 | 1 | 617 |
| 756 | 572 | 19.14894 | 75 | 1 | 617 |
| 757 | 0 | 11.11111 | 49 | 1 | 301 |
| 757 | 28 | 2.380952 | 49 | 1 | 301 |
| 757 | 84 | 5.555556 | 49 | 1 | 301 |
| 757 | 161 | 7.920792 | 49 | 1 | 301 |
| 757 | 238 | 7.346939 | 49 | 1 | 301 |
| 758 | 0 | 6.422018 | 75 | 0 | 148 |
| 758 | 105 | 12.51863 | 75 | 0 | 148 |
| 758 | 147 | 8.695652 | 75 | 0 | 148 |
| 759 | 0 | 3.607085 | 61 | 0 | 687 |
| 759 | 28 | 2.758621 | 61 | 0 | 687 |
| 759 | 98 | 2.735043 | 61 | 0 | 687 |
| 759 | 133 | 2.93578 | 61 | 0 | 687 |
| 759 | 224 | 2.882883 | 61 | 0 | 687 |
| 759 | 329 | 3.636364 | 61 | 0 | 687 |
| 759 | 448 | 3.448276 | 61 | 0 | 687 |
| 759 | 511 | 3.508772 | 61 | 0 | 687 |
| 759 | 567 | 3.636364 | 61 | 0 | 687 |
| 760 | 0 | 5.504587 | 47 | 1 | 632 |
| 760 | 28 | 5.454545 | 47 | 1 | 632 |
| 760 | 76 | 1.428571 | 47 | 1 | 632 |
| 760 | 161 | 1.071429 | 47 | 1 | 632 |
| 760 | 252 | 0.952381 | 47 | 1 | 632 |
| 760 | 329 | 1.376147 | 47 | 1 | 632 |
| 760 | 413 | 1.52381 | 47 | 1 | 632 |
| 760 | 483 | 2.990654 | 47 | 1 | 632 |
| 760 | 581 | 2.150538 | 47 | 1 | 632 |
| 761 | 0 | 5.825243 | 65 | 1 | 274 |
| 761 | 84 | 5.882353 | 65 | 1 | 274 |
| 761 | 168 | 2.912621 | 65 | 1 | 274 |
| 761 | 252 | 11.53846 | 65 | 1 | 274 |
| 762 | 0 | 4.83871 | 73 | 0 | 1198 |

| | | | | | |
|---|---|---|---|---|---|
| 762 | 28 | 4.067797 | 73 | 0 | 1198 |
| 762 | 98 | 6.504065 | 73 | 0 | 1198 |
| 762 | 177 | 2.466236 | 73 | 0 | 1198 |
| 762 | 259 | 2.419355 | 73 | 0 | 1198 |
| 762 | 343 | 4.247788 | 73 | 0 | 1198 |
| 762 | 427 | 4.033613 | 73 | 0 | 1198 |
| 762 | 511 | 4.444444 | 73 | 0 | 1198 |
| 762 | 595 | 4.651163 | 73 | 0 | 1198 |
| 763 | 0 | 2.258065 | 65 | 0 | 751 |
| 763 | 30 | 4.95137 | 65 | 0 | 751 |
| 763 | 70 | 3.846154 | 65 | 0 | 751 |
| 763 | 147 | 9.278351 | 65 | 0 | 751 |
| 763 | 238 | 9.230769 | 65 | 0 | 751 |
| 763 | 343 | 10.80617 | 65 | 0 | 751 |
| 763 | 420 | 11.53846 | 65 | 0 | 751 |
| 763 | 490 | 9.056604 | 65 | 0 | 751 |
| 763 | 597 | 11.01399 | 65 | 0 | 751 |
| 764 | 0 | 1.801802 | 60 | 0 | 680 |
| 764 | 42 | 2.5 | 60 | 0 | 680 |
| 764 | 153 | 3.111111 | 60 | 0 | 680 |
| 764 | 252 | 7.881773 | 60 | 0 | 680 |
| 764 | 357 | 6.666667 | 60 | 0 | 680 |
| 764 | 420 | 11.17517 | 60 | 0 | 680 |
| 764 | 511 | 15.38462 | 60 | 0 | 680 |
| 764 | 574 | 14.69388 | 60 | 0 | 680 |
| 765 | 0 | 0.335062 | 85 | 0 | 702 |
| 765 | 78 | 0.323799 | 85 | 0 | 702 |
| 766 | 0 | 5.714286 | 69 | 0 | 1044 |
| 766 | 28 | 2.830189 | 69 | 0 | 1044 |
| 766 | 84 | 4.5 | 69 | 0 | 1044 |
| 766 | 139 | 4.411765 | 69 | 0 | 1044 |
| 766 | 251 | 15.31915 | 69 | 0 | 1044 |
| 766 | 315 | 12.63158 | 69 | 0 | 1044 |
| 766 | 385 | 10.52632 | 69 | 0 | 1044 |
| 766 | 497 | 5.853659 | 69 | 0 | 1044 |
| 766 | 587 | 10.81081 | 69 | 0 | 1044 |
| 767 | 0 | 5.825243 | 68 | 0 | 757 |
| 767 | 28 | 7.058824 | 68 | 0 | 757 |
| 767 | 63 | 11.65049 | 68 | 0 | 757 |

| | | | | | |
|---|---|---|---|---|---|
| 767 | 161 | 11.00917 | 68 | 0 | 757 |
| 767 | 245 | 7.933884 | 68 | 0 | 757 |
| 767 | 315 | 6 | 68 | 0 | 757 |
| 767 | 408 | 4.958678 | 68 | 0 | 757 |
| 767 | 511 | 5.309735 | 68 | 0 | 757 |
| 767 | 574 | 4.285714 | 68 | 0 | 757 |
| 768 | 0 | 3 | 58 | 1 | 282 |
| 768 | 28 | 4.918033 | 58 | 1 | 282 |
| 768 | 84 | 3 | 58 | 1 | 282 |
| 768 | 182 | 12.85141 | 58 | 1 | 282 |
| 769 | 0 | 10.90909 | 71 | 1 | 509 |
| 769 | 27 | 4.40367 | 71 | 1 | 509 |
| 769 | 82 | 5.405405 | 71 | 1 | 509 |
| 769 | 165 | 3.125 | 71 | 1 | 509 |
| 769 | 238 | 5.660377 | 71 | 1 | 509 |
| 769 | 321 | 3.030303 | 71 | 1 | 509 |
| 769 | 402 | 11.29032 | 71 | 1 | 509 |
| 769 | 476 | 21.05263 | 71 | 1 | 509 |
| 770 | 0 | 4.705882 | 47 | 0 | 589 |
| 770 | 70 | 8.333333 | 47 | 0 | 589 |
| 770 | 161 | 8 | 47 | 0 | 589 |
| 770 | 203 | 5.555556 | 47 | 0 | 589 |
| 771 | 0 | 2.836879 | 70 | 1 | 339 |
| 771 | 42 | 2.173913 | 70 | 1 | 339 |
| 771 | 98 | 2.020202 | 70 | 1 | 339 |
| 772 | 0 | 4.948454 | 77 | 0 | 591 |
| 772 | 35 | 6.339623 | 77 | 0 | 591 |
| 772 | 63 | 4.444444 | 77 | 0 | 591 |
| 772 | 161 | 4.285714 | 77 | 0 | 591 |
| 772 | 224 | 3.636364 | 77 | 0 | 591 |
| 772 | 338 | 5.714286 | 77 | 0 | 591 |
| 772 | 394 | 5.128205 | 77 | 0 | 591 |
| 772 | 485 | 4.137931 | 77 | 0 | 591 |
| 773 | 0 | 12.6506 | 84 | 0 | 337 |
| 773 | 28 | 15 | 84 | 0 | 337 |
| 773 | 84 | 14.63415 | 84 | 0 | 337 |
| 773 | 168 | 23.30097 | 84 | 0 | 337 |
| 773 | 252 | 19.67213 | 84 | 0 | 337 |
| 774 | 0 | 1.573034 | 73 | 0 | 912 |

| | | | | | |
|---|---|---|---|---|---|
| 774 | 35 | 4.247788 | 73 | 0 | 912 |
| 774 | 70 | 2.492212 | 73 | 0 | 912 |
| 774 | 161 | 5.266458 | 73 | 0 | 912 |
| 774 | 252 | 2.469136 | 73 | 0 | 912 |
| 775 | 0 | 2.4 | 68 | 0 | 1239 |
| 775 | 28 | 2.325581 | 68 | 0 | 1239 |
| 775 | 77 | 3.508772 | 68 | 0 | 1239 |
| 775 | 168 | 5.660377 | 68 | 0 | 1239 |
| 775 | 259 | 5.263158 | 68 | 0 | 1239 |
| 775 | 322 | 5.357143 | 68 | 0 | 1239 |
| 775 | 406 | 7.54717 | 68 | 0 | 1239 |
| 775 | 511 | 4.6875 | 68 | 0 | 1239 |
| 775 | 594 | 2.125506 | 68 | 0 | 1239 |
| 776 | 0 | 1.538462 | 64 | 0 | 1266 |
| 776 | 28 | 2.086957 | 64 | 0 | 1266 |
| 776 | 63 | 4.571429 | 64 | 0 | 1266 |
| 776 | 189 | 5.454545 | 64 | 0 | 1266 |
| 776 | 245 | 4.102564 | 64 | 0 | 1266 |
| 776 | 308 | 3.571429 | 64 | 0 | 1266 |
| 776 | 385 | 5.172414 | 64 | 0 | 1266 |
| 776 | 469 | 7.54717 | 64 | 0 | 1266 |
| 776 | 553 | 2.857143 | 64 | 0 | 1266 |
| 777 | 0 | 1.070234 | 75 | 1 | 523 |
| 777 | 24 | 1.997503 | 75 | 1 | 523 |
| 777 | 148 | 5.474096 | 75 | 1 | 523 |
| 777 | 241 | 13.74795 | 75 | 1 | 523 |
| 777 | 339 | 3.316226 | 75 | 1 | 523 |
| 777 | 428 | 4.281709 | 75 | 1 | 523 |
| 778 | 0 | 3.361345 | 74 | 0 | 1010 |
| 778 | 21 | 1.101928 | 74 | 0 | 1010 |
| 778 | 84 | 1.206637 | 74 | 0 | 1010 |
| 778 | 175 | 2.097902 | 74 | 0 | 1010 |
| 778 | 252 | 3.450755 | 74 | 0 | 1010 |
| 778 | 343 | 6.35481 | 74 | 0 | 1010 |
| 778 | 420 | 5.454545 | 74 | 0 | 1010 |
| 778 | 504 | 5.882353 | 74 | 0 | 1010 |
| 778 | 588 | 5.769231 | 74 | 0 | 1010 |
| 779 | 0 | 1.074169 | 64 | 0 | 1046 |
| 779 | 79 | 3.636364 | 64 | 0 | 1046 |

| | | | | | |
|---|---|---|---|---|---|
| 779 | 177 | 1.769912 | 64 | 0 | 1046 |
| 779 | 233 | 2.803738 | 64 | 0 | 1046 |
| 779 | 338 | 2.051282 | 64 | 0 | 1046 |
| 779 | 401 | 2.521008 | 64 | 0 | 1046 |
| 779 | 492 | 1.138057 | 64 | 0 | 1046 |
| 779 | 590 | 1.818182 | 64 | 0 | 1046 |
| 780 | 0 | 2.439024 | 86 | 0 | 1091 |
| 780 | 28 | 2 | 86 | 0 | 1091 |
| 780 | 98 | 2.142857 | 86 | 0 | 1091 |
| 780 | 182 | 2.521008 | 86 | 0 | 1091 |
| 780 | 273 | 1.724138 | 86 | 0 | 1091 |
| 780 | 343 | 1.129944 | 86 | 0 | 1091 |
| 780 | 427 | 1.129944 | 86 | 0 | 1091 |
| 780 | 511 | 0.746965 | 86 | 0 | 1091 |
| 780 | 574 | 1.234568 | 86 | 0 | 1091 |
| 781 | 0 | 2.857143 | 53 | 1 | 475 |
| 781 | 28 | 3 | 53 | 1 | 475 |
| 781 | 84 | 4.571429 | 53 | 1 | 475 |
| 781 | 175 | 5.607477 | 53 | 1 | 475 |
| 781 | 238 | 4.363636 | 53 | 1 | 475 |
| 781 | 308 | 16.66667 | 53 | 1 | 475 |
| 781 | 420 | 6.997085 | 53 | 1 | 475 |
| 781 | 469 | 6.068268 | 53 | 1 | 475 |
| 782 | 0 | 4.752475 | 76 | 1 | 272 |
| 782 | 35 | 5.052632 | 76 | 1 | 272 |
| 782 | 98 | 5.714286 | 76 | 1 | 272 |
| 782 | 189 | 14.63415 | 76 | 1 | 272 |
| 782 | 245 | 8.421053 | 76 | 1 | 272 |
| 783 | 0 | 2.727273 | 68 | 0 | 899 |
| 783 | 28 | 2.752294 | 68 | 0 | 899 |
| 783 | 84 | 2.777778 | 68 | 0 | 899 |
| 783 | 170 | 2.991453 | 68 | 0 | 899 |
| 783 | 254 | 2.201835 | 68 | 0 | 899 |
| 783 | 345 | 2.970297 | 68 | 0 | 899 |
| 783 | 429 | 2.201835 | 68 | 0 | 899 |
| 783 | 527 | 2.941176 | 68 | 0 | 899 |
| 783 | 625 | 2.330097 | 68 | 0 | 899 |
| 784 | 0 | 2.439024 | 54 | 1 | 674 |
| 784 | 21 | 0.769231 | 54 | 1 | 674 |

| | | | | | |
|---|---|---|---|---|---|
| 784 | 105 | 0.943396 | 54 | 1 | 674 |
| 784 | 154 | 7.54717 | 54 | 1 | 674 |
| 784 | 238 | 8.347826 | 54 | 1 | 674 |
| 784 | 329 | 7.017544 | 54 | 1 | 674 |
| 784 | 413 | 10.71429 | 54 | 1 | 674 |
| 784 | 497 | 11.76471 | 54 | 1 | 674 |
| 784 | 574 | 13.71429 | 54 | 1 | 674 |
| 785 | 0 | 4.660194 | 69 | 1 | 260 |
| 785 | 35 | 11.53846 | 69 | 1 | 260 |
| 785 | 91 | 18 | 69 | 1 | 260 |
| 785 | 154 | 9.703504 | 69 | 1 | 260 |
| 785 | 231 | 11.68831 | 69 | 1 | 260 |
| 786 | 0 | 14.40823 | 86 | 0 | 933 |
| 786 | 98 | 13.71429 | 86 | 0 | 933 |
| 786 | 211 | 13.35453 | 86 | 0 | 933 |
| 786 | 316 | 18.75 | 86 | 0 | 933 |
| 786 | 434 | 19.74922 | 86 | 0 | 933 |
| 786 | 526 | 27.92244 | 86 | 0 | 933 |
| 786 | 561 | 23.76238 | 86 | 0 | 933 |
| 787 | 0 | 9.411765 | 92 | 0 | 114 |
| 787 | 29 | 12.76596 | 92 | 0 | 114 |
| 788 | 0 | 3.603604 | 83 | 0 | 948 |
| 788 | 28 | 3.636364 | 83 | 0 | 948 |
| 788 | 84 | 3.539823 | 83 | 0 | 948 |
| 788 | 168 | 3.571429 | 83 | 0 | 948 |
| 788 | 259 | 5.660377 | 83 | 0 | 948 |
| 788 | 343 | 4.485981 | 83 | 0 | 948 |
| 788 | 406 | 5.357143 | 83 | 0 | 948 |
| 788 | 504 | 5.357143 | 83 | 0 | 948 |
| 788 | 588 | 5.825243 | 83 | 0 | 948 |
| 789 | 0 | 5.217391 | 76 | 0 | 690 |
| 789 | 28 | 5.217391 | 76 | 0 | 690 |
| 789 | 84 | 5.555556 | 76 | 0 | 690 |
| 789 | 143 | 5.217391 | 76 | 0 | 690 |
| 789 | 238 | 5.714286 | 76 | 0 | 690 |
| 789 | 329 | 5.940594 | 76 | 0 | 690 |
| 789 | 420 | 5.769231 | 76 | 0 | 690 |
| 789 | 511 | 6.122449 | 76 | 0 | 690 |
| 789 | 602 | 6.122449 | 76 | 0 | 690 |

| | | | | | |
|---|---|---|---|---|---|
| 790 | 0 | 2.727273 | 84 | 0 | 184 |
| 790 | 100 | 9.753266 | 84 | 0 | 184 |
| 790 | 152 | 9.208002 | 84 | 0 | 184 |
| 791 | 0 | 7.207207 | 71 | 0 | 861 |
| 791 | 21 | 5.042017 | 71 | 0 | 861 |
| 791 | 77 | 4.485981 | 71 | 0 | 861 |
| 791 | 147 | 5.607477 | 71 | 0 | 861 |
| 791 | 231 | 11.00917 | 71 | 0 | 861 |
| 791 | 336 | 7.76699 | 71 | 0 | 861 |
| 791 | 413 | 10.52632 | 71 | 0 | 861 |
| 791 | 497 | 10.08403 | 71 | 0 | 861 |
| 791 | 588 | 9.320388 | 71 | 0 | 861 |
| 792 | 0 | 1.666667 | 75 | 0 | 331 |
| 793 | 0 | 9.591778 | 62 | 1 | 127 |
| 793 | 31 | 10.43478 | 62 | 1 | 127 |
| 793 | 87 | 10.25641 | 62 | 1 | 127 |
| 794 | 0 | 2.439024 | 77 | 0 | 1010 |
| 794 | 28 | 2.702703 | 77 | 0 | 1010 |
| 794 | 84 | 3.738318 | 77 | 0 | 1010 |
| 794 | 168 | 5.660377 | 77 | 0 | 1010 |
| 794 | 224 | 9.917355 | 77 | 0 | 1010 |
| 794 | 334 | 5.714286 | 77 | 0 | 1010 |
| 794 | 392 | 5.614035 | 77 | 0 | 1010 |
| 794 | 483 | 5.228758 | 77 | 0 | 1010 |
| 794 | 546 | 5.280528 | 77 | 0 | 1010 |
| 795 | 0 | 5.825243 | 68 | 0 | 459 |
| 795 | 28 | 9.017713 | 68 | 0 | 459 |
| 795 | 86 | 5.370844 | 68 | 0 | 459 |
| 795 | 188 | 5.771213 | 68 | 0 | 459 |
| 795 | 244 | 9.302326 | 68 | 0 | 459 |
| 795 | 345 | 6.100218 | 68 | 0 | 459 |
| 795 | 386 | 6.521739 | 68 | 0 | 459 |
| 796 | 0 | 4.83871 | 76 | 1 | 407 |
| 796 | 28 | 2.564103 | 76 | 1 | 407 |
| 796 | 84 | 3.125 | 76 | 1 | 407 |
| 796 | 168 | 6.060606 | 76 | 1 | 407 |
| 796 | 252 | 7.142857 | 76 | 1 | 407 |
| 796 | 336 | 6.976744 | 76 | 1 | 407 |
| 797 | 0 | 4.958678 | 59 | 0 | 988 |

| | | | | | |
|---|---|---|---|---|---|
| 797 | 28 | 2 | 59 | 0 | 988 |
| 797 | 98 | 2.123894 | 59 | 0 | 988 |
| 797 | 154 | 4.83871 | 59 | 0 | 988 |
| 797 | 245 | 4.958678 | 59 | 0 | 988 |
| 797 | 326 | 4.370447 | 59 | 0 | 988 |
| 797 | 413 | 4.580153 | 59 | 0 | 988 |
| 797 | 497 | 2.479339 | 59 | 0 | 988 |
| 798 | 0 | 5.555556 | 65 | 0 | 589 |
| 798 | 28 | 5.263158 | 65 | 0 | 589 |
| 798 | 84 | 10.71429 | 65 | 0 | 589 |
| 798 | 182 | 5.454545 | 65 | 0 | 589 |
| 798 | 266 | 7.476636 | 65 | 0 | 589 |
| 798 | 322 | 16.51376 | 65 | 0 | 589 |
| 798 | 427 | 13.33333 | 65 | 0 | 589 |
| 798 | 511 | 16.66667 | 65 | 0 | 589 |
| 799 | 0 | 2.264151 | 70 | 1 | 204 |
| 799 | 105 | 4.081633 | 70 | 1 | 204 |
| 799 | 172 | 1.960784 | 70 | 1 | 204 |
| 800 | 0 | 4.615385 | 73 | 0 | 1144 |
| 800 | 49 | 1.459854 | 73 | 0 | 1144 |
| 800 | 175 | 1.769912 | 73 | 0 | 1144 |
| 800 | 203 | 2.086957 | 73 | 0 | 1144 |
| 800 | 308 | 2.033898 | 73 | 0 | 1144 |
| 800 | 378 | 2.631579 | 73 | 0 | 1144 |
| 801 | 0 | 2.105263 | 81 | 0 | 638 |
| 801 | 21 | 5.405405 | 81 | 0 | 638 |
| 801 | 77 | 5.454545 | 81 | 0 | 638 |
| 801 | 168 | 7.33945 | 81 | 0 | 638 |
| 801 | 245 | 4.067797 | 81 | 0 | 638 |
| 801 | 343 | 6.779661 | 81 | 0 | 638 |
| 801 | 399 | 3.2 | 81 | 0 | 638 |
| 801 | 497 | 2.654867 | 81 | 0 | 638 |
| 801 | 581 | 2.564103 | 81 | 0 | 638 |
| 802 | 0 | 1.503759 | 31 | 0 | 869 |
| 802 | 98 | 1.602136 | 31 | 0 | 869 |
| 802 | 147 | 2.446483 | 31 | 0 | 869 |
| 802 | 245 | 1.818182 | 31 | 0 | 869 |
| 802 | 357 | 1.834862 | 31 | 0 | 869 |
| 802 | 413 | 0.869565 | 31 | 0 | 869 |

| | | | | | |
|---|---|---|---|---|---|
| 802 | 525 | 0.909091 | 31 | 0 | 869 |
| 802 | 581 | 1.818182 | 31 | 0 | 869 |
| 803 | 0 | 2.181818 | 87 | 0 | 82 |
| 803 | 25 | 3.738318 | 87 | 0 | 82 |
| 803 | 81 | 2.330097 | 87 | 0 | 82 |
| 804 | 0 | 6.481481 | 55 | 1 | 586 |
| 804 | 35 | 20.79208 | 55 | 1 | 586 |
| 804 | 116 | 11.66667 | 55 | 1 | 586 |
| 804 | 172 | 12.5 | 55 | 1 | 586 |
| 804 | 242 | 16.16162 | 55 | 1 | 586 |
| 804 | 340 | 12.63158 | 55 | 1 | 586 |
| 804 | 424 | 25.26316 | 55 | 1 | 586 |
| 804 | 501 | 11.11111 | 55 | 1 | 586 |
| 804 | 585 | 5.357143 | 55 | 1 | 586 |
| 805 | 0 | 0.91638 | 76 | 0 | 594 |
| 805 | 32 | 0.880088 | 76 | 0 | 594 |
| 805 | 102 | 0.36494 | 76 | 0 | 594 |
| 805 | 176 | 0.342822 | 76 | 0 | 594 |
| 805 | 274 | 1.584607 | 76 | 0 | 594 |
| 805 | 348 | 2.5 | 76 | 0 | 594 |
| 805 | 404 | 1.88383 | 76 | 0 | 594 |
| 805 | 498 | 1.433692 | 76 | 0 | 594 |
| 806 | 0 | 3.225806 | 66 | 0 | 938 |
| 806 | 28 | 3.418803 | 66 | 0 | 938 |
| 806 | 84 | 3.603604 | 66 | 0 | 938 |
| 806 | 168 | 3.448276 | 66 | 0 | 938 |
| 806 | 252 | 3.603604 | 66 | 0 | 938 |
| 806 | 336 | 4.892966 | 66 | 0 | 938 |
| 806 | 399 | 11.53846 | 66 | 0 | 938 |
| 806 | 511 | 5.084746 | 66 | 0 | 938 |
| 806 | 595 | 5.504587 | 66 | 0 | 938 |
| 807 | 0 | 2.681992 | 76 | 0 | 1261 |
| 807 | 27 | 2.631579 | 76 | 0 | 1261 |
| 807 | 83 | 2.803738 | 76 | 0 | 1261 |
| 807 | 139 | 3.773585 | 76 | 0 | 1261 |
| 807 | 216 | 2.654867 | 76 | 0 | 1261 |
| 807 | 314 | 1.754386 | 76 | 0 | 1261 |
| 807 | 412 | 6 | 76 | 0 | 1261 |
| 807 | 497 | 5.217391 | 76 | 0 | 1261 |

| | | | | | |
|---|---|---|---|---|---|
| 808 | 28 | 11.65049 | 75 | 1 | 100 |
| 808 | 56 | 12.5 | 75 | 1 | 100 |
| 809 | 0 | 1.503759 | 86 | 0 | 701 |
| 809 | 77 | 2.142857 | 86 | 0 | 701 |
| 809 | 168 | 2.777778 | 86 | 0 | 701 |
| 809 | 259 | 8.727273 | 86 | 0 | 701 |
| 809 | 350 | 5.172414 | 86 | 0 | 701 |
| 809 | 406 | 4.137931 | 86 | 0 | 701 |
| 809 | 497 | 12.37113 | 86 | 0 | 701 |
| 810 | 0 | 6.315789 | 80 | 0 | 869 |
| 810 | 28 | 6 | 80 | 0 | 869 |
| 810 | 91 | 5.405405 | 80 | 0 | 869 |
| 810 | 173 | 4.137931 | 80 | 0 | 869 |
| 810 | 236 | 5.263158 | 80 | 0 | 869 |
| 810 | 327 | 4.210526 | 80 | 0 | 869 |
| 810 | 397 | 4.137931 | 80 | 0 | 869 |
| 810 | 502 | 4.137931 | 80 | 0 | 869 |
| 810 | 572 | 3.902439 | 80 | 0 | 869 |
| 811 | 0 | 3.686636 | 77 | 0 | 733 |
| 811 | 49 | 5.95428 | 77 | 0 | 733 |
| 811 | 154 | 12.76596 | 77 | 0 | 733 |
| 812 | 0 | 1.834862 | 63 | 0 | 792 |
| 813 | 0 | 6.280374 | 65 | 0 | 977 |
| 813 | 25 | 3.636364 | 65 | 0 | 977 |
| 813 | 95 | 2.608696 | 65 | 0 | 977 |
| 813 | 123 | 1.391304 | 65 | 0 | 977 |
| 813 | 256 | 0.139543 | 65 | 0 | 977 |
| 813 | 333 | 0.143215 | 65 | 0 | 977 |
| 813 | 503 | 0.152584 | 65 | 0 | 977 |
| 813 | 606 | 2.285714 | 65 | 0 | 977 |
| 814 | 0 | 2.068966 | 63 | 0 | 764 |
| 814 | 35 | 2.380952 | 63 | 0 | 764 |
| 814 | 91 | 2.921739 | 63 | 0 | 764 |
| 814 | 140 | 2.419355 | 63 | 0 | 764 |
| 814 | 252 | 2.362205 | 63 | 0 | 764 |
| 814 | 364 | 2.380952 | 63 | 0 | 764 |
| 814 | 420 | 2.5 | 63 | 0 | 764 |
| 814 | 476 | 3.389831 | 63 | 0 | 764 |
| 814 | 588 | 2.4 | 63 | 0 | 764 |

| | | | | | |
|---|---|---|---|---|---|
| 815 | 0 | 2.752294 | 59 | 0 | 981 |
| 815 | 98 | 4.247788 | 59 | 0 | 981 |
| 815 | 182 | 5.084746 | 59 | 0 | 981 |
| 815 | 273 | 2.83353 | 59 | 0 | 981 |
| 815 | 350 | 4.243496 | 59 | 0 | 981 |
| 815 | 427 | 8.112023 | 59 | 0 | 981 |
| 815 | 511 | 10.71429 | 59 | 0 | 981 |
| 815 | 574 | 8.275862 | 59 | 0 | 981 |
| 816 | 0 | 2.654867 | 41 | 1 | 277 |
| 816 | 28 | 2.777778 | 41 | 1 | 277 |
| 816 | 91 | 4.485981 | 41 | 1 | 277 |
| 816 | 154 | 5.555556 | 41 | 1 | 277 |
| 817 | 0 | 17.30769 | 73 | 0 | 956 |
| 817 | 28 | 15.51724 | 73 | 0 | 956 |
| 817 | 87 | 13.01653 | 73 | 0 | 956 |
| 817 | 266 | 14.17323 | 73 | 0 | 956 |
| 817 | 329 | 10 | 73 | 0 | 956 |
| 817 | 448 | 8.571429 | 73 | 0 | 956 |
| 817 | 539 | 8.067227 | 73 | 0 | 956 |
| 817 | 574 | 9.142857 | 73 | 0 | 956 |
| 818 | 0 | 3.669725 | 45 | 1 | 273 |
| 818 | 35 | 5.405405 | 45 | 1 | 273 |
| 818 | 91 | 6.588235 | 45 | 1 | 273 |
| 818 | 147 | 4.948454 | 45 | 1 | 273 |
| 818 | 266 | 17.96791 | 45 | 1 | 273 |
| 819 | 0 | 4.195804 | 82 | 0 | 37 |
| 820 | 0 | 5.660377 | 81 | 1 | 157 |
| 820 | 35 | 2.857143 | 81 | 1 | 157 |
| 820 | 63 | 2.242991 | 81 | 1 | 157 |
| 820 | 145 | 12.63158 | 81 | 1 | 157 |
| 821 | 0 | 5.555556 | 65 | 0 | 995 |
| 821 | 28 | 5.357143 | 65 | 0 | 995 |
| 821 | 84 | 5 | 65 | 0 | 995 |
| 821 | 168 | 5.357143 | 65 | 0 | 995 |
| 821 | 252 | 5.128205 | 65 | 0 | 995 |
| 821 | 337 | 5.263158 | 65 | 0 | 995 |
| 821 | 400 | 5.309735 | 65 | 0 | 995 |
| 821 | 483 | 5.454545 | 65 | 0 | 995 |
| 821 | 714 | 4.444444 | 65 | 0 | 995 |

| | | | | | |
|---|---|---|---|---|---|
| 822 | 0 | 6.185567 | 73 | 1 | 305 |
| 822 | 28 | 4.444444 | 73 | 1 | 305 |
| 822 | 89 | 12.12121 | 73 | 1 | 305 |
| 823 | 0 | 1.37931 | 61 | 1 | 360 |
| 823 | 35 | 1.169591 | 61 | 1 | 360 |
| 823 | 56 | 1.066667 | 61 | 1 | 360 |
| 823 | 133 | 6 | 61 | 1 | 360 |
| 823 | 244 | 13.16614 | 61 | 1 | 360 |
| 823 | 332 | 3.703704 | 61 | 1 | 360 |
| 824 | 0 | 0.560523 | 87 | 0 | 699 |
| 824 | 28 | 0.585325 | 87 | 0 | 699 |
| 824 | 84 | 2.564103 | 87 | 0 | 699 |
| 824 | 175 | 0.795756 | 87 | 0 | 699 |
| 824 | 238 | 0.765306 | 87 | 0 | 699 |
| 824 | 336 | 5.442177 | 87 | 0 | 699 |
| 824 | 510 | 2.4 | 87 | 0 | 699 |
| 824 | 580 | 2.970297 | 87 | 0 | 699 |
| 825 | 0 | 0.636364 | 66 | 1 | 455 |
| 825 | 28 | 0.694215 | 66 | 1 | 455 |
| 825 | 70 | 1.351351 | 66 | 1 | 455 |
| 825 | 168 | 4.247788 | 66 | 1 | 455 |
| 825 | 252 | 4.137931 | 66 | 1 | 455 |
| 826 | 0 | 3.225806 | 74 | 0 | 1065 |
| 826 | 28 | 13.15789 | 74 | 0 | 1065 |
| 826 | 56 | 15.53398 | 74 | 0 | 1065 |
| 826 | 175 | 0.917431 | 74 | 0 | 1065 |
| 826 | 252 | 3.584765 | 74 | 0 | 1065 |
| 826 | 322 | 3.026482 | 74 | 0 | 1065 |
| 826 | 378 | 1.219512 | 74 | 0 | 1065 |
| 826 | 525 | 1.339286 | 74 | 0 | 1065 |
| 826 | 623 | 2.123894 | 74 | 0 | 1065 |
| 827 | 18 | 4.895105 | 67 | 0 | 1341 |
| 827 | 81 | 12.76596 | 67 | 0 | 1341 |
| 827 | 165 | 17.64706 | 67 | 0 | 1341 |
| 827 | 252 | 21.62162 | 67 | 0 | 1341 |
| 827 | 326 | 16.36364 | 67 | 0 | 1341 |
| 827 | 410 | 19.35484 | 67 | 0 | 1341 |
| 827 | 494 | 19.14894 | 67 | 0 | 1341 |
| 827 | 578 | 18.75 | 67 | 0 | 1341 |

| | | | | | |
|---|---|---|---|---|---|
| 828 | 0 | 3.069054 | 53 | 0 | 785 |
| 828 | 63 | 2.142857 | 53 | 0 | 785 |
| 828 | 154 | 3.167421 | 53 | 0 | 785 |
| 828 | 231 | 3.114187 | 53 | 0 | 785 |
| 828 | 322 | 7.5 | 53 | 0 | 785 |
| 828 | 402 | 3.45679 | 53 | 0 | 785 |
| 828 | 500 | 6.779661 | 53 | 0 | 785 |
| 828 | 554 | 14.4 | 53 | 0 | 785 |
| 829 | 0 | 4 | 75 | 1 | 45 |
| 829 | 22 | 4.307692 | 75 | 1 | 45 |
| 830 | 91 | 6.122449 | 48 | 1 | 165 |
| 830 | 154 | 18.3908 | 48 | 1 | 165 |
| 831 | 0 | 10.61947 | 49 | 0 | 701 |
| 831 | 28 | 10.52632 | 49 | 0 | 701 |
| 831 | 91 | 11.88119 | 49 | 0 | 701 |
| 831 | 175 | 10.43478 | 49 | 0 | 701 |
| 831 | 259 | 5.263158 | 49 | 0 | 701 |
| 831 | 350 | 10.90909 | 49 | 0 | 701 |
| 831 | 427 | 9.210526 | 49 | 0 | 701 |
| 831 | 497 | 4.8 | 49 | 0 | 701 |
| 831 | 560 | 8.653846 | 49 | 0 | 701 |
| 832 | 0 | 4.485981 | 69 | 1 | 31 |
| 833 | 0 | 4 | 68 | 0 | 967 |
| 833 | 31 | 4 | 68 | 0 | 967 |
| 833 | 84 | 3.883495 | 68 | 0 | 967 |
| 833 | 168 | 4.363636 | 68 | 0 | 967 |
| 833 | 231 | 3.571429 | 68 | 0 | 967 |
| 833 | 399 | 8.571429 | 68 | 0 | 967 |
| 833 | 490 | 10.16949 | 68 | 0 | 967 |
| 833 | 574 | 10.34483 | 68 | 0 | 967 |
| 834 | 0 | 0.47619 | 53 | 0 | 1049 |
| 834 | 28 | 0.4884 | 53 | 0 | 1049 |
| 834 | 56 | 3 | 53 | 0 | 1049 |
| 835 | 0 | 2.702703 | 68 | 1 | 388 |
| 835 | 28 | 1.666667 | 68 | 1 | 388 |
| 835 | 91 | 2.469136 | 68 | 1 | 388 |
| 835 | 168 | 2.678571 | 68 | 1 | 388 |
| 835 | 258 | 5.147059 | 68 | 1 | 388 |
| 835 | 349 | 6.185567 | 68 | 1 | 388 |

| | | | | | |
|---|---|---|---|---|---|
| 836 | 0 | 8.430913 | 58 | 0 | 820 |
| 836 | 49 | 10 | 58 | 0 | 820 |
| 836 | 91 | 10.34483 | 58 | 0 | 820 |
| 836 | 175 | 11.90083 | 58 | 0 | 820 |
| 836 | 252 | 12.52174 | 58 | 0 | 820 |
| 836 | 329 | 12.41379 | 58 | 0 | 820 |
| 836 | 441 | 12.10084 | 58 | 0 | 820 |
| 836 | 504 | 12.52174 | 58 | 0 | 820 |
| 836 | 568 | 16.09195 | 58 | 0 | 820 |
| 837 | 0 | 6.334842 | 76 | 1 | 347 |
| 837 | 26 | 5.490196 | 76 | 1 | 347 |
| 837 | 84 | 5.714286 | 76 | 1 | 347 |
| 837 | 168 | 9.375 | 76 | 1 | 347 |
| 837 | 252 | 19.78022 | 76 | 1 | 347 |
| 837 | 336 | 56 | 76 | 1 | 347 |
| 838 | 0 | 1.724138 | 69 | 1 | 206 |
| 838 | 28 | 0.661157 | 69 | 1 | 206 |
| 838 | 91 | 0.833333 | 69 | 1 | 206 |
| 838 | 168 | 5.25 | 69 | 1 | 206 |
| 839 | 0 | 5.874126 | 56 | 0 | 393 |
| 839 | 55 | 2.415876 | 56 | 0 | 393 |
| 839 | 182 | 2.033898 | 56 | 0 | 393 |
| 839 | 252 | 2.123894 | 56 | 0 | 393 |
| 840 | 0 | 1.490683 | 68 | 1 | 465 |
| 840 | 119 | 2.136182 | 68 | 1 | 465 |
| 840 | 158 | 2.469136 | 68 | 1 | 465 |
| 840 | 242 | 2.857143 | 68 | 1 | 465 |
| 840 | 340 | 2.580645 | 68 | 1 | 465 |
| 840 | 417 | 4.637681 | 68 | 1 | 465 |
| 841 | 0 | 5.357143 | 81 | 0 | 657 |
| 841 | 61 | 10.27523 | 81 | 0 | 657 |
| 841 | 236 | 2.222222 | 81 | 0 | 657 |
| 841 | 306 | 2.181818 | 81 | 0 | 657 |
| 841 | 439 | 2.666667 | 81 | 0 | 657 |
| 841 | 509 | 4.8 | 81 | 0 | 657 |
| 841 | 586 | 6.990291 | 81 | 0 | 657 |
| 842 | 0 | 12.2449 | 67 | 0 | 831 |
| 842 | 28 | 3.669725 | 67 | 0 | 831 |
| 842 | 70 | 3.960396 | 67 | 0 | 831 |

| | | | | | |
|---|---|---|---|---|---|
| 842 | 140 | 1.371429 | 67 | 0 | 831 |
| 842 | 266 | 3.370787 | 67 | 0 | 831 |
| 842 | 322 | 6.593407 | 67 | 0 | 831 |
| 842 | 406 | 5.555556 | 67 | 0 | 831 |
| 842 | 490 | 6 | 67 | 0 | 831 |
| 842 | 602 | 5.454545 | 67 | 0 | 831 |
| 843 | 0 | 5.714286 | 74 | 0 | 808 |
| 843 | 14 | 7.142857 | 74 | 0 | 808 |
| 843 | 84 | 0.795756 | 74 | 0 | 808 |
| 843 | 163 | 5.660377 | 74 | 0 | 808 |
| 843 | 240 | 0.991736 | 74 | 0 | 808 |
| 843 | 324 | 5.217391 | 74 | 0 | 808 |
| 843 | 415 | 1.639344 | 74 | 0 | 808 |
| 843 | 506 | 2.201835 | 74 | 0 | 808 |
| 843 | 576 | 2.086957 | 74 | 0 | 808 |
| 844 | 0 | 16.16162 | 51 | 0 | 674 |
| 844 | 21 | 8.275862 | 51 | 0 | 674 |
| 844 | 84 | 12.30769 | 51 | 0 | 674 |
| 844 | 154 | 11.6129 | 51 | 0 | 674 |
| 844 | 263 | 11.43635 | 51 | 0 | 674 |
| 844 | 329 | 6.818182 | 51 | 0 | 674 |
| 844 | 420 | 9.6 | 51 | 0 | 674 |
| 844 | 504 | 10.82126 | 51 | 0 | 674 |
| 844 | 617 | 16.51376 | 51 | 0 | 674 |
| 845 | 0 | 5.825243 | 37 | 0 | 1135 |
| 845 | 28 | 11.88119 | 37 | 0 | 1135 |
| 845 | 84 | 8.421053 | 37 | 0 | 1135 |
| 845 | 175 | 10.08403 | 37 | 0 | 1135 |
| 845 | 259 | 4.444444 | 37 | 0 | 1135 |
| 845 | 343 | 5.660377 | 37 | 0 | 1135 |
| 845 | 427 | 11.65049 | 37 | 0 | 1135 |
| 845 | 490 | 14.95327 | 37 | 0 | 1135 |
| 846 | 0 | 6.382979 | 78 | 1 | 416 |
| 846 | 28 | 10.10526 | 78 | 1 | 416 |
| 846 | 91 | 13.63636 | 78 | 1 | 416 |
| 846 | 182 | 21.42857 | 78 | 1 | 416 |
| 846 | 273 | 32.22506 | 78 | 1 | 416 |
| 846 | 371 | 5.150741 | 78 | 1 | 416 |
| 846 | 406 | 4.283529 | 78 | 1 | 416 |

| | | | | | |
|---|---|---|---|---|---|
| 847 | 0 | 8.470588 | 74 | 1 | 72 |
| 847 | 35 | 25.80645 | 74 | 1 | 72 |
| 848 | 0 | 4.173913 | 43 | 1 | 349 |
| 848 | 35 | 3.669725 | 43 | 1 | 349 |
| 848 | 77 | 4.324324 | 43 | 1 | 349 |
| 848 | 182 | 4.033613 | 43 | 1 | 349 |
| 848 | 259 | 17.16914 | 43 | 1 | 349 |
| 848 | 319 | 12.06897 | 43 | 1 | 349 |
| 849 | 0 | 1.441441 | 52 | 0 | 659 |
| 849 | 28 | 1.769912 | 52 | 0 | 659 |
| 849 | 84 | 1.754386 | 52 | 0 | 659 |
| 849 | 168 | 4.081633 | 52 | 0 | 659 |
| 849 | 252 | 6.185567 | 52 | 0 | 659 |
| 849 | 308 | 2.702703 | 52 | 0 | 659 |
| 849 | 420 | 3.498542 | 52 | 0 | 659 |
| 849 | 469 | 2.678571 | 52 | 0 | 659 |
| 849 | 581 | 2.912621 | 52 | 0 | 659 |
| 850 | 0 | 3.106796 | 74 | 1 | 525 |
| 850 | 35 | 6.060606 | 74 | 1 | 525 |
| 850 | 91 | 7.476636 | 74 | 1 | 525 |
| 850 | 175 | 5.042017 | 74 | 1 | 525 |
| 850 | 287 | 3.539823 | 74 | 1 | 525 |
| 850 | 371 | 3.738318 | 74 | 1 | 525 |
| 850 | 455 | 6.451613 | 74 | 1 | 525 |
| 850 | 483 | 27.49591 | 74 | 1 | 525 |
| 851 | 0 | 2.711864 | 71 | 0 | 1072 |
| 851 | 28 | 2.259887 | 71 | 0 | 1072 |
| 851 | 70 | 1.666667 | 71 | 0 | 1072 |
| 851 | 161 | 2.136182 | 71 | 0 | 1072 |
| 851 | 266 | 1.886792 | 71 | 0 | 1072 |
| 851 | 315 | 1.652893 | 71 | 0 | 1072 |
| 851 | 420 | 1.937046 | 71 | 0 | 1072 |
| 851 | 504 | 1.724138 | 71 | 0 | 1072 |
| 851 | 588 | 3.636364 | 71 | 0 | 1072 |
| 852 | 0 | 5.217391 | 65 | 0 | 995 |
| 852 | 28 | 5.042017 | 65 | 0 | 995 |
| 852 | 105 | 3.204272 | 65 | 0 | 995 |
| 852 | 154 | 11.11111 | 65 | 0 | 995 |
| 852 | 217 | 11.21495 | 65 | 0 | 995 |

| | | | | | |
|---|---|---|---|---|---|
| 852 | 302 | 10.25641 | 65 | 0 | 995 |
| 852 | 414 | 11.21495 | 65 | 0 | 995 |
| 852 | 497 | 9.836066 | 65 | 0 | 995 |
| 852 | 553 | 10.08403 | 65 | 0 | 995 |
| 853 | 0 | 0.564972 | 92 | 0 | 598 |
| 853 | 28 | 0.600601 | 92 | 0 | 598 |
| 853 | 84 | 2.970297 | 92 | 0 | 598 |
| 853 | 168 | 0.892857 | 92 | 0 | 598 |
| 853 | 254 | 1.960784 | 92 | 0 | 598 |
| 853 | 338 | 3.478261 | 92 | 0 | 598 |
| 853 | 422 | 0.976801 | 92 | 0 | 598 |
| 853 | 513 | 5.940594 | 92 | 0 | 598 |
| 853 | 597 | 5.042017 | 92 | 0 | 598 |
| 854 | 0 | 3.539823 | 73 | 0 | 715 |
| 854 | 28 | 7.491639 | 73 | 0 | 715 |
| 854 | 84 | 3.361345 | 73 | 0 | 715 |
| 854 | 168 | 3.225806 | 73 | 0 | 715 |
| 854 | 252 | 3.305785 | 73 | 0 | 715 |
| 854 | 343 | 2.737048 | 73 | 0 | 715 |
| 854 | 404 | 2.831858 | 73 | 0 | 715 |
| 854 | 497 | 3.539823 | 73 | 0 | 715 |
| 854 | 588 | 3.703704 | 73 | 0 | 715 |
| 855 | 0 | 19.80198 | 79 | 0 | 729 |
| 855 | 28 | 16.12903 | 79 | 0 | 729 |
| 855 | 84 | 2.302158 | 79 | 0 | 729 |
| 855 | 175 | 5.660377 | 79 | 0 | 729 |
| 855 | 273 | 6.792453 | 79 | 0 | 729 |
| 855 | 364 | 8.928571 | 79 | 0 | 729 |
| 855 | 427 | 9.130435 | 79 | 0 | 729 |
| 855 | 490 | 10.90909 | 79 | 0 | 729 |
| 855 | 581 | 8.275862 | 79 | 0 | 729 |
| 856 | 0 | 0.393185 | 75 | 1 | 289 |
| 856 | 21 | 0.372671 | 75 | 1 | 289 |
| 856 | 63 | 0.396825 | 75 | 1 | 289 |
| 856 | 140 | 4.081633 | 75 | 1 | 289 |
| 856 | 224 | 1.787995 | 75 | 1 | 289 |
| 857 | 0 | 5.555556 | 39 | 1 | 317 |
| 857 | 28 | 2.5 | 39 | 1 | 317 |
| 857 | 84 | 2.857143 | 39 | 1 | 317 |

| | | | | | |
|---|---|---|---|---|---|
| 857 | 161 | 12.5 | 39 | 1 | 317 |
| 857 | 245 | 1.574213 | 39 | 1 | 317 |
| 858 | 0 | 3.478261 | 76 | 0 | 880 |
| 858 | 70 | 5.128205 | 76 | 0 | 880 |
| 859 | 0 | 2.479339 | 63 | 1 | 221 |
| 859 | 28 | 2.5 | 63 | 1 | 221 |
| 859 | 112 | 2.542373 | 63 | 1 | 221 |
| 859 | 175 | 2.568807 | 63 | 1 | 221 |
| 860 | 0 | 6.666667 | 75 | 1 | 74 |
| 860 | 28 | 11.29032 | 75 | 1 | 74 |
| 861 | 0 | 12.2449 | 69 | 0 | 514 |
| 861 | 42 | 11.32075 | 69 | 0 | 514 |
| 861 | 98 | 5.309735 | 69 | 0 | 514 |
| 861 | 182 | 3.508772 | 69 | 0 | 514 |
| 861 | 259 | 7.142857 | 69 | 0 | 514 |
| 861 | 371 | 13.46154 | 69 | 0 | 514 |
| 861 | 427 | 20.86957 | 69 | 0 | 514 |
| 862 | 0 | 6.976744 | 75 | 0 | 140 |
| 862 | 87 | 6.060606 | 75 | 0 | 140 |
| 863 | 0 | 9.813084 | 81 | 0 | 267 |
| 863 | 28 | 5.309735 | 81 | 0 | 267 |
| 863 | 77 | 4.571429 | 81 | 0 | 267 |
| 863 | 168 | 18.75 | 81 | 0 | 267 |
| 863 | 243 | 11.40788 | 81 | 0 | 267 |
| 864 | 0 | 5.263158 | 84 | 0 | 701 |
| 864 | 28 | 5.084746 | 84 | 0 | 701 |
| 864 | 161 | 4.067797 | 84 | 0 | 701 |
| 864 | 266 | 4.247788 | 84 | 0 | 701 |
| 864 | 329 | 4.285714 | 84 | 0 | 701 |
| 864 | 406 | 3.448276 | 84 | 0 | 701 |
| 864 | 490 | 3.603604 | 84 | 0 | 701 |
| 864 | 574 | 3.738318 | 84 | 0 | 701 |
| 865 | 0 | 11.88119 | 83 | 1 | 459 |
| 865 | 28 | 9.142857 | 83 | 1 | 459 |
| 865 | 94 | 10.52302 | 83 | 1 | 459 |
| 865 | 154 | 12.12121 | 83 | 1 | 459 |
| 865 | 238 | 5.454545 | 83 | 1 | 459 |
| 865 | 322 | 4.8 | 83 | 1 | 459 |
| 865 | 420 | 12.2449 | 83 | 1 | 459 |

| | | | | | |
|---|---|---|---|---|---|
| 866 | 0 | 4.912281 | 85 | 1 | 549 |
| 866 | 30 | 5.405405 | 85 | 1 | 549 |
| 866 | 86 | 5.357143 | 85 | 1 | 549 |
| 866 | 170 | 5.172414 | 85 | 1 | 549 |
| 866 | 254 | 6.730769 | 85 | 1 | 549 |
| 866 | 331 | 6 | 85 | 1 | 549 |
| 866 | 443 | 5.405405 | 85 | 1 | 549 |
| 866 | 506 | 5.045045 | 85 | 1 | 549 |
| 867 | 0 | 11.42857 | 71 | 0 | 813 |
| 867 | 28 | 5.882353 | 71 | 0 | 813 |
| 867 | 84 | 10.08403 | 71 | 0 | 813 |
| 867 | 168 | 9.448819 | 71 | 0 | 813 |
| 867 | 252 | 9.677419 | 71 | 0 | 813 |
| 867 | 336 | 9.677419 | 71 | 0 | 813 |
| 867 | 420 | 5.042017 | 71 | 0 | 813 |
| 867 | 504 | 1.666667 | 71 | 0 | 813 |
| 867 | 588 | 1.694915 | 71 | 0 | 813 |
| 868 | 0 | 2.564103 | 60 | 0 | 589 |
| 868 | 28 | 2.608696 | 60 | 0 | 589 |
| 868 | 84 | 2.678571 | 60 | 0 | 589 |
| 868 | 182 | 2.402402 | 60 | 0 | 589 |
| 868 | 259 | 4.705882 | 60 | 0 | 589 |
| 868 | 329 | 5.454545 | 60 | 0 | 589 |
| 868 | 434 | 3.539823 | 60 | 0 | 589 |
| 868 | 497 | 10.71429 | 60 | 0 | 589 |
| 868 | 588 | 4.6875 | 60 | 0 | 589 |
| 869 | 0 | 1.709402 | 84 | 0 | 818 |
| 869 | 28 | 2.941176 | 84 | 0 | 818 |
| 869 | 84 | 0.943396 | 84 | 0 | 818 |
| 869 | 169 | 3.98293 | 84 | 0 | 818 |
| 869 | 264 | 0.862069 | 84 | 0 | 818 |
| 869 | 320 | 2.752294 | 84 | 0 | 818 |
| 869 | 404 | 5.769231 | 84 | 0 | 818 |
| 869 | 509 | 4.285714 | 84 | 0 | 818 |
| 869 | 600 | 5.714286 | 84 | 0 | 818 |
| 870 | 0 | 11.63274 | 83 | 0 | 953 |
| 870 | 28 | 11.56627 | 83 | 0 | 953 |
| 870 | 91 | 19.78022 | 83 | 0 | 953 |
| 870 | 175 | 19.56522 | 83 | 0 | 953 |

| | | | | | |
|---|---|---|---|---|---|
| 870 | 252 | 16.55172 | 83 | 0 | 953 |
| 870 | 343 | 21.42857 | 83 | 0 | 953 |
| 870 | 434 | 16 | 83 | 0 | 953 |
| 870 | 525 | 18 | 83 | 0 | 953 |
| 870 | 588 | 17.82178 | 83 | 0 | 953 |
| 871 | 0 | 5 | 75 | 1 | 326 |
| 871 | 28 | 1.612903 | 75 | 1 | 326 |
| 871 | 84 | 1.652893 | 75 | 1 | 326 |
| 871 | 161 | 1.694915 | 75 | 1 | 326 |
| 871 | 252 | 1.666667 | 75 | 1 | 326 |
| 872 | 0 | 1.304348 | 83 | 0 | 778 |
| 872 | 28 | 0.745342 | 83 | 0 | 778 |
| 872 | 77 | 0.4662 | 83 | 0 | 778 |
| 872 | 154 | 0.45045 | 83 | 0 | 778 |
| 872 | 238 | 0.617284 | 83 | 0 | 778 |
| 872 | 322 | 0.600601 | 83 | 0 | 778 |
| 873 | 0 | 8.602151 | 79 | 1 | 102 |
| 873 | 21 | 6.25 | 79 | 1 | 102 |
| 874 | 0 | 5.172414 | 73 | 0 | 806 |
| 874 | 28 | 5.454545 | 73 | 0 | 806 |
| 874 | 84 | 12 | 73 | 0 | 806 |
| 874 | 168 | 9.320388 | 73 | 0 | 806 |
| 874 | 259 | 14.45783 | 73 | 0 | 806 |
| 874 | 343 | 21.68675 | 73 | 0 | 806 |
| 874 | 427 | 20.93023 | 73 | 0 | 806 |
| 874 | 511 | 4.878049 | 73 | 0 | 806 |
| 874 | 595 | 5 | 73 | 0 | 806 |
| 875 | 0 | 5.882353 | 65 | 1 | 382 |
| 875 | 28 | 2.857143 | 65 | 1 | 382 |
| 875 | 84 | 4.948454 | 65 | 1 | 382 |
| 875 | 182 | 5.882353 | 65 | 1 | 382 |
| 875 | 266 | 5.714286 | 65 | 1 | 382 |
| 875 | 333 | 5.30303 | 65 | 1 | 382 |
| 876 | 0 | 11.21495 | 63 | 1 | 115 |
| 876 | 28 | 16.98113 | 63 | 1 | 115 |
| 876 | 86 | 27.45098 | 63 | 1 | 115 |
| 877 | 0 | 2.242991 | 79 | 0 | 850 |
| 877 | 70 | 2.431373 | 79 | 0 | 850 |
| 877 | 175 | 2.402402 | 79 | 0 | 850 |

| | | | | | |
|---|---|---|---|---|---|
| 877 | 266 | 2.298851 | 79 | 0 | 850 |
| 877 | 322 | 3.53461 | 79 | 0 | 850 |
| 877 | 399 | 1.693719 | 79 | 0 | 850 |
| 877 | 490 | 1.52381 | 79 | 0 | 850 |
| 877 | 595 | 1.568627 | 79 | 0 | 850 |
| 878 | 0 | 2.439024 | 73 | 0 | 631 |
| 878 | 28 | 2.068966 | 73 | 0 | 631 |
| 878 | 91 | 2.608696 | 73 | 0 | 631 |
| 878 | 154 | 2.142857 | 73 | 0 | 631 |
| 878 | 252 | 2.242991 | 73 | 0 | 631 |
| 878 | 322 | 2.285714 | 73 | 0 | 631 |
| 878 | 420 | 2.727273 | 73 | 0 | 631 |
| 878 | 497 | 2.941176 | 73 | 0 | 631 |
| 878 | 560 | 2.352941 | 73 | 0 | 631 |
| 879 | 0 | 4.804805 | 77 | 0 | 834 |
| 879 | 63 | 3.827751 | 77 | 0 | 834 |
| 879 | 140 | 5.504587 | 77 | 0 | 834 |
| 879 | 224 | 6.382979 | 77 | 0 | 834 |
| 879 | 308 | 15.48387 | 77 | 0 | 834 |
| 879 | 441 | 17.47573 | 77 | 0 | 834 |
| 879 | 476 | 16.82243 | 77 | 0 | 834 |
| 879 | 560 | 18 | 77 | 0 | 834 |
| 880 | 0 | 5.405405 | 90 | 0 | 421 |
| 880 | 28 | 5.405405 | 90 | 0 | 421 |
| 880 | 84 | 2.453271 | 90 | 0 | 421 |
| 880 | 161 | 4.123711 | 90 | 0 | 421 |
| 880 | 252 | 3.542062 | 90 | 0 | 421 |
| 880 | 329 | 29.26829 | 90 | 0 | 421 |
| 880 | 415 | 11.03448 | 90 | 0 | 421 |
| 881 | 0 | 2.608696 | 77 | 1 | 344 |
| 881 | 28 | 1.260504 | 77 | 1 | 344 |
| 881 | 84 | 1.320132 | 77 | 1 | 344 |
| 881 | 168 | 1.923077 | 77 | 1 | 344 |
| 881 | 252 | 10.82474 | 77 | 1 | 344 |
| 881 | 329 | 12.04301 | 77 | 1 | 344 |
| 882 | 0 | 5.405405 | 68 | 1 | 562 |
| 882 | 28 | 5.309735 | 68 | 1 | 562 |
| 882 | 84 | 5.357143 | 68 | 1 | 562 |
| 882 | 168 | 5.042017 | 68 | 1 | 562 |

| | | | | | |
|---|---|---|---|---|---|
| 882 | 240 | 10.46512 | 68 | 1 | 562 |
| 882 | 324 | 13.18681 | 68 | 1 | 562 |
| 882 | 408 | 12.12121 | 68 | 1 | 562 |
| 882 | 499 | 10.46729 | 68 | 1 | 562 |
| 882 | 559 | 10.81081 | 68 | 1 | 562 |
| 883 | 0 | 6.122449 | 80 | 1 | 309 |
| 883 | 28 | 5.825243 | 80 | 1 | 309 |
| 883 | 56 | 0.521512 | 80 | 1 | 309 |
| 883 | 147 | 0.575126 | 80 | 1 | 309 |
| 884 | 0 | 2.264151 | 81 | 1 | 240 |
| 884 | 77 | 8.791209 | 81 | 1 | 240 |
| 884 | 161 | 5.940594 | 81 | 1 | 240 |
| 884 | 238 | 11.11111 | 81 | 1 | 240 |
| 885 | 0 | 2.608696 | 70 | 0 | 706 |
| 885 | 28 | 0.594228 | 70 | 0 | 706 |
| 885 | 89 | 0.639075 | 70 | 0 | 706 |
| 885 | 152 | 1.351351 | 70 | 0 | 706 |
| 885 | 264 | 2.020202 | 70 | 0 | 706 |
| 885 | 341 | 1.785714 | 70 | 0 | 706 |
| 885 | 425 | 1.801802 | 70 | 0 | 706 |
| 886 | 0 | 2.564103 | 65 | 1 | 369 |
| 886 | 28 | 2.564103 | 65 | 1 | 369 |
| 886 | 84 | 6.792453 | 65 | 1 | 369 |
| 886 | 154 | 6.068268 | 65 | 1 | 369 |
| 886 | 252 | 17.14286 | 65 | 1 | 369 |
| 886 | 329 | 15.9292 | 65 | 1 | 369 |
| 887 | 0 | 2.419355 | 90 | 0 | 597 |
| 887 | 28 | 1.229508 | 90 | 0 | 597 |
| 887 | 84 | 1.212121 | 90 | 0 | 597 |
| 887 | 168 | 1.129944 | 90 | 0 | 597 |
| 887 | 252 | 1.923077 | 90 | 0 | 597 |
| 887 | 336 | 1.818182 | 90 | 0 | 597 |
| 887 | 420 | 3.921569 | 90 | 0 | 597 |
| 887 | 504 | 3.508772 | 90 | 0 | 597 |
| 887 | 588 | 5.940594 | 90 | 0 | 597 |
| 888 | 0 | 2.34375 | 81 | 0 | 755 |
| 888 | 28 | 2.663707 | 81 | 0 | 755 |
| 888 | 81 | 1.724138 | 81 | 0 | 755 |
| 888 | 168 | 1.785714 | 81 | 0 | 755 |

| | | | | | |
|---|---|---|---|---|---|
| 888 | 252 | 5.607477 | 81 | 0 | 755 |
| 888 | 336 | 1.257862 | 81 | 0 | 755 |
| 888 | 413 | 1.509705 | 81 | 0 | 755 |
| 888 | 497 | 4.868154 | 81 | 0 | 755 |
| 888 | 581 | 4.247788 | 81 | 0 | 755 |
| 889 | 0 | 2.238806 | 75 | 1 | 565 |
| 889 | 28 | 2.380952 | 75 | 1 | 565 |
| 889 | 84 | 2.123894 | 75 | 1 | 565 |
| 889 | 175 | 3.076923 | 75 | 1 | 565 |
| 889 | 252 | 2.086957 | 75 | 1 | 565 |
| 889 | 350 | 3.296703 | 75 | 1 | 565 |
| 889 | 413 | 4.660194 | 75 | 1 | 565 |
| 889 | 504 | 13.7931 | 75 | 1 | 565 |
| 890 | 0 | 2.285714 | 77 | 1 | 813 |
| 890 | 35 | 3.636364 | 77 | 1 | 813 |
| 890 | 63 | 6 | 77 | 1 | 813 |
| 890 | 154 | 4.324324 | 77 | 1 | 813 |
| 890 | 266 | 6.185567 | 77 | 1 | 813 |
| 890 | 350 | 4.848485 | 77 | 1 | 813 |
| 890 | 410 | 16.49485 | 77 | 1 | 813 |
| 890 | 497 | 10.81081 | 77 | 1 | 813 |
| 890 | 581 | 12.76596 | 77 | 1 | 813 |
| 891 | 0 | 3.636364 | 72 | 1 | 460 |
| 891 | 28 | 8.080808 | 72 | 1 | 460 |
| 891 | 84 | 10.58824 | 72 | 1 | 460 |
| 891 | 168 | 9.782609 | 72 | 1 | 460 |
| 891 | 231 | 15.18987 | 72 | 1 | 460 |
| 891 | 343 | 14.11765 | 72 | 1 | 460 |
| 891 | 396 | 28.91566 | 72 | 1 | 460 |
| 892 | 0 | 2.752294 | 43 | 0 | 841 |
| 892 | 28 | 2.752294 | 43 | 0 | 841 |
| 892 | 56 | 2.727273 | 43 | 0 | 841 |
| 892 | 168 | 2.678571 | 43 | 0 | 841 |
| 892 | 259 | 2.777778 | 43 | 0 | 841 |
| 892 | 350 | 2.105263 | 43 | 0 | 841 |
| 892 | 378 | 2.631579 | 43 | 0 | 841 |
| 892 | 469 | 2.803738 | 43 | 0 | 841 |
| 892 | 616 | 3.33069 | 43 | 0 | 841 |
| 893 | 0 | 2.020202 | 84 | 0 | 159 |

| | | | | | |
|---|---|---|---|---|---|
| 893 | 28 | 2.298851 | 84 | 0 | 159 |
| 893 | 84 | 11.42857 | 84 | 0 | 159 |
| 894 | 0 | 2.105263 | 68 | 0 | 680 |
| 894 | 70 | 3.168317 | 68 | 0 | 680 |
| 894 | 168 | 5.274725 | 68 | 0 | 680 |
| 894 | 238 | 9.896907 | 68 | 0 | 680 |
| 894 | 336 | 8.888889 | 68 | 0 | 680 |
| 894 | 406 | 4.210526 | 68 | 0 | 680 |
| 894 | 511 | 4.444444 | 68 | 0 | 680 |
| 895 | 0 | 4.878049 | 84 | 0 | 1227 |
| 895 | 28 | 4.210526 | 84 | 0 | 1227 |
| 895 | 91 | 5 | 84 | 0 | 1227 |
| 895 | 166 | 1.904762 | 84 | 0 | 1227 |
| 895 | 245 | 2.803738 | 84 | 0 | 1227 |
| 895 | 329 | 2.631579 | 84 | 0 | 1227 |
| 895 | 414 | 2.479339 | 84 | 0 | 1227 |
| 895 | 498 | 2.702703 | 84 | 0 | 1227 |
| 895 | 589 | 1.388889 | 84 | 0 | 1227 |
| 896 | 0 | 4.724409 | 63 | 0 | 905 |
| 896 | 28 | 2.238806 | 63 | 0 | 905 |
| 896 | 84 | 1.587302 | 63 | 0 | 905 |
| 896 | 168 | 1.550388 | 63 | 0 | 905 |
| 896 | 252 | 0.847458 | 63 | 0 | 905 |
| 896 | 336 | 0.787402 | 63 | 0 | 905 |
| 896 | 420 | 0.819672 | 63 | 0 | 905 |
| 896 | 504 | 0.826446 | 63 | 0 | 905 |
| 896 | 586 | 0.828402 | 63 | 0 | 905 |
| 897 | 0 | 1.648352 | 52 | 1 | 242 |
| 897 | 98 | 1.680672 | 52 | 1 | 242 |
| 897 | 147 | 2.970297 | 52 | 1 | 242 |
| 898 | 0 | 6.315789 | 84 | 0 | 409 |
| 898 | 28 | 5.194805 | 84 | 0 | 409 |
| 898 | 70 | 5.137615 | 84 | 0 | 409 |
| 898 | 154 | 6.593407 | 84 | 0 | 409 |
| 899 | 0 | 1.477833 | 79 | 0 | 689 |
| 899 | 86 | 2.912621 | 79 | 0 | 689 |
| 899 | 184 | 1.980198 | 79 | 0 | 689 |
| 899 | 226 | 4.301075 | 79 | 0 | 689 |
| 899 | 310 | 3.960396 | 79 | 0 | 689 |

| | | | | | |
|---|---|---|---|---|---|
| 899 | 415 | 7.058824 | 79 | 0 | 689 |
| 899 | 506 | 22.78481 | 79 | 0 | 689 |
| 899 | 590 | 21.95122 | 79 | 0 | 689 |
| 900 | 0 | 5.405405 | 73 | 0 | 1254 |
| 900 | 28 | 6.122449 | 73 | 0 | 1254 |
| 900 | 91 | 10 | 73 | 0 | 1254 |
| 900 | 161 | 11.42857 | 73 | 0 | 1254 |
| 900 | 256 | 9.50764 | 73 | 0 | 1254 |
| 900 | 322 | 8.727273 | 73 | 0 | 1254 |
| 900 | 392 | 8.807339 | 73 | 0 | 1254 |
| 900 | 490 | 12.12121 | 73 | 0 | 1254 |
| 900 | 553 | 8.571429 | 73 | 0 | 1254 |
| 901 | 0 | 1.869159 | 85 | 0 | 865 |
| 901 | 84 | 1.851852 | 85 | 0 | 865 |
| 901 | 140 | 1.904762 | 85 | 0 | 865 |
| 901 | 224 | 2.521008 | 85 | 0 | 865 |
| 901 | 315 | 2.909091 | 85 | 0 | 865 |
| 901 | 406 | 5.504587 | 85 | 0 | 865 |
| 901 | 490 | 4.067797 | 85 | 0 | 865 |
| 901 | 588 | 11.21495 | 85 | 0 | 865 |
| 902 | 0 | 2.201258 | 61 | 0 | 692 |
| 902 | 36 | 1.388889 | 61 | 0 | 692 |
| 902 | 92 | 1.388889 | 61 | 0 | 692 |
| 902 | 148 | 1.234568 | 61 | 0 | 692 |
| 902 | 273 | 1.257862 | 61 | 0 | 692 |
| 902 | 337 | 1.257862 | 61 | 0 | 692 |
| 902 | 400 | 1.282051 | 61 | 0 | 692 |
| 902 | 519 | 1.376147 | 61 | 0 | 692 |
| 902 | 575 | 1.485149 | 61 | 0 | 692 |
| 903 | 0 | 1.5625 | 69 | 0 | 627 |
| 903 | 98 | 1.941748 | 69 | 0 | 627 |
| 903 | 161 | 2.4 | 69 | 0 | 627 |
| 903 | 252 | 6 | 69 | 0 | 627 |
| 903 | 336 | 5.882353 | 69 | 0 | 627 |
| 903 | 427 | 6.593407 | 69 | 0 | 627 |
| 903 | 511 | 14.28571 | 69 | 0 | 627 |
| 903 | 595 | 10.2252 | 69 | 0 | 627 |
| 904 | 0 | 1.680672 | 70 | 1 | 147 |
| 904 | 70 | 5.052632 | 70 | 1 | 147 |

| | | | | | |
|---|---|---|---|---|---|
| 905 | 0 | 5.042017 | 79 | 0 | 647 |
| 905 | 28 | 4.137931 | 79 | 0 | 647 |
| 905 | 84 | 5.357143 | 79 | 0 | 647 |
| 905 | 168 | 5.263158 | 79 | 0 | 647 |
| 905 | 252 | 5.042017 | 79 | 0 | 647 |
| 905 | 334 | 4.958678 | 79 | 0 | 647 |
| 905 | 427 | 7.54717 | 79 | 0 | 647 |
| 905 | 504 | 5.940594 | 79 | 0 | 647 |
| 905 | 580 | 8.034433 | 79 | 0 | 647 |
| 906 | 0 | 7.76699 | 85 | 0 | 517 |
| 906 | 21 | 5.357143 | 85 | 0 | 517 |
| 906 | 77 | 11.53846 | 85 | 0 | 517 |
| 906 | 168 | 10.43478 | 85 | 0 | 517 |
| 906 | 252 | 11.32075 | 85 | 0 | 517 |
| 907 | 0 | 1.485149 | 44 | 0 | 1175 |
| 907 | 56 | 3.125 | 44 | 0 | 1175 |
| 907 | 140 | 2.648172 | 44 | 0 | 1175 |
| 907 | 236 | 2.181818 | 44 | 0 | 1175 |
| 907 | 341 | 1.339286 | 44 | 0 | 1175 |
| 907 | 425 | 1.834862 | 44 | 0 | 1175 |
| 908 | 0 | 0.462963 | 76 | 0 | 733 |
| 908 | 31 | 0.462963 | 76 | 0 | 733 |
| 908 | 168 | 0.952381 | 76 | 0 | 733 |
| 908 | 231 | 0.249896 | 76 | 0 | 733 |
| 908 | 336 | 0.247372 | 76 | 0 | 733 |
| 908 | 413 | 0.284765 | 76 | 0 | 733 |
| 908 | 490 | 0.266193 | 76 | 0 | 733 |
| 908 | 574 | 13.48315 | 76 | 0 | 733 |
| 909 | 0 | 4.040404 | 69 | 0 | 1097 |
| 909 | 42 | 8.421053 | 69 | 0 | 1097 |
| 909 | 140 | 7.843137 | 69 | 0 | 1097 |
| 909 | 210 | 17.02128 | 69 | 0 | 1097 |
| 909 | 357 | 11.11111 | 69 | 0 | 1097 |
| 909 | 413 | 12 | 69 | 0 | 1097 |
| 909 | 483 | 12.12121 | 69 | 0 | 1097 |
| 909 | 581 | 9.320388 | 69 | 0 | 1097 |
| 910 | 0 | 4.8 | 77 | 0 | 1139 |
| 910 | 28 | 5.940594 | 77 | 0 | 1139 |
| 910 | 84 | 5.769231 | 77 | 0 | 1139 |

| | | | | | |
|---|---|---|---|---|---|
| 911 | 0 | 6.741573 | 84 | 1 | 213 |
| 911 | 28 | 13.63636 | 84 | 1 | 213 |
| 911 | 84 | 20.93023 | 84 | 1 | 213 |
| 911 | 168 | 19.35484 | 84 | 1 | 213 |
| 912 | 0 | 2.803738 | 73 | 0 | 1003 |
| 912 | 56 | 2.830189 | 73 | 0 | 1003 |
| 912 | 168 | 2.777778 | 73 | 0 | 1003 |
| 912 | 224 | 2.727273 | 73 | 0 | 1003 |
| 912 | 336 | 2.803738 | 73 | 0 | 1003 |
| 912 | 448 | 2.912621 | 73 | 0 | 1003 |
| 912 | 511 | 2.941176 | 73 | 0 | 1003 |
| 912 | 567 | 2.941176 | 73 | 0 | 1003 |
| 913 | 0 | 11.11111 | 73 | 0 | 580 |
| 913 | 28 | 16 | 73 | 0 | 580 |
| 913 | 91 | 13.98058 | 73 | 0 | 580 |
| 913 | 196 | 10.52632 | 73 | 0 | 580 |
| 913 | 252 | 13.33333 | 73 | 0 | 580 |
| 914 | 0 | 13.30693 | 48 | 1 | 24 |
| 915 | 0 | 0.322581 | 72 | 0 | 756 |
| 915 | 77 | 0.345622 | 72 | 0 | 756 |
| 915 | 189 | 0.358423 | 72 | 0 | 756 |
| 915 | 231 | 0.934579 | 72 | 0 | 756 |
| 915 | 315 | 1.904762 | 72 | 0 | 756 |
| 915 | 392 | 2.201835 | 72 | 0 | 756 |
| 915 | 518 | 1.680672 | 72 | 0 | 756 |
| 915 | 567 | 3.394625 | 72 | 0 | 756 |
| 916 | 0 | 1.239669 | 71 | 1 | 618 |
| 916 | 28 | 1.260504 | 71 | 1 | 618 |
| 916 | 84 | 1.327434 | 71 | 1 | 618 |
| 916 | 168 | 2.857143 | 71 | 1 | 618 |
| 916 | 252 | 3.703704 | 71 | 1 | 618 |
| 916 | 336 | 2.424242 | 71 | 1 | 618 |
| 916 | 406 | 5.769231 | 71 | 1 | 618 |
| 916 | 490 | 5.714286 | 71 | 1 | 618 |
| 916 | 582 | 4.848485 | 71 | 1 | 618 |
| 917 | 0 | 1.95122 | 62 | 1 | 371 |
| 917 | 35 | 1.293103 | 62 | 1 | 371 |
| 917 | 91 | 1.194922 | 62 | 1 | 371 |
| 917 | 182 | 1.243201 | 62 | 1 | 371 |

| | | | | | |
|---|---|---|---|---|---|
| 917 | 273 | 8.888889 | 62 | 1 | 371 |
| 917 | 350 | 6.530612 | 62 | 1 | 371 |
| 918 | 0 | 2.939633 | 92 | 0 | 731 |
| 918 | 30 | 3.305785 | 92 | 0 | 731 |
| 918 | 86 | 3.305785 | 92 | 0 | 731 |
| 918 | 198 | 3.448276 | 92 | 0 | 731 |
| 918 | 254 | 3.603604 | 92 | 0 | 731 |
| 918 | 338 | 4.807692 | 92 | 0 | 731 |
| 918 | 422 | 5.357143 | 92 | 0 | 731 |
| 918 | 506 | 7.758621 | 92 | 0 | 731 |
| 918 | 590 | 8.571429 | 92 | 0 | 731 |
| 919 | 0 | 0.623053 | 73 | 1 | 1233 |
| 919 | 30 | 0.720721 | 73 | 1 | 1233 |
| 919 | 93 | 0.917431 | 73 | 1 | 1233 |
| 919 | 191 | 0.733945 | 73 | 1 | 1233 |
| 919 | 275 | 0.884956 | 73 | 1 | 1233 |
| 919 | 317 | 1.680672 | 73 | 1 | 1233 |
| 919 | 401 | 1.440576 | 73 | 1 | 1233 |
| 919 | 541 | 1.801802 | 73 | 1 | 1233 |
| 919 | 583 | 1.801802 | 73 | 1 | 1233 |
| 920 | 0 | 4.067797 | 77 | 1 | 121 |
| 920 | 28 | 4.285714 | 77 | 1 | 121 |
| 920 | 63 | 6.185567 | 77 | 1 | 121 |
| 921 | 0 | 6.976744 | 67 | 1 | 134 |
| 921 | 14 | 6.451613 | 67 | 1 | 134 |
| 921 | 70 | 1.94332 | 67 | 1 | 134 |
| 922 | 0 | 1.92 | 54 | 0 | 645 |
| 922 | 35 | 1.260504 | 54 | 0 | 645 |
| 922 | 91 | 2.721088 | 54 | 0 | 645 |
| 922 | 154 | 5.825243 | 54 | 0 | 645 |
| 922 | 252 | 6.153846 | 54 | 0 | 645 |
| 922 | 336 | 3.448276 | 54 | 0 | 645 |
| 922 | 399 | 5.079365 | 54 | 0 | 645 |
| 922 | 518 | 4.804805 | 54 | 0 | 645 |
| 922 | 581 | 4.984424 | 54 | 0 | 645 |
| 923 | 0 | 20.86957 | 81 | 0 | 911 |
| 923 | 21 | 6.5625 | 81 | 0 | 911 |
| 923 | 101 | 6.847826 | 81 | 0 | 911 |
| 923 | 196 | 4.736842 | 81 | 0 | 911 |

| | | | | | |
|---|---|---|---|---|---|
| 923 | 245 | 5.429864 | 81 | 0 | 911 |
| 923 | 336 | 4.639175 | 81 | 0 | 911 |
| 923 | 406 | 4.591837 | 81 | 0 | 911 |
| 923 | 490 | 4.8583 | 81 | 0 | 911 |
| 923 | 581 | 4.539723 | 81 | 0 | 911 |
| 924 | 0 | 2.884615 | 78 | 0 | 836 |
| 924 | 63 | 1.442308 | 78 | 0 | 836 |
| 924 | 161 | 5.714286 | 78 | 0 | 836 |
| 924 | 245 | 4.848485 | 78 | 0 | 836 |
| 924 | 345 | 8.331267 | 78 | 0 | 836 |
| 924 | 415 | 17.50912 | 78 | 0 | 836 |
| 924 | 527 | 9.911504 | 78 | 0 | 836 |
| 924 | 590 | 5.031447 | 78 | 0 | 836 |
| 925 | 0 | 1.061947 | 62 | 0 | 645 |
| 925 | 28 | 2.201835 | 62 | 0 | 645 |
| 925 | 91 | 5.607477 | 62 | 0 | 645 |
| 925 | 175 | 4.285714 | 62 | 0 | 645 |
| 925 | 268 | 5.660377 | 62 | 0 | 645 |
| 925 | 324 | 5.882353 | 62 | 0 | 645 |
| 925 | 408 | 12 | 62 | 0 | 645 |
| 925 | 499 | 12 | 62 | 0 | 645 |
| 925 | 583 | 12.12121 | 62 | 0 | 645 |
| 926 | 0 | 0.967742 | 62 | 1 | 658 |
| 926 | 35 | 2.298851 | 62 | 1 | 658 |
| 926 | 84 | 2.752294 | 62 | 1 | 658 |
| 926 | 168 | 1.694915 | 62 | 1 | 658 |
| 926 | 252 | 2.727273 | 62 | 1 | 658 |
| 927 | 0 | 1.139601 | 52 | 0 | 1016 |
| 927 | 28 | 1.092896 | 52 | 0 | 1016 |
| 927 | 91 | 4.705882 | 52 | 0 | 1016 |
| 927 | 161 | 2.051282 | 52 | 0 | 1016 |
| 927 | 259 | 5.084746 | 52 | 0 | 1016 |
| 927 | 350 | 5.128205 | 52 | 0 | 1016 |
| 927 | 413 | 5.882353 | 52 | 0 | 1016 |
| 927 | 497 | 4.247788 | 52 | 0 | 1016 |
| 927 | 588 | 3.030303 | 52 | 0 | 1016 |
| 928 | 0 | 2.539683 | 63 | 1 | 404 |
| 928 | 21 | 5.454545 | 63 | 1 | 404 |
| 928 | 77 | 8.991169 | 63 | 1 | 404 |

| | | | | | |
|---|---|---|---|---|---|
| 928 | 177 | 5.714286 | 63 | 1 | 404 |
| 928 | 240 | 5.940594 | 63 | 1 | 404 |
| 928 | 336 | 11.34113 | 63 | 1 | 404 |
| 929 | 0 | 4.615385 | 47 | 0 | 953 |
| 929 | 28 | 4.897959 | 47 | 0 | 953 |
| 929 | 105 | 8.602151 | 47 | 0 | 953 |
| 930 | 0 | 2.803738 | 82 | 0 | 476 |
| 930 | 28 | 2.830189 | 82 | 0 | 476 |
| 930 | 87 | 2.839757 | 82 | 0 | 476 |
| 930 | 175 | 2.752294 | 82 | 0 | 476 |
| 930 | 266 | 5.940594 | 82 | 0 | 476 |
| 930 | 329 | 2.752294 | 82 | 0 | 476 |
| 930 | 420 | 5.313093 | 82 | 0 | 476 |
| 931 | 0 | 23.07692 | 80 | 1 | 498 |
| 931 | 49 | 2.676296 | 80 | 1 | 498 |
| 931 | 175 | 2.701544 | 80 | 1 | 498 |
| 931 | 255 | 14.7541 | 80 | 1 | 498 |
| 931 | 343 | 28.8 | 80 | 1 | 498 |
| 931 | 427 | 6.086957 | 80 | 1 | 498 |
| 932 | 0 | 3.448276 | 68 | 1 | 654 |
| 932 | 28 | 3.448276 | 68 | 1 | 654 |
| 932 | 49 | 11.42857 | 68 | 1 | 654 |
| 932 | 140 | 4.878049 | 68 | 1 | 654 |
| 932 | 259 | 3.174603 | 68 | 1 | 654 |
| 932 | 308 | 5.128205 | 68 | 1 | 654 |
| 932 | 421 | 10.43478 | 68 | 1 | 654 |
| 932 | 526 | 6.896552 | 68 | 1 | 654 |
| 932 | 568 | 9.160305 | 68 | 1 | 654 |
| 933 | 0 | 2.5 | 70 | 1 | 733 |
| 933 | 28 | 10.90909 | 70 | 1 | 733 |
| 933 | 84 | 8.205128 | 70 | 1 | 733 |
| 933 | 182 | 2.608696 | 70 | 1 | 733 |
| 933 | 245 | 10.81081 | 70 | 1 | 733 |
| 933 | 336 | 18.94737 | 70 | 1 | 733 |
| 933 | 402 | 3.921569 | 70 | 1 | 733 |
| 933 | 493 | 3.478261 | 70 | 1 | 733 |
| 933 | 593 | 9.160305 | 70 | 1 | 733 |
| 934 | 0 | 2.307692 | 60 | 1 | 813 |
| 934 | 35 | 2.586207 | 60 | 1 | 813 |

| | | | | | |
|---|---|---|---|---|---|
| 934 | 91 | 2.033898 | 60 | 1 | 813 |
| 934 | 189 | 2.242991 | 60 | 1 | 813 |
| 934 | 256 | 2.376238 | 60 | 1 | 813 |
| 934 | 326 | 4.848485 | 60 | 1 | 813 |
| 934 | 438 | 3.960396 | 60 | 1 | 813 |
| 934 | 522 | 4.040404 | 60 | 1 | 813 |
| 934 | 599 | 4.705882 | 60 | 1 | 813 |
| 935 | 0 | 4.301075 | 58 | 1 | 453 |
| 935 | 40 | 12.2449 | 58 | 1 | 453 |
| 935 | 182 | 9.320388 | 58 | 1 | 453 |
| 935 | 238 | 15.53398 | 58 | 1 | 453 |
| 935 | 308 | 14.54545 | 58 | 1 | 453 |
| 935 | 441 | 25 | 58 | 1 | 453 |
| 936 | 0 | 2.803738 | 73 | 0 | 93 |
| 936 | 28 | 2.912621 | 73 | 0 | 93 |
| 937 | 0 | 8.510638 | 79 | 1 | 28 |
| 937 | 21 | 13.33333 | 79 | 1 | 28 |
| 938 | 0 | 4.247788 | 81 | 1 | 607 |
| 938 | 101 | 5.6 | 81 | 1 | 607 |
| 938 | 133 | 5.825243 | 81 | 1 | 607 |
| 938 | 227 | 4.444444 | 81 | 1 | 607 |
| 938 | 290 | 8.495575 | 81 | 1 | 607 |
| 938 | 395 | 9.142857 | 81 | 1 | 607 |
| 938 | 465 | 10.98039 | 81 | 1 | 607 |
| 938 | 563 | 9.896907 | 81 | 1 | 607 |
| 939 | 0 | 3.703704 | 88 | 0 | 1149 |
| 939 | 28 | 1.754386 | 88 | 0 | 1149 |
| 939 | 84 | 1.904762 | 88 | 0 | 1149 |
| 939 | 168 | 4.123711 | 88 | 0 | 1149 |
| 939 | 252 | 3.539823 | 88 | 0 | 1149 |
| 939 | 336 | 3.603604 | 88 | 0 | 1149 |
| 939 | 420 | 6.25 | 88 | 0 | 1149 |
| 939 | 504 | 6.185567 | 88 | 0 | 1149 |
| 939 | 588 | 6.185567 | 88 | 0 | 1149 |
| 940 | 0 | 1.02916 | 76 | 1 | 286 |
| 940 | 77 | 6.122449 | 76 | 1 | 286 |
| 940 | 182 | 7.142857 | 76 | 1 | 286 |
| 940 | 227 | 6 | 76 | 1 | 286 |
| 941 | 0 | 2.654867 | 72 | 0 | 239 |

| | | | | | |
|---|---|---|---|---|---|
| 941 | 28 | 2.727273 | 72 | 0 | 239 |
| 941 | 91 | 2 | 72 | 0 | 239 |
| 941 | 182 | 5.940594 | 72 | 0 | 239 |
| 941 | 215 | 4.628099 | 72 | 0 | 239 |
| 942 | 0 | 5.128205 | 70 | 0 | 1 |
| 943 | 0 | 2.73794 | 61 | 1 | 859 |
| 943 | 24 | 5.154165 | 61 | 1 | 859 |
| 943 | 77 | 2.133333 | 61 | 1 | 859 |
| 943 | 140 | 2.727273 | 61 | 1 | 859 |
| 943 | 245 | 2.161606 | 61 | 1 | 859 |
| 943 | 343 | 22.64151 | 61 | 1 | 859 |
| 943 | 448 | 5.263158 | 61 | 1 | 859 |
| 943 | 504 | 6.25 | 61 | 1 | 859 |
| 943 | 560 | 5.607477 | 61 | 1 | 859 |
| 944 | 0 | 0.249169 | 68 | 0 | 1168 |
| 944 | 35 | 0.256027 | 68 | 0 | 1168 |
| 944 | 91 | 0.249169 | 68 | 0 | 1168 |
| 944 | 161 | 0.251414 | 68 | 0 | 1168 |
| 944 | 245 | 0.738689 | 68 | 0 | 1168 |
| 944 | 350 | 0.772574 | 68 | 0 | 1168 |
| 945 | 0 | 8.421053 | 67 | 1 | 36 |
| 945 | 14 | 5.825243 | 67 | 1 | 36 |
| 946 | 0 | 5.142332 | 88 | 0 | 678 |
| 946 | 33 | 5.882353 | 88 | 0 | 678 |
| 946 | 61 | 9.142857 | 88 | 0 | 678 |
| 946 | 152 | 11.00917 | 88 | 0 | 678 |
| 946 | 236 | 10.90909 | 88 | 0 | 678 |
| 946 | 355 | 8.888889 | 88 | 0 | 678 |
| 946 | 411 | 10.52632 | 88 | 0 | 678 |
| 946 | 502 | 10.61947 | 88 | 0 | 678 |
| 946 | 586 | 8.421053 | 88 | 0 | 678 |
| 947 | 0 | 2.727273 | 64 | 0 | 1044 |
| 947 | 28 | 2.702703 | 64 | 0 | 1044 |
| 947 | 84 | 2.335279 | 64 | 0 | 1044 |
| 947 | 175 | 1.212121 | 64 | 0 | 1044 |
| 947 | 259 | 1.440576 | 64 | 0 | 1044 |
| 947 | 357 | 1.769912 | 64 | 0 | 1044 |
| 947 | 441 | 2.181818 | 64 | 0 | 1044 |
| 947 | 602 | 2.752294 | 64 | 0 | 1044 |

| | | | | | |
|---|---|---|---|---|---|
| 948 | 0 | 1.026769 | 80 | 0 | 936 |
| 948 | 27 | 1.834862 | 80 | 0 | 936 |
| 948 | 83 | 1.869159 | 80 | 0 | 936 |
| 948 | 167 | 0.952381 | 80 | 0 | 936 |
| 948 | 258 | 1.553398 | 80 | 0 | 936 |
| 948 | 348 | 2.318841 | 80 | 0 | 936 |
| 948 | 440 | 1 | 80 | 0 | 936 |
| 948 | 496 | 0.389864 | 80 | 0 | 936 |
| 948 | 559 | 0.352734 | 80 | 0 | 936 |
| 949 | 0 | 1.724138 | 77 | 0 | 297 |
| 949 | 28 | 1.851852 | 77 | 0 | 297 |
| 949 | 84 | 1.754386 | 77 | 0 | 297 |
| 949 | 168 | 4.123711 | 77 | 0 | 297 |
| 950 | 0 | 4.918033 | 60 | 1 | 624 |
| 950 | 56 | 5.263158 | 60 | 1 | 624 |
| 950 | 140 | 5.217391 | 60 | 1 | 624 |
| 950 | 224 | 5.454545 | 60 | 1 | 624 |
| 950 | 336 | 5.607477 | 60 | 1 | 624 |
| 950 | 420 | 7.894737 | 60 | 1 | 624 |
| 950 | 476 | 7.826087 | 60 | 1 | 624 |
| 950 | 560 | 11.65049 | 60 | 1 | 624 |
| 951 | 0 | 5.769231 | 89 | 0 | 40 |
| 952 | 0 | 2.752294 | 74 | 1 | 283 |
| 952 | 28 | 4.752475 | 74 | 1 | 283 |
| 952 | 63 | 11.53846 | 74 | 1 | 283 |
| 952 | 148 | 9.230769 | 74 | 1 | 283 |
| 952 | 234 | 10.32258 | 74 | 1 | 283 |
| 953 | 0 | 2.542373 | 74 | 0 | 85 |
| 953 | 28 | 2.631579 | 74 | 0 | 85 |
| 953 | 84 | 2.777778 | 74 | 0 | 85 |
| 954 | 0 | 2.459016 | 65 | 0 | 1016 |
| 954 | 28 | 1.209677 | 65 | 0 | 1016 |
| 954 | 84 | 1.246106 | 65 | 0 | 1016 |
| 954 | 168 | 1.851852 | 65 | 0 | 1016 |
| 954 | 252 | 3.921569 | 65 | 0 | 1016 |
| 954 | 336 | 3.326403 | 65 | 0 | 1016 |
| 954 | 427 | 7.038123 | 65 | 0 | 1016 |
| 954 | 504 | 5.714286 | 65 | 0 | 1016 |
| 954 | 588 | 5.940594 | 65 | 0 | 1016 |

| | | | | | |
|---|---|---|---|---|---|
| 955 | 0 | 7.272727 | 59 | 0 | 676 |
| 955 | 77 | 24 | 59 | 0 | 676 |
| 955 | 211 | 6.112796 | 59 | 0 | 676 |
| 955 | 267 | 19.14894 | 59 | 0 | 676 |
| 955 | 364 | 12.30769 | 59 | 0 | 676 |
| 956 | 28 | 5.454545 | 78 | 0 | 871 |
| 956 | 56 | 5.217391 | 78 | 0 | 871 |
| 956 | 140 | 2.853261 | 78 | 0 | 871 |
| 957 | 0 | 8.219178 | 70 | 0 | 1067 |
| 957 | 28 | 5.405405 | 70 | 0 | 1067 |
| 957 | 43 | 8.571429 | 70 | 0 | 1067 |
| 957 | 168 | 17.14286 | 70 | 0 | 1067 |
| 957 | 210 | 10.90909 | 70 | 0 | 1067 |
| 957 | 304 | 25.80645 | 70 | 0 | 1067 |
| 957 | 413 | 11.56627 | 70 | 0 | 1067 |
| 957 | 484 | 13.33333 | 70 | 0 | 1067 |
| 957 | 597 | 10.21277 | 70 | 0 | 1067 |
| 958 | 17 | 12.69841 | 72 | 0 | 476 |
| 958 | 87 | 7.194245 | 72 | 0 | 476 |
| 958 | 171 | 4.8 | 72 | 0 | 476 |
| 958 | 255 | 1.930147 | 72 | 0 | 476 |
| 958 | 343 | 5.309735 | 72 | 0 | 476 |
| 958 | 399 | 7.438017 | 72 | 0 | 476 |
| 959 | 0 | 11.76471 | 80 | 0 | 719 |
| 959 | 91 | 16 | 80 | 0 | 719 |
| 959 | 168 | 12.12121 | 80 | 0 | 719 |
| 959 | 245 | 6.060606 | 80 | 0 | 719 |
| 959 | 329 | 11.32075 | 80 | 0 | 719 |
| 959 | 413 | 10.49563 | 80 | 0 | 719 |
| 959 | 511 | 12.10084 | 80 | 0 | 719 |
| 960 | 0 | 4.424779 | 35 | 0 | 710 |
| 960 | 28 | 8.695652 | 35 | 0 | 710 |
| 960 | 91 | 6.349206 | 35 | 0 | 710 |
| 960 | 154 | 1.526718 | 35 | 0 | 710 |
| 961 | 0 | 1.428571 | 70 | 0 | 1037 |
| 961 | 28 | 1.162791 | 70 | 0 | 1037 |
| 961 | 84 | 0.854701 | 70 | 0 | 1037 |
| 961 | 168 | 0.806452 | 70 | 0 | 1037 |
| 961 | 252 | 0.854701 | 70 | 0 | 1037 |

| | | | | | |
|---|---|---|---|---|---|
| 961 | 336 | 0.877193 | 70 | 0 | 1037 |
| 961 | 420 | 1.201201 | 70 | 0 | 1037 |
| 961 | 504 | 1.120448 | 70 | 0 | 1037 |
| 961 | 588 | 1.15942 | 70 | 0 | 1037 |
| 962 | 0 | 5.309735 | 81 | 0 | 715 |
| 962 | 28 | 1.886792 | 81 | 0 | 715 |
| 962 | 70 | 2.912621 | 81 | 0 | 715 |
| 962 | 154 | 2.857143 | 81 | 0 | 715 |
| 962 | 252 | 6.185567 | 81 | 0 | 715 |
| 962 | 343 | 4.848485 | 81 | 0 | 715 |
| 962 | 406 | 4.40367 | 81 | 0 | 715 |
| 962 | 511 | 4.8 | 81 | 0 | 715 |
| 962 | 616 | 2.167183 | 81 | 0 | 715 |
| 963 | 0 | 1.132075 | 70 | 1 | 309 |
| 963 | 28 | 1.363636 | 70 | 1 | 309 |
| 964 | 0 | 1.767305 | 84 | 1 | 234 |
| 964 | 56 | 5.785124 | 84 | 1 | 234 |
| 964 | 171 | 14.6789 | 84 | 1 | 234 |
| 965 | 0 | 1.639344 | 67 | 0 | 1138 |
| 965 | 28 | 1.785714 | 67 | 0 | 1138 |
| 965 | 84 | 1.818182 | 67 | 0 | 1138 |
| 965 | 168 | 2.608696 | 67 | 0 | 1138 |
| 965 | 259 | 1.769912 | 67 | 0 | 1138 |
| 965 | 343 | 1.785714 | 67 | 0 | 1138 |
| 965 | 427 | 1.754386 | 67 | 0 | 1138 |
| 965 | 511 | 3.738318 | 67 | 0 | 1138 |
| 965 | 595 | 3.703704 | 67 | 0 | 1138 |
| 966 | 0 | 2.727273 | 75 | 0 | 1156 |
| 966 | 28 | 2.678571 | 75 | 0 | 1156 |
| 966 | 84 | 3.539823 | 75 | 0 | 1156 |
| 966 | 168 | 3.539823 | 75 | 0 | 1156 |
| 966 | 252 | 2.678571 | 75 | 0 | 1156 |
| 966 | 336 | 2.777778 | 75 | 0 | 1156 |
| 966 | 420 | 2.752294 | 75 | 0 | 1156 |
| 966 | 511 | 4.752475 | 75 | 0 | 1156 |
| 966 | 574 | 12.90323 | 75 | 0 | 1156 |
| 967 | 0 | 3.418803 | 64 | 1 | 552 |
| 967 | 80 | 2.026171 | 64 | 1 | 552 |
| 967 | 153 | 1.987578 | 64 | 1 | 552 |

| | | | | | |
|---|---|---|---|---|---|
| 967 | 237 | 7.954545 | 64 | 1 | 552 |
| 967 | 321 | 5.940594 | 64 | 1 | 552 |
| 967 | 412 | 7.550562 | 64 | 1 | 552 |
| 967 | 514 | 10.54945 | 64 | 1 | 552 |
| 968 | 0 | 10.28571 | 69 | 0 | 1121 |
| 968 | 84 | 7.346939 | 69 | 0 | 1121 |
| 968 | 182 | 7.792208 | 69 | 0 | 1121 |
| 968 | 238 | 7.912088 | 69 | 0 | 1121 |
| 968 | 336 | 7.563025 | 69 | 0 | 1121 |
| 968 | 420 | 5.142857 | 69 | 0 | 1121 |
| 968 | 490 | 11.52 | 69 | 0 | 1121 |
| 968 | 567 | 9.302326 | 69 | 0 | 1121 |
| 969 | 0 | 3.333333 | 86 | 0 | 1047 |
| 969 | 28 | 1.626016 | 86 | 0 | 1047 |
| 969 | 84 | 1.169591 | 86 | 0 | 1047 |
| 969 | 168 | 1.869159 | 86 | 0 | 1047 |
| 969 | 252 | 1.769912 | 86 | 0 | 1047 |
| 969 | 336 | 3.550296 | 86 | 0 | 1047 |
| 969 | 427 | 6.545455 | 86 | 0 | 1047 |
| 969 | 504 | 5.825243 | 86 | 0 | 1047 |
| 969 | 588 | 5.769231 | 86 | 0 | 1047 |
| 970 | 0 | 1.652893 | 57 | 0 | 719 |
| 970 | 84 | 2.970297 | 57 | 0 | 719 |
| 970 | 168 | 2.912621 | 57 | 0 | 719 |
| 970 | 252 | 6.690562 | 57 | 0 | 719 |
| 970 | 336 | 16.51376 | 57 | 0 | 719 |
| 970 | 406 | 8.181818 | 57 | 0 | 719 |
| 970 | 518 | 9.115572 | 57 | 0 | 719 |
| 970 | 614 | 12 | 57 | 0 | 719 |
| 971 | 0 | 6.796117 | 59 | 0 | 907 |
| 971 | 24 | 4.324324 | 59 | 0 | 907 |
| 971 | 122 | 2.279202 | 59 | 0 | 907 |
| 971 | 164 | 3.061224 | 59 | 0 | 907 |
| 971 | 360 | 4.444444 | 59 | 0 | 907 |
| 971 | 395 | 3.340624 | 59 | 0 | 907 |
| 971 | 479 | 15.60372 | 59 | 0 | 907 |
| 971 | 577 | 5.357143 | 59 | 0 | 907 |
| 972 | 0 | 1.454545 | 54 | 1 | 546 |
| 972 | 84 | 1.139601 | 54 | 1 | 546 |

| | | | | | |
|---|---|---|---|---|---|
| 972 | 168 | 1.205857 | 54 | 1 | 546 |
| 972 | 253 | 1.294498 | 54 | 1 | 546 |
| 972 | 379 | 1.904762 | 54 | 1 | 546 |
| 972 | 456 | 4.255319 | 54 | 1 | 546 |
| 972 | 539 | 22.85714 | 54 | 1 | 546 |
| 973 | 0 | 2.439024 | 81 | 1 | 222 |
| 973 | 28 | 2.702703 | 81 | 1 | 222 |
| 973 | 84 | 11.56627 | 81 | 1 | 222 |
| 973 | 161 | 10.61947 | 81 | 1 | 222 |
| 974 | 0 | 1.119403 | 76 | 0 | 995 |
| 974 | 77 | 1.754386 | 76 | 0 | 995 |
| 974 | 175 | 1.81308 | 76 | 0 | 995 |
| 974 | 273 | 4.285714 | 76 | 0 | 995 |
| 974 | 329 | 2.605459 | 76 | 0 | 995 |
| 974 | 385 | 1.816216 | 76 | 0 | 995 |
| 974 | 511 | 1.660079 | 76 | 0 | 995 |
| 974 | 574 | 5.454545 | 76 | 0 | 995 |
| 975 | 0 | 8.695652 | 78 | 1 | 681 |
| 975 | 21 | 8.602151 | 78 | 1 | 681 |
| 975 | 91 | 5.333333 | 78 | 1 | 681 |
| 976 | 0 | 2.470588 | 67 | 0 | 679 |
| 976 | 34 | 1.973221 | 67 | 0 | 679 |
| 976 | 79 | 3.345281 | 67 | 0 | 679 |
| 976 | 174 | 3.612903 | 67 | 0 | 679 |
| 976 | 244 | 5.274725 | 67 | 0 | 679 |
| 976 | 314 | 6.494845 | 67 | 0 | 679 |
| 976 | 454 | 9.896907 | 67 | 0 | 679 |
| 976 | 531 | 6.997085 | 67 | 0 | 679 |
| 976 | 608 | 8.080808 | 67 | 0 | 679 |
| 977 | 0 | 15.58442 | 85 | 0 | 906 |
| 977 | 28 | 16.7916 | 85 | 0 | 906 |
| 977 | 51 | 48 | 85 | 0 | 906 |
| 977 | 149 | 18.46154 | 85 | 0 | 906 |
| 977 | 247 | 12.76596 | 85 | 0 | 906 |
| 977 | 422 | 2.884615 | 85 | 0 | 906 |
| 977 | 492 | 11.65049 | 85 | 0 | 906 |
| 977 | 576 | 12.37113 | 85 | 0 | 906 |
| 978 | 0 | 3.870968 | 68 | 1 | 590 |
| 978 | 35 | 3.609023 | 68 | 1 | 590 |

| | | | | | |
|---|---|---|---|---|---|
| 978 | 70 | 4.371585 | 68 | 1 | 590 |
| 978 | 176 | 3.75923 | 68 | 1 | 590 |
| 978 | 252 | 4.320988 | 68 | 1 | 590 |
| 978 | 323 | 7.207207 | 68 | 1 | 590 |
| 978 | 399 | 9.89011 | 68 | 1 | 590 |
| 978 | 484 | 15.25424 | 68 | 1 | 590 |
| 979 | 0 | 6.896552 | 64 | 0 | 836 |
| 979 | 21 | 5.172414 | 64 | 0 | 836 |
| 979 | 84 | 6.796117 | 64 | 0 | 836 |
| 979 | 136 | 4.247788 | 64 | 0 | 836 |
| 979 | 227 | 4.247788 | 64 | 0 | 836 |
| 979 | 309 | 6.603774 | 64 | 0 | 836 |
| 979 | 379 | 4.752475 | 64 | 0 | 836 |
| 979 | 520 | 4.752475 | 64 | 0 | 836 |
| 979 | 590 | 4.948454 | 64 | 0 | 836 |
| 980 | 0 | 2.777778 | 77 | 0 | 636 |
| 980 | 28 | 2.335279 | 77 | 0 | 636 |
| 980 | 89 | 2.752294 | 77 | 0 | 636 |
| 980 | 180 | 2.884615 | 77 | 0 | 636 |
| 980 | 243 | 2.803738 | 77 | 0 | 636 |
| 980 | 334 | 2.201835 | 77 | 0 | 636 |
| 980 | 427 | 2.222222 | 77 | 0 | 636 |
| 980 | 502 | 2.857143 | 77 | 0 | 636 |
| 980 | 566 | 2.242991 | 77 | 0 | 636 |
| 981 | 0 | 2.4 | 89 | 0 | 1457 |
| 981 | 28 | 1.440576 | 89 | 0 | 1457 |
| 981 | 77 | 1.385281 | 89 | 0 | 1457 |
| 981 | 154 | 1.827875 | 89 | 0 | 1457 |
| 981 | 245 | 3.584765 | 89 | 0 | 1457 |
| 981 | 336 | 10.16949 | 89 | 0 | 1457 |
| 981 | 413 | 3.305785 | 89 | 0 | 1457 |
| 981 | 497 | 3.571429 | 89 | 0 | 1457 |
| 981 | 581 | 3.389831 | 89 | 0 | 1457 |
| 982 | 0 | 5.940594 | 76 | 0 | 1146 |
| 982 | 105 | 4.324324 | 76 | 0 | 1146 |
| 982 | 175 | 4.324324 | 76 | 0 | 1146 |
| 982 | 252 | 4.571429 | 76 | 0 | 1146 |
| 982 | 322 | 6.923077 | 76 | 0 | 1146 |
| 982 | 427 | 6.428571 | 76 | 0 | 1146 |

| | | | | | |
|---|---|---|---|---|---|
| 982 | 497 | 6.666667 | 76 | 0 | 1146 |
| 983 | 0 | 11.32075 | 63 | 1 | 694 |
| 983 | 35 | 12.12121 | 63 | 1 | 694 |
| 983 | 91 | 11.32075 | 63 | 1 | 694 |
| 983 | 154 | 0.555813 | 63 | 1 | 694 |
| 983 | 268 | 11.85603 | 63 | 1 | 694 |
| 983 | 350 | 6.185567 | 63 | 1 | 694 |
| 983 | 420 | 5.882353 | 63 | 1 | 694 |
| 983 | 507 | 3.703704 | 63 | 1 | 694 |
| 983 | 588 | 6.060606 | 63 | 1 | 694 |
| 984 | 0 | 3.230769 | 86 | 0 | 330 |
| 984 | 84 | 6.451613 | 86 | 0 | 330 |
| 984 | 168 | 11.42857 | 86 | 0 | 330 |
| 984 | 224 | 11.32075 | 86 | 0 | 330 |
| 984 | 329 | 7.079646 | 86 | 0 | 330 |
| 985 | 0 | 1.315789 | 69 | 0 | 781 |
| 985 | 24 | 1.219512 | 69 | 0 | 781 |
| 985 | 87 | 2.453271 | 69 | 0 | 781 |
| 985 | 178 | 1.694915 | 69 | 0 | 781 |
| 985 | 241 | 0.714286 | 69 | 0 | 781 |
| 985 | 339 | 0.761905 | 69 | 0 | 781 |
| 985 | 409 | 0.617284 | 69 | 0 | 781 |
| 985 | 479 | 0.701754 | 69 | 0 | 781 |
| 985 | 549 | 0.357143 | 69 | 0 | 781 |
| 986 | 0 | 2.631579 | 65 | 1 | 126 |
| 986 | 28 | 2.857143 | 65 | 1 | 126 |
| 986 | 56 | 2.666667 | 65 | 1 | 126 |
| 987 | 0 | 0.612245 | 74 | 0 | 200 |
| 987 | 70 | 0.705882 | 74 | 0 | 200 |
| 988 | 0 | 1.906059 | 80 | 0 | 709 |
| 988 | 39 | 4.991087 | 80 | 0 | 709 |
| 988 | 77 | 4.948454 | 80 | 0 | 709 |
| 988 | 175 | 8 | 80 | 0 | 709 |
| 988 | 259 | 12.5 | 80 | 0 | 709 |
| 988 | 336 | 12.76596 | 80 | 0 | 709 |
| 988 | 420 | 30.37975 | 80 | 0 | 709 |
| 988 | 476 | 13.7931 | 80 | 0 | 709 |
| 988 | 574 | 19.35484 | 80 | 0 | 709 |
| 989 | 0 | 2.884615 | 48 | 1 | 67 |

| | | | | | |
|---|---|---|---|---|---|
| 989 | 28 | 2.803738 | 48 | 1 | 67 |
| 989 | 63 | 25.80645 | 48 | 1 | 67 |
| 990 | 0 | 5.128205 | 80 | 1 | 731 |
| 990 | 28 | 5.128205 | 80 | 1 | 731 |
| 990 | 91 | 5.309735 | 80 | 1 | 731 |
| 990 | 179 | 5.405405 | 80 | 1 | 731 |
| 990 | 277 | 12.07764 | 80 | 1 | 731 |
| 991 | 0 | 4.132231 | 70 | 0 | 1327 |
| 991 | 28 | 3.149606 | 70 | 0 | 1327 |
| 991 | 84 | 1.550388 | 70 | 0 | 1327 |
| 991 | 168 | 0.833333 | 70 | 0 | 1327 |
| 991 | 224 | 1.587302 | 70 | 0 | 1327 |
| 991 | 336 | 1.190476 | 70 | 0 | 1327 |
| 991 | 420 | 1.260504 | 70 | 0 | 1327 |
| 991 | 504 | 1.2 | 70 | 0 | 1327 |
| 991 | 588 | 1.229508 | 70 | 0 | 1327 |
| 992 | 0 | 2.752294 | 73 | 0 | 405 |
| 992 | 28 | 2.586207 | 73 | 0 | 405 |
| 992 | 56 | 5.405405 | 73 | 0 | 405 |
| 992 | 168 | 11.11111 | 73 | 0 | 405 |
| 992 | 231 | 12.5 | 73 | 0 | 405 |
| 993 | 0 | 0.802676 | 60 | 1 | 449 |
| 993 | 31 | 0.816882 | 60 | 1 | 449 |
| 993 | 63 | 2.424242 | 60 | 1 | 449 |
| 993 | 161 | 2.857143 | 60 | 1 | 449 |
| 993 | 245 | 6 | 60 | 1 | 449 |
| 993 | 329 | 16 | 60 | 1 | 449 |
| 993 | 420 | 4.210526 | 60 | 1 | 449 |
| 994 | 0 | 5.128205 | 74 | 0 | 645 |
| 994 | 28 | 2.34375 | 74 | 0 | 645 |
| 994 | 56 | 2.564103 | 74 | 0 | 645 |
| 994 | 162 | 1.315789 | 74 | 0 | 645 |
| 994 | 260 | 1.470588 | 74 | 0 | 645 |
| 994 | 336 | 1.456311 | 74 | 0 | 645 |
| 994 | 420 | 1.470588 | 74 | 0 | 645 |
| 994 | 504 | 1.401869 | 74 | 0 | 645 |
| 994 | 588 | 1.376147 | 74 | 0 | 645 |
| 995 | 0 | 1.004304 | 78 | 0 | 961 |
| 995 | 28 | 2.586207 | 78 | 0 | 961 |

| | | | | | |
|---|---|---|---|---|---|
| 995 | 84 | 0.465116 | 78 | 0 | 961 |
| 995 | 168 | 0.535714 | 78 | 0 | 961 |
| 996 | 0 | 4.958678 | 65 | 1 | 50 |
| 996 | 28 | 4.444444 | 65 | 1 | 50 |
| 997 | 0 | 2.051282 | 72 | 0 | 581 |
| 997 | 28 | 2.393844 | 72 | 0 | 581 |
| 997 | 57 | 2.521008 | 72 | 0 | 581 |
| 997 | 139 | 2.4 | 72 | 0 | 581 |
| 997 | 251 | 2.497027 | 72 | 0 | 581 |
| 997 | 308 | 2.181818 | 72 | 0 | 581 |
| 997 | 371 | 2.564103 | 72 | 0 | 581 |
| 997 | 511 | 2.777778 | 72 | 0 | 581 |
| 997 | 546 | 2.631579 | 72 | 0 | 581 |
| 998 | 0 | 2.727273 | 78 | 1 | 667 |
| 998 | 28 | 2.777778 | 78 | 1 | 667 |
| 998 | 56 | 2.702703 | 78 | 1 | 667 |
| 998 | 154 | 2.830189 | 78 | 1 | 667 |
| 998 | 238 | 2.803738 | 78 | 1 | 667 |
| 998 | 336 | 2.830189 | 78 | 1 | 667 |
| 998 | 420 | 2.264151 | 78 | 1 | 667 |
| 998 | 483 | 2.474227 | 78 | 1 | 667 |
| 998 | 574 | 2.912621 | 78 | 1 | 667 |
| 999 | 0 | 3.603604 | 43 | 1 | 772 |
| 999 | 28 | 3.738318 | 43 | 1 | 772 |
| 999 | 56 | 3.636364 | 43 | 1 | 772 |
| 999 | 140 | 9.230769 | 43 | 1 | 772 |
| 999 | 210 | 10 | 43 | 1 | 772 |
| 999 | 301 | 14.81481 | 43 | 1 | 772 |
| 999 | 378 | 6.896552 | 43 | 1 | 772 |
| 999 | 469 | 5.357143 | 43 | 1 | 772 |
| 999 | 553 | 26.56126 | 43 | 1 | 772 |
| 1000 | 0 | 3.738318 | 54 | 0 | 1142 |
| 1000 | 28 | 3.738318 | 54 | 0 | 1142 |
| 1000 | 84 | 3.603604 | 54 | 0 | 1142 |
| 1000 | 168 | 3.773585 | 54 | 0 | 1142 |
| 1000 | 254 | 3.988604 | 54 | 0 | 1142 |
| 1000 | 336 | 3.246377 | 54 | 0 | 1142 |
| 1000 | 415 | 3.448276 | 54 | 0 | 1142 |
| 1000 | 506 | 3.305785 | 54 | 0 | 1142 |

| | | | | | |
|---|---|---|---|---|---|
| 1000 | 562 | 3.539823 | 54 | 0 | 1142 |
| 1001 | 0 | 6.338028 | 41 | 1 | 73 |
| 1001 | 35 | 10 | 41 | 1 | 73 |
| 1001 | 72 | 27.69231 | 41 | 1 | 73 |
| 1002 | 0 | 2.222222 | 69 | 1 | 2 |
| 1003 | 0 | 3.349282 | 65 | 0 | 1080 |
| 1003 | 21 | 0.589474 | 65 | 0 | 1080 |
| 1003 | 91 | 0.59469 | 65 | 0 | 1080 |
| 1003 | 146 | 1.843318 | 65 | 0 | 1080 |
| 1003 | 237 | 1.632653 | 65 | 0 | 1080 |
| 1003 | 328 | 2.777778 | 65 | 0 | 1080 |
| 1003 | 412 | 3.960396 | 65 | 0 | 1080 |
| 1003 | 503 | 6.722689 | 65 | 0 | 1080 |
| 1003 | 552 | 10.60383 | 65 | 0 | 1080 |
| 1004 | 0 | 10.18388 | 65 | 0 | 1119 |
| 1004 | 49 | 5.011435 | 65 | 0 | 1119 |
| 1004 | 105 | 4.758757 | 65 | 0 | 1119 |
| 1004 | 189 | 10.34483 | 65 | 0 | 1119 |
| 1004 | 280 | 8.294931 | 65 | 0 | 1119 |
| 1004 | 371 | 5.081157 | 65 | 0 | 1119 |
| 1004 | 454 | 5.783133 | 65 | 0 | 1119 |
| 1004 | 545 | 5.594406 | 65 | 0 | 1119 |
| 1005 | 0 | 1.574803 | 90 | 0 | 820 |
| 1005 | 28 | 1.587302 | 90 | 0 | 820 |
| 1005 | 84 | 2.419355 | 90 | 0 | 820 |
| 1005 | 168 | 0.806452 | 90 | 0 | 820 |
| 1006 | 0 | 1.801802 | 63 | 1 | 77 |
| 1006 | 18 | 1.960784 | 63 | 1 | 77 |
| 1006 | 76 | 1.367855 | 63 | 1 | 77 |
| 1007 | 0 | 5.504587 | 78 | 0 | 957 |
| 1007 | 28 | 2.689076 | 78 | 0 | 957 |
| 1007 | 98 | 2.758621 | 78 | 0 | 957 |
| 1007 | 161 | 5.172414 | 78 | 0 | 957 |
| 1007 | 252 | 2.5 | 78 | 0 | 957 |
| 1007 | 343 | 0.813008 | 78 | 0 | 957 |
| 1007 | 399 | 1.441441 | 78 | 0 | 957 |
| 1007 | 490 | 1.246106 | 78 | 0 | 957 |
| 1007 | 602 | 3.539823 | 78 | 0 | 957 |
| 1008 | 0 | 2.376238 | 73 | 0 | 1192 |

| | | | | | |
|---|---|---|---|---|---|
| 1008 | 35 | 1.237113 | 73 | 0 | 1192 |
| 1008 | 105 | 1.188119 | 73 | 0 | 1192 |
| 1008 | 162 | 1.306173 | 73 | 0 | 1192 |
| 1008 | 221 | 3.030303 | 73 | 0 | 1192 |
| 1008 | 315 | 3.689065 | 73 | 0 | 1192 |
| 1008 | 389 | 4.182226 | 73 | 0 | 1192 |
| 1008 | 466 | 3.69637 | 73 | 0 | 1192 |
| 1008 | 546 | 4.242424 | 73 | 0 | 1192 |
| 1009 | 0 | 0.763359 | 49 | 0 | 974 |
| 1009 | 28 | 0.862069 | 49 | 0 | 974 |
| 1009 | 84 | 0.254291 | 49 | 0 | 974 |
| 1009 | 175 | 0.442478 | 49 | 0 | 974 |
| 1009 | 231 | 0.683761 | 49 | 0 | 974 |
| 1009 | 329 | 0.787402 | 49 | 0 | 974 |
| 1009 | 427 | 0.877193 | 49 | 0 | 974 |
| 1009 | 518 | 0.620155 | 49 | 0 | 974 |
| 1009 | 588 | 0.64 | 49 | 0 | 974 |
| 1010 | 0 | 5.882353 | 50 | 1 | 463 |
| 1010 | 28 | 4.615385 | 50 | 1 | 463 |
| 1010 | 91 | 9.69697 | 50 | 1 | 463 |
| 1010 | 161 | 15.31915 | 50 | 1 | 463 |
| 1010 | 252 | 16.17978 | 50 | 1 | 463 |
| 1010 | 353 | 20.68966 | 50 | 1 | 463 |
| 1010 | 409 | 20 | 50 | 1 | 463 |
| 1011 | 0 | 1.401869 | 79 | 1 | 154 |
| 1011 | 104 | 3 | 79 | 1 | 154 |
| 1011 | 141 | 26.37363 | 79 | 1 | 154 |
| 1012 | 0 | 5.714286 | 81 | 1 | 749 |
| 1012 | 28 | 5.660377 | 81 | 1 | 749 |
| 1012 | 84 | 4.444444 | 81 | 1 | 749 |
| 1012 | 182 | 6.818182 | 81 | 1 | 749 |
| 1012 | 273 | 11.21495 | 81 | 1 | 749 |
| 1012 | 329 | 2.279202 | 81 | 1 | 749 |
| 1012 | 413 | 4.040404 | 81 | 1 | 749 |
| 1012 | 518 | 4.285714 | 81 | 1 | 749 |
| 1012 | 588 | 5 | 81 | 1 | 749 |
| 1013 | 0 | 2.604651 | 79 | 0 | 827 |
| 1013 | 25 | 2.380952 | 79 | 0 | 827 |
| 1013 | 95 | 3.252033 | 79 | 0 | 827 |

| | | | | | |
|---|---|---|---|---|---|
| 1013 | 137 | 3.174603 | 79 | 0 | 827 |
| 1013 | 256 | 3.225806 | 79 | 0 | 827 |
| 1013 | 336 | 0.787402 | 79 | 0 | 827 |
| 1013 | 420 | 2.586207 | 79 | 0 | 827 |
| 1013 | 504 | 2.542373 | 79 | 0 | 827 |
| 1013 | 588 | 1.694915 | 79 | 0 | 827 |
| 1014 | 0 | 11.65049 | 84 | 0 | 925 |
| 1014 | 14 | 2.608696 | 84 | 0 | 925 |
| 1014 | 77 | 2.4 | 84 | 0 | 925 |
| 1014 | 175 | 2.941176 | 84 | 0 | 925 |
| 1014 | 259 | 3.603604 | 84 | 0 | 925 |
| 1014 | 322 | 2.586207 | 84 | 0 | 925 |
| 1014 | 420 | 5.882353 | 84 | 0 | 925 |
| 1014 | 504 | 5.882353 | 84 | 0 | 925 |
| 1014 | 595 | 6.122449 | 84 | 0 | 925 |
| 1015 | 0 | 0.497512 | 88 | 0 | 1259 |
| 1015 | 28 | 0.516796 | 88 | 0 | 1259 |
| 1015 | 84 | 0.869565 | 88 | 0 | 1259 |
| 1015 | 168 | 0.2849 | 88 | 0 | 1259 |
| 1015 | 252 | 0.2849 | 88 | 0 | 1259 |
| 1015 | 336 | 0.254453 | 88 | 0 | 1259 |
| 1015 | 420 | 0.409836 | 88 | 0 | 1259 |
| 1015 | 504 | 0.384615 | 88 | 0 | 1259 |
| 1015 | 588 | 0.406504 | 88 | 0 | 1259 |
| 1016 | 0 | 4.75382 | 67 | 0 | 376 |
| 1016 | 31 | 24.74227 | 67 | 0 | 376 |
| 1016 | 91 | 6.222222 | 67 | 0 | 376 |
| 1016 | 175 | 17.97753 | 67 | 0 | 376 |
| 1016 | 266 | 22.85714 | 67 | 0 | 376 |
| 1016 | 347 | 18.94737 | 67 | 0 | 376 |
| 1017 | 0 | 5.825243 | 73 | 0 | 888 |
| 1017 | 28 | 5.504587 | 73 | 0 | 888 |
| 1017 | 84 | 5.084746 | 73 | 0 | 888 |
| 1017 | 154 | 5.263158 | 73 | 0 | 888 |
| 1017 | 238 | 4.137931 | 73 | 0 | 888 |
| 1017 | 329 | 6.666667 | 73 | 0 | 888 |
| 1017 | 448 | 7.563025 | 73 | 0 | 888 |
| 1017 | 483 | 2.016807 | 73 | 0 | 888 |
| 1017 | 581 | 2.033898 | 73 | 0 | 888 |

| | | | | | |
|---|---|---|---|---|---|
| 1018 | 0 | 2.912621 | 79 | 0 | 1121 |
| 1018 | 56 | 4.984424 | 79 | 0 | 1121 |
| 1018 | 168 | 3.571429 | 79 | 0 | 1121 |
| 1018 | 287 | 2.758621 | 79 | 0 | 1121 |
| 1018 | 343 | 8.247423 | 79 | 0 | 1121 |
| 1018 | 413 | 3.389831 | 79 | 0 | 1121 |
| 1018 | 518 | 3.448276 | 79 | 0 | 1121 |
| 1018 | 630 | 3.856749 | 79 | 0 | 1121 |
| 1019 | 0 | 5.940594 | 68 | 0 | 1086 |
| 1019 | 98 | 5.405405 | 68 | 0 | 1086 |
| 1019 | 154 | 6.593407 | 68 | 0 | 1086 |
| 1019 | 252 | 10.71429 | 68 | 0 | 1086 |
| 1019 | 357 | 9.375 | 68 | 0 | 1086 |
| 1019 | 406 | 9.375 | 68 | 0 | 1086 |
| 1019 | 518 | 7.619048 | 68 | 0 | 1086 |
| 1019 | 581 | 7.76699 | 68 | 0 | 1086 |
| 1020 | 0 | 1.982301 | 77 | 0 | 1061 |
| 1020 | 27 | 2.339181 | 77 | 0 | 1061 |
| 1020 | 69 | 1.630989 | 77 | 0 | 1061 |
| 1020 | 132 | 1.630989 | 77 | 0 | 1061 |
| 1020 | 258 | 1.677149 | 77 | 0 | 1061 |
| 1020 | 300 | 0.880088 | 77 | 0 | 1061 |
| 1020 | 426 | 0.897868 | 77 | 0 | 1061 |
| 1021 | 0 | 1.305361 | 78 | 0 | 786 |
| 1021 | 34 | 1.652893 | 78 | 0 | 786 |
| 1021 | 69 | 0.516796 | 78 | 0 | 786 |
| 1021 | 167 | 0.387597 | 78 | 0 | 786 |
| 1022 | 0 | 5.660377 | 81 | 1 | 186 |
| 1022 | 28 | 5.454545 | 81 | 1 | 186 |
| 1022 | 84 | 12.5 | 81 | 1 | 186 |
| 1022 | 175 | 19.35484 | 81 | 1 | 186 |
| 1023 | 0 | 8.737864 | 82 | 1 | 856 |
| 1023 | 28 | 11.65049 | 82 | 1 | 856 |
| 1023 | 84 | 18.36735 | 82 | 1 | 856 |
| 1023 | 175 | 25.26316 | 82 | 1 | 856 |
| 1023 | 245 | 18.18182 | 82 | 1 | 856 |
| 1023 | 329 | 18.5567 | 82 | 1 | 856 |
| 1023 | 427 | 16.17978 | 82 | 1 | 856 |
| 1023 | 518 | 19.56522 | 82 | 1 | 856 |

| | | | | | |
|---|---|---|---|---|---|
| 1023 | 581 | 20.45455 | 82 | 1 | 856 |
| 1024 | 0 | 2.564103 | 67 | 0 | 981 |
| 1024 | 70 | 3.738318 | 67 | 0 | 981 |
| 1025 | 0 | 0.847458 | 68 | 0 | 653 |
| 1025 | 21 | 0.862069 | 68 | 0 | 653 |
| 1025 | 84 | 0.745342 | 68 | 0 | 653 |
| 1025 | 147 | 2.777778 | 68 | 0 | 653 |
| 1025 | 231 | 1.754386 | 68 | 0 | 653 |
| 1025 | 336 | 2.016807 | 68 | 0 | 653 |
| 1025 | 406 | 2.542373 | 68 | 0 | 653 |
| 1025 | 490 | 2.142857 | 68 | 0 | 653 |
| 1025 | 560 | 2.222222 | 68 | 0 | 653 |
| 1026 | 0 | 2.803738 | 69 | 0 | 666 |
| 1026 | 35 | 1.904762 | 69 | 0 | 666 |
| 1026 | 119 | 2.857143 | 69 | 0 | 666 |
| 1026 | 217 | 2 | 69 | 0 | 666 |
| 1026 | 329 | 2.777778 | 69 | 0 | 666 |
| 1026 | 392 | 2.264151 | 69 | 0 | 666 |
| 1026 | 483 | 1.388889 | 69 | 0 | 666 |
| 1026 | 588 | 7.42268 | 69 | 0 | 666 |
| 1027 | 0 | 2.882883 | 60 | 0 | 743 |
| 1027 | 35 | 3.773585 | 60 | 0 | 743 |
| 1027 | 89 | 7.272727 | 60 | 0 | 743 |
| 1027 | 182 | 8.928571 | 60 | 0 | 743 |
| 1027 | 238 | 12.61261 | 60 | 0 | 743 |
| 1027 | 329 | 12.84404 | 60 | 0 | 743 |
| 1027 | 420 | 9.911504 | 60 | 0 | 743 |
| 1027 | 509 | 11.26437 | 60 | 0 | 743 |
| 1027 | 595 | 15.2381 | 60 | 0 | 743 |
| 1028 | 0 | 1.605351 | 62 | 1 | 261 |
| 1028 | 21 | 1.564537 | 62 | 1 | 261 |
| 1028 | 56 | 6.185567 | 62 | 1 | 261 |
| 1028 | 139 | 15.65217 | 62 | 1 | 261 |
| 1028 | 210 | 35.29412 | 62 | 1 | 261 |
| 1029 | 0 | 4.958678 | 71 | 0 | 381 |
| 1029 | 28 | 5.128205 | 71 | 0 | 381 |
| 1029 | 84 | 5.084746 | 71 | 0 | 381 |
| 1029 | 168 | 4.705882 | 71 | 0 | 381 |
| 1030 | 0 | 3.204272 | 71 | 1 | 620 |

| | | | | | |
|---|---|---|---|---|---|
| 1030 | 56 | 1.5 | 71 | 1 | 620 |
| 1030 | 157 | 3.636364 | 71 | 1 | 620 |
| 1030 | 241 | 8.163265 | 71 | 1 | 620 |
| 1030 | 306 | 5.708461 | 71 | 1 | 620 |
| 1030 | 402 | 10.54945 | 71 | 1 | 620 |
| 1030 | 504 | 8.791209 | 71 | 1 | 620 |
| 1030 | 546 | 10.25641 | 71 | 1 | 620 |
| 1031 | 0 | 0.57554 | 59 | 0 | 737 |
| 1031 | 21 | 0.588235 | 59 | 0 | 737 |
| 1031 | 56 | 0.83004 | 59 | 0 | 737 |
| 1031 | 155 | 0.852273 | 59 | 0 | 737 |
| 1031 | 232 | 1.632653 | 59 | 0 | 737 |
| 1031 | 337 | 0.943396 | 59 | 0 | 737 |
| 1031 | 442 | 0.869565 | 59 | 0 | 737 |
| 1031 | 505 | 0.934579 | 59 | 0 | 737 |
| 1032 | 0 | 1.666667 | 79 | 0 | 852 |
| 1032 | 28 | 1.652893 | 79 | 0 | 852 |
| 1032 | 77 | 1.818182 | 79 | 0 | 852 |
| 1032 | 203 | 1.139601 | 79 | 0 | 852 |
| 1032 | 266 | 1.454545 | 79 | 0 | 852 |
| 1032 | 364 | 1.454545 | 79 | 0 | 852 |
| 1033 | 0 | 8.247423 | 87 | 0 | 969 |
| 1033 | 28 | 10.52632 | 87 | 0 | 969 |
| 1033 | 84 | 11.42857 | 87 | 0 | 969 |
| 1033 | 168 | 17.64706 | 87 | 0 | 969 |
| 1033 | 266 | 13.21101 | 87 | 0 | 969 |
| 1033 | 357 | 12.74336 | 87 | 0 | 969 |
| 1033 | 441 | 16.82243 | 87 | 0 | 969 |
| 1033 | 574 | 22.01835 | 87 | 0 | 969 |
| 1034 | 0 | 1.923077 | 75 | 0 | 876 |
| 1034 | 28 | 1.923077 | 75 | 0 | 876 |
| 1034 | 84 | 1 | 75 | 0 | 876 |
| 1034 | 168 | 1.380671 | 75 | 0 | 876 |
| 1034 | 246 | 1.408097 | 75 | 0 | 876 |
| 1034 | 315 | 0.970874 | 75 | 0 | 876 |
| 1034 | 399 | 1.030928 | 75 | 0 | 876 |
| 1034 | 511 | 0.934579 | 75 | 0 | 876 |
| 1035 | 0 | 2.086957 | 88 | 0 | 460 |
| 1035 | 70 | 1.818182 | 88 | 0 | 460 |

| | | | | | |
|---|---|---|---|---|---|
| 1035 | 161 | 4.752475 | 88 | 0 | 460 |
| 1035 | 232 | 4.615385 | 88 | 0 | 460 |
| 1036 | 0 | 1.904762 | 66 | 0 | 680 |
| 1036 | 35 | 2.609506 | 66 | 0 | 680 |
| 1036 | 91 | 5.454545 | 66 | 0 | 680 |
| 1036 | 154 | 4.958678 | 66 | 0 | 680 |
| 1036 | 210 | 3.053435 | 66 | 0 | 680 |
| 1036 | 364 | 5.128205 | 66 | 0 | 680 |
| 1036 | 447 | 5.084746 | 66 | 0 | 680 |
| 1037 | 0 | 1.869159 | 89 | 0 | 575 |
| 1037 | 84 | 4.123711 | 89 | 0 | 575 |
| 1037 | 168 | 3.773585 | 89 | 0 | 575 |
| 1037 | 252 | 6.747891 | 89 | 0 | 575 |
| 1037 | 329 | 6.480648 | 89 | 0 | 575 |
| 1037 | 406 | 7.272727 | 89 | 0 | 575 |
| 1037 | 476 | 7.272727 | 89 | 0 | 575 |
| 1038 | 0 | 8.571429 | 86 | 0 | 1023 |
| 1038 | 84 | 14.6789 | 86 | 0 | 1023 |
| 1038 | 168 | 5.177994 | 86 | 0 | 1023 |
| 1038 | 224 | 11.21495 | 86 | 0 | 1023 |
| 1038 | 308 | 8.727273 | 86 | 0 | 1023 |
| 1038 | 399 | 10.61947 | 86 | 0 | 1023 |
| 1038 | 483 | 9.836066 | 86 | 0 | 1023 |
| 1039 | 0 | 5.263158 | 76 | 1 | 544 |
| 1039 | 28 | 5 | 76 | 1 | 544 |
| 1039 | 56 | 4.102564 | 76 | 1 | 544 |
| 1039 | 140 | 5.454545 | 76 | 1 | 544 |
| 1039 | 224 | 3.488372 | 76 | 1 | 544 |
| 1039 | 296 | 12.37113 | 76 | 1 | 544 |
| 1039 | 392 | 11.32075 | 76 | 1 | 544 |
| 1039 | 485 | 11.53846 | 76 | 1 | 544 |
| 1040 | 0 | 3.703704 | 67 | 0 | 749 |
| 1041 | 0 | 4.752475 | 87 | 0 | 743 |
| 1041 | 35 | 5.882353 | 87 | 0 | 743 |
| 1041 | 112 | 6.25 | 87 | 0 | 743 |
| 1041 | 168 | 4.580153 | 87 | 0 | 743 |
| 1041 | 259 | 2.727273 | 87 | 0 | 743 |
| 1041 | 350 | 2.485943 | 87 | 0 | 743 |
| 1041 | 441 | 2.830189 | 87 | 0 | 743 |

| | | | | | |
|---|---|---|---|---|---|
| 1041 | 532 | 5.607477 | 87 | 0 | 743 |
| 1041 | 595 | 4.444444 | 87 | 0 | 743 |
| 1042 | 0 | 0.396825 | 76 | 1 | 713 |
| 1042 | 28 | 0.406504 | 76 | 1 | 713 |
| 1042 | 84 | 2.702703 | 76 | 1 | 713 |
| 1042 | 175 | 1.694915 | 76 | 1 | 713 |
| 1042 | 259 | 1.818182 | 76 | 1 | 713 |
| 1042 | 322 | 1.043478 | 76 | 1 | 713 |
| 1042 | 420 | 4.363636 | 76 | 1 | 713 |
| 1042 | 490 | 2.654867 | 76 | 1 | 713 |
| 1042 | 616 | 9.795918 | 76 | 1 | 713 |
| 1043 | 0 | 2.5 | 72 | 0 | 771 |
| 1043 | 28 | 2.459016 | 72 | 0 | 771 |
| 1043 | 91 | 2.727273 | 72 | 0 | 771 |
| 1043 | 168 | 2.086957 | 72 | 0 | 771 |
| 1043 | 259 | 3.703704 | 72 | 0 | 771 |
| 1043 | 336 | 2.264151 | 72 | 0 | 771 |
| 1043 | 427 | 2.521008 | 72 | 0 | 771 |
| 1043 | 504 | 2.307692 | 72 | 0 | 771 |
| 1043 | 595 | 12.2449 | 72 | 0 | 771 |
| 1044 | 0 | 2.857143 | 63 | 1 | 422 |
| 1044 | 28 | 2.857143 | 63 | 1 | 422 |
| 1044 | 77 | 1.490683 | 63 | 1 | 422 |
| 1044 | 165 | 1.25 | 63 | 1 | 422 |
| 1044 | 256 | 5.16129 | 63 | 1 | 422 |
| 1044 | 347 | 5.714286 | 63 | 1 | 422 |
| 1044 | 420 | 4.605263 | 63 | 1 | 422 |
| 1045 | 0 | 4.166667 | 57 | 0 | 673 |
| 1045 | 36 | 1.490683 | 57 | 0 | 673 |
| 1045 | 78 | 1.558442 | 57 | 0 | 673 |
| 1045 | 168 | 5.882353 | 57 | 0 | 673 |
| 1045 | 257 | 2.380952 | 57 | 0 | 673 |
| 1045 | 364 | 2 | 57 | 0 | 673 |
| 1045 | 420 | 2.151639 | 57 | 0 | 673 |
| 1045 | 490 | 1.782683 | 57 | 0 | 673 |
| 1045 | 568 | 6.722689 | 57 | 0 | 673 |
| 1046 | 0 | 2.380952 | 64 | 0 | 666 |
| 1047 | 0 | 3.448276 | 76 | 0 | 995 |
| 1047 | 98 | 5.357143 | 76 | 0 | 995 |

| | | | | | |
|---|---|---|---|---|---|
| 1047 | 161 | 7.76699 | 76 | 0 | 995 |
| 1047 | 245 | 25 | 76 | 0 | 995 |
| 1047 | 343 | 11.32075 | 76 | 0 | 995 |
| 1047 | 420 | 3.210702 | 76 | 0 | 995 |
| 1047 | 511 | 2.857143 | 76 | 0 | 995 |
| 1047 | 567 | 2.678571 | 76 | 0 | 995 |
| 1048 | 0 | 1.503759 | 81 | 0 | 639 |
| 1048 | 79 | 1.071429 | 81 | 0 | 639 |
| 1048 | 149 | 2.803738 | 81 | 0 | 639 |
| 1048 | 231 | 4.705882 | 81 | 0 | 639 |
| 1048 | 357 | 0.661157 | 81 | 0 | 639 |
| 1048 | 420 | 0.655201 | 81 | 0 | 639 |
| 1048 | 492 | 1.263348 | 81 | 0 | 639 |
| 1048 | 609 | 1.069519 | 81 | 0 | 639 |
| 1049 | 0 | 2.991453 | 72 | 0 | 1044 |
| 1049 | 56 | 2.990654 | 72 | 0 | 1044 |
| 1049 | 175 | 3.603604 | 72 | 0 | 1044 |
| 1049 | 231 | 3.571429 | 72 | 0 | 1044 |
| 1049 | 350 | 2.242991 | 72 | 0 | 1044 |
| 1049 | 441 | 2.912621 | 72 | 0 | 1044 |
| 1049 | 497 | 2.970297 | 72 | 0 | 1044 |
| 1049 | 616 | 3.168317 | 72 | 0 | 1044 |
| 1050 | 0 | 4.670559 | 34 | 1 | 68 |
| 1051 | 0 | 1.626016 | 47 | 0 | 698 |
| 1051 | 27 | 5.137615 | 47 | 0 | 698 |
| 1051 | 82 | 5.090909 | 47 | 0 | 698 |
| 1051 | 160 | 6.19469 | 47 | 0 | 698 |
| 1051 | 245 | 5.045045 | 47 | 0 | 698 |
| 1051 | 335 | 8.028674 | 47 | 0 | 698 |
| 1051 | 420 | 10.20833 | 47 | 0 | 698 |
| 1051 | 497 | 5.291339 | 47 | 0 | 698 |
| 1051 | 578 | 5.084746 | 47 | 0 | 698 |
| 1052 | 0 | 2.307692 | 49 | 0 | 645 |
| 1052 | 35 | 2.222222 | 49 | 0 | 645 |
| 1052 | 98 | 2.727273 | 49 | 0 | 645 |
| 1052 | 161 | 2.830189 | 49 | 0 | 645 |
| 1052 | 252 | 2.912621 | 49 | 0 | 645 |
| 1052 | 315 | 2.912621 | 49 | 0 | 645 |
| 1052 | 399 | 2.016807 | 49 | 0 | 645 |

| | | | | | |
|---|---|---|---|---|---|
| 1052 | 525 | 2.631579 | 49 | 0 | 645 |
| 1052 | 588 | 2.105263 | 49 | 0 | 645 |
| 1053 | 0 | 12 | 79 | 0 | 456 |
| 1053 | 28 | 11.76471 | 79 | 0 | 456 |
| 1053 | 56 | 1.801802 | 79 | 0 | 456 |
| 1053 | 259 | 3.870968 | 79 | 0 | 456 |
| 1053 | 329 | 4 | 79 | 0 | 456 |
| 1053 | 420 | 5.309735 | 79 | 0 | 456 |
| 1054 | 0 | 3.252033 | 64 | 1 | 1123 |
| 1054 | 28 | 4.6875 | 64 | 1 | 1123 |
| 1054 | 56 | 6.722689 | 64 | 1 | 1123 |
| 1054 | 140 | 5.084746 | 64 | 1 | 1123 |
| 1054 | 266 | 4.485981 | 64 | 1 | 1123 |
| 1054 | 330 | 4.533189 | 64 | 1 | 1123 |
| 1054 | 392 | 4.324324 | 64 | 1 | 1123 |
| 1054 | 488 | 5.263158 | 64 | 1 | 1123 |
| 1054 | 546 | 10.61947 | 64 | 1 | 1123 |
| 1055 | 28 | 0.847458 | 56 | 1 | 996 |
| 1055 | 84 | 1.481481 | 56 | 1 | 996 |
| 1055 | 154 | 2.539683 | 56 | 1 | 996 |
| 1055 | 241 | 3.333333 | 56 | 1 | 996 |
| 1055 | 315 | 2.909091 | 56 | 1 | 996 |
| 1055 | 420 | 2.990654 | 56 | 1 | 996 |
| 1055 | 490 | 2.909091 | 56 | 1 | 996 |
| 1055 | 553 | 3.846154 | 56 | 1 | 996 |
| 1056 | 0 | 5.263158 | 76 | 1 | 567 |
| 1056 | 28 | 7.272727 | 76 | 1 | 567 |
| 1056 | 77 | 5.454545 | 76 | 1 | 567 |
| 1056 | 161 | 5.555556 | 76 | 1 | 567 |
| 1056 | 245 | 5.454545 | 76 | 1 | 567 |
| 1056 | 329 | 6.213592 | 76 | 1 | 567 |
| 1056 | 413 | 11.65049 | 76 | 1 | 567 |
| 1056 | 497 | 23.30097 | 76 | 1 | 567 |
| 1057 | 0 | 15.38462 | 67 | 1 | 792 |
| 1057 | 35 | 20.44625 | 67 | 1 | 792 |
| 1057 | 92 | 18.07747 | 67 | 1 | 792 |
| 1057 | 176 | 30.58252 | 67 | 1 | 792 |
| 1057 | 262 | 17.82178 | 67 | 1 | 792 |
| 1057 | 339 | 34.95146 | 67 | 1 | 792 |

| | | | | | |
|---|---|---|---|---|---|
| 1057 | 437 | 17.14286 | 67 | 1 | 792 |
| 1057 | 521 | 18.60465 | 67 | 1 | 792 |
| 1057 | 570 | 14.17323 | 67 | 1 | 792 |
| 1058 | 0 | 7.54717 | 62 | 0 | 561 |
| 1058 | 28 | 2.666667 | 62 | 0 | 561 |
| 1058 | 98 | 2.990654 | 62 | 0 | 561 |
| 1058 | 161 | 4.615385 | 62 | 0 | 561 |
| 1058 | 252 | 4.485981 | 62 | 0 | 561 |
| 1058 | 322 | 4.615385 | 62 | 0 | 561 |
| 1058 | 434 | 3.703704 | 62 | 0 | 561 |
| 1058 | 504 | 5.769231 | 62 | 0 | 561 |
| 1058 | 560 | 5.504587 | 62 | 0 | 561 |
| 1059 | 0 | 1.043025 | 74 | 0 | 1081 |
| 1059 | 28 | 3.448276 | 74 | 0 | 1081 |
| 1059 | 56 | 2.815485 | 74 | 0 | 1081 |
| 1059 | 168 | 0.833333 | 74 | 0 | 1081 |
| 1059 | 224 | 0.833333 | 74 | 0 | 1081 |
| 1060 | 0 | 8.888889 | 84 | 1 | 204 |
| 1060 | 21 | 13.7931 | 84 | 1 | 204 |
| 1060 | 84 | 26.96629 | 84 | 1 | 204 |
| 1060 | 161 | 3.809524 | 84 | 1 | 204 |
| 1061 | 0 | 7.023411 | 62 | 1 | 262 |
| 1061 | 28 | 5.357143 | 62 | 1 | 262 |
| 1061 | 82 | 4.363636 | 62 | 1 | 262 |
| 1061 | 194 | 3.609023 | 62 | 1 | 262 |
| 1061 | 246 | 12.58427 | 62 | 1 | 262 |
| 1062 | 0 | 2.476415 | 57 | 0 | 645 |
| 1062 | 31 | 2.364865 | 57 | 0 | 645 |
| 1062 | 94 | 2.630755 | 57 | 0 | 645 |
| 1062 | 157 | 2.548544 | 57 | 0 | 645 |
| 1062 | 245 | 2.735043 | 57 | 0 | 645 |
| 1062 | 339 | 3.017241 | 57 | 0 | 645 |
| 1062 | 427 | 3.508772 | 57 | 0 | 645 |
| 1062 | 490 | 3.448276 | 57 | 0 | 645 |
| 1062 | 581 | 2.882883 | 57 | 0 | 645 |
| 1063 | 0 | 4.363636 | 90 | 0 | 580 |
| 1063 | 28 | 5.504587 | 90 | 0 | 580 |
| 1063 | 91 | 4.485981 | 90 | 0 | 580 |
| 1063 | 147 | 5.405405 | 90 | 0 | 580 |

| | | | | | |
|---|---|---|---|---|---|
| 1063 | 260 | 6 | 90 | 0 | 580 |
| 1063 | 316 | 3.539823 | 90 | 0 | 580 |
| 1063 | 414 | 10.61947 | 90 | 0 | 580 |
| 1063 | 470 | 12.2449 | 90 | 0 | 580 |
| 1064 | 0 | 11.88119 | 75 | 1 | 408 |
| 1064 | 28 | 15.427 | 75 | 1 | 408 |
| 1064 | 74 | 15.09434 | 75 | 1 | 408 |
| 1064 | 165 | 6.722689 | 75 | 1 | 408 |
| 1064 | 256 | 27.90698 | 75 | 1 | 408 |
| 1064 | 326 | 5.263158 | 75 | 1 | 408 |
| 1064 | 386 | 37.75281 | 75 | 1 | 408 |
| 1065 | 0 | 3.361345 | 50 | 0 | 365 |
| 1065 | 28 | 3.883495 | 50 | 0 | 365 |
| 1065 | 70 | 6 | 50 | 0 | 365 |
| 1065 | 126 | 5.940594 | 50 | 0 | 365 |
| 1065 | 245 | 4.528302 | 50 | 0 | 365 |
| 1066 | 0 | 1.282051 | 89 | 0 | 1086 |
| 1066 | 28 | 0.696864 | 89 | 0 | 1086 |
| 1066 | 77 | 0.708383 | 89 | 0 | 1086 |
| 1066 | 175 | 0.721154 | 89 | 0 | 1086 |
| 1066 | 287 | 0.700935 | 89 | 0 | 1086 |
| 1066 | 357 | 1.132075 | 89 | 0 | 1086 |
| 1066 | 413 | 1.339286 | 89 | 0 | 1086 |
| 1066 | 518 | 1.648352 | 89 | 0 | 1086 |
| 1066 | 567 | 1.617251 | 89 | 0 | 1086 |
| 1067 | 0 | 1.572739 | 63 | 0 | 911 |
| 1067 | 98 | 1.490683 | 63 | 0 | 911 |
| 1067 | 154 | 1.304348 | 63 | 0 | 911 |
| 1067 | 210 | 1.834862 | 63 | 0 | 911 |
| 1067 | 322 | 1.769912 | 63 | 0 | 911 |
| 1067 | 434 | 1.724138 | 63 | 0 | 911 |
| 1067 | 490 | 1.834862 | 63 | 0 | 911 |
| 1067 | 602 | 1.769912 | 63 | 0 | 911 |
| 1068 | 0 | 10.08403 | 73 | 1 | 139 |
| 1068 | 77 | 4.259635 | 73 | 1 | 139 |
| 1069 | 0 | 2.380952 | 78 | 0 | 682 |
| 1069 | 29 | 1.935484 | 78 | 0 | 682 |
| 1069 | 99 | 2.142857 | 78 | 0 | 682 |
| 1069 | 190 | 2.051282 | 78 | 0 | 682 |

| | | | | | |
|---|---|---|---|---|---|
| 1069 | 254 | 2.777778 | 78 | 0 | 682 |
| 1069 | 358 | 3.305785 | 78 | 0 | 682 |
| 1069 | 428 | 11.53846 | 78 | 0 | 682 |
| 1069 | 513 | 5.263158 | 78 | 0 | 682 |
| 1069 | 576 | 10.34483 | 78 | 0 | 682 |
| 1070 | 0 | 4.102564 | 36 | 0 | 953 |
| 1070 | 28 | 4.102564 | 36 | 0 | 953 |
| 1070 | 63 | 4.285714 | 36 | 0 | 953 |
| 1070 | 168 | 16.66667 | 36 | 0 | 953 |
| 1070 | 217 | 17.82178 | 36 | 0 | 953 |
| 1070 | 329 | 13.84615 | 36 | 0 | 953 |
| 1070 | 427 | 15.38462 | 36 | 0 | 953 |
| 1070 | 497 | 25.26316 | 36 | 0 | 953 |
| 1070 | 581 | 18.75 | 36 | 0 | 953 |
| 1071 | 28 | 4.210526 | 83 | 0 | 785 |
| 1071 | 63 | 4.571429 | 83 | 0 | 785 |
| 1071 | 154 | 4.040404 | 83 | 0 | 785 |
| 1071 | 238 | 5.882353 | 83 | 0 | 785 |
| 1071 | 350 | 6.060606 | 83 | 0 | 785 |
| 1071 | 413 | 5.177994 | 83 | 0 | 785 |
| 1071 | 504 | 21.62162 | 83 | 0 | 785 |
| 1071 | 616 | 3.571429 | 83 | 0 | 785 |
| 1072 | 0 | 2.330097 | 79 | 0 | 986 |
| 1072 | 21 | 2.702703 | 79 | 0 | 986 |
| 1072 | 49 | 2.830189 | 79 | 0 | 986 |
| 1072 | 140 | 0.707965 | 79 | 0 | 986 |
| 1072 | 245 | 12.63158 | 79 | 0 | 986 |
| 1072 | 322 | 0.630252 | 79 | 0 | 986 |
| 1072 | 413 | 1.294498 | 79 | 0 | 986 |
| 1072 | 511 | 4.255319 | 79 | 0 | 986 |
| 1072 | 595 | 4.485981 | 79 | 0 | 986 |
| 1073 | 0 | 16.21622 | 89 | 0 | 467 |
| 1073 | 28 | 16.21622 | 89 | 0 | 467 |
| 1073 | 84 | 16.66667 | 89 | 0 | 467 |
| 1074 | 0 | 1.15942 | 80 | 0 | 745 |
| 1074 | 99 | 3.478261 | 80 | 0 | 745 |
| 1074 | 162 | 8.727273 | 80 | 0 | 745 |
| 1074 | 256 | 18.66667 | 80 | 0 | 745 |
| 1074 | 316 | 21.42857 | 80 | 0 | 745 |

| | | | | | |
|---|---|---|---|---|---|
| 1074 | 421 | 15.38462 | 80 | 0 | 745 |
| 1074 | 519 | 12.21818 | 80 | 0 | 745 |
| 1074 | 561 | 8.064516 | 80 | 0 | 745 |
| 1075 | 0 | 5.405405 | 64 | 0 | 778 |
| 1075 | 28 | 4.571429 | 64 | 0 | 778 |
| 1075 | 63 | 4.848485 | 64 | 0 | 778 |
| 1075 | 168 | 4.40367 | 64 | 0 | 778 |
| 1075 | 266 | 5.769231 | 64 | 0 | 778 |
| 1075 | 350 | 4.137931 | 64 | 0 | 778 |
| 1075 | 420 | 5.504587 | 64 | 0 | 778 |
| 1075 | 508 | 5.112599 | 64 | 0 | 778 |
| 1076 | 0 | 2.678571 | 85 | 1 | 649 |
| 1076 | 28 | 2.293202 | 85 | 1 | 649 |
| 1076 | 84 | 5.825243 | 85 | 1 | 649 |
| 1076 | 168 | 5.714286 | 85 | 1 | 649 |
| 1077 | 0 | 4.050145 | 67 | 0 | 666 |
| 1077 | 38 | 1.780038 | 67 | 0 | 666 |
| 1077 | 80 | 1.680672 | 67 | 0 | 666 |
| 1077 | 182 | 2.011494 | 67 | 0 | 666 |
| 1077 | 248 | 2.469136 | 67 | 0 | 666 |
| 1077 | 336 | 1.754386 | 67 | 0 | 666 |
| 1077 | 413 | 1.754386 | 67 | 0 | 666 |
| 1077 | 498 | 3.652174 | 67 | 0 | 666 |
| 1077 | 589 | 3.745819 | 67 | 0 | 666 |
| 1078 | 0 | 2.479339 | 77 | 0 | 1067 |
| 1078 | 28 | 2.5 | 77 | 0 | 1067 |
| 1078 | 84 | 2.608696 | 77 | 0 | 1067 |
| 1078 | 168 | 2.542373 | 77 | 0 | 1067 |
| 1078 | 252 | 2.678571 | 77 | 0 | 1067 |
| 1078 | 338 | 0.787402 | 77 | 0 | 1067 |
| 1078 | 422 | 2.678571 | 77 | 0 | 1067 |
| 1078 | 511 | 2.033898 | 77 | 0 | 1067 |
| 1078 | 581 | 2.631579 | 77 | 0 | 1067 |
| 1079 | 0 | 4.056012 | 80 | 0 | 753 |
| 1079 | 94 | 4.882302 | 80 | 0 | 753 |
| 1079 | 157 | 3.007519 | 80 | 0 | 753 |
| 1079 | 269 | 2.930403 | 80 | 0 | 753 |
| 1079 | 332 | 7.724138 | 80 | 0 | 753 |
| 1079 | 388 | 5.504587 | 80 | 0 | 753 |

| | | | | | |
|---|---|---|---|---|---|
| 1079 | 514 | 5.16129 | 80 | 0 | 753 |
| 1079 | 570 | 4.444444 | 80 | 0 | 753 |
| 1080 | 0 | 4.040404 | 74 | 1 | 521 |
| 1080 | 42 | 11.53846 | 74 | 1 | 521 |
| 1080 | 154 | 8.333333 | 74 | 1 | 521 |
| 1080 | 273 | 13.48315 | 74 | 1 | 521 |
| 1080 | 329 | 12.08198 | 74 | 1 | 521 |
| 1080 | 405 | 12.12121 | 74 | 1 | 521 |
| 1080 | 496 | 15.54117 | 74 | 1 | 521 |
| 1081 | 0 | 4.081633 | 54 | 0 | 897 |
| 1081 | 91 | 6.997085 | 54 | 0 | 897 |
| 1081 | 182 | 7.207207 | 54 | 0 | 897 |
| 1081 | 224 | 6.177606 | 54 | 0 | 897 |
| 1081 | 315 | 6.593407 | 54 | 0 | 897 |
| 1081 | 428 | 5.607477 | 54 | 0 | 897 |
| 1081 | 484 | 6.997085 | 54 | 0 | 897 |
| 1081 | 602 | 17.77778 | 54 | 0 | 897 |
| 1082 | 0 | 4.210526 | 74 | 0 | 938 |
| 1082 | 28 | 5.357143 | 74 | 0 | 938 |
| 1082 | 56 | 5.405405 | 74 | 0 | 938 |
| 1082 | 168 | 5.454545 | 74 | 0 | 938 |
| 1082 | 252 | 4.615385 | 74 | 0 | 938 |
| 1082 | 315 | 4.615385 | 74 | 0 | 938 |
| 1082 | 420 | 4.897959 | 74 | 0 | 938 |
| 1082 | 483 | 9.6 | 74 | 0 | 938 |
| 1082 | 630 | 12.63158 | 74 | 0 | 938 |
| 1083 | 0 | 3.457502 | 64 | 0 | 589 |
| 1083 | 70 | 4.571429 | 64 | 0 | 589 |
| 1083 | 168 | 4.485981 | 64 | 0 | 589 |
| 1083 | 273 | 8.571429 | 64 | 0 | 589 |
| 1083 | 329 | 10.25641 | 64 | 0 | 589 |
| 1083 | 420 | 10.16949 | 64 | 0 | 589 |
| 1083 | 511 | 11.32075 | 64 | 0 | 589 |
| 1083 | 588 | 13.71429 | 64 | 0 | 589 |
| 1084 | 0 | 2.005013 | 66 | 0 | 939 |
| 1084 | 70 | 3.418803 | 66 | 0 | 939 |
| 1084 | 161 | 3.966942 | 66 | 0 | 939 |
| 1084 | 231 | 10.3725 | 66 | 0 | 939 |
| 1084 | 329 | 8.971963 | 66 | 0 | 939 |

| | | | | | |
|---|---|---|---|---|---|
| 1084 | 399 | 8.275862 | 66 | 0 | 939 |
| 1084 | 476 | 9.836066 | 66 | 0 | 939 |
| 1084 | 553 | 7.804878 | 66 | 0 | 939 |
| 1085 | 0 | 14.4 | 65 | 0 | 1093 |
| 1085 | 14 | 15.75 | 65 | 0 | 1093 |
| 1085 | 81 | 8.163265 | 65 | 0 | 1093 |
| 1085 | 154 | 5.970149 | 65 | 0 | 1093 |
| 1085 | 245 | 17.37931 | 65 | 0 | 1093 |
| 1085 | 340 | 9.836066 | 65 | 0 | 1093 |
| 1085 | 411 | 6.060606 | 65 | 0 | 1093 |
| 1085 | 487 | 31.57895 | 65 | 0 | 1093 |
| 1086 | 21 | 13.04348 | 82 | 0 | 694 |
| 1086 | 77 | 10.81081 | 82 | 0 | 694 |
| 1086 | 175 | 9.896907 | 82 | 0 | 694 |
| 1086 | 231 | 10.52632 | 82 | 0 | 694 |
| 1086 | 322 | 10.90909 | 82 | 0 | 694 |
| 1086 | 413 | 9.142857 | 82 | 0 | 694 |
| 1086 | 476 | 12 | 82 | 0 | 694 |
| 1086 | 574 | 9.142857 | 82 | 0 | 694 |
| 1087 | 0 | 1.550388 | 71 | 1 | 575 |
| 1087 | 28 | 1.851852 | 71 | 1 | 575 |
| 1087 | 91 | 1.886792 | 71 | 1 | 575 |
| 1087 | 182 | 1.923077 | 71 | 1 | 575 |
| 1087 | 329 | 2.962963 | 71 | 1 | 575 |
| 1087 | 427 | 5.128205 | 71 | 1 | 575 |
| 1088 | 0 | 1.581325 | 80 | 0 | 406 |
| 1088 | 23 | 0.403303 | 80 | 0 | 406 |
| 1088 | 93 | 0.416419 | 80 | 0 | 406 |
| 1088 | 182 | 0.457317 | 80 | 0 | 406 |
| 1088 | 238 | 0.465632 | 80 | 0 | 406 |
| 1088 | 351 | 0.517369 | 80 | 0 | 406 |
| 1089 | 0 | 2.242991 | 72 | 0 | 797 |
| 1089 | 70 | 2.803738 | 72 | 0 | 797 |
| 1089 | 168 | 1.785714 | 72 | 0 | 797 |
| 1089 | 238 | 1.754386 | 72 | 0 | 797 |
| 1089 | 315 | 3.571429 | 72 | 0 | 797 |
| 1089 | 413 | 0.606061 | 72 | 0 | 797 |
| 1089 | 497 | 0.574713 | 72 | 0 | 797 |
| 1089 | 574 | 4.444444 | 72 | 0 | 797 |

| | | | | | |
|---|---|---|---|---|---|
| 1090 | 0 | 2.542373 | 79 | 0 | 693 |
| 1090 | 28 | 1.889764 | 79 | 0 | 693 |
| 1090 | 91 | 1.652893 | 79 | 0 | 693 |
| 1090 | 175 | 1.754386 | 79 | 0 | 693 |
| 1090 | 266 | 1.46789 | 79 | 0 | 693 |
| 1090 | 315 | 1.851852 | 79 | 0 | 693 |
| 1090 | 427 | 1.403509 | 79 | 0 | 693 |
| 1090 | 490 | 1.680672 | 79 | 0 | 693 |
| 1090 | 574 | 1.694915 | 79 | 0 | 693 |
| 1091 | 0 | 5.660377 | 58 | 1 | 568 |
| 1091 | 26 | 11.42857 | 58 | 1 | 568 |
| 1091 | 87 | 19.14894 | 58 | 1 | 568 |
| 1091 | 160 | 20.22472 | 58 | 1 | 568 |
| 1091 | 241 | 16.47059 | 58 | 1 | 568 |
| 1091 | 334 | 16.07143 | 58 | 1 | 568 |
| 1091 | 409 | 18.63905 | 58 | 1 | 568 |
| 1091 | 490 | 13.85377 | 58 | 1 | 568 |
| 1092 | 0 | 4.360993 | 60 | 0 | 357 |
| 1092 | 28 | 16.07143 | 60 | 0 | 357 |
| 1092 | 84 | 4.967475 | 60 | 0 | 357 |
| 1092 | 160 | 9.680207 | 60 | 0 | 357 |
| 1092 | 245 | 8.333333 | 60 | 0 | 357 |
| 1092 | 343 | 32.70604 | 60 | 0 | 357 |
| 1093 | 0 | 5.925926 | 77 | 0 | 733 |
| 1093 | 70 | 4.615385 | 77 | 0 | 733 |
| 1093 | 161 | 10.5 | 77 | 0 | 733 |
| 1093 | 214 | 5.555556 | 77 | 0 | 733 |
| 1093 | 326 | 5.825243 | 77 | 0 | 733 |
| 1093 | 417 | 2.339181 | 77 | 0 | 733 |
| 1093 | 522 | 3.703704 | 77 | 0 | 733 |
| 1093 | 588 | 1.248885 | 77 | 0 | 733 |
| 1094 | 0 | 1.339286 | 68 | 0 | 1058 |
| 1094 | 28 | 1.209677 | 68 | 0 | 1058 |
| 1094 | 84 | 1.694915 | 68 | 0 | 1058 |
| 1095 | 0 | 2.276423 | 48 | 1 | 366 |
| 1095 | 30 | 1.754386 | 48 | 1 | 366 |
| 1095 | 114 | 1.724138 | 48 | 1 | 366 |
| 1095 | 170 | 6.060606 | 48 | 1 | 366 |
| 1095 | 240 | 2.471315 | 48 | 1 | 366 |

| | | | | | |
|---|---|---|---|---|---|
| 1095 | 338 | 4.830362 | 48 | 1 | 366 |
| 1096 | 0 | 13.63636 | 82 | 0 | 383 |
| 1096 | 35 | 3.265306 | 82 | 0 | 383 |
| 1096 | 77 | 14.11765 | 82 | 0 | 383 |
| 1096 | 147 | 14.12952 | 82 | 0 | 383 |
| 1096 | 217 | 17.16037 | 82 | 0 | 383 |
| 1096 | 322 | 11.88119 | 82 | 0 | 383 |
| 1096 | 378 | 18.5567 | 82 | 0 | 383 |
| 1097 | 0 | 2.654867 | 70 | 1 | 93 |
| 1097 | 35 | 4.485981 | 70 | 1 | 93 |
| 1098 | 0 | 4.772727 | 52 | 1 | 269 |
| 1098 | 28 | 10.66667 | 52 | 1 | 269 |
| 1098 | 84 | 11.49425 | 52 | 1 | 269 |
| 1098 | 168 | 14.11765 | 52 | 1 | 269 |
| 1098 | 231 | 13.33333 | 52 | 1 | 269 |
| 1099 | 0 | 2.564103 | 73 | 0 | 722 |
| 1099 | 28 | 2.586207 | 73 | 0 | 722 |
| 1099 | 56 | 3.636364 | 73 | 0 | 722 |
| 1099 | 147 | 2.990654 | 73 | 0 | 722 |
| 1099 | 245 | 5.405405 | 73 | 0 | 722 |
| 1099 | 343 | 6.25 | 73 | 0 | 722 |
| 1099 | 434 | 2.242991 | 73 | 0 | 722 |
| 1099 | 504 | 11.2 | 73 | 0 | 722 |
| 1099 | 602 | 6.857143 | 73 | 0 | 722 |
| 1100 | 0 | 1.481481 | 77 | 0 | 876 |
| 1100 | 28 | 2.654867 | 77 | 0 | 876 |
| 1100 | 56 | 4.8 | 77 | 0 | 876 |
| 1100 | 161 | 4.363636 | 77 | 0 | 876 |
| 1100 | 287 | 7.142857 | 77 | 0 | 876 |
| 1100 | 413 | 10.81081 | 77 | 0 | 876 |
| 1100 | 511 | 9.52381 | 77 | 0 | 876 |
| 1100 | 574 | 11.11111 | 77 | 0 | 876 |
| 1101 | 0 | 5.660377 | 59 | 0 | 1044 |
| 1101 | 28 | 9.142857 | 59 | 0 | 1044 |
| 1101 | 91 | 8.421053 | 59 | 0 | 1044 |
| 1101 | 168 | 9.756098 | 59 | 0 | 1044 |
| 1101 | 238 | 7.017544 | 59 | 0 | 1044 |
| 1101 | 336 | 10.16949 | 59 | 0 | 1044 |
| 1101 | 427 | 8 | 59 | 0 | 1044 |

| | | | | | |
|---|---|---|---|---|---|
| 1101 | 518 | 10 | 59 | 0 | 1044 |
| 1101 | 581 | 10.08403 | 59 | 0 | 1044 |
| 1102 | 0 | 5.660377 | 68 | 0 | 1128 |
| 1102 | 28 | 5.940594 | 68 | 0 | 1128 |
| 1102 | 98 | 5.660377 | 68 | 0 | 1128 |
| 1102 | 189 | 4.948454 | 68 | 0 | 1128 |
| 1102 | 252 | 10.62619 | 68 | 0 | 1128 |
| 1102 | 350 | 8.727273 | 68 | 0 | 1128 |
| 1102 | 441 | 5.504587 | 68 | 0 | 1128 |
| 1102 | 504 | 11.11111 | 68 | 0 | 1128 |
| 1102 | 595 | 9.6 | 68 | 0 | 1128 |
| 1103 | 0 | 4.528302 | 72 | 0 | 649 |
| 1103 | 35 | 4.444444 | 72 | 0 | 649 |
| 1103 | 70 | 4.8 | 72 | 0 | 649 |
| 1103 | 175 | 4.705882 | 72 | 0 | 649 |
| 1103 | 245 | 6.153846 | 72 | 0 | 649 |
| 1104 | 0 | 5.309735 | 71 | 0 | 1100 |
| 1104 | 28 | 5 | 71 | 0 | 1100 |
| 1104 | 91 | 8.888889 | 71 | 0 | 1100 |
| 1104 | 126 | 10.25641 | 71 | 0 | 1100 |
| 1104 | 244 | 8.421053 | 71 | 0 | 1100 |
| 1104 | 307 | 10.34483 | 71 | 0 | 1100 |
| 1104 | 426 | 9.736308 | 71 | 0 | 1100 |
| 1104 | 510 | 10.34483 | 71 | 0 | 1100 |
| 1104 | 574 | 9.320388 | 71 | 0 | 1100 |
| 1105 | 0 | 1.30719 | 52 | 0 | 925 |
| 1105 | 49 | 2.330097 | 52 | 0 | 925 |
| 1105 | 168 | 3.448276 | 52 | 0 | 925 |
| 1105 | 259 | 1.714286 | 52 | 0 | 925 |
| 1105 | 315 | 1.327434 | 52 | 0 | 925 |
| 1105 | 371 | 1.294498 | 52 | 0 | 925 |
| 1105 | 504 | 1.237113 | 52 | 0 | 925 |
| 1105 | 581 | 2.160216 | 52 | 0 | 925 |
| 1106 | 0 | 1.084011 | 73 | 0 | 988 |
| 1106 | 28 | 0.672269 | 73 | 0 | 988 |
| 1106 | 63 | 0.854701 | 73 | 0 | 988 |
| 1106 | 154 | 0.589971 | 73 | 0 | 988 |
| 1106 | 252 | 0.104112 | 73 | 0 | 988 |
| 1106 | 350 | 0.363636 | 73 | 0 | 988 |

| | | | | | |
|---|---|---|---|---|---|
| 1106 | 420 | 3.448276 | 73 | 0 | 988 |
| 1106 | 483 | 1.538462 | 73 | 0 | 988 |
| 1106 | 553 | 2.526316 | 73 | 0 | 988 |
| 1107 | 0 | 17.77778 | 79 | 1 | 154 |
| 1107 | 63 | 15.78947 | 79 | 1 | 154 |
| 1107 | 153 | 0.3432 | 79 | 1 | 154 |
| 1108 | 0 | 3.809524 | 67 | 0 | 1422 |
| 1108 | 28 | 3.738318 | 67 | 0 | 1422 |
| 1108 | 98 | 2.640264 | 67 | 0 | 1422 |
| 1108 | 140 | 6.315789 | 67 | 0 | 1422 |
| 1108 | 259 | 25.35211 | 67 | 0 | 1422 |
| 1108 | 417 | 5.825243 | 67 | 0 | 1422 |
| 1108 | 515 | 4.067797 | 67 | 0 | 1422 |
| 1108 | 581 | 4.485981 | 67 | 0 | 1422 |
| 1109 | 0 | 3.2 | 63 | 0 | 1219 |
| 1109 | 28 | 2.488889 | 63 | 0 | 1219 |
| 1109 | 84 | 2.380952 | 63 | 0 | 1219 |
| 1109 | 168 | 2.727273 | 63 | 0 | 1219 |
| 1109 | 224 | 5.555556 | 63 | 0 | 1219 |
| 1109 | 336 | 4.918033 | 63 | 0 | 1219 |
| 1109 | 427 | 5.357143 | 63 | 0 | 1219 |
| 1109 | 511 | 4.918033 | 63 | 0 | 1219 |
| 1109 | 574 | 4.83871 | 63 | 0 | 1219 |
| 1110 | 0 | 2.201835 | 68 | 0 | 719 |
| 1110 | 35 | 1.851852 | 68 | 0 | 719 |
| 1110 | 77 | 0.300752 | 68 | 0 | 719 |
| 1110 | 203 | 0.289715 | 68 | 0 | 719 |
| 1110 | 273 | 0.27946 | 68 | 0 | 719 |
| 1110 | 343 | 3 | 68 | 0 | 719 |
| 1110 | 420 | 1.142857 | 68 | 0 | 719 |
| 1110 | 518 | 5.454545 | 68 | 0 | 719 |
| 1110 | 616 | 4.571429 | 68 | 0 | 719 |
| 1111 | 0 | 0.820713 | 52 | 1 | 736 |
| 1111 | 28 | 0.862689 | 52 | 1 | 736 |
| 1111 | 77 | 0.941915 | 52 | 1 | 736 |
| 1111 | 167 | 3.617571 | 52 | 1 | 736 |
| 1111 | 273 | 2.777778 | 52 | 1 | 736 |
| 1111 | 336 | 4.455446 | 52 | 1 | 736 |
| 1111 | 392 | 2.424242 | 52 | 1 | 736 |

| | | | | | |
|---|---|---|---|---|---|
| 1111 | 497 | 53.84615 | 52 | 1 | 736 |
| 1111 | 602 | 5.647692 | 52 | 1 | 736 |
| 1112 | 0 | 0.153081 | 65 | 0 | 701 |
| 1112 | 91 | 0.161355 | 65 | 0 | 701 |
| 1112 | 196 | 0.165837 | 65 | 0 | 701 |
| 1112 | 272 | 0.164316 | 65 | 0 | 701 |
| 1112 | 343 | 0.159915 | 65 | 0 | 701 |
| 1112 | 427 | 0.161355 | 65 | 0 | 701 |
| 1112 | 539 | 0.152905 | 65 | 0 | 701 |
| 1112 | 637 | 0.152905 | 65 | 0 | 701 |
| 1113 | 0 | 1.832061 | 67 | 0 | 373 |
| 1113 | 42 | 3.508772 | 67 | 0 | 373 |
| 1113 | 86 | 4.347826 | 67 | 0 | 373 |
| 1114 | 0 | 6.185567 | 60 | 1 | 359 |
| 1114 | 56 | 1.498127 | 60 | 1 | 359 |
| 1114 | 182 | 1.606426 | 60 | 1 | 359 |
| 1114 | 238 | 6.451613 | 60 | 1 | 359 |
| 1115 | 0 | 4.210526 | 61 | 1 | 578 |
| 1115 | 28 | 3.427173 | 61 | 1 | 578 |
| 1115 | 71 | 3.930284 | 61 | 1 | 578 |
| 1115 | 154 | 4.116638 | 61 | 1 | 578 |
| 1115 | 231 | 3.738318 | 61 | 1 | 578 |
| 1115 | 315 | 3.163842 | 61 | 1 | 578 |
| 1115 | 413 | 5.555556 | 61 | 1 | 578 |
| 1115 | 469 | 4.528302 | 61 | 1 | 578 |
| 1115 | 539 | 4.897959 | 61 | 1 | 578 |
| 1116 | 0 | 0.467836 | 78 | 1 | 392 |
| 1116 | 70 | 2.5 | 78 | 1 | 392 |
| 1116 | 154 | 2.631579 | 78 | 1 | 392 |
| 1116 | 238 | 6.122449 | 78 | 1 | 392 |
| 1116 | 308 | 5.504587 | 78 | 1 | 392 |
| 1117 | 0 | 1.351351 | 64 | 1 | 397 |
| 1117 | 28 | 1.081081 | 64 | 1 | 397 |
| 1117 | 91 | 1.142857 | 64 | 1 | 397 |
| 1117 | 182 | 2.830189 | 64 | 1 | 397 |
| 1117 | 259 | 1.904762 | 64 | 1 | 397 |
| 1118 | 0 | 5.048077 | 60 | 1 | 474 |
| 1118 | 82 | 5.309735 | 60 | 1 | 474 |
| 1118 | 166 | 5.185185 | 60 | 1 | 474 |

| | | | | | |
|---|---|---|---|---|---|
| 1118 | 250 | 9.056604 | 60 | 1 | 474 |
| 1118 | 313 | 11.88119 | 60 | 1 | 474 |
| 1118 | 411 | 11.88119 | 60 | 1 | 474 |
| 1119 | 0 | 11.00917 | 61 | 1 | 354 |
| 1119 | 32 | 4.8 | 61 | 1 | 354 |
| 1119 | 70 | 6.508264 | 61 | 1 | 354 |
| 1119 | 172 | 4.247788 | 61 | 1 | 354 |
| 1119 | 242 | 9.183673 | 61 | 1 | 354 |
| 1119 | 326 | 24.74227 | 61 | 1 | 354 |
| 1120 | 0 | 2.830189 | 57 | 0 | 743 |
| 1120 | 63 | 2.830189 | 57 | 0 | 743 |
| 1120 | 147 | 2.631579 | 57 | 0 | 743 |
| 1120 | 252 | 1.664355 | 57 | 0 | 743 |
| 1121 | 0 | 4.83871 | 57 | 1 | 517 |
| 1121 | 70 | 4.363636 | 57 | 1 | 517 |
| 1121 | 140 | 5.263158 | 57 | 1 | 517 |
| 1121 | 252 | 5.128205 | 57 | 1 | 517 |
| 1121 | 357 | 9.722222 | 57 | 1 | 517 |
| 1121 | 455 | 5.504587 | 57 | 1 | 517 |
| 1121 | 490 | 8.495575 | 57 | 1 | 517 |
| 1122 | 18 | 5.274725 | 82 | 0 | 939 |
| 1122 | 70 | 9.896907 | 82 | 0 | 939 |
| 1122 | 182 | 6.78925 | 82 | 0 | 939 |
| 1122 | 245 | 10 | 82 | 0 | 939 |
| 1122 | 420 | 20.45455 | 82 | 0 | 939 |
| 1122 | 500 | 9.98613 | 82 | 0 | 939 |
| 1122 | 560 | 16.21622 | 82 | 0 | 939 |
| 1123 | 0 | 4.210526 | 73 | 0 | 1285 |
| 1123 | 35 | 4.671858 | 73 | 0 | 1285 |
| 1123 | 91 | 4.210526 | 73 | 0 | 1285 |
| 1123 | 196 | 4.067797 | 73 | 0 | 1285 |
| 1123 | 252 | 3.934426 | 73 | 0 | 1285 |
| 1123 | 322 | 3.966942 | 73 | 0 | 1285 |
| 1123 | 385 | 3.448276 | 73 | 0 | 1285 |
| 1123 | 490 | 3.636364 | 73 | 0 | 1285 |
| 1123 | 574 | 7.142857 | 73 | 0 | 1285 |
| 1124 | 0 | 5.233645 | 78 | 0 | 617 |
| 1124 | 28 | 7.76699 | 78 | 0 | 617 |
| 1124 | 70 | 7.499163 | 78 | 0 | 617 |

| | | | | | |
|---|---|---|---|---|---|
| 1124 | 154 | 7.843137 | 78 | 0 | 617 |
| 1124 | 238 | 7.692308 | 78 | 0 | 617 |
| 1124 | 322 | 7.54717 | 78 | 0 | 617 |
| 1124 | 420 | 7.476636 | 78 | 0 | 617 |
| 1124 | 504 | 7.476636 | 78 | 0 | 617 |
| 1124 | 588 | 7.920792 | 78 | 0 | 617 |
| 1125 | 0 | 4.040404 | 69 | 0 | 29 |
| 1126 | 0 | 1.889339 | 89 | 0 | 712 |
| 1126 | 39 | 3.555556 | 89 | 0 | 712 |
| 1126 | 84 | 5.769231 | 89 | 0 | 712 |
| 1126 | 172 | 3.636364 | 89 | 0 | 712 |
| 1126 | 280 | 3.738318 | 89 | 0 | 712 |
| 1126 | 322 | 3.603604 | 89 | 0 | 712 |
| 1126 | 406 | 3.921569 | 89 | 0 | 712 |
| 1126 | 512 | 11.41304 | 89 | 0 | 712 |
| 1127 | 0 | 5.555556 | 73 | 0 | 763 |
| 1127 | 28 | 8.823529 | 73 | 0 | 763 |
| 1127 | 84 | 10.61947 | 73 | 0 | 763 |
| 1127 | 175 | 10.34483 | 73 | 0 | 763 |
| 1127 | 259 | 11.00917 | 73 | 0 | 763 |
| 1127 | 343 | 10.81081 | 73 | 0 | 763 |
| 1127 | 427 | 10.61947 | 73 | 0 | 763 |
| 1127 | 510 | 11.42857 | 73 | 0 | 763 |
| 1127 | 595 | 11.42857 | 73 | 0 | 763 |
| 1128 | 0 | 2.068966 | 76 | 1 | 403 |
| 1128 | 35 | 1.344538 | 76 | 1 | 403 |
| 1128 | 70 | 0.379867 | 76 | 1 | 403 |
| 1128 | 196 | 0.407747 | 76 | 1 | 403 |
| 1128 | 266 | 0.754717 | 76 | 1 | 403 |
| 1128 | 371 | 0.747664 | 76 | 1 | 403 |
| 1129 | 0 | 0.485942 | 75 | 0 | 612 |
| 1129 | 28 | 0.525131 | 75 | 0 | 612 |
| 1129 | 98 | 1.166667 | 75 | 0 | 612 |
| 1129 | 184 | 0.555556 | 75 | 0 | 612 |
| 1129 | 275 | 2.162162 | 75 | 0 | 612 |
| 1129 | 359 | 0.757576 | 75 | 0 | 612 |
| 1129 | 443 | 1.239669 | 75 | 0 | 612 |
| 1129 | 527 | 2.325581 | 75 | 0 | 612 |
| 1129 | 611 | 2.290076 | 75 | 0 | 612 |

| | | | | | |
|---|---|---|---|---|---|
| 1130 | 0 | 4.705882 | 69 | 1 | 445 |
| 1130 | 28 | 4.444444 | 69 | 1 | 445 |
| 1130 | 63 | 5.607477 | 69 | 1 | 445 |
| 1130 | 161 | 5.882353 | 69 | 1 | 445 |
| 1130 | 245 | 5.825243 | 69 | 1 | 445 |
| 1130 | 329 | 11.17393 | 69 | 1 | 445 |
| 1130 | 420 | 10.9375 | 69 | 1 | 445 |
| 1131 | 0 | 1.666667 | 53 | 1 | 363 |
| 1131 | 28 | 1.73913 | 53 | 1 | 363 |
| 1131 | 84 | 2.777778 | 53 | 1 | 363 |
| 1131 | 168 | 1.724138 | 53 | 1 | 363 |
| 1131 | 266 | 5.263158 | 53 | 1 | 363 |
| 1131 | 350 | 5.263158 | 53 | 1 | 363 |
| 1132 | 0 | 1.260504 | 85 | 1 | 535 |
| 1132 | 28 | 1.171875 | 85 | 1 | 535 |
| 1132 | 84 | 2.521008 | 85 | 1 | 535 |
| 1132 | 175 | 0.710059 | 85 | 1 | 535 |
| 1132 | 238 | 1.002028 | 85 | 1 | 535 |
| 1132 | 335 | 3.191489 | 85 | 1 | 535 |
| 1132 | 427 | 4.8 | 85 | 1 | 535 |
| 1132 | 497 | 5.263158 | 85 | 1 | 535 |
| 1133 | 0 | 3.636364 | 62 | 0 | 645 |
| 1133 | 28 | 1.481481 | 62 | 0 | 645 |
| 1133 | 91 | 6.741573 | 62 | 0 | 645 |
| 1133 | 175 | 5.555556 | 62 | 0 | 645 |
| 1133 | 252 | 6.229143 | 62 | 0 | 645 |
| 1133 | 350 | 8.495575 | 62 | 0 | 645 |
| 1133 | 427 | 6.61157 | 62 | 0 | 645 |
| 1133 | 504 | 8.944099 | 62 | 0 | 645 |
| 1133 | 595 | 6.61157 | 62 | 0 | 645 |
| 1134 | 0 | 5.128205 | 74 | 0 | 451 |
| 1134 | 28 | 4.511278 | 74 | 0 | 451 |
| 1134 | 84 | 5.172414 | 74 | 0 | 451 |
| 1134 | 168 | 5.882353 | 74 | 0 | 451 |
| 1134 | 252 | 5.825243 | 74 | 0 | 451 |
| 1134 | 343 | 5.504587 | 74 | 0 | 451 |
| 1134 | 427 | 5.607477 | 74 | 0 | 451 |
| 1135 | 0 | 2.362205 | 86 | 0 | 4 |
| 1136 | 0 | 2.777778 | 72 | 0 | 1024 |

| | | | | | |
|---|---|---|---|---|---|
| 1136 | 28 | 2.307692 | 72 | 0 | 1024 |
| 1136 | 63 | 2.181818 | 72 | 0 | 1024 |
| 1136 | 168 | 2.264151 | 72 | 0 | 1024 |
| 1136 | 231 | 2.912621 | 72 | 0 | 1024 |
| 1136 | 343 | 2.678571 | 72 | 0 | 1024 |
| 1136 | 406 | 2.376238 | 72 | 0 | 1024 |
| 1136 | 511 | 4.615385 | 72 | 0 | 1024 |
| 1136 | 581 | 4.848485 | 72 | 0 | 1024 |
| 1137 | 0 | 2.586207 | 58 | 0 | 645 |
| 1137 | 28 | 2.777778 | 58 | 0 | 645 |
| 1137 | 84 | 2.702703 | 58 | 0 | 645 |
| 1137 | 147 | 1.405152 | 58 | 0 | 645 |
| 1137 | 350 | 2.5 | 58 | 0 | 645 |
| 1137 | 427 | 2.139037 | 58 | 0 | 645 |
| 1137 | 525 | 2.586207 | 58 | 0 | 645 |
| 1137 | 581 | 2.264151 | 58 | 0 | 645 |
| 1138 | 0 | 0.649351 | 73 | 0 | 1002 |
| 1138 | 28 | 0.659341 | 73 | 0 | 1002 |
| 1138 | 84 | 3 | 73 | 0 | 1002 |
| 1138 | 168 | 1.229508 | 73 | 0 | 1002 |
| 1138 | 252 | 6.593407 | 73 | 0 | 1002 |
| 1138 | 336 | 1.347558 | 73 | 0 | 1002 |
| 1138 | 420 | 5.940594 | 73 | 0 | 1002 |
| 1138 | 504 | 1 | 73 | 0 | 1002 |
| 1138 | 574 | 6.25 | 73 | 0 | 1002 |
| 1139 | 0 | 0.237954 | 80 | 0 | 829 |
| 1139 | 42 | 0.237954 | 80 | 0 | 829 |
| 1139 | 84 | 0.243902 | 80 | 0 | 829 |
| 1139 | 161 | 0.245952 | 80 | 0 | 829 |
| 1139 | 245 | 0.266075 | 80 | 0 | 829 |
| 1140 | 0 | 10.21277 | 85 | 0 | 501 |
| 1140 | 97 | 6.021505 | 85 | 0 | 501 |
| 1140 | 168 | 8.403361 | 85 | 0 | 501 |
| 1140 | 273 | 9.036145 | 85 | 0 | 501 |
| 1140 | 336 | 16.28959 | 85 | 0 | 501 |
| 1140 | 427 | 20.84367 | 85 | 0 | 501 |
| 1141 | 0 | 2.696629 | 83 | 1 | 322 |
| 1141 | 35 | 4.938272 | 83 | 1 | 322 |
| 1141 | 85 | 11.50685 | 83 | 1 | 322 |

| | | | | | |
|---|---|---|---|---|---|
| 1141 | 161 | 5.940594 | 83 | 1 | 322 |
| 1141 | 245 | 5 | 83 | 1 | 322 |
| 1142 | 0 | 5.217391 | 77 | 1 | 56 |
| 1142 | 35 | 5.714286 | 77 | 1 | 56 |
| 1142 | 55 | 4.494382 | 77 | 1 | 56 |
| 1143 | 0 | 1.242236 | 43 | 1 | 272 |
| 1143 | 23 | 1.92 | 43 | 1 | 272 |
| 1143 | 86 | 1.860465 | 43 | 1 | 272 |
| 1143 | 149 | 2.380952 | 43 | 1 | 272 |
| 1143 | 240 | 5.714286 | 43 | 1 | 272 |
| 1144 | 0 | 11.70732 | 34 | 0 | 1142 |
| 1144 | 105 | 14.7541 | 34 | 0 | 1142 |
| 1144 | 175 | 14.87603 | 34 | 0 | 1142 |
| 1144 | 266 | 11.07692 | 34 | 0 | 1142 |
| 1144 | 322 | 11.16279 | 34 | 0 | 1142 |
| 1144 | 434 | 13.84615 | 34 | 0 | 1142 |
| 1144 | 504 | 12.30769 | 34 | 0 | 1142 |
| 1144 | 567 | 14.28571 | 34 | 0 | 1142 |
| 1145 | 0 | 3.169213 | 58 | 0 | 627 |
| 1145 | 31 | 3.478261 | 58 | 0 | 627 |
| 1145 | 87 | 2.735043 | 58 | 0 | 627 |
| 1145 | 157 | 2.93578 | 58 | 0 | 627 |
| 1145 | 255 | 2.990654 | 58 | 0 | 627 |
| 1145 | 346 | 3.738318 | 58 | 0 | 627 |
| 1145 | 430 | 4.892966 | 58 | 0 | 627 |
| 1145 | 486 | 2.059202 | 58 | 0 | 627 |
| 1146 | 11 | 2.830189 | 61 | 0 | 239 |
| 1146 | 105 | 2.058319 | 61 | 0 | 239 |
| 1147 | 0 | 3.636364 | 44 | 1 | 240 |
| 1147 | 21 | 2.608696 | 44 | 1 | 240 |
| 1147 | 84 | 2.654867 | 44 | 1 | 240 |
| 1148 | 0 | 1.046859 | 84 | 0 | 965 |
| 1148 | 28 | 1.093181 | 84 | 0 | 965 |
| 1148 | 82 | 2.803738 | 84 | 0 | 965 |
| 1148 | 166 | 2.830189 | 84 | 0 | 965 |
| 1148 | 250 | 2.181818 | 84 | 0 | 965 |
| 1148 | 341 | 9.782609 | 84 | 0 | 965 |
| 1148 | 397 | 10.32258 | 84 | 0 | 965 |
| 1148 | 572 | 21.62162 | 84 | 0 | 965 |

| | | | | | |
|---|---|---|---|---|---|
| 1149 | 0 | 1.2 | 63 | 1 | 549 |
| 1149 | 28 | 2.586207 | 63 | 1 | 549 |
| 1149 | 56 | 5.172414 | 63 | 1 | 549 |
| 1149 | 175 | 10.08403 | 63 | 1 | 549 |
| 1149 | 259 | 1.212121 | 63 | 1 | 549 |
| 1150 | 0 | 2.912621 | 35 | 0 | 274 |
| 1150 | 28 | 2.752294 | 35 | 0 | 274 |
| 1150 | 84 | 2.264151 | 35 | 0 | 274 |
| 1150 | 161 | 2.912621 | 35 | 0 | 274 |
| 1150 | 252 | 3.773585 | 35 | 0 | 274 |
| 1151 | 0 | 0.498931 | 83 | 0 | 673 |
| 1151 | 28 | 0.323887 | 83 | 0 | 673 |
| 1151 | 91 | 0.301659 | 83 | 0 | 673 |
| 1151 | 147 | 0.943396 | 83 | 0 | 673 |
| 1151 | 266 | 0.786627 | 83 | 0 | 673 |
| 1151 | 329 | 4.081633 | 83 | 0 | 673 |
| 1151 | 392 | 1.242236 | 83 | 0 | 673 |
| 1151 | 511 | 1.661475 | 83 | 0 | 673 |
| 1151 | 567 | 1.769912 | 83 | 0 | 673 |
| 1152 | 0 | 4.444444 | 67 | 1 | 429 |
| 1152 | 63 | 5.714286 | 67 | 1 | 429 |
| 1152 | 154 | 4.752475 | 67 | 1 | 429 |
| 1152 | 245 | 6.25 | 67 | 1 | 429 |
| 1152 | 301 | 5.769231 | 67 | 1 | 429 |
| 1152 | 399 | 13.7931 | 67 | 1 | 429 |
| 1153 | 0 | 8.571429 | 79 | 0 | 820 |
| 1153 | 112 | 15.12605 | 79 | 0 | 820 |
| 1153 | 168 | 4.83871 | 79 | 0 | 820 |
| 1153 | 280 | 5.504587 | 79 | 0 | 820 |
| 1153 | 336 | 2.608696 | 79 | 0 | 820 |
| 1153 | 448 | 2.631579 | 79 | 0 | 820 |
| 1154 | 0 | 12.76596 | 77 | 0 | 1 |
| 1155 | 0 | 0.673401 | 80 | 1 | 358 |
| 1155 | 91 | 3.529412 | 80 | 1 | 358 |
| 1155 | 147 | 6.480648 | 80 | 1 | 358 |
| 1155 | 224 | 5.081157 | 80 | 1 | 358 |
| 1155 | 315 | 5.377147 | 80 | 1 | 358 |
| 1156 | 0 | 8.411215 | 62 | 1 | 915 |
| 1156 | 56 | 3.283174 | 62 | 1 | 915 |

| | | | | | |
|---|---|---|---|---|---|
| 1156 | 175 | 4.758757 | 62 | 1 | 915 |
| 1157 | 0 | 12.5 | 39 | 0 | 667 |
| 1157 | 32 | 4.40367 | 39 | 0 | 667 |
| 1157 | 102 | 8.807339 | 39 | 0 | 667 |
| 1157 | 175 | 9.022556 | 39 | 0 | 667 |
| 1157 | 266 | 5.765271 | 39 | 0 | 667 |
| 1157 | 357 | 2.424242 | 39 | 0 | 667 |
| 1157 | 427 | 3.870968 | 39 | 0 | 667 |
| 1157 | 518 | 5.714286 | 39 | 0 | 667 |
| 1157 | 609 | 4.660194 | 39 | 0 | 667 |
| 1158 | 0 | 1.760176 | 81 | 0 | 582 |
| 1158 | 35 | 1.843318 | 81 | 0 | 582 |
| 1158 | 133 | 0.922722 | 81 | 0 | 582 |
| 1158 | 252 | 0.8329 | 81 | 0 | 582 |
| 1158 | 336 | 0.57971 | 81 | 0 | 582 |
| 1158 | 413 | 0.688765 | 81 | 0 | 582 |
| 1159 | 0 | 4.040404 | 56 | 0 | 659 |
| 1159 | 28 | 3.883495 | 56 | 0 | 659 |
| 1159 | 84 | 5.940594 | 56 | 0 | 659 |
| 1159 | 175 | 9.411765 | 56 | 0 | 659 |
| 1159 | 238 | 18 | 56 | 0 | 659 |
| 1159 | 322 | 4.918033 | 56 | 0 | 659 |
| 1159 | 406 | 11.65049 | 56 | 0 | 659 |
| 1159 | 462 | 11.21495 | 56 | 0 | 659 |
| 1159 | 574 | 5.607477 | 56 | 0 | 659 |
| 1160 | 0 | 4.019139 | 90 | 0 | 707 |
| 1160 | 70 | 7.272727 | 90 | 0 | 707 |
| 1160 | 182 | 5.504587 | 90 | 0 | 707 |
| 1160 | 238 | 3.571429 | 90 | 0 | 707 |
| 1160 | 301 | 7.843137 | 90 | 0 | 707 |
| 1161 | 0 | 0.816882 | 65 | 0 | 981 |
| 1161 | 28 | 0.831601 | 65 | 0 | 981 |
| 1161 | 175 | 2.222222 | 65 | 0 | 981 |
| 1161 | 266 | 2.727273 | 65 | 0 | 981 |
| 1161 | 308 | 1.834862 | 65 | 0 | 981 |
| 1161 | 392 | 3.773585 | 65 | 0 | 981 |
| 1161 | 476 | 7.207207 | 65 | 0 | 981 |
| 1161 | 560 | 10.71429 | 65 | 0 | 981 |
| 1162 | 0 | 2.777778 | 77 | 1 | 415 |

| | | | | | |
|---|---|---|---|---|---|
| 1162 | 42 | 6.382979 | 77 | 1 | 415 |
| 1162 | 98 | 4.752475 | 77 | 1 | 415 |
| 1162 | 168 | 11.32075 | 77 | 1 | 415 |
| 1162 | 266 | 10.10526 | 77 | 1 | 415 |
| 1162 | 336 | 19.35484 | 77 | 1 | 415 |
| 1163 | 0 | 5.172414 | 72 | 0 | 1247 |
| 1163 | 28 | 3.174603 | 72 | 0 | 1247 |
| 1163 | 84 | 2.5 | 72 | 0 | 1247 |
| 1163 | 175 | 2.586207 | 72 | 0 | 1247 |
| 1163 | 252 | 3.478261 | 72 | 0 | 1247 |
| 1163 | 336 | 14.15929 | 72 | 0 | 1247 |
| 1163 | 392 | 4.8 | 72 | 0 | 1247 |
| 1163 | 497 | 5.357143 | 72 | 0 | 1247 |
| 1163 | 581 | 11.21495 | 72 | 0 | 1247 |
| 1164 | 0 | 3.658537 | 69 | 1 | 219 |
| 1164 | 28 | 3.855422 | 69 | 1 | 219 |
| 1164 | 63 | 1.428571 | 69 | 1 | 219 |
| 1164 | 154 | 1.410935 | 69 | 1 | 219 |
| 1164 | 210 | 1.235658 | 69 | 1 | 219 |
| 1165 | 0 | 1.617251 | 73 | 0 | 820 |
| 1165 | 49 | 1.327434 | 73 | 0 | 820 |
| 1165 | 161 | 0.254507 | 73 | 0 | 820 |
| 1165 | 224 | 0.268516 | 73 | 0 | 820 |
| 1165 | 301 | 0.273535 | 73 | 0 | 820 |
| 1165 | 392 | 0.713012 | 73 | 0 | 820 |
| 1165 | 525 | 0.917431 | 73 | 0 | 820 |
| 1165 | 588 | 0.990099 | 73 | 0 | 820 |
| 1166 | 0 | 0.625 | 83 | 0 | 889 |
| 1166 | 28 | 1.510248 | 83 | 0 | 889 |
| 1166 | 105 | 2.991453 | 83 | 0 | 889 |
| 1167 | 0 | 3.571429 | 91 | 0 | 1 |
| 1168 | 0 | 6.382979 | 80 | 0 | 326 |
| 1168 | 28 | 6.818182 | 80 | 0 | 326 |
| 1168 | 84 | 10.32258 | 80 | 0 | 326 |
| 1168 | 182 | 8.971963 | 80 | 0 | 326 |
| 1168 | 238 | 15 | 80 | 0 | 326 |
| 1168 | 316 | 10.18182 | 80 | 0 | 326 |
| 1169 | 0 | 13.84615 | 81 | 0 | 1149 |
| 1169 | 66 | 11.52 | 81 | 0 | 1149 |

| | | | | | |
|---|---|---|---|---|---|
| 1169 | 157 | 16.51376 | 81 | 0 | 1149 |
| 1170 | 0 | 2.307692 | 73 | 0 | 851 |
| 1170 | 31 | 1.025641 | 73 | 0 | 851 |
| 1170 | 88 | 1.37931 | 73 | 0 | 851 |
| 1170 | 161 | 1.108801 | 73 | 0 | 851 |
| 1170 | 252 | 1.869159 | 73 | 0 | 851 |
| 1170 | 336 | 3.773585 | 73 | 0 | 851 |
| 1170 | 420 | 3.278689 | 73 | 0 | 851 |
| 1170 | 504 | 2.953846 | 73 | 0 | 851 |
| 1170 | 595 | 3.051494 | 73 | 0 | 851 |
| 1171 | 0 | 5.769231 | 83 | 0 | 1196 |
| 1171 | 26 | 5.940594 | 83 | 0 | 1196 |
| 1171 | 54 | 6.060606 | 83 | 0 | 1196 |
| 1171 | 182 | 5.660377 | 83 | 0 | 1196 |
| 1171 | 266 | 21.05263 | 83 | 0 | 1196 |
| 1171 | 329 | 9.836066 | 83 | 0 | 1196 |
| 1171 | 420 | 9.230769 | 83 | 0 | 1196 |
| 1171 | 483 | 9.235844 | 83 | 0 | 1196 |
| 1171 | 579 | 12.12121 | 83 | 0 | 1196 |
| 1172 | 0 | 0.393185 | 86 | 0 | 660 |
| 1172 | 28 | 0.332226 | 86 | 0 | 660 |
| 1172 | 84 | 0.363196 | 86 | 0 | 660 |
| 1172 | 168 | 0.311526 | 86 | 0 | 660 |
| 1172 | 252 | 0.320513 | 86 | 0 | 660 |
| 1172 | 336 | 0.584795 | 86 | 0 | 660 |
| 1172 | 420 | 1.129944 | 86 | 0 | 660 |
| 1173 | 0 | 3.539823 | 65 | 1 | 190 |
| 1173 | 105 | 4.615385 | 65 | 1 | 190 |
| 1173 | 186 | 3.723404 | 65 | 1 | 190 |
| 1174 | 0 | 10.52632 | 79 | 0 | 67 |
| 1174 | 28 | 16.90141 | 79 | 0 | 67 |
| 1175 | 0 | 5.410628 | 68 | 1 | 390 |
| 1175 | 27 | 2.393844 | 68 | 1 | 390 |
| 1175 | 56 | 5.660377 | 68 | 1 | 390 |
| 1175 | 168 | 11.00917 | 68 | 1 | 390 |
| 1175 | 224 | 14.15929 | 68 | 1 | 390 |
| 1175 | 308 | 9.917355 | 68 | 1 | 390 |
| 1175 | 385 | 19.2 | 68 | 1 | 390 |
| 1176 | 0 | 11.32075 | 89 | 0 | 429 |

| | | | | | |
|---|---|---|---|---|---|
| 1176 | 81 | 5.377147 | 89 | 0 | 429 |
| 1176 | 161 | 7.42268 | 89 | 0 | 429 |
| 1176 | 245 | 13.63636 | 89 | 0 | 429 |
| 1176 | 329 | 17.14286 | 89 | 0 | 429 |
| 1176 | 399 | 20 | 89 | 0 | 429 |
| 1177 | 0 | 6.326492 | 78 | 0 | 1282 |
| 1177 | 124 | 8.571429 | 78 | 0 | 1282 |
| 1177 | 187 | 9.142857 | 78 | 0 | 1282 |
| 1177 | 250 | 15.11244 | 78 | 0 | 1282 |
| 1177 | 364 | 16.31068 | 78 | 0 | 1282 |
| 1177 | 518 | 16.36364 | 78 | 0 | 1282 |
| 1177 | 582 | 16.39558 | 78 | 0 | 1282 |
| 1178 | 0 | 2.359882 | 72 | 1 | 604 |
| 1178 | 21 | 0.503145 | 72 | 1 | 604 |
| 1178 | 42 | 0.467836 | 72 | 1 | 604 |
| 1178 | 126 | 0.733945 | 72 | 1 | 604 |
| 1178 | 227 | 1.156786 | 72 | 1 | 604 |
| 1178 | 360 | 1.073414 | 72 | 1 | 604 |
| 1178 | 427 | 3.211009 | 72 | 1 | 604 |
| 1179 | 0 | 3.669725 | 74 | 1 | 664 |
| 1179 | 28 | 3.773585 | 74 | 1 | 664 |
| 1179 | 84 | 5.714286 | 74 | 1 | 664 |
| 1179 | 172 | 4.444444 | 74 | 1 | 664 |
| 1179 | 266 | 5.607477 | 74 | 1 | 664 |
| 1179 | 350 | 5.660377 | 74 | 1 | 664 |
| 1180 | 0 | 0.721154 | 66 | 0 | 862 |
| 1180 | 28 | 0.678733 | 66 | 0 | 862 |
| 1180 | 91 | 0.940439 | 66 | 0 | 862 |
| 1180 | 168 | 1.246106 | 66 | 0 | 862 |
| 1180 | 252 | 2.181818 | 66 | 0 | 862 |
| 1180 | 329 | 3.767661 | 66 | 0 | 862 |
| 1180 | 420 | 3.571429 | 66 | 0 | 862 |
| 1180 | 504 | 5.714286 | 66 | 0 | 862 |
| 1180 | 588 | 5.084746 | 66 | 0 | 862 |
| 1181 | 0 | 7.843137 | 80 | 0 | 813 |
| 1181 | 42 | 7.017544 | 80 | 0 | 813 |
| 1181 | 105 | 6.779661 | 80 | 0 | 813 |
| 1181 | 168 | 5.555556 | 80 | 0 | 813 |
| 1181 | 245 | 11.00917 | 80 | 0 | 813 |

| | | | | | |
|---|---|---|---|---|---|
| 1181 | 350 | 14.03509 | 80 | 0 | 813 |
| 1181 | 406 | 9.677419 | 80 | 0 | 813 |
| 1181 | 525 | 10.34483 | 80 | 0 | 813 |
| 1181 | 595 | 12.5 | 80 | 0 | 813 |
| 1182 | 0 | 10.61947 | 78 | 0 | 1013 |
| 1182 | 42 | 14.71963 | 78 | 0 | 1013 |
| 1182 | 74 | 12.74336 | 78 | 0 | 1013 |
| 1182 | 172 | 12 | 78 | 0 | 1013 |
| 1182 | 284 | 11.33603 | 78 | 0 | 1013 |
| 1182 | 326 | 12.74336 | 78 | 0 | 1013 |
| 1182 | 434 | 12.63158 | 78 | 0 | 1013 |
| 1182 | 529 | 12.97297 | 78 | 0 | 1013 |
| 1182 | 613 | 8.716707 | 78 | 0 | 1013 |
| 1183 | 0 | 3.448276 | 76 | 0 | 694 |
| 1183 | 42 | 2.881152 | 76 | 0 | 694 |
| 1183 | 168 | 2.11827 | 76 | 0 | 694 |
| 1183 | 217 | 2.727273 | 76 | 0 | 694 |
| 1183 | 329 | 2.203857 | 76 | 0 | 694 |
| 1183 | 392 | 2.380952 | 76 | 0 | 694 |
| 1183 | 455 | 2.446483 | 76 | 0 | 694 |
| 1183 | 581 | 8.421053 | 76 | 0 | 694 |
| 1184 | 0 | 1.538462 | 61 | 1 | 580 |
| 1184 | 35 | 2.447552 | 61 | 1 | 580 |
| 1184 | 84 | 2.242991 | 61 | 1 | 580 |
| 1184 | 168 | 2.830189 | 61 | 1 | 580 |
| 1184 | 259 | 7.76699 | 61 | 1 | 580 |
| 1184 | 329 | 10.90909 | 61 | 1 | 580 |
| 1184 | 406 | 10 | 61 | 1 | 580 |
| 1184 | 490 | 7.142857 | 61 | 1 | 580 |
| 1184 | 574 | 5.555556 | 61 | 1 | 580 |
| 1185 | 14 | 2.542373 | 67 | 0 | 708 |
| 1185 | 70 | 0.569801 | 67 | 0 | 708 |
| 1185 | 161 | 0.57971 | 67 | 0 | 708 |
| 1185 | 252 | 5.555556 | 67 | 0 | 708 |
| 1185 | 308 | 2.142857 | 67 | 0 | 708 |
| 1185 | 399 | 3.611111 | 67 | 0 | 708 |
| 1185 | 490 | 2.962963 | 67 | 0 | 708 |
| 1185 | 581 | 8.163265 | 67 | 0 | 708 |
| 1186 | 0 | 5 | 77 | 1 | 957 |

| | | | | | |
|---|---|---|---|---|---|
| 1186 | 28 | 2.4 | 77 | 1 | 957 |
| 1186 | 84 | 4.95283 | 77 | 1 | 957 |
| 1186 | 217 | 3.478261 | 77 | 1 | 957 |
| 1186 | 308 | 3.061224 | 77 | 1 | 957 |
| 1186 | 406 | 3.061224 | 77 | 1 | 957 |
| 1186 | 504 | 3.265306 | 77 | 1 | 957 |
| 1186 | 602 | 3.174603 | 77 | 1 | 957 |
| 1187 | 0 | 5.555556 | 64 | 1 | 263 |
| 1187 | 28 | 5.454545 | 64 | 1 | 263 |
| 1187 | 84 | 3.437164 | 64 | 1 | 263 |
| 1187 | 168 | 5.555556 | 64 | 1 | 263 |
| 1187 | 257 | 7.526882 | 64 | 1 | 263 |
| 1188 | 0 | 2.362205 | 68 | 0 | 621 |
| 1188 | 28 | 2.255639 | 68 | 0 | 621 |
| 1188 | 84 | 5.504587 | 68 | 0 | 621 |
| 1188 | 168 | 2.31405 | 68 | 0 | 621 |
| 1188 | 256 | 5.309735 | 68 | 0 | 621 |
| 1188 | 340 | 5.309735 | 68 | 0 | 621 |
| 1188 | 424 | 2.586207 | 68 | 0 | 621 |
| 1188 | 508 | 5.172414 | 68 | 0 | 621 |
| 1188 | 592 | 5.309735 | 68 | 0 | 621 |
| 1189 | 0 | 2.521008 | 86 | 0 | 1025 |
| 1189 | 28 | 2.393162 | 86 | 0 | 1025 |
| 1189 | 86 | 2.564103 | 86 | 0 | 1025 |
| 1189 | 168 | 2.439024 | 86 | 0 | 1025 |
| 1189 | 252 | 2.459016 | 86 | 0 | 1025 |
| 1189 | 336 | 2.459016 | 86 | 0 | 1025 |
| 1189 | 427 | 2.542373 | 86 | 0 | 1025 |
| 1189 | 485 | 2.5 | 86 | 0 | 1025 |
| 1189 | 595 | 2.785146 | 86 | 0 | 1025 |
| 1190 | 0 | 1.5625 | 73 | 0 | 848 |
| 1190 | 84 | 1.30719 | 73 | 0 | 848 |
| 1190 | 168 | 1.941748 | 73 | 0 | 848 |
| 1190 | 252 | 4.040404 | 73 | 0 | 848 |
| 1190 | 336 | 6 | 73 | 0 | 848 |
| 1190 | 420 | 5.940594 | 73 | 0 | 848 |
| 1190 | 504 | 6.545455 | 73 | 0 | 848 |
| 1190 | 581 | 6.417112 | 73 | 0 | 848 |
| 1191 | 0 | 6.122449 | 84 | 0 | 1156 |

| | | | | | |
|---|---|---|---|---|---|
| 1191 | 28 | 5.314774 | 84 | 0 | 1156 |
| 1191 | 84 | 2.752294 | 84 | 0 | 1156 |
| 1191 | 168 | 5.714286 | 84 | 0 | 1156 |
| 1191 | 252 | 5.504587 | 84 | 0 | 1156 |
| 1191 | 343 | 5.504587 | 84 | 0 | 1156 |
| 1191 | 427 | 2.542373 | 84 | 0 | 1156 |
| 1191 | 497 | 5.882353 | 84 | 0 | 1156 |
| 1191 | 581 | 5.263158 | 84 | 0 | 1156 |
| 1192 | 0 | 2.782609 | 81 | 0 | 1044 |
| 1192 | 35 | 1.333333 | 81 | 0 | 1044 |
| 1192 | 105 | 3.960396 | 81 | 0 | 1044 |
| 1192 | 203 | 2.711864 | 81 | 0 | 1044 |
| 1192 | 287 | 0.909091 | 81 | 0 | 1044 |
| 1193 | 0 | 4.48 | 67 | 1 | 527 |
| 1193 | 30 | 5.217391 | 67 | 1 | 527 |
| 1193 | 86 | 4.063861 | 67 | 1 | 527 |
| 1193 | 415 | 8.96 | 67 | 1 | 527 |
| 1193 | 525 | 7.342657 | 67 | 1 | 527 |
| 1194 | 0 | 9.677419 | 69 | 1 | 330 |
| 1194 | 28 | 8.910891 | 69 | 1 | 330 |
| 1194 | 84 | 12 | 69 | 1 | 330 |
| 1194 | 161 | 5.180389 | 69 | 1 | 330 |
| 1194 | 252 | 4.582651 | 69 | 1 | 330 |
| 1194 | 315 | 56 | 69 | 1 | 330 |
| 1195 | 0 | 0.708502 | 78 | 1 | 62 |
| 1195 | 20 | 3.883495 | 78 | 1 | 62 |
| 1196 | 0 | 18.18182 | 52 | 1 | 155 |
| 1196 | 28 | 15.38462 | 52 | 1 | 155 |
| 1196 | 77 | 12.41379 | 52 | 1 | 155 |
| 1197 | 0 | 2.727273 | 70 | 0 | 763 |
| 1197 | 28 | 2.752294 | 70 | 0 | 763 |
| 1197 | 84 | 2.631579 | 70 | 0 | 763 |
| 1197 | 168 | 2.380952 | 70 | 0 | 763 |
| 1197 | 273 | 1.465201 | 70 | 0 | 763 |
| 1197 | 315 | 1.886792 | 70 | 0 | 763 |
| 1198 | 0 | 1.875 | 70 | 1 | 275 |
| 1198 | 13 | 4.955752 | 70 | 1 | 275 |
| 1198 | 98 | 1.680672 | 70 | 1 | 275 |
| 1198 | 153 | 1.092896 | 70 | 1 | 275 |

| | | | | | |
|---|---|---|---|---|---|
| 1198 | 259 | 1.284109 | 70 | 1 | 275 |
| 1199 | 0 | 2.335929 | 79 | 0 | 1044 |
| 1199 | 29 | 2.592593 | 79 | 0 | 1044 |
| 1199 | 56 | 2.051282 | 79 | 0 | 1044 |
| 1199 | 175 | 1.983471 | 79 | 0 | 1044 |
| 1199 | 238 | 1.818182 | 79 | 0 | 1044 |
| 1199 | 322 | 2.777778 | 79 | 0 | 1044 |
| 1199 | 406 | 1.282051 | 79 | 0 | 1044 |
| 1199 | 504 | 2.307692 | 79 | 0 | 1044 |
| 1199 | 574 | 1.37931 | 79 | 0 | 1044 |
| 1200 | 0 | 1.6 | 80 | 0 | 815 |
| 1201 | 0 | 3.076923 | 72 | 0 | 897 |
| 1201 | 35 | 4.660194 | 72 | 0 | 897 |
| 1201 | 91 | 10.90909 | 72 | 0 | 897 |
| 1201 | 154 | 10.81081 | 72 | 0 | 897 |
| 1201 | 293 | 5.263158 | 72 | 0 | 897 |
| 1201 | 343 | 2.702703 | 72 | 0 | 897 |
| 1201 | 399 | 2.912621 | 72 | 0 | 897 |
| 1202 | 0 | 5.504587 | 76 | 0 | 672 |
| 1202 | 91 | 3.246377 | 76 | 0 | 672 |
| 1202 | 161 | 4.571429 | 76 | 0 | 672 |
| 1202 | 252 | 4.948454 | 76 | 0 | 672 |
| 1202 | 315 | 6 | 76 | 0 | 672 |
| 1202 | 427 | 6.122449 | 76 | 0 | 672 |
| 1202 | 497 | 10.10526 | 76 | 0 | 672 |
| 1202 | 637 | 14.25743 | 76 | 0 | 672 |
| 1203 | 0 | 2.727273 | 65 | 0 | 1072 |
| 1203 | 28 | 2.803738 | 65 | 0 | 1072 |
| 1203 | 84 | 2.654867 | 65 | 0 | 1072 |
| 1203 | 168 | 2.330097 | 65 | 0 | 1072 |
| 1203 | 266 | 2.285714 | 65 | 0 | 1072 |
| 1203 | 336 | 3 | 65 | 0 | 1072 |
| 1203 | 420 | 2.941176 | 65 | 0 | 1072 |
| 1203 | 476 | 2.330097 | 65 | 0 | 1072 |
| 1203 | 609 | 2.376238 | 65 | 0 | 1072 |
| 1204 | 0 | 18.5567 | 78 | 0 | 316 |
| 1204 | 28 | 18.36735 | 78 | 0 | 316 |
| 1204 | 84 | 14.11765 | 78 | 0 | 316 |
| 1204 | 163 | 8.510638 | 78 | 0 | 316 |

| | | | | | |
|---|---|---|---|---|---|
| 1204 | 252 | 16 | 78 | 0 | 316 |
| 1204 | 315 | 18.94737 | 78 | 0 | 316 |
| 1205 | 0 | 1.544402 | 57 | 0 | 736 |
| 1205 | 91 | 1.869159 | 57 | 0 | 736 |
| 1205 | 140 | 1.544402 | 57 | 0 | 736 |
| 1205 | 245 | 1.661475 | 57 | 0 | 736 |
| 1205 | 308 | 1.601602 | 57 | 0 | 736 |
| 1205 | 434 | 1.573255 | 57 | 0 | 736 |
| 1205 | 497 | 1.661475 | 57 | 0 | 736 |
| 1205 | 560 | 1.661475 | 57 | 0 | 736 |
| 1206 | 0 | 3.738318 | 75 | 0 | 989 |
| 1206 | 28 | 3.738318 | 75 | 0 | 989 |
| 1206 | 91 | 3.106796 | 75 | 0 | 989 |
| 1206 | 182 | 8.080808 | 75 | 0 | 989 |
| 1206 | 245 | 8.849558 | 75 | 0 | 989 |
| 1206 | 336 | 12.2449 | 75 | 0 | 989 |
| 1206 | 420 | 10.90909 | 75 | 0 | 989 |
| 1206 | 539 | 11.21495 | 75 | 0 | 989 |
| 1206 | 602 | 13.33333 | 75 | 0 | 989 |
| 1207 | 0 | 5.660377 | 62 | 1 | 471 |
| 1207 | 28 | 5.825243 | 62 | 1 | 471 |
| 1207 | 84 | 6.060606 | 62 | 1 | 471 |
| 1207 | 175 | 11.65049 | 62 | 1 | 471 |
| 1207 | 283 | 6.451613 | 62 | 1 | 471 |
| 1207 | 353 | 5.769231 | 62 | 1 | 471 |
| 1207 | 441 | 16.66667 | 62 | 1 | 471 |
| 1207 | 469 | 12.44444 | 62 | 1 | 471 |
| 1208 | 0 | 2.263083 | 80 | 0 | 210 |
| 1208 | 98 | 2.308802 | 80 | 0 | 210 |
| 1209 | 0 | 5.309735 | 55 | 0 | 595 |
| 1209 | 28 | 3.756708 | 55 | 0 | 595 |
| 1209 | 98 | 5.363985 | 55 | 0 | 595 |
| 1209 | 189 | 5.128205 | 55 | 0 | 595 |
| 1209 | 251 | 5.6 | 55 | 0 | 595 |
| 1209 | 335 | 4.745763 | 55 | 0 | 595 |
| 1209 | 426 | 5.357143 | 55 | 0 | 595 |
| 1209 | 506 | 4.442094 | 55 | 0 | 595 |
| 1210 | 0 | 3.703704 | 72 | 0 | 584 |
| 1210 | 72 | 5.309735 | 72 | 0 | 584 |

| | | | | | |
|---|---|---|---|---|---|
| 1210 | 162 | 12 | 72 | 0 | 584 |
| 1210 | 247 | 5.128205 | 72 | 0 | 584 |
| 1210 | 336 | 6.666667 | 72 | 0 | 584 |
| 1210 | 406 | 6.315789 | 72 | 0 | 584 |
| 1210 | 492 | 3.84498 | 72 | 0 | 584 |
| 1210 | 583 | 8.181818 | 72 | 0 | 584 |
| 1211 | 0 | 5.660377 | 79 | 0 | 1010 |
| 1211 | 77 | 8.807339 | 79 | 0 | 1010 |
| 1211 | 168 | 6.857143 | 79 | 0 | 1010 |
| 1211 | 245 | 11.53846 | 79 | 0 | 1010 |
| 1211 | 315 | 8.648649 | 79 | 0 | 1010 |
| 1211 | 427 | 9.6 | 79 | 0 | 1010 |
| 1211 | 496 | 14.54545 | 79 | 0 | 1010 |
| 1211 | 567 | 10.71429 | 79 | 0 | 1010 |
| 1212 | 0 | 2.016807 | 69 | 0 | 769 |
| 1212 | 19 | 1.239669 | 69 | 0 | 769 |
| 1212 | 75 | 2.521008 | 69 | 0 | 769 |
| 1212 | 138 | 3.508772 | 69 | 0 | 769 |
| 1212 | 229 | 3.060109 | 69 | 0 | 769 |
| 1212 | 322 | 2.359882 | 69 | 0 | 769 |
| 1212 | 399 | 2.601626 | 69 | 0 | 769 |
| 1212 | 476 | 3.305785 | 69 | 0 | 769 |
| 1212 | 574 | 3.305785 | 69 | 0 | 769 |
| 1213 | 0 | 29.37063 | 67 | 0 | 258 |
| 1213 | 41 | 27.27273 | 67 | 0 | 258 |
| 1213 | 97 | 27.69231 | 67 | 0 | 258 |
| 1213 | 181 | 30 | 67 | 0 | 258 |
| 1213 | 244 | 27.90698 | 67 | 0 | 258 |
| 1214 | 0 | 2.439024 | 58 | 0 | 750 |
| 1215 | 0 | 4.40367 | 69 | 0 | 923 |
| 1215 | 28 | 4.40367 | 69 | 0 | 923 |
| 1215 | 91 | 5.607477 | 69 | 0 | 923 |
| 1215 | 161 | 7.272727 | 69 | 0 | 923 |
| 1215 | 238 | 4.444444 | 69 | 0 | 923 |
| 1215 | 343 | 5.490196 | 69 | 0 | 923 |
| 1215 | 413 | 4.705882 | 69 | 0 | 923 |
| 1215 | 483 | 5.280528 | 69 | 0 | 923 |
| 1215 | 581 | 9.056604 | 69 | 0 | 923 |
| 1216 | 0 | 3.361345 | 84 | 0 | 1002 |

| | | | | | |
|---|---|---|---|---|---|
| 1216 | 28 | 1.694915 | 84 | 0 | 1002 |
| 1216 | 84 | 1.818182 | 84 | 0 | 1002 |
| 1216 | 168 | 3.883495 | 84 | 0 | 1002 |
| 1216 | 252 | 3.636364 | 84 | 0 | 1002 |
| 1216 | 336 | 3.703704 | 84 | 0 | 1002 |
| 1216 | 420 | 6.480648 | 84 | 0 | 1002 |
| 1216 | 497 | 4.945055 | 84 | 0 | 1002 |
| 1216 | 588 | 5.128205 | 84 | 0 | 1002 |
| 1217 | 0 | 2.666667 | 70 | 0 | 946 |
| 1217 | 35 | 4.615385 | 70 | 0 | 946 |
| 1217 | 70 | 8.333333 | 70 | 0 | 946 |
| 1217 | 154 | 3.966942 | 70 | 0 | 946 |
| 1217 | 252 | 3.508772 | 70 | 0 | 946 |
| 1217 | 343 | 1.626016 | 70 | 0 | 946 |
| 1217 | 399 | 1.481481 | 70 | 0 | 946 |
| 1217 | 518 | 1.344538 | 70 | 0 | 946 |
| 1217 | 617 | 1.481481 | 70 | 0 | 946 |
| 1218 | 0 | 3.333333 | 77 | 0 | 806 |
| 1218 | 28 | 9.431579 | 77 | 0 | 806 |
| 1218 | 81 | 11.42857 | 77 | 0 | 806 |
| 1218 | 175 | 10.42184 | 77 | 0 | 806 |
| 1218 | 252 | 2.222222 | 77 | 0 | 806 |
| 1218 | 336 | 2.330097 | 77 | 0 | 806 |
| 1218 | 417 | 4.48 | 77 | 0 | 806 |
| 1218 | 504 | 5.309735 | 77 | 0 | 806 |
| 1218 | 560 | 5.454545 | 77 | 0 | 806 |
| 1219 | 0 | 5.405405 | 67 | 0 | 694 |
| 1219 | 28 | 5.607477 | 67 | 0 | 694 |
| 1219 | 84 | 8.256881 | 67 | 0 | 694 |
| 1219 | 168 | 10.43478 | 67 | 0 | 694 |
| 1219 | 252 | 11.00917 | 67 | 0 | 694 |
| 1219 | 336 | 10.90909 | 67 | 0 | 694 |
| 1219 | 420 | 10.71429 | 67 | 0 | 694 |
| 1219 | 476 | 17.82178 | 67 | 0 | 694 |
| 1219 | 560 | 10.52632 | 67 | 0 | 694 |
| 1220 | 0 | 2.752294 | 78 | 1 | 905 |
| 1220 | 30 | 1.339286 | 78 | 1 | 905 |
| 1220 | 86 | 2.492212 | 78 | 1 | 905 |
| 1220 | 121 | 2.086957 | 78 | 1 | 905 |

| | | | | | |
|---|---|---|---|---|---|
| 1220 | 212 | 1.572739 | 78 | 1 | 905 |
| 1220 | 289 | 4.444444 | 78 | 1 | 905 |
| 1220 | 373 | 4.660194 | 78 | 1 | 905 |
| 1220 | 471 | 4.660194 | 78 | 1 | 905 |
| 1220 | 590 | 5.365698 | 78 | 1 | 905 |
| 1221 | 0 | 3.092784 | 83 | 0 | 876 |
| 1221 | 56 | 4.395604 | 83 | 0 | 876 |
| 1221 | 147 | 3.296703 | 83 | 0 | 876 |
| 1221 | 245 | 5.769231 | 83 | 0 | 876 |
| 1221 | 301 | 8.807339 | 83 | 0 | 876 |
| 1221 | 420 | 11.21495 | 83 | 0 | 876 |
| 1221 | 483 | 10.34483 | 83 | 0 | 876 |
| 1221 | 574 | 4.848485 | 83 | 0 | 876 |
| 1222 | 0 | 2.702703 | 82 | 0 | 1377 |
| 1222 | 28 | 1.851852 | 82 | 0 | 1377 |
| 1222 | 84 | 1.983471 | 82 | 0 | 1377 |
| 1222 | 161 | 3.387438 | 82 | 0 | 1377 |
| 1222 | 252 | 3.448276 | 82 | 0 | 1377 |
| 1222 | 336 | 5.607477 | 82 | 0 | 1377 |
| 1222 | 420 | 5.357143 | 82 | 0 | 1377 |
| 1222 | 504 | 5.128205 | 82 | 0 | 1377 |
| 1222 | 588 | 5.309735 | 82 | 0 | 1377 |
| 1223 | 0 | 0.681818 | 57 | 0 | 1107 |
| 1223 | 28 | 0.681818 | 57 | 0 | 1107 |
| 1223 | 63 | 1.142857 | 57 | 0 | 1107 |
| 1223 | 161 | 2.352941 | 57 | 0 | 1107 |
| 1223 | 231 | 16.32653 | 57 | 0 | 1107 |
| 1223 | 336 | 12.5 | 57 | 0 | 1107 |
| 1223 | 399 | 2.242991 | 57 | 0 | 1107 |
| 1223 | 504 | 8.080808 | 57 | 0 | 1107 |
| 1223 | 581 | 4.444444 | 57 | 0 | 1107 |
| 1224 | 0 | 1.574803 | 62 | 1 | 992 |
| 1224 | 28 | 2.259887 | 62 | 1 | 992 |
| 1224 | 77 | 7.692308 | 62 | 1 | 992 |
| 1224 | 126 | 1.680672 | 62 | 1 | 992 |
| 1224 | 238 | 4.705882 | 62 | 1 | 992 |
| 1224 | 336 | 4.210526 | 62 | 1 | 992 |
| 1224 | 434 | 7.76699 | 62 | 1 | 992 |
| 1224 | 490 | 5.607477 | 62 | 1 | 992 |

| | | | | | |
|---|---|---|---|---|---|
| 1224 | 581 | 4.40367 | 62 | 1 | 992 |
| 1225 | 0 | 2.362205 | 69 | 1 | 700 |
| 1225 | 28 | 1.382488 | 69 | 1 | 700 |
| 1225 | 77 | 3.047619 | 69 | 1 | 700 |
| 1225 | 154 | 1.626016 | 69 | 1 | 700 |
| 1225 | 252 | 2.492212 | 69 | 1 | 700 |
| 1225 | 336 | 7.371654 | 69 | 1 | 700 |
| 1225 | 420 | 8.716707 | 69 | 1 | 700 |
| 1225 | 504 | 9.375 | 69 | 1 | 700 |
| 1225 | 588 | 2.492212 | 69 | 1 | 700 |
| 1226 | 0 | 2.564103 | 90 | 1 | 500 |
| 1226 | 28 | 0.813008 | 90 | 1 | 500 |
| 1226 | 84 | 1.923077 | 90 | 1 | 500 |
| 1226 | 168 | 5.405405 | 90 | 1 | 500 |
| 1226 | 251 | 2.185792 | 90 | 1 | 500 |
| 1226 | 343 | 6.060606 | 90 | 1 | 500 |
| 1226 | 427 | 0.694444 | 90 | 1 | 500 |
| 1227 | 0 | 2.051282 | 82 | 0 | 869 |
| 1227 | 35 | 1.378122 | 82 | 0 | 869 |
| 1227 | 98 | 1.092896 | 82 | 0 | 869 |
| 1227 | 161 | 1.694915 | 82 | 0 | 869 |
| 1227 | 266 | 1.333333 | 82 | 0 | 869 |
| 1227 | 350 | 9.605489 | 82 | 0 | 869 |
| 1228 | 0 | 4.804805 | 76 | 1 | 415 |
| 1228 | 21 | 2.402402 | 76 | 1 | 415 |
| 1228 | 63 | 4.334365 | 76 | 1 | 415 |
| 1228 | 164 | 9.320388 | 76 | 1 | 415 |
| 1228 | 210 | 8.991169 | 76 | 1 | 415 |
| 1228 | 336 | 20.58824 | 76 | 1 | 415 |
| 1228 | 399 | 18.36735 | 76 | 1 | 415 |
| 1229 | 21 | 2.424242 | 72 | 0 | 883 |
| 1229 | 84 | 2.359882 | 72 | 0 | 883 |
| 1229 | 168 | 2.884615 | 72 | 0 | 883 |
| 1229 | 224 | 5.607477 | 72 | 0 | 883 |
| 1229 | 315 | 5.660377 | 72 | 0 | 883 |
| 1229 | 399 | 5.607477 | 72 | 0 | 883 |
| 1229 | 483 | 5.660377 | 72 | 0 | 883 |
| 1229 | 595 | 10.90909 | 72 | 0 | 883 |
| 1230 | 0 | 1.886792 | 55 | 0 | 610 |

| | | | | | |
|---|---|---|---|---|---|
| 1230 | 28 | 1.834862 | 55 | 0 | 610 |
| 1230 | 84 | 1.801802 | 55 | 0 | 610 |
| 1230 | 189 | 0.718778 | 55 | 0 | 610 |
| 1230 | 231 | 1.630989 | 55 | 0 | 610 |
| 1230 | 322 | 3.2 | 55 | 0 | 610 |
| 1230 | 388 | 0.87146 | 55 | 0 | 610 |
| 1230 | 476 | 1.725998 | 55 | 0 | 610 |
| 1231 | 0 | 3.01941 | 62 | 1 | 281 |
| 1231 | 24 | 2.884615 | 62 | 1 | 281 |
| 1231 | 87 | 4.705882 | 62 | 1 | 281 |
| 1231 | 157 | 2.819265 | 62 | 1 | 281 |
| 1231 | 244 | 13.18681 | 62 | 1 | 281 |
| 1232 | 0 | 5.714286 | 63 | 0 | 685 |
| 1232 | 28 | 2.830189 | 63 | 0 | 685 |
| 1232 | 84 | 2.631579 | 63 | 0 | 685 |
| 1232 | 168 | 6.185567 | 63 | 0 | 685 |
| 1232 | 252 | 15.51724 | 63 | 0 | 685 |
| 1232 | 336 | 21.81818 | 63 | 0 | 685 |
| 1232 | 441 | 6.469003 | 63 | 0 | 685 |
| 1232 | 526 | 2.830189 | 63 | 0 | 685 |
| 1232 | 582 | 6 | 63 | 0 | 685 |
| 1233 | 0 | 5.825243 | 79 | 0 | 1109 |
| 1233 | 28 | 12.37113 | 79 | 0 | 1109 |
| 1233 | 84 | 16 | 79 | 0 | 1109 |
| 1233 | 140 | 14.49838 | 79 | 0 | 1109 |
| 1233 | 252 | 22.42991 | 79 | 0 | 1109 |
| 1233 | 336 | 3.007519 | 79 | 0 | 1109 |
| 1233 | 420 | 9.917355 | 79 | 0 | 1109 |
| 1233 | 525 | 3.278689 | 79 | 0 | 1109 |
| 1233 | 588 | 5.555556 | 79 | 0 | 1109 |
| 1234 | 0 | 5.504587 | 84 | 0 | 967 |
| 1234 | 28 | 5.714286 | 84 | 0 | 967 |
| 1234 | 84 | 5.607477 | 84 | 0 | 967 |
| 1234 | 168 | 5.660377 | 84 | 0 | 967 |
| 1234 | 252 | 12.32575 | 84 | 0 | 967 |
| 1234 | 336 | 12.12121 | 84 | 0 | 967 |
| 1234 | 420 | 7.68 | 84 | 0 | 967 |
| 1234 | 490 | 7.68 | 84 | 0 | 967 |
| 1234 | 595 | 8.135593 | 84 | 0 | 967 |

| | | | | | |
|---|---|---|---|---|---|
| 1235 | 0 | 2.777778 | 85 | 1 | 376 |
| 1235 | 28 | 6.25 | 85 | 1 | 376 |
| 1235 | 84 | 13.04348 | 85 | 1 | 376 |
| 1235 | 175 | 11.91067 | 85 | 1 | 376 |
| 1235 | 273 | 9.896907 | 85 | 1 | 376 |
| 1235 | 343 | 26.08696 | 85 | 1 | 376 |
| 1236 | 0 | 5.490196 | 68 | 0 | 1011 |
| 1236 | 30 | 6.185567 | 68 | 0 | 1011 |
| 1236 | 86 | 8.247423 | 68 | 0 | 1011 |
| 1236 | 261 | 9.056604 | 68 | 0 | 1011 |
| 1236 | 324 | 13.63636 | 68 | 0 | 1011 |
| 1236 | 408 | 15.84158 | 68 | 0 | 1011 |
| 1236 | 534 | 12.42718 | 68 | 0 | 1011 |
| 1236 | 597 | 12.54902 | 68 | 0 | 1011 |
| 1237 | 0 | 2.830189 | 65 | 0 | 596 |
| 1237 | 28 | 2.884615 | 65 | 0 | 596 |
| 1237 | 91 | 3.671329 | 65 | 0 | 596 |
| 1237 | 161 | 5.825243 | 65 | 0 | 596 |
| 1237 | 245 | 5.555556 | 65 | 0 | 596 |
| 1238 | 0 | 5.645161 | 72 | 1 | 137 |
| 1238 | 23 | 3.100775 | 72 | 1 | 137 |
| 1238 | 81 | 8.805031 | 72 | 1 | 137 |
| 1239 | 0 | 3.465347 | 61 | 0 | 649 |
| 1239 | 35 | 3.960396 | 61 | 0 | 649 |
| 1239 | 147 | 4.660194 | 61 | 0 | 649 |
| 1239 | 214 | 5.210918 | 61 | 0 | 649 |
| 1239 | 333 | 3.809524 | 61 | 0 | 649 |
| 1239 | 396 | 3.018868 | 61 | 0 | 649 |
| 1239 | 501 | 5.704584 | 61 | 0 | 649 |
| 1239 | 581 | 3.609023 | 61 | 0 | 649 |
| 1240 | 0 | 6.036651 | 48 | 0 | 995 |
| 1240 | 49 | 1.946472 | 48 | 0 | 995 |
| 1240 | 140 | 2.459016 | 48 | 0 | 995 |
| 1240 | 236 | 4.033613 | 48 | 0 | 995 |
| 1241 | 0 | 6.060606 | 78 | 0 | 785 |
| 1241 | 28 | 2.803738 | 78 | 0 | 785 |
| 1241 | 84 | 6 | 78 | 0 | 785 |
| 1241 | 168 | 5.172414 | 78 | 0 | 785 |
| 1241 | 252 | 5.172414 | 78 | 0 | 785 |

| | | | | | |
|---|---|---|---|---|---|
| 1241 | 336 | 5.217391 | 78 | 0 | 785 |
| 1241 | 421 | 5.882353 | 78 | 0 | 785 |
| 1241 | 504 | 4.017857 | 78 | 0 | 785 |
| 1241 | 588 | 3.947368 | 78 | 0 | 785 |
| 1242 | 0 | 2.298851 | 69 | 0 | 722 |
| 1242 | 28 | 2.307692 | 69 | 0 | 722 |
| 1242 | 84 | 5.042017 | 69 | 0 | 722 |
| 1242 | 168 | 4.878049 | 69 | 0 | 722 |
| 1242 | 248 | 2.333333 | 69 | 0 | 722 |
| 1242 | 336 | 6.62918 | 69 | 0 | 722 |
| 1242 | 420 | 7.563025 | 69 | 0 | 722 |
| 1242 | 504 | 7.377049 | 69 | 0 | 722 |
| 1242 | 588 | 7.758621 | 69 | 0 | 722 |
| 1243 | 0 | 12.5 | 66 | 0 | 715 |
| 1243 | 28 | 5.660377 | 66 | 0 | 715 |
| 1243 | 84 | 3.921569 | 66 | 0 | 715 |
| 1243 | 168 | 2.590394 | 66 | 0 | 715 |
| 1243 | 277 | 2.466055 | 66 | 0 | 715 |
| 1243 | 364 | 2.92581 | 66 | 0 | 715 |
| 1243 | 434 | 2.666667 | 66 | 0 | 715 |
| 1243 | 539 | 2.644628 | 66 | 0 | 715 |
| 1243 | 595 | 2.459016 | 66 | 0 | 715 |
| 1244 | 0 | 2.339181 | 75 | 0 | 1177 |
| 1244 | 56 | 2 | 75 | 0 | 1177 |
| 1244 | 168 | 1.818182 | 75 | 0 | 1177 |
| 1244 | 252 | 0.759734 | 75 | 0 | 1177 |
| 1244 | 315 | 2.830189 | 75 | 0 | 1177 |
| 1244 | 412 | 1.339286 | 75 | 0 | 1177 |
| 1245 | 0 | 3.883495 | 73 | 0 | 547 |
| 1245 | 28 | 3.508772 | 73 | 0 | 547 |
| 1245 | 91 | 2.990654 | 73 | 0 | 547 |
| 1245 | 154 | 9.142857 | 73 | 0 | 547 |
| 1245 | 224 | 8.727273 | 73 | 0 | 547 |
| 1245 | 329 | 8.495575 | 73 | 0 | 547 |
| 1245 | 420 | 13.7931 | 73 | 0 | 547 |
| 1245 | 518 | 7.058824 | 73 | 0 | 547 |
| 1245 | 546 | 5 | 73 | 0 | 547 |
| 1246 | 0 | 12 | 81 | 0 | 680 |
| 1246 | 28 | 14.45161 | 81 | 0 | 680 |

| | | | | | |
|---|---|---|---|---|---|
| 1246 | 77 | 13.18681 | 81 | 0 | 680 |
| 1246 | 161 | 9.677419 | 81 | 0 | 680 |
| 1246 | 249 | 6.962288 | 81 | 0 | 680 |
| 1246 | 347 | 9.108159 | 81 | 0 | 680 |
| 1246 | 434 | 10.90909 | 81 | 0 | 680 |
| 1246 | 497 | 10.61947 | 81 | 0 | 680 |
| 1246 | 588 | 8.495575 | 81 | 0 | 680 |
| 1247 | 0 | 1.739671 | 75 | 0 | 469 |
| 1247 | 29 | 1.73913 | 75 | 0 | 469 |
| 1247 | 85 | 2.727273 | 75 | 0 | 469 |
| 1247 | 169 | 2.752294 | 75 | 0 | 469 |
| 1247 | 267 | 1.941748 | 75 | 0 | 469 |
| 1248 | 0 | 3 | 85 | 0 | 335 |
| 1248 | 24 | 4.223228 | 85 | 0 | 335 |
| 1248 | 91 | 6.122449 | 85 | 0 | 335 |
| 1249 | 0 | 1.279318 | 51 | 1 | 123 |
| 1250 | 0 | 6.666667 | 73 | 0 | 409 |
| 1250 | 28 | 6.185567 | 73 | 0 | 409 |
| 1250 | 81 | 3.571429 | 73 | 0 | 409 |
| 1250 | 154 | 1.965602 | 73 | 0 | 409 |
| 1250 | 263 | 6.122449 | 73 | 0 | 409 |
| 1250 | 316 | 6.122449 | 73 | 0 | 409 |
| 1251 | 0 | 6.481481 | 82 | 0 | 379 |
| 1251 | 29 | 2.666667 | 82 | 0 | 379 |
| 1251 | 64 | 2.359882 | 82 | 0 | 379 |
| 1251 | 134 | 0.714286 | 82 | 0 | 379 |
| 1251 | 225 | 1.818182 | 82 | 0 | 379 |
| 1251 | 316 | 4.255319 | 82 | 0 | 379 |
| 1252 | 0 | 2.707058 | 82 | 0 | 735 |
| 1252 | 28 | 3.448276 | 82 | 0 | 735 |
| 1252 | 91 | 4.371585 | 82 | 0 | 735 |
| 1252 | 168 | 3.225806 | 82 | 0 | 735 |
| 1252 | 224 | 3.333333 | 82 | 0 | 735 |
| 1252 | 308 | 3.508772 | 82 | 0 | 735 |
| 1253 | 0 | 1.162791 | 71 | 0 | 708 |
| 1253 | 28 | 1.271186 | 71 | 0 | 708 |
| 1253 | 84 | 1.376147 | 71 | 0 | 708 |
| 1253 | 168 | 1.428571 | 71 | 0 | 708 |
| 1253 | 252 | 2.654867 | 71 | 0 | 708 |

| | | | | | |
|---|---|---|---|---|---|
| 1253 | 308 | 2.564103 | 71 | 0 | 708 |
| 1253 | 420 | 1.315789 | 71 | 0 | 708 |
| 1253 | 504 | 1.401869 | 71 | 0 | 708 |
| 1253 | 588 | 1.442308 | 71 | 0 | 708 |
| 1254 | 0 | 3.539823 | 78 | 0 | 678 |
| 1254 | 63 | 3.232323 | 78 | 0 | 678 |
| 1254 | 147 | 1.616162 | 78 | 0 | 678 |
| 1254 | 245 | 5.555556 | 78 | 0 | 678 |
| 1254 | 301 | 14.81481 | 78 | 0 | 678 |
| 1254 | 406 | 17.58242 | 78 | 0 | 678 |
| 1254 | 488 | 18.46154 | 78 | 0 | 678 |
| 1255 | 0 | 2.702703 | 66 | 1 | 786 |
| 1255 | 28 | 1.339286 | 66 | 1 | 786 |
| 1255 | 56 | 1.886792 | 66 | 1 | 786 |
| 1255 | 140 | 1.785714 | 66 | 1 | 786 |
| 1255 | 231 | 1.851852 | 66 | 1 | 786 |
| 1255 | 315 | 1.666667 | 66 | 1 | 786 |
| 1255 | 413 | 2.352941 | 66 | 1 | 786 |
| 1255 | 497 | 3.018868 | 66 | 1 | 786 |
| 1255 | 588 | 5.555556 | 66 | 1 | 786 |
| 1256 | 0 | 2.828283 | 76 | 0 | 785 |
| 1256 | 28 | 2.831858 | 76 | 0 | 785 |
| 1256 | 62 | 3.571429 | 76 | 0 | 785 |
| 1256 | 126 | 1.622718 | 76 | 0 | 785 |
| 1256 | 273 | 1.456311 | 76 | 0 | 785 |
| 1256 | 329 | 0.683761 | 76 | 0 | 785 |
| 1256 | 406 | 0.116959 | 76 | 0 | 785 |
| 1256 | 538 | 0.373483 | 76 | 0 | 785 |
| 1256 | 566 | 0.40404 | 76 | 0 | 785 |
| 1257 | 0 | 2.970297 | 81 | 1 | 234 |
| 1257 | 28 | 2.777778 | 81 | 1 | 234 |
| 1257 | 77 | 4.528302 | 81 | 1 | 234 |
| 1257 | 168 | 18.5567 | 81 | 1 | 234 |
| 1258 | 0 | 3.234501 | 44 | 0 | 352 |
| 1258 | 42 | 3.445447 | 44 | 0 | 352 |
| 1258 | 126 | 7.466667 | 44 | 0 | 352 |
| 1258 | 245 | 14 | 44 | 0 | 352 |
| 1258 | 304 | 11.42857 | 44 | 0 | 352 |
| 1259 | 0 | 8.490566 | 85 | 0 | 1021 |

| | | | | | |
|---|---|---|---|---|---|
| 1259 | 28 | 9.143687 | 85 | 0 | 1021 |
| 1259 | 82 | 8.490566 | 85 | 0 | 1021 |
| 1259 | 168 | 12.12121 | 85 | 0 | 1021 |
| 1259 | 252 | 5.940594 | 85 | 0 | 1021 |
| 1259 | 336 | 10.71429 | 85 | 0 | 1021 |
| 1259 | 420 | 10 | 85 | 0 | 1021 |
| 1259 | 504 | 11.11111 | 85 | 0 | 1021 |
| 1259 | 588 | 10.52632 | 85 | 0 | 1021 |
| 1260 | 0 | 1.393728 | 81 | 0 | 1037 |
| 1260 | 21 | 2.285714 | 81 | 0 | 1037 |
| 1260 | 91 | 2.702703 | 81 | 0 | 1037 |
| 1260 | 182 | 14.41441 | 81 | 0 | 1037 |
| 1260 | 272 | 8.034433 | 81 | 0 | 1037 |
| 1260 | 357 | 5.91133 | 81 | 0 | 1037 |
| 1260 | 420 | 10.81081 | 81 | 0 | 1037 |
| 1260 | 518 | 14.28571 | 81 | 0 | 1037 |
| 1260 | 566 | 2.137405 | 81 | 0 | 1037 |
| 1261 | 0 | 11.21495 | 72 | 1 | 331 |
| 1261 | 28 | 10.71429 | 72 | 1 | 331 |
| 1261 | 84 | 8.888889 | 72 | 1 | 331 |
| 1261 | 154 | 8.807339 | 72 | 1 | 331 |
| 1261 | 245 | 11.66667 | 72 | 1 | 331 |
| 1262 | 0 | 3.846154 | 79 | 0 | 568 |
| 1262 | 28 | 3.773585 | 79 | 0 | 568 |
| 1262 | 84 | 3.738318 | 79 | 0 | 568 |
| 1263 | 0 | 5.882353 | 58 | 1 | 73 |
| 1264 | 0 | 5.084746 | 61 | 0 | 1185 |
| 1264 | 108 | 3.902439 | 61 | 0 | 1185 |
| 1264 | 164 | 1.832061 | 61 | 0 | 1185 |
| 1264 | 262 | 2 | 61 | 0 | 1185 |
| 1264 | 325 | 1.709402 | 61 | 0 | 1185 |
| 1264 | 423 | 2.051282 | 61 | 0 | 1185 |
| 1264 | 521 | 1.149425 | 61 | 0 | 1185 |
| 1264 | 577 | 2.439024 | 61 | 0 | 1185 |
| 1265 | 0 | 3.57599 | 71 | 0 | 693 |
| 1265 | 27 | 3.508772 | 71 | 0 | 693 |
| 1265 | 83 | 2.290076 | 71 | 0 | 693 |
| 1265 | 167 | 1.311475 | 71 | 0 | 693 |
| 1265 | 237 | 1.333333 | 71 | 0 | 693 |

| | | | | | |
|---|---|---|---|---|---|
| 1265 | 342 | 1.37931 | 71 | 0 | 693 |
| 1265 | 412 | 1.311475 | 71 | 0 | 693 |
| 1265 | 517 | 1.415929 | 71 | 0 | 693 |
| 1265 | 587 | 2.285714 | 71 | 0 | 693 |
| 1266 | 0 | 3.252033 | 74 | 0 | 673 |
| 1266 | 28 | 3.508772 | 74 | 0 | 673 |
| 1266 | 84 | 4.347826 | 74 | 0 | 673 |
| 1266 | 168 | 4.310345 | 74 | 0 | 673 |
| 1266 | 252 | 5.042017 | 74 | 0 | 673 |
| 1266 | 336 | 5.042017 | 74 | 0 | 673 |
| 1266 | 420 | 3.361345 | 74 | 0 | 673 |
| 1266 | 504 | 3.389831 | 74 | 0 | 673 |
| 1266 | 560 | 3.278689 | 74 | 0 | 673 |
| 1267 | 0 | 5.882353 | 84 | 0 | 693 |
| 1267 | 28 | 6.521739 | 84 | 0 | 693 |
| 1267 | 84 | 11.11111 | 84 | 0 | 693 |
| 1267 | 182 | 9.142857 | 84 | 0 | 693 |
| 1267 | 245 | 14.69388 | 84 | 0 | 693 |
| 1267 | 336 | 14.69388 | 84 | 0 | 693 |
| 1267 | 427 | 19.2 | 84 | 0 | 693 |
| 1267 | 518 | 19.78022 | 84 | 0 | 693 |
| 1267 | 574 | 20.22472 | 84 | 0 | 693 |
| 1268 | 0 | 2.446483 | 72 | 0 | 803 |
| 1268 | 28 | 2.539683 | 72 | 0 | 803 |
| 1268 | 102 | 7.204117 | 72 | 0 | 803 |
| 1268 | 165 | 12 | 72 | 0 | 803 |
| 1268 | 249 | 6.896552 | 72 | 0 | 803 |
| 1268 | 326 | 2.608696 | 72 | 0 | 803 |
| 1268 | 445 | 2.203857 | 72 | 0 | 803 |
| 1268 | 500 | 7.476636 | 72 | 0 | 803 |
| 1268 | 612 | 7.407407 | 72 | 0 | 803 |
| 1269 | 0 | 13.04348 | 62 | 0 | 295 |
| 1269 | 28 | 3.883495 | 62 | 0 | 295 |
| 1269 | 84 | 16.16162 | 62 | 0 | 295 |
| 1269 | 168 | 16.84211 | 62 | 0 | 295 |
| 1269 | 254 | 19.78022 | 62 | 0 | 295 |
| 1270 | 0 | 4.882302 | 63 | 1 | 194 |
| 1270 | 31 | 4.528302 | 63 | 1 | 194 |
| 1270 | 66 | 5.454545 | 63 | 1 | 194 |

| | | | | | |
|---|---|---|---|---|---|
| 1270 | 178 | 3.536842 | 63 | 1 | 194 |
| 1271 | 0 | 1.246106 | 78 | 0 | 596 |
| 1271 | 81 | 1.101278 | 78 | 0 | 596 |
| 1271 | 126 | 0.909091 | 78 | 0 | 596 |
| 1271 | 259 | 2.568807 | 78 | 0 | 596 |
| 1271 | 343 | 2.469136 | 78 | 0 | 596 |
| 1271 | 427 | 1.223242 | 78 | 0 | 596 |
| 1271 | 511 | 1.294498 | 78 | 0 | 596 |
| 1271 | 595 | 1.269841 | 78 | 0 | 596 |
| 1272 | 0 | 0.900901 | 74 | 0 | 694 |
| 1272 | 28 | 0.862069 | 74 | 0 | 694 |
| 1272 | 84 | 2.803738 | 74 | 0 | 694 |
| 1272 | 168 | 1.058201 | 74 | 0 | 694 |
| 1272 | 259 | 2.727273 | 74 | 0 | 694 |
| 1272 | 322 | 2.123894 | 74 | 0 | 694 |
| 1272 | 413 | 1.315789 | 74 | 0 | 694 |
| 1272 | 497 | 2.654867 | 74 | 0 | 694 |
| 1273 | 0 | 4.918033 | 68 | 0 | 697 |
| 1273 | 28 | 5 | 68 | 0 | 697 |
| 1273 | 84 | 4.666667 | 68 | 0 | 697 |
| 1273 | 175 | 7.894737 | 68 | 0 | 697 |
| 1273 | 258 | 12.08198 | 68 | 0 | 697 |
| 1274 | 0 | 13.43284 | 72 | 0 | 1058 |
| 1274 | 28 | 2.466598 | 72 | 0 | 1058 |
| 1274 | 175 | 2.539683 | 72 | 0 | 1058 |
| 1275 | 28 | 8.205128 | 52 | 0 | 734 |
| 1275 | 112 | 3.32871 | 52 | 0 | 734 |
| 1275 | 175 | 2.777778 | 52 | 0 | 734 |
| 1275 | 231 | 6.926407 | 52 | 0 | 734 |
| 1275 | 322 | 6.65742 | 52 | 0 | 734 |
| 1275 | 413 | 9.320388 | 52 | 0 | 734 |
| 1275 | 476 | 7.692308 | 52 | 0 | 734 |
| 1275 | 581 | 8.247423 | 52 | 0 | 734 |
| 1276 | 0 | 1.304348 | 64 | 0 | 659 |
| 1276 | 28 | 1.363636 | 64 | 0 | 659 |
| 1276 | 91 | 4.705882 | 64 | 0 | 659 |
| 1276 | 161 | 1.315789 | 64 | 0 | 659 |
| 1276 | 252 | 4.040404 | 64 | 0 | 659 |
| 1276 | 343 | 2.857143 | 64 | 0 | 659 |

| | | | | | |
|---|---|---|---|---|---|
| 1276 | 399 | 3.498542 | 64 | 0 | 659 |
| 1276 | 497 | 8.421053 | 64 | 0 | 659 |
| 1276 | 581 | 14.54545 | 64 | 0 | 659 |
| 1277 | 0 | 2.5 | 69 | 0 | 665 |
| 1277 | 35 | 3.703704 | 69 | 0 | 665 |
| 1277 | 91 | 5.517241 | 69 | 0 | 665 |
| 1277 | 182 | 12.12121 | 69 | 0 | 665 |
| 1277 | 245 | 12.12121 | 69 | 0 | 665 |
| 1277 | 329 | 7.476636 | 69 | 0 | 665 |
| 1277 | 413 | 13.18681 | 69 | 0 | 665 |
| 1277 | 504 | 1.27186 | 69 | 0 | 665 |
| 1277 | 595 | 8.791209 | 69 | 0 | 665 |
| 1278 | 0 | 1.142857 | 57 | 1 | 525 |
| 1278 | 35 | 1.442308 | 57 | 1 | 525 |
| 1278 | 119 | 1.52381 | 57 | 1 | 525 |
| 1278 | 147 | 4.848485 | 57 | 1 | 525 |
| 1278 | 231 | 6.334842 | 57 | 1 | 525 |
| 1278 | 343 | 5.454545 | 57 | 1 | 525 |
| 1278 | 406 | 9.795918 | 57 | 1 | 525 |
| 1278 | 490 | 10.38961 | 57 | 1 | 525 |
| 1279 | 0 | 2.105263 | 69 | 0 | 715 |
| 1279 | 28 | 2.44898 | 69 | 0 | 715 |
| 1279 | 91 | 4.123711 | 69 | 0 | 715 |
| 1279 | 182 | 8.807339 | 69 | 0 | 715 |
| 1279 | 280 | 10.52632 | 69 | 0 | 715 |
| 1279 | 406 | 5.614035 | 69 | 0 | 715 |
| 1279 | 476 | 5.981308 | 69 | 0 | 715 |
| 1279 | 581 | 6.4 | 69 | 0 | 715 |
| 1280 | 0 | 6.451613 | 76 | 0 | 355 |
| 1280 | 98 | 0.712347 | 76 | 0 | 355 |
| 1280 | 154 | 7.058824 | 76 | 0 | 355 |
| 1280 | 266 | 6.060606 | 76 | 0 | 355 |
| 1281 | 0 | 5.607477 | 37 | 1 | 288 |
| 1281 | 28 | 4.40367 | 37 | 1 | 288 |
| 1281 | 91 | 5.940594 | 37 | 1 | 288 |
| 1281 | 160 | 17.30769 | 37 | 1 | 288 |
| 1281 | 245 | 15.78947 | 37 | 1 | 288 |
| 1282 | 0 | 5.773196 | 76 | 0 | 94 |
| 1282 | 70 | 10.32258 | 76 | 0 | 94 |

| | | | | | |
|---|---|---|---|---|---|
| 1283 | 0 | 10.90909 | 76 | 0 | 752 |
| 1283 | 28 | 9.230769 | 76 | 0 | 752 |
| 1283 | 63 | 7.476636 | 76 | 0 | 752 |
| 1283 | 161 | 10.43478 | 76 | 0 | 752 |
| 1283 | 241 | 4.724409 | 76 | 0 | 752 |
| 1283 | 308 | 6.837607 | 76 | 0 | 752 |
| 1283 | 378 | 4.324324 | 76 | 0 | 752 |
| 1283 | 463 | 4.40367 | 76 | 0 | 752 |
| 1283 | 581 | 9.52381 | 76 | 0 | 752 |
| 1284 | 0 | 2.542373 | 78 | 0 | 729 |
| 1284 | 77 | 1.754386 | 78 | 0 | 729 |
| 1284 | 168 | 0.884956 | 78 | 0 | 729 |
| 1284 | 224 | 1.664355 | 78 | 0 | 729 |
| 1284 | 322 | 1.485149 | 78 | 0 | 729 |
| 1284 | 378 | 0.655738 | 78 | 0 | 729 |
| 1284 | 609 | 1.111111 | 78 | 0 | 729 |
| 1285 | 0 | 4.615385 | 87 | 1 | 402 |
| 1285 | 35 | 4.851752 | 87 | 1 | 402 |
| 1285 | 140 | 12.37113 | 87 | 1 | 402 |
| 1285 | 231 | 19.56522 | 87 | 1 | 402 |
| 1285 | 329 | 9.375 | 87 | 1 | 402 |
| 1285 | 385 | 10.71429 | 87 | 1 | 402 |
| 1286 | 0 | 10.98039 | 80 | 0 | 1059 |
| 1286 | 28 | 10.62955 | 80 | 0 | 1059 |
| 1286 | 85 | 7.559055 | 80 | 0 | 1059 |
| 1286 | 155 | 7.68 | 80 | 0 | 1059 |
| 1287 | 28 | 5.042017 | 68 | 1 | 317 |
| 1287 | 84 | 8.411215 | 68 | 1 | 317 |
| 1287 | 132 | 11.21495 | 68 | 1 | 317 |
| 1287 | 245 | 16.21622 | 68 | 1 | 317 |
| 1288 | 0 | 2.990654 | 75 | 0 | 1444 |
| 1288 | 65 | 2.123894 | 75 | 0 | 1444 |
| 1288 | 149 | 1.652893 | 75 | 0 | 1444 |
| 1288 | 226 | 7.758621 | 75 | 0 | 1444 |
| 1288 | 317 | 13.71429 | 75 | 0 | 1444 |
| 1288 | 380 | 10.90909 | 75 | 0 | 1444 |
| 1288 | 499 | 7.843137 | 75 | 0 | 1444 |
| 1288 | 560 | 14.6789 | 75 | 0 | 1444 |
| 1289 | 0 | 10.61947 | 53 | 1 | 391 |

| | | | | | |
|---|---|---|---|---|---|
| 1289 | 28 | 17.82178 | 53 | 1 | 391 |
| 1289 | 84 | 18.18182 | 53 | 1 | 391 |
| 1289 | 168 | 17.64706 | 53 | 1 | 391 |
| 1289 | 252 | 16.36364 | 53 | 1 | 391 |
| 1289 | 336 | 31.57895 | 53 | 1 | 391 |
| 1290 | 0 | 4.761905 | 64 | 0 | 1060 |
| 1290 | 28 | 13.48315 | 64 | 0 | 1060 |
| 1290 | 84 | 11.65049 | 64 | 0 | 1060 |
| 1290 | 147 | 9.896907 | 64 | 0 | 1060 |
| 1290 | 252 | 8.648649 | 64 | 0 | 1060 |
| 1290 | 329 | 8.347826 | 64 | 0 | 1060 |
| 1290 | 406 | 8.495575 | 64 | 0 | 1060 |
| 1290 | 483 | 9.411765 | 64 | 0 | 1060 |
| 1290 | 595 | 7.692308 | 64 | 0 | 1060 |
| 1291 | 0 | 0.991736 | 67 | 0 | 673 |
| 1291 | 28 | 0.956938 | 67 | 0 | 673 |
| 1291 | 98 | 1.239669 | 67 | 0 | 673 |
| 1291 | 182 | 5.607477 | 67 | 0 | 673 |
| 1291 | 280 | 4.752475 | 67 | 0 | 673 |
| 1291 | 364 | 2.359882 | 67 | 0 | 673 |
| 1291 | 427 | 5.769231 | 67 | 0 | 673 |
| 1291 | 518 | 4.363636 | 67 | 0 | 673 |
| 1291 | 609 | 5.309735 | 67 | 0 | 673 |
| 1292 | 0 | 1.219512 | 75 | 0 | 1119 |
| 1292 | 28 | 1.025641 | 75 | 0 | 1119 |
| 1292 | 98 | 0.847458 | 75 | 0 | 1119 |
| 1292 | 182 | 1.304348 | 75 | 0 | 1119 |
| 1292 | 273 | 1.25 | 75 | 0 | 1119 |
| 1292 | 329 | 0.983607 | 75 | 0 | 1119 |
| 1292 | 424 | 1.229508 | 75 | 0 | 1119 |
| 1292 | 508 | 0.96 | 75 | 0 | 1119 |
| 1293 | 0 | 3.865624 | 84 | 0 | 685 |
| 1293 | 41 | 3.738318 | 84 | 0 | 685 |
| 1293 | 83 | 2.446483 | 84 | 0 | 685 |
| 1293 | 209 | 2.298851 | 84 | 0 | 685 |
| 1293 | 272 | 0.808081 | 84 | 0 | 685 |
| 1293 | 335 | 0.740741 | 84 | 0 | 685 |
| 1293 | 461 | 0.934579 | 84 | 0 | 685 |
| 1293 | 531 | 0.815494 | 84 | 0 | 685 |

| | | | | | |
|---|---|---|---|---|---|
| 1293 | 622 | 3.603604 | 84 | 0 | 685 |
| 1294 | 0 | 2.727273 | 72 | 0 | 665 |
| 1294 | 28 | 2.559025 | 72 | 0 | 665 |
| 1294 | 93 | 1.797176 | 72 | 0 | 665 |
| 1294 | 175 | 6.122449 | 72 | 0 | 665 |
| 1294 | 273 | 4.247788 | 72 | 0 | 665 |
| 1294 | 381 | 1.690821 | 72 | 0 | 665 |
| 1294 | 437 | 12.12121 | 72 | 0 | 665 |
| 1294 | 528 | 2.666667 | 72 | 0 | 665 |
| 1294 | 591 | 5.442177 | 72 | 0 | 665 |
| 1295 | 0 | 6.666667 | 75 | 0 | 760 |
| 1295 | 31 | 10.46512 | 75 | 0 | 760 |
| 1295 | 87 | 14.63415 | 75 | 0 | 760 |
| 1295 | 206 | 13.04348 | 75 | 0 | 760 |
| 1295 | 241 | 11.32075 | 75 | 0 | 760 |
| 1295 | 325 | 11.65049 | 75 | 0 | 760 |
| 1295 | 409 | 8.648649 | 75 | 0 | 760 |
| 1295 | 500 | 8.571429 | 75 | 0 | 760 |
| 1295 | 591 | 9.917355 | 75 | 0 | 760 |
| 1296 | 0 | 25.35211 | 80 | 0 | 101 |
| 1296 | 28 | 9 | 80 | 0 | 101 |
| 1296 | 84 | 9.677419 | 80 | 0 | 101 |
| 1297 | 0 | 5.660377 | 71 | 0 | 473 |
| 1297 | 28 | 6.363636 | 71 | 0 | 473 |
| 1297 | 77 | 11.16279 | 71 | 0 | 473 |
| 1297 | 178 | 5.25 | 71 | 0 | 473 |
| 1297 | 234 | 12 | 71 | 0 | 473 |
| 1297 | 332 | 10.66667 | 71 | 0 | 473 |
| 1297 | 423 | 11.65453 | 71 | 0 | 473 |
| 1298 | 0 | 10.90909 | 68 | 0 | 599 |
| 1298 | 30 | 10.19417 | 68 | 0 | 599 |
| 1298 | 56 | 10.71429 | 68 | 0 | 599 |
| 1298 | 199 | 11.21495 | 68 | 0 | 599 |
| 1298 | 255 | 10.43478 | 68 | 0 | 599 |
| 1298 | 339 | 8.318479 | 68 | 0 | 599 |
| 1298 | 395 | 2.83353 | 68 | 0 | 599 |
| 1298 | 486 | 10.61947 | 68 | 0 | 599 |
| 1298 | 570 | 10.34483 | 68 | 0 | 599 |
| 1299 | 0 | 5.172414 | 77 | 1 | 920 |

| | | | | | |
|---|---|---|---|---|---|
| 1299 | 21 | 3.225806 | 77 | 1 | 920 |
| 1299 | 84 | 5.128205 | 77 | 1 | 920 |
| 1299 | 140 | 4.869565 | 77 | 1 | 920 |
| 1299 | 259 | 2.803738 | 77 | 1 | 920 |
| 1299 | 357 | 5.607477 | 77 | 1 | 920 |
| 1300 | 0 | 2.830189 | 50 | 0 | 160 |
| 1300 | 28 | 3.703704 | 50 | 0 | 160 |
| 1300 | 84 | 5.504587 | 50 | 0 | 160 |
| 1300 | 159 | 8.333333 | 50 | 0 | 160 |
| 1301 | 0 | 6.837607 | 70 | 0 | 196 |
| 1301 | 27 | 10.08403 | 70 | 0 | 196 |
| 1301 | 83 | 10.34483 | 70 | 0 | 196 |
| 1301 | 167 | 11.86441 | 70 | 0 | 196 |
| 1302 | 0 | 7.619048 | 80 | 1 | 27 |
| 1303 | 0 | 2.678571 | 76 | 1 | 620 |
| 1303 | 28 | 5.454545 | 76 | 1 | 620 |
| 1303 | 84 | 5.454545 | 76 | 1 | 620 |
| 1303 | 140 | 5.454545 | 76 | 1 | 620 |
| 1303 | 287 | 6.060606 | 76 | 1 | 620 |
| 1303 | 371 | 12.5 | 76 | 1 | 620 |
| 1303 | 427 | 12.37113 | 76 | 1 | 620 |
| 1303 | 511 | 12.90323 | 76 | 1 | 620 |
| 1303 | 567 | 12.90323 | 76 | 1 | 620 |
| 1304 | 0 | 5.555556 | 76 | 0 | 860 |
| 1304 | 28 | 6.792453 | 76 | 0 | 860 |
| 1304 | 91 | 16.49485 | 76 | 0 | 860 |
| 1304 | 175 | 10.90909 | 76 | 0 | 860 |
| 1304 | 259 | 11.11111 | 76 | 0 | 860 |
| 1304 | 320 | 11.00917 | 76 | 0 | 860 |
| 1304 | 432 | 10.16949 | 76 | 0 | 860 |
| 1304 | 516 | 11.42857 | 76 | 0 | 860 |
| 1304 | 600 | 10.61947 | 76 | 0 | 860 |
| 1305 | 0 | 10 | 61 | 0 | 911 |
| 1305 | 35 | 16.49485 | 61 | 0 | 911 |
| 1305 | 84 | 10.71429 | 61 | 0 | 911 |
| 1305 | 168 | 1.441441 | 61 | 0 | 911 |
| 1305 | 238 | 3.030303 | 61 | 0 | 911 |
| 1305 | 343 | 5.882353 | 61 | 0 | 911 |
| 1305 | 406 | 4.40367 | 61 | 0 | 911 |

| | | | | | |
|---|---|---|---|---|---|
| 1305 | 504 | 6.060606 | 61 | 0 | 911 |
| 1305 | 567 | 2.330097 | 61 | 0 | 911 |
| 1306 | 0 | 4.984424 | 50 | 1 | 74 |
| 1306 | 28 | 6 | 50 | 1 | 74 |
| 1307 | 0 | 2.752294 | 77 | 0 | 722 |
| 1307 | 28 | 2.521008 | 77 | 0 | 722 |
| 1307 | 112 | 1.6 | 77 | 0 | 722 |
| 1307 | 168 | 0.833333 | 77 | 0 | 722 |
| 1307 | 252 | 0.813008 | 77 | 0 | 722 |
| 1307 | 308 | 0.550964 | 77 | 0 | 722 |
| 1307 | 420 | 0.666667 | 77 | 0 | 722 |
| 1307 | 490 | 0.666667 | 77 | 0 | 722 |
| 1308 | 0 | 2.586207 | 44 | 1 | 742 |
| 1308 | 28 | 2.608696 | 44 | 1 | 742 |
| 1308 | 84 | 2.702703 | 44 | 1 | 742 |
| 1308 | 168 | 2.777778 | 44 | 1 | 742 |
| 1308 | 252 | 2.242991 | 44 | 1 | 742 |
| 1308 | 343 | 2.830189 | 44 | 1 | 742 |
| 1308 | 427 | 12.12121 | 44 | 1 | 742 |
| 1308 | 511 | 11.21495 | 44 | 1 | 742 |
| 1308 | 588 | 15.68627 | 44 | 1 | 742 |
| 1309 | 0 | 9.142857 | 66 | 0 | 678 |
| 1309 | 49 | 8.971963 | 66 | 0 | 678 |
| 1309 | 196 | 1.096105 | 66 | 0 | 678 |
| 1309 | 323 | 1.165857 | 66 | 0 | 678 |
| 1309 | 408 | 1.424936 | 66 | 0 | 678 |
| 1309 | 519 | 11.24874 | 66 | 0 | 678 |
| 1310 | 0 | 4.761905 | 81 | 0 | 561 |
| 1310 | 28 | 2.34375 | 81 | 0 | 561 |
| 1310 | 84 | 1.709402 | 81 | 0 | 561 |
| 1310 | 175 | 1.37931 | 81 | 0 | 561 |
| 1310 | 238 | 1.169591 | 81 | 0 | 561 |
| 1310 | 322 | 1.769912 | 81 | 0 | 561 |
| 1310 | 434 | 1.149425 | 81 | 0 | 561 |
| 1310 | 476 | 1.179941 | 81 | 0 | 561 |
| 1310 | 560 | 1.129944 | 81 | 0 | 561 |
| 1311 | 0 | 2.937063 | 80 | 0 | 727 |
| 1311 | 26 | 2.564103 | 80 | 0 | 727 |
| 1311 | 82 | 2.678571 | 80 | 0 | 727 |

| | | | | | |
|---|---|---|---|---|---|
| 1311 | 175 | 2.859088 | 80 | 0 | 727 |
| 1311 | 257 | 2.666667 | 80 | 0 | 727 |
| 1311 | 343 | 5.825243 | 80 | 0 | 727 |
| 1311 | 427 | 4.285714 | 80 | 0 | 727 |
| 1311 | 497 | 5.314774 | 80 | 0 | 727 |
| 1311 | 596 | 3.846154 | 80 | 0 | 727 |
| 1312 | 0 | 1.25 | 45 | 1 | 505 |
| 1312 | 28 | 3.448276 | 45 | 1 | 505 |
| 1312 | 84 | 1.545894 | 45 | 1 | 505 |
| 1312 | 175 | 3.448276 | 45 | 1 | 505 |
| 1312 | 257 | 3.603604 | 45 | 1 | 505 |
| 1312 | 343 | 3.809524 | 45 | 1 | 505 |
| 1312 | 392 | 4.795889 | 45 | 1 | 505 |
| 1312 | 485 | 5.517241 | 45 | 1 | 505 |
| 1313 | 0 | 3.030303 | 75 | 0 | 636 |
| 1313 | 28 | 2.941176 | 75 | 0 | 636 |
| 1313 | 88 | 4.123711 | 75 | 0 | 636 |
| 1313 | 179 | 3.478261 | 75 | 0 | 636 |
| 1313 | 285 | 6.436782 | 75 | 0 | 636 |
| 1313 | 340 | 4.528302 | 75 | 0 | 636 |
| 1313 | 403 | 4.705882 | 75 | 0 | 636 |
| 1313 | 501 | 4.8 | 75 | 0 | 636 |
| 1313 | 571 | 5.16129 | 75 | 0 | 636 |
| 1314 | 0 | 0.847458 | 74 | 0 | 1038 |
| 1314 | 34 | 2.4 | 74 | 0 | 1038 |
| 1314 | 56 | 2.307692 | 74 | 0 | 1038 |
| 1314 | 182 | 1.617251 | 74 | 0 | 1038 |
| 1314 | 230 | 1.632653 | 74 | 0 | 1038 |
| 1314 | 341 | 2.181818 | 74 | 0 | 1038 |
| 1314 | 517 | 4.660194 | 74 | 0 | 1038 |
| 1315 | 0 | 13.63636 | 69 | 1 | 469 |
| 1315 | 28 | 12.76596 | 69 | 1 | 469 |
| 1315 | 84 | 13.48315 | 69 | 1 | 469 |
| 1315 | 140 | 31.57895 | 69 | 1 | 469 |
| 1315 | 231 | 12.37113 | 69 | 1 | 469 |
| 1315 | 315 | 12.80488 | 69 | 1 | 469 |
| 1315 | 403 | 15.38462 | 69 | 1 | 469 |
| 1316 | 0 | 0.607287 | 74 | 0 | 631 |
| 1316 | 63 | 0.765306 | 74 | 0 | 631 |

| | | | | | |
|---|---|---|---|---|---|
| 1316 | 175 | 1.388889 | 74 | 0 | 631 |
| 1316 | 238 | 2.474227 | 74 | 0 | 631 |
| 1316 | 308 | 1.904762 | 74 | 0 | 631 |
| 1316 | 448 | 5.769231 | 74 | 0 | 631 |
| 1316 | 504 | 2.539683 | 74 | 0 | 631 |
| 1316 | 567 | 5.734767 | 74 | 0 | 631 |
| 1317 | 0 | 3.773585 | 70 | 0 | 1404 |
| 1317 | 91 | 3.32871 | 70 | 0 | 1404 |
| 1317 | 140 | 3.296703 | 70 | 0 | 1404 |
| 1317 | 238 | 3.32871 | 70 | 0 | 1404 |
| 1317 | 336 | 3.174603 | 70 | 0 | 1404 |
| 1317 | 385 | 2.752294 | 70 | 0 | 1404 |
| 1317 | 504 | 2.640264 | 70 | 0 | 1404 |
| 1317 | 567 | 3.32871 | 70 | 0 | 1404 |
| 1318 | 0 | 7.427056 | 79 | 1 | 33 |
| 1319 | 0 | 3.298969 | 86 | 0 | 274 |
| 1319 | 35 | 1.960784 | 86 | 0 | 274 |
| 1319 | 91 | 1.980198 | 86 | 0 | 274 |
| 1319 | 147 | 1.980198 | 86 | 0 | 274 |
| 1319 | 273 | 11.85185 | 86 | 0 | 274 |
| 1320 | 0 | 5.24836 | 64 | 0 | 775 |
| 1320 | 27 | 4.444444 | 64 | 0 | 775 |
| 1320 | 54 | 4.848485 | 64 | 0 | 775 |
| 1320 | 146 | 7.692308 | 64 | 0 | 775 |
| 1320 | 214 | 23.07692 | 64 | 0 | 775 |
| 1321 | 0 | 2.521008 | 67 | 0 | 1122 |
| 1321 | 70 | 1.438849 | 67 | 0 | 1122 |
| 1322 | 0 | 5.454545 | 61 | 1 | 143 |
| 1322 | 28 | 4.411765 | 61 | 1 | 143 |
| 1322 | 62 | 5.363985 | 61 | 1 | 143 |
| 1323 | 0 | 9.917355 | 66 | 1 | 265 |
| 1323 | 28 | 9.836066 | 66 | 1 | 265 |
| 1323 | 84 | 7.933884 | 66 | 1 | 265 |
| 1323 | 175 | 10.71429 | 66 | 1 | 265 |
| 1323 | 238 | 9.411765 | 66 | 1 | 265 |
| 1324 | 0 | 0.641026 | 46 | 0 | 764 |
| 1324 | 28 | 0.657895 | 46 | 0 | 764 |
| 1324 | 63 | 0.590261 | 46 | 0 | 764 |
| 1324 | 161 | 0.549199 | 46 | 0 | 764 |

| | | | | | |
|---|---|---|---|---|---|
| 1324 | 252 | 1.271186 | 46 | 0 | 764 |
| 1324 | 336 | 2.631579 | 46 | 0 | 764 |
| 1324 | 399 | 0.983607 | 46 | 0 | 764 |
| 1324 | 490 | 2.068966 | 46 | 0 | 764 |
| 1324 | 588 | 1.246106 | 46 | 0 | 764 |
| 1325 | 0 | 1.678322 | 74 | 0 | 1118 |
| 1325 | 77 | 3.448276 | 74 | 0 | 1118 |
| 1325 | 165 | 1.983471 | 74 | 0 | 1118 |
| 1325 | 235 | 1.151079 | 74 | 0 | 1118 |
| 1325 | 333 | 5.714286 | 74 | 0 | 1118 |
| 1325 | 431 | 1.95122 | 74 | 0 | 1118 |
| 1325 | 494 | 0.895522 | 74 | 0 | 1118 |
| 1325 | 564 | 4.485981 | 74 | 0 | 1118 |
| 1326 | 0 | 2.439024 | 65 | 1 | 841 |
| 1326 | 28 | 1.171875 | 65 | 1 | 841 |
| 1326 | 84 | 1.201201 | 65 | 1 | 841 |
| 1326 | 168 | 1.941748 | 65 | 1 | 841 |
| 1326 | 252 | 4.166667 | 65 | 1 | 841 |
| 1326 | 336 | 6.417112 | 65 | 1 | 841 |
| 1326 | 413 | 5.538462 | 65 | 1 | 841 |
| 1326 | 504 | 12.63158 | 65 | 1 | 841 |
| 1326 | 588 | 12.76596 | 65 | 1 | 841 |
| 1327 | 0 | 5.217391 | 77 | 1 | 52 |
| 1327 | 35 | 32.87671 | 77 | 1 | 52 |
| 1328 | 0 | 2.242991 | 66 | 1 | 309 |
| 1328 | 28 | 5.504587 | 66 | 1 | 309 |
| 1328 | 56 | 5.357143 | 66 | 1 | 309 |
| 1328 | 140 | 5.309735 | 66 | 1 | 309 |
| 1328 | 224 | 6.408545 | 66 | 1 | 309 |
| 1328 | 308 | 10.71429 | 66 | 1 | 309 |
| 1329 | 0 | 6.666667 | 68 | 0 | 897 |
| 1329 | 77 | 4.247788 | 68 | 0 | 897 |
| 1329 | 168 | 10.25641 | 68 | 0 | 897 |
| 1329 | 230 | 5.314774 | 68 | 0 | 897 |
| 1329 | 315 | 5.504587 | 68 | 0 | 897 |
| 1329 | 420 | 4.137931 | 68 | 0 | 897 |
| 1329 | 518 | 8.421053 | 68 | 0 | 897 |
| 1329 | 552 | 7.969639 | 68 | 0 | 897 |
| 1330 | 0 | 2.242991 | 81 | 0 | 596 |

| | | | | | |
|---|---|---|---|---|---|
| 1330 | 35 | 2.086957 | 81 | 0 | 596 |
| 1330 | 105 | 2.105263 | 81 | 0 | 596 |
| 1330 | 168 | 2.586207 | 81 | 0 | 596 |
| 1331 | 0 | 2.162162 | 63 | 0 | 270 |
| 1331 | 35 | 2.086957 | 63 | 0 | 270 |
| 1331 | 70 | 5.769231 | 63 | 0 | 270 |
| 1331 | 126 | 4.137931 | 63 | 0 | 270 |
| 1331 | 217 | 5.128205 | 63 | 0 | 270 |
| 1332 | 0 | 12.07764 | 56 | 1 | 875 |
| 1332 | 34 | 4.827586 | 56 | 1 | 875 |
| 1332 | 91 | 4.958678 | 56 | 1 | 875 |
| 1332 | 175 | 10.61947 | 56 | 1 | 875 |
| 1332 | 266 | 2.133333 | 56 | 1 | 875 |
| 1332 | 336 | 11.65049 | 56 | 1 | 875 |
| 1332 | 424 | 10.81081 | 56 | 1 | 875 |
| 1332 | 504 | 11.32075 | 56 | 1 | 875 |
| 1332 | 574 | 8 | 56 | 1 | 875 |
| 1333 | 0 | 3.636364 | 80 | 0 | 946 |
| 1333 | 98 | 1.190476 | 80 | 0 | 946 |
| 1333 | 154 | 2.205882 | 80 | 0 | 946 |
| 1333 | 266 | 3.296703 | 80 | 0 | 946 |
| 1334 | 0 | 16.82243 | 65 | 0 | 1108 |
| 1334 | 42 | 12.10084 | 65 | 0 | 1108 |
| 1334 | 112 | 8.067227 | 65 | 0 | 1108 |
| 1334 | 203 | 2.086957 | 65 | 0 | 1108 |
| 1335 | 0 | 8.247423 | 76 | 1 | 118 |
| 1335 | 28 | 6 | 76 | 1 | 118 |
| 1335 | 84 | 3.571429 | 76 | 1 | 118 |
| 1336 | 0 | 6.593407 | 55 | 1 | 969 |
| 1336 | 28 | 6.25 | 55 | 1 | 969 |
| 1336 | 84 | 7.692308 | 55 | 1 | 969 |
| 1336 | 140 | 11.88119 | 55 | 1 | 969 |
| 1336 | 238 | 6.956522 | 55 | 1 | 969 |
| 1336 | 350 | 12.97297 | 55 | 1 | 969 |
| 1336 | 413 | 10.43478 | 55 | 1 | 969 |
| 1336 | 490 | 12.63158 | 55 | 1 | 969 |
| 1336 | 608 | 13.07732 | 55 | 1 | 969 |
| 1337 | 0 | 2.162162 | 74 | 1 | 103 |
| 1337 | 35 | 4.296675 | 74 | 1 | 103 |

| | | | | | |
|---|---|---|---|---|---|
| 1337 | 45 | 4.661487 | 74 | 1 | 103 |
| 1338 | 0 | 2.5 | 71 | 0 | 1072 |
| 1338 | 28 | 1.282051 | 71 | 0 | 1072 |
| 1338 | 84 | 2.803738 | 71 | 0 | 1072 |
| 1338 | 173 | 5.454545 | 71 | 0 | 1072 |
| 1338 | 258 | 2.884615 | 71 | 0 | 1072 |
| 1338 | 334 | 2.912621 | 71 | 0 | 1072 |
| 1338 | 418 | 5.660377 | 71 | 0 | 1072 |
| 1338 | 499 | 2.459016 | 71 | 0 | 1072 |
| 1338 | 615 | 2.330097 | 71 | 0 | 1072 |
| 1339 | 0 | 4.363636 | 51 | 1 | 583 |
| 1339 | 36 | 1.333333 | 51 | 1 | 583 |
| 1339 | 69 | 2.318841 | 51 | 1 | 583 |
| 1339 | 155 | 2.735043 | 51 | 1 | 583 |
| 1339 | 216 | 4.137931 | 51 | 1 | 583 |
| 1339 | 323 | 3.636364 | 51 | 1 | 583 |
| 1339 | 398 | 4.210526 | 51 | 1 | 583 |
| 1339 | 503 | 10.08403 | 51 | 1 | 583 |
| 1339 | 552 | 3.794038 | 51 | 1 | 583 |
| 1340 | 0 | 2.459016 | 68 | 0 | 923 |
| 1341 | 0 | 1.403509 | 57 | 1 | 350 |
| 1341 | 35 | 1.980198 | 57 | 1 | 350 |
| 1341 | 98 | 1.980198 | 57 | 1 | 350 |
| 1341 | 182 | 1.834862 | 57 | 1 | 350 |
| 1341 | 273 | 1.584158 | 57 | 1 | 350 |
| 1342 | 0 | 5.555556 | 64 | 0 | 1231 |
| 1342 | 28 | 5.555556 | 64 | 0 | 1231 |
| 1342 | 84 | 2.752294 | 64 | 0 | 1231 |
| 1342 | 175 | 6.122449 | 64 | 0 | 1231 |
| 1342 | 266 | 4.067797 | 64 | 0 | 1231 |
| 1342 | 347 | 3.345281 | 64 | 0 | 1231 |
| 1342 | 411 | 2.962963 | 64 | 0 | 1231 |
| 1342 | 489 | 3.44086 | 64 | 0 | 1231 |
| 1342 | 587 | 2.93578 | 64 | 0 | 1231 |
| 1343 | 0 | 1.206637 | 81 | 0 | 1080 |
| 1343 | 91 | 1.118881 | 81 | 0 | 1080 |
| 1343 | 182 | 1.161103 | 81 | 0 | 1080 |
| 1343 | 340 | 0.394477 | 81 | 0 | 1080 |
| 1343 | 518 | 1.851852 | 81 | 0 | 1080 |

| | | | | | |
|---|---|---|---|---|---|
| 1343 | 581 | 8.602151 | 81 | 0 | 1080 |
| 1344 | 0 | 2.564103 | 70 | 0 | 841 |
| 1344 | 28 | 2.631579 | 70 | 0 | 841 |
| 1344 | 84 | 2.857143 | 70 | 0 | 841 |
| 1344 | 140 | 2.830189 | 70 | 0 | 841 |
| 1344 | 280 | 2.752294 | 70 | 0 | 841 |
| 1344 | 343 | 2.285714 | 70 | 0 | 841 |
| 1344 | 406 | 3.773585 | 70 | 0 | 841 |
| 1344 | 462 | 4.186916 | 70 | 0 | 841 |
| 1344 | 581 | 3.137255 | 70 | 0 | 841 |
| 1345 | 0 | 8.100289 | 86 | 0 | 291 |
| 1345 | 35 | 4.338843 | 86 | 0 | 291 |
| 1345 | 80 | 5.793103 | 86 | 0 | 291 |
| 1345 | 161 | 11.31313 | 86 | 0 | 291 |
| 1346 | 0 | 2.061856 | 68 | 0 | 1023 |
| 1346 | 84 | 2.197802 | 68 | 0 | 1023 |
| 1346 | 161 | 1.320132 | 68 | 0 | 1023 |
| 1346 | 238 | 0.595238 | 68 | 0 | 1023 |
| 1346 | 322 | 1.46789 | 68 | 0 | 1023 |
| 1346 | 406 | 0.37037 | 68 | 0 | 1023 |
| 1346 | 483 | 0.672269 | 68 | 0 | 1023 |
| 1346 | 553 | 0.727273 | 68 | 0 | 1023 |
| 1347 | 0 | 9.917355 | 66 | 0 | 844 |
| 1347 | 42 | 7.894737 | 66 | 0 | 844 |
| 1347 | 98 | 16.82243 | 66 | 0 | 844 |
| 1347 | 182 | 15.25424 | 66 | 0 | 844 |
| 1347 | 259 | 11.16279 | 66 | 0 | 844 |
| 1347 | 325 | 10.90909 | 66 | 0 | 844 |
| 1347 | 413 | 10.28571 | 66 | 0 | 844 |
| 1347 | 497 | 13.13869 | 66 | 0 | 844 |
| 1347 | 560 | 13.43284 | 66 | 0 | 844 |
| 1348 | 0 | 2.105263 | 82 | 0 | 1286 |
| 1348 | 25 | 2.631579 | 82 | 0 | 1286 |
| 1348 | 81 | 3.902439 | 82 | 0 | 1286 |
| 1348 | 172 | 5.042017 | 82 | 0 | 1286 |
| 1348 | 256 | 5.309735 | 82 | 0 | 1286 |
| 1348 | 347 | 5.128205 | 82 | 0 | 1286 |
| 1348 | 420 | 6.436782 | 82 | 0 | 1286 |
| 1348 | 505 | 26.37363 | 82 | 0 | 1286 |

| | | | | | |
|---|---|---|---|---|---|
| 1348 | 592 | 15.84158 | 82 | 0 | 1286 |
| 1349 | 0 | 2.242991 | 75 | 0 | 799 |
| 1349 | 70 | 1.048951 | 75 | 0 | 799 |
| 1349 | 147 | 1.019541 | 75 | 0 | 799 |
| 1349 | 213 | 4.848485 | 75 | 0 | 799 |
| 1349 | 329 | 1.363636 | 75 | 0 | 799 |
| 1349 | 414 | 3.498542 | 75 | 0 | 799 |
| 1349 | 501 | 3.478261 | 75 | 0 | 799 |
| 1349 | 581 | 1.664355 | 75 | 0 | 799 |
| 1350 | 0 | 1.905626 | 77 | 0 | 333 |
| 1350 | 73 | 1.415929 | 77 | 0 | 333 |
| 1350 | 143 | 1.190476 | 77 | 0 | 333 |
| 1351 | 0 | 5.393258 | 58 | 1 | 241 |
| 1351 | 35 | 17.77778 | 58 | 1 | 241 |
| 1351 | 91 | 17.97753 | 58 | 1 | 241 |
| 1351 | 154 | 5.882353 | 58 | 1 | 241 |
| 1351 | 240 | 8 | 58 | 1 | 241 |
| 1352 | 0 | 2.524418 | 68 | 1 | 450 |
| 1352 | 28 | 5.357143 | 68 | 1 | 450 |
| 1352 | 84 | 2.521008 | 68 | 1 | 450 |
| 1352 | 168 | 11.42857 | 68 | 1 | 450 |
| 1352 | 252 | 4.958678 | 68 | 1 | 450 |
| 1352 | 336 | 5.555556 | 68 | 1 | 450 |
| 1352 | 420 | 5.405405 | 68 | 1 | 450 |
| 1353 | 0 | 12.92308 | 79 | 1 | 30 |
| 1353 | 27 | 11.14058 | 79 | 1 | 30 |
| 1354 | 0 | 5 | 85 | 0 | 670 |
| 1354 | 28 | 3.636364 | 85 | 0 | 670 |
| 1354 | 70 | 3.921569 | 85 | 0 | 670 |
| 1354 | 161 | 3.393939 | 85 | 0 | 670 |
| 1355 | 0 | 4.651163 | 58 | 1 | 590 |
| 1355 | 28 | 4 | 58 | 1 | 590 |
| 1355 | 63 | 4.173913 | 58 | 1 | 590 |
| 1355 | 154 | 3.966942 | 58 | 1 | 590 |
| 1355 | 217 | 5.314774 | 58 | 1 | 590 |
| 1355 | 336 | 11.88119 | 58 | 1 | 590 |
| 1355 | 392 | 9.056604 | 58 | 1 | 590 |
| 1355 | 490 | 16 | 58 | 1 | 590 |
| 1355 | 553 | 14.6789 | 58 | 1 | 590 |

| | | | | | |
|---|---|---|---|---|---|
| 1356 | 0 | 4.210526 | 85 | 0 | 682 |
| 1356 | 28 | 4.752475 | 85 | 0 | 682 |
| 1356 | 63 | 5.454545 | 85 | 0 | 682 |
| 1356 | 161 | 5.714286 | 85 | 0 | 682 |
| 1356 | 259 | 8.931419 | 85 | 0 | 682 |
| 1356 | 322 | 10.32258 | 85 | 0 | 682 |
| 1356 | 431 | 13.04348 | 85 | 0 | 682 |
| 1356 | 498 | 13.04348 | 85 | 0 | 682 |
| 1356 | 588 | 13.7931 | 85 | 0 | 682 |
| 1357 | 0 | 24.63343 | 64 | 0 | 122 |
| 1357 | 36 | 11.25 | 64 | 0 | 122 |
| 1357 | 71 | 12.10084 | 64 | 0 | 122 |
| 1358 | 0 | 0.634921 | 55 | 0 | 1058 |
| 1358 | 21 | 0.634921 | 55 | 0 | 1058 |
| 1358 | 84 | 0.833333 | 55 | 0 | 1058 |
| 1358 | 168 | 2.631579 | 55 | 0 | 1058 |
| 1358 | 252 | 0.819672 | 55 | 0 | 1058 |
| 1358 | 336 | 0.847458 | 55 | 0 | 1058 |
| 1358 | 420 | 0.869565 | 55 | 0 | 1058 |
| 1358 | 504 | 0.900901 | 55 | 0 | 1058 |
| 1358 | 588 | 0.869565 | 55 | 0 | 1058 |
| 1359 | 0 | 6.338028 | 84 | 0 | 1100 |
| 1359 | 28 | 8.362369 | 84 | 0 | 1100 |
| 1359 | 77 | 11.25 | 84 | 0 | 1100 |
| 1359 | 182 | 6.153846 | 84 | 0 | 1100 |
| 1359 | 231 | 4.114286 | 84 | 0 | 1100 |
| 1359 | 336 | 12.20339 | 84 | 0 | 1100 |
| 1359 | 399 | 5.882353 | 84 | 0 | 1100 |
| 1359 | 490 | 7.03125 | 84 | 0 | 1100 |
| 1360 | 0 | 5.172414 | 72 | 0 | 892 |
| 1360 | 28 | 5.263158 | 72 | 0 | 892 |
| 1360 | 56 | 5.405405 | 72 | 0 | 892 |
| 1360 | 140 | 5.405405 | 72 | 0 | 892 |
| 1360 | 252 | 5.084746 | 72 | 0 | 892 |
| 1360 | 336 | 5.172414 | 72 | 0 | 892 |
| 1360 | 420 | 5.357143 | 72 | 0 | 892 |
| 1360 | 504 | 5.405405 | 72 | 0 | 892 |
| 1360 | 588 | 5.217391 | 72 | 0 | 892 |
| 1361 | 0 | 2.142857 | 74 | 0 | 813 |

| | | | | | |
|---|---|---|---|---|---|
| 1361 | 28 | 3.636364 | 74 | 0 | 813 |
| 1361 | 91 | 2.735043 | 74 | 0 | 813 |
| 1361 | 154 | 7.079646 | 74 | 0 | 813 |
| 1361 | 245 | 6.779661 | 74 | 0 | 813 |
| 1361 | 336 | 3.812117 | 74 | 0 | 813 |
| 1361 | 397 | 3.779528 | 74 | 0 | 813 |
| 1361 | 490 | 6.837607 | 74 | 0 | 813 |
| 1361 | 567 | 4.478806 | 74 | 0 | 813 |
| 1362 | 0 | 3.463203 | 74 | 1 | 421 |
| 1362 | 49 | 3.921569 | 74 | 1 | 421 |
| 1362 | 175 | 7.142857 | 74 | 1 | 421 |
| 1363 | 0 | 0.634921 | 80 | 0 | 318 |
| 1363 | 28 | 0.696864 | 80 | 0 | 318 |
| 1363 | 84 | 1.169591 | 80 | 0 | 318 |
| 1363 | 168 | 1.190476 | 80 | 0 | 318 |
| 1363 | 252 | 1.201201 | 80 | 0 | 318 |
| 1364 | 0 | 5.128205 | 80 | 0 | 813 |
| 1364 | 28 | 5 | 80 | 0 | 813 |
| 1364 | 84 | 7.692308 | 80 | 0 | 813 |
| 1364 | 168 | 5.172414 | 80 | 0 | 813 |
| 1364 | 252 | 1.785714 | 80 | 0 | 813 |
| 1364 | 336 | 0.909091 | 80 | 0 | 813 |
| 1364 | 420 | 0.900901 | 80 | 0 | 813 |
| 1364 | 476 | 0.970874 | 80 | 0 | 813 |
| 1365 | 0 | 5.042017 | 66 | 0 | 925 |
| 1365 | 28 | 4.8 | 66 | 0 | 925 |
| 1365 | 84 | 4.724409 | 66 | 0 | 925 |
| 1365 | 168 | 4.761905 | 66 | 0 | 925 |
| 1365 | 252 | 4.6875 | 66 | 0 | 925 |
| 1365 | 336 | 4.977778 | 66 | 0 | 925 |
| 1365 | 420 | 3.75 | 66 | 0 | 925 |
| 1365 | 511 | 4.83871 | 66 | 0 | 925 |
| 1365 | 595 | 4.958678 | 66 | 0 | 925 |
| 1366 | 0 | 0.694732 | 68 | 1 | 356 |
| 1366 | 89 | 5.412371 | 68 | 1 | 356 |
| 1366 | 170 | 3.916084 | 68 | 1 | 356 |
| 1366 | 222 | 1.68607 | 68 | 1 | 356 |
| 1366 | 353 | 24.24242 | 68 | 1 | 356 |
| 1367 | 0 | 13.18681 | 58 | 0 | 225 |

| | | | | | |
|---|---|---|---|---|---|
| 1367 | 28 | 12.63158 | 58 | 0 | 225 |
| 1367 | 84 | 17.14286 | 58 | 0 | 225 |
| 1367 | 168 | 13.18681 | 58 | 0 | 225 |
| 1368 | 0 | 2.276423 | 72 | 0 | 1147 |
| 1368 | 30 | 4.918033 | 72 | 0 | 1147 |
| 1368 | 86 | 5.357143 | 72 | 0 | 1147 |
| 1368 | 170 | 18.75 | 72 | 0 | 1147 |
| 1368 | 254 | 21.05263 | 72 | 0 | 1147 |
| 1368 | 338 | 17.14286 | 72 | 0 | 1147 |
| 1368 | 422 | 18.18182 | 72 | 0 | 1147 |
| 1368 | 506 | 27.58621 | 72 | 0 | 1147 |
| 1368 | 597 | 36.52174 | 72 | 0 | 1147 |
| 1369 | 0 | 7.784986 | 66 | 0 | 825 |
| 1369 | 26 | 10.09009 | 66 | 0 | 825 |
| 1369 | 91 | 16.94118 | 66 | 0 | 825 |
| 1369 | 271 | 18.18182 | 66 | 0 | 825 |
| 1369 | 355 | 26.86567 | 66 | 0 | 825 |
| 1369 | 411 | 25.71429 | 66 | 0 | 825 |
| 1369 | 495 | 27.69231 | 66 | 0 | 825 |
| 1370 | 0 | 1.12782 | 74 | 0 | 882 |
| 1370 | 28 | 2.419355 | 74 | 0 | 882 |
| 1370 | 84 | 2.459016 | 74 | 0 | 882 |
| 1370 | 182 | 0.902256 | 74 | 0 | 882 |
| 1370 | 252 | 0.569801 | 74 | 0 | 882 |
| 1370 | 350 | 0.555556 | 74 | 0 | 882 |
| 1370 | 441 | 2.702703 | 74 | 0 | 882 |
| 1370 | 497 | 2.542373 | 74 | 0 | 882 |
| 1370 | 565 | 1.058201 | 74 | 0 | 882 |
| 1371 | 0 | 6.122449 | 73 | 1 | 624 |
| 1371 | 27 | 6 | 73 | 1 | 624 |
| 1371 | 83 | 7.535322 | 73 | 1 | 624 |
| 1371 | 196 | 9.411765 | 73 | 1 | 624 |
| 1371 | 286 | 11.25 | 73 | 1 | 624 |
| 1371 | 335 | 8.888889 | 73 | 1 | 624 |
| 1371 | 440 | 16.56805 | 73 | 1 | 624 |
| 1371 | 530 | 15.78947 | 73 | 1 | 624 |
| 1371 | 588 | 16.43836 | 73 | 1 | 624 |
| 1372 | 0 | 2.051282 | 75 | 1 | 387 |
| 1372 | 21 | 2.521008 | 75 | 1 | 387 |

| | | | | | |
|---|---|---|---|---|---|
| 1372 | 77 | 1.983471 | 75 | 1 | 387 |
| 1372 | 231 | 5.454545 | 75 | 1 | 387 |
| 1372 | 364 | 4.948454 | 75 | 1 | 387 |
| 1373 | 0 | 0.9375 | 83 | 0 | 596 |
| 1373 | 32 | 1.025641 | 83 | 0 | 596 |
| 1373 | 61 | 1.052632 | 83 | 0 | 596 |
| 1373 | 159 | 4.571429 | 83 | 0 | 596 |
| 1373 | 245 | 0.974026 | 83 | 0 | 596 |
| 1373 | 336 | 3.960396 | 83 | 0 | 596 |
| 1373 | 427 | 2.424242 | 83 | 0 | 596 |
| 1373 | 518 | 2.298851 | 83 | 0 | 596 |
| 1373 | 588 | 5.660377 | 83 | 0 | 596 |
| 1374 | 0 | 2.459016 | 70 | 1 | 1117 |
| 1374 | 28 | 0.492308 | 70 | 1 | 1117 |
| 1374 | 84 | 0.544588 | 70 | 1 | 1117 |
| 1374 | 168 | 7.079646 | 70 | 1 | 1117 |
| 1374 | 238 | 1.851852 | 70 | 1 | 1117 |
| 1374 | 301 | 1.612903 | 70 | 1 | 1117 |
| 1374 | 399 | 1.37931 | 70 | 1 | 1117 |
| 1374 | 462 | 1.355932 | 70 | 1 | 1117 |
| 1374 | 553 | 2.727273 | 70 | 1 | 1117 |
| 1375 | 0 | 2.702703 | 73 | 0 | 557 |
| 1375 | 32 | 2.702703 | 73 | 0 | 557 |
| 1375 | 88 | 5.940594 | 73 | 0 | 557 |
| 1375 | 172 | 13.18681 | 73 | 0 | 557 |
| 1375 | 256 | 5.128205 | 73 | 0 | 557 |
| 1376 | 0 | 1.754386 | 82 | 0 | 1013 |
| 1376 | 42 | 1.415094 | 82 | 0 | 1013 |
| 1376 | 280 | 0.859291 | 82 | 0 | 1013 |
| 1376 | 343 | 1.250651 | 82 | 0 | 1013 |
| 1377 | 0 | 0.533333 | 68 | 0 | 1086 |
| 1377 | 28 | 0.529101 | 68 | 0 | 1086 |
| 1377 | 84 | 0.464576 | 68 | 0 | 1086 |
| 1377 | 175 | 0.574713 | 68 | 0 | 1086 |
| 1378 | 0 | 5.506391 | 92 | 0 | 684 |
| 1378 | 27 | 5.860806 | 92 | 0 | 684 |
| 1378 | 76 | 4.958678 | 92 | 0 | 684 |
| 1378 | 174 | 2.413273 | 92 | 0 | 684 |
| 1378 | 228 | 2.991453 | 92 | 0 | 684 |

| | | | | | |
|---|---|---|---|---|---|
| 1378 | 347 | 7.894737 | 92 | 0 | 684 |
| 1378 | 396 | 6.349206 | 92 | 0 | 684 |
| 1378 | 501 | 8.490566 | 92 | 0 | 684 |
| 1378 | 557 | 8.571429 | 92 | 0 | 684 |
| 1379 | 0 | 7.881773 | 55 | 0 | 1138 |
| 1379 | 63 | 7.228916 | 55 | 0 | 1138 |
| 1379 | 119 | 6.666667 | 55 | 0 | 1138 |
| 1379 | 225 | 10.58824 | 55 | 0 | 1138 |
| 1380 | 0 | 5.128205 | 80 | 0 | 1212 |
| 1380 | 84 | 1.52381 | 80 | 0 | 1212 |
| 1380 | 189 | 1.259843 | 80 | 0 | 1212 |
| 1380 | 245 | 0.806452 | 80 | 0 | 1212 |
| 1381 | 0 | 2.830189 | 71 | 1 | 396 |
| 1381 | 28 | 4.081633 | 71 | 1 | 396 |
| 1381 | 91 | 3.669725 | 71 | 1 | 396 |
| 1381 | 154 | 4.941176 | 71 | 1 | 396 |
| 1381 | 213 | 3.378922 | 71 | 1 | 396 |
| 1381 | 343 | 9.545455 | 71 | 1 | 396 |
| 1382 | 0 | 0.659341 | 85 | 1 | 77 |
| 1382 | 75 | 5.581395 | 85 | 1 | 77 |
| 1383 | 0 | 2.330097 | 61 | 1 | 905 |
| 1383 | 35 | 2.884615 | 61 | 1 | 905 |
| 1383 | 84 | 1.558442 | 61 | 1 | 905 |
| 1383 | 161 | 2.564103 | 61 | 1 | 905 |
| 1383 | 224 | 1.10957 | 61 | 1 | 905 |
| 1383 | 364 | 3.061224 | 61 | 1 | 905 |
| 1383 | 420 | 6.122449 | 61 | 1 | 905 |
| 1383 | 504 | 5.940594 | 61 | 1 | 905 |
| 1383 | 588 | 5.555556 | 61 | 1 | 905 |
| 1384 | 0 | 2 | 83 | 0 | 792 |
| 1384 | 63 | 2.364865 | 83 | 0 | 792 |
| 1384 | 182 | 1.442308 | 83 | 0 | 792 |
| 1385 | 0 | 1.503759 | 66 | 1 | 450 |
| 1385 | 28 | 1.550388 | 66 | 1 | 450 |
| 1385 | 84 | 1.680672 | 66 | 1 | 450 |
| 1385 | 168 | 1.626016 | 66 | 1 | 450 |
| 1385 | 254 | 1.5625 | 66 | 1 | 450 |
| 1385 | 338 | 1.801802 | 66 | 1 | 450 |
| 1385 | 422 | 4.950495 | 66 | 1 | 450 |

| | | | | | |
|---|---|---|---|---|---|
| 1386 | 0 | 1.043478 | 87 | 0 | 166 |
| 1386 | 28 | 1.061947 | 87 | 0 | 166 |
| 1387 | 0 | 2.201835 | 80 | 1 | 48 |
| 1387 | 35 | 5 | 80 | 1 | 48 |
| 1388 | 0 | 10.81081 | 66 | 0 | 995 |
| 1388 | 35 | 2.242991 | 66 | 0 | 995 |
| 1388 | 98 | 3.061224 | 66 | 0 | 995 |
| 1388 | 154 | 6.593407 | 66 | 0 | 995 |
| 1388 | 294 | 5 | 66 | 0 | 995 |
| 1388 | 357 | 6.25 | 66 | 0 | 995 |
| 1388 | 448 | 7.44186 | 66 | 0 | 995 |
| 1388 | 518 | 13.47368 | 66 | 0 | 995 |
| 1388 | 553 | 14.54545 | 66 | 0 | 995 |
| 1389 | 0 | 4.83871 | 57 | 0 | 982 |
| 1389 | 28 | 2.362205 | 57 | 0 | 982 |
| 1389 | 84 | 0.976801 | 57 | 0 | 982 |
| 1389 | 189 | 1.754386 | 57 | 0 | 982 |
| 1389 | 252 | 1.67916 | 57 | 0 | 982 |
| 1389 | 337 | 1.639344 | 57 | 0 | 982 |
| 1389 | 428 | 3.333333 | 57 | 0 | 982 |
| 1389 | 491 | 3.508772 | 57 | 0 | 982 |
| 1389 | 603 | 1.666667 | 57 | 0 | 982 |
| 1390 | 0 | 2.752294 | 75 | 0 | 169 |
| 1390 | 28 | 2.631579 | 75 | 0 | 169 |
| 1390 | 84 | 2.727273 | 75 | 0 | 169 |
| 1391 | 0 | 5.274725 | 79 | 1 | 195 |
| 1391 | 35 | 6.666667 | 79 | 1 | 195 |
| 1391 | 91 | 15.58442 | 79 | 1 | 195 |
| 1392 | 0 | 6.382979 | 73 | 0 | 1051 |
| 1392 | 28 | 4.948454 | 73 | 0 | 1051 |
| 1392 | 105 | 11.03448 | 73 | 0 | 1051 |
| 1392 | 182 | 10.21277 | 73 | 0 | 1051 |
| 1392 | 273 | 9.320388 | 73 | 0 | 1051 |
| 1392 | 329 | 13.18681 | 73 | 0 | 1051 |
| 1392 | 413 | 8.163265 | 73 | 0 | 1051 |
| 1392 | 497 | 12.90323 | 73 | 0 | 1051 |
| 1393 | 0 | 5.263158 | 82 | 0 | 1224 |
| 1393 | 28 | 4.491979 | 82 | 0 | 1224 |
| 1393 | 62 | 3.809524 | 82 | 0 | 1224 |

| | | | | | |
|---|---|---|---|---|---|
| 1393 | 146 | 6.095791 | 82 | 0 | 1224 |
| 1393 | 230 | 4.008112 | 82 | 0 | 1224 |
| 1393 | 328 | 6.162876 | 82 | 0 | 1224 |
| 1393 | 426 | 13.16099 | 82 | 0 | 1224 |
| 1393 | 510 | 14.28571 | 82 | 0 | 1224 |
| 1393 | 594 | 16.82243 | 82 | 0 | 1224 |
| 1394 | 0 | 2.654867 | 73 | 1 | 467 |
| 1394 | 28 | 3.069054 | 73 | 1 | 467 |
| 1394 | 84 | 2.702703 | 73 | 1 | 467 |
| 1394 | 170 | 2.573529 | 73 | 1 | 467 |
| 1394 | 240 | 2.912621 | 73 | 1 | 467 |
| 1394 | 331 | 4.210526 | 73 | 1 | 467 |
| 1394 | 436 | 7.407407 | 73 | 1 | 467 |
| 1395 | 0 | 6.382979 | 68 | 1 | 223 |
| 1395 | 28 | 13.95349 | 68 | 1 | 223 |
| 1395 | 56 | 4.651163 | 68 | 1 | 223 |
| 1395 | 140 | 4.597701 | 68 | 1 | 223 |
| 1396 | 0 | 2.068966 | 60 | 1 | 1093 |
| 1396 | 28 | 0.188976 | 60 | 1 | 1093 |
| 1396 | 70 | 0.196721 | 60 | 1 | 1093 |
| 1396 | 168 | 0.206897 | 60 | 1 | 1093 |
| 1396 | 231 | 0.214286 | 60 | 1 | 1093 |
| 1396 | 308 | 0.201681 | 60 | 1 | 1093 |
| 1396 | 392 | 3.669725 | 60 | 1 | 1093 |
| 1397 | 0 | 12.44444 | 66 | 1 | 58 |
| 1397 | 21 | 10.26393 | 66 | 1 | 58 |
| 1398 | 0 | 4.705882 | 77 | 0 | 251 |
| 1398 | 35 | 5.882353 | 77 | 0 | 251 |
| 1398 | 91 | 12.2449 | 77 | 0 | 251 |
| 1398 | 175 | 17.3913 | 77 | 0 | 251 |
| 1398 | 245 | 22.42991 | 77 | 0 | 251 |
| 1399 | 0 | 0.884956 | 71 | 1 | 1336 |
| 1399 | 28 | 1.818182 | 71 | 1 | 1336 |
| 1399 | 112 | 3.603604 | 71 | 1 | 1336 |
| 1399 | 175 | 3.571429 | 71 | 1 | 1336 |
| 1399 | 259 | 3.478261 | 71 | 1 | 1336 |
| 1399 | 350 | 2.807018 | 71 | 1 | 1336 |
| 1399 | 420 | 3.809524 | 71 | 1 | 1336 |
| 1399 | 504 | 2.962963 | 71 | 1 | 1336 |

| | | | | | |
|---|---|---|---|---|---|
| 1399 | 574 | 2.990654 | 71 | 1 | 1336 |
| 1400 | 0 | 2.752294 | 44 | 1 | 433 |
| 1400 | 27 | 2.812186 | 44 | 1 | 433 |
| 1400 | 56 | 4.752475 | 44 | 1 | 433 |
| 1400 | 119 | 5.679513 | 44 | 1 | 433 |
| 1400 | 216 | 12.12121 | 44 | 1 | 433 |
| 1400 | 336 | 10.43478 | 44 | 1 | 433 |
| 1400 | 399 | 37.1134 | 44 | 1 | 433 |
| 1401 | 0 | 4.761905 | 79 | 1 | 224 |
| 1401 | 70 | 9.598172 | 79 | 1 | 224 |
| 1401 | 140 | 33.51064 | 79 | 1 | 224 |
| 1402 | 0 | 4.166667 | 81 | 0 | 491 |
| 1402 | 112 | 2.807018 | 81 | 0 | 491 |
| 1402 | 196 | 3.100775 | 81 | 0 | 491 |
| 1402 | 238 | 4.545455 | 81 | 0 | 491 |
| 1403 | 0 | 8.421053 | 72 | 1 | 386 |
| 1403 | 21 | 3.092784 | 72 | 1 | 386 |
| 1403 | 77 | 1.664355 | 72 | 1 | 386 |
| 1403 | 175 | 2.970297 | 72 | 1 | 386 |
| 1403 | 231 | 4.660194 | 72 | 1 | 386 |
| 1404 | 0 | 3.076923 | 61 | 1 | 998 |
| 1404 | 42 | 3.72093 | 61 | 1 | 998 |
| 1404 | 105 | 4.444444 | 61 | 1 | 998 |
| 1404 | 147 | 5 | 61 | 1 | 998 |
| 1404 | 262 | 3.966942 | 61 | 1 | 998 |
| 1404 | 339 | 4.918033 | 61 | 1 | 998 |
| 1404 | 409 | 3.934426 | 61 | 1 | 998 |
| 1404 | 521 | 3.934426 | 61 | 1 | 998 |
| 1404 | 556 | 3.902439 | 61 | 1 | 998 |
| 1405 | 0 | 1.73913 | 69 | 0 | 624 |
| 1405 | 28 | 1.415929 | 69 | 0 | 624 |
| 1405 | 161 | 1.441441 | 69 | 0 | 624 |
| 1405 | 266 | 1.616162 | 69 | 0 | 624 |
| 1405 | 329 | 2.330097 | 69 | 0 | 624 |
| 1405 | 399 | 1.454545 | 69 | 0 | 624 |
| 1405 | 469 | 2.44898 | 69 | 0 | 624 |
| 1405 | 567 | 6.25 | 69 | 0 | 624 |
| 1406 | 0 | 2.573529 | 62 | 0 | 667 |
| 1406 | 35 | 1.590909 | 62 | 0 | 667 |

| | | | | | |
|---|---|---|---|---|---|
| 1406 | 92 | 5.490196 | 62 | 0 | 667 |
| 1407 | 0 | 8.823529 | 78 | 0 | 435 |
| 1407 | 28 | 4.136912 | 78 | 0 | 435 |
| 1407 | 85 | 6.61157 | 78 | 0 | 435 |
| 1407 | 161 | 5.957447 | 78 | 0 | 435 |
| 1407 | 231 | 3.844394 | 78 | 0 | 435 |
| 1408 | 0 | 3.603604 | 72 | 1 | 1309 |
| 1408 | 28 | 1.626016 | 72 | 1 | 1309 |
| 1408 | 84 | 4.123711 | 72 | 1 | 1309 |
| 1408 | 168 | 6.78925 | 72 | 1 | 1309 |
| 1408 | 251 | 11.00917 | 72 | 1 | 1309 |
| 1408 | 335 | 10.61947 | 72 | 1 | 1309 |
| 1408 | 413 | 8.108108 | 72 | 1 | 1309 |
| 1408 | 497 | 8.716707 | 72 | 1 | 1309 |
| 1408 | 588 | 10.71429 | 72 | 1 | 1309 |
| 1409 | 0 | 5.052632 | 61 | 1 | 103 |
| 1409 | 35 | 8.695652 | 61 | 1 | 103 |
| 1409 | 56 | 7.15198 | 61 | 1 | 103 |
| 1410 | 0 | 0.386473 | 85 | 0 | 589 |
| 1410 | 98 | 0.448934 | 85 | 0 | 589 |
| 1411 | 0 | 1.818182 | 41 | 0 | 561 |
| 1411 | 77 | 2.631579 | 41 | 0 | 561 |
| 1411 | 140 | 2.027027 | 41 | 0 | 561 |
| 1411 | 224 | 3.752513 | 41 | 0 | 561 |
| 1411 | 315 | 3.902439 | 41 | 0 | 561 |
| 1411 | 385 | 5.219012 | 41 | 0 | 561 |
| 1411 | 511 | 5.112599 | 41 | 0 | 561 |
| 1411 | 560 | 9.52381 | 41 | 0 | 561 |
| 1412 | 0 | 2.586207 | 81 | 0 | 1146 |
| 1412 | 28 | 1.6 | 81 | 0 | 1146 |
| 1412 | 84 | 1.311475 | 81 | 0 | 1146 |
| 1413 | 0 | 3.738318 | 85 | 1 | 776 |
| 1414 | 0 | 3.571429 | 73 | 0 | 827 |
| 1414 | 70 | 2.807018 | 73 | 0 | 827 |
| 1414 | 154 | 3.234501 | 73 | 0 | 827 |
| 1414 | 245 | 2.402402 | 73 | 0 | 827 |
| 1414 | 336 | 3.394625 | 73 | 0 | 827 |
| 1414 | 385 | 3.394625 | 73 | 0 | 827 |
| 1414 | 490 | 2.588997 | 73 | 0 | 827 |

| | | | | | |
|---|---|---|---|---|---|
| 1414 | 553 | 2.539683 | 73 | 0 | 827 |
| 1415 | 0 | 3.703704 | 76 | 0 | 1212 |
| 1415 | 28 | 6.060606 | 76 | 0 | 1212 |
| 1415 | 84 | 5.555556 | 76 | 0 | 1212 |
| 1415 | 196 | 5.504587 | 76 | 0 | 1212 |
| 1415 | 252 | 5.405405 | 76 | 0 | 1212 |
| 1415 | 336 | 5.309735 | 76 | 0 | 1212 |
| 1415 | 448 | 5.042017 | 76 | 0 | 1212 |
| 1415 | 512 | 5.363985 | 76 | 0 | 1212 |
| 1415 | 568 | 5.185185 | 76 | 0 | 1212 |
| 1416 | 0 | 9.183673 | 61 | 0 | 729 |
| 1416 | 49 | 7.964602 | 61 | 0 | 729 |
| 1416 | 161 | 7.142857 | 61 | 0 | 729 |
| 1416 | 224 | 6.837607 | 61 | 0 | 729 |
| 1416 | 350 | 6.896552 | 61 | 0 | 729 |
| 1416 | 406 | 7.33945 | 61 | 0 | 729 |
| 1416 | 532 | 7.272727 | 61 | 0 | 729 |
| 1416 | 595 | 6.956522 | 61 | 0 | 729 |
| 1417 | 0 | 5.357143 | 71 | 1 | 450 |
| 1417 | 28 | 5.454545 | 71 | 1 | 450 |
| 1417 | 84 | 5.357143 | 71 | 1 | 450 |
| 1417 | 168 | 12.5 | 71 | 1 | 450 |
| 1417 | 252 | 21.05263 | 71 | 1 | 450 |
| 1417 | 343 | 20.68966 | 71 | 1 | 450 |
| 1417 | 420 | 24 | 71 | 1 | 450 |
| 1418 | 0 | 4.093567 | 41 | 0 | 205 |
| 1418 | 17 | 1.457574 | 41 | 0 | 205 |
| 1418 | 108 | 0.885796 | 41 | 0 | 205 |
| 1419 | 0 | 9.320388 | 86 | 0 | 665 |
| 1419 | 70 | 11.11111 | 86 | 0 | 665 |
| 1419 | 133 | 16.63366 | 86 | 0 | 665 |
| 1420 | 0 | 16 | 78 | 0 | 109 |
| 1420 | 35 | 23.07692 | 78 | 0 | 109 |
| 1420 | 56 | 24.74227 | 78 | 0 | 109 |
| 1421 | 0 | 6.25 | 64 | 1 | 465 |
| 1421 | 28 | 5.940594 | 64 | 1 | 465 |
| 1421 | 91 | 10.32258 | 64 | 1 | 465 |
| 1421 | 154 | 18.94737 | 64 | 1 | 465 |
| 1421 | 238 | 17.47573 | 64 | 1 | 465 |

| | | | | | |
|---|---|---|---|---|---|
| 1421 | 329 | 16.07656 | 64 | 1 | 465 |
| 1421 | 420 | 16.17978 | 64 | 1 | 465 |
| 1422 | 0 | 3.508772 | 54 | 0 | 743 |
| 1422 | 42 | 5.263158 | 54 | 0 | 743 |
| 1422 | 98 | 11.21495 | 54 | 0 | 743 |
| 1422 | 182 | 9.756098 | 54 | 0 | 743 |
| 1422 | 266 | 3.305785 | 54 | 0 | 743 |
| 1422 | 364 | 4.918033 | 54 | 0 | 743 |
| 1422 | 434 | 3.966942 | 54 | 0 | 743 |
| 1422 | 532 | 2.689076 | 54 | 0 | 743 |
| 1422 | 602 | 2.727273 | 54 | 0 | 743 |
| 1423 | 0 | 7.692308 | 59 | 1 | 206 |
| 1423 | 28 | 3.149606 | 59 | 1 | 206 |
| 1423 | 56 | 3.305785 | 59 | 1 | 206 |
| 1423 | 147 | 5.940594 | 59 | 1 | 206 |
| 1424 | 0 | 1.374065 | 67 | 1 | 373 |
| 1424 | 27 | 1.477768 | 67 | 1 | 373 |
| 1424 | 91 | 2.162162 | 67 | 1 | 373 |
| 1424 | 161 | 1.595745 | 67 | 1 | 373 |
| 1424 | 273 | 4.590164 | 67 | 1 | 373 |
| 1425 | 0 | 3.603604 | 59 | 0 | 967 |
| 1425 | 28 | 3.738318 | 59 | 0 | 967 |
| 1425 | 84 | 3.508772 | 59 | 0 | 967 |
| 1425 | 175 | 1.666667 | 59 | 0 | 967 |
| 1425 | 259 | 1.709402 | 59 | 0 | 967 |
| 1425 | 350 | 1.311475 | 59 | 0 | 967 |
| 1425 | 406 | 0.97561 | 59 | 0 | 967 |
| 1425 | 501 | 1.25 | 59 | 0 | 967 |
| 1426 | 0 | 2.586207 | 73 | 0 | 806 |
| 1426 | 28 | 4.72973 | 73 | 0 | 806 |
| 1426 | 84 | 12 | 73 | 0 | 806 |
| 1426 | 168 | 9.320388 | 73 | 0 | 806 |
| 1426 | 343 | 14.45783 | 73 | 0 | 806 |
| 1426 | 427 | 6.976744 | 73 | 0 | 806 |
| 1426 | 511 | 5 | 73 | 0 | 806 |
| 1427 | 0 | 8.035714 | 84 | 0 | 1167 |
| 1427 | 28 | 7.894737 | 84 | 0 | 1167 |
| 1427 | 84 | 10.52632 | 84 | 0 | 1167 |
| 1427 | 182 | 8.648649 | 84 | 0 | 1167 |

| | | | | | |
|---|---|---|---|---|---|
| 1427 | 238 | 10.71429 | 84 | 0 | 1167 |
| 1427 | 329 | 10.90909 | 84 | 0 | 1167 |
| 1427 | 413 | 4.477612 | 84 | 0 | 1167 |
| 1427 | 504 | 4.285714 | 84 | 0 | 1167 |
| 1427 | 595 | 10 | 84 | 0 | 1167 |
| 1428 | 0 | 5.217391 | 67 | 1 | 275 |
| 1428 | 28 | 5.042017 | 67 | 1 | 275 |
| 1428 | 84 | 4.485981 | 67 | 1 | 275 |
| 1428 | 173 | 8.974359 | 67 | 1 | 275 |
| 1428 | 244 | 6.160616 | 67 | 1 | 275 |
| 1429 | 0 | 12.5 | 70 | 1 | 169 |
| 1429 | 28 | 13.18681 | 70 | 1 | 169 |
| 1429 | 84 | 16.66667 | 70 | 1 | 169 |
| 1430 | 0 | 5.624372 | 50 | 1 | 547 |
| 1430 | 29 | 7.804878 | 50 | 1 | 547 |
| 1430 | 91 | 6.122449 | 50 | 1 | 547 |
| 1430 | 168 | 11.11111 | 50 | 1 | 547 |
| 1430 | 259 | 17.82178 | 50 | 1 | 547 |
| 1430 | 315 | 17.37931 | 50 | 1 | 547 |
| 1430 | 445 | 18.46154 | 50 | 1 | 547 |
| 1430 | 546 | 18.46154 | 50 | 1 | 547 |
| 1431 | 0 | 5.607477 | 88 | 0 | 1002 |
| 1431 | 28 | 5.607477 | 88 | 0 | 1002 |
| 1431 | 84 | 8.490566 | 88 | 0 | 1002 |
| 1431 | 168 | 4.672897 | 88 | 0 | 1002 |
| 1431 | 252 | 8.333333 | 88 | 0 | 1002 |
| 1431 | 336 | 8.333333 | 88 | 0 | 1002 |
| 1431 | 420 | 2.494802 | 88 | 0 | 1002 |
| 1431 | 511 | 10.11236 | 88 | 0 | 1002 |
| 1431 | 581 | 10.71429 | 88 | 0 | 1002 |
| 1432 | 0 | 10.52632 | 78 | 0 | 1023 |
| 1432 | 28 | 4.481793 | 78 | 0 | 1023 |
| 1432 | 63 | 4.301075 | 78 | 0 | 1023 |
| 1432 | 182 | 4.137931 | 78 | 0 | 1023 |
| 1432 | 245 | 5.128205 | 78 | 0 | 1023 |
| 1432 | 308 | 2.402402 | 78 | 0 | 1023 |
| 1432 | 434 | 4.102564 | 78 | 0 | 1023 |
| 1432 | 497 | 10.08403 | 78 | 0 | 1023 |
| 1432 | 560 | 12.56231 | 78 | 0 | 1023 |

| | | | | | |
|---|---|---|---|---|---|
| 1433 | 0 | 6.130268 | 83 | 1 | 651 |
| 1433 | 21 | 4.395604 | 83 | 1 | 651 |
| 1433 | 77 | 2.886598 | 83 | 1 | 651 |
| 1433 | 154 | 3.542062 | 83 | 1 | 651 |
| 1433 | 245 | 1.923077 | 83 | 1 | 651 |
| 1433 | 322 | 2.614379 | 83 | 1 | 651 |
| 1433 | 413 | 5.243446 | 83 | 1 | 651 |
| 1433 | 501 | 4.123711 | 83 | 1 | 651 |
| 1433 | 585 | 4.347826 | 83 | 1 | 651 |
| 1434 | 0 | 1.414141 | 79 | 1 | 8 |
| 1435 | 0 | 2.735043 | 32 | 0 | 575 |
| 1435 | 28 | 2.479339 | 32 | 0 | 575 |
| 1435 | 84 | 2.5 | 32 | 0 | 575 |
| 1435 | 175 | 2.564103 | 32 | 0 | 575 |
| 1435 | 238 | 2.586207 | 32 | 0 | 575 |
| 1435 | 329 | 3.809524 | 32 | 0 | 575 |
| 1435 | 420 | 3.305785 | 32 | 0 | 575 |
| 1435 | 511 | 2.105263 | 32 | 0 | 575 |
| 1435 | 574 | 2.962963 | 32 | 0 | 575 |
| 1436 | 0 | 28.57143 | 67 | 0 | 1102 |
| 1436 | 36 | 15.58442 | 67 | 0 | 1102 |
| 1437 | 0 | 1.391304 | 72 | 0 | 1001 |
| 1437 | 105 | 1.441441 | 72 | 0 | 1001 |
| 1437 | 166 | 3.240741 | 72 | 0 | 1001 |
| 1437 | 245 | 1.769912 | 72 | 0 | 1001 |
| 1437 | 329 | 1.680672 | 72 | 0 | 1001 |
| 1437 | 413 | 1.680672 | 72 | 0 | 1001 |
| 1437 | 497 | 1.834862 | 72 | 0 | 1001 |
| 1437 | 581 | 1.769912 | 72 | 0 | 1001 |
| 1438 | 0 | 2.086957 | 76 | 0 | 738 |
| 1438 | 35 | 1.754386 | 76 | 0 | 738 |
| 1438 | 77 | 3.846154 | 76 | 0 | 738 |
| 1438 | 154 | 2.162162 | 76 | 0 | 738 |
| 1438 | 273 | 3.809524 | 76 | 0 | 738 |
| 1438 | 357 | 4.40367 | 76 | 0 | 738 |
| 1438 | 427 | 1.530612 | 76 | 0 | 738 |
| 1438 | 497 | 1.754386 | 76 | 0 | 738 |
| 1438 | 588 | 3.265306 | 76 | 0 | 738 |
| 1439 | 0 | 11.76471 | 57 | 0 | 1009 |

| | | | | | |
|---|---|---|---|---|---|
| 1439 | 28 | 18.5567 | 57 | 0 | 1009 |
| 1439 | 91 | 13.09091 | 57 | 0 | 1009 |
| 1439 | 147 | 8.181818 | 57 | 0 | 1009 |
| 1439 | 238 | 16.07143 | 57 | 0 | 1009 |
| 1439 | 329 | 7.559055 | 57 | 0 | 1009 |
| 1439 | 420 | 10.08403 | 57 | 0 | 1009 |
| 1439 | 483 | 10.16949 | 57 | 0 | 1009 |
| 1439 | 581 | 9.69697 | 57 | 0 | 1009 |
| 1440 | 0 | 2.782609 | 72 | 1 | 160 |
| 1440 | 35 | 6.315789 | 72 | 1 | 160 |
| 1440 | 98 | 4.363636 | 72 | 1 | 160 |
| 1441 | 0 | 13.89578 | 43 | 0 | 1163 |
| 1441 | 31 | 9.864944 | 43 | 0 | 1163 |
| 1441 | 115 | 7.887324 | 43 | 0 | 1163 |
| 1441 | 182 | 1.812102 | 43 | 0 | 1163 |
| 1441 | 255 | 4.363636 | 43 | 0 | 1163 |
| 1441 | 374 | 0.511727 | 43 | 0 | 1163 |
| 1441 | 423 | 0.548571 | 43 | 0 | 1163 |
| 1441 | 500 | 0.535714 | 43 | 0 | 1163 |
| 1441 | 574 | 0.562061 | 43 | 0 | 1163 |
| 1442 | 0 | 4.334365 | 51 | 0 | 1057 |
| 1442 | 34 | 4.713805 | 51 | 0 | 1057 |
| 1442 | 102 | 4.444444 | 51 | 0 | 1057 |
| 1442 | 193 | 6 | 51 | 0 | 1057 |
| 1442 | 277 | 9.448819 | 51 | 0 | 1057 |
| 1442 | 361 | 5.309735 | 51 | 0 | 1057 |
| 1442 | 431 | 8.807339 | 51 | 0 | 1057 |
| 1442 | 522 | 11.53846 | 51 | 0 | 1057 |
| 1442 | 606 | 5.357143 | 51 | 0 | 1057 |
| 1443 | 35 | 3.018868 | 54 | 0 | 1069 |
| 1443 | 154 | 5.940594 | 54 | 0 | 1069 |
| 1443 | 259 | 4.705882 | 54 | 0 | 1069 |
| 1443 | 294 | 3.846154 | 54 | 0 | 1069 |
| 1443 | 420 | 6.653465 | 54 | 0 | 1069 |
| 1443 | 469 | 12.37113 | 54 | 0 | 1069 |
| 1443 | 553 | 11.76471 | 54 | 0 | 1069 |
| 1444 | 0 | 6.315789 | 89 | 0 | 472 |
| 1444 | 28 | 12.12121 | 89 | 0 | 472 |
| 1444 | 84 | 13.91304 | 89 | 0 | 472 |

| | | | | | |
|---|---|---|---|---|---|
| 1444 | 168 | 12 | 89 | 0 | 472 |
| 1444 | 266 | 11.88119 | 89 | 0 | 472 |
| 1444 | 350 | 13.04348 | 89 | 0 | 472 |
| 1444 | 446 | 15.25424 | 89 | 0 | 472 |
| 1445 | 0 | 10.28466 | 77 | 1 | 211 |
| 1445 | 33 | 22.59077 | 77 | 1 | 211 |
| 1445 | 84 | 18.36735 | 77 | 1 | 211 |
| 1445 | 167 | 20.97378 | 77 | 1 | 211 |
| 1446 | 0 | 1.517067 | 74 | 0 | 349 |
| 1446 | 28 | 1.558442 | 74 | 0 | 349 |
| 1446 | 63 | 4.444444 | 74 | 0 | 349 |
| 1446 | 133 | 4 | 74 | 0 | 349 |
| 1446 | 252 | 3.883495 | 74 | 0 | 349 |
| 1446 | 294 | 4 | 74 | 0 | 349 |
| 1447 | 0 | 0.897436 | 89 | 0 | 437 |
| 1447 | 17 | 0.847971 | 89 | 0 | 437 |
| 1447 | 80 | 10.81081 | 89 | 0 | 437 |
| 1448 | 0 | 5.882353 | 74 | 0 | 15 |
| 1449 | 0 | 2.285714 | 79 | 0 | 664 |
| 1449 | 28 | 1.30719 | 79 | 0 | 664 |
| 1449 | 91 | 3.262136 | 79 | 0 | 664 |
| 1449 | 151 | 6.060606 | 79 | 0 | 664 |
| 1449 | 242 | 6 | 79 | 0 | 664 |
| 1449 | 333 | 3.703704 | 79 | 0 | 664 |
| 1449 | 375 | 5.714286 | 79 | 0 | 664 |
| 1449 | 487 | 5.555556 | 79 | 0 | 664 |
| 1449 | 578 | 4.705882 | 79 | 0 | 664 |
| 1450 | 0 | 3.669725 | 81 | 0 | 1341 |
| 1450 | 21 | 2.777778 | 81 | 0 | 1341 |
| 1450 | 84 | 1.626016 | 81 | 0 | 1341 |
| 1450 | 168 | 1.025641 | 81 | 0 | 1341 |
| 1450 | 238 | 1.052632 | 81 | 0 | 1341 |
| 1450 | 343 | 1.111111 | 81 | 0 | 1341 |
| 1450 | 413 | 1.428571 | 81 | 0 | 1341 |
| 1450 | 532 | 1.234568 | 81 | 0 | 1341 |
| 1450 | 581 | 1.440576 | 81 | 0 | 1341 |
| 1451 | 0 | 3.361345 | 79 | 1 | 1044 |
| 1451 | 28 | 1.526718 | 79 | 1 | 1044 |
| 1451 | 84 | 1.639344 | 79 | 1 | 1044 |

| | | | | | |
|---|---|---|---|---|---|
| 1452 | 0 | 4.40367 | 78 | 1 | 275 |
| 1452 | 34 | 4.571429 | 78 | 1 | 275 |
| 1452 | 69 | 5.955335 | 78 | 1 | 275 |
| 1452 | 181 | 6.896552 | 78 | 1 | 275 |
| 1452 | 252 | 19.83471 | 78 | 1 | 275 |
| 1453 | 0 | 5.405405 | 72 | 0 | 1233 |
| 1453 | 28 | 5.504587 | 72 | 0 | 1233 |
| 1453 | 91 | 10 | 72 | 0 | 1233 |
| 1453 | 126 | 9.50495 | 72 | 0 | 1233 |
| 1453 | 210 | 12 | 72 | 0 | 1233 |
| 1453 | 301 | 12 | 72 | 0 | 1233 |
| 1453 | 392 | 11.76471 | 72 | 0 | 1233 |
| 1453 | 483 | 9.320388 | 72 | 0 | 1233 |
| 1453 | 609 | 18.18182 | 72 | 0 | 1233 |
| 1454 | 0 | 1.851852 | 67 | 0 | 1093 |
| 1454 | 28 | 1.851852 | 67 | 0 | 1093 |
| 1454 | 84 | 1.754386 | 67 | 0 | 1093 |
| 1454 | 168 | 5.882353 | 67 | 0 | 1093 |
| 1454 | 252 | 1.5 | 67 | 0 | 1093 |
| 1454 | 322 | 1.495327 | 67 | 0 | 1093 |
| 1454 | 427 | 4.285714 | 67 | 0 | 1093 |
| 1454 | 497 | 7.619048 | 67 | 0 | 1093 |
| 1454 | 581 | 3.361345 | 67 | 0 | 1093 |
| 1455 | 0 | 11.42857 | 57 | 1 | 180 |
| 1455 | 28 | 2.34375 | 57 | 1 | 180 |
| 1455 | 84 | 5.769231 | 57 | 1 | 180 |
| 1455 | 168 | 10.50657 | 57 | 1 | 180 |
| 1456 | 0 | 1.984877 | 75 | 0 | 190 |
| 1456 | 30 | 28.23529 | 75 | 0 | 190 |
| 1456 | 154 | 6.315789 | 75 | 0 | 190 |
| 1457 | 14 | 1.190476 | 73 | 0 | 876 |
| 1457 | 42 | 2.631579 | 73 | 0 | 876 |
| 1457 | 161 | 2.586207 | 73 | 0 | 876 |
| 1457 | 252 | 2.608696 | 73 | 0 | 876 |
| 1457 | 308 | 2.753196 | 73 | 0 | 876 |
| 1457 | 392 | 2.678571 | 73 | 0 | 876 |
| 1457 | 476 | 2.727273 | 73 | 0 | 876 |
| 1457 | 567 | 2.307692 | 73 | 0 | 876 |
| 1458 | 0 | 1.980198 | 60 | 1 | 430 |

| | | | | | |
|---|---|---|---|---|---|
| 1458 | 28 | 1.941748 | 60 | 1 | 430 |
| 1458 | 84 | 1.886792 | 60 | 1 | 430 |
| 1458 | 140 | 1.869159 | 60 | 1 | 430 |
| 1458 | 224 | 4.063861 | 60 | 1 | 430 |
| 1458 | 350 | 7.272727 | 60 | 1 | 430 |
| 1458 | 427 | 7.76699 | 60 | 1 | 430 |
| 1459 | 0 | 6.382979 | 77 | 0 | 991 |
| 1459 | 28 | 3.883495 | 77 | 0 | 991 |
| 1459 | 84 | 5.882353 | 77 | 0 | 991 |
| 1459 | 133 | 4.705882 | 77 | 0 | 991 |
| 1459 | 259 | 1.769912 | 77 | 0 | 991 |
| 1459 | 336 | 4.848485 | 77 | 0 | 991 |
| 1459 | 441 | 4.571429 | 77 | 0 | 991 |
| 1459 | 518 | 4.571429 | 77 | 0 | 991 |
| 1459 | 595 | 3.669725 | 77 | 0 | 991 |
| 1460 | 0 | 5.825243 | 69 | 1 | 140 |
| 1460 | 28 | 8 | 69 | 1 | 140 |
| 1460 | 91 | 25 | 69 | 1 | 140 |
| 1461 | 0 | 6.722689 | 82 | 0 | 241 |
| 1461 | 49 | 5.347764 | 82 | 0 | 241 |
| 1461 | 175 | 22.22222 | 82 | 0 | 241 |
| 1461 | 224 | 8.347826 | 82 | 0 | 241 |
| 1462 | 0 | 5.084746 | 77 | 0 | 476 |
| 1462 | 28 | 2.586207 | 77 | 0 | 476 |
| 1462 | 84 | 2.678571 | 77 | 0 | 476 |
| 1462 | 149 | 2.884615 | 77 | 0 | 476 |
| 1462 | 238 | 5.16129 | 77 | 0 | 476 |
| 1462 | 336 | 2.678571 | 77 | 0 | 476 |
| 1462 | 420 | 2.721088 | 77 | 0 | 476 |
| 1462 | 462 | 10.38961 | 77 | 0 | 476 |
| 1463 | 0 | 3 | 70 | 0 | 93 |
| 1463 | 28 | 2.884615 | 70 | 0 | 93 |
| 1464 | 0 | 1.517067 | 65 | 0 | 666 |
| 1464 | 77 | 2.542373 | 65 | 0 | 666 |
| 1464 | 161 | 0.884956 | 65 | 0 | 666 |
| 1464 | 245 | 1.834862 | 65 | 0 | 666 |
| 1464 | 329 | 1.73913 | 65 | 0 | 666 |
| 1464 | 371 | 1.344538 | 65 | 0 | 666 |
| 1464 | 483 | 1.801802 | 65 | 0 | 666 |

| | | | | | |
|---|---|---|---|---|---|
| 1464 | 567 | 1.801802 | 65 | 0 | 666 |
| 1465 | 0 | 2.378929 | 66 | 1 | 760 |
| 1465 | 70 | 2.4 | 66 | 1 | 760 |
| 1465 | 133 | 2.264151 | 66 | 1 | 760 |
| 1465 | 224 | 1.666667 | 66 | 1 | 760 |
| 1465 | 301 | 3.030303 | 66 | 1 | 760 |
| 1465 | 427 | 3.636364 | 66 | 1 | 760 |
| 1465 | 511 | 5.504587 | 66 | 1 | 760 |
| 1465 | 595 | 5.454545 | 66 | 1 | 760 |
| 1466 | 0 | 9.278351 | 79 | 0 | 786 |
| 1466 | 27 | 8.910891 | 79 | 0 | 786 |
| 1466 | 84 | 10.16949 | 79 | 0 | 786 |
| 1466 | 168 | 12.76596 | 79 | 0 | 786 |
| 1466 | 238 | 17.30769 | 79 | 0 | 786 |
| 1466 | 322 | 6.61157 | 79 | 0 | 786 |
| 1466 | 414 | 9.813084 | 79 | 0 | 786 |
| 1466 | 476 | 12.12121 | 79 | 0 | 786 |
| 1466 | 581 | 29.65578 | 79 | 0 | 786 |
| 1467 | 0 | 2.352941 | 74 | 0 | 575 |
| 1467 | 70 | 2.307692 | 74 | 0 | 575 |
| 1467 | 140 | 4.897959 | 74 | 0 | 575 |
| 1467 | 210 | 4.285714 | 74 | 0 | 575 |
| 1467 | 307 | 4.320988 | 74 | 0 | 575 |
| 1467 | 413 | 4.444444 | 74 | 0 | 575 |
| 1467 | 504 | 5.769231 | 74 | 0 | 575 |
| 1467 | 574 | 4.660194 | 74 | 0 | 575 |
| 1468 | 0 | 5.940594 | 77 | 1 | 428 |
| 1468 | 28 | 4.485981 | 77 | 1 | 428 |
| 1468 | 63 | 2.941176 | 77 | 1 | 428 |
| 1468 | 161 | 4.040404 | 77 | 1 | 428 |
| 1468 | 231 | 10.66667 | 77 | 1 | 428 |
| 1468 | 301 | 12 | 77 | 1 | 428 |
| 1468 | 385 | 11.65049 | 77 | 1 | 428 |
| 1469 | 0 | 2.201835 | 71 | 0 | 884 |
| 1469 | 28 | 1.212121 | 71 | 0 | 884 |
| 1469 | 91 | 1.179941 | 71 | 0 | 884 |
| 1469 | 161 | 1.923077 | 71 | 0 | 884 |
| 1469 | 245 | 1.796791 | 71 | 0 | 884 |
| 1469 | 330 | 1.834862 | 71 | 0 | 884 |

| | | | | | |
|---|---|---|---|---|---|
| 1469 | 414 | 1.481481 | 71 | 0 | 884 |
| 1469 | 519 | 1.441441 | 71 | 0 | 884 |
| 1469 | 554 | 5.45809 | 71 | 0 | 884 |
| 1470 | 0 | 5.172414 | 62 | 0 | 995 |
| 1470 | 90 | 5.263158 | 62 | 0 | 995 |
| 1470 | 160 | 4.285714 | 62 | 0 | 995 |
| 1470 | 342 | 10.52632 | 62 | 0 | 995 |
| 1470 | 398 | 6.956522 | 62 | 0 | 995 |
| 1470 | 496 | 60.28708 | 62 | 0 | 995 |
| 1470 | 608 | 36.73469 | 62 | 0 | 995 |
| 1471 | 0 | 9.183673 | 82 | 0 | 477 |
| 1471 | 28 | 3.333333 | 82 | 0 | 477 |
| 1471 | 91 | 3.773585 | 82 | 0 | 477 |
| 1471 | 154 | 9.756098 | 82 | 0 | 477 |
| 1471 | 217 | 17.56098 | 82 | 0 | 477 |
| 1471 | 336 | 6.923077 | 82 | 0 | 477 |
| 1471 | 392 | 13.84615 | 82 | 0 | 477 |
| 1472 | 28 | 0.386847 | 69 | 0 | 1223 |
| 1472 | 84 | 0.407056 | 69 | 0 | 1223 |
| 1472 | 154 | 0.204082 | 69 | 0 | 1223 |
| 1472 | 252 | 0.186945 | 69 | 0 | 1223 |
| 1472 | 336 | 0.180072 | 69 | 0 | 1223 |
| 1472 | 420 | 0.186945 | 69 | 0 | 1223 |
| 1472 | 497 | 0.674916 | 69 | 0 | 1223 |
| 1472 | 595 | 0.726392 | 69 | 0 | 1223 |
| 1473 | 0 | 5 | 73 | 0 | 729 |
| 1473 | 84 | 10.71429 | 73 | 0 | 729 |
| 1473 | 168 | 4.693611 | 73 | 0 | 729 |
| 1473 | 259 | 7.894737 | 73 | 0 | 729 |
| 1473 | 315 | 7.826087 | 73 | 0 | 729 |
| 1473 | 406 | 3.664122 | 73 | 0 | 729 |
| 1473 | 511 | 10.61947 | 73 | 0 | 729 |
| 1473 | 553 | 10.61947 | 73 | 0 | 729 |
| 1474 | 0 | 8.807339 | 68 | 1 | 403 |
| 1474 | 70 | 13.21101 | 68 | 1 | 403 |
| 1474 | 147 | 10.52632 | 68 | 1 | 403 |
| 1474 | 259 | 15.38462 | 68 | 1 | 403 |
| 1474 | 294 | 5.042017 | 68 | 1 | 403 |
| 1474 | 392 | 9.89011 | 68 | 1 | 403 |

| | | | | | |
|---|---|---|---|---|---|
| 1475 | 0 | 2.016807 | 77 | 0 | 932 |
| 1475 | 91 | 2.5 | 77 | 0 | 932 |
| 1475 | 147 | 2.521008 | 77 | 0 | 932 |
| 1475 | 245 | 1.967213 | 77 | 0 | 932 |
| 1476 | 0 | 4.137931 | 75 | 0 | 1083 |
| 1476 | 35 | 4.491979 | 75 | 0 | 1083 |
| 1476 | 104 | 4.324324 | 75 | 0 | 1083 |
| 1476 | 139 | 4.363636 | 75 | 0 | 1083 |
| 1476 | 230 | 3.508772 | 75 | 0 | 1083 |
| 1476 | 307 | 3.478261 | 75 | 0 | 1083 |
| 1476 | 391 | 3.539823 | 75 | 0 | 1083 |
| 1476 | 468 | 3.274854 | 75 | 0 | 1083 |
| 1476 | 555 | 3.363363 | 75 | 0 | 1083 |
| 1477 | 0 | 6.451613 | 68 | 0 | 995 |
| 1477 | 28 | 6.896552 | 68 | 0 | 995 |
| 1477 | 84 | 7.207207 | 68 | 0 | 995 |
| 1477 | 168 | 3.738318 | 68 | 0 | 995 |
| 1477 | 249 | 6.122449 | 68 | 0 | 995 |
| 1477 | 336 | 3.773585 | 68 | 0 | 995 |
| 1477 | 378 | 5.555556 | 68 | 0 | 995 |
| 1477 | 469 | 5.454545 | 68 | 0 | 995 |
| 1477 | 595 | 3.703704 | 68 | 0 | 995 |
| 1478 | 0 | 2.542373 | 70 | 1 | 710 |
| 1478 | 28 | 2.086957 | 70 | 1 | 710 |
| 1478 | 91 | 2.21914 | 70 | 1 | 710 |
| 1478 | 168 | 8.421053 | 70 | 1 | 710 |
| 1478 | 252 | 11.11111 | 70 | 1 | 710 |
| 1478 | 336 | 9.795918 | 70 | 1 | 710 |
| 1478 | 420 | 11.21495 | 70 | 1 | 710 |
| 1478 | 504 | 11.88119 | 70 | 1 | 710 |
| 1478 | 588 | 13.48315 | 70 | 1 | 710 |
| 1479 | 0 | 2.083333 | 69 | 1 | 315 |
| 1479 | 70 | 2.040816 | 69 | 1 | 315 |
| 1479 | 154 | 1.684211 | 69 | 1 | 315 |
| 1479 | 236 | 2.898551 | 69 | 1 | 315 |
| 1479 | 299 | 14.63415 | 69 | 1 | 315 |
| 1480 | 0 | 4.615385 | 57 | 0 | 1072 |
| 1480 | 28 | 5.660377 | 57 | 0 | 1072 |
| 1480 | 56 | 7.346939 | 57 | 0 | 1072 |

| | | | | | |
|---|---|---|---|---|---|
| 1480 | 126 | 11.42857 | 57 | 0 | 1072 |
| 1480 | 245 | 19.14894 | 57 | 0 | 1072 |
| 1480 | 301 | 8.648649 | 57 | 0 | 1072 |
| 1480 | 406 | 6.122449 | 57 | 0 | 1072 |
| 1480 | 511 | 7.373272 | 57 | 0 | 1072 |
| 1480 | 567 | 9.278351 | 57 | 0 | 1072 |
| 1481 | 0 | 2.702703 | 59 | 0 | 1235 |
| 1481 | 28 | 1.490683 | 59 | 0 | 1235 |
| 1481 | 77 | 2.884615 | 59 | 0 | 1235 |
| 1481 | 182 | 2.564103 | 59 | 0 | 1235 |
| 1481 | 238 | 3.174603 | 59 | 0 | 1235 |
| 1481 | 329 | 2.586207 | 59 | 0 | 1235 |
| 1481 | 385 | 3.703704 | 59 | 0 | 1235 |
| 1481 | 469 | 1.948052 | 59 | 0 | 1235 |
| 1481 | 602 | 3 | 59 | 0 | 1235 |
| 1482 | 0 | 5.309735 | 57 | 0 | 610 |
| 1482 | 28 | 5.607477 | 57 | 0 | 610 |
| 1482 | 84 | 5.555556 | 57 | 0 | 610 |
| 1482 | 175 | 5.405405 | 57 | 0 | 610 |
| 1482 | 273 | 5.263158 | 57 | 0 | 610 |
| 1482 | 336 | 8.275862 | 57 | 0 | 610 |
| 1482 | 371 | 4.173913 | 57 | 0 | 610 |
| 1482 | 504 | 5.309735 | 57 | 0 | 610 |
| 1482 | 574 | 4.444444 | 57 | 0 | 610 |
| 1483 | 0 | 2.764977 | 74 | 0 | 597 |
| 1483 | 49 | 0.464576 | 74 | 0 | 597 |
| 1484 | 0 | 1.526718 | 71 | 0 | 1163 |
| 1484 | 28 | 1.145038 | 71 | 0 | 1163 |
| 1484 | 140 | 1.219512 | 71 | 0 | 1163 |
| 1484 | 259 | 0.42343 | 71 | 0 | 1163 |
| 1484 | 315 | 1.304348 | 71 | 0 | 1163 |
| 1485 | 0 | 21.68675 | 95 | 0 | 317 |
| 1485 | 28 | 17.67181 | 95 | 0 | 317 |
| 1485 | 84 | 18.94737 | 95 | 0 | 317 |
| 1485 | 168 | 21.17647 | 95 | 0 | 317 |
| 1485 | 224 | 13.02326 | 95 | 0 | 317 |
| 1485 | 301 | 20.51282 | 95 | 0 | 317 |
| 1486 | 0 | 2.702703 | 76 | 0 | 873 |
| 1486 | 28 | 6.382979 | 76 | 0 | 873 |

| | | | | | |
|---|---|---|---|---|---|
| 1486 | 84 | 2.402402 | 76 | 0 | 873 |
| 1486 | 154 | 4.485981 | 76 | 0 | 873 |
| 1486 | 252 | 6.060606 | 76 | 0 | 873 |
| 1486 | 343 | 2.749141 | 76 | 0 | 873 |
| 1486 | 511 | 5.769231 | 76 | 0 | 873 |
| 1486 | 602 | 5.607477 | 76 | 0 | 873 |
| 1487 | 0 | 4.324324 | 55 | 1 | 854 |
| 1487 | 35 | 7.476636 | 55 | 1 | 854 |
| 1487 | 77 | 7.272727 | 55 | 1 | 854 |
| 1487 | 188 | 3.145478 | 55 | 1 | 854 |
| 1487 | 266 | 3.361345 | 55 | 1 | 854 |
| 1487 | 319 | 3.846154 | 55 | 1 | 854 |
| 1487 | 413 | 4.528302 | 55 | 1 | 854 |
| 1487 | 504 | 3.53461 | 55 | 1 | 854 |
| 1487 | 553 | 3.428571 | 55 | 1 | 854 |
| 1488 | 0 | 4.8 | 78 | 0 | 720 |
| 1488 | 28 | 4.8 | 78 | 0 | 720 |
| 1488 | 89 | 5.128205 | 78 | 0 | 720 |
| 1488 | 187 | 4.137931 | 78 | 0 | 720 |
| 1488 | 257 | 5.217391 | 78 | 0 | 720 |
| 1488 | 313 | 4.918033 | 78 | 0 | 720 |
| 1488 | 425 | 4.724409 | 78 | 0 | 720 |
| 1488 | 481 | 4.724409 | 78 | 0 | 720 |
| 1488 | 593 | 4.83871 | 78 | 0 | 720 |
| 1489 | 0 | 4.123711 | 83 | 0 | 423 |
| 1489 | 28 | 5.607477 | 83 | 0 | 423 |
| 1489 | 112 | 5.357143 | 83 | 0 | 423 |
| 1489 | 175 | 3.883495 | 83 | 0 | 423 |
| 1489 | 277 | 4.392157 | 83 | 0 | 423 |
| 1489 | 359 | 3.296703 | 83 | 0 | 423 |
| 1489 | 422 | 3.047619 | 83 | 0 | 423 |
| 1490 | 0 | 2.295082 | 80 | 0 | 645 |
| 1490 | 91 | 2.276423 | 80 | 0 | 645 |
| 1490 | 148 | 0.359697 | 80 | 0 | 645 |
| 1490 | 217 | 0.365742 | 80 | 0 | 645 |
| 1490 | 312 | 2.586207 | 80 | 0 | 645 |
| 1490 | 398 | 2.011494 | 80 | 0 | 645 |
| 1490 | 523 | 2.175602 | 80 | 0 | 645 |
| 1490 | 602 | 1.517067 | 80 | 0 | 645 |

| | | | | | |
|---|---|---|---|---|---|
| 1491 | 0 | 1.95122 | 68 | 1 | 902 |
| 1491 | 35 | 1.075269 | 68 | 1 | 902 |
| 1491 | 98 | 1.322314 | 68 | 1 | 902 |
| 1491 | 175 | 1.851852 | 68 | 1 | 902 |
| 1491 | 259 | 4 | 68 | 1 | 902 |
| 1491 | 343 | 5.660377 | 68 | 1 | 902 |
| 1491 | 427 | 6.818182 | 68 | 1 | 902 |
| 1491 | 511 | 12.12121 | 68 | 1 | 902 |
| 1491 | 595 | 11.42857 | 68 | 1 | 902 |
| 1492 | 0 | 2.830189 | 80 | 1 | 212 |
| 1492 | 28 | 2.752294 | 80 | 1 | 212 |
| 1492 | 84 | 2.970297 | 80 | 1 | 212 |
| 1492 | 168 | 3.773585 | 80 | 1 | 212 |
| 1493 | 0 | 6.593407 | 63 | 0 | 1144 |
| 1493 | 28 | 2.721088 | 63 | 0 | 1144 |
| 1493 | 91 | 4.545455 | 63 | 0 | 1144 |
| 1493 | 154 | 3.137255 | 63 | 0 | 1144 |
| 1494 | 0 | 4.660194 | 79 | 0 | 743 |
| 1494 | 35 | 0.611621 | 79 | 0 | 743 |
| 1494 | 119 | 0.606061 | 79 | 0 | 743 |
| 1494 | 203 | 0.623053 | 79 | 0 | 743 |
| 1494 | 287 | 0.634921 | 79 | 0 | 743 |
| 1494 | 350 | 2.564103 | 79 | 0 | 743 |
| 1494 | 420 | 5 | 79 | 0 | 743 |
| 1495 | 0 | 4.918033 | 75 | 0 | 170 |
| 1495 | 28 | 5.172414 | 75 | 0 | 170 |
| 1495 | 84 | 6.060606 | 75 | 0 | 170 |
| 1496 | 0 | 5.16129 | 36 | 1 | 170 |
| 1496 | 35 | 4.897959 | 36 | 1 | 170 |
| 1496 | 85 | 6.593407 | 36 | 1 | 170 |
| 1496 | 168 | 18.30065 | 36 | 1 | 170 |
| 1497 | 0 | 2.307692 | 63 | 0 | 714 |
| 1497 | 21 | 2.857143 | 63 | 0 | 714 |
| 1497 | 84 | 17.97753 | 63 | 0 | 714 |
| 1497 | 168 | 10.71429 | 63 | 0 | 714 |
| 1497 | 224 | 10.43478 | 63 | 0 | 714 |
| 1497 | 336 | 3.478261 | 63 | 0 | 714 |
| 1497 | 420 | 11.53846 | 63 | 0 | 714 |
| 1497 | 497 | 5.593785 | 63 | 0 | 714 |

| | | | | | |
|---|---|---|---|---|---|
| 1497 | 582 | 5.798435 | 63 | 0 | 714 |
| 1498 | 0 | 2.731707 | 80 | 0 | 915 |
| 1498 | 18 | 1.083871 | 80 | 0 | 915 |
| 1498 | 77 | 1.271186 | 80 | 0 | 915 |
| 1498 | 165 | 0.165936 | 80 | 0 | 915 |
| 1498 | 252 | 0.165936 | 80 | 0 | 915 |
| 1498 | 336 | 1.801802 | 80 | 0 | 915 |
| 1498 | 420 | 1.801802 | 80 | 0 | 915 |
| 1498 | 511 | 3.225806 | 80 | 0 | 915 |
| 1498 | 567 | 4.660194 | 80 | 0 | 915 |
| 1499 | 0 | 10.16949 | 75 | 0 | 1163 |
| 1499 | 42 | 9.917355 | 75 | 0 | 1163 |
| 1499 | 84 | 12.97297 | 75 | 0 | 1163 |
| 1499 | 189 | 12.85714 | 75 | 0 | 1163 |
| 1499 | 259 | 7.826087 | 75 | 0 | 1163 |
| 1499 | 371 | 8.333333 | 75 | 0 | 1163 |
| 1499 | 427 | 7.563025 | 75 | 0 | 1163 |
| 1499 | 532 | 9.52381 | 75 | 0 | 1163 |
| 1499 | 588 | 15.65217 | 75 | 0 | 1163 |
| 1500 | 0 | 7.407407 | 84 | 0 | 1142 |
| 1500 | 21 | 5.769231 | 84 | 0 | 1142 |
| 1500 | 77 | 4.324324 | 84 | 0 | 1142 |
| 1500 | 168 | 5.405405 | 84 | 0 | 1142 |
| 1500 | 252 | 4.173913 | 84 | 0 | 1142 |
| 1500 | 329 | 3.333333 | 84 | 0 | 1142 |
| 1500 | 413 | 2.5 | 84 | 0 | 1142 |
| 1500 | 497 | 0.793651 | 84 | 0 | 1142 |
| 1500 | 553 | 1.327434 | 84 | 0 | 1142 |
| 1501 | 0 | 2.201835 | 77 | 0 | 649 |
| 1501 | 35 | 2.777778 | 77 | 0 | 649 |
| 1501 | 74 | 2.419355 | 77 | 0 | 649 |
| 1501 | 137 | 0.990099 | 77 | 0 | 649 |
| 1501 | 277 | 2.474227 | 77 | 0 | 649 |
| 1501 | 341 | 2.474227 | 77 | 0 | 649 |
| 1501 | 403 | 4.897959 | 77 | 0 | 649 |
| 1501 | 543 | 5.874126 | 77 | 0 | 649 |
| 1501 | 578 | 4.033613 | 77 | 0 | 649 |
| 1502 | 0 | 3.018868 | 47 | 1 | 1097 |
| 1502 | 35 | 2.614379 | 47 | 1 | 1097 |

| | | | | | |
|---|---|---|---|---|---|
| 1502 | 126 | 2.263083 | 47 | 1 | 1097 |
| 1502 | 216 | 2.21914 | 47 | 1 | 1097 |
| 1502 | 328 | 1.932367 | 47 | 1 | 1097 |
| 1502 | 391 | 1.760176 | 47 | 1 | 1097 |
| 1503 | 0 | 2.439024 | 55 | 1 | 567 |
| 1503 | 77 | 0.914286 | 55 | 1 | 567 |
| 1503 | 161 | 1.344538 | 55 | 1 | 567 |
| 1503 | 259 | 1.311475 | 55 | 1 | 567 |
| 1503 | 343 | 1.709402 | 55 | 1 | 567 |
| 1503 | 413 | 2.086957 | 55 | 1 | 567 |
| 1503 | 518 | 2.180685 | 55 | 1 | 567 |
| 1504 | 0 | 2.167183 | 51 | 0 | 758 |
| 1504 | 34 | 1.652893 | 51 | 0 | 758 |
| 1504 | 62 | 1.414141 | 51 | 0 | 758 |
| 1504 | 181 | 1.885522 | 51 | 0 | 758 |
| 1504 | 240 | 2.424242 | 51 | 0 | 758 |
| 1504 | 338 | 2.777778 | 51 | 0 | 758 |
| 1504 | 427 | 2.44898 | 51 | 0 | 758 |
| 1504 | 499 | 12.76596 | 51 | 0 | 758 |
| 1505 | 0 | 0.793651 | 72 | 0 | 874 |
| 1505 | 21 | 0.763359 | 72 | 0 | 874 |
| 1505 | 84 | 1.052632 | 72 | 0 | 874 |
| 1505 | 168 | 0.840336 | 72 | 0 | 874 |
| 1505 | 252 | 0.900901 | 72 | 0 | 874 |
| 1505 | 336 | 1.149425 | 72 | 0 | 874 |
| 1506 | 0 | 2.245389 | 50 | 1 | 345 |
| 1506 | 29 | 1.196581 | 50 | 1 | 345 |
| 1506 | 84 | 0.386207 | 50 | 1 | 345 |
| 1506 | 171 | 1.994302 | 50 | 1 | 345 |
| 1506 | 245 | 1.271186 | 50 | 1 | 345 |
| 1506 | 336 | 10 | 50 | 1 | 345 |
| 1507 | 0 | 2.830189 | 63 | 1 | 226 |
| 1507 | 56 | 0.943396 | 63 | 1 | 226 |
| 1507 | 140 | 2.44898 | 63 | 1 | 226 |
| 1507 | 203 | 3.488372 | 63 | 1 | 226 |
| 1508 | 0 | 10.90909 | 74 | 0 | 1079 |
| 1508 | 28 | 7.756233 | 74 | 0 | 1079 |
| 1508 | 84 | 14.11765 | 74 | 0 | 1079 |
| 1508 | 168 | 18 | 74 | 0 | 1079 |

| | | | | | |
|---|---|---|---|---|---|
| 1508 | 252 | 17.47573 | 74 | 0 | 1079 |
| 1508 | 336 | 17.82178 | 74 | 0 | 1079 |
| 1508 | 420 | 16.49485 | 74 | 0 | 1079 |
| 1508 | 511 | 17.47573 | 74 | 0 | 1079 |
| 1508 | 616 | 24.74227 | 74 | 0 | 1079 |
| 1509 | 0 | 0.47348 | 79 | 0 | 652 |
| 1509 | 98 | 2.068966 | 79 | 0 | 652 |
| 1509 | 168 | 2.033898 | 79 | 0 | 652 |
| 1509 | 273 | 2.051282 | 79 | 0 | 652 |
| 1509 | 343 | 2.201835 | 79 | 0 | 652 |
| 1509 | 441 | 2.678571 | 79 | 0 | 652 |
| 1509 | 504 | 2.330097 | 79 | 0 | 652 |
| 1509 | 595 | 2.912621 | 79 | 0 | 652 |
| 1510 | 0 | 1.388889 | 67 | 0 | 1234 |
| 1510 | 28 | 5.555556 | 67 | 0 | 1234 |
| 1510 | 105 | 6.542056 | 67 | 0 | 1234 |
| 1510 | 182 | 5.309735 | 67 | 0 | 1234 |
| 1510 | 245 | 5.405405 | 67 | 0 | 1234 |
| 1510 | 329 | 2.5 | 67 | 0 | 1234 |
| 1510 | 421 | 3.669725 | 67 | 0 | 1234 |
| 1510 | 498 | 2.802803 | 67 | 0 | 1234 |
| 1510 | 583 | 1.392573 | 67 | 0 | 1234 |
| 1511 | 0 | 0.263635 | 74 | 0 | 778 |
| 1511 | 91 | 0.293201 | 74 | 0 | 778 |
| 1511 | 147 | 0.268141 | 74 | 0 | 778 |
| 1511 | 280 | 0.313725 | 74 | 0 | 778 |
| 1511 | 322 | 1.367521 | 74 | 0 | 778 |
| 1512 | 0 | 13.48315 | 76 | 0 | 722 |
| 1512 | 28 | 24.65753 | 76 | 0 | 722 |
| 1512 | 84 | 17.77778 | 76 | 0 | 722 |
| 1512 | 182 | 18.46154 | 76 | 0 | 722 |
| 1512 | 273 | 16.98113 | 76 | 0 | 722 |
| 1512 | 329 | 32.72727 | 76 | 0 | 722 |
| 1513 | 20 | 1.614453 | 60 | 0 | 715 |
| 1513 | 63 | 1.269841 | 60 | 0 | 715 |
| 1513 | 168 | 2.033898 | 60 | 0 | 715 |
| 1513 | 252 | 2.479339 | 60 | 0 | 715 |
| 1513 | 308 | 2.419355 | 60 | 0 | 715 |
| 1513 | 420 | 1.393728 | 60 | 0 | 715 |

| | | | | | |
|---|---|---|---|---|---|
| 1513 | 504 | 2 | 60 | 0 | 715 |
| 1513 | 581 | 5.6 | 60 | 0 | 715 |
| 1514 | 0 | 2.803738 | 73 | 0 | 1058 |
| 1514 | 28 | 1.327434 | 73 | 0 | 1058 |
| 1514 | 84 | 4.204204 | 73 | 0 | 1058 |
| 1514 | 140 | 3.145478 | 73 | 0 | 1058 |
| 1514 | 231 | 4.40367 | 73 | 0 | 1058 |
| 1514 | 301 | 4.285714 | 73 | 0 | 1058 |
| 1514 | 399 | 2.777778 | 73 | 0 | 1058 |
| 1514 | 483 | 2.181818 | 73 | 0 | 1058 |
| 1514 | 574 | 2.857143 | 73 | 0 | 1058 |
| 1515 | 0 | 1.869159 | 24 | 1 | 602 |
| 1515 | 42 | 2.912621 | 24 | 1 | 602 |
| 1515 | 95 | 4.040404 | 24 | 1 | 602 |
| 1515 | 182 | 3.174603 | 24 | 1 | 602 |
| 1515 | 266 | 3.448276 | 24 | 1 | 602 |
| 1515 | 351 | 7.761608 | 24 | 1 | 602 |
| 1515 | 420 | 1.851852 | 24 | 1 | 602 |
| 1515 | 504 | 3.669725 | 24 | 1 | 602 |
| 1516 | 0 | 0.766284 | 81 | 0 | 1021 |
| 1516 | 63 | 0.444444 | 81 | 0 | 1021 |
| 1516 | 126 | 0.42735 | 81 | 0 | 1021 |
| 1516 | 252 | 0.815494 | 81 | 0 | 1021 |
| 1516 | 315 | 0.57971 | 81 | 0 | 1021 |
| 1516 | 378 | 0.606061 | 81 | 0 | 1021 |
| 1516 | 511 | 0.824742 | 81 | 0 | 1021 |
| 1516 | 581 | 2.352941 | 81 | 0 | 1021 |
| 1517 | 0 | 2.884615 | 76 | 0 | 554 |
| 1517 | 28 | 2.524038 | 76 | 0 | 554 |
| 1517 | 88 | 5.042017 | 76 | 0 | 554 |
| 1517 | 172 | 5.128205 | 76 | 0 | 554 |
| 1517 | 256 | 4.878049 | 76 | 0 | 554 |
| 1517 | 340 | 1.73913 | 76 | 0 | 554 |
| 1517 | 424 | 5.27307 | 76 | 0 | 554 |
| 1517 | 483 | 4.067797 | 76 | 0 | 554 |
| 1517 | 553 | 4.210526 | 76 | 0 | 554 |
| 1518 | 0 | 26.37363 | 28 | 0 | 530 |
| 1518 | 85 | 12.08198 | 28 | 0 | 530 |
| 1518 | 169 | 6.122449 | 28 | 0 | 530 |

| | | | | | |
|---|---|---|---|---|---|
| 1518 | 253 | 5.769231 | 28 | 0 | 530 |
| 1518 | 337 | 14.47028 | 28 | 0 | 530 |
| 1518 | 420 | 13.04348 | 28 | 0 | 530 |
| 1518 | 476 | 23.07692 | 28 | 0 | 530 |
| 1519 | 0 | 2.654867 | 79 | 1 | 463 |
| 1519 | 91 | 0.961538 | 79 | 1 | 463 |
| 1519 | 175 | 1.212121 | 79 | 1 | 463 |
| 1519 | 238 | 1.223242 | 79 | 1 | 463 |
| 1520 | 0 | 4.848485 | 83 | 1 | 18 |
| 1521 | 0 | 11.07692 | 85 | 0 | 647 |
| 1521 | 28 | 10.58824 | 85 | 0 | 647 |
| 1521 | 63 | 14.17323 | 85 | 0 | 647 |
| 1521 | 161 | 19.2 | 85 | 0 | 647 |
| 1521 | 238 | 14.17323 | 85 | 0 | 647 |
| 1521 | 326 | 15.78947 | 85 | 0 | 647 |
| 1521 | 413 | 13.43284 | 85 | 0 | 647 |
| 1521 | 490 | 13.04348 | 85 | 0 | 647 |
| 1521 | 553 | 27.48092 | 85 | 0 | 647 |
| 1522 | 0 | 1.320132 | 41 | 0 | 649 |
| 1522 | 63 | 5.30303 | 41 | 0 | 649 |
| 1522 | 163 | 4.301075 | 41 | 0 | 649 |
| 1522 | 268 | 2.930403 | 41 | 0 | 649 |
| 1522 | 338 | 9.090909 | 41 | 0 | 649 |
| 1522 | 421 | 6.382979 | 41 | 0 | 649 |
| 1522 | 513 | 6 | 41 | 0 | 649 |
| 1522 | 613 | 6.801619 | 41 | 0 | 649 |
| 1523 | 0 | 4.786325 | 50 | 0 | 455 |
| 1523 | 84 | 7.54717 | 50 | 0 | 455 |
| 1523 | 175 | 1.811907 | 50 | 0 | 455 |
| 1523 | 251 | 4.660194 | 50 | 0 | 455 |
| 1523 | 314 | 4.081633 | 50 | 0 | 455 |
| 1523 | 433 | 5.194805 | 50 | 0 | 455 |
| 1524 | 0 | 4.592674 | 56 | 0 | 1208 |
| 1524 | 31 | 10.90909 | 56 | 0 | 1208 |
| 1524 | 91 | 8.205128 | 56 | 0 | 1208 |
| 1524 | 161 | 10.81081 | 56 | 0 | 1208 |
| 1524 | 252 | 8.495575 | 56 | 0 | 1208 |
| 1524 | 315 | 8.421053 | 56 | 0 | 1208 |
| 1524 | 405 | 11.24874 | 56 | 0 | 1208 |

| | | | | | |
|---|---|---|---|---|---|
| 1524 | 525 | 9.69697 | 56 | 0 | 1208 |
| 1524 | 580 | 11.35903 | 56 | 0 | 1208 |
| 1525 | 0 | 3.883495 | 50 | 1 | 248 |
| 1525 | 42 | 3.809524 | 50 | 1 | 248 |
| 1525 | 84 | 3.669725 | 50 | 1 | 248 |
| 1525 | 168 | 6.990291 | 50 | 1 | 248 |
| 1525 | 224 | 6.191646 | 50 | 1 | 248 |
| 1526 | 0 | 5.228758 | 72 | 0 | 791 |
| 1526 | 27 | 5.217391 | 72 | 0 | 791 |
| 1526 | 83 | 5 | 72 | 0 | 791 |
| 1526 | 167 | 4.958678 | 72 | 0 | 791 |
| 1526 | 251 | 5.309735 | 72 | 0 | 791 |
| 1526 | 335 | 5.607477 | 72 | 0 | 791 |
| 1526 | 419 | 5.217391 | 72 | 0 | 791 |
| 1526 | 510 | 4.285714 | 72 | 0 | 791 |
| 1526 | 601 | 5.660377 | 72 | 0 | 791 |
| 1527 | 0 | 4.918033 | 69 | 1 | 312 |
| 1527 | 28 | 5.084746 | 69 | 1 | 312 |
| 1527 | 94 | 2.539683 | 69 | 1 | 312 |
| 1527 | 154 | 4.571429 | 69 | 1 | 312 |
| 1527 | 245 | 13.46801 | 69 | 1 | 312 |
| 1528 | 0 | 2.765584 | 49 | 0 | 886 |
| 1528 | 24 | 2.573529 | 49 | 0 | 886 |
| 1528 | 94 | 2.941176 | 49 | 0 | 886 |
| 1528 | 164 | 0.960384 | 49 | 0 | 886 |
| 1528 | 234 | 1.085973 | 49 | 0 | 886 |
| 1528 | 339 | 1.832061 | 49 | 0 | 886 |
| 1528 | 416 | 1.694915 | 49 | 0 | 886 |
| 1528 | 514 | 1.420118 | 49 | 0 | 886 |
| 1528 | 556 | 1.476923 | 49 | 0 | 886 |
| 1529 | 0 | 1.15942 | 66 | 0 | 190 |
| 1529 | 42 | 1.201201 | 66 | 0 | 190 |
| 1530 | 0 | 4.807692 | 80 | 0 | 685 |
| 1530 | 26 | 8.196721 | 80 | 0 | 685 |
| 1530 | 82 | 7.575758 | 80 | 0 | 685 |
| 1530 | 166 | 3.72093 | 80 | 0 | 685 |
| 1530 | 257 | 5.405405 | 80 | 0 | 685 |
| 1530 | 341 | 4.285714 | 80 | 0 | 685 |
| 1530 | 411 | 4.485981 | 80 | 0 | 685 |

| | | | | | |
|---|---|---|---|---|---|
| 1530 | 502 | 7.54717 | 80 | 0 | 685 |
| 1530 | 586 | 9.803922 | 80 | 0 | 685 |
| 1531 | 0 | 5.084746 | 89 | 0 | 390 |
| 1531 | 22 | 2.857143 | 89 | 0 | 390 |
| 1531 | 77 | 1.538462 | 89 | 0 | 390 |
| 1531 | 161 | 1.785714 | 89 | 0 | 390 |
| 1531 | 245 | 2.912621 | 89 | 0 | 390 |
| 1531 | 329 | 2.56332 | 89 | 0 | 390 |
| 1532 | 0 | 3.773585 | 80 | 0 | 1198 |
| 1532 | 28 | 2.990654 | 80 | 0 | 1198 |
| 1532 | 91 | 3.571429 | 80 | 0 | 1198 |
| 1532 | 154 | 1.666667 | 80 | 0 | 1198 |
| 1532 | 245 | 1.709402 | 80 | 0 | 1198 |
| 1532 | 336 | 1.851852 | 80 | 0 | 1198 |
| 1532 | 427 | 3.738318 | 80 | 0 | 1198 |
| 1532 | 490 | 3.571429 | 80 | 0 | 1198 |
| 1533 | 0 | 0.892857 | 67 | 0 | 1040 |
| 1533 | 21 | 0.900901 | 67 | 0 | 1040 |
| 1533 | 161 | 0.934579 | 67 | 0 | 1040 |
| 1533 | 245 | 1.923077 | 67 | 0 | 1040 |
| 1533 | 329 | 0.952381 | 67 | 0 | 1040 |
| 1533 | 413 | 0.952381 | 67 | 0 | 1040 |
| 1533 | 518 | 1.360544 | 67 | 0 | 1040 |
| 1533 | 574 | 2.020202 | 67 | 0 | 1040 |
| 1534 | 0 | 2.330097 | 64 | 1 | 541 |
| 1534 | 35 | 2.580645 | 64 | 1 | 541 |
| 1534 | 98 | 0.904977 | 64 | 1 | 541 |
| 1534 | 161 | 3.092784 | 64 | 1 | 541 |
| 1534 | 231 | 3.296703 | 64 | 1 | 541 |
| 1534 | 322 | 2 | 64 | 1 | 541 |
| 1534 | 427 | 3.225806 | 64 | 1 | 541 |
| 1534 | 505 | 4.719101 | 64 | 1 | 541 |
| 1535 | 0 | 5.940594 | 74 | 0 | 1037 |
| 1535 | 28 | 8.823529 | 74 | 0 | 1037 |
| 1535 | 84 | 5.309735 | 74 | 0 | 1037 |
| 1535 | 168 | 11.00917 | 74 | 0 | 1037 |
| 1535 | 252 | 12.79513 | 74 | 0 | 1037 |
| 1535 | 336 | 18.18182 | 74 | 0 | 1037 |
| 1535 | 434 | 11.00917 | 74 | 0 | 1037 |

| | | | | | |
|---|---|---|---|---|---|
| 1535 | 490 | 17.47573 | 74 | 0 | 1037 |
| 1535 | 574 | 17.30769 | 74 | 0 | 1037 |
| 1536 | 0 | 1.818182 | 84 | 0 | 974 |
| 1536 | 28 | 1.818182 | 84 | 0 | 974 |
| 1536 | 84 | 2.727273 | 84 | 0 | 974 |
| 1536 | 175 | 2.242991 | 84 | 0 | 974 |
| 1536 | 238 | 2.586207 | 84 | 0 | 974 |
| 1536 | 336 | 1.851852 | 84 | 0 | 974 |
| 1536 | 413 | 2.307692 | 84 | 0 | 974 |
| 1536 | 476 | 2.222222 | 84 | 0 | 974 |
| 1536 | 588 | 1.617251 | 84 | 0 | 974 |
| 1537 | 0 | 13.04348 | 75 | 0 | 710 |
| 1537 | 23 | 8.727273 | 75 | 0 | 710 |
| 1537 | 86 | 15.51724 | 75 | 0 | 710 |
| 1537 | 170 | 11.14058 | 75 | 0 | 710 |
| 1537 | 247 | 5.384615 | 75 | 0 | 710 |
| 1537 | 331 | 1.666667 | 75 | 0 | 710 |
| 1537 | 422 | 3.809524 | 75 | 0 | 710 |
| 1537 | 492 | 5.263158 | 75 | 0 | 710 |
| 1537 | 576 | 5.263158 | 75 | 0 | 710 |
| 1538 | 0 | 5 | 68 | 0 | 890 |
| 1538 | 28 | 5.263158 | 68 | 0 | 890 |
| 1538 | 84 | 5.454545 | 68 | 0 | 890 |
| 1538 | 168 | 5.217391 | 68 | 0 | 890 |
| 1538 | 252 | 5.128205 | 68 | 0 | 890 |
| 1538 | 343 | 6 | 68 | 0 | 890 |
| 1538 | 427 | 5.714286 | 68 | 0 | 890 |
| 1538 | 518 | 5.405405 | 68 | 0 | 890 |
| 1539 | 0 | 2.061856 | 46 | 0 | 944 |
| 1539 | 105 | 6.666667 | 46 | 0 | 944 |
| 1539 | 168 | 6.521739 | 46 | 0 | 944 |
| 1539 | 224 | 6.593407 | 46 | 0 | 944 |
| 1539 | 343 | 6.451613 | 46 | 0 | 944 |
| 1539 | 420 | 12.16216 | 46 | 0 | 944 |
| 1539 | 504 | 11.53846 | 46 | 0 | 944 |
| 1540 | 0 | 4.278075 | 69 | 0 | 626 |
| 1540 | 28 | 4.8 | 69 | 0 | 626 |
| 1540 | 84 | 5.263158 | 69 | 0 | 626 |
| 1540 | 168 | 5.660377 | 69 | 0 | 626 |

| | | | | | |
|---|---|---|---|---|---|
| 1540 | 252 | 11.88119 | 69 | 0 | 626 |
| 1540 | 335 | 14.51613 | 69 | 0 | 626 |
| 1541 | 0 | 2.479339 | 71 | 1 | 75 |
| 1541 | 28 | 12 | 71 | 1 | 75 |
| 1541 | 42 | 11.65049 | 71 | 1 | 75 |
| 1542 | 0 | 7.5 | 78 | 0 | 818 |
| 1542 | 29 | 7.058824 | 78 | 0 | 818 |
| 1542 | 85 | 6.366048 | 78 | 0 | 818 |
| 1542 | 169 | 3.418803 | 78 | 0 | 818 |
| 1542 | 253 | 3.883495 | 78 | 0 | 818 |
| 1542 | 337 | 6.060606 | 78 | 0 | 818 |
| 1542 | 421 | 12.05165 | 78 | 0 | 818 |
| 1542 | 463 | 16.09195 | 78 | 0 | 818 |
| 1543 | 0 | 3.846154 | 64 | 1 | 185 |
| 1543 | 14 | 5.309735 | 64 | 1 | 185 |
| 1543 | 70 | 5.172414 | 64 | 1 | 185 |
| 1543 | 164 | 11.88119 | 64 | 1 | 185 |
| 1544 | 0 | 2.082128 | 58 | 1 | 449 |
| 1544 | 21 | 2.387268 | 58 | 1 | 449 |
| 1544 | 84 | 2.612482 | 58 | 1 | 449 |
| 1544 | 180 | 6.956522 | 58 | 1 | 449 |
| 1544 | 243 | 13.09091 | 58 | 1 | 449 |
| 1544 | 329 | 7.85169 | 58 | 1 | 449 |
| 1544 | 447 | 33.33333 | 58 | 1 | 449 |
| 1545 | 0 | 2.777778 | 72 | 0 | 1035 |
| 1545 | 56 | 0.555556 | 72 | 0 | 1035 |
| 1545 | 168 | 0.728155 | 72 | 0 | 1035 |
| 1545 | 280 | 0.721154 | 72 | 0 | 1035 |
| 1545 | 336 | 1.388889 | 72 | 0 | 1035 |
| 1545 | 392 | 2.857143 | 72 | 0 | 1035 |
| 1545 | 502 | 2.857143 | 72 | 0 | 1035 |
| 1545 | 558 | 2.970297 | 72 | 0 | 1035 |
| 1546 | 0 | 1.339286 | 71 | 0 | 72 |
| 1546 | 71 | 1.075544 | 71 | 0 | 72 |
| 1547 | 0 | 2.608696 | 71 | 0 | 1122 |
| 1547 | 28 | 2.408257 | 71 | 0 | 1122 |
| 1547 | 91 | 2.419355 | 71 | 0 | 1122 |
| 1547 | 168 | 2.608696 | 71 | 0 | 1122 |
| 1547 | 252 | 2.162162 | 71 | 0 | 1122 |

| | | | | | |
|---|---|---|---|---|---|
| 1547 | 322 | 2.105263 | 71 | 0 | 1122 |
| 1547 | 413 | 2.678571 | 71 | 0 | 1122 |
| 1547 | 497 | 2.727273 | 71 | 0 | 1122 |
| 1547 | 588 | 2.803738 | 71 | 0 | 1122 |
| 1548 | 0 | 6.593407 | 68 | 0 | 660 |
| 1548 | 28 | 6.25 | 68 | 0 | 660 |
| 1548 | 112 | 6.382979 | 68 | 0 | 660 |
| 1548 | 175 | 8.421053 | 68 | 0 | 660 |
| 1548 | 238 | 8.910891 | 68 | 0 | 660 |
| 1548 | 364 | 7.128713 | 68 | 0 | 660 |
| 1548 | 434 | 7.272727 | 68 | 0 | 660 |
| 1548 | 526 | 8.835905 | 68 | 0 | 660 |
| 1548 | 589 | 7.5 | 68 | 0 | 660 |
| 1549 | 0 | 1.239669 | 70 | 1 | 959 |
| 1549 | 28 | 1.092896 | 70 | 1 | 959 |
| 1549 | 77 | 1.980198 | 70 | 1 | 959 |
| 1549 | 168 | 3.106796 | 70 | 1 | 959 |
| 1549 | 259 | 4.938272 | 70 | 1 | 959 |
| 1549 | 336 | 3.846154 | 70 | 1 | 959 |
| 1549 | 392 | 3.773585 | 70 | 1 | 959 |
| 1549 | 504 | 6.666667 | 70 | 1 | 959 |
| 1549 | 588 | 5.769231 | 70 | 1 | 959 |
| 1550 | 0 | 3.846154 | 63 | 1 | 114 |
| 1550 | 68 | 2.608291 | 63 | 1 | 114 |
| 1551 | 0 | 2.803738 | 72 | 0 | 1156 |
| 1551 | 28 | 2.631579 | 72 | 0 | 1156 |
| 1551 | 84 | 2.654867 | 72 | 0 | 1156 |
| 1551 | 168 | 2.162162 | 72 | 0 | 1156 |
| 1551 | 259 | 2.702703 | 72 | 0 | 1156 |
| 1551 | 350 | 2.586207 | 72 | 0 | 1156 |
| 1551 | 422 | 1.707317 | 72 | 0 | 1156 |
| 1551 | 490 | 2.439024 | 72 | 0 | 1156 |
| 1551 | 553 | 2.051282 | 72 | 0 | 1156 |
| 1552 | 0 | 6.741573 | 89 | 1 | 273 |
| 1552 | 28 | 6.763285 | 89 | 1 | 273 |
| 1552 | 84 | 6.741573 | 89 | 1 | 273 |
| 1552 | 147 | 9.782609 | 89 | 1 | 273 |
| 1552 | 231 | 25.3012 | 89 | 1 | 273 |
| 1553 | 0 | 14.32225 | 83 | 1 | 270 |

| | | | | | |
|---|---|---|---|---|---|
| 1553 | 24 | 8.205128 | 83 | 1 | 270 |
| 1553 | 94 | 8.888889 | 83 | 1 | 270 |
| 1553 | 164 | 13.58491 | 83 | 1 | 270 |
| 1553 | 251 | 21.28378 | 83 | 1 | 270 |
| 1554 | 0 | 4.285714 | 74 | 0 | 942 |
| 1554 | 35 | 2.830189 | 74 | 0 | 942 |
| 1554 | 91 | 2.016807 | 74 | 0 | 942 |
| 1554 | 161 | 2.678571 | 74 | 0 | 942 |
| 1554 | 273 | 4.301075 | 74 | 0 | 942 |
| 1554 | 364 | 10.54945 | 74 | 0 | 942 |
| 1554 | 427 | 15.73034 | 74 | 0 | 942 |
| 1554 | 525 | 18 | 74 | 0 | 942 |
| 1554 | 581 | 16.66667 | 74 | 0 | 942 |
| 1555 | 0 | 2.222222 | 88 | 1 | 219 |
| 1555 | 98 | 2.181818 | 88 | 1 | 219 |
| 1555 | 154 | 1.48122 | 88 | 1 | 219 |
| 1555 | 217 | 12.63158 | 88 | 1 | 219 |
| 1556 | 0 | 2.777778 | 81 | 0 | 832 |
| 1556 | 28 | 1.315789 | 81 | 0 | 832 |
| 1556 | 91 | 2.307692 | 81 | 0 | 832 |
| 1556 | 168 | 1.904762 | 81 | 0 | 832 |
| 1557 | 0 | 5.454545 | 76 | 0 | 377 |
| 1557 | 29 | 5.504587 | 76 | 0 | 377 |
| 1557 | 84 | 5.679513 | 76 | 0 | 377 |
| 1557 | 168 | 5.940594 | 76 | 0 | 377 |
| 1557 | 252 | 4.324324 | 76 | 0 | 377 |
| 1557 | 343 | 5.660377 | 76 | 0 | 377 |
| 1558 | 0 | 2.727273 | 85 | 0 | 1177 |
| 1558 | 28 | 2.803738 | 85 | 0 | 1177 |
| 1558 | 84 | 2.857143 | 85 | 0 | 1177 |
| 1558 | 174 | 2.970297 | 85 | 0 | 1177 |
| 1558 | 322 | 2.941176 | 85 | 0 | 1177 |
| 1558 | 441 | 2.912621 | 85 | 0 | 1177 |
| 1558 | 469 | 2.912621 | 85 | 0 | 1177 |
| 1558 | 553 | 2.912621 | 85 | 0 | 1177 |
| 1559 | 0 | 1.73913 | 89 | 0 | 785 |
| 1559 | 98 | 1.282051 | 89 | 0 | 785 |
| 1559 | 140 | 3.703704 | 89 | 0 | 785 |
| 1559 | 273 | 3.773585 | 89 | 0 | 785 |

| | | | | | |
|---|---|---|---|---|---|
| 1559 | 364 | 4.272363 | 89 | 0 | 785 |
| 1559 | 469 | 4.938272 | 89 | 0 | 785 |
| 1559 | 581 | 4.247788 | 89 | 0 | 785 |
| 1560 | 0 | 3.738318 | 74 | 0 | 638 |
| 1560 | 28 | 5.504587 | 74 | 0 | 638 |
| 1560 | 77 | 4.918033 | 74 | 0 | 638 |
| 1560 | 168 | 6.779661 | 74 | 0 | 638 |
| 1560 | 252 | 1.162791 | 74 | 0 | 638 |
| 1560 | 336 | 2.479339 | 74 | 0 | 638 |
| 1560 | 392 | 1.612903 | 74 | 0 | 638 |
| 1560 | 497 | 1.875 | 74 | 0 | 638 |
| 1560 | 581 | 2.380952 | 74 | 0 | 638 |
| 1561 | 0 | 4.628099 | 62 | 0 | 734 |
| 1561 | 30 | 4.172876 | 62 | 0 | 734 |
| 1561 | 105 | 4.6875 | 62 | 0 | 734 |
| 1561 | 168 | 3.418803 | 62 | 0 | 734 |
| 1561 | 252 | 3.246377 | 62 | 0 | 734 |
| 1561 | 343 | 5.882353 | 62 | 0 | 734 |
| 1561 | 427 | 5.309735 | 62 | 0 | 734 |
| 1561 | 504 | 5.217391 | 62 | 0 | 734 |
| 1561 | 588 | 8.247423 | 62 | 0 | 734 |
| 1562 | 0 | 5.172414 | 51 | 1 | 525 |
| 1562 | 28 | 4.958678 | 51 | 1 | 525 |
| 1562 | 84 | 5.084746 | 51 | 1 | 525 |
| 1562 | 174 | 5.940594 | 51 | 1 | 525 |
| 1562 | 252 | 5.607477 | 51 | 1 | 525 |
| 1562 | 336 | 7.76699 | 51 | 1 | 525 |
| 1562 | 420 | 7.017544 | 51 | 1 | 525 |
| 1562 | 505 | 7.214171 | 51 | 1 | 525 |
| 1563 | 0 | 8.648649 | 55 | 1 | 371 |
| 1563 | 77 | 8.510638 | 55 | 1 | 371 |
| 1563 | 161 | 11.88119 | 55 | 1 | 371 |
| 1563 | 239 | 24.23077 | 55 | 1 | 371 |
| 1563 | 336 | 19.78022 | 55 | 1 | 371 |
| 1564 | 0 | 6.25 | 49 | 1 | 904 |
| 1564 | 67 | 6.382979 | 49 | 1 | 904 |
| 1564 | 154 | 9.50495 | 49 | 1 | 904 |
| 1564 | 259 | 4.897959 | 49 | 1 | 904 |
| 1564 | 336 | 5.177994 | 49 | 1 | 904 |

| | | | | | |
|---|---|---|---|---|---|
| 1564 | 399 | 12 | 49 | 1 | 904 |
| 1564 | 497 | 9.896907 | 49 | 1 | 904 |
| 1564 | 560 | 11.00917 | 49 | 1 | 904 |
| 1565 | 0 | 3.773585 | 57 | 1 | 274 |
| 1565 | 84 | 13.04348 | 57 | 1 | 274 |
| 1565 | 168 | 12.90323 | 57 | 1 | 274 |
| 1565 | 252 | 21.81818 | 57 | 1 | 274 |
| 1566 | 0 | 5.274725 | 64 | 1 | 574 |
| 1566 | 84 | 7.407407 | 64 | 1 | 574 |
| 1566 | 147 | 5.405405 | 64 | 1 | 574 |
| 1566 | 210 | 9.333333 | 64 | 1 | 574 |
| 1566 | 329 | 5.517241 | 64 | 1 | 574 |
| 1566 | 399 | 5.783133 | 64 | 1 | 574 |
| 1566 | 504 | 35.8209 | 64 | 1 | 574 |
| 1567 | 0 | 1.960784 | 76 | 1 | 472 |
| 1567 | 42 | 4 | 76 | 1 | 472 |
| 1567 | 105 | 3.624595 | 76 | 1 | 472 |
| 1567 | 147 | 4.307692 | 76 | 1 | 472 |
| 1567 | 238 | 2.539683 | 76 | 1 | 472 |
| 1567 | 350 | 4.166667 | 76 | 1 | 472 |
| 1567 | 420 | 4.597701 | 76 | 1 | 472 |
| 1568 | 0 | 2.016807 | 78 | 0 | 776 |
| 1568 | 35 | 2.068966 | 78 | 0 | 776 |
| 1568 | 105 | 2.181818 | 78 | 0 | 776 |
| 1568 | 189 | 2.380952 | 78 | 0 | 776 |
| 1568 | 252 | 2.654867 | 78 | 0 | 776 |
| 1568 | 350 | 2.201835 | 78 | 0 | 776 |
| 1568 | 427 | 2.201835 | 78 | 0 | 776 |
| 1568 | 462 | 2.123894 | 78 | 0 | 776 |
| 1568 | 567 | 6.273338 | 78 | 0 | 776 |
| 1569 | 0 | 9.302326 | 55 | 0 | 1114 |
| 1569 | 42 | 3.007519 | 55 | 0 | 1114 |
| 1569 | 105 | 2.797203 | 55 | 0 | 1114 |
| 1569 | 196 | 13.33333 | 55 | 0 | 1114 |
| 1569 | 266 | 3.72093 | 55 | 0 | 1114 |
| 1569 | 336 | 2.983838 | 55 | 0 | 1114 |
| 1569 | 434 | 4.47205 | 55 | 0 | 1114 |
| 1569 | 504 | 4.321729 | 55 | 0 | 1114 |
| 1570 | 0 | 3.305785 | 68 | 0 | 1122 |

| | | | | | |
|---|---|---|---|---|---|
| 1570 | 28 | 2.735043 | 68 | 0 | 1122 |
| 1570 | 63 | 5.607477 | 68 | 0 | 1122 |
| 1570 | 182 | 5.454545 | 68 | 0 | 1122 |
| 1570 | 235 | 4.363636 | 68 | 0 | 1122 |
| 1570 | 328 | 5.357143 | 68 | 0 | 1122 |
| 1570 | 420 | 5.555556 | 68 | 0 | 1122 |
| 1570 | 511 | 5.660377 | 68 | 0 | 1122 |
| 1570 | 567 | 5.714286 | 68 | 0 | 1122 |
| 1571 | 0 | 5.412371 | 74 | 0 | 631 |
| 1571 | 77 | 2.397602 | 74 | 0 | 631 |
| 1571 | 154 | 2.321083 | 74 | 0 | 631 |
| 1572 | 0 | 2.702703 | 83 | 0 | 1338 |
| 1572 | 28 | 2.631579 | 83 | 0 | 1338 |
| 1572 | 91 | 2.142857 | 83 | 0 | 1338 |
| 1572 | 175 | 2.631579 | 83 | 0 | 1338 |
| 1572 | 259 | 2.564103 | 83 | 0 | 1338 |
| 1572 | 336 | 2.758621 | 83 | 0 | 1338 |
| 1572 | 421 | 4.395604 | 83 | 0 | 1338 |
| 1572 | 595 | 5.405405 | 83 | 0 | 1338 |
| 1573 | 0 | 2.330097 | 73 | 1 | 186 |
| 1573 | 35 | 2.702703 | 73 | 1 | 186 |
| 1574 | 0 | 2.941176 | 75 | 0 | 687 |
| 1574 | 28 | 4.8 | 75 | 0 | 687 |
| 1574 | 91 | 5.714286 | 75 | 0 | 687 |
| 1574 | 182 | 11.65049 | 75 | 0 | 687 |
| 1574 | 245 | 13.58491 | 75 | 0 | 687 |
| 1574 | 350 | 13.21101 | 75 | 0 | 687 |
| 1574 | 441 | 14.87603 | 75 | 0 | 687 |
| 1574 | 525 | 15.25424 | 75 | 0 | 687 |
| 1574 | 588 | 15.78947 | 75 | 0 | 687 |
| 1575 | 0 | 2.181818 | 76 | 0 | 646 |
| 1575 | 77 | 0.877193 | 76 | 0 | 646 |
| 1575 | 161 | 1.257862 | 76 | 0 | 646 |
| 1575 | 245 | 2.222222 | 76 | 0 | 646 |
| 1576 | 0 | 2.830189 | 75 | 1 | 426 |
| 1576 | 77 | 4.285714 | 75 | 1 | 426 |
| 1576 | 161 | 1.744548 | 75 | 1 | 426 |
| 1576 | 262 | 5.333333 | 75 | 1 | 426 |
| 1576 | 325 | 4.444444 | 75 | 1 | 426 |

| | | | | | |
|---|---|---|---|---|---|
| 1577 | 0 | 3.204272 | 72 | 1 | 311 |
| 1577 | 28 | 3.394625 | 72 | 1 | 311 |
| 1577 | 84 | 6.896552 | 72 | 1 | 311 |
| 1577 | 182 | 5.517241 | 72 | 1 | 311 |
| 1577 | 252 | 4.597701 | 72 | 1 | 311 |
| 1578 | 0 | 4.651163 | 80 | 0 | 673 |
| 1578 | 28 | 4.761905 | 80 | 0 | 673 |
| 1578 | 84 | 4.8 | 80 | 0 | 673 |
| 1578 | 168 | 2.752294 | 80 | 0 | 673 |
| 1578 | 252 | 5 | 80 | 0 | 673 |
| 1578 | 420 | 5.172414 | 80 | 0 | 673 |
| 1578 | 504 | 5.217391 | 80 | 0 | 673 |
| 1578 | 560 | 5.607477 | 80 | 0 | 673 |
| 1579 | 0 | 1.754386 | 76 | 0 | 561 |
| 1579 | 56 | 1.73913 | 76 | 0 | 561 |
| 1579 | 168 | 1.769912 | 76 | 0 | 561 |
| 1579 | 224 | 1.785714 | 76 | 0 | 561 |
| 1579 | 336 | 2.702703 | 76 | 0 | 561 |
| 1579 | 392 | 3.738318 | 76 | 0 | 561 |
| 1579 | 504 | 1.666667 | 76 | 0 | 561 |
| 1579 | 560 | 1.724138 | 76 | 0 | 561 |
| 1580 | 0 | 4.247788 | 80 | 0 | 791 |
| 1580 | 35 | 4.137931 | 80 | 0 | 791 |
| 1580 | 70 | 3.539823 | 80 | 0 | 791 |
| 1580 | 154 | 2.542373 | 80 | 0 | 791 |
| 1580 | 266 | 2.394868 | 80 | 0 | 791 |
| 1580 | 322 | 2.35393 | 80 | 0 | 791 |
| 1580 | 392 | 0.900901 | 80 | 0 | 791 |
| 1580 | 615 | 2.77686 | 80 | 0 | 791 |
| 1581 | 0 | 2.5 | 62 | 0 | 924 |
| 1581 | 28 | 1.219512 | 62 | 0 | 924 |
| 1581 | 83 | 1.172161 | 62 | 0 | 924 |
| 1581 | 175 | 2.272727 | 62 | 0 | 924 |
| 1581 | 252 | 3.883495 | 62 | 0 | 924 |
| 1581 | 336 | 6.315789 | 62 | 0 | 924 |
| 1581 | 423 | 5.555556 | 62 | 0 | 924 |
| 1581 | 504 | 5.405405 | 62 | 0 | 924 |
| 1581 | 588 | 5.882353 | 62 | 0 | 924 |
| 1582 | 0 | 1.378122 | 48 | 1 | 286 |

| | | | | | |
|---|---|---|---|---|---|
| 1582 | 28 | 1.357082 | 48 | 1 | 286 |
| 1582 | 56 | 3.363363 | 48 | 1 | 286 |
| 1582 | 164 | 3.703704 | 48 | 1 | 286 |
| 1582 | 248 | 10.25641 | 48 | 1 | 286 |
| 1583 | 0 | 5.504587 | 77 | 1 | 1020 |
| 1583 | 21 | 5.714286 | 77 | 1 | 1020 |
| 1583 | 80 | 5.940594 | 77 | 1 | 1020 |
| 1583 | 168 | 11.32075 | 77 | 1 | 1020 |
| 1583 | 224 | 18.18182 | 77 | 1 | 1020 |
| 1583 | 336 | 18 | 77 | 1 | 1020 |
| 1583 | 417 | 16.42229 | 77 | 1 | 1020 |
| 1583 | 501 | 16.98113 | 77 | 1 | 1020 |
| 1583 | 564 | 15 | 77 | 1 | 1020 |
| 1584 | 0 | 1.709402 | 39 | 1 | 1167 |
| 1584 | 28 | 1.666667 | 39 | 1 | 1167 |
| 1584 | 84 | 2.777778 | 39 | 1 | 1167 |
| 1584 | 168 | 3.738318 | 39 | 1 | 1167 |
| 1584 | 224 | 3.254868 | 39 | 1 | 1167 |
| 1584 | 336 | 3.703704 | 39 | 1 | 1167 |
| 1584 | 392 | 3.669725 | 39 | 1 | 1167 |
| 1584 | 504 | 3.703704 | 39 | 1 | 1167 |
| 1584 | 588 | 3.749582 | 39 | 1 | 1167 |
| 1585 | 0 | 12.2449 | 83 | 0 | 932 |
| 1585 | 28 | 12.90323 | 83 | 0 | 932 |
| 1585 | 84 | 18.94737 | 83 | 0 | 932 |
| 1585 | 140 | 4.958678 | 83 | 0 | 932 |
| 1585 | 224 | 8.888889 | 83 | 0 | 932 |
| 1585 | 333 | 2.353106 | 83 | 0 | 932 |
| 1585 | 515 | 5.970149 | 83 | 0 | 932 |
| 1585 | 592 | 4.133858 | 83 | 0 | 932 |
| 1586 | 0 | 3.773585 | 49 | 1 | 217 |
| 1586 | 42 | 11.16279 | 49 | 1 | 217 |
| 1586 | 77 | 15.82418 | 49 | 1 | 217 |
| 1586 | 161 | 21.17647 | 49 | 1 | 217 |
| 1587 | 0 | 1.227621 | 81 | 1 | 594 |
| 1587 | 42 | 3.234501 | 81 | 1 | 594 |
| 1587 | 91 | 3.145478 | 81 | 1 | 594 |
| 1587 | 140 | 2.123894 | 81 | 1 | 594 |
| 1587 | 293 | 3.418803 | 81 | 1 | 594 |

| | | | | | |
|---|---|---|---|---|---|
| 1587 | 335 | 2.199529 | 81 | 1 | 594 |
| 1587 | 385 | 3.060109 | 81 | 1 | 594 |
| 1587 | 511 | 6.524272 | 81 | 1 | 594 |
| 1587 | 567 | 5.645161 | 81 | 1 | 594 |
| 1588 | 0 | 1.41844 | 80 | 0 | 819 |
| 1588 | 28 | 1.181102 | 80 | 0 | 819 |
| 1588 | 84 | 1.269841 | 80 | 0 | 819 |
| 1588 | 168 | 1.960784 | 80 | 0 | 819 |
| 1588 | 252 | 4.301075 | 80 | 0 | 819 |
| 1588 | 336 | 6.122449 | 80 | 0 | 819 |
| 1588 | 420 | 5.607477 | 80 | 0 | 819 |
| 1588 | 504 | 5.940594 | 80 | 0 | 819 |
| 1588 | 588 | 6 | 80 | 0 | 819 |
| 1589 | 0 | 5.326569 | 75 | 0 | 267 |
| 1589 | 38 | 10.78652 | 75 | 0 | 267 |
| 1589 | 104 | 10.37998 | 75 | 0 | 267 |
| 1589 | 183 | 7.407407 | 75 | 0 | 267 |
| 1589 | 260 | 3.137255 | 75 | 0 | 267 |
| 1590 | 0 | 0.384 | 73 | 0 | 680 |
| 1590 | 31 | 0.390244 | 73 | 0 | 680 |
| 1590 | 105 | 1.769912 | 73 | 0 | 680 |
| 1590 | 147 | 1.169591 | 73 | 0 | 680 |
| 1590 | 266 | 3.034134 | 73 | 0 | 680 |
| 1590 | 315 | 1.025641 | 73 | 0 | 680 |
| 1590 | 467 | 2.631579 | 73 | 0 | 680 |
| 1590 | 476 | 2.586207 | 73 | 0 | 680 |
| 1590 | 616 | 3.738318 | 73 | 0 | 680 |
| 1591 | 0 | 5.16129 | 26 | 0 | 330 |
| 1591 | 70 | 1.641587 | 26 | 0 | 330 |
| 1591 | 154 | 4.195804 | 26 | 0 | 330 |
| 1591 | 245 | 4.705882 | 26 | 0 | 330 |
| 1591 | 329 | 7.594937 | 26 | 0 | 330 |
| 1592 | 0 | 5.221445 | 61 | 1 | 306 |
| 1592 | 40 | 25.53191 | 61 | 1 | 306 |
| 1592 | 89 | 16.32653 | 61 | 1 | 306 |
| 1592 | 173 | 4.40367 | 61 | 1 | 306 |
| 1593 | 0 | 4.207363 | 85 | 0 | 1164 |
| 1593 | 22 | 4.285714 | 85 | 0 | 1164 |
| 1593 | 113 | 2.830189 | 85 | 0 | 1164 |

| | | | | | |
|---|---|---|---|---|---|
| 1593 | 155 | 2.321083 | 85 | 0 | 1164 |
| 1593 | 253 | 10.43478 | 85 | 0 | 1164 |
| 1593 | 302 | 12.10811 | 85 | 0 | 1164 |
| 1593 | 400 | 15.65217 | 85 | 0 | 1164 |
| 1593 | 491 | 15.65217 | 85 | 0 | 1164 |
| 1594 | 0 | 2.201835 | 63 | 0 | 582 |
| 1594 | 35 | 2.654867 | 63 | 0 | 582 |
| 1594 | 98 | 2.162162 | 63 | 0 | 582 |
| 1594 | 161 | 2.222222 | 63 | 0 | 582 |
| 1594 | 259 | 2.631579 | 63 | 0 | 582 |
| 1594 | 343 | 2.702703 | 63 | 0 | 582 |
| 1594 | 434 | 2.123894 | 63 | 0 | 582 |
| 1594 | 497 | 3.809524 | 63 | 0 | 582 |
| 1594 | 581 | 2.654867 | 63 | 0 | 582 |
| 1595 | 0 | 3.603604 | 89 | 0 | 708 |
| 1595 | 28 | 7.272727 | 89 | 0 | 708 |
| 1595 | 74 | 3.010753 | 89 | 0 | 708 |
| 1595 | 170 | 3.988604 | 89 | 0 | 708 |
| 1595 | 259 | 3.636364 | 89 | 0 | 708 |
| 1595 | 315 | 4.761905 | 89 | 0 | 708 |
| 1595 | 420 | 2.375398 | 89 | 0 | 708 |
| 1595 | 504 | 3.669725 | 89 | 0 | 708 |
| 1595 | 588 | 3.786342 | 89 | 0 | 708 |
| 1596 | 0 | 2.424242 | 54 | 1 | 188 |
| 1596 | 84 | 4.324324 | 54 | 1 | 188 |
| 1596 | 147 | 6.095791 | 54 | 1 | 188 |
| 1597 | 0 | 3.669725 | 66 | 1 | 702 |
| 1597 | 28 | 11.42857 | 66 | 1 | 702 |
| 1597 | 56 | 2.222222 | 66 | 1 | 702 |
| 1597 | 119 | 1.785714 | 66 | 1 | 702 |
| 1597 | 203 | 3.296703 | 66 | 1 | 702 |
| 1597 | 294 | 4.247788 | 66 | 1 | 702 |
| 1597 | 399 | 3.305785 | 66 | 1 | 702 |
| 1597 | 483 | 3.636364 | 66 | 1 | 702 |
| 1597 | 567 | 3.508772 | 66 | 1 | 702 |
| 1598 | 0 | 8.163265 | 75 | 0 | 1366 |
| 1598 | 21 | 4.597701 | 75 | 0 | 1366 |
| 1598 | 84 | 6.233766 | 75 | 0 | 1366 |
| 1598 | 161 | 14.28571 | 75 | 0 | 1366 |

| | | | | | |
|---|---|---|---|---|---|
| 1598 | 252 | 11.53846 | 75 | 0 | 1366 |
| 1598 | 336 | 11.32075 | 75 | 0 | 1366 |
| 1598 | 409 | 6.440072 | 75 | 0 | 1366 |
| 1598 | 500 | 14.41648 | 75 | 0 | 1366 |
| 1598 | 588 | 11.42857 | 75 | 0 | 1366 |
| 1599 | 0 | 2.614379 | 71 | 1 | 953 |
| 1599 | 28 | 1.248885 | 71 | 1 | 953 |
| 1599 | 84 | 5.106383 | 71 | 1 | 953 |
| 1599 | 168 | 3.603604 | 71 | 1 | 953 |
| 1599 | 252 | 5.660377 | 71 | 1 | 953 |
| 1599 | 337 | 6.451613 | 71 | 1 | 953 |
| 1599 | 420 | 13.33333 | 71 | 1 | 953 |
| 1599 | 504 | 12.76596 | 71 | 1 | 953 |
| 1599 | 588 | 12.90323 | 71 | 1 | 953 |
| 1600 | 0 | 1.565704 | 61 | 1 | 519 |
| 1600 | 28 | 1.845748 | 61 | 1 | 519 |
| 1600 | 88 | 5.555556 | 61 | 1 | 519 |
| 1600 | 161 | 3.84 | 61 | 1 | 519 |
| 1600 | 245 | 4.375 | 61 | 1 | 519 |
| 1600 | 335 | 4.705882 | 61 | 1 | 519 |
| 1600 | 389 | 10 | 61 | 1 | 519 |
| 1601 | 0 | 2.752294 | 88 | 0 | 911 |
| 1601 | 35 | 10.43478 | 88 | 0 | 911 |
| 1601 | 91 | 10.90909 | 88 | 0 | 911 |
| 1601 | 161 | 1.758242 | 88 | 0 | 911 |
| 1601 | 252 | 1.694915 | 88 | 0 | 911 |
| 1601 | 371 | 4.660194 | 88 | 0 | 911 |
| 1601 | 511 | 5.811138 | 88 | 0 | 911 |
| 1601 | 553 | 11.42857 | 88 | 0 | 911 |
| 1602 | 0 | 2.521008 | 74 | 0 | 981 |
| 1602 | 28 | 2.884615 | 74 | 0 | 981 |
| 1602 | 84 | 2.702703 | 74 | 0 | 981 |
| 1602 | 168 | 2.803738 | 74 | 0 | 981 |
| 1602 | 252 | 2.941176 | 74 | 0 | 981 |
| 1602 | 336 | 3.26087 | 74 | 0 | 981 |
| 1602 | 420 | 2.830189 | 74 | 0 | 981 |
| 1602 | 504 | 2.777778 | 74 | 0 | 981 |
| 1602 | 588 | 2.803738 | 74 | 0 | 981 |
| 1603 | 0 | 12.12121 | 41 | 1 | 236 |

| | | | | | |
|---|---|---|---|---|---|
| 1603 | 35 | 5.283019 | 41 | 1 | 236 |
| 1603 | 98 | 10.43478 | 41 | 1 | 236 |
| 1603 | 161 | 10.43478 | 41 | 1 | 236 |
| 1603 | 217 | 10.90909 | 41 | 1 | 236 |
| 1604 | 0 | 5.607477 | 78 | 0 | 834 |
| 1604 | 28 | 5.607477 | 78 | 0 | 834 |
| 1604 | 84 | 5.769231 | 78 | 0 | 834 |
| 1604 | 175 | 4.660194 | 78 | 0 | 834 |
| 1604 | 273 | 4.705882 | 78 | 0 | 834 |
| 1604 | 329 | 5.769231 | 78 | 0 | 834 |
| 1604 | 427 | 7.920792 | 78 | 0 | 834 |
| 1604 | 511 | 5.504587 | 78 | 0 | 834 |
| 1604 | 602 | 4.571429 | 78 | 0 | 834 |
| 1605 | 0 | 5.555556 | 72 | 0 | 1312 |
| 1605 | 28 | 4.444444 | 72 | 0 | 1312 |
| 1605 | 63 | 5.454545 | 72 | 0 | 1312 |
| 1605 | 154 | 5.454545 | 72 | 0 | 1312 |
| 1605 | 210 | 5.504587 | 72 | 0 | 1312 |
| 1605 | 301 | 4.40367 | 72 | 0 | 1312 |
| 1605 | 392 | 5.309735 | 72 | 0 | 1312 |
| 1605 | 490 | 4.067797 | 72 | 0 | 1312 |
| 1605 | 588 | 5.454545 | 72 | 0 | 1312 |
| 1606 | 0 | 9.292035 | 69 | 0 | 591 |
| 1606 | 66 | 2.181818 | 69 | 0 | 591 |
| 1606 | 185 | 2.586207 | 69 | 0 | 591 |
| 1606 | 238 | 3.418803 | 69 | 0 | 591 |
| 1606 | 325 | 11.23746 | 69 | 0 | 591 |
| 1606 | 423 | 6.222222 | 69 | 0 | 591 |
| 1607 | 0 | 6.185567 | 79 | 1 | 489 |
| 1607 | 28 | 6.25 | 79 | 1 | 489 |
| 1607 | 56 | 12.90323 | 79 | 1 | 489 |
| 1607 | 175 | 12.5 | 79 | 1 | 489 |
| 1607 | 266 | 10.54945 | 79 | 1 | 489 |
| 1607 | 336 | 13.7931 | 79 | 1 | 489 |
| 1607 | 420 | 17.94872 | 79 | 1 | 489 |
| 1608 | 0 | 1.88383 | 73 | 1 | 515 |
| 1608 | 49 | 8.163265 | 73 | 1 | 515 |
| 1608 | 168 | 4.363636 | 73 | 1 | 515 |
| 1608 | 259 | 2.521008 | 73 | 1 | 515 |

| | | | | | |
|---|---|---|---|---|---|
| 1608 | 322 | 2.564103 | 73 | 1 | 515 |
| 1608 | 420 | 4.528302 | 73 | 1 | 515 |
| 1609 | 0 | 3.508772 | 88 | 0 | 228 |
| 1609 | 28 | 3.636364 | 88 | 0 | 228 |
| 1609 | 84 | 2.93578 | 88 | 0 | 228 |
| 1609 | 161 | 1.812298 | 88 | 0 | 228 |
| 1610 | 0 | 3.418803 | 74 | 0 | 1120 |
| 1610 | 28 | 3.418803 | 74 | 0 | 1120 |
| 1610 | 91 | 1.355932 | 74 | 0 | 1120 |
| 1610 | 182 | 1.754386 | 74 | 0 | 1120 |
| 1610 | 238 | 2.678571 | 74 | 0 | 1120 |
| 1610 | 329 | 1.403509 | 74 | 0 | 1120 |
| 1610 | 434 | 1.415929 | 74 | 0 | 1120 |
| 1610 | 504 | 1.834862 | 74 | 0 | 1120 |
| 1611 | 0 | 3.389831 | 73 | 0 | 1006 |
| 1611 | 28 | 3.448276 | 73 | 0 | 1006 |
| 1611 | 84 | 5.769231 | 73 | 0 | 1006 |
| 1611 | 168 | 5.263158 | 73 | 0 | 1006 |
| 1611 | 266 | 8.411215 | 73 | 0 | 1006 |
| 1611 | 350 | 11.76471 | 73 | 0 | 1006 |
| 1611 | 406 | 17.82178 | 73 | 0 | 1006 |
| 1611 | 518 | 16.82243 | 73 | 0 | 1006 |
| 1611 | 602 | 17.82178 | 73 | 0 | 1006 |
| 1612 | 0 | 13.18681 | 68 | 0 | 1147 |
| 1612 | 28 | 10.32258 | 68 | 0 | 1147 |
| 1612 | 63 | 9.621993 | 68 | 0 | 1147 |
| 1612 | 133 | 12.2449 | 68 | 0 | 1147 |
| 1612 | 259 | 10.21277 | 68 | 0 | 1147 |
| 1612 | 364 | 8.695652 | 68 | 0 | 1147 |
| 1612 | 434 | 9.6 | 68 | 0 | 1147 |
| 1612 | 504 | 12.37113 | 68 | 0 | 1147 |
| 1612 | 623 | 8.333333 | 68 | 0 | 1147 |
| 1613 | 0 | 4.067797 | 51 | 0 | 120 |
| 1613 | 35 | 5.555556 | 51 | 0 | 120 |
| 1613 | 91 | 5.825243 | 51 | 0 | 120 |
| 1614 | 0 | 1.119403 | 73 | 0 | 701 |
| 1614 | 28 | 3.636364 | 73 | 0 | 701 |
| 1614 | 70 | 7.843137 | 73 | 0 | 701 |
| 1614 | 189 | 8.347826 | 73 | 0 | 701 |

| | | | | | |
|---|---|---|---|---|---|
| 1614 | 273 | 2.431611 | 73 | 0 | 701 |
| 1614 | 301 | 2.721088 | 73 | 0 | 701 |
| 1614 | 434 | 3.361345 | 73 | 0 | 701 |
| 1614 | 504 | 4.724409 | 73 | 0 | 701 |
| 1614 | 595 | 2.298851 | 73 | 0 | 701 |
| 1615 | 0 | 1.889764 | 70 | 0 | 1104 |
| 1615 | 35 | 1.889764 | 70 | 0 | 1104 |
| 1615 | 105 | 2.44898 | 70 | 0 | 1104 |
| 1615 | 168 | 5.357143 | 70 | 0 | 1104 |
| 1615 | 266 | 6.153846 | 70 | 0 | 1104 |
| 1615 | 336 | 7.758621 | 70 | 0 | 1104 |
| 1615 | 420 | 6.153846 | 70 | 0 | 1104 |
| 1615 | 483 | 5.853659 | 70 | 0 | 1104 |
| 1615 | 581 | 7.804878 | 70 | 0 | 1104 |
| 1616 | 0 | 5.357143 | 82 | 0 | 990 |
| 1616 | 56 | 3.934426 | 82 | 0 | 990 |
| 1616 | 161 | 3.603604 | 82 | 0 | 990 |
| 1616 | 224 | 2.689076 | 82 | 0 | 990 |
| 1616 | 329 | 2.666667 | 82 | 0 | 990 |
| 1616 | 427 | 2.644628 | 82 | 0 | 990 |
| 1616 | 525 | 3.571429 | 82 | 0 | 990 |
| 1616 | 560 | 2.735043 | 82 | 0 | 990 |
| 1617 | 0 | 2.402402 | 60 | 0 | 1058 |
| 1617 | 35 | 2.203857 | 60 | 0 | 1058 |
| 1617 | 91 | 2.469136 | 60 | 0 | 1058 |
| 1617 | 154 | 1.694915 | 60 | 0 | 1058 |
| 1617 | 273 | 8.247423 | 60 | 0 | 1058 |
| 1617 | 329 | 2.181818 | 60 | 0 | 1058 |
| 1617 | 406 | 4.395604 | 60 | 0 | 1058 |
| 1617 | 504 | 2.803738 | 60 | 0 | 1058 |
| 1617 | 595 | 4.528302 | 60 | 0 | 1058 |
| 1618 | 0 | 5.555556 | 51 | 0 | 785 |
| 1618 | 28 | 4.363636 | 51 | 0 | 785 |
| 1618 | 91 | 3.448276 | 51 | 0 | 785 |
| 1618 | 161 | 5.555556 | 51 | 0 | 785 |
| 1618 | 245 | 8.035714 | 51 | 0 | 785 |
| 1618 | 343 | 6.060606 | 51 | 0 | 785 |
| 1618 | 420 | 6.990291 | 51 | 0 | 785 |
| 1618 | 511 | 6.545455 | 51 | 0 | 785 |

| | | | | | |
|---|---|---|---|---|---|
| 1618 | 581 | 6.486486 | 51 | 0 | 785 |
| 1619 | 0 | 2.068966 | 51 | 0 | 1128 |
| 1619 | 35 | 2.678571 | 51 | 0 | 1128 |
| 1619 | 63 | 2.201835 | 51 | 0 | 1128 |
| 1619 | 154 | 4.485981 | 51 | 0 | 1128 |
| 1619 | 217 | 5.769231 | 51 | 0 | 1128 |
| 1619 | 308 | 5.504587 | 51 | 0 | 1128 |
| 1619 | 399 | 9.411765 | 51 | 0 | 1128 |
| 1619 | 490 | 14.4 | 51 | 0 | 1128 |
| 1619 | 553 | 14.69388 | 51 | 0 | 1128 |
| 1620 | 14 | 0.763359 | 73 | 1 | 143 |
| 1620 | 70 | 6.873977 | 73 | 1 | 143 |
| 1621 | 0 | 3.603604 | 79 | 0 | 890 |
| 1621 | 21 | 4.660194 | 79 | 0 | 890 |
| 1621 | 91 | 9.896907 | 79 | 0 | 890 |
| 1621 | 161 | 8.421053 | 79 | 0 | 890 |
| 1621 | 231 | 8.971963 | 79 | 0 | 890 |
| 1621 | 322 | 11.65049 | 79 | 0 | 890 |
| 1621 | 378 | 7.692308 | 79 | 0 | 890 |
| 1621 | 497 | 3.32871 | 79 | 0 | 890 |
| 1621 | 588 | 3.846154 | 79 | 0 | 890 |
| 1622 | 0 | 4.666667 | 68 | 0 | 229 |
| 1622 | 25 | 3.168317 | 68 | 0 | 229 |
| 1622 | 95 | 1.693122 | 68 | 0 | 229 |
| 1622 | 158 | 4.494382 | 68 | 0 | 229 |
| 1623 | 0 | 0.839161 | 68 | 0 | 614 |
| 1623 | 88 | 1.869159 | 68 | 0 | 614 |
| 1623 | 186 | 1.376147 | 68 | 0 | 614 |
| 1623 | 249 | 1.223242 | 68 | 0 | 614 |
| 1623 | 312 | 1.725998 | 68 | 0 | 614 |
| 1623 | 417 | 8.247423 | 68 | 0 | 614 |
| 1623 | 494 | 7.692308 | 68 | 0 | 614 |
| 1623 | 578 | 7.692308 | 68 | 0 | 614 |
| 1624 | 0 | 4.724409 | 74 | 1 | 513 |
| 1624 | 28 | 4.6875 | 74 | 1 | 513 |
| 1624 | 91 | 1.049869 | 74 | 1 | 513 |
| 1624 | 154 | 1.95122 | 74 | 1 | 513 |
| 1624 | 224 | 1.869159 | 74 | 1 | 513 |
| 1624 | 406 | 5.660377 | 74 | 1 | 513 |

| | | | | | |
|---|---|---|---|---|---|
| 1625 | 0 | 1.860465 | 26 | 1 | 723 |
| 1625 | 35 | 1.935484 | 26 | 1 | 723 |
| 1625 | 70 | 2.162162 | 26 | 1 | 723 |
| 1625 | 161 | 2.542373 | 26 | 1 | 723 |
| 1625 | 280 | 2.479339 | 26 | 1 | 723 |
| 1625 | 350 | 4.173913 | 26 | 1 | 723 |
| 1625 | 406 | 2.439024 | 26 | 1 | 723 |
| 1625 | 497 | 3.448276 | 26 | 1 | 723 |
| 1625 | 553 | 1.967213 | 26 | 1 | 723 |
