# Supplementary material for: Rapid increase in erythropoiesis-stimulating agent resistance is a risk factor for poor renal prognosis in patients with chronic kidney disease pre-dialysis: A BRIGHTEN study sub-analysis
Source: PLoS One. 2025 Nov 21;20(11):e0325616. doi: 10.1371/journal.pone.0325616 (PMC12637981; doi:10.1371/journal.pone.0325616)
Supplement: S4 File — (PDF) [file pone.0325616.s004.pdf]

random_I	AGE	SEX	BMI	PDIAG1_[	PDIAG1_N	PDIAG1_c	PDIAG1_(	CIGARET_	CIGARET_	CIGARET_	HYPE	MHP01FL	MHP05FL	MHP06FL	MHP04FL	MHP08FL	RASPFL	ANTH04F	ANTH03F	IRONFL	FED12	BPS	BPS1	BPD	BPD1	BCREAT	BCREAT1	BEGFRJAI	BEGFRJAI	BHGB	BHGB1	BALB	BALB1	BIRONC	BIRONC1	BFERRIT(	BFERRIT(	BTSAT	BTSAT1	BCRP	BCRP1	BPROBNF	BPROBNF	BPROTCF	BPROTCF	BHGBA1C	latent_class
1	83	F	18.02397	0	1	0	0	1	0	0	0	0	0	0	0	0	1	1	1	1	1	135	132	64	75	1.24	1.06	31.87627	37.84305	9.2	11	3.8	3.7	74	106	129	96.2	29.13386	40	2180	835	5880	8030	0.33654	NA	6.4	Class A
2	70	M	24.07625	0	1	0	0	1	0	0	0	0	0	0	0	0	0	0	0	0	0	118	128	61	73	6.56	6.05	7.32092	7.99868	10.8	11.7	4.1	3.9	81	100	97	83.7	30.11152	39.52569	499	246	1650	1250	2.22904	2.73913	5.2	Class B
3	79	F	19.78357	0	1	0	0	0	0	1	1	0	0	0	0	0	1	1	0	0	0	143	149	78	90	1.23	1.47	32.61903	26.83997	10	11.7	4.21	4.14	67	109	55.5	52.5	21.54341	32.34421	228	317	369	240	0.03627	0.04394	NA	Class A
4	88	M	15.03945	0	0	0	1	0	1	0	0	0	0	0	1	1	0	0	0	0	1	111	164	55	99	2.51	1.79	19.61066	28.38664	10.4	11.7	3.3	3.3	43	54	60	38.1	17.91667	23.27586	1010	316	660	836	0.19103	0.40625	NA	Class A
5	77	M	25.1992	0	0	1	0	1	0	0	1	0	0	0	0	0	1	1	0	0	0	148	129	76	70	2.53	2.53	20.20064	20.12597	10.2	11.4	3.6	4	68	75	31.5	38.3	31.7757	31.64557	887	598	928	780	0.41667	NA	NA	Class A
6	46	F	19.63117	0	0	1	0	0	1	0	1	0	0	0	0	0	1	1	0	1	1	133	128	105	101	2	2.15	22.38246	20.67983	10.2	10.9	4	4.4	40	62	37.2	45.4	16.19433	25.10121	1770	289	111	92	0.82192	0.4	NA	Class A
7	74	M	26.77593	1	0	0	0	0	1	0	0	1	0	0	0	0	1	1	0	0	0	128	148	65	81	3.27	3.2	15.43181	15.80149	10.1	10	4.2	4.2	105	104	28.1	11.8	32.11009	29.37853	173	260	177	110	0.92553	0.91935	6.3	Class A
8	67	M	25.21584	0	1	0	0	0	0	1	1	1	1	1	0	0	1	1	0	0	0	163	166	73	74	4.27	5.13	11.85851	9.66058	9.7	12.1	3.21	2.71	82	95	327	264	39.23445	43.7788	1470	2160	8560	5630	6.84932	11.33333	5.2	Class A
9	78	M	22.50573	0	1	0	0	0	0	1	0	0	0	0	0	0	1	1	0	1	1	127	135	60	73	2.18	2.15	23.68641	24.04822	10.4	11.2	3.6	3.5	116	123	160	131	51.32743	56.42202	523	2890	78	75	0.000931	0.11103	NA	Class A
10	68	M	26.40625	0	0	1	0	0	1	0	0	1	0	0	1	0	0	0	0	0	0	139	135	79	78	3.32	NA	15.55058	NA	11	NA	3.8	NA	88	104	165	192	38.76652	46.63677	359	209	563	619	2.81481	NA	6.3	Class A
11	89	M	20.83253	0	1	0	0	0	1	0	0	0	0	0	0	1	1	1	0	0	0	128	116	66	59	2.34	2.33	21.10594	21.20506	10.3	10.1	3.6	3.4	64	74	86.2	72.7	26.89076	29.48207	1660	2050	245	696	1.05333	4.09211	NA	Class B
12	54	F	36.31623	0	0	0	1	1	0	0	0	0	0	0	0	0	1	1	0	1	1	123	119	71	68	5.07	5.01	7.72628	7.82757	8	9.2	3.4	3.7	38	38	45.2	54.6	15.32258	14.55939	9350	8080	186	228	1.46341	1.42209	NA	Class B
13	67	M	21.44531	0	0	1	0	1	0	0	0	1	0	0	0	0	1	1	0	0	1	113	124	68	74	3.58	3.48	14.38038	14.83305	9.1	12	3.7	3.8	72	85	145	28.5	29.75207	27.77778	613	270	719	617	0.90218	2.04751	5.5	Class A
14	83	F	22.94498	0	0	1	0	1	0	0	1	0	0	0	0	0	0	0	0	1	1	155	144	81	90	0.86	0.78	47.56959	52.75049	10	11	3.6	3.9	47	30	26.7	24.1	12.73713	9.202454	2050	4500	128	123	0.30303	0.1039	5.3	Class A
15	77	F	NA	0	1	0	0	1	0	0	1	0	0	0	0	0	0	0	0	0	1	163	137	63	64	1.33	1.61	30.16683	24.47687	8.7	11.6	3.4	3.7	19	43	23.6	80.5	6.713781	15.69343	755	5300	5110	16600	2.38095	1.45614	NA	Class A
16	71	M	28.04478	1	0	0	0	1	0	0	0	1	1	0	0	1	1	0	1	0	0	126	133	46	66	1.2	NA	46.75912	NA	9.6	NA	4.3	NA	57	86	180	124	22.61905	32.57576	464	380	6130	4790	0.15209	NA	7.3	Class A
17	56	M	25.34402	1	0	0	0	1	0	0	0	1	0	0	0	0	0	0	0	0	0	137	120	82	68	1.92	2.04	29.93231	28.0115	9.1	10.7	3.9	3.9	75	NA	162	NA	23.73418	NA	70	NA	453	NA	0.96053	1.49333	7.2	Class A
18	69	M	29.77778	0	0	0	1	1	0	0	0	1	1	1	0	0	1	1	0	1	1	135	148	79	86	2.54	2.1	20.75697	25.55902	9.4	13.2	3.68	3.94	46	55	26.6	62.5	16.66667	21.40078	546	10500	1080	1500	3.79374	5.53611	5.8	Class A
19	78	M	19.95936	0	0	0	1	1	0	0	1	0	0	0	0	0	1	1	0	0	0	142	134	74	73	3.59	5.11	13.67444	9.29331	8.5	9.9	3.97	3.88	110	150	45.7	18.1	33.53659	35.88517	81	90	2380	2930	1.39064	4.10891	NA	Class A
20	74	F	26.8343	1	0	0	0	0	0	1	1	1	0	0	0	0	0	0	0	0	0	156	153	77	85	2.45	2.63	15.63972	14.47255	9.1	10.5	3.4	3.6	60	38	37.6	39.5	25.97403	13.97059	518	343	1320	1170	2.6034	4.36242	5.7	Class C
21	75	M	NA	0	0	0	1	0	1	0	1	0	0	0	0	0	0	0	0	0	0	166	157	77	69	4.41	6.56	11.04059	7.15015	9.7	10.9	3.7	NA	63	98	170	209	27.87611	40.32922	433	367	1200	1300	3.84615	3.79747	NA	Class B
22	67	M	29.49495	1	0	0	0	0	1	0	0	1	0	0	0	0	1	1	0	0	0	133	126	77	77	4.04	3.93	12.59903	12.93023	10.8	11.8	4.1	4.2	97	95	104	69.6	30.21807	26.38889	1320	1670	225	102	NA	0.44156	6.6	Class A
23	86	M	22.75899	0	1	0	0	0	0	1	0	1	0	0	0	0	1	1	0	0	0	118	113	59	55	1.77	1.86	28.92797	27.40019	11	10.3	3.5	3.4	56	83	35.2	36.8	24.77876	35.62232	1200	488	546	469	0.62937	0.71146	6.5	Class B
24	62	M	22.29568	0	1	0	0	0	1	0	1	0	0	0	0	0	1	1	0	0	0	150	161	75	86	2.65	2.56	20.43404	21.22124	9.3	11.4	3.9	3.9	69	82	119	122	27.49004	32.66932	916	1120	260	179	0.72326	1.31677	5.6	Class A
25	74	F	21.72235	0	1	0	0	1	0	0	0	0	0	0	0	0	1	1	0	0	0	122	120	74	70	3.56	4.13	10.39179	8.83339	9.6	10.6	3.8	4	94	91	156	145	25.89532	25.4902	399	677	651	662	3.45106	3.65764	5.5	Class C
26	78	M	18.23646	0	0	0	1	0	1	0	1	1	1	0	0	1	0	0	0	0	0	168	116	91	44	5.54	6.04	8.53829	7.76812	10.1	11.2	4.2	4.2	77	88	127	81.3	29.50192	31.88406	107	389	10500	27200	1.92982	2.00787	5.6	Class A
27	70	F	17.03923	0	1	0	0	0	1	0	1	0	0	0	0	0	1	1	0	0	0	140	123	79	75	1.87	1.89	21.35543	21.10833	10	11.4	3.8	4	85	108	70.6	53.8	37.44493	44.2623	405	739	230	198	0.13333	6.3	5.5	Class A
28	53	F	NA	0	0	1	0	1	0	0	0	0	0	0	0	0	1	1	0	0	0	NA	NA	NA	NA	1.35	NA	32.85973	NA	9.7	NA	3.6	NA	50	125	14	27.9	16.44737	34.43526	120	50	146	166	2.10714	NA	5.8	Class A
29	83	M	24.93588	0	0	1	0	0	1	0	0	1	0	0	1	0	0	0	0	0	0	132	137	79	73	3.75	3.89	12.85394	12.34871	10	11.6	3.7	3.6	85	88	75.2	54	35.56485	34.10853	3540	2980	391	541	0.57276	1.03418	6.4	Class A
30	70	M	18.13748	1	0	0	0	0	0	0	1	0	0	0	0	0	1	1	0	0	0	139	127	64	65	5.26	5.53	9.32181	8.82505	9.9	10	4.4	4.1	98	65	74.2	92.4	36.70412	24.16357	226	908	12400	42800	NA	NA	NA	Class A
31	70	F	18.37639	0	0	0	1	1	0	0	1	0	0	0	0	0	0	0	0	1	1	145	140	80	77	2.14	2	18.42597	19.84158	9.3	10.5	3.3	3.3	64	86	30.8	82.9	24.06015	37.3913	484	295	647	1070	1.63158	2.42857	NA	Class A
32	76	F	26.7094	1	0	0	0	1	0	0	1	1	0	0	1	1	0	0	0	0	0	144	144	60	56	2.17	2.68	17.72416	14.01664	9.3	9.2	3.5	NA	45	45	102	118	23.68421	22.61307	3100	1560	3090	2520	NA	NA	8	Class A
33	51	F	18.51782	0	1	0	0	0	0	0	0	0	0	0	0	0	1	1	0	0	0	NA	127	NA	NA	2.11	2.36	20.49314	18.13042	10.8	11.4	4.6	4.6	NA	84	NA	34.5	NA	28.57143	NA	81	NA	50	0.17647	0.27083	NA	Class A
34	60	F	20.04138	0	0	0	1	1	0	0	0	0	0	0	0	0	1	1	0	0	0	116	125	50	51	3.19	2.75	12.44428	14.56889	11	11.3	4.1	3.9	73	58	57.3	57	25.25952	21.01449	819	301	329	328	0.81667	1.37736	5.6	Class A
35	69	M	20.26875	1	0	0	0	1	0	0	0	1	0	1	0	0	1	1	0	0	1	132	137	57	61	2.3	1.95	23.13779	27.71751	8.3	12.1	4.1	4	59	51	145	58.9	23.79032	18.68132	223	390	302	1350	0.32095	1.17704	6.3	Class A
36	83	F	24	1	0	0	0	1	0	0	1	1	1	0	0	0	1	1	0	0	0	147	137	77	70	1.36	NA	28.81238	NA	10.2	NA	3.6	NA	54	42	198	94.4	25.11628	19.09091	739	445	291	422	0.53272	NA	6.7	Class A
37	44	M	25.79601	0	0	0	1	0	1	0	1	0	0	0	0	0	0	0	0	0	0	176	152	102	80	4.38	6.16	13.01242	8.90284	9.1	12	3.1	3.6	97	106	352	223	35.92593	40.92664	125	72	554	175	4.3632	1.81488	5.4	Class C
38	78	F	23.06667	0	0	0	1	1	0	0	1	0	0	0	1	1	0	0	0	1	1	154	175	96	115	2.29	2.03	16.58651	18.92406	9.4	10.1	2.7	3.1	50	72	109	69.5	28.24859	35.29412	10600	1150	937	2740	7.06452	12.51613	NA	Class C
39	80	F	20.62617	0	0	0	1	1	0	0	0	0	0	1	0	0	1	1	0	0	0	113	101	69	58	0.82	0.87	50.64621	47.47066	10.8	10.4	4.1	4.1	91	82	13.8	14.6	26.84366	25.625	80	91	140	163	0.04167	0.03509	NA	Class A
40	83	F	25.22097	0	1	0	0	1	0	0	1	0	0	0	0	0	1	1	0	0	0	148	129	77	60	1.3	1.19	30.2703	33.34436	9.9	9.7	3.8	3.9	104	72	10.4	7.3	28.26087	19.30295	481	409	182	118	0.375	0.27273	NA	Class A
41	75	M	20.47173	1	0	0	0	0	1	0	0	1	0	0	0	0	1	1	0	0	1	114	129	63	70	1.67	1.38	32.06323	39.50312	8.6	10.6	3.2	2.5	41	35	407	330	17.15481	16.27907	23800	35700	356	332	2.12821	6.35632	5.5	Class A
42	74	M	20.28123	0	0	0	1	0	1	0	0	0	0	0	0	0	0	0	0	0	0	136	150	81	83	5.91	8.29	8.07636	5.57742	9.6	10.1	3.3	3.5	65	91	186	111	27.42616	39.73799	120	95	346	582	2.08556	3.34132	NA	Class C
43	70	M	22.59295	0	0	1	0	0	1	0	0	1	0	0	0	1	0	0	0	0	0	118	146	80	88	4.35	4.38	11.47496	11.34274	10.6	12	4.3	4.3	65	54	125	72	29.01786	22.59414	968	876	1100	679	1.14923	1.72131	6.3	Class A
44	86	M	21.08628	0	1	0	0	0	1	0	1	0	1	0	0	1	1	0	1	0	0	145	115	64	53	1.61	1.74	32.0873	29.47405	9.8	11	3.5	3.7	77	70	51.7	26.3	33.47826	23.33333	339	445	6970	8930	1.04054	1.51235	NA	Class A
45	93	F	26.08369	0	0	1	0	1	0	0	1	0	0	0	0	0	0	0	0	0	0	155	162	72	81	2.26	2.01	15.99915	18.18842	9.1	9.9	3.7	3.9	57	80	408	258	30.15873	34.33476	2140	436	1830	1400	0.74663	1.17579	NA	Class A
46	47	F	22.67702	0	0	0	1	1	0	0	0	0	0	0	0	0	1	1	0	0	0	128	NA	76	NA	2.05	NA	21.65187	NA	11.1	NA	3.9	NA	65	NA	55.7	NA	25.09653	NA	2330	NA	241	NA	1.28	NA	NA	Class A
47	62	F	27.05515	1	0	0	0	1	0	0	1	1	1	0	0	0	1	1	0	0	0	143	144	80	71	1.64	1.88	25.40951	21.88299	10.4	12.1	3.5	3.3	52	76	48.7	76.3	19.54887	30.52209	176	149	5830	2240	9.54545	27.6908	10.7	Class A
48	66	M	27.18775	0	1	0	0	1	0	0	1	1	1	1	0	0	1	1	0	0	0	126	116	68	66	2.65	3.06	20.07066	17.07415	10.1	11.4	3.8	3.8	47	61	283	193	17.02899	21.18056	3640	1250	17100	7990	0.20339	0.16216	5.8	Class A
49	82	F	22.44898	0	0	0	1	1	0	0	0	0	0	0	0	0	1	1	0	0	0	130	138	73	72	1.44	1.4	27.1602	28.01028	10.2	12.2	4.3	4	93	86	63.8	29.7	22.09026	24.71264	680	314	279	221	2.14644	2.85291	NA	Class A
50	55	F	28.7649	1	0	0	0	1	0	0	0	1	0	0	0	0	1	1	0	0	0	129	128	80	76	2.13	2.24	19.84791	18.78412	9.3	10.2	3.7	3.7	59	68	177	156	19.15584	21.51899	2110	2050	126	86	2.56046	2.67735	9	Class A
51	68	M	28.12645	0	0	1	0	0	1	0	0	0	0	0	1	0	1	1	0	0	0	126	146	80	81	2.78	1.98	18.88368	27.37285	9.4	10.6	4.3	4.5	76	100	226	173	29.23077	36.10108	662	726	800	1070	0.54382	1.07987	NA	Class A
52	80	M	27.77032	0	0	0	1	1	0	0	0	1	1	0	0	0	1	1	0	0	1	111	143	52	70	1.36	1.24	39.40246	43.43734	10.4	12.4	4.1	4	60	84	9.9	42	16.57459	29.47368	2320	1070	269	329	0.06461	0.09379	6.1	Class A
53	60	M	24.9619	0	0	1	0	1	0	0	0	0	0	0	0	0	1	1	0	1	1	110	131	70	79	5	5.72	10.2991	8.8475	9.2	11.9	3	3	76	80	102	97.7	37.62376	37.38318	50	77	393	332	6.53061	7.81984	NA	Class B
54	69	M	24.91077	0	0	1	0	1	0	0	0	0	0	0	1	0	1	0	1	0	0	133	137	81	92	4.96	5.79	9.98148	8.42717	10.1	12.8	4	4	85	110	43.4	58	21.8509	29.49062	253	212	9120	12700	2.25	1.21669	6.4	Class B
55	67	M	NA	1	0	0	0	0	0	1	0	1	0	0	0	0	1	1	0	0	1	120	129	60	57	4.31	5.13	11.73816	9.70175	9.6	9.2	3.5	3.6	102	NA	96.6	NA	37.63838	NA	416	NA	402	NA	5.32143	4.09773	6.8	Class B
56	94	M	18.94108	0	1	0	0	0	1	0	0	0	0	1	0	0	0	0	0	0	0	120	119	52	61	2.1	2.08	23.3887	23.56317	11.3	12.1	4.1	4.2	62	68	168	167	26.95652	27.41935	6010	7530	536	605	0.22059	0.18904	6	Class A
57	78	M	25.39063	0	0	0	1	1	0	0	1	0	0	0	0	0	1	1	0	1	1	142	170	72	92	2.66	2.46	18.98289	20.67758	10.8	11.3	3.6	3.3	97	88	191	146	33.44828	38.76652	648	1170	190	756	2.62195	4.91111	NA	Class B
58	71	M	25.29263	0	1	0	0	0	1	0	1	0	0	0	0	0	0	0	0	0	0	149	157	79	73	3.68	4.27	13.72313	11.66279	9.4	10.4	3.34	3.68	52	61	53.1	32.1	15.61562	15.17413	4120	1800	658	609	4.56606	2.3602	NA	Class A
59	84	M	22.29359	1	0	0	0	1	0	0	1	1	0	1	0	0	1	1	0	1	1	143	130	47	51	2.09	1.82	24.28251	28.15402	8.9	10	4.1	3.8	52	47	337	364	23.52941	21.65899	3950	4140	2050	1510	0.22556	0.15408	5.4	Class B
60	69	M	23.9076	0	1	0	0	1	0	0	0	1	1	0	0	0	1	1	0	0	0	134	127	69	63	4.92	5.27	10.0703	9.30246	9.8	10.8	3	3	60	75	209	172	24.89627	33.18584	724	845	8200	7860	8.95238	10.85106	NA	Class C
61	66	F	24.74745	0	0	1	0	1	0	0	1	0	0	0	0	0	1	1	0	0	0	158	119	87	76	1.7	1.74	24.10604	23.50045	10.6	12.6	4.3	4.2	70	83	152	79.4	24.22145	27.94613	756	1170	236	197	1.28031	1.18774	5.9	Class A
62	77	F	24.22269	0	0	0	1	1	0	0	1	0	0	0	0	0	1	1	0	0	0	160	149	96	74	4.25	6.54	8.46381	5.28179	9.7	10.5	3.2	3.4	91	82	156	143	36.25498	34.0249	109	984	784	1370	7.40695	12.7907	NA	Class A
63	79	F	29.65681	0	0	1	0	0	1	0	0	0	0	0	1	1	1	0	0	0	1	126	135	66	82	0.79	0.72	52.94478	58.60106	9.6	11.7	4	3.9	53	55	9	14.2	13.73057	15.23546	5750	7060	76	53	0.0237	0.2231	6.2	Class A
64	49	F	20.8949	0	0	0	1	0	0	1	0	0	0	0	0	0	0	0	0	1	1	136	139	85	85	3.09	3.13	13.65669	13.46587	9.6	11.4	3.3	3.1	83	107	61.1	51.8	30.62731	42.46032	3290	4210	216	920	2.19307	1.75042	NA	Class A
65	25	M	20.40428	0	0	1	0	1	0	0	0	0	0	0	0	0	0	0	0	0	0	114	118	64	72	4.59	3.98	14.54018	16.80476	10.2	12.7	4.7	4.5	76	105	87.2	13.5	24.51613	28.61035	895	1170	29	45	1.8429	2.39394	NA	Class A
66	74	F	20.9752	0	0	0	1	1	0	0	1	0	0	0	0	0	0	0	0	0	0	142	142	67	77	2.92	3.96	12.90767	9.24907	9.2	10.4	4	3.9	90	116	95.5	79.9	30.82192	40.55944	53	50	590	390	4.375	2.89326	NA	Class B
67	67	F	16.72793	0	0	1	0	1	0	0	1	0	0	0	0	0	0	0	0	0	0	147	152	90	80	3.5	4	10.89312	9.37265	10.4	11	3.8	3.7	45	100	92.3	88.8	18.90756	38.61004	893	50	701	556	2.63462	3.58242	NA	Class B
68	65	M	26.67276	0	1	0	0	0	1	0	0	1	0	0	0	0	0	0	0	0	0	128	129	59	65	1.73	2.07	32.00149	26.2979	9.9	11.5	4.1	4.3	62	58	382	362	20.87542	19.14191	1400	2000	173	92	0.23377	0.20321	6.3	Class A
69	73	M	31.49645	0	0	0	1	0	1	0	1	0	1	0	1	0	1	1	0	0	0	142	168	91	97	3.2	3.49	15.86332	14.42703	9.7	11.3	4	4.2	70	66	135	129	28.92562	25.5814	1170	755	382	321	3.09091	NA	NA	Class C
70	74	M	23.98438	1	0	0	0	1	0	0	1	1	1	0	0	1	1	1	0	0	1	122	131	66	65	1.9	NA	27.94961	NA	10.7	NA	3.9	NA	88	60	160	92.8	32.59259	24.2915	429	653	1170	621	2.5222	NA	6.2	Class A
71	66	M	30.59935	0	1	0	0	0	1	0	0	0	0	0	0	0	1	1	0	0	0	128	124	80	72	6.63	6.24	7.35964	7.86431	10.1	12	3.8	3.7	92	132	327	277	33.33333	50	860	537	896	906	2.20599	2.9136	NA	Class A
72	87	F	21.09629	0	0	0	1	1	0	0	0	0	1	0	0	0	0	0	0	0	0	104	158	55	65	1.66	NA	22.8563	NA	11.7	NA	4.1	NA	90	NA	31.5	NA	30.50847	NA	221	NA	224	NA	0.11236	NA	NA	Class A
73	60	M	22.33242	0	0	0	1	0	1	0	0	0	1	0	1	0	1	1	0	0	0	124	117	86	82	2.91	2.94	18.61973	18.41197	9.1	8.7	3	2.8	85	72	94.6	131	31.83521	29.26829	267	1030	352	407	5.22897	4.06181	NA	Class C
74	75	M	23.04688	1	0	0	0	0	1	0	0	1	0	0	0	0	1	1	0	0	0	134	137	70	71	1.77	2.05	30.08683	25.62126	9.7	11.3	3.8	4.2	96	95	227	173	37.94466	34.92647	1680	155	191	207	4.60904	5.57596	5.8	Class A
75	59	M	16.91752	1	0	0	0	1	0	0	1	0	1	0	0	0	1	0	1	0	0	118	145	64	88	3.66	NA	14.48852	NA	8.6	NA	3.4	NA	76	NA	64.4	NA	26.95035	NA	199	NA	11000	NA	4.51811	NA	5.5	Class B
76	71	M	17.75148	1	0	0	0	1	0	0	1	1	1	1	0	0	1	1	0	0	0	142	135	72	65	3.49	5.34	14.54252	9.13189	7.8	9.9	2.4	2.8	NA	NA	NA	NA	NA	NA	NA	NA	NA	NA	7.64894	9.27778	6.3	Class C
77	66	F	20.77221	0	0	1	0	1	0	0	1	0	0	0	0	0	0	0	0	0	0	167	NA	82	NA	2.27	2.23	17.56891	17.91396	10.3	10.1	4.2	3.9	78	53	31.2	17.7	21.4876	13.76623	1670	504	183	109	0.5	0.71875	5.8	Class A
78	78	F	19.37778	0	0	1	0	1	0	0	0	0	0	0	0	0	1	1	0	0	0	120	166	74	105	5.3	NA	6.62304	NA	10.6	NA	4.2	NA	99	95	59.2	69.6	37.5	36.53846	333	600	388	646	0.85075	NA	NA	Class C
79	92	F	24.77961	0	0	1	0	1	0	0	0	0	0	0	0	0	1	1	0	0	1	119	112	55	60	1.38	1.43	27.44504	26.39696	10.2	11.3	3.7	4.2	73	70	63.7	52.7	27.54717	22.4359	413	2090	106	152	0.12132	0.12467	5.6	Class A
80	44	F	NA	0	0	1	0	1	0	0	0	0	0	0	0	0	0	0	0	0	1	NA	NA	NA	NA	1.1	NA	43.60052	NA	11	NA	3.7	NA	NA	NA	NA	NA	NA	NA	NA	NA	NA	NA	0.30597	NA	5.8	Class A
81	83	F	18.26667	0	0	1	0	1	0	0	1	1	1	1	0	0	0	0	0	1	1	198	143	60	55	1.51	1.58	25.69626	24.45344	7.9	9.1	3.1	3.4	35	52	46.1	71.1	14.83051	20.63492	4360	1300	6820	4090	1.35458	1.51724	7.9	Class A
82	75	M	25.08662	0	1	0	0	0	0	0	1	0	0	0	0	0	1	1	0	0	0	184	163	91	82	2.53	2.66	20.35379	19.26809	10.9	11.6	3.3	3.2	58	60	97.3	59.6	24.26778	26.54867	1010	190	564	422	1.5035	0.53589	5.9	Class B
83	83	F	20.69844	0	0	1	0	0	1	0	1	0	1	0	0	1	0	0	0	0	0	146	147	71	60	2.86	3.39	12.77634	10.60797	9.5	10.9	3.4	3.4	83	95	105	121	39.52381	44.18605	1620	654	990	750	2.3535	3.13852	NA	Class A
84	78	F	NA	0	0	0	1	1	0	0	0	0	0	0	1	0	1	1	0	0	0	NA	124	NA	80	1.76	1.26	22.12199	31.8867	9.6	10.9	4.1	4.1	66	86	69.1	45.3	24	28.28947	95	718	585	543	NA	0.39808	5.3	Class A
85	84	M	19.9549	0	0	0	1	0	1	0	1	1	0	0	1	0	1	1	0	0	1	152	123	57	50	2.92	3.02	16.84243	16.23327	10.4	10.8	3.7	3.7	32	19	11.9	41.2	9.384164	6.643357	6410	46900	1250	1360	2.72727	NA	6	Class A
86	66	F	25.33333	0	0	1	0	1	0	0	0	0	0	0	0	0	1	1	0	1	1	125	139	71	82	2.52	2.59	15.67129	15.20853	10.1	12	3.8	4	82	128	77.9	48.4	30.14706	45.5516	969	446	364	202	0.46154	0.32353	NA	Class A
87	75	M	24.16221	1	0	0	0	1	0	0	1	0	0	1	1	1	1	1	0	1	1	126	132	72	71	2.31	2.63	22.39839	19.43465	10.2	11.3	3.3	3.4	58	80	42.6	81.6	23.57724	34.33476	183	147	212	138	1.88482	NA	5.7	Class A
88	57	F	17.97332	0	0	1	0	1	0	0	1	0	0	0	0	0	0	0	0	0	0	168	161	75	72	2.96	3.12	13.7062	12.93913	9.8	9.1	3.8	3.6	76	82	111	99.4	26.48084	29.28571	1730	1150	1270	1730	11.51843	4.42434	NA	Class C
89	71	M	27.30469	1	0	0	0	0	0	1	0	1	1	0	0	0	1	1	0	1	1	122	138	58	70	3.22	3.12	15.88167	16.43938	9.6	11.3	3.4	3.8	79	122	50.3	61.2	26.68919	39.86928	315	534	287	165	0.85963	1.52597	5.8	Class A
90	85	F	21.11471	0	0	1	0	1	0	0	1	1	1	1	0	1	0	0	0	1	1	181	139	72	48	1.18	1.26	33.42443	31.10982	10.4	12.1	2.8	3.8	72	84	536	544	41.14286	40.97561	2360	977	6820	4270	12.21918	8.71134	7.9	Class B
91	48	F	30.45766	1	0	0	0	0	1	0	1	1	0	0	0	0	1	1	0	0	1	166	168	79	81	1.09	0.99	42.95219	47.7205	10.3	9.4	2.9	2.8	NA	88	NA	36.4	NA	40.93023	NA	454	NA	99	5.613	4.88753	6.1	Class A
92	65	F	20.94478	0	0	1	0	1	0	0	0	0	0	0	1	1	1	0	0	0	0	135	145	50	46	1.03	1.14	41.88863	37.32357	8.7	9.4	4.2	4	67	91	551	463	31.90476	43.75	549	264	1360	1360	0.76923	0.001724	NA	Class A
93	77	F	20.04414	0	1	0	0	1	0	0	1	0	0	0	1	0	1	1	0	0	0	154	120	55	53	1.24	1.34	32.57017	29.92063	9.8	10.8	3.8	3.9	114	88	50.9	41.2	43.01887	32	347	507	102	120	0.29412	0.13483	5.9	Class A
94	74	F	25.4094	1	0	0	0	0	1	0	1	1	0	0	0	1	1	1	0	1	1	171	131	98	68	1.62	1.49	24.59049	26.94703	10.3	11.6	3.7	3.5	58	70	190	135	21.56134	29.53586	1310	583	350	494	0.6015	0.34843	6.9	Class A
95	79	M	19.40809	0	0	0	1	1	0	0	0	0	0	0	0	0	0	0	0	0	0	130	137	66	77	3.93	4.38	12.38561	11.00044	9.3	10.7	3.9	3.6	99	129	360	277	40.08097	53.9749	88	115	1520	1290	1.75115	2.41316	NA	Class C
96	42	F	19.97919	0	0	0	1	0	1	0	0	0	0	0	0	0	1	1	0	0	0	137	128	85	78	2.76	2.98	16.15173	14.85187	10.7	12.2	4.2	4	90	94	42.8	100	32.3741	39.4958	818	449	417	506	1.67055	0.76503	5.2	Class A
97	69	F	NA	0	1	0	0	0	1	0	1	0	0	0	0	0	0	0	0	0	0	144	123	83	71	1.08	0.98	39.09587	43.30138	10.1	11.7	4	3.8	69	73	59.3	23.1	25.55556	25.52448	179	157	263	171	0.06126	NA	NA	Class A
98	75	F	22.46999	0	0	0	1	0	0	0	0	0	0	0	0	0	0	0	0	0	0	130	NA	62	NA	3.68	3.73	9.98311	9.83681	10.5	11.2	3.8	3.4	96	NA	105	NA	37.35409	NA	158	NA	953	NA	1.52899	NA	NA	Class A
99	58	M	25.6732	1	0	0	0	0	1	0	1	1	0	1	0	1	1	0	0	0	0	137	136	75	74	2.08	2.54	27.14792	21.81774	8.8	10.4	2.7	3.4	102	123	555	490	43.03797	50	85	154	384	297	8.86207	9.36667	6.5	Class B
100	72	F	27.45136	0	1	0	0	0	1	0	1	0	0	0	0	0	0	0	0	0	0	167	136	68	77	1.59	1.55	25.29667	26.01171	10.7	11.3	4.05	3.71	72	131	60.4	47.8	19.61853	39.45783	566	830	302	242	1.59434	5.17007	NA	Class A
101	52	F	25.88574	0	0	1	0	0	0	1	0	1	0	0	0	0	0	0	0	0	0	NA	NA	NA	NA	0.75	NA	63.18823	NA	10.7	NA	3.8	NA	19	NA	82.5	NA	7.19697	NA	38700	NA	79	NA	0.13924	NA	5.2	Class A
102	71	M	23.31672	1	0	0	0	0	1	0	0	1	0	0	0	0	1	1	0	0	0	119	123	58	59	4.03	5.86	12.42473	8.21613	9.1	11.6	3.5	3.2	56	38	110	55.5	23.04527	15.01976	5750	2050	834	1030	2.8125	7.04885	5.7	Class A
103	68	M	17.03246	0	1	0	0	0	0	0	1	1	1	0	0	0	1	1	0	0	0	100	98	60	67	1.93	1.97	28.14958	27.40981	10.2	11.7	3.1	3.9	NA	57	NA	373	NA	26.14679	NA	2870	NA	5830	0.33333	0.10357	5.7	Class A
104	58	M	NA	0	0	0	1	0	0	0	1	1	1	0	0	0	1	1	0	0	0	160	152	100	96	7.22	7.28	6.92353	6.86113	10.1	9.9	3.6	3.6	63	109	25.6	19.3	23.77358	38.79004	575	807	1370	3000	3.52381	3.03636	NA	Class A
105	41	M	22.29568	0	0	0	1	1	0	0	0	0	0	0	0	0	1	1	0	0	0	127	102	67	69	2.8	2.77	21.66416	21.92098	10.6	12.6	4	4.2	82	106	62.1	43.3	31.66023	38.97059	905	533	41	47	0.24823	0.45415	NA	Class A
106	62	F	20.85534	1	0	0	0	0	0	1	0	1	0	0	0	0	1	1	0	0	0	117	127	62	80	4.04	4.3	9.52025	8.89232	9.7	11	3.3	3.4	57	NA	98	NA	25.11013	NA	585	NA	835	NA	5.8885	11.55738	7.5	Class B
107	30	F	25.177	1	0	0	0	0	0	1	0	1	0	0	0	0	0	0	0	1	1	96	84	63	59	3.18	4.38	15.23551	10.73346	9.4	10.7	2	2.3	45	43	20.4	65.1	16.18705	17.47967	399	2670	384	367	10.60563	12.05085	11.9	Class B
108	87	F	20.19894	1	0	0	0	0	0	1	1	0	0	0	0	0	1	1	0	0	0	150	200	70	80	2.87	3.05	12.55687	11.74844	10	10.3	3.8	3.3	74	38	37.7	99.1	27.0073	17.04036	231	48300	685	1740	3.90121	6.31579	5.4	Class B
109	77	M	28.57796	0	1	0	0	0	1	0	0	0	0	0	1	0	1	0	1	0	0	111	126	62	72	4.86	5.54	9.89006	8.56997	8.9	9.8	4	4	73	113	294	280	28.85375	42.80303	984	504	554	425	1.50435	3.83077	5.5	Class C
110	78	F	NA	0	0	1	0	1	0	0	1	0	0	0	0	0	1	0	1	0	0	115	NA	63	NA	2.11	NA	18.14053	NA	10	NA	3.7	NA	39	54	14.3	49	14.8289	21.01167	32400	42100	851	405	NA	NA	7	Class A
111	71	F	26.27135	0	0	0	1	0	1	0	1	1	0	0	0	0	1	1	0	1	1	149	130	68	77	2.18	2.14	17.98306	18.35111	9.3	11.6	4.5	4.6	88	123	60.6	49.6	29.23588	39.80583	486	94	169	115	0.60594	0.49589	6.4	Class A
112	78	F	NA	1	0	0	0	0	0	0	1	1	0	0	0	1	1	0	1	0	0	163	148	85	75	1.33	1.21	30.05532	33.33096	8.9	10.2	3.2	2.9	59	107	139	88.1	22.60536	40.99617	3290	541	282	831	5.625	8.38986	6	Class A
113	84	M	20.95717	0	1	0	0	1	0	0	0	0	1	0	0	0	1	1	0	0	0	120	122	73	74	3.51	3.74	13.77107	12.84731	8.6	10	3.6	3.6	61	69	47.9	17.5	21.63121	20.35398	427	489	2050	2150	0.86729	1.16352	5.2	Class B
114	59	F	18.98657	0	0	1	0	1	0	0	1	0	0	0	0	0	1	0	1	1	1	140	121	90	95	5.33	6.07	7.13136	6.1859	8.8	10.3	3.6	3.8	67	88	93.5	97.9	26.8	32.11679	829	794	1430	976	0.97826	1.05128	NA	Class A
115	67	M	17.15626	0	0	1	0	1	0	0	0	0	0	0	0	0	1	1	0	0	0	120	120	60	60	4.17	4.3	12.16997	11.76803	9.8	11.7	3.9	4	86	100	60.8	48.6	30.82437	32.25806	262	696	432	452	1.36047	1.15493	NA	Class A
116	67	M	22.1765	0	0	0	1	0	1	0	0	0	0	0	0	0	1	1	0	0	1	129	118	79	72	2.17	2.51	24.86745	21.11682	9.4	10.1	3.4	3.3	39	60	55.4	60.5	15.35433	25.42373	151	134	83	163	1.83696	3.6069	5.8	Class A
117	91	M	22.50693	0	0	1	0	1	0	0	0	0	0	0	1	0	0	0	0	1	1	124	132	54	64	1.48	NA	34.61709	NA	9.4	NA	4.2	NA	87	101	48.6	89.2	27.70701	38.69732	265	208	781	394	NA	NA	5.5	Class A
118	84	F	19.99978	0	1	0	0	1	0	0	0	0	0	0	0	0	0	0	0	0	0	126	115	59	54	1.41	1.19	27.60148	33.22995	7.6	10	4	4.2	97	100	347	208	28.44575	30.8642	99	493	786	421	1.49239	2.29274	NA	Class A
119	35	F	22.71897	0	0	1	0	1	0	0	1	0	0	0	0	0	1	1	0	0	0	140	133	80	70	2.04	2.17	23.6899	22.14174	10.2	10.9	3.7	3.9	69	NA	26.4	NA	24.38163	NA	404	NA	129	NA	0.4	0.4	NA	Class A
120	79	M	NA	1	0	0	0	0	0	0	0	1	1	0	0	0	1	1	0	0	0	138	NA	70	NA	2.59	NA	19.54487	NA	9.2	NA	NA	NA	59	46	139	45.3	33.71429	26.90058	2180	344	5760	1080	0.54167	NA	5.8	Class A
121	78	M	19.70553	0	0	1	0	1	0	0	1	0	0	0	1	0	1	1	1	1	1	156	NA	69	NA	1.72	NA	30.69746	NA	10.2	NA	3.76	NA	42	NA	214	NA	19.35484	NA	25400	NA	264	NA	1.07792	NA	NA	Class A
122	84	M	21.24837	0	1	0	0	0	1	0	0	0	1	0	0	0	1	1	0	0	0	112	110	71	69	1.37	1.24	38.5444	42.98632	10.3	11.6	4.2	4	68	88	72.6	34.9	25.46816	30.98592	660	414	391	397	0.06508	0.0638	5.7	Class A
123	74	M	22.26563	0	1	0	0	0	1	0	1	0	0	0	1	0	1	1	0	0	0	141	148	75	78	3.03	3.27	16.7739	15.43181	10.6	11.6	3.7	3.4	79	81	16.8	16.8	26.24585	27	180	1420	270	101	2.47772	2.37785	5.9	Class A
124	89	M	25.47347	0	0	0	1	0	0	1	1	0	0	0	0	0	0	0	0	0	0	164	NA	58	NA	4.84	8.6	9.53029	5.08142	9	5.31	3.1	2.8	63	23	182	161	26.92308	13.21839	50	4410	2040	5270	5.64198	NA	NA	Class A
125	77	M	26.94384	0	1	0	0	0	1	0	1	1	0	0	0	0	0	0	0	1	1	147	144	63	70	3.5	4.7	14.16343	10.22105	10.3	11.6	3.8	3.4	101	121	186	180	37.68657	47.26563	2120	1370	785	818	1.01156	3.44988	6.4	Class A
126	79	M	20.70082	0	1	0	0	0	1	0	0	0	1	0	0	0	1	1	0	0	0	104	142	46	81	2.08	1.41	24.844	37.87644	9.1	10.6	4.4	4.6	99	71	64.4	22.3	26.32979	16.24714	367	323	1870	984	0.02564	0.03226	5.6	Class A
127	67	F	18.92985	0	0	1	0	1	0	0	0	0	0	0	0	0	1	1	0	0	0	126	118	80	68	1.4	1.37	29.68237	30.39418	8.9	9.9	3.6	3.7	44	54	136	55	18.64407	18.94737	16500	976	458	132	0.16949	0.15369	NA	Class A
128	61	M	20.66464	0	0	0	1	0	1	0	0	0	0	0	0	0	1	1	0	0	0	125	119	71	71	3.95	3.78	13.26584	13.9199	10.1	11.6	3.96	3.93	66	79	144	105	23.65591	28.72727	83	245	422	310	0.40745	1.12645	NA	Class A
129	49	M	25.65437	0	0	1	0	0	1	0	0	0	0	0	0	0	1	1	0	0	0	NA	NA	NA	NA	5.7	NA	9.40308	NA	10	NA	4.4	NA	67	NA	110	NA	23.34495	NA	6500	NA	1160	NA	1.80822	NA	5.8	Class A
130	67	F	20.45033	0	0	1	0	1	0	0	1	0	0	0	0	0	1	0	1	0	0	169	156	89	77	2.89	3.05	13.43199	12.66306	9.9	11.7	4	4.4	60	42	60.9	35.9	21.05263	12.38938	783	292	149	100	1.03901	0.7596	NA	Class A
131	72	M	21.86395	0	0	1	0	1	0	0	0	0	0	0	0	0	1	1	0	0	0	108	134	78	71	3.54	3.25	14.2606	15.59652	11.1	11.9	4.1	3.7	84	113	180	76.1	28.57143	38.04714	1450	562	1220	1450	0.70673	2.41573	5.3	Class A
132	85	M	21.42014	0	1	0	0	0	0	0	1	1	0	0	0	0	0	0	0	0	0	150	140	80	80	2.6	2.85	19.05801	17.23686	9.4	10.8	4.2	4	51	87	180	139	21.25	34.11765	112	72	942	1240	2.825	2.98	NA	Class C
133	68	F	19.34996	0	0	0	1	0	1	0	0	1	0	0	0	0	0	0	0	0	1	129	135	60	69	2.46	3.41	15.95266	11.16048	8.9	8	3.3	2.1	45	59	24.8	15.2	16.79104	38.31169	18200	11700	1990	2760	0.41429	0.94118	4.6	Class C
134	65	M	23.14529	0	0	0	1	0	1	0	0	0	0	0	0	0	1	1	0	0	0	134	128	85	92	3.02	3.44	17.39663	15.08682	10.3	11.6	4.53	4.07	114	121	77.6	82.2	33.33333	34.18079	600	784	86	65	0.94589	1.61706	NA	Class A
135	31	F	34.91746	0	0	0	1	1	0	0	1	0	0	0	0	0	1	1	0	1	1	179	140	100	90	5.31	NA	8.61335	NA	8.7	NA	3.7	NA	39	NA	14	NA	13.68421	NA	2910	NA	204	NA	2.74753	NA	NA	Class B
136	68	M	20.84481	0	0	0	1	1	0	0	1	0	0	0	0	0	0	0	0	0	0	152	159	82	90	3.4	3.99	15.15074	12.71766	10	10.3	3.5	3.4	104	74	41.5	55.2	39.09774	27.50929	147	1180	199	128	1.31608	1.43593	5.1	Class B
137	67	F	20.40449	0	0	0	1	1	0	0	0	0	0	0	0	0	1	1	0	0	0	121	113	68	66	1.11	1	38.26303	42.70868	9.4	10.7	3.6	3.9	22	83	68	39	7.971014	26.01881	23300	882	148	57	1.30016	1	NA	Class A
138	64	F	NA	0	1	0	0	0	1	0	0	0	0	0	0	0	0	0	0	0	0	96	112	52	66	2.1	2.25	19.30032	17.89719	8.4	11.2	3.6	4.4	42	NA	73.3	NA	17.35537	NA	5780	NA	275	NA	0.03861	0.02837	6.1	Class A
139	65	F	18.04444	1	0	0	0	0	1	0	0	1	0	0	0	0	1	1	1	0	0	137	140	67	70	1.92	2.15	21.19378	18.72632	9.5	11.3	3	2.5	63	94	65.8	39.5	25.92593	40.69264	90	121	516	738	12.61111	15.31579	7.2	Class A
140	70	M	22.62035	0	0	0	1	0	1	0	1	0	0	0	0	0	0	0	0	0	0	125	128	73	72	1.41	1.43	39.35618	38.7544	9.4	11.1	3.8	3.9	80	125	66.3	58.9	21.97802	33.96739	89	212	199	192	1.55414	2.25933	NA	Class A
141	68	M	21.90561	0	0	1	0	0	1	0	1	0	0	0	0	0	1	0	1	0	0	133	138	96	75	2.98	2.98	17.50165	17.50165	10.8	11.2	3.6	3.7	55	75	262	221	21.56863	29.29688	1370	976	366	622	1.01081	0.92467	5.9	Class A
142	69	F	23.33231	0	0	0	1	0	0	1	0	1	0	0	0	0	0	0	0	0	1	118	122	70	74	2.58	2.87	15.07942	13.42065	10.3	12.7	3.9	3.8	68	62	48.9	43.6	24.81752	21.75439	199	734	367	499	4.83495	8.30508	7.3	Class A
143	70	F	19.47341	0	0	0	1	1	0	0	1	0	0	0	0	0	0	0	0	0	0	148	120	83	73	3.25	3.25	11.66548	11.61809	8.8	9.3	3.8	3.8	36	97	90.7	24	13.18681	29.84615	9000	374	718	631	1.56923	1.88136	NA	Class A
144	74	M	19.97823	0	0	1	0	1	0	0	0	0	0	0	1	0	1	1	1	0	0	137	134	80	71	2.02	2.02	26.13833	26.13833	10	11.2	4.5	4.8	NA	NA	NA	NA	NA	NA	NA	NA	NA	NA	0.26217	NA		Class A
145	73	F	25.68371	1	0	0	0	0	1	0	0	1	1	0	0	1	0	1	0	1	1	120	149	60	66	1.25	0.98	32.78332	42.78299	10.4	11	3.5	3.8	56	69	49.4	71.7	21.29278	24.82014	2190	901	2570	232	0.25899	0.19651	6	Class A
146	70	M	19.62323	0	0	0	1	0	1	0	1	0	0	0	0	0	0	0	0	0	0	148	127	93	76	1.58	1.26	34.74783	44.50955	10.5	10.9	4	4.2	52	48	145	105	23.42342	22.11982	263	468	494	365	0.19238	1.11825	NA	Class A
147	81	M	21.99219	0	1	0	0	0	1	0	0	1	0	0	0	0	1	1	0	0	1	118	138	52	84	2.43	2.13	20.80718	24.03362	9.3	11.8	3.8	4.3	56	68	57.4	66.7	20.81784	22.66667	360	1300	61	183	0.84615	2.31633	6.8	Class A
148	88	F	16.24777	0	0	1	0	1	0	0	0	0	0	0	0	0	0	0	0	0	0	130	140	70	80	1.89	1.76	19.76653	21.30004	10.7	11.8	4.3	4.5	59	67	210	171	28.78049	32.21154	270	213	465	407	0.16	0.15	NA	Class A
149	77	M	29.99406	1	0	0	0	0	1	0	0	1	0	0	0	0	0	0	0	0	0	126	NA	67	NA	5.63	NA	8.42021	NA	10.3	NA	3.5	NA	89	101	68.2	50.3	38.69565	44.69027	85	160	650	529	0.48513	NA	6.4	Class A
150	75	M	20.59495	0	1	0	0	0	1	0	1	0	0	0	0	0	0	0	0	1	1	186	162	89	90	3.92	3.46	12.60678	14.4514	8.8	11.4	2.3	2.8	89	118	60.1	67.5	36.32653	54.12844	110	230	1900	1350	1.63652	0.75567	NA	Class B
151	73	F	20.56982	0	1	0	0	1	0	0	1	0	0	0	0	0	0	0	0	1	1	154	159	77	84	2.52	2.68	15.2244	14.17742	9.8	10.8	4	3.9	66	103	125	143	24.35424	38.28996	434	464	699	525	1.89091	2.03226	5.5	Class A
152	66	M	20.57036	0	0	0	1	1	0	0	0	0	0	0	0	0	1	1	0	0	0	133	124	78	75	2.69	2.76	19.74439	19.19721	11.3	12	3.8	3.8	52	49	65	51.3	18.37456	16.83849	112	96	248	170	0.46667	0.35714	NA	Class A
153	73	F	NA	0	1	0	0	1	0	0	0	0	1	0	1	0	1	1	0	0	0	NA	115	NA	72	1.5	1.71	26.85522	23.17816	11.1	11	3.9	3.4	116	105	75.9	54.1	46.4	46.46018	748	708	231	187	0.20225	0.0625	5.8	Class A
154	79	F	23.61424	0	0	1	0	0	1	0	0	0	0	0	0	0	1	1	0	0	0	110	116	50	58	2.6	2.8	14.3829	16.60529	10.1	12.8	3.8	4	91	88	162	83	30.33333	26.11276	497	458	519	369	0.22512	0.40435	5.7	Class A
155	72	M	27.85201	0	0	1	0	0	1	0	0	0	0	0	0	0	1	1	0	0	0	119	117	75	70	1.99	1.99	26.77947	26.77947	10.6	10.2	3.3	3.2	30	26	67.5	88.5	10.71429	10.03861	30700	52200	60	89	NA	0.53846	6.1	Class A
156	68	M	24.27012	0	0	1	0	0	0	1	0	0	0	0	0	1	1	0	1	0	0	128	137	76	80	3.55	3.87	14.4518	13.09472	11.1	11.4	4.3	4.2	91	97	43	48.5	28.17337	29.57317	2280	3860	120	116	0.72263	1.90731	NA	Class A
157	59	F	16.72483	1	0	0	0	0	1	0	0	1	1	0	0	0	1	1	0	1	1	146	131	72	60	1.01	0.92	44.00318	48.73352	8.1	12.3	3.6	3.6	36	67	60.7	60.6	14.45783	30.45455	111	53	866	313	5.45536	5.04141	5.7	Class A
158	95	M	NA	0	0	1	0	1	0	0	0	0	1	0	0	0	0	0	0	0	0	135	160	61	80	2.2	2.2	22.16076	22.16076	9.3	11.2	3.6	3.9	57	79	43.8	6.1	26.88679	26.77966	4770	1040	501	393	0.22044	0.29455	NA	Class A
159	89	M	19.37512	0	1	0	0	0	1	0	1	1	0	0	0	0	1	1	0	0	0	110	106	62	57	1.69	1.46	30.13136	35.24781	9.9	10.1	3.8	3.7	41	76	36.1	26.7	14.69534	25.93857	941	1490	1380	1450	0.10147	0.13793	6.8	Class A
160	70	M	21.08135	0	1	0	0	0	1	0	1	0	0	1	1	0	0	0	0	0	1	144	126	74	68	2.74	2.75	19.02653	18.95085	10.7	12.9	4.6	4.9	50	71	27.4	67.4	15.29052	21.64634	229	578	575	326	0.10753	0.072	5.4	Class A
161	74	M	22.58955	0	1	0	0	0	1	0	0	0	0	0	0	0	1	1	0	0	0	120	110	60	60	2.86	3.39	17.86766	14.77817	10.9	11.2	4.4	4.1	74	155	402	399	18.6398	37.80488	1880	180	734	624	2.47143	1.1625	NA	Class A
162	77	M	25.59993	0	1	0	0	0	1	0	0	0	0	0	1	1	1	0	1	1	1	118	126	75	61	4.28	NA	11.36526	NA	8.2	NA	3.3	NA	14	NA	6.8	NA	3.804348	NA	1720	NA	7060	NA	NA	NA	5.9	Class B
163	78	M	19.10457	1	0	0	0	0	1	0	1	1	0	0	0	1	0	0	0	0	0	180	135	88	70	2.95	3.67	17.01317	13.39757	9.6	12.8	3.6	3.3	77	78	231	120	34.52915	28.57143	82	125	663	523	7.2454	12.25387	6.1	Class B
164	60	F	23.25714	1	0	0	0	1	0	0	1	1	0	0	0	0	1	1	0	0	0	161	170	79	78	2.93	3.38	13.65726	11.68105	8.5	10.6	4.1	4.7	63	90	198	130	24.23077	27.52294	871	415	489	530	2.84778	5.06486	6.4	Class A
165	47	F	NA	0	0	1	0	1	0	0	0	0	0	0	0	0	0	0	0	0	1	134	125	83	80	2.42	2.7	18.0576	16.01925	9.7	12.6	3.9	4	35	69	3.6	13.4	7.261411	17.25	923	644	196	131	0.26471	NA	NA	Class A
166	57	F	NA	0	0	1	0	1	0	0	0	0	0	0	0	0	0	0	0	1	1	NA	NA	NA	NA	1.03	0.84	43.49769	54.36865	10	13.1	3	3.1	27	43	3.8	14.8	6.766917	13.35404	922	2870	71	52	0.07368	0.07463	NA	Class A
167	80	F	21.09685	0	0	0	1	1	0	0	0	1	1	0	0	0	1	1	0	0	0	136	144	76	66	2.51	1.81	14.89417	21.29899	9.2	9.6	3.6	3.6	53	58	25.8	9.5	17.32026	15.38462	656	582	190	246	1.12108	1.38313	5.6	Class A
168	73	M	24.17057	0	0	1	0	1	0	0	1	1	0	0	0	0	1	1	0	0	0	142	140	86	80	4.8	5.48	10.18006	8.77215	9.7	10.9	4	4	81	124	78	47.4	33.33333	45.25547	554	188	482	368	1.43422	1.26067	6.2	Class C
169	76	F	21.82853	0	0	1	0	1	0	0	0	0	0	0	0	0	1	1	0	0	0	127	140	65	67	0.9	0.77	46.42067	55.05944	9.4	11.3	4.5	4.3	67	113	653	233	18.0593	31.38889	11400	3190	628	378	0.33929	0.7561	NA	Class A
170	74	M	18.32264	0	1	0	0	0	1	0	0	0	0	0	0	0	0	0	0	0	0	120	134	70	71	1.4	1.4	39.03626	39.03626	10.4	12.1	4.2	3.6	137	77	577	470	47.07904	31.17409	190	23300	140	135	0.13836	0.14419	NA	Class A
171	81	M	19.94806	0	0	1	0	1	0	0	1	0	0	0	1	0	0	0	0	0	1	170	168	81	53	2.3	2.4	22.09715	21.09189	8.7	8.8	3.3	3	38	NA	14.6	NA	11.11111	NA	5590	NA	718	NA	3.31579	2.59143	NA	Class A
172	60	F	NA	0	0	1	0	0	0	0	0	0	0	0	0	0	0	NA	NA	NA	0	NA	NA	NA	NA	4.46	NA	8.62471	NA	11.5	NA	NA	NA	NA	NA	NA	NA	NA	NA	NA	NA	NA	NA	NA	NA	NA	Class A
173	79	M	31.24876	0	0	1	0	0	1	0	0	1	0	0	0	1	0	0	0	0	1	93	NA	63	NA	0.92	0.57	60.64554	102.3896	9.3	13.5	4.2	4.2	NA	NA	17.9	53.7	NA	NA	510	7050	708	964	0.32	0.78571	5.3	Class A
174	53	F	21.98694	0	0	1	0	1	0	0	0	0	0	0	0	0	1	1	0	0	0	124	134	76	84	2.13	2.02	19.95271	21.14437	10.5	11.2	4.6	4.5	89	90	80.1	108	28.70968	30.82192	822	651	69	53	NA	0.5	NA	Class A
175	86	M	25.70063	0	0	0	1	0	1	0	0	0	0	0	0	0	1	1	0	0	1	104	128	53	63	1.8	1.8	28.30685	28.30685	9.4	10.4	3.9	3.8	96	126	139	179	38.55422	51.63934	188	625	168	105	0.03828	0.09585	NA	Class B
176	71	M	21.404	1	0	0	0	0	1	0	1	1	0	0	0	1	0	0	0	1	1	157	142	53	48	1.7	1.55	31.94328	35.19852	10.8	11	3.6	3.7	71	63	149	185	29.46058	25.71429	3770	1920	134	127	0.57471	0.53885	6.4	Class A
177	41	F	21.1657	0	0	1	0	0	1	0	0	0	0	0	0	0	1	1	0	0	0	NA	NA	NA	NA	0.87	0.9	57.50997	55.03416	10.1	9.7	2.6	2.4	80	42	6	4.4	28.36879	17.72152	84	106	195	146	2.90541	4.55738	5.6	Class A
178	57	M	31.73327	0	0	1	0	1	0	0	1	1	0	0	0	0	1	1	0	1	1	164	154	88	98	3.85	6.36	13.91142	7.99311	9.4	12.5	3.2	4.1	65	NA	63	NA	30.23256	NA	694	NA	1200	NA	1.25714	7.84932	6.8	Class B
179	65	M	18.96856	0	0	0	1	0	1	0	0	0	0	0	0	0	1	1	0	0	1	125	118	78	74	3.62	3.49	14.33073	14.85052	10.4	11.9	3.8	3.6	67	87	40.8	70.8	26.5873	34.8	342	138	595	451	0.55357	0.56579	5.6	Class A
180	69	M	33.73061	0	0	0	1	0	0	1	0	1	0	0	0	0	0	0	0	0	0	138	NA	62	NA	2.6	3.07	20.23351	16.80077	10.4	10.4	2.5	2.4	NA	25	NA	43.7	NA	8.012821	NA	23400	NA	5890	13.14286	8.56604	NA	Class A
181	49	F	18.15479	0	0	0	1	1	0	0	0	0	0	0	0	0	1	1	0	0	1	112	113	79	77	1.5	1.7	30.11037	26.10542	10.6	13.6	NA	NA	90	121	208	193	39.47368	49.79424	133	84	128	157	1.52009	1.7624	NA	Class A
182	63	M	NA	0	0	1	0	1	0	0	0	0	0	0	0	0	0	0	0	0	0	132	130	86	78	6.25	6.03	7.95606	8.27416	10.3	11.2	4.5	4.3	141	123	46.8	42.8	41.22807	37.16012	52	70	195	197	2.38095	2.27273	NA	Class B
183	61	F	23.31962	0	0	1	0	0	1	0	1	0	0	0	0	0	0	0	0	0	0	118	116	70	74	4.71	NA	8.08671	NA	8.2	NA	4.2	NA	135	NA	627	NA	53.78486	NA	162	NA	2470	NA	2.57294	NA	NA	Class B
184	73	M	21.67636	0	0	0	1	0	1	0	0	0	0	1	0	1	1	1	0	0	0	133	156	66	63	3.99	4.03	12.46131	12.32606	10.6	10.3	3.4	3.5	51	70	56.8	39.6	19.61538	24.22145	2420	4310	195	819	2.3516	2.57023	5.8	Class C
185	59	M	25.1559	1	0	0	0	0	1	0	1	0	0	0	1	0	1	1	0	0	0	121	141	52	70	3.52	3.34	15.19321	16.09121	10.8	11.7	3.2	3.6	94	89	31.6	38.2	26.25698	24.79109	134	138	413	486	1.46618	1.76962	6.1	Class A
186	68	M	NA	0	0	0	1	0	0	1	0	1	0	0	0	0	0	0	0	0	0	NA	NA	NA	NA	3.6	3.65	14.23235	14.0192	9.7	10.6	3.7	4.1	43	61	57.9	39.2	15.19435	21.03448	2170	1210	223	182	0.75	0.47143	6	Class A
187	66	F	29.26824	0	0	1	0	0	1	0	1	0	0	0	0	0	1	1	0	0	1	119	126	55	66	2.22	2.03	18.00225	19.85347	11.1	11.1	4.4	4.2	78	102	56.8	87.9	23.85321	32.58786	1750	1690	669	472	0.04252	0.18634	6.6	Class A
188	62	F	22.35448	0	0	1	0	1	0	0	0	0	0	0	0	0	1	1	0	0	0	133	156	87	97	3.31	3.6	11.83962	10.80028	10.3	10.7	3.9	3.8	89	53	34.9	33.5	34.23077	18.79433	866	1310	1010	634	0.42857	1.04545	NA	Class A
189	79	F	21.37474	1	0	0	0	0	0	0	1	1	0	0	1	0	1	1	0	0	0	142	124	78	60	1.64	1.66	23.81157	23.41322	10	9.5	3.2	2.9	NA	60	NA	53.1	NA	26.20087	NA	365	NA	480	0.71942	0.67568	5.1	Class A
190	87	M	24.2079	1	0	0	0	0	1	0	0	1	0	0	0	1	0	0	0	0	0	137	136	69	68	2.63	4.53	18.69511	10.31308	9.4	10.4	3.5	3.7	49	67	39.3	21.2	18.77395	20.4893	139	247	2350	681	6.30559	6.21778	7.6	Class C

191	79	M	21.13268	1	0	0	0	0	1	0	0	1	1	1	0	0	0	0	0	0	0	134	132	85	72	1.96	1.93	26.51274	26.96392	10	11.9	3.9	4.2	61	66	115	60.5	30.34826	27.27273	2710	1030	6280	6080	0.90909	1.8949	5.3	Class A
192	84	F	15.54067	0	0	1	0	1	0	0	0	0	0	0	0	0	1	1	0	0	0	124	136	58	62	1.4	1.59	27.81723	24.20191	9.6	9.7	2.6	3.6	36	58	79.2	43	15.85903	17.79141	7030	349	1190	1240	0.92092	3.14286	NA	Class A
193	67	F	23.37258	0	1	0	0	1	0	0	0	0	0	0	0	0	0	0	0	0	0	137	143	75	86	2.73	3.37	14.29555	11.35365	10.2	10.3	4.2	4.1	86	125	16.6	13.6	26.46154	34.62604	188	130	408	353	2.37307	1.98565	5.6	Class A
194	75	M	24.25565	0	1	0	0	0	1	0	1	0	0	0	0	1	1	1	0	0	0	158	175	49	83	2.06	2.35	25.48522	21.98163	9	13.5	2.9	2.4	57	105	79.1	62.4	27.27273	51.47059	2030	151	13600	21800	6.15385	14.96496	5.4	Class A
195	37	F	20.68906	1	0	0	0	1	0	0	0	1	0	0	0	0	0	0	0	0	1	128	102	71	62	1.9	NA	25.20089	NA	9.1	NA	3.3	NA	72	71	47.3	68	30.76923	30.34188	141	226	1910	1970	4.22	NA	7.7	Class A
196	64	M	NA	0	0	1	0	0	1	0	1	1	0	0	0	0	1	1	0	0	0	158	NA	98	NA	1.62	1.69	34.53712	32.97521	11	9.4	3.7	3.5	100	83	103	110	32.46753	27.85235	1470	3390	102	123	0.05	0.06667	5.9	Class A
197	56	F	20.93664	0	1	0	0	0	0	1	1	0	0	0	0	0	1	1	0	0	1	173	165	101	97	2.16	2	19.44571	21.15385	7.8	8.8	3.2	3.4	NA	55	NA	148	NA	23.01255	NA	16000	NA	1640	0.57899	0.01392	4.6	Class A
198	76	M	21.35945	0	0	1	0	1	0	0	1	0	0	0	0	0	1	1	0	0	0	143	146	71	76	1.94	1.7	27.11147	31.32543	9.6	10.4	4.3	4.2	124	100	453	308	45.09091	41.15226	437	1450	93	83	0.24955	0.11194	NA	Class A
199	75	M	22.2707	1	0	0	0	0	0	1	0	1	1	0	1	0	0	0	0	1	1	132	132	60	54	1.91	2.17	27.68271	24.07533	11.2	13.5	3.8	3.2	61	NA	57.4	NA	20.33333	NA	246	NA	205	NA	4.01149	9.20339	5.8	Class A
200	81	M	20.83253	0	0	1	0	0	1	0	0	0	1	0	0	0	0	0	0	0	0	107	114	62	65	2.51	NA	20.08276	NA	10.2	NA	4.1	NA	43	39	99.7	99.6	15.80882	18.05556	1890	4050	6270	10500	1.59048	NA	NA	Class A
201	72	M	20.63633	1	0	0	0	1	0	0	1	1	0	0	0	0	1	1	0	0	1	160	120	80	70	5.61	7.35	8.61751	6.38719	10.2	10.4	4.2	NA	99	91	39.4	34.7	31.13208	28.70662	364	478	286	679	NA	NA	NA	Class A
202	73	M	21.61084	1	0	0	0	0	1	0	0	1	0	1	0	0	1	1	0	0	0	113	101	65	74	3.41	3.6	14.79772	13.94547	10	12.4	3.6	4	88	95	67.2	29.3	36.21399	30.06329	5550	3920	6170	10600	4.34579	5.82397	6.4	Class C
203	73	M	28.959	0	0	0	1	1	0	0	0	0	0	0	0	0	0	0	0	0	1	110	126	58	73	2.7	2.5	19.10364	20.78173	10.3	11.5	3.4	3.2	95	85	86.7	58.4	38.61789	35.26971	2370	2450	404	322	0.42095	0.3	NA	Class A
204	72	M	24.61662	0	0	0	1	0	1	0	0	0	0	0	0	0	1	1	0	0	0	124	124	54	66	2.76	2.9	18.72375	17.66708	10.2	12.1	3.5	3.5	84	60	278	138	35.14644	25.86207	208	2280	344	385	1.10018	2.44697	6.3	Class A
205	78	F	24.32604	0	0	0	1	0	0	0	0	1	1	0	0	0	0	0	0	0	0	126	126	60	66	1.57	1.65	25.06691	23.74038	10.7	11.6	3.7	3.8	44	61	208	148	16.17647	22.26277	2120	1360	445	453	0.00036	NA	6	Class A
206	88	F	16.54915	0	1	0	0	0	0	0	0	0	0	1	0	0	0	0	0	0	0	118	110	60	68	1.2	1.04	32.49042	37.87362	9.3	11	3.3	3.1	76	83	279	166	32.20339	37.38739	401	700	1080	2970	0.23973	0.67308	NA	Class A
207	63	M	16.60133	0	1	0	0	0	1	0	1	0	0	0	0	0	0	0	0	0	0	151	124	92	83	3.3	3.8	16.00062	13.71222	10.3	11.6	3.9	4	108	120	48.9	22.2	34.3949	33.99433	20100	697	356	349	1.12782	1.75601	NA	Class A
208	69	M	21.2715	1	0	0	0	0	1	0	0	1	1	0	0	0	1	1	0	0	0	138	152	86	68	3.07	3.44	16.87029	14.89557	8.8	9.7	2.9	2.9	43	56	109	68	16.86275	21.21212	229	138	1190	777	10.07749	7.52674	NA	Class A
209	70	M	19.55034	0	0	1	0	0	1	0	0	1	1	0	0	0	0	0	0	0	1	127	136	77	67	2.82	2.81	18.43682	18.43342	9.8	9	3.1	2.7	24	45	441	311	13.95349	25.71429	109000	37100	369	4210	1.07059	2.33766	5.9	Class C
210	49	F	17.41915	0	0	0	1	1	0	0	0	0	0	0	0	0	1	1	0	0	1	120	130	70	70	2.63	2.83	16.29027	15.03507	9.3	12.3	NA	NA	83	NA	22.8	NA	27.94613	NA	1300	NA	277	NA	4.68983	7.29008	NA	Class A
211	78	M	NA	0	0	1	0	0	1	0	0	0	0	0	0	0	0	0	0	0	0	NA	126	NA	87	1.83	1.73	28.57994	30.39207	7.6	12.2	3.4	3.6	24	44	287	102	13.40782	19.21397	29200	176	2760	423	0.29508	NA	5.6	Class A
212	68	M	25.43129	0	0	0	1	0	1	0	0	1	1	1	0	1	0	0	0	1	1	130	148	78	77	2.19	1.95	24.51466	27.83388	9.4	11.6	3.3	3.1	63	70	340	166	21	25.73529	1400	1950	77	84	3.30631	8.67742	5.6	Class A
213	51	M	24.84851	1	0	0	0	0	1	0	1	1	0	0	0	0	1	1	0	0	0	162	NA	78	NA	1.91	NA	30.75093	NA	11.5	NA	3.6	NA	82	NA	384	NA	26.88525	NA	130	NA	47	NA	4.26823	NA	7.2	Class A
214	85	M	19.06771	0	1	0	0	1	0	0	0	0	0	0	0	0	0	0	0	0	0	125	128	85	76	0.88	0.98	62.34397	55.41881	10.7	11.4	4.4	4	96	122	52	38.8	30.57325	35.56851	128	842	324	541	0.05063	0.03419	5.2	Class A
215	68	M	NA	0	0	0	1	1	0	0	1	1	0	0	1	0	1	1	0	0	0	180	132	72	52	2.92	3.58	17.89546	14.25949	6.7	8.3	NA	1.8	37	34	29.3	99.1	17.78846	24.63768	7140	2780	10600	8910	3.95283	2.87342	NA	Class B
216	58	F	21.82855	0	0	1	0	0	0	1	0	0	0	0	0	0	1	1	0	0	0	130	128	86	86	3.34	3.65	11.94988	10.84411	10.2	11.2	4.1	4.1	56	67	34.8	24	19.64912	21.89542	1170	1910	177	205	0.67347	0.97436	NA	Class A
217	57	M	29.13728	0	0	0	1	0	0	0	1	0	0	0	0	0	1	1	0	0	0	178	164	90	94	5.21	6.71	9.99183	7.53813	8.8	9.7	2.7	2.4	112	294	631	861	27.58621	85.46512	975	1080	1900	2680	11.95219	10.14925	4.9	Class C
218	72	F	NA	0	0	1	0	0	0	0	0	0	0	0	0	0	0	0	0	0	0	135	132	79	76	2.75	2.63	13.8919	14.5868	9.4	11.6	4.1	4	60	86	30.2	40	20.33898	29.35154	1000	1290	814	345	0.50521	0.49005	5.4	Class A
219	74	M	25.59434	0	1	0	0	0	1	0	1	0	0	0	0	0	0	0	0	0	0	143	NA	86	NA	3.95	5.29	12.55033	9.11741	10.1	9.8	3.7	3.5	89	88	104	101	29.96633	32.35294	3230	4010	514	622	4.29245	3.60058	NA	Class B
220	67	M	21.42836	0	0	1	0	0	1	0	0	0	0	0	1	0	1	1	0	0	0	138	115	60	56	2.73	2.71	19.34445	19.41795	9.2	10.3	3.9	4	63	NA	137	NA	23.07692	NA	640	NA	466	NA	0.53666	0.46154	5.6	Class B
221	65	F	19.3517	0	0	1	0	1	0	0	1	1	0	1	0	0	1	0	1	1	1	177	196	95	101	1.94	2.17	20.95487	18.45653	9.3	12.5	3.3	3.5	68	69	61.9	68.1	29.05983	26.23574	1230	2670	2190	1870	NA	3.53125	6.3	Class A
222	64	F	33.15638	0	0	0	1	1	0	0	0	1	0	0	0	0	1	1	0	0	0	131	132	66	63	1.99	2.25	20.47044	17.89719	10.7	11.2	3.7	3.8	81	81	49.7	70.9	22.75281	23.4104	1090	1310	262	213	0.18182	0.17143	6.9	Class A
223	67	M	22.65625	0	1	0	0	0	1	0	0	0	0	0	0	0	1	1	0	0	0	120	140	70	75	2.07	2.1	26.18465	25.77569	11.5	12.4	4.6	4.1	133	96	153	110	44.03974	33.68421	200	391	132	98	1.08734	1.80439	5.3	Class A
224	75	F	19.21321	0	0	1	0	1	0	0	1	1	0	0	0	0	1	1	0	0	0	145	128	78	83	3.79	5.36	9.66657	6.61603	8.2	11.3	2.6	3	53	18	150	18.8	23.04348	6.143345	50	1040	1330	779	5.13433	4.34615	NA	Class C
225	62	M	26.96655	1	0	0	0	1	0	0	0	1	0	0	0	0	0	0	0	0	0	112	156	70	76	5.94	4.83	8.45012	10.54758	10.3	10.9	3.6	3.7	92	49	37.4	10.4	25.77031	12.53197	767	299	702	1020	0.38806	0.66743	6.1	Class B
226	70	M	21.93635	1	0	0	0	0	1	0	0	1	0	0	1	0	1	1	0	0	0	121	122	61	65	2.79	2.84	18.65381	18.2205	9.5	10.7	4.1	3.6	64	81	418	321	32.82051	44.75138	215	395	576	674	2	2	5.5	Class B
227	50	M	27.39818	1	0	0	0	0	0	1	0	1	0	0	0	1	1	1	0	0	0	116	138	65	82	4.1	3.2	13.48378	17.6833	10.1	11.3	3.7	3.4	41	49	122	79.2	16.26984	23.22275	4130	12600	55	203	2.43333	5.39683	11.3	Class A
228	75	M	23.30668	0	1	0	0	1	0	0	0	0	0	0	0	0	0	0	0	0	1	121	135	60	80	4.31	4.68	11.32114	10.34569	9.5	10.3	4.07	4.06	79	75	179	198	28.11388	27.57353	427	1930	1040	1130	2.00242	2.35488	NA	Class B
229	59	M	25.82231	1	0	0	0	0	0	1	0	1	0	0	0	0	1	1	0	0	0	127	120	86	64	5.75	5.74	8.88159	8.8557	10.2	9.7	4.5	4.1	NA	NA	NA	NA	NA	NA	NA	NA	NA	NA	1.48649	1.48225	5.2	Class B
230	75	F	26.66667	0	1	0	0	1	0	0	0	1	0	0	0	0	0	0	0	0	0	136	114	66	68	1.85	1.71	21.18448	23.00143	9	12.6	4.1	4.3	96	116	187	80.4	21.67043	27.68496	1070	2170	151	79	0.05489	0.42169	7.2	Class A
231	51	M	23.66144	0	0	0	1	0	1	0	0	0	0	0	0	0	1	1	0	0	0	123	132	67	75	1.83	1.8	32.40467	32.99598	10.6	13.5	2.9	3.2	110	90	215	134	40.89219	28.48101	670	2200	89	72	2.17674	4.88971	5.6	Class A
232	66	F	22.75208	0	0	0	1	0	1	0	0	0	0	0	0	0	1	1	0	0	0	128	147	64	72	2.04	2.4	19.74702	16.5305	9.7	10.1	3.9	3.9	75	52	74.4	44.3	25.25253	19.84733	4190	3100	279	330	2.87958	1.26126	NA	Class B
233	48	M	23.57459	0	0	1	0	1	0	0	0	1	0	0	0	0	1	1	0	0	0	129	142	72	82	2.57	3.36	22.74148	16.96174	9.3	11.1	3.3	3.5	94	80	105	125	36.71875	29.09091	751	6510	688	150	1.39434	1.20968	NA	Class B
234	79	M	27.87926	1	0	0	0	0	1	0	0	1	1	0	0	0	1	1	0	0	0	120	168	80	58	3.02	NA	16.52172	NA	9.1	NA	3	NA	53	73	23.1	25.6	21.9917	32.01754	2990	734	890	1140	3	NA	7.5	Class C
235	71	M	22.03065	0	0	1	0	0	1	0	0	0	0	0	0	0	0	0	0	0	0	128	140	85	89	3.06	3.13	16.79234	16.3163	10.1	11.8	4.1	4.1	63	54	49.3	34	24.90119	17.58958	168	669	140	112	0.81188	1.06145	5.5	Class A
236	75	M	20.62626	1	0	0	0	0	1	0	0	1	1	0	0	1	0	0	0	0	0	110	NA	88	NA	3.62	3.81	13.75409	13.00551	9	9	3.5	3.7	NA	37	NA	38.1	NA	13.0742	NA	3510	NA	2410	0.88889	1.36667	5.9	Class A
237	76	M	20.65744	1	0	0	0	1	0	0	0	1	0	0	1	1	1	1	0	0	1	127	NA	81	NA	2.18	NA	23.86365	NA	10.9	NA	3.9	NA	71	71	7.4	32.2	21.64634	25.1773	748	958	523	277	0.89713	NA	6.9	Class A
238	79	M	23.59494	1	0	0	0	0	1	0	0	1	0	0	0	0	1	1	0	0	1	122	130	63	61	1.4	1.45	38.31058	36.73485	9.3	10.5	4	3.9	58	93	30	25.2	20.78853	31.41892	428	302	78	58	0.10465	0.09756	6.6	Class A
239	69	F	20.91011	0	0	0	1	1	0	0	0	0	0	0	0	0	1	1	0	0	0	126	127	83	83	2.12	2.07	18.69326	19.18779	11	11	4	4	67	96	48.1	50.5	24.63235	34.53237	156	318	111	134	0.875	1.70588	NA	Class A
240	49	F	22.68431	1	0	0	0	1	0	0	1	1	0	0	0	0	0	0	0	1	1	142	120	80	74	2.97	4.05	14.26147	10.1579	9.9	11.9	3.4	3.2	48	36	62.3	40.5	18.60465	14.4	317	1670	541	635	4.40351	1.90329	8.8	Class A
241	78	F	22.71582	0	1	0	0	1	0	0	0	0	0	0	1	0	0	0	0	1	1	106	126	70	66	1.9	1.73	20.34504	22.54201	10.1	11.7	4.1	4.2	58	92	177	45.6	21.56134	31.39932	1330	1860	537	635	0.57578	0.23095	NA	Class A
242	57	M	23.61834	1	0	0	0	0	1	0	1	1	1	0	0	0	1	1	0	0	1	143	139	86	86	1.27	1.17	46.80631	51.20004	10.3	11.9	4.7	4.3	48	NA	44.2	NA	16.72474	NA	265	NA	208	NA	0.09003	NA	6.5	Class A
243	69	M	22.12824	0	0	1	0	0	1	0	1	1	1	0	0	0	1	1	0	0	1	162	146	70	66	3.9	4.54	12.98456	10.99595	8.2	10	2.9	3	84	87	16.9	59.8	29.78723	33.98438	1180	1040	1160	1330	12.14511	10.51095	6.4	Class C
244	88	M	NA	0	0	1	0	0	1	0	0	1	1	0	0	0	1	1	0	0	0	NA	NA	NA	NA	1.5	1.27	34.44224	41.3213	9.3	11.1	2.9	2.9	75	100	61.4	31.1	28.84615	30.30303	307	288	498	670	0.77049	2.37255	NA	Class A
245	89	F	22.8862	0	1	0	0	1	0	0	0	0	0	1	0	0	0	0	0	0	0	135	140	55	61	1.75	1.95	21.43323	19.04028	9.5	10.1	4.3	4.1	57	40	234	172	26.63551	19.32367	589	2810	1760	1330	0.04024	0.04854	NA	Class B
246	73	M	23.43392	0	0	0	1	1	0	0	1	0	0	0	1	0	0	0	0	0	0	149	123	70	67	5.51	7.53	8.75403	6.22034	9.4	10.3	4	3.7	60	27	234	207	26.54867	12.85714	790	15100	558	1070	3.7471	7.00379	5.5	Class C
247	85	M	NA	0	0	1	0	0	0	0	0	0	1	1	0	0	0	0	0	0	0	NA	90	NA	50	1.16	1.45	46.08308	35.98024	9.8	10.9	NA	3.6	79	104	496	486	32.2449	47.70642	873	3680	5350	7580	0.39394	NA	NA	Class A
248	82	M	18.66688	0	0	0	1	0	1	0	0	0	0	1	0	0	1	0	1	0	0	119	139	47	64	1.21	1.31	44.46005	40.76077	10.5	10.2	4.01	3.99	56	73	64.1	128	17.55486	23.7785	208	254	2600	1600	0.10072	0.06928	NA	Class A
249	62	M	26.17383	0	1	0	0	1	0	0	0	1	0	0	1	0	1	1	0	0	0	136	146	74	77	4.19	4.86	12.37893	10.52459	9.9	10.7	3.7	3.8	38	82	193	157	19.28934	37.44292	1140	327	128	130	3.87826	2.17135	5.8	Class A
250	74	F	20.08766	0	0	0	1	0	0	0	1	0	0	0	0	0	1	1	0	0	0	155	163	92	91	2.17	2.24	17.86034	17.25064	9.7	10.7	4.1	3.9	96	104	137	109	37.2093	43.88186	50	50	4170	4670	2.90863	3.20865	5.7	Class A
251	77	F	23.8728	0	1	0	0	1	0	0	1	0	0	0	0	0	1	1	0	0	1	152	173	64	58	1.27	1.1	31.72941	36.99401	8.6	10.9	3.98	3.96	48	52	116	80.4	16.72474	17.93103	103	67	390	359	0.0625	0.21127	NA	Class A
252	80	F	37.71219	0	1	0	0	0	1	0	1	1	1	1	0	0	1	1	0	1	1	162	181	65	70	1.53	1.36	25.59805	29.01479	10.3	10.7	3.5	3.6	58	79	72	75.2	21.16788	29.47761	557	433	1720	3080	1.7644	6.16229	6.5	Class A
253	86	F	15.36397	0	0	0	1	1	0	0	0	0	1	0	0	1	1	1	0	0	1	128	152	78	76	1.15	1.02	34.26429	39.06938	10	13.3	4.1	4.1	35	95	32.1	40	11.47541	34.29603	749	616	3190	3690	0.32258	0.27778	NA	Class A
254	59	M	20.25019	1	0	0	0	0	1	0	1	1	0	0	0	0	0	0	0	0	0	151	125	81	73	1.94	1.94	29.15494	29.01464	9.7	11.6	3	3.1	87	50	212	77.4	35.08065	20.83333	124	106	311	347	6.51741	4.22777	5.7	Class A
255	69	F	20.05344	0	1	0	0	0	0	1	0	0	0	0	0	1	1	1	1	0	0	106	109	56	60	3.11	2.99	12.29183	12.83253	10.3	12.3	3.9	4.3	74	114	67.5	104	22.42424	31.23288	247	639	768	590	0.35211	0.94183	5.7	Class A
256	71	M	23.73107	1	0	0	0	0	0	1	0	1	1	0	0	1	1	1	0	0	0	102	110	60	52	1.25	1.43	44.71683	38.44233	9.7	13.4	3.3	3.7	109	NA	203	NA	44.85597	NA	73	NA	305	NA	1.78409	2.0125	6.1	Class A
257	45	M	35.22286	1	0	0	0	1	0	0	1	1	0	0	0	0	1	1	0	0	0	142	125	74	73	3.3	3.4	17.51201	16.94932	9.9	10.3	4.2	4.4	66	75	373	341	25.68093	28.73563	1220	2490	501	525	2.21381	2.9142	6.8	Class A
258	79	M	25.36791	1	0	0	0	0	1	0	0	1	0	0	1	0	1	1	0	1	1	134	140	67	65	2.08	2.43	24.844	20.8815	11.4	11.8	3.4	2.9	238	76	33.6	99.7	90.4943	33.18777	1360	4580	4050	3200	7.07921	6.95	7.3	Class A
259	81	M	25.49866	0	0	0	1	0	0	1	0	0	0	0	0	0	1	1	0	0	0	NA	NA	NA	NA	2.84	2.65	17.54431	18.92498	8	9	3.4	3.6	NA	NA	NA	NA	NA	NA	NA	NA	NA	NA	0.29032	NA	NA	Class A
260	59	F	22.84525	0	0	0	1	1	0	0	1	0	0	0	0	0	1	1	0	0	1	152	154	86	84	1.66	1.88	25.55132	22.19157	9.1	9.3	3.1	3.1	52	63	36.1	43.2	19.62264	28.89908	130	396	238	300	NA	NA	5.6	Class A
261	78	F	21.9944	0	0	1	0	1	0	0	0	0	0	0	0	0	1	1	0	0	1	134	125	73	67	3.04	3.51	12.1217	10.35765	10.3	9.9	4.01	3.67	51	47	68.8	50.9	17.70833	14.59627	725	520	1360	648	3.40813	3.91494	NA	Class C
262	80	F	23.17382	0	1	0	0	1	0	0	0	0	0	0	0	0	0	0	0	1	1	132	149	68	78	2.02	1.88	18.88883	20.43293	7.8	10.8	3	3	16	46	108	50.3	6.9869	20.90909	5110	867	1030	953	4.14462	4.40523	NA	Class B
263	81	F	28.1996	0	0	0	1	1	0	0	1	0	0	0	0	0	1	1	0	0	0	161	194	66	66	3.97	4.01	8.95581	8.85812	7	9.1	3.2	3.3	44	29	144	51.5	21.25604	13.80952	147	145	1390	1560	4.38196	4.9354	5.5	Class C
264	69	M	24.75159	0	0	0	1	0	0	0	0	1	1	0	0	0	0	0	0	0	0	NA	NA	NA	NA	2.18	2.15	24.53469	24.90946	11.9	13.5	3.9	3.9	48	75	94.1	31.3	17.45455	21.61383	1170	1640	4860	3950	1.57143	1.72414	NA	Class A
265	81	F	27.30123	0	1	0	0	1	0	0	1	1	0	0	0	0	1	1	0	0	0	142	154	78	90	1.63	2.13	23.80004	17.76085	9.9	11.5	3.6	3.9	81	41	26.9	9.3	32.27092	12.0944	150	304	1480	13100	1.21643	1.46533	5.2	Class A
266	76	M	20.74238	0	0	1	0	0	0	1	0	1	1	0	0	0	1	1	0	0	0	123	114	65	60	2.25	2.28	23.05263	22.721	10.7	10.8	3.8	3.6	109	76	178	212	46.5812	33.48018	426	501	264	258	1.09232	1.34776	NA	Class A
267	84	M	22.23201	0	1	0	0	1	0	0	0	0	0	0	0	0	1	1	0	1	1	105	132	42	70	2.43	2.79	20.59114	17.70283	8.4	11.1	4	3.95	94	74	44.1	65.2	31.86441	25.96491	145	392	650	510	1.52547	2.44455	NA	Class B
268	74	M	26.13346	1	0	0	0	1	0	0	0	1	0	0	0	0	0	0	0	0	0	122	129	71	68	2.99	3.29	17.01955	15.32921	10.1	10.7	3.77	3.19	66	70	274	249	25.28736	27.77778	3030	2760	439	834	3.50701	3.54705	7.1	Class C
269	60	F	17.58309	0	0	0	1	1	0	0	0	0	0	0	0	0	1	1	0	0	0	110	118	78	78	3.36	3.75	11.75714	10.42621	10	11.1	3.5	3.6	104	99	49.5	35.8	35.49488	35.23132	109	152	196	158	2.43902	1.99601	NA	Class A
270	68	M	23.45786	0	0	0	1	0	1	0	1	0	0	0	0	0	1	0	1	0	0	155	NA	90	NA	4.38	NA	11.48415	NA	10.3	NA	4	NA	117	NA	13.8	NA	34.11079	NA	361	NA	425	NA	2.08824	NA	NA	Class A
271	72	M	NA	0	0	0	1	0	0	1	0	1	0	0	0	0	0	0	0	0	0	NA	NA	NA	NA	1.49	1.32	36.75205	41.96038	9.1	10.3	2.6	2.9	21	53	231	136	13.3758	32.12121	20000	7980	226	283	0.79211	1.42105	7.2	Class A
272	70	M	23.69201	0	0	1	0	0	1	0	0	1	0	0	0	0	0	0	0	0	0	114	113	67	66	2.97	3.34	17.4206	15.2585	10.6	13	3.6	3.9	60	96	55.4	30.4	22.90076	29.00302	1520	1690	257	328	1.07576	0.90816	5.9	Class A
273	86	F	17.4646	0	1	0	0	1	0	0	0	0	0	0	0	0	1	1	0	0	0	133	119	83	69	3.69	3.43	9.57014	10.33219	11.3	10.8	4.4	4.2	161	117	654	628	61.92308	53.66972	193	1280	2160	2370	0.24931	0.43478	NA	Class A
274	69	F	24.89074	0	0	0	1	1	0	0	0	1	1	0	0	0	0	0	0	0	0	NA	NA	NA	NA	1.47	1.21	27.90303	34.52465	10.1	10.7	3.3	3	137	124	119	86.2	39.94169	42.0339	97	80	473	491	2.7501	4.43947	NA	Class A
275	65	M	19.58592	0	0	0	1	0	1	0	0	0	1	1	1	0	0	0	0	0	0	132	136	60	78	1.9	2.19	29.00943	24.83417	9.6	10.9	4	3.6	112	83	135	129	43.57977	33.60324	89	19400	140	111	1.26667	0.81679	NA	Class A
276	68	M	22.78726	0	1	0	0	0	1	0	1	0	0	0	0	0	0	0	0	0	1	141	107	76	65	2.79	3.12	18.80965	16.64434	10.3	13	3.6	3.5	67	91	44.5	66.9	21.75325	33.33333	428	596	232	169	0.84463	1.55978	5.8	Class A
277	94	M	26.3656	0	1	0	0	0	1	0	0	0	1	0	1	1	1	1	0	1	1	107	112	50	57	1.92	NA	25.79779	NA	9.4	NA	3.5	NA	39	54	102	54	15.91837	21.01167	22900	17900	2080	1260	0.08827	NA	NA	Class A
278	67	M	24.45502	1	0	0	0	0	0	1	1	1	0	0	0	1	1	1	0	0	0	154	147	67	75	3.06	3.74	17.07415	13.65055	7.5	10	3.6	3	101	116	358	662	50.5	89.92248	2870	8680	2920	1600	1.21053	1.00559	5.4	Class A
279	60	F	17.45161	0	0	1	0	0	1	0	0	0	0	0	0	0	1	0	1	0	0	129	136	68	84	2.17	1.87	18.96836	22.32143	10.1	11	3.4	3.4	57	83	67.5	23.1	24.67532	30.07246	24000	1890	874	952	0.50761	0.22121	5.6	Class B
280	79	F	22.75047	1	0	0	0	1	0	0	0	1	0	0	0	0	1	1	0	0	0	120	118	70	62	3.97	3.68	9.05212	9.83535	9.7	12	4.3	NA	44	78	157	67	15.33101	24.7619	1600	1170	6500	4680	0.47414	0.86705	5.6	Class A
281	77	F	25.99318	0	1	0	0	1	0	0	0	1	1	1	0	1	0	0	0	0	1	137	131	48	51	1.75	1.34	22.34291	29.81003	10.5	11.4	3.9	3.4	57	55	28.1	51.5	16.33238	15.98837	1090	2230	3680	4370	0.26866	0.34	NA	Class A
282	83	M	18.89698	0	0	0	1	1	0	0	0	0	0	0	0	1	0	0	0	1	1	103	125	60	68	1.4	1.5	37.77133	34.90517	11	12.1	3.9	4	111	92	27.9	59.3	42.52874	34.98099	1070	1120	627	1270	0.09964	0.28517	NA	Class A
283	83	M	19.50458	0	0	1	0	0	1	0	0	1	1	0	0	0	1	1	0	0	1	104	117	67	68	1.51	1.43	34.77167	36.9053	11	13.2	3.7	3.7	92	117	32.9	18.1	31.61512	35.56231	433	382	78	70	0.07831	0.13372	5.6	Class A
284	46	F	30.35925	1	0	0	0	0	1	0	0	1	0	0	0	0	1	1	0	1	1	107	125	62	66	1.14	1.2	41.39813	39.13906	10.9	11.3	3.8	3.6	63	73	59.8	75.2	25.0996	26.35379	2980	642	113	40	1.50356	2.827	6.6	Class A
285	77	F	22.17778	0	1	0	0	1	0	0	1	0	1	0	0	0	1	1	0	1	1	144	128	70	78	1.53	1.72	25.8804	22.68542	9.8	12.4	4.2	4.4	61	83	38.7	65.4	21.47887	29.12281	337	917	251	234	0.0625	0.02381	NA	Class A
286	67	M	NA	0	0	0	1	1	0	0	0	0	0	0	0	0	1	1	0	0	0	135	NA	85	NA	3.58	NA	14.38038	NA	9.7	NA	3.6	NA	120	NA	110	NA	38.70968	NA	50	NA	865	NA	1.79592	NA	NA	Class B
287	75	M	22.41426	0	1	0	0	0	1	0	0	0	0	0	0	0	1	1	0	1	1	136	140	76	67	2.58	3.01	19.92265	16.8309	9.6	10.6	3.8	3.5	58	56	181	91.1	23.57724	22.67206	1330	932	305	265	1.36923	2.23626	5.5	Class B
288	52	F	22.32829	0	0	0	1	1	0	0	0	0	0	0	0	0	1	1	0	1	1	125	118	74	80	2.87	3.01	14.55561	13.81661	10.1	12.3	3.3	3.5	89	70	59.6	74.9	37.87234	32.11009	653	614	221	134	3.48	4.99697	NA	Class A
289	87	M	22.68502	0	1	0	0	0	1	0	1	0	0	0	0	0	1	1	0	0	0	153	113	99	69	3.98	4.62	11.88195	10.09349	10.5	12.8	4.1	4.2	61	73	313	232	25.10288	29.08367	1740	1220	1330	884	1.48	1.29432	NA	Class A
290	51	F	18.86717	0	0	1	0	1	0	0	0	0	0	0	0	0	1	1	0	1	1	111	NA	74	NA	2.65	NA	15.97138	NA	10.9	NA	3.9	NA	73	82	41.6	51.9	25	31.06061	75	70	231	440	1.88889	NA	NA	Class A
291	79	M	25.69348	1	0	0	0	0	1	0	0	1	0	0	0	0	0	0	0	0	0	124	NA	62	NA	3.21	NA	15.4549	NA	10.2	NA	NA	NA	68	NA	26	NA	25	NA	824	NA	7750	NA	2.84298	NA	NA	Class A
292	69	F	29.25176	0	1	0	0	1	0	0	1	0	0	0	0	0	0	0	0	0	0	207	151	109	84	4.06	6.18	9.18267	5.79903	9.4	10.3	3.5	3.9	94	52	57.7	36.6	29.74684	16.19938	957	1260	1070	2560	3.47458	2.31746	5.6	Class B
293	38	F	22.31429	0	0	1	0	0	0	1	0	0	0	0	0	0	1	1	0	1	1	115	141	71	83	2.75	3.04	16.68854	14.95494	9.8	11.1	3.96	3.93	92	97	292	309	43.39623	44.70046	52	65	184	226	NA	1.31764	NA	Class B
294	57	M	NA	1	0	0	0	0	1	0	0	1	0	0	1	0	1	1	0	0	0	138	132	80	80	3.88	3.66	13.79379	14.70339	10.4	10	4.4	4.3	73	78	152	108	27.03704	30.46875	251	158	257	554	NA	NA	5.6	Class A
295	65	F	NA	0	0	1	0	0	1	0	0	0	0	0	0	0	1	1	0	0	0	NA	NA	NA	NA	0.79	1.07	55.99329	40.17854	9.2	9.2	3.5	3.9	29	NA	43.5	NA	11.24031	NA	72500	NA	275	NA	1.2619	0.45333	NA	Class B
296	71	M	22.05964	0	0	0	1	0	1	0	0	0	0	0	0	0	0	0	0	0	0	121	174	59	92	3.07	3.53	16.73251	14.36234	10.6	12.3	3.54	3.18	96	114	380	260	36.22642	45.96774	2500	3620	543	866	1.69091	4.52174	NA	Class A
297	46	M	29.20479	1	0	0	0	0	1	0	1	1	0	0	1	0	1	1	0	0	0	158	120	95	82	4.3	6.23	13.10919	8.73817	10.6	11.5	2.4	3.2	70	120	430	487	37.83784	49.38272	154	1040	927	221	8.74325	5.25745	4.9	Class B
298	76	M	21.33821	1	0	0	0	0	0	1	0	1	0	0	0	1	1	1	0	0	0	105	110	65	54	4.53	4.63	10.68089	10.42877	9.7	10.8	3	3.3	101	99	132	86.3	38.69732	37.21805	4330	4220	650	373	1.43472	NA	5.8	Class A
299	67	F	32.6822	0	0	1	0	1	0	0	0	1	0	0	0	0	0	0	0	0	1	127	164	78	72	3.41	3.52	11.20803	10.82543	9.6	9.7	3.5	3.6	68	59	19.8	14	29.69432	24.58333	428	391	406	636	3.90707	4.16138	6.7	Class B
300	67	F	26.45484	0	1	0	0	1	0	0	0	1	0	0	0	0	1	1	0	0	0	131	166	54	68	2.13	2.06	18.67535	19.3707	9.1	10.9	3.9	3.9	57	62	129	125	24.35897	25	426	1580	236	479	0.90895	1.30332	6.6	Class A
301	87	F	21.76529	0	0	0	1	1	0	0	0	0	0	0	0	0	1	1	0	0	0	123	114	55	44	2.96	2.72	12.13979	13.27276	10.5	13.2	3.4	3.2	85	74	109	67.6	37.44493	33.63636	1140	1610	532	353	1.2987	1.23967	NA	Class A
302	69	M	21.59087	1	0	0	0	0	0	1	1	1	0	0	0	0	1	0	1	0	0	140	147	61	66	4.55	4.88	10.92431	10.11876	9.2	12	2.8	3.5	58	94	178	62.2	25.21739	40.51724	195	173	188	294	NA	6.7381	NA	Class B
303	80	F	24.06652	0	0	0	1	1	0	0	0	1	1	0	0	0	0	0	0	0	0	124	112	79	66	3.33	3.53	10.93214	10.25637	9.7	12.1	4.2	4.1	103	NA	170	NA	39.31298	NA	229	NA	4150	NA	1.61538	1.78487	5.8	Class A
304	60	M	21.69163	0	1	0	0	0	0	1	1	0	0	0	1	0	0	0	0	0	0	175	152	92	84	4.98	5.28	10.34436	9.65719	9.5	10.1	3.7	3.5	76	58	325	119	32.47863	25	372	144	457	2350	3.85787	3.72737	NA	Class A
305	79	M	30.07813	1	0	0	0	0	1	0	0	1	0	0	0	1	0	0	0	0	0	120	130	70	64	3.88	3.94	12.56032	12.35122	9.2	10.3	3.7	3.8	56	64	86.6	79.7	21.29278	24.06015	211	760	1270	1340	2.69606	2.87958	5.9	Class C
306	61	M	19.37716	1	0	0	0	1	0	0	1	1	0	0	0	0	1	1	0	0	0	143	129	79	55	2.16	2.02	25.67554	27.49989	9.5	10.4	3.7	3.6	84	73	168	130	38.18182	31.73913	50	104	557	444	NA	0.65641	6.2	Class A
307	71	F	NA	0	1	0	0	1	0	0	0	0	0	0	0	0	0	0	0	1	1	NA	NA	NA	NA	1.14	1.02	36.54947	41.27874	9.1	10	4	NA	56	62	50	44.9	19.11263	21.08844	239	495	355	196	0.05376	0.0722	NA	Class A
308	78	M	18.08891	0	1	0	0	0	1	0	0	1	1	1	0	0	1	1	0	0	0	101	113	63	68	2.51	2.43	20.30147	21.03378	10	12.4	3.5	3.9	59	80	426	434	29.64824	35.24229	1310	2160	2750	2220	0.03324	0.05184	6.1	Class A
309	73	M	22.3757	1	0	0	0	0	1	0	1	1	1	0	0	0	1	1	0	0	0	146	144	65	62	2.41	NA	21.63224	NA	10.4	11.5	3.9	NA	61	62	119	80.8	22.76119	24.21875	447	101	1080	1010	1.31269	1.296	NA	Class A
310	76	M	20.95661	1	0	0	0	0	0	1	0	1	0	0	1	0	0	0	0	1	1	127	166	55	79	3.92	4.26	12.55895	11.46659	9.9	12.1	3.6	3.8	77	85	93.5	120	34.52915	35.56485	451	386	1090	1040	4.56063	5.40788	5.8	Class C
311	78	M	22.31328	0	0	0	1	0	1	0	0	0	0	0	0	0	1	1	0	0	0	125	132	81	85	3.78	3.9	12.97163	12.48987	11.4	11.2	4.1	4	63	100	96.5	94.2	23.16176	37.59398	312	201	3280	3170	0.89992	1.22677	NA	Class A
312	86	M	22.59253	0	1	0	0	1	0	0	1	0	0	0	0	0	0	0	0	0	0	151	169	67	68	2.42	2.45	20.54502	20.26996	9.5	10.9	3.4	3.3	87	93	209	144	35.08065	37.65182	7320	1320	2660	3670	3.70435	2.5	NA	Class B
313	75	M	20.40816	0	1	0	0	0	1	0	0	0	0	0	1	0	0	0	0	0	0	132	145	65	69	3.78	3.53	13.11847	14.13818	9.5	10.9	3.95	3.63	69	101	161	175	27.6	41.39344	96	229	790	713	0.64206	1.05817	NA	Class A
314	52	M	23.14815	1	0	0	0	0	1	0	1	1	0	0	0	0	1	1	0	0	0	152	117	78	67	2.88	NA	19.62155	NA	8.9	NA	3.6	NA	68	78	55.8	48	26.77165	32.09877	500	536	315	403	2.70513	NA	5.9	Class A
315	70	M	24.64864	1	0	0	0	1	0	0	1	1	0	0	0	0	1	1	0	0	1	140	128	86	80	6.43	5.4	7.483	9.05774	10	11.9	4.2	4.1	77	65	35.1	33.4	26.19048	21.88552	1290	1540	261	265	1.29853	1.25477	6.1	Class A
316	71	F	17.89119	0	0	0	1	1	0	0	1	0	0	0	0	0	1	1	0	0	0	142	147	78	88	0.9	0.81	47.33624	53.11931	10.8	11.9	4	4	75	100	34.6	40.6	24.35065	34.12969	494	725	98	171	0.1	0.20755	NA	Class A
317	51	M	18.99672	0	0	0	1	1	0	0	0	0	0	0	0	0	0	0	0	0	0	NA	NA	NA	NA	2.38	3.11	24.30826	18.1405	8	9.7	1.8	1.7	42	NA	537	NA	25.92593	NA	4490	NA	310	NA	6.04651	NA	NA	Class B
318	72	M	NA	0	1	0	0	1	0	0	0	0	0	1	0	0	0	0	0	0	0	136	NA	44	NA	3.27	4.33	15.55364	11.39489	9.9	10.6	2.8	2.5	93	NA	234	NA	48.4375	NA	276	NA	806	NA	16.94915	NA	5.6	Class A
319	80	M	20.27548	0	0	0	1	0	1	0	0	0	0	0	0	0	0	0	0	0	0	117	134	65	90	4.25	3.91	11.32811	12.41005	9.9	12.1	4.1	3.9	60	44	120	39.2	26.90583	17.05426	6700	962	422	325	0.67295	1.51515	5.4	Class A
320	87	F	23.4617	1	0	0	0	1	0	0	1	1	0	0	0	0	1	1	0	0	0	148	134	65	59	1.11	1.21	35.49938	32.30256	9.7	11.1	3.4	2.4	54	61	102	54.9	24.43439	30.19802	118	489	1040	1110	8.21739	14.82456	5.6	Class A
321	48	M	19.33363	0	1	0	0	1	0	0	0	0	0	1	0	0	1	1	0	0	0	103	125	62	77	2.14	2.26	27.78516	26.17525	10.9	10.7	3.4	3.7	50	81	54.2	48.9	17.06485	25.55205	105	71	162	220	0.28891	0.64797	NA	Class A
322	54	F	17.81366	0	0	0	1	1	0	0	0	0	0	0	0	0	1	1	0	1	1	115	107	68	67	1.87	1.96	23.00671	21.8535	10.7	12.3	4.38	4.13	92	NA	88.9	NA	33.09353	NA	206	NA	219	NA	0.73733	0.54435	NA	Class A
323	77	F	32.66339	0	1	0	0	1	0	0	0	1	0	0	0	0	1	1	0	0	0	118	117	69	59	3.32	3.52	11.08915	10.40173	9.7	10.6	4.1	4	56	81	76.8	92.7	20.74074	30.91603	102	135	439	251	1.175	0.80583	6	Class A
324	53	M	30.77498	0	0	0	1	0	0	1	1	0	0	0	0	0	1	1	0	1	1	140	120	80	70	2.85	3.2	19.73942	17.39004	9.8	9.7	2.2	2.5	60	60	146	145	28.43602	26.54867	991	892	151	181	6.97645	5.2318	NA	Class A
325	77	F	28.46409	0	1	0	0	1	0	0	0	0	0	0	0	0	0	0	0	0	0	126	115	84	79	2.18	2.07	17.5692	18.5931	10.5	10.5	4	3.5	71	49	32.3	23.9	23.27869	16.83849	1180	2040	442	293	0.63636	0.5	NA	Class A
326	66	M	21.44295	0	0	1	0	0	1	0	0	1	0	0	0	0	1	1	0	1	1	112	125	60	55	1.84	2.08	29.91451	26.15961	9.2	7.7	1.4	2.1	35	66	215	175	26.31579	39.28571	4230	323	1300	596	5.51118	5.21959	5.4	Class A
327	70	F	22.23682	0	0	1	0	1	0	0	0	0	0	0	0	0	0	0	0	0	0	108	118	69	75	1.66	NA	24.32791	NA	9.7	NA	4.4	NA	107	117	74.5	30.6	27.08861	37.26115	235	347	135	55	0.01075	NA	NA	Class A
328	72	M	20.12252	0	0	0	1	0	1	0	0	0	0	0	0	0	1	1	0	0	0	91	122	51	60	3.55	3.54	14.21666	14.2606	9.9	12	2.8	3.3	25	70	262	125	13.81215	31.96347	4600	889	458	550	4.38894	5.43703	5.8	Class A
329	83	M	20.49587	0	1	0	0	0	1	0	1	0	0	0	0	0	0	0	0	0	0	163	170	94	92	2	2.04	25.56816	25.02021	11	12.1	3.7	3.7	93	140	46.2	38.9	32.97872	48.27586	254	266	460	434	0.58388	1.06363	6.3	Class A
330	69	F	23.73333	0	0	1	0	1	0	0	0	1	0	0	0	0	1	1	0	0	0	110	122	74	70	3.33	3.74	11.40624	10.00418	9	12.2	4.1	4	85	90	133	29.6	34.55285	34.48276	281	5070	544	730	0.14345	0.80708	5.7	Class A
331	79	M	22.2707	1	0	0	0	0	1	0	0	1	0	0	1	1	1	1	0	0	0	136	148	58	60	2.5	2.77	20.2427	18.09431	9.3	11.2	2.9	2.2	61	68	237	232	28.37209	34	265	2050	4390	3940	7.93552	13.33664	5.3	Class A
332	75	F	21.57298	0	1	0	0	0	1	0	0	0	0	0	0	0	0	0	0	1	1	128	135	68	74	3.37	3.42	10.99199	10.77527	9.8	10.4	3.8	3.3	34	42	61.1	83.1	12.87879	17.21311	5970	5660	1940	3190	1.85185	6.45038	NA	Class B
333	56	M	24.72452	0	1	0	0	0	0	1	1	0	0	1	0	0	1	1	0	1	1	133	141	92	89	3.6	3.53	15.04793	15.29678	9.4	10.7	3.5	3.7	55	NA	229	NA	24.88688	NA	217	NA	2430	NA	1.70115	1.86538	NA	Class B
334	66	M	25.65435	1	0	0	0	0	1	0	0	1	1	0	0	0	1	1	0	0	0	130	140	60	80	3.12	4.33	16.78756	11.72937	9.6	11.8	2.3	2.5	41	65	118	154	22.65193	31.10048	270	125	1900	6160	11.62791	10.10101	8.7	Class B
335	44	M	20.17715	0	0	1	0	0	0	1	1	0	0	0	0	0	1	1	0	0	0	130	120	90	80	4.56	4.9	12.45155	11.5095	10	12	4	4.1	54	118	325	452	26.08696	51.52838	325	404	562	569	4.27273	4.94118	NA	Class A
336	76	F	21.14116	0	0	0	1	1	0	0	0	0	0	0	0	0	1	1	0	0	0	138	124	75	72	3.32	3.44	11.08915	10.66666	9	10.4	3.9	3.9	76	87	45.3	28.7	28.25279	29.49153	120	204	401	248	0.49477	0.87852	NA	Class A
337	69	M	23.45527	0	0	1	0	1	0	0	0	1	0	0	0	0	1	0	1	0	0	120	124	70	64	1.48	1.62	37.4788	33.95022	10.3	12.6	3.1	3	55	69	96.6	121	25.5814	30.9417	118	81	121	106	4.09375	5.40323	NA	Class A
338	74	M	19.81768	0	0	1	0	1	0	0	0	0	0	0	1	0	1	0	1	0	0	NA	132	NA	56	1.83	1.58	29.1213	34.06656	9	10.1	NA	4.4	85	71	244	136	27.15655	22.11838	763	643	692	609	NA	NA	5.6	Class B
339	71	M	NA	0	1	0	0	0	1	0	0	0	0	0	0	0	0	0	0	0	1	130	131	69	74	3.54	4.75	14.2606	10.33819	9	10.6	2.9	2.7	68	88	33.7	74.6	26.35659	37.93103	504	939	499	500	5.7541	6.56164	5.2	Class B
340	76	M	18.52557	0	1	0	0	1	0	0	1	0	1	0	0	1	0	0	0	0	1	148	110	77	58	3.05	3.16	16.52663	15.8983	10.5	10.2	NA	4.6	40	97	8.3	40.6	14.08451	31.49351	1900	571	847	673	NA	0.775	NA	Class A
341	70	M	19.11822	0	1	0	0	0	1	0	0	0	0	0	0	0	0	0	0	0	0	133	NA	66	NA	2.79	NA	18.65381	NA	11.1	NA	4.1	NA	107	90	471	376	36.89655	30.20134	174	411	1290	946	0.36268	NA	NA	Class A
342	77	F	19.99231	0	1	0	0	1	0	0	1	1	0	0	0	0	1	1	0	0	0	149	143	68	70	1.45	1.1	27.44649	37.13126	10	10.6	4.5	4.3	150	115	21.5	17.8	37.59398	28.25553	307	287	136	376	NA	0.55102	7.6	Class A
343	73	M	23.12126	1	0	0	0	0	1	0	0	1	1	1	0	0	0	0	0	1	1	102	120	50	60	3.63	2.96	13.81943	17.20834	7.7	12.1	2.9	4.1	37	145	91	125	16.74208	55.34351	4240	300	4740	2630	0.19412	0.79058	6.2	Class A
344	58	M	NA	1	0	0	0	0	0	1	0	1	0	0	0	0	1	1	1	0	0	128	162	73	92	2.77	2.69	19.84381	20.39005	9	10.2	2.3	2.4	85	65	412	175	36.95652	32.82828	103	97	1090	1290	6.52174	NA	7	Class A
345	74	M	18.14509	1	0	0	0	0	1	0	0	1	0	0	1	0	1	1	0	0	0	125	141	53	59	1.95	2.31	27.16654	22.57048	9.9	9.4	3.4	3.5	68	38	96.3	88.7	28.33333	15.63786	85	111	124	268	2.63013	NA	7.1	Class A
346	42	F	23.37617	0	0	1	0	0	0	1	1	0	0	0	0	0	1	1	1	0	0	180	164	104	92	5.2	5.78	8.0773	7.1949	10.8	11.4	3.9	3.7	72	52	17.9	14.5	27.79923	21.75732	50	50	15100	20500	2.13213	2.92729	NA	Class A
347	50	F	19.97249	0	0	1	0	0	1	0	0	0	0	0	0	0	1	1	0	1	1	128	134	76	103	4.23	4.57	9.57541	8.79884	10.5	11.7	3.2	3.4	63	76	51	75.8	29.85782	37.2549	693	592	557	1360	3.25424	5.63636	5.6	Class C
348	86	F	29.45068	1	0	0	0	1	0	0	1	1	0	0	0	0	1	1	0	0	0	153	137	68	72	1.09	1.35	36.33294	28.75146	10.1	11.1	3.6	3.8	75	75	71.5	56.9	27.47253	26.78571	4680	4130	150	147	1.71053	1.31081	8.1	Class A
349	87	M	21.85406	0	0	0	1	0	1	0	1	0	1	0	0	0	1	1	0	1	1	154	130	89	72	3.91	3.54	12.15512	13.50673	9.6	12.5	3.7	3.7	71	76	27.5	37.5	26.9962	27.94118	463	691	7940	9510	0.65476	1.41935	6.2	Class A
350	84	M	24.08924	0	0	0	1	0	0	1	0	0	0	0	1	0	0	0	0	0	0	120	101	54	64	2.92	1.66	16.84243	31.13582	10.1	9.7	2.8	2.3	72	41	219	202	38.70968	30.37037	18700	53100	1700	2830	1.59494	NA	5.7	Class A
351	65	F	22.96991	1	0	0	0	1	0	0	1	1	0	0	0	0	1	1	0	0	0	161	NA	72	NA	0.78	NA	56.53086	NA	9.8	NA	3.5	NA	NA	48	NA	165	NA	17.45455	NA	659	NA	128	5.66506	NA	NA	Class A
352	87	F	26.1621	0	1	0	0	1	0	0	0	0	0	0	0	0	0	0	0	0	0	122	126	57	64	2.88	2.54	12.50918	14.35212	9.9	11.2	4	4.4	74	79	146	93.5	24.66667	25.23962	3930	576	530	480	0.14851	0.08696	NA	Class A
353	67	M	19.94321	0	0	1	0	0	1	0	0	1	0	1	0	0	0	0	0	0	0	110	135	61	89	3.05	3.86	17.1354	13.18697	8.9	10.1	3	3.3	39	47	244	149	17.48879	22.81553	799	776	7040	13500	5.35802	NA	6.7	Class A
354	78	M	24.92188	0	1	0	0	0	0	1	1	0	0	1	0	0	1	1	0	0	0	162	148	68	67	4.85	8.14	9.87573	5.60464	9.1	10.3	2.97	3.03	93	121	252	196	36.18677	43.68231	431	2450	1970	8190	6.3548	10.09514	NA	Class B
355	41	M	33.14892	1	0	0	0	1	0	0	1	1	0	0	0	0	1	1	0	0	0	158	137	85	82	2.49	3.18	24.63149	18.71862	10.1	11.1	2.4	2.1	52	49	306	382	32.5	33.7931	1270	912	587	1750	8.50438	10.71204	5.6	Class B
356	59	M	25.14861	1	0	0	0	0	0	1	1	1	1	0	0	1	0	0	0	0	0	164	116	70	52	3.88	4.55	13.65794	11.41847	10.6	9.8	3.4	3.6	80	113	114	152	32	48.91775	428	788	7830	4330	7.15142	3.68159	6.1	Class C
357	56	M	17.40326	1	0	0	0	0	0	1	0	1	0	0	0	0	0	0	0	0	0	108	116	58	64	2.21	NA	25.66295	NA	9.7	NA	3.4	NA	135	192	481	278	67.5	91.86603	339	1640	783	372	0.02284	NA	7.5	Class A
358	56	M	25.9635	0	0	1	0	0	1	0	0	0	1	0	0	0	0	0	0	0	0	131	131	85	91	5.52	6.81	9.3796	7.45423	9.3	11	4.3	4.3	65	109	234	159	22.26027	34.9359	1850	414	165	159	2.08071	2.51381	NA	Class A
359	71	M	20.32537	0	0	0	1	0	1	0	0	0	1	1	0	1	1	1	0	0	1	87	129	53	58	2.12	1.4	25.08876	39.34443	11.1	12.3	4.1	4.1	75	48	62.6	48.8	28.30189	19.91701	4800	737	3170	4930	NA	0.35141	NA	Class A
360	77	M	25.88235	0	0	1	0	0	0	0	0	0	0	0	0	0	1	1	0	0	0	126	128	61	53	2.17	2	23.89417	26.02818	10.6	10.3	NA	3.5	123	95	91.7	74.7	53.71179	45.0237	1160	8210	123	334	NA	0.04854	NA	Class A
361	57	F	21.31778	0	1	0	0	1	0	0	0	0	0	0	0	0	1	1	0	0	0	127	120	82	82	1.68	1.51	25.46958	28.62261	10.7	11.9	1.81	4.8	74	99	163	125	24.10423	33.55932	164	241	144	79	0.20979	0.33582	NA	Class A
362	64	F	33.77778	1	0	0	0	0	1	0	0	1	1	0	1	0	1	0	1	0	0	122	116	64	67	1.45	1.47	28.94249	28.51198	10.2	12.1	3.6	4.1	61	73	54.6	52.1	21.25436	22.12121	1070	1610	227	176	2.95838	2.66667	9.7	Class A
363	81	M	20.34061	0	1	0	0	0	1	0	1	0	0	0	0	0	1	1	0	0	1	145	148	64	72	5.35	6.25	8.77501	7.40242	8.9	11.2	4.2	3.8	69	107	145	81	26.33588	37.67606	94	142	848	1270	2.45724	4.5017	5.1	Class B
364	61	F	27.6398	0	0	1	0	1	0	0	0	1	0	0	1	0	1	1	0	0	0	120	150	70	80	3.24	3.33	12.17644	11.81687	10.5	9.5	4.8	4.3	74	39	177	171	24.2623	14.02878	1630	13100	42	111	0.275	0.37778	5.5	Class A
365	87	M	19.59929	0	1	0	0	1	0	0	0	1	0	0	0	0	0	0	0	0	1	126	160	59	85	2.42	2.46	20.47697	20.11299	10.2	13.3	4.1	4.5	76	67	101	50.9	26.29758	16.54321	50	123	581	357	0.11148	0.25532	NA	Class A
366	25	M	22.46753	0	0	0	1	1	0	0	0	0	0	0	0	0	0	0	0	0	0	NA	NA	NA	NA	5.35	NA	12.29628	NA	10.3	NA	3.3	NA	72	NA	147	NA	25.89928	NA	357	NA	989	NA	4.78378	NA	5.6	Class A
367	21	M	18.42404	0	0	1	0	1	0	0	0	0	0	0	0	0	0	0	0	0	0	132	122	84	74	4.05	6.17	17.52948	11.05994	11.7	10.9	1.9	1.9	67	NA	34.3	NA	34.01015	NA	455	NA	368	NA	17.42016	14.78	5.4	Class C
368	58	M	23.75882	1	0	0	0	0	1	0	1	1	0	0	0	0	1	1	0	0	0	156	156	75	81	1.92	1.99	29.63237	28.49395	10.7	11.4	3.8	3.6	79	80	44.6	30.1	27.62238	26.93603	1950	2700	170	194	1.92771	5.18519	7.3	Class A
369	71	M	20.62065	1	0	0	0	0	1	0	0	1	1	1	0	0	1	1	0	0	0	80	111	42	52	2.25	2.17	23.50731	24.35905	9.3	9.7	3.7	3.6	116	126	305	309	39.32203	42.14047	95	50	1710	1550	NA	3.61835	NA	Class A
370	82	F	22.18935	0	0	1	0	0	0	0	1	0	0	0	0	0	0	0	0	0	0	145	151	82	75	2.48	1.83	14.98482	20.89585	7.5	8.4	3.8	3.6	97	NA	147	NA	24.49495	NA	752	NA	2670	NA	0.49837	3.01811	4.9	Class A
371	67	M	NA	1	0	0	0	1	0	0	0	1	0	0	0	0	0	0	0	0	0	130	130	80	80	5.1	6.3	9.7642	7.74889	10	11.1	4.2	4.1	NA	NA	NA	NA	NA	NA	NA	NA	NA	NA	2.00246	1.83673	7.2	Class A
372	76	M	20.26181	1	0	0	0	0	1	0	0	1	1	0	0	0	1	0	1	0	1	138	137	87	69	3.77	3.41	13.10662	14.62766	8.2	8.4	2.7	3	77	52	36	17.9	24.67949	17.86942	408	250	424	392	6.22222	5.50265	6.4	Class A
373	71	M	20.5239	0	0	0	1	0	1	0	0	0	0	0	0	0	1	1	1	1	1	119	124	68	69	2.76	3.27	18.79906	15.55364	9.4	11.9	3.4	3.3	84	52	79.3	26.1	36.52174	20.63492	255	369	611	670	4.73418	NA	5.8	Class A
374	72	M	NA	0	0	1	0	0	1	0	0	1	0	0	0	0	1	1	0	0	0	135	120	64	62	4.14	NA	12.0157	NA	10.2	NA	3.8	NA	88	NA	108	NA	30.55556	NA	915	NA	431	NA	0.60345	NA	6	Class A
375	70	M	21.2295	0	0	1	0	0	1	0	1	0	0	0	0	0	1	1	0	1	1	155	132	83	62	1.93	2.59	27.91637	20.23525	9.4	9.5	3.2	2.5	52	72	163	171	23.85321	37.89474	23400	25100	2600	5180	1.68224	1.61233	5.7	Class C
376	77	M	22.90657	0	1	0	0	0	1	0	1	0	1	0	1	1	1	1	0	0	1	141	127	78	76	4	3.87	12.23842	12.68887	10.3	11.7	3.4	3.4	107	83	93.5	88	49.53704	36.24454	154	148	712	866	0.49313	0.77455	NA	Class A
377	71	M	20.64992	1	0	0	0	0	1	0	1	1	1	0	0	0	1	1	0	0	0	159	126	76	59	4.16	3.89	12.00059	12.91474	10.4	11.6	4.2	4.2	71	62	111	43.8	28.1746	21.01695	2800	1030	1010	1620	1.13752	1.20205	6.1	Class A
378	70	F	39.36011	1	0	0	0	1	0	0	1	1	0	0	0	0	1	0	1	1	1	156	180	86	90	2.53	3.12	15.34225	12.19826	9.1	9.1	3.8	3.5	34	63	36.9	64.7	13.87755	24.51362	3550	687	1840	1440	1.80952	1.27586	6.7	Class B
379	73	M	32.78449	1	0	0	0	0	1	0	1	1	0	0	0	0	0	0	0	1	1	159	154	69	77	3.3	4.93	15.2784	9.84822	9.9	9.4	3.44	2.86	48	58	56.8	85.7	18.3908	25.66372	1690	2000	375	1560	4.34739	7.3002	7.1	Class B
380	85	M	27.45562	0	1	0	0	0	1	0	0	0	0	0	1	0	1	1	0	0	0	128	131	63	65	1.39	1.26	37.80945	42.09719	10.3	10.4	3.4	3.4	55	69	147	82.6	26.69903	30.66667	16800	2900	87	101	0.07516	0.0876	NA	Class A
381	48	F	32.83813	1	0	0	0	1	0	0	1	1	0	0	0	0	1	1	1	1	1	151	137	88	79	2.93	3.31	14.56052	12.74201	10.6	10.6	3.8	3.8	65	72	38.1	40.9	24.5283	29.03226	248	281	471	199	9.36486	3.62745	7.4	Class A

382	79	M	24.27915	1	0	0	0	0	1	0	1	1	0	0	1	0	1	1	0	0	0	144	NA	59	NA	2.15	NA	23.96046	NA	9.4	NA	3.4	NA	88	NA	70.5	NA	30.76923	NA	204	NA	532	NA	1.64566	NA		6	Class A
383	72	M	19.53125	0	1	0	0	0	1	0	1	0	0	0	0	0	0	0	0	1	1	160	198	72	78	2	1.91	26.63302	28.00895	8.4	12.4	4	3.8	41	99	44.3	52.4	12.97468	29.90937	2800	614	1330	1480	1.443	0.39877	NA		Class A
384	70	M	16.12489	1	0	0	0	0	0	1	0	1	0	0	0	1	1	0	1	1	1	130	NA	82	NA	3.68	NA	13.77911	NA	8.2	NA	3.7	NA	79	49	203	392	35.11111	23.11321	459	4130	2050	8970	5	NA		5.8	Class A
385	72	M	24.38029	0	0	0	1	0	1	0	0	0	0	0	0	0	1	1	0	0	0	NA	122	NA	74	2.26	2.31	23.29983	22.74866	11.1	11.5	4.2	4	93	88	252	236	36.75889	34.64567	174	181	106	205	1.45833	2.31959	NA		Class A
386	73	M	25.70895	0	1	0	0	0	1	0	0	0	0	0	0	0	0	0	0	0	1	112	131	59	61	1.5	1.5	36.33994	36.33994	10.5	12.4	3.6	3.9	89	124	18.8	38.6	29.76589	41.47157	678	818	128	54	NA	0.05714		6	Class A
387	80	F	NA	1	0	0	0	1	0	0	0	1	0	0	0	0	0	0	0	0	0	NA	113	NA	60	1.16	1.01	34.65312	40.32104	9.8	10.6	3.8	3.9	90	117	31.3	34.2	27.19033	32.77311	166	294	757	460	0.25641	NA		6.1	Class A
388	63	F	18.49112	0	1	0	0	1	0	0	0	0	0	0	0	0	0	0	0	0	0	NA	141	NA	77	3.97	4.28	9.65959	8.85672	10.2	10.7	4.4	4.2	92	96	54.4	42.6	33.213	35.55556	74	250	213	171	3.2071	3.4029		5.6	Class A
389	63	M	20.14768	0	1	0	0	0	0	1	1	0	0	0	0	0	1	1	0	1	1	159	137	91	71	3.88	3.58	13.40322	14.63669	10.4	12.2	3.9	3.7	50	81	71.8	74.1	19.60784	31.88976	880	988	671	840	0.14706	1.5		5	Class A
390	54	M	23.09753	0	0	1	0	0	0	1	1	1	0	0	0	0	1	1	0	0	0	156	149	78	81	2.15	3.02	26.72496	18.42795	10	10.9	3.6	3.6	91	NA	120	NA	33.09091	NA	258	NA	450	NA	8.37931	6.7766		6.7	Class A
391	74	M	24.99708	0	0	1	0	0	1	0	0	0	0	0	0	0	1	1	0	0	0	134	122	73	73	4.19	4.28	11.76603	11.49563	10.6	12.5	4	4.1	45	81	215	182	17.24138	30.79848	16500	3300	198	268	0.61809	0.35444	NA		Class A
392	75	M	25.31543	1	0	0	0	0	0	1	0	1	0	0	1	0	0	0	0	0	1	126	120	84	58	1.26	1.74	43.47132	30.53848	9.1	10.7	NA	3.3	23	31	98	44.5	11.05769	13.90135	66100	59000	743	432	0.07193	NA		7	Class A
393	54	F	18.94622	0	0	1	0	1	0	0	0	0	1	0	0	0	1	1	0	0	0	126	134	77	81	2.79	2.65	14.85108	15.629	9.9	10.2	4.5	4.1	82	59	25.8	21.1	29.07801	20.84806	335	406	263	213	0.24653	0.34364	NA		Class A
394	82	F	21.32776	1	0	0	0	1	0	0	0	1	0	0	0	0	0	0	0	0	1	127	136	72	70	3.01	3.38	12.12355	10.6794	10.1	8.7	3.9	3.1	41	29	116	138	15.41353	13.24201	2530	70600	88	677	0.7874	1.53509	NA		Class B
395	77	M	23.43963	0	0	1	0	0	1	0	0	0	0	0	0	0	1	1	0	0	0	139	158	79	76	5.19	5.14	9.20419	9.26781	9.6	10.9	4.4	4.5	77	93	180	202	33.18966	39.57447	86	75	610	404	0.17778	1.95652	NA		Class A
396	74	F	23.53506	0	1	0	0	0	0	1	0	0	0	0	0	0	1	1	0	0	0	123	127	72	76	3.75	3.2	9.81717	11.6773	9.7	9.7	4.41	3.95	74	69	94.2	54.2	27.81955	27.93522	827	428	957	1610	0.39343	0.31405	NA		Class A
397	85	M	22.03857	1	0	0	0	0	1	0	1	1	1	0	0	1	1	1	0	0	1	148	NA	85	NA	2.04	2.19	24.84981	22.9939	9.5	9	3.2	3	70	65	36.3	43.5	29.04564	32.0197	170	188	531	802	4.78067	5.46418		6.8	Class A
398	71	F	NA	0	1	0	0	1	0	0	1	0	0	0	0	0	1	1	0	0	0	144	141	73	80	2.74	2.78	14.00348	13.72798	10.3	11.3	4.3	4.3	55	100	55.8	42.3	18.51852	30.8642	1730	302	310	353	2.63333	2.73196	NA		Class A
399	46	M	20.3141	1	0	0	0	1	0	0	1	1	0	0	0	1	0	0	0	0	0	154	125	74	75	4.37	NA	12.87963	NA	10.8	NA	NA	NA	78	58	354	227	27.1777	20.13889	505	675	120	96	NA	NA		6	Class A
400	64	F	20.5046	0	1	0	0	0	0	0	0	0	0	0	0	0	0	0	0	0	0	121	135	63	87	2.28	2.21	17.63972	18.17084	10	11.7	4.1	4.2	75	60	101	82.3	28.08989	23.80952	216	119	357	364	1.69779	2.82855		6	Class A
401	80	F	18.96353	0	0	0	1	1	0	0	0	0	0	0	0	0	1	0	1	0	0	134	161	81	101	1.9	2.04	20.19775	18.68633	9.3	9.8	2.9	3	68	70	46.5	27.5	27.2	25.54745	283	131	383	667	4.07692	3.59458		5.3	Class A
402	67	F	30.80942	0	0	0	1	0	0	1	1	0	0	0	0	0	1	1	0	0	0	144	149	80	87	2.85	2.74	13.63837	14.23848	9	9.1	3.6	3.3	48	36	226	195	18.89764	15.31915	4020	7470	508	626	4.74596	4.34834	NA		Class B
403	63	M	26.7576	0	0	1	0	0	0	0	0	1	0	0	0	0	1	1	0	0	0	123	158	71	111	3.59	4.27	14.5921	12.06988	9.9	11.2	3.8	3.7	92	91	82.2	54.6	35.65891	32.61649	3200	1130	1070	1650	NA	3.89485		6.7	Class A
404	72	M	25.94787	0	0	0	1	0	0	1	0	1	0	0	0	1	1	1	0	0	0	129	129	71	87	4.16	5.17	11.95251	9.42299	10.5	12.2	3.4	3.4	98	121	229	154	42.79476	54.5045	1900	1580	691	999	4.69571	6.15759		5.4	Class B
405	60	F	26.27574	0	0	0	1	1	0	0	1	1	0	0	0	0	1	1	0	0	0	139	130	91	90	1.9	1.87	21.93615	22.32143	11.2	12.1	3.7	3.4	87	117	97.7	80.9	31.86813	43.65672	928	1390	409	297	1.93976	3.01923		6.1	Class A
406	59	F	30.53042	1	0	0	0	1	0	0	1	1	1	0	0	0	1	1	0	1	1	151	145	75	71	1.71	2.24	24.73511	18.32084	8.2	9.8	2.4	3	33	35	81.2	56.6	15.49296	15.21739	2020	3370	2420	1290	11.98044	10.89172		5.4	Class A
407	85	M	23.61275	0	1	0	0	0	1	0	1	0	0	0	0	0	1	1	0	0	0	140	145	60	55	3.23	3.23	15.03111	15.03111	10.7	10.8	4	NA	63	102	210	197	21.875	40.8	1620	1170	1690	1800	0.77576	NA		5.7	Class B
408	74	F	16.75591	0	0	0	1	1	0	0	0	0	0	0	0	0	0	0	0	0	0	138	137	77	71	1.37	1.54	29.53958	25.99136	9.4	9.9	3.9	3.8	80	76	20.1	28.9	27.97203	26.11684	492	2500	233	175	1.89655	0.91852		5.5	Class A
409	60	M	23.67394	1	0	0	0	1	0	0	0	1	0	0	0	0	1	1	0	0	0	114	156	60	78	5.08	4.45	10.12179	11.69948	9.4	12.7	3.2	3.6	50	71	190	44.7	17.85714	20.4611	308	376	214	263	4.84674	5.05721		7.1	Class B
410	84	M	NA	0	0	0	1	0	0	0	0	0	0	0	0	0	0	NA	NA	NA	0	NA	NA	NA	NA	3.83	3.41	12.5174	14.21348	9.8	9.5	NA	3.5	NA	NA	NA	NA	NA	NA	NA	NA	NA	NA	NA	NA	NA		Class A
411	72	M	20.68747	1	0	0	0	0	1	0	0	1	0	0	0	0	1	0	1	0	0	114	160	66	80	3.81	4.91	13.2117	9.97021	10.4	12.1	3.3	2.4	NA	NA	NA	NA	NA	NA	NA	NA	NA	NA	7.19398	13.25704		5.7	Class A
412	82	M	27.38266	0	0	1	0	0	0	0	0	1	0	0	1	1	1	1	0	0	0	120	150	70	70	1.42	0.83	37.31932	67.15305	7.8	9.4	3.6	3.4	58	58	77.1	71.4	20.13889	22.1374	5990	363	2220	3660	0.22599	0.91566		6.7	Class A
413	86	M	25.29938	1	0	0	0	1	0	0	1	1	0	0	0	0	0	0	0	0	0	140	132	70	70	3.39	3.45	14.20896	13.89267	10.1	10.5	4.1	3.9	75	69	199	122	32.18884	32.85714	239	349	214	274	0.12727	0.13846		6.1	Class A
414	77	M	23.04367	0	0	0	1	0	1	0	0	0	0	0	1	0	0	0	0	0	0	101	113	55	71	1.49	1.39	36.05065	38.89741	10.3	10.6	4.1	4.1	80	72	93.9	127	27.11864	24.65753	902	6690	261	149	0.38051	0.42551	NA		Class A
415	75	F	23.04688	0	1	0	0	1	0	0	1	0	0	0	0	0	0	0	0	0	0	146	117	73	63	1.9	2.01	20.57535	19.2733	10	11.3	3.9	4.1	59	93	49.2	39.8	23.6	34.06593	50	50	407	439	0.40059	0.29772	NA		Class A
416	65	F	30.496	1	0	0	0	1	0	0	0	1	0	0	0	0	1	1	0	0	0	119	129	50	67	2.53	2.44	15.67206	16.30555	9.1	9.9	4	3.8	66	64	56.4	38.9	23.65591	22.37762	891	330	413	597	4.32703	7.54386		6.7	Class B
417	79	M	22.00184	0	1	0	0	1	0	0	0	0	0	0	0	0	0	0	0	0	0	114	147	64	76	1.8	1.82	29.10145	28.75177	10.9	11.7	4.1	4.1	71	88	145	124	29.46058	35.2	50	72	1580	1850	NA	NA		4.7	Class A
418	81	F	20	0	0	0	1	1	0	0	1	1	0	0	0	0	0	0	0	0	0	152	NA	68	NA	2.04	NA	18.61982	NA	10.1	NA	3.3	NA	58	NA	276	NA	25.21739	NA	410	NA	2680	NA	7.1875	NA	NA		Class A
419	74	M	25.57214	1	0	0	0	0	1	0	0	1	0	0	1	0	1	1	0	0	0	137	134	71	63	3.94	4.34	12.58518	11.32188	9.6	10.2	4.1	4.2	59	58	192	199	26.94064	25.66372	2070	4560	619	821	2.09281	2.40838		5.2	Class B
420	52	M	NA	1	0	0	0	1	0	0	1	1	0	0	0	0	1	1	0	0	0	140	NA	76	NA	6.67	NA	7.82916	NA	10	NA	3.8	NA	90	91	40.1	71.3	31.80212	31.27148	210	63	843	120	4.54545	NA	NA		Class C
421	71	M	22.24065	0	0	1	0	0	1	0	0	0	0	0	0	0	1	1	0	0	0	136	157	52	70	2.75	3.19	18.87386	16.04514	9.1	9.5	3.6	4	50	65	392	177	22.02643	27.19665	12500	1210	825	1420	NA	3.07636	NA		Class A
422	73	M	21.22449	1	0	0	0	0	1	0	1	1	0	0	1	1	1	0	1	0	0	171	140	73	58	3.95	3.38	12.59943	14.94147	7.1	10.2	2.9	3.3	81	24	992	412	52.5974	15.89404	4600	27900	2390	13600	NA	NA		7.2	Class A
423	81	F	18.61073	0	1	0	0	1	0	0	1	0	0	0	0	0	1	1	0	0	1	140	166	49	81	1.74	2.07	22.159	18.26039	10.2	11.8	3.5	4	90	62	44	13.1	34.74903	17.51412	150	273	598	538	0.05967	0.94972	NA		Class A
424	78	M	23.99946	0	1	0	0	0	1	0	0	0	1	0	1	0	1	1	0	0	0	109	127	49	54	2.41	3.02	21.22482	16.52172	10.6	10.8	3.8	4.1	79	102	87.7	80.5	27.43056	30.0885	726	519	496	451	1.00945	0.72915	NA		Class A
425	59	M	30.48214	1	0	0	0	0	1	0	0	1	1	1	1	0	1	1	0	0	0	137	143	71	84	3.88	5.33	13.65794	9.65001	10.2	12	2.5	2.9	71	78	147	118	33.02326	33.47639	148	156	1410	812	12.22906	10.77859		8.1	Class B
426	77	M	20.41196	0	1	0	0	0	0	1	0	0	0	0	0	0	1	0	1	0	0	122	96	67	64	2.64	3.43	19.28165	14.47995	8.7	11.8	3.5	3.9	93	93	136	23.8	34.44444	25.76177	575	146	494	148	0.14199	0.14733	NA		Class A
427	66	F	21.10299	1	0	0	0	0	1	0	0	1	0	0	0	0	1	1	0	0	0	135	121	74	55	1.18	1.02	35.94166	42.153	9.4	11.4	3.7	3.7	67	89	81.7	65.7	22.40803	28.80259	1170	666	290	259	1.36573	1.37688		6.9	Class A
428	86	F	NA	0	1	0	0	1	0	0	0	1	0	0	0	0	0	0	0	0	1	NA	NA	NA	NA	1.45	1.29	26.5894	30.2176	9.1	10.7	3.6	3.6	62	85	24.7	55.2	20.46205	27.15655	285	147	445	658	0.13333	0.16279		6.8	Class A
429	64	M	24.4796	0	0	0	1	0	0	1	0	0	0	0	0	1	1	1	0	0	0	134	135	75	81	4.13	4.36	12.46174	11.69223	10.1	12.6	4.3	4.7	66	97	184	151	22.91667	30.50314	208	385	210	137	0.3866	0.57692	NA		Class B
430	73	M	23.2998	0	1	0	0	1	0	0	0	0	0	0	0	0	0	0	0	0	0	120	137	81	82	3.24	3.11	15.5882	16.30243	10.3	10.7	4.5	4.4	88	85	229	206	30.13699	31.71642	857	410	199	147	NA	0.09032	NA		Class A
431	68	F	18.11396	0	0	0	1	1	0	0	0	0	0	0	0	0	0	0	0	0	0	131	142	76	89	1.8	1.18	22.45164	35.48604	9.4	11.9	3.3	3.5	69	85	436	227	36.8984	35.12397	1230	149	382	171	1.22857	0.65405	NA		Class A
432	73	F	28.53333	0	1	0	0	1	0	0	0	1	0	0	0	0	1	0	1	0	1	130	105	64	59	1.29	1.21	31.67287	33.97076	8.9	11.2	4.1	3.9	28	71	19.9	45.3	7.671233	20.0565	842	479	484	380	0.04627	0.06542		5.4	Class A
433	83	F	23.57083	0	1	0	0	0	1	0	0	0	0	0	0	0	0	0	0	0	0	103	105	59	41	2.92	3.27	12.48941	10.99671	10.5	11.7	4.3	4.5	63	59	56.5	44.4	22.5	18.97106	13200	3170	559	726	0.11111	0.1588		5.4	Class A
434	88	F	23.56345	1	0	0	0	1	0	0	0	1	0	0	0	0	0	0	0	1	1	134	160	73	83	2.13	1.71	17.34332	22.05372	10.2	12	3.3	3.7	58	124	440	442	29.89691	58.49057	181	85	658	289	0.74684	1.98737	NA		Class A
435	76	M	20.19509	0	0	1	0	1	0	0	0	0	1	0	0	0	1	1	0	0	0	127	131	80	74	2.6	2.34	19.6801	22.08442	9.9	10.6	4	4	81	122	43.4	23.6	28.125	43.41637	181	195	244	341	0.34922	0.75421		5.9	Class A
436	77	F	13.17978	0	0	0	1	0	1	0	0	1	0	0	0	0	0	0	0	0	0	120	NA	64	NA	1.64	1.61	23.89879	24.38639	10.4	10.1	3.8	3.1	NA	74	NA	133	NA	30.57851	NA	81	NA	249	NA	NA		6.2	Class A
437	83	M	28.125	1	0	0	0	0	1	0	0	1	1	1	0	1	0	0	0	0	0	118	119	63	69	2.69	2.66	18.48746	18.65147	10.4	11.8	4	4.1	69	91	73.7	78.6	26.43678	32.73381	579	455	2800	3160	1.82891	1.89093		7	Class A
438	64	M	21.8615	1	0	0	0	0	0	1	0	1	1	1	1	0	1	1	0	1	1	NA	130	NA	70	6.18	6.07	8.01838	8.14118	9.2	11.4	3.9	4.6	58	NA	82.1	NA	18.70968	NA	2140	NA	14700	NA	4.83798	3.81271		4.5	Class C
439	69	M	28.5423	0	0	0	1	1	0	0	1	0	0	0	0	0	1	1	0	0	0	140	146	71	49	6.31	6.47	7.67043	7.4324	9.8	9.5	4.3	4	56	48	82.2	81.2	19.85816	18.89764	6030	9560	383	408	0.89985	0.72148	NA		Class A
440	43	M	NA	0	0	0	1	0	1	0	0	0	0	0	0	0	0	0	0	0	0	NA	130	NA	80	3.33	4.23	17.67837	13.60755	10.6	11	3.8	4	85	104	185	174	29.51389	35.61644	4730	1490	135	125	NA	NA	NA		Class A
441	71	M	21.53305	0	1	0	0	0	1	0	1	0	0	0	1	0	0	0	0	0	0	150	148	91	85	3.23	3.02	15.82789	17.03582	9.6	10.3	4.2	3.9	42	53	192	277	15.05376	23.04348	4010	5370	1160	923	1.30802	0.8991	NA		Class A
442	76	M	19.764	0	0	1	0	0	1	0	1	0	0	0	0	0	0	0	0	0	0	160	148	75	73	3.28	3.99	15.26308	12.3181	10.6	9.8	4.4	4.2	96	112	18.9	26.4	25.06527	31.46067	72	62	859	1050	0.59901	0.70363	NA		Class B
443	77	F	17.3329	0	0	0	1	1	0	0	0	0	0	0	0	0	0	0	0	0	1	128	153	52	74	1.18	1.04	34.38622	39.48106	10.3	10.4	4.1	3.9	66	58	3.8	9	16.92308	16.57143	157	183	93	227	0.16667	0.19149	NA		Class A
444	72	M	19.38349	0	1	0	0	0	0	1	0	0	0	0	0	0	0	0	0	0	0	132	126	70	88	4.34	4.1	11.41126	12.144	10.6	11.6	4	3.9	67	26	175	147	26.17188	10.78838	1060	14600	281	308	0.50784	0.60413		5.6	Class A
445	81	M	21.33821	0	1	0	0	1	0	0	1	0	0	0	0	0	1	1	0	0	0	161	164	73	87	2.51	2.26	20.08276	22.52537	10.5	12.9	3.8	3.9	82	96	104	93.9	34.59916	37.5	900	1710	238	167	0.14764	0.58939		5.4	Class A
446	57	F	31.89517	1	0	0	0	1	0	0	0	1	0	0	0	0	0	0	0	0	0	NA	130	NA	64	1.17	1.2	37.83683	36.80322	10.1	10.1	3.6	3.3	NA	NA	NA	NA	NA	NA	NA	NA	NA	NA	0.000862	NA	NA		Class A
447	59	F	35.74951	1	0	0	0	1	0	0	1	1	0	0	0	0	1	1	0	0	0	153	133	76	74	2.21	2.45	18.68299	16.60998	10.8	11.4	3.2	3.1	56	56	43.3	22.8	18.30065	18.00643	499	466	384	558	6.97638	6.39906	NA		Class A
448	63	F	21.32643	0	0	1	0	1	0	0	0	0	0	0	0	0	0	0	0	0	0	130	152	71	95	4.41	4.94	8.61033	7.60498	9.7	11.3	4.1	4.3	60	81	99.7	58.1	24.69136	30.22388	720	351	210	212	0.56066	0.59138	NA		Class A
449	78	M	21.32647	0	1	0	0	0	0	1	0	0	0	0	0	0	0	0	0	0	0	132	128	75	83	3.99	6.1	12.22661	7.65653	10	12.9	3.3	3.8	61	82	60.6	23.4	24.89796	28.17869	2100	1150	1730	1430	1.48889	1.21008	NA		Class B
450	83	F	20.22027	0	1	0	0	1	0	0	0	0	1	0	0	0	1	1	0	0	1	118	123	60	62	1.46	1.51	26.56905	25.60809	10.7	13	4.4	4.2	105	160	14.1	20.1	31.06509	51.77994	63	51	163	208	0.03521	0.12821	NA		Class A
451	68	M	NA	1	0	0	0	1	0	0	1	1	0	0	1	0	0	0	0	0	0	140	140	50	70	2.75	2.76	19.10916	19.03343	9.3	8.6	3.4	3	92	41	12	10.5	17.49049	8.541667	1940	1470	201	317	4.78469	4.78469		5.6	Class A
452	67	M	19.66412	0	1	0	0	0	1	0	1	1	0	0	0	0	1	1	0	0	1	143	144	71	84	4.07	4.44	12.44444	11.31448	10.1	13.5	3.81	4.17	55	83	83	36.1	23.91304	30.62731	695	475	345	255	1.08434	1.95822		6.9	Class A
453	86	M	19.69124	0	0	0	1	0	1	0	0	0	0	0	0	1	1	1	0	0	0	124	110	69	70	1.92	2.24	26.46483	22.35781	10.4	10	3.4	3.5	57	51	119	141	25.79186	23.18182	7560	5040	1280	714	0.9203	0.40936		5.4	Class A
454	80	F	28.10935	0	1	0	0	1	0	0	1	0	0	0	1	0	1	1	0	0	1	168	118	72	54	2.42	2.35	15.5012	16.00704	10.8	10	4.4	3.7	81	58	37.6	166	25.39185	21.32353	428	374	297	266	0.37697	0.20468	NA		Class A
455	74	M	18.0998	1	0	0	0	0	1	0	0	1	1	0	1	0	1	1	0	0	0	130	160	57	71	1.7	1.8	31.56611	29.65269	10.5	11.5	3.3	3.2	30	56	71.7	55.7	12.76596	22.31076	7550	8070	1200	521	3.6098	3.49206		8.2	Class A
456	71	M	26.7326	1	0	0	0	0	0	1	1	1	0	0	0	0	0	0	0	0	0	146	142	68	65	4.41	4.69	11.25835	10.52513	10.3	9.9	4.3	4.2	38	58	107	169	17.59259	27.61905	713	1310	328	426	2.18692	1.87619		5.2	Class A
457	82	M	25.29938	0	1	0	0	1	0	0	1	0	1	0	0	0	0	0	0	1	1	157	122	68	73	5.42	5.76	8.62069	8.06557	8.5	11.8	2.5	3.6	26	62	170	245	15.95092	33.87978	3540	4040	15400	2690	2.73333	5.49932	NA		Class B
458	63	M	22.68848	0	0	1	0	0	1	0	0	0	1	0	0	0	0	0	0	0	0	116	NA	67	NA	2.02	2.01	27.37389	27.52292	10.8	12.3	4.5	4.4	67	124	64.9	48.3	23.10345	43.66197	558	478	271	156	0.06061	0.04		5.9	Class B
459	62	M	23.80869	1	0	0	0	1	0	0	1	1	1	1	0	0	0	0	0	0	0	178	137	89	82	2.88	3.47	18.65563	15.14504	11	11.8	3.8	3.5	71	99	71	36.7	20.28571	29.03226	175	74	1300	1530	8.37178	8.02979		6.2	Class B
460	67	M	24.67702	1	0	0	0	1	0	0	0	1	1	0	0	0	1	1	0	0	0	129	140	68	83	4.77	6.4	10.50556	7.61653	9.5	10	3.9	4	70	52	159	146	29.91453	20.23346	424	9160	4360	22500	2.8589	4.15947	NA		Class A
461	87	M	21.55471	0	0	0	1	0	1	0	0	1	1	1	0	1	0	0	0	0	1	120	88	66	41	1.82	1.92	27.96672	26.37717	10.5	10.1	3.6	3.3	59	83	45.7	42.2	18.78981	26.77419	1850	1720	3730	5890	0.06012	0.19126		6.7	Class A
462	65	F	19.68277	0	0	1	0	1	0	0	0	0	0	0	1	0	1	1	0	0	0	134	125	84	75	1.17	1.15	36.43718	37.131	9.7	11	4.3	4.6	63	87	135	112	22.58065	27.61905	591	469	194	288	0.26531	0.19672	NA		Class A
463	86	F	17.94182	0	1	0	0	1	0	0	0	0	0	0	0	0	0	0	0	0	0	123	130	60	62	2.23	2.27	16.6035	16.28369	8.7	10.1	3.9	4	34	52	141	65.3	15.45455	25.36585	204	89	849	679	0.11844	0.17991		5.3	Class C
464	65	M	25.35693	1	0	0	0	0	0	1	1	1	0	0	0	0	1	1	0	0	0	163	180	84	92	2.81	2.86	18.90649	18.54518	9.3	12.6	2.65	3.14	39	80	76.1	61.2	15.78947	29.5203	936	708	17600	24700	9.36037	11.19821		5.9	Class A
465	84	F	26.39715	0	0	1	0	1	0	0	0	0	0	0	0	1	0	0	0	0	1	117	110	63	57	2.29	1.63	16.1824	23.47306	9.3	10.2	3.6	3.1	29	66	186	146	8.841463	22.91667	11700	1390	283	793	0.03361	0.05698	NA		Class A
466	74	M	23.30668	0	1	0	0	0	0	1	1	1	0	0	0	0	1	1	0	0	1	141	137	70	65	3.15	2.31	16.07609	22.48369	7.2	10.6	4.2	4	36	67	60.4	180	12.5	26.17188	10300	3550	495	373	0.3	0.67089		7.3	Class A
467	75	M	24.71902	1	0	0	0	0	1	0	0	1	0	0	0	0	0	0	0	0	0	117	117	67	67	3.06	3.52	16.53027	14.18213	10	11.5	4	4.2	53	70	191	169	17.37705	21.60494	7800	6570	367	170	2.5071	1.45038		5.4	Class B
468	65	F	29.71347	1	0	0	0	1	0	0	0	1	1	1	0	0	0	0	0	0	0	119	117	63	57	2.86	3.24	13.70489	11.95649	9.6	9.8	3	2.8	48	NA	12.6	NA	13.00813	NA	968	NA	1300	NA	9.65217	7.64865		7.9	Class A
469	67	F	24.33604	0	0	0	1	1	0	0	0	0	0	0	0	0	1	1	0	0	1	138	174	70	82	5.37	5.65	6.81979	6.45092	8	10.2	4.3	NA	86	151	47.1	39.4	34.53815	47.33542	190	268	1050	912	1.32007	2.85412	NA		Class B
470	74	M	25.8783	1	0	0	0	0	0	1	0	1	0	0	0	0	0	0	0	0	0	136	NA	72	NA	2.86	2.84	17.86766	18.00537	10.2	12.5	3.8	3.8	93	95	335	237	39.40678	38.46154	445	1200	498	680	1.2807	2.51316		6.4	Class A
471	40	M	18.3556	1	0	0	0	0	0	1	0	1	0	0	0	0	1	1	0	0	0	110	110	80	80	2.82	5.01	21.64901	11.54484	9.9	9.6	1.4	1.5	31	41	148	135	19.0184	23.97661	8880	2590	1020	1130	19.26059	23.77551		7.2	Class B
472	86	M	18.14415	0	1	0	0	0	1	0	0	0	0	0	0	0	1	1	0	0	0	119	128	63	66	2.36	NA	21.11713	NA	10.5	NA	3.7	NA	126	132	59.8	56.3	37.61194	42.30769	1300	2670	412	470	4.48819	NA	NA		Class A
473	67	F	24.45672	1	0	0	0	1	0	0	0	1	0	0	0	0	1	1	0	1	1	133	143	67	75	4.46	4.43	8.35584	8.41777	10.5	11.4	4.3	4.2	63	80	111	83.6	24.60938	30.41825	156	159	1640	1120	3.39787	4.2801		5.4	Class A
474	79	F	16.52666	0	1	0	0	1	0	0	0	0	0	0	0	0	0	0	0	0	0	118	139	56	64	3.33	4.23	10.97168	8.44522	9	10.3	4.11	4.38	60	90	77.2	60.9	25.75107	38.62661	50	52	1010	1260	1.49785	1.90153		5.1	Class C
475	86	F	NA	0	0	1	0	1	0	0	0	1	0	0	1	0	1	1	0	0	0	NA	NA	NA	NA	0.94	0.74	42.72118	55.50161	10.5	9.6	3.4	3.2	42	NA	35	NA	16.09195	NA	7090	NA	258	NA	0.05882	0.17391		5.9	Class A
476	72	M	23.03445	1	0	0	0	0	1	0	1	1	0	0	0	0	1	0	1	1	1	150	160	84	78	1.94	NA	27.53545	NA	8.6	NA	3.3	NA	47	52	151	44.5	20.25862	19.92337	3750	360	895	559	3.5215	NA		6.4	Class A
477	67	M	20.64398	1	0	0	0	1	0	0	0	1	0	0	0	0	1	1	1	1	1	133	132	74	73	3.2	2.75	16.25864	19.19059	8.7	12.5	4	4.3	63	120	84.3	55.8	19.09091	35.92814	184	428	238	218	1.28521	2.79391		6	Class B
478	69	F	18.83474	0	0	0	1	1	0	0	0	0	0	0	0	0	1	1	0	0	0	121	125	75	73	1.19	1.14	35.15994	36.85043	9.8	10.6	3.7	3.8	56	71	43.4	52.3	21.37405	26.59176	170	156	287	265	1.48599	3.49548	NA		Class A
479	69	M	25.06754	1	0	0	0	0	0	1	1	1	0	0	0	0	1	1	0	0	0	140	151	52	58	2.23	2.81	23.93352	18.5852	10.7	10.4	2.5	2.9	54	70	426	328	23.68421	33.49282	2570	3340	1210	1310	12.73191	6.17792		6.6	Class B
480	73	M	22.44031	0	1	0	0	0	1	0	0	1	0	0	1	0	1	1	0	0	0	117	121	66	67	1.98	1.76	26.82109	30.39075	9.4	11	3.9	3.7	64	98	141	91.9	29.22374	39.8374	64	96	256	181	0.000713	0.000661	NA		Class A
481	67	F	21.8144	0	0	0	1	1	0	0	0	0	0	0	0	0	1	1	0	0	0	113	124	68	67	1.76	1.78	23.1085	22.8246	10.6	11	4.3	4.5	98	71	118	98.2	28.3237	22.32704	326	398	224	184	1.50956	1.15327	NA		Class A
482	79	F	17.07451	0	1	0	0	1	0	0	0	0	0	1	0	0	1	1	0	1	1	122	128	47	54	2.18	2.14	17.44037	17.73318	8.1	9.7	3.4	3.3	84	63	364	345	45.40541	37.05882	999	741	6460	8250	1.74242	3.53846		5.1	Class A
483	73	M	22.94213	0	1	0	0	0	1	0	1	0	0	0	1	0	1	1	0	0	0	142	139	78	85	2.44	2.76	21.34143	18.64977	10.6	11.1	3.5	3.8	76	141	22.3	15.6	26.57343	44.7619	6070	4430	291	538	0.6338	0.52841		5.5	Class A
484	78	M	22.94812	0	0	1	0	0	1	0	0	1	0	0	0	1	1	1	0	0	0	124	155	73	76	4.4	4.23	10.98584	11.46976	9.6	11.1	3.3	3.6	52	86	121	74.2	20.23346	33.33333	3220	581	140	204	2.77038	2.4639		6.9	Class A
485	77	F	23.43946	0	1	0	0	1	0	0	1	0	1	0	0	0	0	0	0	0	0	143	168	72	82	1.74	1.61	22.48342	24.38639	9.7	10.6	3.9	3.8	56	100	30.1	22	17.72152	28.73563	154	135	274	178	0.262	0.22219	NA		Class A
486	53	F	22.99996	0	0	1	0	1	0	0	0	0	0	0	0	0	1	1	0	1	1	NA	NA	NA	NA	2.4	2.59	17.60467	16.11014	8.5	10.5	3.8	4.1	73	36	243	251	33.64055	17.3913	868	5500	152	197	0.22	0.2439		5	Class B
487	60	F	22.51304	0	1	0	0	1	0	0	1	0	0	0	1	0	1	0	1	0	0	134	151	91	88	1.6	1.82	26.47339	22.88434	10	12.7	3.8	3.6	51	79	70.7	21.6	18.95911	27.8169	604	188	114	91	1.76636	1.23881	NA		Class B
488	70	M	19.58543	1	0	0	0	0	1	0	1	1	0	0	0	0	0	0	0	0	0	180	156	60	60	1.88	2.42	28.72962	21.79539	10.4	11.2	2.7	NA	NA	NA	NA	NA	NA	NA	NA	NA	NA	NA	3.33333	7.19231		6	Class A
489	88	F	20.86219	1	0	0	0	1	0	0	1	1	1	0	0	1	0	0	0	0	0	154	140	74	80	2.37	NA	15.43139	NA	9	NA	3.8	NA	75	64	123	44.4	33.93665	27.23404	453	2530	1270	1100	0.31211	NA	NA		Class A
490	70	F	30.27921	0	1	0	0	1	0	0	0	0	0	0	0	0	1	1	0	0	0	136	137	74	74	1.33	1.32	31.0034	31.26044	11.1	11	4.5	4	98	94	18.7	18.7	22.8972	23.38308	629	481	82	95	0.001661	0.001116	NA		Class A
491	75	F	19.41486	0	0	0	1	1	0	0	0	0	0	0	0	0	1	1	0	0	0	112	126	57	73	1.99	2.05	19.55953	18.93411	10.2	11.1	3.9	4	96	95	44	32.8	40.67797	36.82171	143	465	150	250	NA	0.85912		5.7	Class A
492	63	M	NA	1	0	0	0	0	0	1	1	1	0	0	0	0	1	1	0	1	1	142	120	70	64	3.27	3.99	16.16128	12.94088	10.1	9.7	3.9	3	103	73	89.2	108	35.27397	28.51563	72	145	650	987	5.55556	17.24138		6.4	Class A
493	54	F	23.13852	1	0	0	0	0	1	0	1	1	0	0	0	0	1	1	0	0	1	158	156	70	73	2.86	3.07	14.45389	13.3758	9.9	10	4	4	49	83	32.7	51.1	17.5	26.51757	600	203	7720	590	4.12557	5.47205		6.6	Class A
494	65	M	30.62824	1	0	0	0	0	1	0	0	1	0	0	0	0	1	1	1	0	0	115	145	55	79	3.25	2.55	16.12483	21.02522	10.4	12.4	3.8	3.9	46	65	113	111	17.35849	20.90032	805	3190	1040	335	0.3557	1.6		6.8	Class A
495	65	M	29.89243	1	0	0	0	0	0	1	1	1	1	0	1	0	1	1	0	0	0	144	115	74	64	3.26	4.55	16.07073	11.15915	10.7	12.1	3.6	3.7	55	62	103	56	20.67669	22.96296	1170	1720	4170	2800	2.86494	2.30361		8.5	Class A
496	37	M	18.85813	0	0	1	0	1	0	0	0	0	0	0	0	0	1	0	1	0	0	110	136	70	86	3.43	3.34	17.7334	18.25682	9.4	11.3	4.2	4.2	61	68	58.3	9.8	21.8638	20.92308	50	110	64	43	1.05479	2.55		5.7	Class A
497	69	F	21.18338	0	0	1	0	1	0	0	0	0	0	0	0	0	1	1	0	0	0	119	154	71	85	2.93	2.86	13.12029	13.472	9.6	11.3	3.6	3.7	61	125	32.8	27.6	24.11067	46.81648	361	241	263	329	1.47059	2.39482		6.2	Class A
498	78	F	19.15152	0	0	0	1	0	1	0	0	0	0	0	1	0	0	0	0	1	1	136	150	80	84	1.91	1.51	20.15472	26.06312	10.3	13	4	4.4	46	146	7.6	29.2	12.39892	39.45946	303	383	321	299	0.20988	1.40476	NA		Class A
499	72	M	NA	1	0	0	0	0	1	0	0	1	0	0	1	0	1	1	0	0	0	NA	156	NA	70	3.6	3.85	14.00078	13.00928	10	11.3	3.1	3.7	49	65	97.8	53.2	23.00469	23.80952	1900	285	1630	1710	2.5	4		8.5	Class A
500	69	M	19.08396	0	0	1	0	0	1	0	0	0	0	0	0	0	0	0	0	0	0	125	107	56	52	2.45	2.93	21.59257	17.75411	9.5	11.1	3.9	3.8	93	75	163	18.8	39.07563	24.83444	840	2240	379	256	0.36384	NA		5.6	Class A
501	78	M	19.12326	1	0	0	0	0	0	1	0	1	0	0	0	1	0	0	0	0	0	110	110	53	53	1.25	1.23	43.52623	44.30109	10.8	11.3	4.1	4	70	51	20.5	21.6	24.64789	17.83217	1570	3110	61	107	0.44048	0.39691		6.8	Class A
502	79	M	23.32415	0	0	0	1	0	1	0	0	0	0	1	0	0	0	0	0	0	1	108	106	52	58	2.8	2.92	17.947	17.1417	9.4	11.1	4	3.9	51	60	25.5	45.3	15.88785	21.73913	1010	944	959	1230	0.12915	0.10174	NA		Class B
503	51	M	19.9838	0	0	0	1	1	0	0	0	0	1	0	0	0	0	0	0	0	0	112	144	76	90	6.96	6.67	7.47299	7.82916	10.2	11.3	4	3.9	99	112	43.1	71.1	37.5	47.25738	122	54	140	260	NA	0.49347	NA		Class A
504	73	M	23.42356	0	1	0	0	1	0	0	1	0	0	0	0	0	0	0	0	0	0	164	126	90	75	2.72	NA	18.95002	NA	9.4	NA	3.6	NA	NA	37	NA	53.2	NA	8.958838	NA	2800	NA	9270	0.67751	NA		6	Class A
505	72	M	20.43733	0	1	0	0	0	1	0	0	1	0	0	0	0	1	0	1	0	0	122	138	46	64	1.8	1.63	29.88678	33.31302	9.8	11.4	4.4	4.2	62	65	74.4	12.7	20.32787	17.47312	12100	1070	984	1110	1.5678	0.93867		5.5	Class A
506	75	M	20.54569	1	0	0	0	1	0	0	0	1	0	0	0	0	1	1	0	0	0	118	130	61	80	2.49	NA	20.71176	NA	6.6	NA	4.2	NA	104	115	235	109	38.66171	50.21834	343	1410	199	151	0.4	NA		6.7	Class A
507	67	F	18.44444	0	0	0	1	1	0	0	0	0	0	0	0	0	1	1	0	0	0	114	116	71	76	3.72	NA	10.19034	NA	9.4	NA	4.3	NA	104	116	157	121	38.37638	43.28358	811	794	478	223	0.61115	NA	NA		Class A
508	92	F	20.64177	0	0	0	1	1	0	0	1	0	0	0	0	0	1	1	0	0	0	182	170	97	76	2.82	3.31	12.59698	10.57176	9.7	10	3.6	3.5	31	43	21.3	17.1	10.50847	13.19018	1290	713	3400	5600	NA	NA	NA		Class A
509	65	M	23.80165	1	0	0	0	1	0	0	0	1	0	0	0	0	1	1	0	0	1	139	138	55	61	3.72	4.27	13.90982	11.9621	9.4	10.8	3.9	3.9	87	93	134	36.1	24.4382	24.09326	466	609	666	853	6.76908	6.39127		5.7	Class A
510	76	F	24.65483	1	0	0	0	1	0	0	0	1	0	0	1	0	1	1	0	0	0	114	126	52	60	3.26	2.97	11.35514	12.57353	9.1	11.1	3.7	3.9	68	75	72.9	36	24.46043	23.00613	129	156	216	187	1.09272	2.55172		6.6	Class A
511	88	F	20.45323	0	1	0	0	1	0	0	0	0	0	0	0	0	1	1	0	0	0	111	129	49	60	5.32	5.24	6.37136	6.45688	8.1	10.4	4.1	4.1	225	101	524	287	87.2093	42.79661	308	173	1030	2000	1.27119	1.63594		6.2	Class B
512	84	F	30.46362	0	0	0	1	1	0	0	1	1	0	0	0	0	0	0	0	1	1	140	122	70	68	3.57	3.68	9.98985	9.66363	10.4	9.8	4.3	3.8	69	68	46.6	44.8	22.25806	26.05364	711	1040	897	978	3.083	1.42857		5.2	Class A
513	81	F	23.28857	0	0	1	0	0	1	0	0	0	0	0	1	0	1	1	0	0	0	106	113	59	62	1.82	1.73	21.09565	22.29916	10	10.9	3.8	3.8	47	66	20.8	16.9	14.24242	17.74194	587	784	345	425	0.15385	0.11111		5.5	Class A
514	59	F	NA	0	0	1	0	0	1	0	0	0	0	0	0	0	0	0	0	0	0	122	111	60	63	6.77	6.65	5.48969	5.59815	8.8	11.3	4.1	4.1	59	76	435	226	17.61194	28.46442	348	144	507	564	2.40625	3.19		5.4	Class A
515	53	M	22.18842	1	0	0	0	0	1	0	0	1	0	0	0	0	0	0	0	0	0	137	126	55	61	4.24	5.32	12.78193	9.91876	8.1	10	2.8	2.9	81	53	103	10.2	31.88976	16.5625	425	528	2320	5970	9.41649	8.84392		6.4	Class A
516	59	F	28.35306	1	0	0	0	1	0	0	1	1	1	1	0	0	1	1	0	0	0	140	146	93	73	1.24	1.32	35.15672	32.83249	10.2	11.3	3.8	3.8	NA	54	NA	77.4	NA	20.30075	NA	4470	NA	125	0.001	0.001		8	Class A
517	76	M	30.34305	1	0	0	0	0	0	1	1	1	0	0	0	0	1	1	1	1	1	147	156	86	92	4.28	4.45	11.40798	10.93207	10.1	11.9	3.1	3.4	74	98	44.7	69.8	28.24427	37.40458	1340	1250	2750	3730	3.31885	4.19005		5.7	Class C
518	80	M	21.96745	0	1	0	0	0	1	0	1	0	1	0	0	0	0	0	0	0	0	176	112	70	58	2.81	3.71	17.74932	13.09699	10.3	12	3.1	3.4	67	109	70.3	68.7	26.37795	38.24561	3010	932	780	599	1.96	1.53	NA		Class A
519	49	F	22.41586	0	0	0	1	1	0	0	0	0	0	0	0	0	0	0	0	0	0	128	NA	84	NA	3.83	4.04	10.79793	10.18541	10.2	10.5	3.8	3.8	58	NA	89.1	NA	22.48062	NA	266	NA	205	NA	2.64706	1.86667		5.3	Class A
520	65	F	21.0251	0	0	1	0	1	0	0	0	1	0	0	0	1	1	1	0	0	0	132	111	73	61	1.81	2.87	22.60683	13.65266	9.9	11.8	3.6	3.4	36	89	102	54.8	14.28571	32.36364	1780	5050	406	384	0.55556	0.3125		6.7	Class A
521	45	F	30.51441	1	0	0	0	0	1	0	0	1	0	0	0	0	1	1	0	1	1	134	152	79	85	3.11	3.05	13.89614	14.19548	10.5	11.1	2.7	2.7	37	73	12.3	20.2	15.61181	30.9322	772	455	378	384	7.9096	13.54074	NA		Class A
522	72	F	NA	0	0	1	0	1	0	0	0	0	0	0	0	0	0	0	0	0	0	128	115	77	81	4.09	4.67	8.99842	7.78322	8.8	10.1	NA	3.9	61	52	118	65.2	24.30279	19.62264	853	1120	1590	1180	2.42308	2.94828	NA		Class A
523	75	M	26.6436	0	0	1	0	0	0	0	0	1	0	0	0	0	1	1	0	1	1	120	114	66	60	4.5	4.4	10.84038	11.1102	9.5	10.4	4.3	4.3	60	83	199	112	22.72727	31.32075	1590	1570	2060	1770	0.3	0.3		5.5	Class A
524	91	F	23.20217	0	1	0	0	1	0	0	1	0	0	1	0	0	1	1	0	0	0	160	NA	90	NA	2.62	NA	13.65267	NA	7.3	NA	3.1	NA	60	NA	98.8	NA	22.30483	NA	150	NA	6940	NA	1.2697	NA	NA		Class A
525	73	M	18.49565	0	0	1	0	0	0	1	0	0	0	0	0	0	0	0	0	0	0	132	126	68	74	3.77	2.95	13.259	17.27217	10.7	11.6	3.5	3.5	111	96	229	155	39.08451	42.47788	685	240	1010	603	6.85131	3.0717	NA		Class A
526	58	M	23.93606	1	0	0	0	1	0	0	0	1	0	0	0	0	0	0	0	0	0	126	136	52	61	5.54	NA	9.29604	NA	8.8	NA	4	NA	60	56	93.3	80.2	22.47191	18.79195	805	553	2820	3100	3.28794	NA		5.8	Class B
527	42	F	20.73905	0	0	0	1	1	0	0	0	0	0	0	0	0	1	1	0	0	0	122	121	75	81	1.98	NA	23.22854	NA	10.8	NA	3.9	NA	NA	63	NA	11.5	NA	21.875	NA	87	NA	46	0.51724	NA	NA		Class A
528	52	F	20.70313	0	0	1	0	1	0	0	0	0	0	0	0	0	1	1	0	1	1	122	124	73	79	3	3.22	13.867	12.83391	9.4	12	4.3	4.3	56	121	48.7	43.7	21.62162	40.19934	770	605	174	117	0.65116	0.72917		5.5	Class A
529	60	M	NA	0	0	1	0	0	0	0	0	1	0	0	0	0	1	1	0	0	0	126	170	79	91	4.5	5.9	11.55734	8.5933	7.5	10	3.3	3.4	89	65	235	106	35.03937	23.72263	111	435	422	1330	2.58152	7.46082		5.7	Class B
530	79	M	19.17576	1	0	0	0	0	1	0	0	1	0	0	1	0	1	1	0	0	0	127	138	75	72	2.14	1.93	23.99619	26.86675	10.2	12	3.6	3.7	92	81	126	96.1	44.87805	37.67442	1590	3520	213	130	0.96667	1.15556		7	Class A
531	88	M	27.57229	0	1	0	0	0	0	0	1	0	0	0	0	0	0	0	0	0	0	146	NA	59	NA	1.23	1.19	42.65506	44.22608	10.3	9.6	3.8	3.5	61	45	34.9	35	15.76227	12.67606	10300	20900	1550	1900	NA	0.17391	NA		Class A
532	56	M	17.96265	0	0	1	0	1	0	0	0	0	0	0	0	0	1	1	0	0	0	120	116	65	73	3.09	2.69	17.78514	20.69773	10.2	11.9	4.4	4.1	108	78	393	266	33.33333	24.14861	125	97	306	85	0.2261	0.13986		5.5	Class A
533	55	M	22.63602	0	0	0	1	0	0	1	0	1	0	0	0	0	1	1	1	0	0	103	148	63	92	4.71	4.34	11.27283	12.26473	8.3	10.1	3.8	3.7	61	54	137	76.1	22.42647	18.30508	4290	7470	263	483	1.82375	2.72244		5.2	Class A
534	78	M	24.9716	1	0	0	0	0	1	0	1	1	1	1	0	0	1	1	0	0	0	140	144	69	74	1.88	1.61	27.85107	32.99917	10.5	10.8	NA	4.2	66	111	191	231	27.16049	40.65934	2450	9190	161	230	NA	3.92289	NA		Class A
535	80	F	23.91566	0	0	1	0	1	0	0	1	1	0	0	0	0	1	1	0	1	1	152	148	63	65	2.67	2.67	13.92054	13.871	9.6	10.3	4.4	4.3	71	75	316	251	16.82464	18.29268	323	910	1840	2420	0.69583	0.54438		6.6	Class B
536	80	F	17.15866	0	0	0	1	1	0	0	1	0	0	0	0	0	1	1	0	1	1	151	136	68	77	1.18	1.16	34.01108	34.65312	8.8	11.4	4.2	4.6	62	74	121	103	20.80537	23.05296	63	189	282	335	0.19054	1.68689	NA		Class A
537	50	F	22.44109	0	0	1	0	1	0	0	0	1	1	0	0	1	1	1	0	1	1	NA	142	NA	81	1.63	1.75	27.33434	25.29054	10.3	13.4	4.3	4.6	66	78	120	205	29.72973	35.45455	312	517	731	175	0.11538	0.002632		5.6	Class A
538	55	M	23.18339	1	0	0	0	0	1	0	0	1	0	0	0	0	1	1	0	0	0	121	136	61	66	1.88	1.91	30.78853	30.1038	10.6	11.6	3.3	3.4	65	62	101	63	23.4657	22.46377	385	126	251	124	4.68392	4.77601		6.1	Class A
539	54	M	27.10459	0	0	0	1	0	0	1	0	1	0	0	1	1	1	1	0	0	1	124	127	74	78	2.2	1.86	25.92431	31.15089	10.9	12	3.8	3.5	73	118	57.7	123	28.74016	54.37788	951	302	233	168	4.77093	2.06506		7.5	Class A
540	83	F	18.88314	0	1	0	0	0	0	1	0	0	1	0	1	0	1	0	1	0	0	114	132	55	69	3.82	NA	9.30879	NA	8.1	NA	3.3	NA	54	54	183	130	31.21387	30.50847	4860	1450	55100	37300	NA	NA	NA		Class A
541	61	F	20.67803	0	0	1	0	1	0	0	1	0	0	0	0	0	1	1	0	0	0	153	NA	99	NA	1.89	1.9	21.95873	21.83233	10.4	10.9	4.3	4	102	106	76.7	61.6	34.45946	34.64052	587	2170	121	121	0.07895	0.1	NA		Class A
542	55	F	19.26363	0	0	0	1	1	0	0	1	0	0	0	0	0	1	1	0	0	0	150	156	94	106	3.06	3.3	13.35312	12.231	9.9	11.2	3.9	4	54	66	65	69.7	20.53232	25.28736	70	50	786	1300	0.71014	0.64407	NA		Class A
543	80	M	25.34435	0	0	0	1	0	1	0	0	0	1	1	0	0	1	0	1	0	0	124	141	65	79	2.87	2.86	17.40572	17.47232	9	10.6	3.2	3.2	72	61	124	24.4	27.90698	19.0625	851	2200	7150	11200	0.57471	1.53465		6	Class A
544	76	F	22.50534	0	0	1	0	1	0	0	0	0	0	0	0	0	0	0	0	0	0	139	110	81	60	1.55	1.73	25.61119	22.71068	10.2	11.7	3.6	3.9	106	102	139	63.7	43.44262	38.78327	1610	1550	139	117	1.3	0.19658	NA		Class A
545	75	M	25.87371	0	0	1	0	0	1	0	0	1	0	1	0	0	0	0	0	0	0	133	141	70	82	1.54	1.51	35.03575	35.66214	11.3	12.2	3.4	3.4	41	113	22.2	25.6	13.31169	37.91946	5080	2230	2470	2330	0.76271	3.42917		6.6	Class A
546	89	F	17.91111	0	1	0	0	1	0	0	0	1	1	0	1	0	1	1	0	0	0	108	98	44	45	4.22	2.95	8.18238	12.06683	9.5	11.2	4.2	3.6	79	44	155	31.8	29.25926	17.46032	176	1380	1330	1350	0.45025	0.67368		6.3	Class A
547	73	M	22.01814	0	1	0	0	0	1	0	0	1	0	0	1	0	1	0	1	1	1	128	140	56	72	3.58	3.22	14.03072	15.75556	10.2	10.9	4.3	4.5	104	96	313	262	35.13514	31.27036	126	174	767	676	1.14458	1.02128		6.3	Class A
548	69	F	NA	1	0	0	0	0	1	0	0	1	1	0	0	1	1	1	0	1	1	NA	120	NA	56	0.76	0.81	57.42307	53.55671	9	8.5	3.9	4	25	34	7.1	6.6	5.263158	7.098121	2900	3470	39	37	0.26471	0.15179		7.5	Class A
549	83	F	24.35507	0	1	0	0	1	0	0	1	0	1	0	0	1	0	0	0	0	0	151	146	86	79	2.65	2.63	13.888	14.00358	9.6	10	3.7	3.3	66	72	98.8	71.7	29.86425	35.29412	2390	1870	447	583	1.21429	NA		5.4	Class A
550	81	M	24.2422	0	1	0	0	0	1	0	1	0	0	0	1	1	1	1	0	1	1	163	137	68	61	2.28	2.28	22.30929	22.23087	9.1	11.2	3.6	3.7	106	109	424	297	54.08163	53.17073	1470	952	502	436	0.92275	1.02151		5.3	Class A
551	83	M	16.00366	0	0	1	0	0	1	0	0	0	0	0	0	0	0	0	0	0	0	124	134	76	74	2.46	1.83	20.38653	28.17765	8.4	10	NA	NA	31	78	157	76.7	12.5	30.58824	3610	284	876	3520	NA	NA		5.8	Class A
552	36	M	42.56055	0	0	0	1	1	0	0	0	0	0	0	0	0	1	1	0	1	1	112	116	63	70	4.58	6.08	13.12672	9.62836	9.3	9.8	3.9	3.9	29	38	39.8	33.2	9.090909	12.45902	27600	3260	866	380	2.53846	2.36364		5.6	Class B
553	80	M	19.82855	0	1	0	0	1	0	0	0	0	0	0	0	0	1	1	0	1	1	120	161	60	81	3.41	3.71	14.4139	13.09699	8.9	11.4	3.5	3.9	96	103	116	129	42.29075	41.2	95	91	5550	7530	1.6588	2.573	NA		Class A
554	86	M	23.60946	1	0	0	0	0	0	0	1	1	0	0	0	0	0	0	0	0	0	160	148	68	62	1.78	2.03	28.75022	24.90004	9.3	10.8	3.7	3.6	85	NA	76.9	NA	39.35185	NA	242	NA	794	NA	0.51	NA		6.2	Class A
555	79	M	22.6784	0	0	0	1	0	1	0	1	0	1	1	0	1	1	1	0	0	0	160	118	79	59	1.43	1.41	37.43218	38.01343	9	10.3	2.9	3.4	48	74	691	60.9	22.85714	28.13688	7600	289	5320	641	1.88777	1.84672	NA		Class C
556	85	F	18.49317	0	0	1	0	1	0	0	1	0	0	0	0	0	1	0	1	0	1	146	144	75	88	1.14	1.18	34.70955	33.42443	10.5	11.2	3.8	3.8	45	50	53.3	30.8	14.51613	14.57726	2630	676	1320	1350	0.12292	0.3556	NA		Class A
557	69	F	24.84079	0	1	0	0	1	0	0	1	0	1	0	0	0	1	0	1	1	1	142	134	80	80	3.85	3.8	9.73201	9.8315	10.2	12.2	4.2	4.4	73	95	246	203	33.03167	40.08439	934	566	233	363	6.1683	2.5095		6.3	Class A
558	56	M	32.52283	1	0	0	0	0	0	1	0	1	0	0	0	0	1	0	1	0	0	134	110	82	72	4.49	5.48	11.81719	9.50267	8.4	9.9	3.44	3.36	155	175	355	598	65.67797	90.20619	1840	483	3200	1420	7.12245	6.17032		4.6	Class B
559	49	F	34.29688	0	1	0	0	1	0	0	1	0	0	0	0	0	1	1	0	0	0	165	177	98	85	2	1.81	21.98027	24.37482	10	11.3	3.9	4	71	59	138	51.6	25.08834	18.4375	8120	3220	170	85	1.03183	0.56568	NA		Class A
560	45	F	24.46038	1	0	0	0	1	0	0	0	1	0	0	0	0	0	0	0	0	1	115	158	66	85	2.31	2.18	19.23901	20.3687	9.8	10.6	3.2	2.7	58	41	37.1	24.7	18.70968	14.80144	151	143	45	191	2.99913	10.68423		9	Class B
561	79	F	22.5567	0	0	0	1	1	0	0	0	0	0	0	0	0	1	1	0	0	0	112	122	53	67	1.19	1.16	33.82041	34.77844	10.1	11.5	4.2	3.8	80	71	201	93.5	28.88807	26.69173	277	205	103	125	1.90769	2.17308	NA		Class A
562	65	M	26.40239	1	0	0	0	0	1	0	1	1	0	0	1	0	1	1	0	0	0	142	131	70	72	1.65	2.3	33.85074	23.43486	10.1	12.3	3.9	4.3	95	93	149	96.9	25.60647	22.68293	2170	820	261	152	1.17333	1.75		6.5	Class A
563	72	M	21.88708	0	0	1	0	1	0	0	1	0	0	0	1	0	1	1	0	1	1	168	161	76	91	4.09	NA	12.17649	NA	9.1	NA	3.81	NA	50	95	435	401	25.64103	49.2228	3320	2470	922	431	1.81481	NA		6	Class B
564	56	M	18.00537	1	0	0	0	0	0	1	0	1	0	0	0	0	0	0	0	0	0	137	147	81	89	1.2	1.3	50.055	45.62592	8.3	9.8	2.8	3	76	179	116	73.3	23.45679	47.3545	501	162	770	1210	4.80488	8.72852		5.7	Class A
565	74	F	18.55148	0	0	1	0	1	0	0	0	0	1	1	0	1	0	0	0	0	0	132	117	54	53	1.6	1.92	24.83112	20.34099	8.5	7.3	3.3	3.4	56	45	60.3	31.8	17.33746	13.39286	2750	6780	4540	3850	NA	0.48571		5	Class C
566	73	M	21.97134	1	0	0	0	1	0	0	0	1	0	0	0	0	1	1	0	0	0	120	123	68	71	1.33	1.43	41.45094	38.14122	9.4	11.7	3.8	4.5	69	127	331	326	26.43678	41.77632	17500	252	655	292	0.17955	0.18243		7	Class A
567	72	F	19.17417	0	1	0	0	1	0	0	0	0	0	0	1	0	0	0	0	1	1	139	136	84	81	3.87	4.14	9.55952	8.84452	8.6	11.3	4.1	NA	75	119	110	221	31.38075	48.97119	160	184	200	176	0.64556	0.92509	NA		Class A
568	71	F	18.369	0	1	0	0	1	0	0	0	0	0	0	0	0	1	1	0	0	0	128	130	60	70	1.63	1.61	24.71734	25.05345	9.8	12.7	4.3	4.6	101	132	106	101	38.11321	45.51724	110	111	324	116	0.08182	0.1	NA		Class A
569	64	F	14.99017	0	0	0	1	1	0	0	0	0	0	0	0	0	1	1	1	1	1	115	109	71	63	2.65	2.8	14.96379	14.08905	10	12.9	3.9	3.8	54	71	51	51.3	18.18182	25.72464	50	50	503	334	1.07252	1.20296		5.4	Class A
570	85	M	19.33594	0	1	0	0	0	1	0	0	0	0	0	0	0	0	0	0	1	1	136	124	60	70	2.34	2.23	21.38633	22.54307	10.7	12.7	3.9	3.9	81	100	40	76.5	30.45113	42.19409	265	684	524	497	0.1	NA	NA		Class A
571	73	M	18.9899	0	0	0	1	0	1	0	0	0	0	1	0	1	1	1	0	0	0	123	113	85	64	1.16	1.17	48.14055	47.69059	10	9.9	3.6	3.3	91	63	56.3	77.2	31.48789	21.14094	246	2780	711	722	NA	0.17921		5.5	Class B
572	78	F	NA	0	0	0	1	1	0	0	0	0	0	0	0	0	1	1	0	0	1	120	114	76	75	2.85	2.5	13.05614	15.06845	9.3	12.1	3.6	3.6	100	137	68.4	22.2	39.68254	43.49206	53	124	216	144	1.09565	1.25224		6.2	Class A

573	61	F	22.7651	1	0	0	0	1	0	0	1	1	1	0	0	0	0	0	0	0	0	170	NA	86	NA	3.85	6.02	10.08237	6.15391	7.2	9.6	2.7	3.8	NA	64	NA	150	NA	27.46781	NA	177	NA	14800	5.04918	7.79545	5.9	Class B
574	56	F	22.22759	0	0	0	1	0	0	1	0	0	0	0	0	0	0	0	0	0	0	NA	NA	NA	NA	2.08	2.29	20.26539	18.24132	8.9	11	3.8	NA	82	NA	171	NA	28.3737	NA	147	NA	127	NA	3.12903	2.625	NA	Class A
575	58	M	27.04164	0	1	0	0	0	0	1	0	0	0	0	0	0	1	1	0	0	0	118	119	76	84	3.13	2.65	17.36091	20.82893	11.2	10.9	4.1	3.7	115	69	119	86	43.56061	26.23574	197	142	92	197	0.67586	0.48923	5.4	Class A
576	71	F	15.67149	0	0	0	1	0	0	0	0	1	0	0	0	0	0	0	0	0	0	116	NA	62	NA	1.94	1.93	20.43054	20.54638	11	11.8	3.9	4.2	76	NA	31.3	NA	27.04626	NA	144	NA	399	NA	0.00122	NA	NA	Class A
577	69	F	22.09479	0	0	0	1	1	0	0	1	0	0	0	0	0	1	1	0	0	0	151	124	74	83	2.5	2.01	15.6081	19.81527	9.6	12.1	3.8	3.7	70	95	24.7	13.2	19.94302	24.23469	394	537	142	91	0.58011	0.54054	NA	Class A
578	81	M	21.02122	1	0	0	0	0	1	0	1	1	1	0	0	0	1	1	0	0	0	154	119	66	67	2.97	3	16.70594	16.46518	10.2	10.5	3.4	3.4	45	58	44.6	43.4	20	23.01587	4530	3890	1050	826	1.008	0.35071	7	Class A
579	87	M	23.08571	0	0	1	0	1	0	0	0	0	1	0	0	0	0	0	0	0	0	131	NA	68	NA	1.44	1.41	36.13359	36.97549	11.3	12.8	4	3.9	53	87	40.4	38.3	15.18625	25.81602	1910	7710	426	422	0.46377	0.38356	5.9	Class A
580	80	M	26.48554	0	1	0	0	0	1	0	1	1	0	0	1	1	1	1	0	0	0	159	122	59	63	1.77	2.47	29.53467	20.51183	10.3	9	3	2.1	86	15	421	761	37.06897	14.01869	2120	104000	438	1590	6.41509	1.59041	6.2	Class A
581	85	M	24.81088	0	0	1	0	0	1	0	0	0	0	0	0	0	0	0	0	0	0	128	130	76	81	1.98	1.98	25.67478	25.67478	9.5	10.9	3.9	3.8	61	71	301	215	26.2931	33.97129	3940	5510	462	476	1.61457	2.03553	5.2	Class A
582	80	M	23.49446	0	1	0	0	1	0	0	0	0	1	0	0	0	1	1	0	0	0	132	102	62	58	3.57	5.75	13.70869	8.13839	10.3	9	3.9	3.7	80	64	34.7	32.9	26.22951	18.02817	251	697	6320	8590	1.88937	0.32716	NA	Class A
583	69	M	22.51996	0	0	0	1	1	0	0	0	1	1	0	1	0	1	1	0	0	0	110	110	60	68	2.13	2.47	25.16545	21.40137	10.5	12.1	4.4	4.2	88	78	126	87.1	33.46008	30.23256	84	414	41	46	0.05556	0.05556	6.2	Class A
584	74	F	22.50931	0	0	1	0	1	0	0	0	0	0	0	0	0	1	1	0	0	0	125	132	72	80	3.51	3.85	10.55384	9.53856	8.9	9.1	4.5	4.5	66	53	100	107	22.22222	17.32026	1650	3060	112	176	1.80993	1.65044	5.8	Class A
585	76	M	21.08356	0	1	0	0	0	0	1	0	1	0	0	0	0	1	1	0	0	0	130	119	75	62	6.21	7.2	7.59217	6.43365	8.6	10.3	3.6	3.9	47	63	187	185	20.34632	27.03863	283	389	1080	1050	1.01997	3.1101	6.1	Class B
586	80	M	29.60382	0	1	0	0	1	0	0	1	1	0	0	0	0	0	0	0	0	1	149	125	87	77	4.43	4.63	10.82553	10.315	9.9	11.2	3.4	3.3	55	50	25.2	25	18.33333	16.23377	335	704	473	455	2.48607	2.75467	6	Class B
587	51	M	25.39295	1	0	0	0	0	1	0	0	1	0	0	0	0	1	0	1	0	0	126	117	80	76	4.74	4.92	11.44004	10.98296	7	10.8	4.3	NA	96	73	37.7	37.5	34.04255	25.97865	85	220	166	165	NA	NA	7	Class A
588	66	M	25.81337	1	0	0	0	0	0	1	0	1	0	0	0	0	1	1	0	0	0	111	132	66	64	2.94	2.98	17.91516	17.65225	9.6	10.3	3.2	2.6	73	61	256	199	30.16529	31.93717	774	543	675	2810	3.25027	10.88377	6.5	Class C
589	72	F	25.43412	0	0	1	0	1	0	0	0	0	0	0	0	1	0	0	0	0	0	134	NA	82	NA	1.74	2.03	22.92086	19.28732	9.4	10	4.1	3.9	99	77	125	59.8	36.13139	29.27757	499	2490	391	382	0.78947	0.73569	5.5	Class A
590	65	M	26.95984	0	0	0	1	0	0	1	0	1	0	0	0	0	0	0	0	0	0	139	145	77	80	6.93	7.55	7.04261	6.41242	10.7	11.7	3.9	3.8	71	94	234	234	26.59176	34.94424	346	281	896	928	2.39492	3.43567	5.6	Class B
591	82	F	26.74048	1	0	0	0	0	0	0	0	1	1	0	0	0	0	0	0	0	0	126	NA	70	NA	2.73	3.01	13.49024	12.12355	10.5	11.4	3.9	3.9	50	NA	70.3	NA	20.24291	NA	857	NA	3770	NA	0.002083	0.94828	6.8	Class A
592	71	F	22.63468	0	0	1	0	1	0	0	1	0	0	0	0	0	1	1	1	0	0	149	132	86	82	3.2	3.49	11.81683	10.74692	10.8	11.7	4.3	4.2	86	103	81.7	58.2	28.66667	31.59509	221	225	550	550	1.83246	2.73224	NA	Class A
593	81	M	18.55698	0	0	1	0	1	0	0	1	0	0	0	0	1	1	1	0	0	0	157	NA	84	NA	2.16	2.09	23.66868	24.53728	9.5	11.6	3.7	3.6	54	59	89.2	38.1	21.95122	25.10638	11700	3180	876	673	1.02057	0.92037	5.6	Class A
594	65	M	24.02381	0	0	0	1	1	0	0	1	0	0	0	0	0	1	1	1	1	1	150	165	70	70	5.53	5.03	9.01476	9.99954	8.3	10.3	4.33	3.69	60	32	41.6	16.3	18.18182	9.667674	287	307	948	718	2.80193	3.38095	NA	Class C
595	54	F	31.99624	0	0	0	1	1	0	0	1	0	0	0	0	1	1	1	0	0	1	164	138	101	99	4.41	5.19	8.95254	7.4915	10.4	11.3	4.1	4.1	89	62	81.1	61.1	28.34395	22.14286	508	625	243	229	1.98165	1.36986	5.9	Class B
596	76	F	24	0	0	0	1	1	0	0	1	0	0	0	0	0	0	0	0	0	0	161	196	58	77	2.55	3.02	14.85586	12.34597	10.3	11	3.53	3.67	78	52	124	122	36.27907	23.85321	866	1430	3130	4220	2.60204	2.71565	5.9	Class A
597	50	M	26.59628	1	0	0	0	0	1	0	1	1	0	0	0	0	1	1	0	0	0	152	174	92	101	1.58	1.65	38.27071	36.29123	10.7	11.5	3.6	3.5	82	60	132	140	28.08219	19.60784	467	934	24	32	2.1	2.76563	6.1	Class A
598	75	M	23.80309	1	0	0	0	0	1	0	0	1	0	0	0	1	1	1	0	0	0	138	NA	50	NA	3.17	NA	15.90378	NA	9.7	NA	3.77	NA	61	NA	45.6	NA	21.40351	NA	337	NA	713	NA	1.44444	NA	5.8	Class A
599	81	F	16.01454	0	1	0	0	1	0	0	1	0	0	0	0	0	1	1	0	0	0	147	132	46	46	1.47	1.27	26.64807	31.27157	9.3	10.3	4.2	4.3	57	94	21.2	21.5	12.72321	22.59615	166	50	271	294	0.07797	0.10286	NA	Class A
600	81	M	24.677	0	0	1	0	0	1	0	0	0	0	0	0	0	1	1	0	1	1	99	104	47	53	2.15	1.9	23.78915	27.23392	10.5	10.6	4.1	4	102	32	57.3	127	30.17751	9.69697	398	23200	406	489	0.21113	0.31509	5.4	Class A
601	84	F	18.61699	0	0	0	1	1	0	0	0	0	0	0	0	0	1	1	0	0	1	125	142	66	80	2.97	3	12.2175	12.08391	9.6	8.9	4.3	3.7	75	64	109	92	36.05769	31.68317	764	5040	4140	7340	3.40136	2.5	5.6	Class A
602	85	F	13.33333	1	0	0	0	1	0	0	0	1	1	1	0	0	1	0	1	1	1	112	154	74	89	2.04	1.49	18.36401	25.89627	9.1	9	4.3	4	34	71	28.8	24.9	10.21021	22.75641	1240	371	1500	1320	0.12077	NA	5	Class A
603	62	M	26.98429	0	0	0	1	0	1	0	1	0	0	0	0	0	0	0	0	0	0	135	138	95	100	1.77	1.71	31.77624	32.998	9.4	11.8	3.7	3.8	94	70	219	86.6	33.33333	23.25581	398	499	95	51	0.000476	NA	NA	Class A
604	61	F	22.77697	0	0	0	1	1	0	0	1	0	0	0	1	0	1	1	0	1	1	148	140	64	57	1.7	NA	24.65729	NA	10.2	NA	4	NA	126	61	52.7	103	39.49843	20.7483	1020	1550	494	320	0.5625	NA	6.3	Class A
605	57	F	27.03894	0	0	1	0	1	0	0	1	0	0	0	0	0	0	0	0	0	0	144	139	93	100	4.39	NA	8.90541	NA	9.2	NA	3.8	NA	62	83	27.8	16.2	22.96296	28.32765	90	125	745	413	0.7619	NA	5.8	Class A
606	88	M	27.09545	0	1	0	0	1	0	0	1	0	0	0	1	0	0	0	0	0	1	162	189	77	107	2.97	3.34	16.31322	14.34685	9.1	9.8	3.9	3.6	37	28	87.8	24.8	16.37168	10.60606	10700	4310	9180	10900	1.43453	1.30609	NA	Class A
607	74	M	22.85495	0	0	0	1	0	1	0	0	0	0	0	0	0	1	1	0	0	0	124	166	53	79	2.97	2.56	17.14497	20.17055	10	11.4	4	4.3	53	82	175	202	20.46332	32.8	442	288	794	693	0.60423	2.19403	NA	Class A
608	85	M	22.21368	0	0	0	1	1	0	0	0	1	0	0	0	0	0	0	0	0	0	131	129	52	54	5.26	5.88	8.81658	7.80476	9.7	11.8	4.2	4.2	44	126	236	157	17.95918	51.42857	1570	282	656	350	0.92701	1.70549	5.6	Class B
609	89	M	15.26764	0	0	0	1	0	1	0	0	0	0	0	0	0	0	0	0	0	0	126	143	61	61	3.77	3.77	12.52591	12.52591	9	10.5	3.2	3.6	39	101	81.9	57.5	12.87129	33.11475	9560	1990	2470	2420	2.01176	2.57353	NA	Class A
610	61	M	19.05023	1	0	0	0	0	0	1	0	1	0	0	0	0	1	0	1	0	0	130	120	50	50	3.72	4.35	14.1657	11.88169	10.2	11.4	3.4	3.3	75	99	141	121	29.64427	39.6	1160	1150	2140	1490	7.15782	4.2521	7.4	Class A
611	76	M	22.9035	0	1	0	0	0	1	0	0	1	0	0	0	0	1	0	1	0	0	127	127	87	81	4.07	4.7	12.05347	10.29753	10.3	10.6	4	3.8	65	82	318	344	26.8595	34.0249	238	2370	1130	1250	2.70333	0.87324	5.8	Class B
612	74	M	NA	0	0	1	0	1	0	0	0	1	0	0	0	1	1	1	0	0	0	NA	NA	NA	NA	1.27	1.05	43.42811	53.47504	8.5	10.3	NA	3.9	154	156	590	380	58.11321	58.64662	304	220	249	185	NA	0.03797	NA	Class A
613	66	M	29.41076	0	0	1	0	0	1	0	1	0	0	0	0	0	1	1	0	0	0	178	157	90	78	4.96	5.31	10.10964	9.38295	9.5	11.4	4	3.8	125	57	136	52.2	44.16961	18.93688	1310	1400	307	385	3.12245	3.25069	NA	Class B
614	77	M	26.05603	0	1	0	0	0	1	0	0	0	0	0	0	0	0	0	0	0	0	136	152	66	66	4.2	6.12	11.6023	7.68551	9.1	9.2	4	3.9	NA	86	NA	254	NA	31.97026	NA	166	NA	924	NA	NA	NA	Class B
615	65	F	17.08327	0	0	1	0	1	0	0	0	0	0	0	0	0	0	0	0	1	1	128	134	75	84	1.77	NA	23.16633	NA	9.7	NA	4.5	NA	147	99	289	205	46.66667	31.93548	90	704	523	326	0.26552	NA	NA	Class A
616	55	M	21.53396	0	0	1	0	0	0	1	0	0	0	0	0	0	0	0	0	0	0	128	154	81	99	1.5	1.3	39.41611	45.85828	9.9	12.3	3.9	4.2	138	104	289	236	49.46237	31.61094	132	178	187	140	0.17632	0.1238	NA	Class A
617	75	M	NA	0	0	0	1	1	0	0	1	0	1	0	0	0	1	1	0	0	0	152	146	64	66	2.96	2.81	17.07714	18.07688	10.8	11.3	4.3	4.2	NA	NA	NA	NA	NA	NA	NA	NA	NA	NA	NA	0.000549	NA	Class A
618	49	F	18.93056	0	1	0	0	0	1	0	0	1	0	0	0	0	1	1	0	0	1	152	106	99	77	2.48	2.68	17.3712	15.95807	10	11.8	4.24	4.39	65	63	44.1	62.4	24.25373	23.59551	320	602	233	111	0.62615	1.0717	NA	Class A
619	86	F	27.50333	0	0	0	1	1	0	0	0	0	0	0	0	0	1	0	1	0	0	120	172	50	62	2.01	2.34	18.60153	15.69936	8.1	10.9	2.8	3.7	64	102	448	123	30.91787	32.17666	1210	1570	363	256	1.29412	2.45455	6	Class B
620	78	M	22.49981	0	1	0	0	0	1	0	0	0	0	0	0	0	0	0	0	0	0	137	153	73	79	5.84	6.02	8.05963	7.76791	9.2	9.8	3.9	4.1	61	72	75.1	74.5	21.32867	23.07692	405	760	170	148	0.39286	0.95161	6	Class C
621	68	M	19.20439	1	0	0	0	1	0	0	0	1	0	0	0	0	1	1	0	0	1	127	NA	65	NA	2.46	NA	21.58682	NA	9.4	NA	3.6	NA	60	NA	43.8	NA	24.59016	NA	203	NA	571	NA	NA	NA	5.9	Class A
622	68	M	24.51494	0	0	1	0	1	0	0	0	0	0	0	0	0	1	1	0	0	0	135	132	78	82	3.2	3.53	16.18966	14.4806	9.4	10.3	3.9	4.2	104	97	44.9	48.6	33.87622	31.69935	128	50	1660	1670	0.3505	NA	NA	Class A
623	57	M	33.13863	1	0	0	0	0	1	0	1	1	0	1	0	0	1	1	0	0	0	157	143	82	68	2.75	2.88	20.10183	19.01615	9.9	10.3	3.8	3.6	94	48	147	104	35.33835	19.4332	375	5960	91	223	2.768	NA	7.3	Class A
624	88	F	NA	0	0	0	1	0	0	0	1	1	0	0	0	0	1	1	0	0	0	NA	NA	NA	NA	1.53	1.48	24.90733	25.74571	9.4	10.7	NA	NA	81	87	63.5	44.6	30.79848	30.74205	390	154	495	220	NA	NA	6.2	Class A
625	76	F	21.98753	0	1	0	0	1	0	0	1	0	0	0	0	0	0	0	0	0	0	144	149	73	70	2.7	2.53	13.95535	14.98438	10.3	11.7	4.2	3.7	64	95	77.9	28.6	21.33333	28.44311	615	527	130	145	0.57105	0.36928	5.5	Class A
626	59	F	22.85446	0	1	0	0	1	0	0	0	1	1	0	0	0	1	1	0	0	0	122	105	68	69	1.28	1.51	33.95658	28.34072	10.4	11.2	4.2	4.4	94	109	373	422	35.74144	39.78102	109	985	36	30	0.09486	0.07727	7.4	Class A
627	75	F	21.0772	1	0	0	0	0	0	0	1	1	0	0	0	0	1	1	0	1	1	143	140	79	70	1.74	1.66	22.65389	23.85093	9.5	10.6	3	3.3	77	71	49	51.3	30.31496	26.10294	3380	3280	544	800	0.33682	0.35606	8.2	Class A
628	70	M	34.20583	0	0	1	0	1	0	0	1	0	0	0	0	0	1	1	0	0	0	144	120	82	69	2.5	3.04	21.03353	16.98224	10.7	11.2	3.8	4	67	72	281	217	24.72325	28.01556	7030	3730	174	148	1.80952	0.83133	5.9	Class A
629	48	M	23.15252	1	0	0	0	0	0	1	1	1	0	0	0	0	1	0	1	0	0	186	197	92	107	3.21	4.55	17.83072	12.17366	8.5	10.6	2.8	2.4	NA	NA	NA	NA	NA	NA	NA	NA	NA	NA	10.31649	13.69879	5	Class C
630	74	F	21.70797	0	0	0	1	1	0	0	0	0	0	0	0	0	1	1	0	0	1	111	127	62	70	1.13	1.15	36.46774	35.77447	9.5	11	2.9	3	97	122	21	40.9	34.51957	43.10954	108	143	287	400	2.79584	4.52113	NA	Class A
631	69	F	22.88927	0	0	0	1	1	0	0	0	0	0	0	0	0	1	1	0	0	0	103	108	71	72	1.58	1.68	25.78491	24.11061	10.2	11.7	3.6	4	74	87	72	64.8	27.61194	29.69283	414	472	109	66	0.77439	0.47854	6.1	Class A
632	51	M	19.9055	0	0	1	0	0	0	0	0	0	0	0	0	0	1	1	1	0	0	132	130	64	80	5.69	5.42	9.36777	9.87948	9.9	12.6	3.9	3.7	83	107	226	121	29.53737	41.31274	92	148	407	389	0.86567	1.40389	5.6	Class A
633	73	M	21.9805	0	1	0	0	0	1	0	1	0	0	0	0	0	1	1	0	0	0	151	153	82	80	1.68	1.73	32.10256	31.08892	10	9.7	4	3.7	82	99	8.6	8.3	21.80851	27.5	468	465	138	119	0.32394	NA	NA	Class A
634	84	M	26.19498	0	1	0	0	0	0	1	0	0	0	0	0	0	1	1	0	0	0	121	144	57	65	5.6	5.01	8.26068	9.33063	9.5	9.9	3.7	3.6	86	88	218	142	31.73432	32.83582	185	80	2790	4560	5.7377	5.32203	5.2	Class B
635	75	F	25.98982	0	0	1	0	1	0	0	0	1	0	0	0	0	0	0	0	0	0	126	136	72	77	2	1.83	19.45256	21.43789	10.5	11.6	4.2	4.3	59	45	154	120	22.34848	19.82379	547	1160	416	518	0.47496	0.46326	5.9	Class A
636	62	M	21.07037	0	1	0	0	0	1	0	1	0	0	0	0	0	1	1	0	0	0	159	152	107	88	4.23	3.92	12.25093	13.25367	10.9	10.8	3.6	3.3	83	98	15.5	54.7	28.71972	36.70412	460	548	2900	2780	1.41	3.33333	NA	Class A
637	68	F	NA	0	1	0	0	1	0	0	0	0	0	1	0	0	1	1	0	0	0	99	116	54	60	0.79	0.8	55.27288	54.28952	10.9	11.8	NA	4.3	47	113	38.7	44.3	16.1512	35.4232	363	273	1090	1090	NA	0.10938	NA	Class A
638	81	F	17.12247	0	0	1	0	1	0	0	1	1	1	1	1	0	1	0	1	0	0	142	141	66	65	3.05	2.13	11.99187	17.76085	9.5	10.2	4.2	3.6	59	47	460	301	26.33929	23.03922	870	1690	1940	4180	NA	0.10606	9.5	Class A
639	68	M	24.68769	1	0	0	0	0	1	0	0	1	1	0	0	0	0	0	0	0	0	172	170	82	77	3.67	4.41	13.93564	11.39871	8.7	13.2	2.8	2.7	96	NA	135	NA	37.06564	NA	206	NA	1420	NA	4	2	NA	Class A
640	86	M	21.87736	0	1	0	0	1	0	0	1	0	1	0	0	0	0	0	0	0	1	163	153	79	77	1.51	1.66	34.41913	30.92869	10.2	12.3	3.4	3.5	84	142	18.7	51.1	26.92308	47.49164	1510	915	195	259	0.2322	1.15207	NA	Class A
641	59	M	26.88512	1	0	0	0	0	0	0	1	1	1	0	0	0	0	0	0	0	0	141	144	66	65	3.39	NA	15.83174	NA	10.9	NA	4.4	NA	62	NA	213	NA	20.59801	NA	186	NA	843	NA	0.75	NA	NA	Class A
642	64	M	26.35974	1	0	0	0	0	0	1	0	1	1	0	0	0	1	1	0	0	0	141	151	77	81	4.05	4.46	12.73129	11.4566	9.9	9.9	3.83	3.87	87	96	60.3	60.4	33.72093	35.0365	83	117	881	731	NA	1.81634	5.3	Class B
643	56	M	NA	0	0	1	0	1	0	0	0	0	0	0	0	0	1	1	0	0	0	128	135	78	79	2.87	2.79	19.28186	19.88753	11.1	13.3	3.6	NA	66	55	256	188	24.7191	20	270	1040	771	677	0.27143	0.29851	NA	Class A
644	70	M	22.75375	0	0	0	1	1	0	0	0	0	0	0	0	0	1	1	1	0	0	130	122	70	70	2.28	2.33	23.26365	22.71806	9.9	11.2	2.8	3.3	57	53	125	112	23.45679	20.94862	4660	6570	265	258	5.67089	6.5102	NA	Class B
645	88	F	20.23705	0	0	1	0	1	0	0	0	0	0	0	0	0	0	0	0	0	0	134	NA	87	NA	2.05	NA	18.0851	NA	9.6	NA	3.7	NA	54	NA	105	NA	24	NA	120	NA	936	NA	0.28424	NA	NA	Class A
646	82	F	20.36158	0	0	1	0	1	0	0	1	0	0	0	0	0	0	0	0	0	1	140	152	78	82	4.36	4.4	8.0832	8.00284	7.7	11.2	NA	NA	103	103	45	25	45.57522	43.45992	50	50	1630	2220	12.97561	15.40426	NA	Class A
647	77	M	25.94879	0	0	0	1	0	1	0	0	0	0	0	0	0	1	1	0	0	0	137	120	73	66	2.03	2	25.70267	26.12475	11.3	12.6	3.8	4	58	92	354	263	31.1828	42.7907	1120	708	380	298	1.27329	0.60658	5.8	Class A
648	77	M	17.65684	0	1	0	0	0	1	0	0	0	1	0	1	0	0	0	0	0	0	95	146	52	74	5.13	5.53	9.32203	8.58692	8.8	11.2	3.2	3.4	54	61	202	60	26.08696	28.50467	1360	710	2250	2860	1.98758	3.2243	NA	Class B
649	78	F	20.37103	0	0	0	1	1	0	0	1	0	0	0	0	0	1	1	0	0	1	153	124	87	78	1.71	1.81	22.8306	21.45432	10.9	11.9	4.1	3.8	59	112	35.3	27.7	20.34483	38.75433	575	871	182	211	3.82883	1.59164	6.1	Class A
650	70	M	25.58897	1	0	0	0	0	1	0	0	1	0	0	0	0	1	1	0	0	0	126	128	57	69	3.07	3.11	16.73251	16.49722	8.4	9.6	3.5	3.8	82	56	79.9	58.6	28.3737	20.07168	443	324	1760	939	1.75495	0.89595	6.4	Class A
651	54	F	24.75762	0	0	0	1	1	0	0	1	0	0	0	0	0	1	1	0	0	0	140	143	86	86	4.42	4.51	8.97754	8.7356	11	12.2	4.52	4.22	85	87	44	41.4	31.36531	31.75182	130	210	165	190	0.91379	1.35556	6.1	Class A
652	79	M	28.64124	1	0	0	0	0	1	0	0	1	0	0	0	0	1	1	0	0	1	106	121	63	63	4.5	4.36	10.67993	11.05566	10.6	11	3.6	3.8	103	111	25.8	44.7	39.61538	36.63366	1830	394	5100	5350	1.07028	1.56813	6.7	Class A
653	78	M	16.46091	0	0	0	1	0	1	0	1	1	0	0	1	1	1	1	1	0	0	152	166	63	65	2.2	2.38	23.36535	21.43915	10.5	12.3	2.3	2.9	76	123	146	182	47.20497	60	1310	857	796	736	9.14286	8.71277	5.9	Class A
654	91	M	24.22145	0	0	0	1	1	0	0	0	0	0	0	0	0	0	0	0	0	0	138	132	56	50	1.09	1.06	48.37403	49.87377	10.5	10.8	3.4	3.4	60	70	119	105	24.89627	30.43478	7700	2040	265	215	0.000562	0.000833	5.4	Class A
655	74	M	19.06771	0	0	1	0	1	0	0	0	0	0	0	0	0	1	1	0	0	0	132	118	81	68	2.59	3.14	19.91509	16.07008	10	10.7	4.2	4.4	63	147	188	215	24.23077	54.85075	257	485	845	404	0.6	0.001	NA	Class B
656	54	M	23.35564	1	0	0	0	0	1	0	0	1	0	0	0	0	1	1	0	0	0	136	146	87	98	2.17	2.76	26.45561	20.22847	10	11.1	3	2.8	63	70	215	179	29.03226	35	336	451	334	655	5.26471	7.58333	6.4	Class B
657	57	M	16.986	1	0	0	0	0	0	1	0	1	0	0	0	0	0	0	0	0	0	133	159	79	93	4.35	5.68	12.1719	9.09094	8.9	11.1	3.1	3.2	121	109	117	102	44.32234	40.97744	410	1030	3070	9540	6.7178	11.64499	6.1	Class C
658	62	M	NA	0	1	0	0	0	1	0	1	0	0	0	1	0	1	1	0	0	0	152	158	78	82	7.01	8.74	7.04969	5.53824	9.2	10.9	3.7	3.9	89	84	49.8	35.6	36.62551	30.32491	3910	6840	3980	3890	NA	NA	NA	Class B
659	66	F	18.20348	0	0	1	0	1	0	0	0	0	0	0	0	0	0	0	0	0	0	134	160	76	87	1.36	1.17	30.77128	36.27787	10.5	11	4.2	4.1	85	135	54	40.5	19.58525	32.45192	3910	1110	208	126	0.1746	0.21429	5.2	Class A
660	61	M	18.71095	0	1	0	0	0	1	0	0	0	0	0	0	0	1	1	0	0	0	126	140	89	94	4.55	5.65	11.36443	8.96749	9.8	10.4	3.8	3.8	65	103	50.7	46.9	21.73913	32.2884	4950	434	9690	10300	1.58537	2.97959	5.5	Class B
661	74	M	20.07813	1	0	0	0	0	1	0	0	1	0	0	0	0	1	0	1	0	0	118	112	80	80	1.21	1.29	45.78941	42.69206	7.5	9.8	3.2	3.1	101	88	567	386	44.1048	38.59649	363	5280	452	757	0.84549	0.79505	5.9	Class A
662	73	F	28.14787	0	0	1	0	1	0	0	0	0	0	0	0	0	1	1	0	0	0	107	NA	50	NA	2.18	NA	17.84026	NA	8.7	NA	4.5	NA	79	NA	141	NA	25	NA	483	NA	88	NA	0.11091	NA	6.1	Class A
663	57	F	16.83476	0	0	0	1	1	0	0	0	0	0	0	0	0	1	0	1	0	0	114	114	56	66	2.86	2.51	14.23134	16.41599	8.5	13.1	4.7	4.9	68	100	130	13.1	31.92488	33.33333	50	50	150	88	0.09166	0.65987	NA	Class A
664	78	M	22.07814	0	1	0	0	0	1	0	1	0	0	0	0	0	1	0	1	0	0	159	137	62	71	1.16	1.18	47.23387	46.35874	9.4	9.8	3.6	3.3	115	83	151	169	40.35088	31.92308	1300	14500	97	96	0.51796	0.71846	5.9	Class A
665	82	F	23.40686	0	1	0	0	0	1	0	1	1	0	0	0	0	0	0	0	0	0	148	162	60	77	4.61	5.09	7.60489	6.8002	7.9	8.6	3.2	3	62	43	255	208	33.15508	26.54321	144	4560	11200	30100	5.19512	5.98756	5.6	Class C
666	81	M	22.06035	0	0	1	0	1	0	0	0	0	1	0	0	0	1	1	1	0	0	112	140	60	60	1.53	1.37	34.51549	38.94881	8.5	9.7	3.2	3.4	82	66	169	140	47.67442	32.35294	2160	6680	1870	1520	2.15862	6.66397	5.3	Class A
667	79	M	29.64778	0	1	0	0	0	1	0	0	0	0	0	0	0	1	1	0	0	0	133	128	73	68	2.1	2.59	24.58526	19.47444	10.3	9.8	4.3	4.1	149	97	253	243	40.82192	28.36257	152	3320	243	410	0.09393	0.05315	NA	Class A
668	63	F	24.96456	0	0	1	0	1	0	0	0	1	0	0	0	0	1	1	0	0	0	118	130	50	68	2.69	3.15	14.78722	12.44181	10.2	10.8	4.3	4.3	52	78	144	150	19.54887	30.46875	1990	1560	195	180	1.72841	1.11868	6.5	Class A
669	64	F	NA	0	0	1	0	1	0	0	1	0	1	0	0	0	1	0	1	0	0	170	143	93	81	2.74	3.17	14.42691	12.30024	9.5	10.4	4	4	78	93	55.5	40.3	29.88506	34.96241	860	331	290	208	0.88095	0.8913	5.9	Class A
670	51	M	19.36288	0	0	0	1	1	0	0	0	0	0	0	0	0	1	1	0	1	1	119	142	77	86	5.69	6.68	9.31571	7.81634	9.1	10.4	4.5	4.22	83	119	66	87.7	27.94613	36.84211	305	244	574	1420	0.78958	4.93112	NA	Class B
671	52	F	34.4941	0	0	0	1	0	0	0	1	0	0	0	0	1	1	1	0	0	0	132	132	88	76	3.1	3.37	13.37838	12.21029	10.8	11.9	3.5	3.7	48	29	88.1	35.9	17.77778	10.06944	5010	7070	91	118	3.17308	4.13601	6.2	Class A
672	75	M	19.61738	1	0	0	0	0	1	0	1	1	0	0	0	0	1	1	0	0	0	122	148	80	88	2.58	3.31	19.92265	15.11181	8.7	10.6	3.9	3.7	49	90	218	98.3	17.88321	28.21317	7930	902	52	46	3.6389	3.30333	6.2	Class A
673	65	F	21.64184	0	0	1	0	1	0	0	1	1	0	0	0	0	0	0	0	0	0	142	106	78	68	3.96	NA	9.59978	NA	10.3	NA	4.6	NA	84	56	80.3	135	32.43243	24.13793	1400	4250	246	395	0.50251	NA	5.7	Class A
674	69	F	19.50351	0	1	0	0	1	0	0	1	0	0	0	0	0	1	0	1	0	0	164	152	95	84	1.88	2.09	21.31905	18.90876	9.9	11.8	4.2	4.6	60	75	43.4	25.7	18.63354	18.29268	95	81	856	424	0.69597	0.38194	5.7	Class A
675	58	F	18.02037	0	0	1	0	1	0	0	0	0	0	0	0	0	0	0	0	0	0	119	105	73	63	2.17	2.11	19.15382	19.75047	10	11.6	4.4	4.7	64	NA	62.6	NA	24.52107	NA	213	NA	277	NA	13	0.15556	6.1	Class A
676	67	M	26.05536	1	0	0	0	0	1	0	1	1	0	0	0	0	1	1	0	0	0	167	NA	75	NA	2.46	NA	21.6788	NA	10.9	NA	3.3	NA	73	NA	70.6	NA	24.49664	NA	347	NA	589	NA	2.06242	NA	6.7	Class A
677	62	F	19.27186	0	0	1	0	1	0	0	1	0	0	0	0	0	1	1	0	0	0	145	148	76	82	4.3	4.8	8.89232	7.88409	9.6	12.5	4.3	3.9	66	62	108	43.7	21.63934	21.08844	2140	1710	560	509	1.63934	1.75439	5.1	Class B
678	57	M	20.23739	1	0	0	0	0	0	1	0	1	0	0	0	0	1	1	0	1	1	122	130	70	77	2.29	2.46	24.55871	22.59513	10	12.3	4.2	4.1	82	44	91.6	47.5	24.84848	14.96599	508	325	662	637	2.67735	3.42222	10.6	Class A
679	83	M	NA	0	0	1	0	0	0	1	0	0	0	0	0	0	0	0	0	0	0	130	NA	60	NA	2.3	NA	21.943	NA	9.3	NA	3	NA	41	62	258	179	19.33962	26.38298	6760	484	981	456	0.16216	NA	5.7	Class A
680	87	F	20.70465	1	0	0	0	0	0	1	1	1	0	0	0	0	1	1	0	0	0	155	153	63	70	2.38	2.09	15.36047	17.70678	11.1	10.6	3.7	3.3	60	72	40.5	32	21.20141	24	626	1640	819	1790	2.60465	4.48485	5.5	Class A
681	41	F	20.95661	0	0	0	1	1	0	0	0	0	0	0	0	0	1	0	1	0	1	100	101	45	59	3.83	4.85	11.36469	8.7776	9.2	12	4	4.1	98	36	30.6	22.4	38.73518	12.04013	372	5770	163	102	0.45007	0.54208	NA	Class B
682	72	F	18.62656	0	0	1	0	1	0	0	1	0	0	0	0	0	0	0	0	0	1	143	132	76	78	1.6	1.48	25.02449	27.25249	10.2	9.5	3.6	3.2	39	45	9.5	21.1	12.03704	15.41096	2830	5020	208	303	1.25714	NA	NA	Class A
683	61	F	26.27195	0	1	0	0	0	0	1	0	0	0	0	0	0	1	1	0	0	1	129	123	81	80	3.66	4.95	10.65634	7.6231	10.5	11.1	4.3	4.3	99	81	96.2	58.8	35.35714	27.93103	334	413	479	431	1.99485	2.30631	5.3	Class A
684	76	M	25.5509	0	0	0	1	0	1	0	0	0	0	0	1	0	1	1	0	0	0	128	125	78	79	2.63	2.74	19.43465	18.58271	10.6	11.5	3.8	3.9	99	86	95.9	92.4	30.84112	25.51929	140	160	261	302	2.20073	2.63577	5.7	Class A
685	79	M	26.16904	0	0	0	1	0	1	0	0	0	0	0	0	0	1	1	0	0	1	110	132	70	76	3.5	4.01	14.05958	12.07188	8.4	8.7	3.7	NA	69	119	21.7	40	23.63014	46.85039	381	414	7510	7540	1.52655	0.83405	NA	Class C
686	77	M	20.85587	0	1	0	0	1	0	0	1	0	0	0	0	0	1	1	0	0	0	132	137	76	85	2.2	2.8	23.53794	18.07957	10.4	11.9	3.4	4	75	138	94.3	111	31.64557	53.48837	293	557	98	73	5.44323	NA	6.2	Class A
687	76	M	28.73469	0	0	0	1	0	1	0	1	1	0	0	1	0	1	1	0	0	0	141	141	87	71	5.62	5.6	8.46831	8.46957	9.6	10.7	3.9	3.9	64	80	306	288	31.06796	37.2093	1090	518	7840	5650	1.46789	1.37838	NA	Class B
688	74	F	27.06887	0	0	0	1	0	1	0	1	0	1	0	1	0	1	1	0	0	0	160	154	78	73	4.13	4.01	8.83339	9.12298	10.6	11.9	3.9	4.3	78	76	131	93.9	24.5283	20.76503	1350	753	1180	961	0.92308	1.80769	6	Class A
689	89	M	23.76691	0	1	0	0	0	1	0	0	1	0	0	0	0	0	0	0	0	0	123	142	45	49	4.03	NA	11.64456	NA	8.7	NA	3.5	NA	18	NA	5	NA	5.538462	NA	7520	NA	1420	NA	0.06719	NA	6.9	Class A
690	77	M	23.05569	0	0	1	0	0	1	0	1	0	0	0	0	0	0	0	0	0	0	158	151	85	87	2.45	2.33	20.92335	22.02334	10.8	11	4.1	3.9	90	95	90.7	78.1	24.93075	27.14286	121	1590	802	884	1.22636	NA	NA	Class A
691	66	F	NA	0	1	0	0	0	0	1	1	1	0	0	0	0	1	1	0	0	0	172	158	76	74	2.44	2.21	16.23426	18.01348	9.6	10.1	4	4.1	58	58	40.2	74.3	20.27972	20.71429	146	2640	1240	398	0.05697	1.05376	6.3	Class A
692	69	M	18.43288	1	0	0	0	0	1	0	0	1	0	0	0	0	1	1	0	0	0	130	145	60	76	3.67	3.35	13.87738	15.33391	10.7	10.9	4.2	4.2	67	74	537	538	27.12551	29.36508	101	67	986	790	1.26316	0.7037	6.1	Class A
693	77	M	21.60719	0	0	0	1	1	0	0	0	0	0	0	0	0	0	0	0	0	0	NA	NA	NA	NA	4.14	3.63	11.78638	13.55916	10.9	12	3.6	4.1	66	NA	354	NA	27.73109	NA	515	NA	880	NA	2.52736	NA	NA	Class A
694	81	F	24.24786	0	1	0	0	1	0	0	0	0	0	0	1	1	0	0	0	0	1	135	150	65	85	1.64	1.4	23.64132	28.1091	10.8	12.2	NA	3.9	83	91	45.2	38.4	27.12418	28.88889	697	964	168	270	NA	0.12	5.9	Class A
695	69	F	20.77562	0	0	0	1	1	0	0	1	1	0	0	0	0	1	1	0	0	0	146	146	92	94	1.17	1.44	35.81798	28.5396	7.8	10.6	3.1	2.7	132	95	407	178	49.62406	39.09465	100	50	369	376	8.18349	14.0137	5.7	Class A
696	76	M	20.04138	1	0	0	0	1	0	0	1	1	1	0	1	1	1	1	0	0	1	143	157	78	74	2.39	2.32	21.57948	22.29279	9.5	10.1	3.4	3.4	78	79	9.8	23.7	24.60568	26.87075	99	98	793	1040	1.75207	2.62201	5.7	Class A
697	74	M	21.47109	0	0	1	0	1	0	0	0	0	0	0	0	0	1	1	0	0	0	138	144	50	44	2.31	2.31	22.57048	22.57048	9.1	11	3.6	3.5	76	72	194	165	32.61803	30.6383	1300	361	955	2090	2.55274	4.29098	NA	Class A
698	68	M	22.57805	0	0	0	1	0	1	0	0	0	0	0	0	0	1	1	0	1	1	136	128	76	76	5.49	NA	8.96973	NA	10.8	NA	4.3	NA	93	146	209	303	34.44444	56.58915	808	790	466	200	1.49921	NA	NA	Class B
699	83	F	24.60738	0	1	0	0	1	0	0	1	0	0	0	0	1	1	1	0	0	0	167	112	76	55	2.81	3.12	13.02525	11.61624	10	11.9	3.6	3.7	99	110	107	89.5	37.35849	45.45455	73	83	853	816	2.97143	2.62136	5.5	Class A
700	73	M	24.74745	1	0	0	0	0	1	0	1	1	0	0	0	0	0	0	0	0	0	168	132	89	75	5.54	5.6	8.70219	8.56672	9.5	10.5	3.6	3.4	100	59	111	21.2	39.84064	20.92199	2440	1600	738	738	1.55	3.60405	6.6	Class C
701	66	M	22.76329	0	1	0	0	0	1	0	0	0	1	1	0	0	1	1	0	0	0	122	126	80	73	1.93	1.94	28.3918	28.11015	10.6	11.3	3.6	3.9	43	75	10.8	12	11.1399	19.18159	601	466	1020	981	0.22556	1.0241	6.1	Class A
702	77	F	26.56434	1	0	0	0	0	1	0	1	1	1	0	0	0	0	0	0	0	0	149	142	75	69	1.73	1.44	22.62564	27.65507	9.9	11.7	4.4	4.3	57	114	122	122	17.98107	34.7561	953	159	1150	716	0.6699	1.56364	6.9	Class A
703	70	M	20.82031	0	0	1	0	0	1	0	0	0	0	1	1	0	1	1	0	0	0	126	126	55	73	2.46	2.4	21.40797	21.99417	11.3	11.8	3.9	3.8	47	57	166	291	23.73737	29.23077	851	418	662	640	NA	0.41145	NA	Class A
704	45	M	24.99901	0	0	0	1	0	0	1	0	1	0	0	0	1	1	1	0	0	0	125	127	63	64	4.02	4.8	14.20059	11.69639	12.2	12.5	4.6	4.6	57	86	79.9	26.9	20.80292	26.70807	332	53	435	79	8.52632	2.34621	7.7	Class A
705	67	F	24.04176	0	0	0	1	0	0	1	0	0	0	0	0	0	1	1	0	0	0	136	NA	70	NA	0.98	NA	43.84917	NA	10	NA	3.5	NA	31	52	34.6	19.4	11.07143	17.10526	6660	5250	112	63	NA	NA	6.8	Class A
706	88	F	24.90156	0	1	0	0	1	0	0	0	0	0	0	0	0	1	1	0	0	0	111	122	69	77	1.41	1.57	27.23541	24.21394	10.1	10.2	3.8	3.8	40	24	34.8	18.4	11.52738	6.233766	283	204	520	583	0.41379	0.17797	NA	Class A
707	83	F	24.55753	0	0	1	0	1	0	0	0	0	0	0	0	0	1	1	0	0	1	119	127	53	67	1.5	1.55	25.88373	24.97169	9.6	12.3	4.1	4.4	82	86	81.9	47.7	29.39068	27.5641	100	77	141	136	0.17683	0.32004	5.9	Class A
708	48	M	NA	0	1	0	0	1	0	0	1	0	0	0	0	0	0	0	0	0	0	134	127	91	99	2.47	2.08	23.75062	28.66317	11.1	12.1	4.1	4.2	94	78	28.6	17	24.2268	17.33333	556	507	152	214	0.15799	0.39192	NA	Class A
709	53	M	27.59938	0	0	0	1	0	0	1	1	0	0	0	0	0	1	0	1	0	0	137	131	96	97	3.55	3.18	15.52332	17.41604	10	11.4	4.2	4	129	165	147	106	34.49198	53.57143	50	332	40	93	1.24719	1.27848	NA	Class B
710	44	M	21.80089	0	0	1	0	1	0	0	1	0	0	0	0	0	1	1	0	1	1	140	138	78	63	1.37	1.2	46.40433	53.64219	10.2	10.5	2.6	2.6	56	67	152	122	27.05314	32.21154	155	313	284	265	5.2521	5.62771	NA	Class A
711	50	F	22.72633	0	0	1	0	1	0	0	0	1	1	1	1	1	1	1	0	1	1	114	131	58	76	3.32	3.27	12.55211	12.76223	9.3	9.9	3.8	3.7	64	70	59.8	67	26.12245	30.837	1920	2210	1130	994	0.30718	0.29754	5.2	Class A
712	68	F	18.29145	0	0	1	0	1	0	0	0	0	0	0	0	0	1	0	1	0	0	120	130	74	80	1.17	1.24	35.96837	33.75303	10.5	11	4.1	3.9	77	109	96.5	92.4	30.92369	38.5159	145	83	244	227	2.01977	1.53471	NA	Class A
713	58	M	NA	1	0	0	0	0	0	1	0	1	0	0	0	0	1	1	0	0	0	NA	NA	NA	NA	4.04	4.03	13.0673	13.10278	10.2	11.7	4	4.4	118	86	253	212	39.20266	30.82437	410	1230	724	800	2.02825	1.68	6.8	Class A
714	64	M	17.78917	1	0	0	0	0	1	0	0	1	0	0	0	0	1	1	0	0	0	130	122	67	67	2.42	2.36	22.36321	22.98594	11	11.8	4.2	3.8	71	65	46.3	41.2	24.82517	20.70064	572	269	179	143	0.97191	3.51786	5.5	Class C
715	59	M	19.95936	0	0	0	1	0	1	0	1	0	0	0	0	0	0	0	0	0	0	142	133	88	81	5.57	6.49	9.1518	7.74242	10.1	10.3	4.1	4.1	101	142	31.7	31.5	29.88166	39.11846	187	66	369	311	4.01575	3.72458	5.7	Class C
716	64	M	17.82457	1	0	0	0	0	1	0	0	1	0	0	0	0	1	1	0	1	1	96	143	60	86	2.89	2.5	18.41645	21.5815	9.9	11.1	2	2.1	38	NA	203	NA	20.65217	NA	18400	NA	1160	NA	4.75893	10.07692	6.2	Class B
717	72	M	22.71713	1	0	0	0	0	0	1	0	1	1	0	1	0	1	1	0	0	0	118	133	50	64	4.09	3.62	12.17649	13.91618	10.3	11.9	3.9	3.3	63	80	80.2	52.6	25.92593	28.98551	2160	5900	788	1810	1.37358	4.23398	5.5	Class B
718	66	M	25.33913	0	0	1	0	1	0	0	0	0	1	0	0	0	1	1	0	0	0	114	150	70	84	4.7	5.51	10.72303	9.011	10.5	11.8	4.5	3.9	92	121	98.8	63.2	35.1145	43.84058	105	76	1060	1250	1.9125	4.37097	NA	Class A
719	78	M	25.78125	0	0	0	1	0	1	0	0	1	0	0	1	0	1	1	0	0	0	135	128	59	68	2.03	2.35	25.60766	21.81837	9.2	12.7	3.4	3.1	70	116	273	176	33.65385	51.10132	656	485	565	190	4.47005	6.29902	7.4	Class A
720	86	F	23.22199	0	1	0	0	0	1	0	0	1	0	1	0	0	1	1	0	0	0	145	108	74	56	1.29	1.18	30.2176	33.20208	10.7	11	4.1	3.2	103	68	69.2	147	40.71146	31.05023	334	24800	78	160	0.06144	11.56	5.7	Class A
721	74	M	21.40102	1	0	0	0	0	1	0	1	1	1	1	0	1	1	0	0	0	0	150	162	80	85	2.13	NA	24.66522	NA	8.9	NA	2.8	NA	NA	NA	NA	NA	NA	NA	NA	NA	NA	NA	NA	NA	6.6	Class A
722	68	M	16.99378	1	0	0	0	0	1	0	1	1	0	0	0	0	0	0	0	0	0	149	NA	73	NA	3.09	NA	16.8212	NA	8.4	NA	2.6	NA	64	NA	78	NA	27.46781	NA	434	NA	903	NA	NA	NA	NA	Class A
723	69	F	23.01698	0	0	0	1	1	0	0	0	0	0	0	1	0	1	1	0	0	0	128	136	68	78	3.15	3.06	12.12118	12.51173	9.5	11	3.7	3.9	65	75	35.6	7.1	24.62121	21.73913	410	268	329	315	2.14592	3.42183	NA	Class A
724	40	M	NA	0	0	1	0	0	1	0	1	0	0	0	0	0	1	1	0	1	1	192	NA	122	NA	3.44	NA	17.41869	NA	9.3	NA	3.6	NA	54	95	349	61.7	22.5	30.64516	509	439	1130	120	0.22917	NA	6.1	Class A
725	80	M	25.33748	0	0	1	0	0	1	0	1	0	1	0	1	0	0	0	0	0	0	156	166	86	88	2.69	2.28	18.68383	22.38897	10.7	10	4.3	4.3	34	41	20	26.1	8.80829	9.808612	451	220	3170	10100	0.26525	0.61333	6.1	Class A
726	55	M	29.32153	1	0	0	0	0	1	0	1	1	0	1	0	0	1	1	0	0	0	140	162	80	88	4.81	4.96	11.01668	10.59778	10.2	11.4	3.8	4	68	79	116	97.1	26.05364	30.38462	250	286	2320	2320	3.82222	3.36364	6.1	Class B
727	76	M	15.81987	0	0	0	1	0	1	0	0	0	0	0	0	0	1	1	1	0	0	97	132	57	79	2.53	2.39	20.27656	21.57948	9.9	10.9	4.1	3.8	78	82	115	60.4	32.5	31.66023	50	50	434	386	NA	1.24675	NA	Class B
728	69	M	23.74754	0	1	0	0	0	0	1	1	0	0	0	0	0	0	0	0	1	1	149	NA	72	NA	1.22	1.41	46.2993	39.51904	9.5	12	4.3	4.6	120	152	264	296	48	47.35202	549	667	191	67	0.14072	0.17831	5.5	Class A
729	49	M	23.25502	0	0	0	1	0	0	1	1	0	1	0	0	0	1	1	0	0	0	152	134	104	94	3.78	5.25	14.73741	10.28829	10.5	11.3	3.2	3.7	NA	NA	NA	NA	NA	NA	NA	NA	NA	NA	2.53731	3.64286	NA	Class A
730	49	M	30.26283	0	0	0	1	0	1	0	1	0	0	0	0	0	1	1	0	0	0	152	182	81	98	6.27	8.32	8.52128	6.25317	10.1	8.6	4.3	4.2	81	112	106	200	28.02768	40.43321	322	645	161	768	1.16923	3.03175	NA	Class C
731	84	F	25.15315	1	0	0	0	0	1	0	1	0	0	0	0	0	0	0	0	0	0	118	111	69	66	1.64	1.73	23.39585	22.06762	10.5	11.6	4.4	4.1	NA	76	NA	71.5	NA	22.09302	NA	2520	NA	467	0.4902	0.71942	NA	Class A
732	72	M	25.91732	0	0	0	1	0	1	0	1	1	0	0	1	0	1	1	0	0	0	146	146	56	62	2.56	NA	20.32978	NA	10.5	NA	3.3	NA	90	99	87.1	106	39.13043	43.04348	155	273	557	820	13.61702	NA	5.5	Class B
733	80	M	25.73203	0	0	0	1	0	1	0	1	0	0	0	0	0	0	0	0	0	0	148	145	80	81	2.95	2.6	16.89	19.3235	9.8	10.7	4.2	4.3	50	67	198	121	19.68504	26.48221	1240	349	1190	887	NA	0.33	NA	Class A
734	50	M	23.5123	0	0	1	0	1	0	0	0	0	0	0	0	0	1	1	0	0	0	102	116	55	76	1.91	1.75	31.09903	34.02871	9	12.8	4	4.2	92	104	111	76.6	29.11392	30.40936	97	406	48	51	0.002125	0.10852	5.8	Class A
735	77	F	18.33576	1	0	0	0	0	1	0	0	1	1	0	0	0	1	1	0	0	0	148	110	78	52	1.01	1.01	40.76578	40.76578	11	11.5	4.7	4.1	101	116	45.2	43.3	23.32268	40.9894	124	148	139	189	1.02767	1.19469	6.7	Class A
736	85	M	18.46272	0	0	1	0	0	1	0	0	0	0	0	0	0	0	0	0	0	1	117	131	63	68	1.1	1.13	48.83992	47.26426	9.5	10.2	3.1	3.2	27	25	36.7	47.8	9.71223	9.615385	16200	19600	545	713	0.06983	0.06564	NA	Class A
737	72	M	23.42601	0	0	1	0	1	0	0	0	0	0	0	0	0	1	1	0	0	0	127	130	52	52	2.55	2.35	20.41702	22.32539	10.2	12	3.9	4.3	101	NA	164	NA	36.99634	NA	2920	NA	215	NA	0.18493	0.2	5.5	Class A
738	67	F	16.21879	0	1	0	0	0	1	0	0	0	0	0	0	0	0	0	0	0	0	129	134	88	85	1.44	1.52	28.78155	27.0134	10.8	11.9	3.73	3.97	66	36	86.9	53.3	26.93878	11.21495	199	1880	2060	2040	0.28321	1.78333	5	Class A
739	68	M	20.53878	0	1	0	0	0	1	0	1	0	0	0	0	0	0	0	0	0	0	135	146	75	85	2.35	2.32	22.69465	22.91966	10.5	11.1	3.8	3.6	56	62	321	292	21.875	24.8996	410	314	164	343	1.93309	4.16133	NA	Class B
740	78	M	30.17778	1	0	0	0	0	1	0	0	1	0	0	0	0	1	1	0	0	0	128	120	65	58	2.41	2.62	21.22482	19.37086	8.8	10.8	4.3	4.2	101	NA	121	NA	32.79221	NA	910	NA	69	NA	5e-04	1	7.2	Class A
741	83	M	24.53896	0	0	1	0	0	0	1	1	0	0	0	0	0	1	1	0	0	0	154	172	76	77	1.6	1.66	32.63766	31.34932	9.4	10	2.6	2.6	70	66	290	269	37.83784	35.29412	533	545	1030	873	2.98824	3.61047	NA	Class A
742	71	F	18.5975	0	0	0	1	1	0	0	0	0	0	0	0	0	1	1	0	0	0	134	130	86	84	2.4	2.39	16.18765	16.26177	9.9	11.5	4.2	4.4	73	80	90.8	77.4	29.6748	30.76923	101	163	474	368	3.3	2.46154	5.4	Class B
743	42	M	32.13577	1	0	0	0	0	0	1	1	1	0	0	0	0	1	1	0	1	1	164	175	87	82	2.69	3.69	22.47917	15.90751	9	10.8	2.6	2.7	50	61	299	199	29.41176	38.36478	502	3560	2140	1020	8.32357	4.97849	4.8	Class A
744	81	M	22.96581	0	1	0	0	0	1	0	1	1	1	0	1	0	0	0	0	0	0	148	163	72	71	2.3	2.69	22.09715	18.55189	9	9.9	3.7	3.9	58	78	273	208	33.72093	42.16216	281	293	861	443	3.39024	2.40909	5.7	Class B
745	42	F	22.60715	0	0	1	0	1	0	0	0	0	0	0	0	0	1	1	0	0	1	NA	NA	NA	NA	1.21	NA	39.81131	NA	11.3	NA	4.1	NA	151	94	30.9	79.8	47.78481	36.29344	128	150	20	63	0.09375	NA	5	Class A
746	78	M	23.17554	0	0	0	1	0	1	0	0	0	0	0	0	0	1	1	0	0	0	125	136	52	63	3.33	3.66	14.90104	13.38858	6.9	11.8	3.8	4.1	79	86	373	153	28.62319	30.60498	605	469	806	329	1.93299	1.58672	5.4	Class B
747	76	F	21.64412	0	0	0	1	1	0	0	1	0	0	0	0	0	0	0	0	1	1	152	134	69	79	2.32	2.76	16.47437	13.6238	7	11.6	3.74	4.19	76	117	117	41.1	20.87912	32.14286	1200	414	1570	174	0.49828	0.44474	NA	Class A
748	66	M	16.72419	0	0	1	0	0	0	1	1	0	0	0	0	0	0	0	0	1	1	151	145	87	81	4.05	3.81	12.61935	13.49151	8.7	9.5	4.5	4.4	31	63	60.1	52.4	12.65306	24.70588	2590	2720	199	110	0.55375	0.7074	NA	Class A
749	59	M	24.74048	1	0	0	0	0	0	1	1	1	0	0	0	0	1	0	1	0	0	140	NA	63	NA	2.59	2.95	21.25285	18.3437	8.6	11	2	2.7	45	NA	733	NA	25.71429	NA	3540	NA	667	NA	5.51471	15.74713	5.5	Class A
750	51	M	25.78077	0	0	0	1	0	0	1	0	0	0	0	0	0	0	0	0	0	0	NA	149	NA	95	3.07	3.09	18.39923	18.26899	10.1	9.8	3.2	3.1	100	91	64.4	55.8	43.29004	38.39662	1320	1550	6100	5810	9.09369	9.40913	5.4	Class A
751	64	F	24.62575	0	0	1	0	1	0	0	1	0	0	0	0	0	0	0	0	0	1	178	NA	80	NA	2.57	2.77	15.47411	14.25606	9.8	10.3	NA	4	105	82	22.3	22.3	30.70175	25.625	808	667	354	297	NA	0.71605	NA	Class B
752	71	F	19.06771	1	0	0	0	1	0	0	0	1	0	0	1	0	0	0	0	0	0	115	129	66	69	1.34	1.26	30.62544	32.62769	11.6	11.4	4.2	3.8	126	75	42.8	48.8	36.84211	22.45509	511	2980	82	79	0.0813	0.07353	6.3	Class A
753	80	F	20.24423	0	1	0	0	1	0	0	0	1	0	0	0	0	1	1	0	0	0	130	124	80	80	1.5	1.53	26.15866	25.59805	10.5	14.1	4.4	4.2	92	136	183	117	29.11392	41.71779	857	381	2020	1920	0.14462	0.00556	5.6	Class A
754	59	M	22.11658	1	0	0	0	0	0	1	1	1	0	0	1	1	1	1	0	0	1	144	134	60	70	4.19	4.95	12.5564	10.46331	9.8	11.2	3.9	3.4	95	110	19	33.4	27.53623	36.42384	100	84	508	1120	5.1145	6.97436	6.1	Class C
755	84	F	21.70957	0	0	0	1	1	0	0	1	0	0	0	0	1	1	1	0	0	0	121	124	54	49	2.61	2.44	14.07257	15.14864	9.9	11.4	3.6	3.7	81	112	117	38.3	27.27273	38.62069	87	132	1770	1910	6.32356	6.96612	5.3	Class A
756	75	F	21.42857	1	0	0	0	0	0	1	0	1	0	0	0	1	1	1	0	0	0	126	NA	84	NA	3.44	NA	10.74753	NA	9.7	NA	3.3	NA	59	NA	44.9	NA	24.48133	NA	412	NA	1370	NA	5.99613	NA	9.3	Class B
757	49	M	24.2874	1	0	0	0	0	1	0	0	1	0	0	0	0	1	1	0	0	0	135	133	77	75	2.13	3.34	27.76311	16.97215	9.9	10.8	3.9	3.7	60	53	175	71.9	22.38806	22.74678	15400	356	9080	7430	4.34655	6.11927	6.2	Class A
758	75	F	14.49965	0	0	1	0	1	0	0	1	1	0	0	1	0	0	0	0	0	1	NA	NA	NA	NA	1.06	0.94	38.96	44.4326	9.4	10.9	3.6	3.7	27	22	22.4	38.6	8.359133	7.586207	4710	4620	1770	1440	0.07547	0.08654	5.9	Class A
759	61	F	18.60071	0	0	1	0	1	0	0	1	0	0	0	0	0	1	1	0	0	0	155	163	108	103	1.57	1.56	26.89938	27.08808	9.8	11.5	3.9	4	91	98	132	100	33.95522	32.77592	116	84	481	374	0.77778	0.37736	NA	Class A
760	47	F	17.96875	0	1	0	0	1	0	0	0	0	0	0	0	0	1	1	0	0	0	110	128	78	92	4.68	4.88	8.77617	8.33295	10.5	10.9	4.4	4.1	67	91	16.4	26.7	25.86873	36.54618	190	295	235	209	NA	0.82927	NA	Class A
761	65	F	16.31111	1	0	0	0	0	1	0	0	1	1	0	0	0	1	0	1	0	0	155	145	76	105	3.92	3.71	9.707	10.2646	9.2	10.3	3.1	3.2	75	85	59.2	43.7	26.04167	26.72956	98	397	637	746	9.67059	16.75377	5.7	Class B
762	73	F	31.23698	0	1	0	0	0	1	0	0	0	0	0	0	0	0	0	0	0	0	137	140	87	71	3.02	3.57	12.4895	10.40048	10.8	12.4	4	4.3	68	83	74.8	47	17.80105	20.49383	6760	1310	91	68	0.40187	0.53571	NA	Class A
763	65	M	24.42452	1	0	0	0	0	1	0	0	1	0	0	0	0	1	1	0	0	0	134	157	77	86	1.57	1.9	35.74221	29.00943	9.5	12	2.6	3.5	72	115	237	250	35.64356	41.51625	1310	847	311	137	3.24138	1.71642	12.5	Class A

764	60	F	18.54435	0	0	1	0	1	0	0	0	0	0	0	0	0	1	1	0	0	0	128	140	69	70	3.06	3.18	13.02379	12.428	9.3	11.1	4	4.3	69	84	81.8	97.1	29.48718	32.30769	65	70	467	355	0.36232	0.77586	5.5	Class B
765	85	F	16.04444	0	0	1	0	1	0	0	1	1	0	0	0	0	0	0	0	0	0	150	133	74	59	1.09	1.13	36.4551	34.92829	11	11.5	3.4	3.5	48	81	31.4	26.9	18.18182	27.93103	5250	4600	771	743	1.44444	1.36	NA	Class A
766	69	F	18.91807	0	1	0	0	1	0	0	0	0	0	0	0	0	0	0	0	1	1	120	121	72	72	2.05	2.71	19.31276	14.23097	9.4	10.5	3.1	3.3	77	88	201	168	37.7451	34.24125	538	697	779	870	4.6496	8.04501	NA	Class A
767	68	M	22.51792	1	0	0	0	0	1	0	0	1	0	0	0	0	1	1	0	1	1	113	128	55	60	1.99	1.78	27.2224	30.75475	9.6	10.3	3	3	78	76	64.4	57	34.21053	34.08072	119	268	213	477	2.08696	2.09945	6.4	Class A
768	58	M	23.84721	1	0	0	0	0	0	1	0	1	0	0	1	0	0	0	0	0	1	123	153	75	87	3.08	4.13	17.66947	12.81883	9.5	10	2.8	2.6	37	NA	36	NA	14.01515	NA	3880	NA	2610	NA	9	11.76667	5.8	Class B
769	71	F	18.66449	0	0	1	0	1	0	0	1	0	0	0	1	0	1	1	0	1	1	146	133	96	84	5.29	5.34	6.81828	6.74846	10.3	11	3.8	3.6	81	80	43.9	44.8	28.82562	28.16901	510	1160	3470	4360	NA	0.73438	5.7	Class B
770	47	F	NA	0	0	0	1	0	0	0	0	0	0	0	0	0	1	1	0	0	0	120	140	82	88	3.21	NA	13.25677	NA	10.1	NA	4.2	NA	80	102	42.5	17.4	30.07519	36.55914	153	167	391	298	2.28125	NA	NA	Class A
771	70	F	NA	0	0	1	0	0	0	0	0	0	0	0	0	0	0	NA	NA	NA	0	NA	NA	NA	NA	3.37	NA	11.21181	NA	9.9	NA	NA	NA	NA	NA	NA	NA	NA	NA	NA	NA	NA	NA	NA	NA	NA	Class A
772	77	F	20.04452	0	0	0	1	1	0	0	0	0	0	0	0	0	1	1	0	0	0	126	111	64	72	2.18	2.22	17.5692	17.22317	8.7	9.7	3.5	3.8	55	92	144	89.4	22.63374	32.97491	1450	183	653	575	1.04651	0.99349	NA	Class A
773	84	F	20.53881	1	0	0	0	0	1	0	0	0	1	0	0	0	1	1	0	0	0	126	138	62	70	4.53	4.92	7.69851	7.0096	6.8	8.3	NA	NA	43	NA	31.1	NA	17.7686	NA	157	NA	3880	NA	1.84211	NA	4.8	Class C
774	73	M	22.67574	0	0	0	1	0	1	0	0	1	1	0	0	0	1	1	0	1	1	120	128	80	74	2.35	2.18	22.23719	24.047	9.9	12	2.8	2.6	79	102	44.4	48.3	27.14777	37.22628	93	294	956	1150	12.96207	14.8098	6.3	Class A
775	68	F	24.0594	0	0	1	0	0	1	0	0	0	0	0	0	0	1	1	0	1	1	131	140	80	83	1.33	1.36	31.26241	30.50876	9.5	12.5	4.1	3.9	110	68	292	125	40.74074	26.77165	402	332	123	284	0.06122	0.06875	NA	Class A
776	64	F	22.60026	0	0	1	0	1	0	0	0	0	0	0	0	0	1	1	0	1	1	120	126	62	83	1.46	1.79	28.72569	22.98536	10.1	13	3.2	3.3	49	70	121	104	20.08197	26.71756	1310	1310	881	613	3.47518	2.10672	NA	Class A
777	75	M	NA	0	1	0	0	0	1	0	0	1	1	0	0	0	1	1	0	0	0	94	115	42	47	1.32	1.54	41.47165	34.90282	10.9	11.5	3.4	3.9	45	NA	101	NA	15.625	NA	6240	NA	2320	NA	NA	0.50584	6.8	Class A
778	74	F	27.63788	0	0	1	0	1	0	0	0	1	0	0	0	1	1	1	0	0	1	134	111	60	49	1.26	1.63	32.24766	24.33158	9.4	11.9	4.1	4.3	53	71	65.3	73.1	21.45749	26.79245	131	170	91	90	0.61697	0.30516	6.6	Class A
779	64	M	18.69707	0	0	0	1	1	0	0	1	0	0	0	0	0	1	1	0	0	0	143	142	90	95	3.29	3.46	15.98143	15.12442	10.9	11.5	4.2	4	102	61	63.1	24.8	31.09756	17.9941	180	150	261	95	0.23729	0.22018	NA	Class A
780	86	F	17.58044	0	0	1	0	1	0	0	0	0	0	0	0	0	0	0	0	0	0	125	130	58	61	1.24	1.25	31.55308	31.17343	10.5	12.3	4.3	4.1	59	70	432	343	26.33929	31.67421	1470	2580	1740	889	0.26923	0.15789	NA	Class A
781	53	F	19.52006	0	0	0	1	1	0	0	0	0	0	0	0	0	1	0	1	0	1	103	119	66	84	3.02	2.77	13.69151	15.04895	9.1	10.5	4.4	4.4	76	80	115	84.9	24.12698	25.72347	54	56	122	255	0.46038	0.75758	NA	Class A
782	76	F	25.64922	0	0	0	1	1	0	0	0	0	0	0	0	0	0	0	0	1	1	122	132	64	80	3.23	3.11	11.42762	11.91087	8.6	10.1	3.2	3.3	111	86	21.9	49.3	36.39344	29.2517	286	398	439	716	3.76812	5.28571	NA	Class B
783	68	F	23.65781	0	0	1	0	1	0	0	0	1	0	0	0	0	0	0	0	0	0	118	116	60	68	3.98	4.3	9.42419	8.62347	9.2	11	4.4	4.2	80	53	187	48.7	28.16901	16.30769	130	266	56	63	0.90257	NA	5	Class A
784	54	F	24.04444	0	0	1	0	1	0	0	0	0	0	0	0	0	1	1	0	0	1	129	130	81	85	5.14	4.78	7.61124	8.24053	10	12.3	4.2	4.2	77	85	61.2	67.6	27.11268	26.15385	360	549	340	469	0.75	2.02	NA	Class B
785	69	M	24.62443	1	0	0	0	0	0	1	1	1	1	0	1	1	1	1	0	0	0	181	NA	82	NA	3.72	NA	13.67345	NA	9.7	NA	3.2	NA	42	NA	138	NA	23.86364	NA	3840	NA	676	NA	5.41516	NA	6.4	Class B
786	86	M	24.92188	0	0	1	0	1	0	0	1	0	0	0	0	0	0	0	0	0	0	154	155	62	54	2.12	2.54	23.74593	19.48555	9.5	10.6	NA	3.5	66	86	37	33.3	22.22222	26.95925	1140	726	793	964	3.41667	1.03077	NA	Class B
787	92	F	NA	0	1	0	0	1	0	0	0	1	0	0	0	0	1	1	0	0	0	NA	NA	NA	NA	2.48	2.59	14.49803	13.82576	7.9	8.5	NA	3.9	68	40	230	172	34	20.1005	206	642	3490	2670	NA	4.70204	NA	Class A
788	83	F	33.03434	1	0	0	0	0	1	0	0	1	1	0	0	1	1	1	0	0	0	144	156	60	60	1.83	1.43	20.82329	27.27301	9.3	11.1	3.9	4.3	53	113	105	91.6	21.37097	36.10224	2050	62	186	203	0.19338	0.3363	5.7	Class A
789	76	M	24.7721	0	0	0	1	0	1	0	0	1	1	0	0	0	1	0	1	0	1	126	115	75	71	5.01	4.6	9.60253	10.5032	9.4	11.5	4.2	4.5	94	94	61.4	71.2	37.90323	31.97279	216	184	331	524	0.50314	1.20683	5	Class A
790	84	F	21.20823	0	0	0	1	1	0	0	1	1	1	0	0	0	0	0	0	0	0	156	142	90	94	2.79	3.33	13.08239	10.78013	9.1	8.8	3.5	3.2	55	56	700	493	22.82158	24.6696	1310	7680	17700	35200	11.93431	9.04762	NA	Class A
791	71	M	18.78005	0	0	0	1	0	1	0	0	0	0	0	0	0	0	0	0	1	1	135	138	80	75	1.27	1.26	43.94701	44.15114	10.2	11.1	3.7	3.5	90	98	50.4	81.1	25.42373	28	4460	710	232	207	0.23904	0.89864	NA	Class A
792	75	F	17.40473	0	1	0	0	1	0	0	0	0	0	0	0	0	0	0	0	1	1	134	115	80	74	1.36	1.19	29.66279	34.32853	10.7	12	3.2	3.4	52	82	38.6	80.6	20.47244	29.4964	141	1930	988	713	0.37662	0.80488	NA	Class A
793	62	M	23.91682	1	0	0	0	0	0	1	1	1	0	0	0	0	1	1	0	0	0	198	215	93	105	4.56	5.57	11.28438	9.06608	9.3	11.3	3.7	3.6	58	62	95.1	45.2	25.77778	27.19298	568	422	3790	3120	4.4293	8.40391	6.1	Class B
794	77	F	15.51079	0	0	1	0	1	0	0	0	0	0	0	0	0	0	0	0	1	1	111	118	64	68	1.56	1.46	25.33641	27.24089	10.7	12.3	4.6	4.2	59	91	208	131	19.86532	30.43478	214	221	359	480	1.02902	4.61735	5.4	Class A
795	68	M	23.12467	0	0	0	1	0	1	0	0	0	0	0	0	0	1	1	0	1	1	122	126	64	62	2.12	2.14	25.40155	25.14195	9.6	10.3	4	3.5	42	151	620	726	18.26087	86.28571	45200	2860	1050	626	1.21569	0.61208	4.7	Class A
796	76	M	23.52861	0	0	0	1	0	1	0	0	1	1	0	0	0	1	0	1	0	0	128	116	59	65	2.99	3.13	16.88978	16.06507	10	12.4	3.9	4	91	143	79.6	49	36.54618	51.25448	291	102	354	253	3.53774	4.64209	5.8	Class A
797	59	M	26.12457	1	0	0	0	0	0	1	0	1	0	0	0	0	1	1	0	0	0	130	150	60	85	2.79	2.63	19.59188	20.79892	10.5	12.1	4.3	3.7	73	116	106	88.1	27.03704	39.72603	559	423	93	301	1.98936	4.24615	7.4	Class A
798	65	M	NA	0	0	1	0	0	1	0	1	0	0	0	0	0	1	1	0	0	0	147	124	86	73	4.75	4.21	10.64616	12.09562	10.3	10.8	4.3	4.4	58	88	126	114	21.56134	33.20755	1120	693	258	472	0.5	1.04545	NA	Class B
799	70	M	21.06105	0	0	0	1	0	1	0	0	0	0	0	0	0	0	0	0	0	0	124	NA	60	NA	4.9	4.85	10.07359	10.18725	10.8	10.6	3.6	3.5	96	97	64	56.6	32.43243	32.22591	124	134	455	715	2.55652	3.91	NA	Class A
800	73	M	NA	0	0	1	0	0	1	0	1	0	0	0	0	0	0	0	0	0	0	147	148	73	85	2.4	NA	21.73086	NA	10.4	NA	3.5	NA	87	NA	181	NA	37.82609	NA	711	NA	730	NA	0.4875	NA	5.4	Class A
801	81	M	24.36804	0	1	0	0	0	1	0	0	0	1	0	0	0	0	0	0	0	0	124	132	71	77	1.79	1.62	29.07002	32.42329	9.3	11.4	3.6	3.8	51	59	99.9	51.5	20.2381	19.93243	2080	1970	1640	885	0.03431	0.0316	NA	Class A
802	31	F	29.3724	0	0	0	1	1	0	0	0	0	0	0	0	0	1	1	0	0	0	125	115	75	75	2.94	2.75	16.44574	17.53227	10.9	11.4	3.8	3.9	73	75	81.3	68	27.34082	28.40909	1060	165	237	169	1.68182	1.68358	NA	Class A
803	87	F	21.94917	0	0	1	0	1	0	0	0	0	0	0	0	0	0	0	0	0	1	126	109	69	60	1.84	1.76	20.42177	21.36923	9.9	11	4.1	4.2	122	83	626	507	50.83333	37.21973	449	1310	365	856	0.53125	0.55556	NA	Class A
804	55	F	26.55492	0	0	1	0	0	0	1	1	0	0	0	0	0	0	0	0	0	0	142	141	91	78	1.51	1.74	28.91754	24.76288	7.1	9.6	3	3.1	62	36	164	27.4	31.15578	15.58442	2960	9560	1910	1690	0.88643	7.83251	NA	Class A
805	76	F	NA	0	1	0	0	0	0	0	0	0	0	0	0	0	0	0	0	0	0	NA	NA	NA	NA	1.42	1.39	28.18702	28.74519	7.7	9.7	3.3	NA	NA	NA	NA	NA	NA	NA	NA	NA	NA	NA	NA	NA	NA	Class A
806	66	F	32.37227	0	1	0	0	0	0	0	1	1	0	0	0	0	1	1	0	1	1	131	135	66	73	3.34	3.11	11.51485	12.39604	10.1	12.4	3.7	3.9	44	86	70.2	66.8	17.6	32.95019	2000	872	1490	1160	1.35154	2.09326	5.8	Class A
807	76	F	23.02526	0	1	0	0	0	1	0	0	1	0	0	0	0	1	1	0	0	0	143	141	79	76	2.04	2.01	18.96345	19.2733	9.5	11.6	3.5	4	78	122	44.2	8.7	29.43396	36.9697	183	126	154	165	0.62766	1.05882	NA	Class A
808	75	M	20.74286	1	0	0	0	0	1	0	1	1	1	0	0	0	1	1	0	0	0	144	132	60	81	3.97	5.24	12.43318	9.17721	7.7	9.7	3.4	3.8	81	68	508	242	38.75598	31.7757	769	402	10300	18000	9.4697	4.02091	5.5	Class C
809	86	F	28.92313	1	0	0	0	0	1	0	0	1	1	0	0	0	0	0	0	0	0	120	151	55	64	2.11	3.05	17.63925	11.78748	9.1	11.4	4	4.5	60	35	79.9	105	21.42857	11.55116	290	11900	974	1090	2.00369	1.76147	5.9	Class A
810	80	M	24.03461	0	1	0	0	0	1	0	0	0	0	0	0	0	1	0	1	0	0	101	98	58	42	2.31	1.89	22.07107	27.48943	8.7	9.5	4	3.6	75	54	145	102	26.1324	23.58079	154	864	215	806	0.25301	0.07115	NA	Class A
811	77	M	24.66005	0	1	0	0	0	1	0	0	0	0	0	0	0	1	1	0	0	0	131	158	57	66	2.42	2.41	21.20728	21.30356	9.6	9.3	3.7	4	64	63	74.8	108	27.70563	31.18812	50	50	1220	1850	1.08602	0.78635	NA	Class A
812	63	F	32.42541	1	0	0	0	0	1	0	0	1	1	0	0	0	1	0	1	0	0	160	125	73	68	1.63	2.98	25.46474	13.16073	9.5	10.9	3.2	4	49	51	178	153	20.85106	17.70833	1030	685	629	271	15.59102	8.38889	6.9	Class A
813	65	F	32.29783	1	0	0	0	0	1	0	0	1	0	0	0	1	1	0	1	0	1	130	118	59	51	2.06	3.46	19.62318	11.12733	8.9	10.7	3.3	3.5	37	37	64.9	90	16.29956	13.26165	26800	9700	4550	865	1.9881	NA	6	Class A
814	63	M	24.71325	1	0	0	0	1	0	0	1	1	0	0	0	0	1	1	0	0	0	111	94	65	63	1.73	1.99	32.43161	27.70019	10.7	11.6	4.2	4.3	54	64	145	151	20.53232	21.99313	1780	447	678	372	0.13333	0.08804	7.1	Class A
815	59	F	19.18073	0	0	1	0	1	0	0	0	0	0	0	0	0	0	0	0	0	0	122	NA	71	NA	3.26	NA	12.21101	NA	9.8	NA	4.5	NA	55	NA	60	NA	17.62821	NA	90	NA	215	NA	0.3681	NA	NA	Class A
816	41	F	20.97448	0	0	0	1	1	0	0	0	0	0	0	0	0	1	1	0	0	0	122	145	76	85	2.37	3	19.21332	14.8459	10.2	11.3	2.9	3	63	83	38.2	22.7	23.50746	30.62731	50	50	771	874	3.2029	4.27778	NA	Class A
817	73	M	23.63657	0	1	0	0	0	1	0	0	0	0	0	0	0	1	1	0	0	0	129	133	64	62	4.32	4.49	11.42375	10.95142	9.6	10.4	4.6	4.4	63	80	84.6	52.3	22.58065	25.15723	154	272	561	526	0.77348	0.7619	5.6	Class A
818	45	M	25.87882	1	0	0	0	0	1	0	0	1	0	0	0	1	1	1	0	0	1	139	121	79	78	4.85	7.2	11.56454	7.50599	9.7	10.9	4.09	3.08	52	47	58.1	56.6	19.11765	21.36364	322	2370	662	331	1.35135	3.73451	5.6	Class B
819	82	M	16.58992	0	0	0	1	0	1	0	0	0	0	0	0	0	0	0	0	0	0	126	104	68	74	3.79	3.83	12.74987	12.60427	7.6	9.1	3	1.7	58	38	651	596	39.72603	43.18182	23000	95200	3960	10400	4.09597	1.67919	NA	Class A
820	81	M	23.77378	0	0	1	0	1	0	0	0	0	1	0	1	0	1	1	0	0	0	118	132	59	84	4.25	4.31	11.28779	11.116	9.3	10.6	3.4	3.4	64	79	143	101	25.80645	31.6	789	1420	1170	1380	3.12726	3.28416	NA	Class B
821	65	M	20.09753	0	0	1	0	1	0	0	1	0	0	0	0	0	0	0	0	0	0	155	125	80	72	2.27	2.58	23.87829	20.75791	8.5	10.8	4.2	4.3	117	119	176	53.9	49.36709	42.04947	2510	88	601	398	1.21972	0.63291	5	Class A
822	73	M	27.11905	1	0	0	0	0	1	0	1	1	1	0	0	0	1	1	0	0	0	171	166	68	76	2.63	2.86	19.66059	17.86766	7.8	9.7	3.7	3.7	97	86	143	63.9	40.41667	32.08955	694	813	1420	2090	3.71106	8.22917	5.7	Class C
823	61	M	24.68401	1	0	0	0	0	1	0	0	1	1	0	0	0	1	1	0	1	1	112	158	73	83	3.84	4.13	13.68213	12.63464	10.2	11.6	3.7	3.5	59	80	102	82.8	25.99119	31.1284	826	377	249	335	1.75148	0.6772	6.3	Class B
824	87	F	24.56348	0	0	0	1	1	0	0	0	1	0	0	0	0	0	NA	NA	NA	0	130	NA	70	NA	1.66	1.35	22.8563	28.65623	9.5	11.8	2.6	3.7	97	106	689	405	43.8914	39.84962	5370	92	1770	282	0.001389	0.000926	NA	Class A
825	66	M	NA	0	0	0	1	0	0	1	0	0	0	0	0	0	0	0	0	0	0	121	NA	69	NA	3.37	3.55	15.42999	14.57615	6.8	13.2	2.7	4.4	46	67	467	102	25.9887	24.45255	7100	356	496	104	0.96203	2.94019	5.2	Class A
826	74	M	25.95664	0	0	1	0	0	0	1	0	1	0	0	0	0	1	1	0	0	0	NA	142	NA	52	2.2	2.08	23.80794	25.31459	10.7	9.3	4.2	3.4	47	55	99.2	262	19.02834	28.06122	4590	6000	80	260	0.11013	0.00595	6.7	Class A
827	67	F	30.33064	1	0	0	0	1	0	0	0	1	0	0	0	0	1	0	1	0	1	128	142	70	88	3.11	3.89	12.39604	9.70416	9	9.9	3.7	3.7	44	42	19.4	27.9	13.96825	15.84906	444	777	362	590	1.03851	0.4648	6.2	Class B
828	53	F	21.45357	0	0	1	0	1	0	0	1	1	0	0	0	0	1	1	0	0	0	138	131	93	84	1.77	1.91	24.56384	22.60104	10.9	11.9	4.4	4.3	47	71	48.5	33.6	14.96815	21.58055	729	348	113	72	0.43902	0.34737	6.1	Class A
829	75	M	35.12397	1	0	0	0	0	1	0	0	1	1	0	0	0	1	0	1	0	0	119	148	60	51	4.74	5.66	10.24136	8.43486	10.1	11.2	3.1	3.4	38	77	48.7	16.8	15.63786	25.83893	591	285	4750	5680	8.35669	8.27927	5.8	Class B
830	48	M	33.1384	0	0	0	1	0	1	0	1	1	0	0	0	0	1	1	0	0	0	166	168	90	90	4.36	5.1	12.7552	10.74493	10.3	11.3	2.9	2.5	64	66	121	73.7	26.55602	30.55556	728	421	1670	3780	8.85502	15.14414	5.3	Class C
831	49	F	26.28861	1	0	0	0	1	0	0	1	0	0	0	0	0	1	0	1	0	0	130	136	54	60	3.3	3.25	12.70883	12.84817	9.6	11.3	3.6	3.6	80	62	356	344	35.55556	29.52381	2370	15900	271	193	0.91922	1.40969	9.4	Class A
832	69	M	20.38761	0	1	0	0	0	0	0	1	1	0	0	1	0	1	1	0	0	0	143	120	71	50	4.85	5.89	10.22941	8.23669	10.6	10.7	4.1	3.6	92	28	185	239	36.65339	13.20755	327	41900	282	718	NA	2.22973	NA	Class C
833	68	F	21.90171	0	0	0	1	1	0	0	0	0	0	0	0	0	1	1	0	0	0	130	120	70	80	2.94	3.01	13.12635	12.73928	9.5	10	4	4.1	75	92	66.5	43.7	29.52756	33.82353	592	69	102	111	0.99091	0.86	NA	Class A
834	53	M	20.5235	0	0	1	0	0	1	0	0	0	0	0	0	0	1	1	0	0	0	110	NA	80	NA	2.73	3.05	20.69058	18.32781	12.2	12	4.3	3.8	60	50	54.7	75.9	16.94915	15.19757	850	164	134	126	0.37793	0.44723	5.6	Class A
835	68	M	24.27916	0	0	1	0	0	1	0	0	0	0	0	0	0	1	1	1	0	0	128	121	78	57	4.4	3.97	11.42706	12.78777	10.4	11.1	4.2	3.6	115	59	288	220	35.71429	28.78049	930	9120	440	634	2.68757	3.52911	5.4	Class A
836	58	F	27.54689	0	0	1	0	1	0	0	0	1	0	0	0	0	1	1	0	0	0	127	NA	79	NA	2.28	NA	18.14519	NA	10.4	NA	3.9	NA	87	76	97	79.4	29.39189	22.09302	5840	1160	121	78	0.05263	NA	NA	Class A
837	76	F	20.79598	0	0	0	1	1	0	0	0	0	0	0	0	0	0	0	0	0	0	137	131	71	71	3.06	3.18	12.16952	11.66803	10.1	10.2	3.9	3.5	99	NA	18.9	NA	32.56579	NA	204	NA	671	NA	0.99756	5.11765	NA	Class C
838	69	M	21.95248	0	0	0	1	0	1	0	1	0	1	0	1	0	1	1	0	0	0	154	132	74	76	5.37	5.73	9.15082	8.52375	9.8	11.6	3.7	3.7	92	106	166	45.5	37.70492	41.73228	911	473	878	670	3.37662	3.19471	NA	Class B
839	56	M	34.68094	1	0	0	0	0	0	1	0	1	0	0	0	0	1	1	0	0	0	117	140	59	60	4.43	3.89	11.9924	13.82506	9.5	10.4	3.3	3.3	33	68	15.6	28.8	9.482759	21.05263	1980	1780	7790	17200	7.91053	4.87905	6	Class A
840	68	M	NA	0	0	1	0	1	0	0	1	1	0	0	0	0	1	1	0	0	0	148	130	58	60	2.83	3.2	18.51899	16.18966	10.7	11.5	4.2	3.8	110	70	45	61.9	33.63914	22.01258	182	295	411	483	6.01538	4.33735	5.4	Class A
841	81	M	28.08594	0	0	1	0	1	0	0	0	0	0	0	0	0	0	0	0	0	0	117	122	45	67	1.81	NA	28.71879	NA	10.4	NA	3.8	NA	68	76	342	246	28.45188	32.47863	586	3010	308	218	1.57927	NA	6.5	Class A
842	67	M	28.89778	1	0	0	0	0	0	1	0	1	0	0	0	0	1	1	0	0	0	102	104	40	47	3.78	2.97	13.55009	17.64098	7.8	9.8	2.98	3.25	147	178	470	406	44.01198	58.36066	212	481	579	859	4.19219	2.99691	5.6	Class A
843	74	F	29.21997	0	1	0	0	0	1	0	1	0	0	0	0	0	1	1	0	0	0	147	123	86	68	2.41	2.4	15.92392	15.99652	10.2	10.5	3.7	3.4	71	86	76.4	68.3	23.35526	31.38686	4320	1940	245	251	0.04	0.15789	6.1	Class A
844	51	M	19.79503	1	0	0	0	1	0	0	0	1	0	0	0	0	0	0	0	1	1	135	167	80	108	1.44	1.62	42.11924	36.82132	8.4	9.9	2.9	4.1	12	109	47.2	49.7	3.498542	28.31169	95800	8240	356	396	0.62407	1.15854	8.8	Class A
845	37	M	26.19609	1	0	0	0	0	1	0	0	1	0	0	0	0	1	1	0	0	0	125	155	68	82	2.76	3.14	22.66592	19.6828	9.8	10.3	3.1	2.6	48	49	40.7	89.6	18.97233	21.68142	4620	12300	111	59	6.51422	2.18271	6.7	Class A
846	78	F	25.76351	1	0	0	0	0	1	0	0	1	1	0	0	0	0	0	0	0	0	155	148	75	79	3.5	NA	10.42809	NA	7.5	NA	4	NA	54	NA	52.1	NA	18.18182	NA	141	NA	4220	NA	3.22981	NA	5.6	Class A
847	74	F	21.88396	1	0	0	0	0	1	0	0	1	1	0	0	0	0	0	0	1	1	152	129	62	36	4.22	4.77	8.6275	7.54532	9.1	8.5	3.4	3.8	65	38	40	53.1	24.34457	16.81416	164	815	1970	4710	11.70819	5	5.6	Class C
848	43	F	14.60349	0	0	1	0	1	0	0	0	1	0	0	0	0	0	0	0	1	1	81	77	57	58	2.58	2.21	17.27145	20.45858	9.8	11.5	3.7	3.9	84	77	10.5	17.4	25.84615	22.25434	87	78	135	126	0.38352	0.11295	NA	Class B
849	52	F	20.62788	0	0	1	0	1	0	0	0	0	0	0	0	0	1	1	0	0	0	107	118	63	75	1.23	1.28	36.77875	35.20996	9.2	11.1	4.25	4.85	79	90	51.4	45.4	25.48387	29.31596	127	68	131	57	0.17341	0.39867	5.1	Class A
850	74	F	20.16158	0	1	0	0	1	0	0	0	0	0	0	0	0	1	1	0	1	1	132	132	NA	NA	3.26	6.9	11.44239	5.0382	9.1	10.3	2.9	3.5	64	102	29.6	37.9	23.27273	37.5	1310	898	1720	2130	9.20664	6.72751	NA	Class B
851	71	F	18.59104	0	0	0	1	1	0	0	0	0	0	0	0	0	1	1	0	0	0	110	126	60	75	2.13	1.9	18.44539	20.81782	10.6	11.8	4.1	3.6	95	113	138	112	34.29603	41.09091	80	72	269	201	0.87234	1.57447	NA	Class A
852	65	F	24.1693	0	1	0	0	1	0	0	1	0	0	0	0	0	1	1	0	0	1	140	149	77	81	1.6	1.66	25.87217	24.85088	9	11.5	4.2	4.4	73	107	33.6	47.8	24.49664	32.32628	83	170	179	190	0.30016	0.8078	NA	Class A
853	92	F	17.64619	0	1	0	0	0	1	0	1	0	0	0	0	0	0	0	0	0	0	149	148	86	80	2.86	2.95	12.40437	11.99096	10.9	11.8	4.5	4.5	65	79	74	41.8	25.39063	27.91519	430	207	612	456	2.45614	5.93407	5.4	Class A
854	73	M	19.92227	0	0	1	0	0	1	0	0	1	0	0	0	1	1	1	0	0	1	131	124	62	83	2.73	2.64	18.87409	19.57913	9.6	11.3	4.1	4.2	51	67	18.3	44	13.28125	20.9375	6900	3440	3050	2560	0.38095	0.33248	6.4	Class A
855	79	F	29.06865	1	0	0	0	0	1	0	0	1	1	0	0	0	1	0	1	0	1	128	133	64	54	1.69	1.9	22.95891	20.19775	10.6	10.1	4.1	4	73	51	26.2	16.3	20.50562	12.53071	2040	827	732	631	0.19169	0.04324	7	Class A
856	75	F	20.95017	0	0	1	0	1	0	0	0	0	0	0	0	1	0	0	0	0	0	NA	NA	NA	NA	2.75	2.59	13.7301	14.60504	9.9	10.9	3.1	3.8	18	87	248	135	8.910891	33.46154	69200	225	3060	3700	0.90179	2.96667	NA	Class A
857	39	M	21.18764	0	0	0	1	0	1	0	0	0	0	0	0	0	1	1	0	0	0	130	136	70	68	4.41	4.78	13.37058	12.15392	7.8	10.8	3.7	3.6	83	106	145	70.1	36.08696	45.68966	408	692	124	204	1.22465	2.93509	NA	Class A
858	76	F	28.84695	0	0	1	0	1	0	0	1	0	0	0	0	1	1	0	1	0	0	115	NA	58	NA	1.02	NA	40.48033	NA	11.8	NA	4.2	NA	119	92	96	129	46.12403	38.01653	383	266	197	216	0.0274	NA	5.6	Class A
859	63	M	23.53483	0	1	0	0	0	0	1	0	0	1	0	0	1	1	0	0	0	1	138	135	74	70	3.51	3.28	14.95633	16.03475	10.5	12.1	3.9	3.5	54	64	104	62	17.64706	20.71197	594	303	853	1050	3.25555	4.71866	NA	Class A
860	75	M	23.36568	0	1	0	0	0	0	1	1	1	0	0	0	0	1	1	0	0	0	160	180	62	NA	2.92	4.04	17.39924	12.1977	8.7	9	2.7	3.2	40	155	166	372	18.01802	67.98246	1570	773	2730	2880	9.22024	1.02236	5.5	Class C
861	69	M	34.03751	1	0	0	0	0	1	0	0	1	1	0	0	0	1	1	0	0	1	136	NA	90	NA	4.54	NA	10.99595	NA	8.9	NA	NA	NA	75	NA	76.3	NA	27.27273	NA	197	NA	478	NA	2.38267	NA	6.5	Class B
862	75	M	24.90681	0	0	0	1	0	0	0	0	0	0	0	0	0	0	0	0	0	0	NA	NA	NA	NA	2.3	4.64	22.59066	10.48307	8.1	8.6	3.7	3.8	28	82	50.7	77.1	11.38211	34.89362	3630	208	1670	4410	0.52564	0.7234	NA	Class A
863	81	M	21.19321	0	0	0	1	0	1	0	0	0	0	0	0	0	1	1	0	1	1	114	120	57	59	2.64	3.29	19.00342	14.93668	8.1	10.7	2	2	47	60	263	105	23.26733	27.64977	11700	1430	1330	1060	13.73016	16.92308	NA	Class B
864	84	M	25.17107	0	1	0	0	1	0	0	1	0	0	0	0	0	0	0	0	0	0	130	NA	72	NA	2.58	2.72	19.28509	18.20184	10.1	11.4	4	4.1	NA	117	NA	138	NA	41.93548	NA	1190	NA	447	0.79032	0.69	NA	Class A
865	83	M	22.19196	0	1	0	0	1	0	0	0	0	0	0	0	0	1	1	0	0	0	112	126	68	69	3.35	3.42	14.54211	14.2168	8	10.1	3.49	3.83	68	90	78.2	46.2	28.21577	29.22078	124	155	2080	2060	0.97354	2.77926	5.6	Class A
866	85	M	22.46879	0	1	0	0	1	0	0	1	0	0	0	0	0	0	0	0	0	1	197	153	90	83	3.8	4.9	12.54057	9.49568	9.4	11.4	3.6	3.5	65	76	25.6	77.8	24.34457	33.48018	749	69300	675	2030	1.93458	NA	5.6	Class A
867	71	M	21.31069	0	0	1	0	1	0	0	0	0	0	0	1	0	1	1	0	0	0	119	125	58	74	3.75	3.98	13.44313	12.59559	9.6	10.5	3.3	3.3	100	41	46.5	8.2	32.89474	11.9883	517	126	1080	958	2.02634	0.79909	NA	Class A
868	60	F	17.63892	0	0	1	0	1	0	0	0	0	0	0	0	0	1	1	0	0	0	135	127	83	73	2.36	2.36	17.30418	17.30418	10.4	11.7	4.4	4.4	82	127	70.6	84.3	27.06271	43.49315	167	194	154	173	1.57692	1.32836	NA	Class A
869	84	F	17.82579	0	0	0	1	1	0	0	0	1	0	0	0	0	1	1	0	0	0	NA	125	NA	71	1.48	1.17	26.17651	33.85187	9.7	11.7	3.3	3.1	69	100	111	30.5	22.92359	33.33333	50	166	390	423	2.55462	2.97376	5.5	Class A
870	83	F	20.72633	0	1	0	0	0	0	0	1	0	0	0	0	0	1	1	0	0	0	152	126	40	40	1.5	1.44	25.88373	27.06588	7.1	8.3	NA	3.6	36	58	175	66.7	14.00778	20.13889	11300	2130	2660	1570	0.09183	0.15323	NA	Class A
871	75	M	22.54632	0	1	0	0	0	1	0	0	0	0	1	0	0	0	0	0	0	1	144	134	81	91	3.12	3.16	16.18281	15.95885	10.8	12	3.8	3.7	110	99	42.9	82.1	41.1985	35.35714	109	155	396	74	0.18605	0.34848	NA	Class A
872	83	F	24.65303	1	0	0	0	0	1	0	0	1	1	0	0	0	1	1	0	1	1	115	113	56	66	1.43	1.26	27.27301	31.21567	10.7	11.5	3.9	3.7	115	66	18.1	24.8	36.50794	22.14765	430	492	81	87	0.02469	NA	7.4	Class A
873	79	M	27.30334	1	0	0	0	0	1	0	1	1	1	0	0	0	1	0	1	0	0	140	127	73	68	2.56	2.5	19.79558	20.31591	8.6	9.3	3.7	3.7	80	74	74.4	53.8	27.87456	24.42244	1750	652	1330	1070	2.99401	6.15254	6.1	Class C
874	73	F	23.14646	0	0	0	1	1	0	0	1	0	0	0	0	0	1	1	0	1	1	132	138	68	90	2.03	1.02	19.28732	40.79135	9.8	11.6	3.5	3.8	239	182	41.5	52.4	67.32394	50.69638	1120	1140	28	33	0.16219	0.27553	5.3	Class A
875	65	F	21.75563	0	0	0	1	1	0	0	0	0	0	0	0	0	1	1	0	0	1	124	141	70	79	4.98	4.89	7.47087	7.5881	8.1	10.2	4.18	4.53	59	98	51.1	67.1	21.61172	35.12545	256	88	919	787	1.69972	1.63459	5.3	Class A
876	63	F	25.76169	1	0	0	0	0	1	0	1	1	0	0	0	0	1	1	0	0	1	172	186	85	81	4.86	5.93	7.74204	6.2275	10.6	10.7	3.8	3.3	58	38	79.5	96.3	22.83465	18.71921	3980	21300	644	5860	7.50649	9.60294	6.5	Class C
877	79	M	20.72403	0	1	0	0	0	0	1	0	0	1	0	0	0	1	1	1	0	1	113	103	66	63	1.87	2.22	27.91181	23.13516	9	10.7	4.4	4.36	81	64	11.8	40.8	27.64505	25.70281	2880	717	1210	1310	0.7618	0.53208	6.4	Class A
878	73	M	23.46099	0	1	0	0	0	1	0	1	0	0	0	0	0	1	1	0	0	0	155	147	90	84	3.31	3.99	15.22791	12.41275	10.8	12.3	4.4	4.3	89	113	34.3	48.8	29.86577	34.76923	50	50	268	323	0.8153	0.99788	NA	Class A
879	77	F	20.3144	0	0	1	0	1	0	0	0	1	1	0	1	1	1	0	0	0	1	111	158	53	99	1.12	NA	36.40649	NA	10.5	NA	3.5	NA	64	38	147	126	30.18868	18.18182	422	880	278	274	14.82	NA	5.5	Class B
880	90	F	NA	0	0	1	0	1	0	0	0	0	1	0	0	0	0	0	0	1	1	108	141	62	65	2.9	3.51	12.29462	9.97729	7.2	11.1	3.4	3.7	45	81	35.8	59.4	14.56311	29.03226	482	1330	2010	7010	0.97421	10.40541	NA	Class A
881	77	M	22.54658	0	0	0	1	1	0	0	0	0	0	0	1	1	1	0	0	1	1	118	132	69	77	3.05	3.5	16.46474	14.16343	10.2	11.5	3.8	3.7	68	65	129	72.2	26.25483	27.77778	317	1050	1180	1650	6.06864	4.23326	5.5	Class B
882	68	M	29.9757	0	0	0	1	0	1	0	0	0	0	0	0	0	1	1	0	0	0	127	124	76	76	5.72	5.68	8.57591	8.64201	10.9	11.1	3.9	3.8	84	63	35.2	28.4	27.00965	20.06369	675	438	222	307	1.06067	0.77626	NA	Class B
883	80	F	34.72222	0	1	0	0	1	0	0	1	0	0	0	0	0	1	1	0	0	0	130	124	70	60	2.12	2.23	17.91628	16.95172	9.6	9.8	3.5	3.7	32	62	6.6	7.2	9.411765	16.93989	2170	1810	658	554	0.33333	0.35955	NA	Class A
884	81	M	24.94031	0	0	0	1	1	0	0	0	0	0	0	0	0	1	1	0	0	0	116	113	60	51	3.55	3.51	13.74411	13.91556	9.6	10.6	3.8	3.9	69	75	93.7	68.5	23.46939	24.91694	901	401	5090	3540	0.79787	1.24194	5.8	Class B
885	70	F	21.21616	0	0	1	0	1	0	0	0	0	0	0	0	0	0	0	0	1	1	95	121	53	60	1.41	1.4	29.08421	29.19248	10	11.5	4	3.9	65	121	76.4	89.3	21.24183	37.11656	159	116	134	195	0.18519	0.20672	NA	Class A
886	65	M	23.91582	0	0	0	1	1	0	0	0	0	0	0	0	0	1	1	0	0	0	112	126	63	93	3.37	4.01	15.49774	12.81314	10	11.7	3.9	3.7	75	57	154	143	31.51261	22.35294	163	1150	169	272	2.49825	4.51373	NA	Class B
887	90	M	25	0	0	0	1	0	1	0	0	1	0	0	0	1	1	1	0	0	0	114	94	63	59	2.75	2.93	17.6321	16.39848	11.1	12.4	4	4	137	101	158	90.6	49.81818	33.55482	3660	4890	9890	8560	0.59701	0.62084	6.8	Class A
888	81	M	25.17012	0	1	0	0	1	0	0	0	0	0	0	0	0	1	1	0	0	0	127	153	65	63	2.03	1.66	25.33179	31.56954	11.3	12.8	4.2	3.9	75	98	289	214	26.22378	36.16236	220	317	353	386	0.69154	1.43836	5.1	Class A
889	75	M	25.41705	0	1	0	0	0	1	0	0	0	0	0	0	0	1	1	0	0	0	120	158	56	80	2.16	2.56	24.19729	20.01676	10.7	13.4	4	3.8	81	44	75.8	57.5	33.89121	16.85824	771	1250	817	1150	2.38424	4.34302	NA	Class A
890	77	M	NA	1	0	0	0	0	1	0	0	1	1	0	0	1	0	0	0	1	1	154	162	66	67	2.47	2.26	20.73807	22.85516	9.5	10.5	3.2	3.6	36	53	129	89.1	21.42857	28.34225	534	302	1080	1400	0.95969	1.86567	5.9	Class B
891	72	F	36.83424	1	0	0	0	0	1	0	0	1	1	0	0	0	1	1	1	0	1	152	135	52	57	3.08	3.91	12.27205	9.45258	9.1	11	2.9	3	65	56	26.3	26.6	24.25373	20.74074	540	788	2230	2500	5.69876	3.93248	5.5	Class C
892	43	M	19.95302	0	1	0	0	0	0	1	0	1	0	1	0	0	1	1	0	0	0	145	128	83	76	6.03	4.22	9.23271	13.64283	9.4	10.9	2.6	4	69	86	229	105	26.95313	33.46304	2130	50	1790	351	3.93542	2.30326	5.4	Class A
893	84	F	23.13851	1	0	0	0	0	1	0	0	1	1	0	0	0	0	0	0	0	0	104	118	45	63	4.72	5.42	7.36013	6.32678	8.1	9.9	3.6	3.7	41	63	123	88.2	16.87243	23.24723	609	131	2530	1980	3.86712	NA	8	Class A
894	68	M	27.19052	1	0	0	0	0	1	0	1	1	0	0	0	0	1	1	0	0	0	184	162	94	72	2.68	2.39	19.65587	22.27945	10.3	11.4	4.2	4.2	52	59	211	169	18.90909	19.53642	284	431	232	213	1.72526	2.44887	5.4	Class A
895	84	F	22.76334	0	0	0	1	1	0	0	1	1	0	0	0	0	0	0	0	0	0	144	115	67	59	1.7	1.5	22.49401	25.79492	10.3	12.3	4	4.2	61	69	207	160	21.32867	22.25806	467	1000	503	536	0.08451	0.60579	5.2	Class A
896	63	F	12.35524	0	0	1	0	1	0	0	0	0	0	0	0	0	0	0	0	0	1	96	99	60	51	2.03	1.5	20.12031	27.88873	10.2	12.7	3.9	4	54	70	22.7	27.7	21.01167	27.13178	207	134	254	203	0.78989	0.67873	NA	Class A
897	52	F	25.59374	0	0	1	0	1	0	0	0	0	0	0	0	0	1	1	0	1	1	135	135	85	85	4.11	4.36	9.82676	9.21202	10.6	10.4	4.2	4	59	71	110	94.7	24.48133	31.14035	2870	3590	116	262	1.09412	1.27778	5.5	Class A
898	84	F	NA	1	0	0	0	1	0	0	0	1	0	0	0	0	0	0	0	0	0	NA	166	NA	74	2.13	2.09	17.57643	17.94477	10.5	9.5	3.8	3.9	72	NA	249	NA	29.87552	NA	245	NA	1560	NA	NA	4.51775	8.7	Class A
899	79	M	23.65093	1	0	0	0	0	1	0	1	1	0	0	0	0	0	0	0	0	0	140	103	74	67	1.95	1.84	26.56544	28.30768	10.8	11.6	3.4	3.5	78	86	123	82	32.5	33.33333	574	643	187	58	0.525	NA	8.5	Class B
900	73	M	NA	0	0	0	1	0	1	0	1	1	0	0	0	0	0	0	0	0	1	159	134	70	64	3.13	5.65	16.25184	8.51701	10.6	11.1	3.7	3.6	69	59	63.4	52.8	27.6	23.04688	968	830	1200	2030	NA	2.00926	6.6	Class A
901	85	M	26.22523	0	0	1	0	1	0	0	1	0	0	0	0	0	1	0	1	0	0	150	NA	87	NA	2.32	NA	21.51577	NA	11.7	NA	3.5	NA	102	87	202	242	37.91822	34.11765	316	293	249	290	NA	NA	6.1	Class A
902	61	F	21.21796	0	0	0	1	1	0	0	0	0	0	0	0	0	1	0	1	0	0	124	134	76	76	3.03	2.83	13.10262	14.11894	9.7	10.6	4.3	4.3	79	77	130	75.9	26.87075	25.49669	200	126	403	289	NA	0.56026	NA	Class A
903	69	M	20.33833	0	0	0	1	1	0	0	0	0	0	0	0	0	1	1	1	0	0	120	110	70	70	2.6	2.91	20.23351	17.88764	11	12.8	3.82	3.92	89	96	429	218	36.47541	32.43243	1520	109	207	317	2.53468	3.30994	5.3	Class A
904	70	M	22.13188	0	0	0	1	1	0	0	1	0	0	0	0	0	0	0	0	0	0	150	143	58	68	5.34	7.36	9.13189	6.42875	9.8	10.2	3.6	3.6	89	99	214	244	37.23849	38.82353	128	224	343	485	3.89371	5.22792	5.7	Class B
905	79	M	20.93775	0	0	1	0	0	1	0	0	0	1	0	0	0	0	0	0	1	1	NA	132	NA	77	2.34	2.37	21.8404	21.53813	9.5	11.9	3.1	3.3	53	66	152	97.1	23.04348	34.55497	6950	7940	1490	6110	0.09188	0.28261	5.9	Class A
906	85	M	20.12666	1	0	0	0	0	1	0	1	1	0	0	1	1	1	0	0	0	0	158	162	76	81	4.46	4.04	10.56054	11.76731	9.4	10.3	3.6	3.4	49	45	109	78.8	24.5	22.38806	1400	2320	1010	1300	0.6236	0.85005	5.1	Class A
907	44	M	19.81198	0	0	1	0	0	0	0	1	0	0	0	0	1	0	0	0	0	0	120	146	77	99	2.2	1.66	27.63888	37.61253	10.1	10.1	3.9	3.7	74	54	131	135	25.87413	22.59414	3530	2650	433	277	NA	NA	6.9	Class A
908	76	F	NA	1	0	0	0	1	0	0	0	1	0	0	0	0	0	0	0	0	0	NA	NA	NA	NA	1.45	NA	27.54965	NA	11	NA	3.5	NA	NA	NA	NA	NA	NA	NA	NA	NA	NA	NA	0.51351	NA	NA	Class A
909	69	M	20.30741	0	0	0	1	0	1	0	0	0	0	0	0	0	1	1	0	0	0	117	126	68	80	4.1	3.92	12.29324	12.91211	8.8	9.9	3.6	3.4	43	56	299	285	23.75691	32.18391	8290	836	1050	1040	0.4598	0.31193	NA	Class A
910	77	F	18.96467	0	0	0	1	1	0	0	0	1	0	0	0	0	0	0	0	0	0	101	100	74	92	2.06	1.75	18.69186	22.34291	10.2	10	4	3.9	31	26	22.6	17.3	7.487923	6.718346	233	85	3250	2740	0.27829	0.40201	7.1	Class A
911	84	M	22.65625	0	1	0	0	0	1	0	1	0	0	0	0	0	0	0	0	0	0	160	118	93	71	2.59	3.37	19.20364	14.34932	8.9	8.9	3.8	3.6	78	72	444	286	29.65779	28.91566	1350	1780	2330	1880	2.85039	2.38554	NA	Class C
912	73	F	25.10957	1	0	0	0	0	0	0	1	1	0	0	0	0	1	1	0	0	0	144	132	76	74	3.11	2.8	12.09463	13.56693	10.8	10.7	3.9	3.8	57	57	27.7	20.1	20.87912	20.65217	2290	271	2670	2930	1.02109	NA	NA	Class A
913	73	M	25.2092	0	0	0	1	0	1	0	0	0	0	0	0	0	1	1	0	0	0	123	112	67	65	4.74	5.28	10.32111	9.17205	9.6	10.8	4.1	3.7	69	93	128	142	25	36.32813	1810	2330	466	683	1.38824	0.98097	6.5	Class A
914	48	M	31.60814	1	0	0	0	0	0	0	1	1	1	1	0	0	1	1	0	0	0	156	156	103	102	5.06	6.71	10.83789	7.9119	10.3	10.1	3	3.2	47	52	266	189	23.15271	26.26263	5580	10800	1970	13800	10.29825	9.10169	6.9	Class C
915	72	F	30.17882	0	0	1	0	1	0	0	1	1	0	0	0	0	1	1	0	0	1	157	133	88	82	1.36	1.55	30.01236	26.01171	9.9	12	3.8	4	84	82	16.9	37.9	30.10753	31.29771	916	1100	317	323	2.25	3.14085	7.4	Class A
916	71	M	19.81405	0	0	1	0	0	1	0	0	0	0	0	0	0	1	1	1	0	0	138	129	67	70	2.89	3.33	17.87592	15.24731	10.2	12.1	3.5	3.5	61	100	36.6	35.1	25.52301	40.48583	709	392	1190	606	1.5969	1.80153	NA	Class A
917	62	M	30.00918	1	0	0	0	0	1	0	1	1	0	0	0	0	1	0	1	0	0	144	121	82	74	2.98	3.45	17.97185	15.31127	10.7	12.3	3.9	3.8	66	67	107	43.8	24.17582	22.78912	1960	1590	348	343	5.35181	7.05882	7.4	Class B
918	92	M	24.2787	0	0	1	0	1	0	0	0	0	0	0	0	0	1	1	0	1	1	131	136	66	67	1.26	1.13	41.15184	46.35823	10.4	12.7	3.7	3.9	105	132	74.7	44.3	30	38.26087	181	50	243	262	0.6673	1.46751	NA	Class A
919	73	F	20.82872	0	1	0	0	1	0	0	0	1	0	0	0	1	1	1	0	0	0	124	NA	66	NA	1.91	1.73	20.61684	22.88517	11.6	10.7	4.2	3.9	41	49	45.6	34	15.95331	18.56061	1440	805	276	396	0.68077	0.68118	6.5	Class A
920	77	M	23.18339	1	0	0	0	0	1	0	0	1	0	0	1	0	0	0	0	0	0	138	140	80	80	3.7	4.64	13.32804	10.40419	10.8	11.8	3.6	3.6	84	94	234	194	30.21583	35.07463	197	444	594	1110	4.35453	4.61988	6.7	Class B
921	67	M	22.01563	1	0	0	0	0	0	0	1	1	0	0	0	0	0	0	0	0	0	NA	132	NA	60	6.84	6.85	7.05213	7.04087	10.3	8.6	3.5	3.2	50	25	136	102	21.0084	10.54852	9950	21600	8270	9050	NA	NA	NA	Class A
922	54	M	24.03486	1	0	0	0	0	1	0	0	1	0	0	0	0	1	1	0	0	0	128	138	88	83	1.99	1.71	29.08436	34.1523	10.5	12.5	4.4	4.2	NA	NA	NA	NA	NA	NA	NA	NA	NA	NA	0.80384	1.27993	6.1	Class A
923	81	M	22.64086	0	0	0	1	1	0	0	0	1	0	0	1	0	0	0	0	0	0	106	114	43	54	3.21	1.9	15.3444	27.23392	6.8	11.5	3.2	3.5	86	49	193	28.2	48.31461	17.81818	8470	303	648	580	0.45802	NA	7.3	Class A
924	78	F	26.894	0	0	1	0	1	0	0	1	1	0	0	0	0	1	1	0	0	0	144	138	80	64	1.66	1.76	23.58396	22.04126	9.6	10.4	4.3	4.2	91	86	64.8	59.4	30.23256	28.28947	219	246	496	222	0.15209	0.2507	7	Class A
925	62	M	28.57043	0	1	0	0	0	1	0	0	0	0	0	1	0	1	1	0	0	1	138	128	83	82	3.07	3.38	17.39626	15.65851	9.8	11.3	4	4	48	85	57.3	110	19.83471	33.73016	2730	3050	1560	940	1.44958	1.37271	6.4	Class B
926	62	M	NA	1	0	0	0	0	1	0	0	1	0	0	0	1	1	1	0	0	0	NA	120	NA	90	4.07	5.19	12.77877	9.79472	10.1	12.4	NA	3.8	70	114	112	83.7	27.23735	44.01544	458	602	323	613	2.78161	4.17367	NA	Class A
927	52	F	20.36958	0	0	1	0	0	1	0	1	0	0	0	0	0	0	0	0	0	0	137	136	93	103	2	1.9	21.60859	22.85582	9.4	11.7	4.6	4.5	91	86	196	88.2	37.91667	33.85827	452	842	102	124	0.65703	1.51915	NA	Class A
928	63	F	23.9334	0	0	1	0	1	0	0	1	0	0	0	0	0	1	1	0	0	1	140	140	82	80	3.87	3.76	9.93298	10.25132	9.5	10.5	4.3	4.2	87	105	50	148	29	37.63441	136	53	580	449	0.93059	0.83524	NA	Class B
929	47	M	20.67544	0	0	1	0	0	1	0	0	0	0	0	0	0	1	1	0	0	1	105	124	68	76	4.77	4.48	11.63083	12.3819	9.9	10.4	3.8	4.6	NA	137	NA	71.9	NA	37.74105	NA	194	NA	401	2.61702	2.2087	NA	Class A
930	82	M	20.87166	0	0	0	1	1	0	0	0	0	0	0	0	0	1	1	0	0	0	132	124	79	71	4.79	5.58	9.86848	8.35063	9.8	10.7	4.28	3.93	63	100	109	91.5	31.18812	46.2963	157	166	374	392	2.55	2.27885	NA	Class A
931	80	F	NA	0	0	1	0	1	0	0	0	0	0	0	0	0	1	1	0	0	0	NA	NA	NA	NA	2.52	2.65	14.82952	14.03552	9.1	10.4	3	2.7	37	17	140	37.2	14.5098	6.115108	13300	35500	730	944	1.1087	1.44262	NA	Class B
932	68	M	27.98616	0	1	0	0	0	1	0	1	0	0	0	0	0	0	0	0	0	0	145	132	84	77	4.08	4.28	12.41108	11.72877	8.9	11.6	4.1	3.6	91	64	279	109	32.15548	26.77824	218	1510	264	199	1.01905	0.98438	NA	Class A
933	70	M	23.88929	0	0	1	0	0	1	0	1	0	0	0	0	0	1	1	0	0	0	146	124	110	72	3.6	4.18	14.11444	11.98648	10.1	11.2	4.7	4.5	125	71	160	128	39.9361	27.20307	238	641	162	174	1.5421	0.85239	5.8	Class A
934	60	F	20.55694	0	0	1	0	1	0	0	0	0	0	0	1	0	0	0	0	0	0	135	149	72	86	2.5	2.71	16.2469	14.8747	10.5	10.4	4.1	3.9	97	81	56	44.2	40.41667	33.75	63	93	784	676	NA	0.68027	NA	Class A
935	58	M	32.86415	1	0	0	0	0	0	1	0	1	0	0	0	0	1	1	0	0	0	130	140	68	74	4.37	4.44	12.05067	11.84298	9.3	9.3	2.8	3.5	32	55	374	253	12.90323	20.99237	19900	536	926	343	NA	2.90244	6.7	Class B
936	73	F	NA	0	0	0	1	0	0	0	1	0	0	0	0	0	0	0	0	0	0	140	129	60	90	2.57	2.85	14.90067	13.2549	9.5	10.7	3.1	3.3	53	69	54.8	48.3	17.78523	21.97452	1280	353	426	289	8.20732	6.63855	NA	Class A
937	79	F	21.77844	1	0	0	0	0	0	0	0	1	0	0	0	0	0	0	0	0	0	120	NA	70	NA	3.31	3.87	11.04423	9.30832	8.9	9.4	2.7	3.6	60	52	96	49.2	22.22222	14.20765	4120	1740	3720	5030	6.48889	NA	NA	Class C
938	81	M	NA	0	1	0	0	0	1	0	1	0	0	0	0	0	1	0	1	0	1	148	160	81	86	2.44	2.7	20.71391	18.54191	10.3	11.3	3.4	3.4	90	76	68.6	116	41.09589	35.51402	1840	4840	322	256	2.17391	2.63158	5.4	Class A
939	88	M	20.13625	0	0	0	1	0	1	0	1	0	1	1	0	1	0	0	0	1	1	141	135	69	71	2.55	2.26	19.27437	21.99584	10.6	10.8	4	3.9	49	57	189	124	19.67871	24.46352	424	678	2250	6280	0.07064	0.16048	6.4	Class A
940	76	F	NA	1	0	0	0	1	0	0	0	1	0	0	0	0	1	1	0	0	1	128	147	41	56	1.86	1.45	20.98	27.54965	10.1	10.6	3.7	3.6	39	48	68.7	67.3	18.48341	17.26619	290	625	381	965	1.87983	2.30769	7.3	Class B
941	72	M	19.56017	0	1	0	0	0	1	0	0	0	0	0	1	0	1	0	1	0	0	118	129	63	72	4.8	5.07	10.22044	9.6265	10.1	11.3	4	4	87	103	106	117	28.33876	38.43284	193	830	943	962	1.84532	1.64341	5.2	Class A
942	70	F	19.29839	0	0	0	1	1	0	0	1	0	0	0	0	0	0	0	0	0	0	171	147	107	89	1.92	1.83	20.74777	21.77778	10.4	11.7	3.4	3.5	61	58	68.5	20.9	23.01887	19.39799	1790	1190	388	386	2.07692	1.80303	5.3	Class A
943	61	M	25.86451	1	0	0	0	1	0	0	0	1	0	0	0	0	1	1	0	0	0	138	195	86	97	2.61	2.74	20.87408	19.79306	10.7	11.8	4	3.8	98	92	162	99.9	35.63636	35.9375	63	50	169	120	4.9968	5.89207	6.4	Class A
944	68	F	25.10388	0	1	0	0	1	0	0	0	0	0	0	1	0	0	0	0	0	0	118	128	86	88	1.25	1.16	33.45774	36.30773	10.6	11.2	4.6	4.3	61	67	122	89	20.19868	24.36364	352	326	108	120	0.001389	1.49669	NA	Class A
945	67	M	19.26717	1	0	0	0	0	1	0	1	1	1	0	0	0	1	0	1	0	1	144	161	72	64	4.23	8.85	11.98124	5.34272	9.9	11.4	3.2	3.1	49	67	23.3	52.6	16.11842	23.92857	397	135	737	4690	7.95181	9.63141	7.4	Class C
946	88	F	26.44444	0	1	0	0	1	0	0	0	0	0	0	0	0	0	0	0	0	0	123	149	50	78	2.1	1.98	17.61456	18.78572	8.7	9.9	3.7	3.7	58	80	39.7	51.5	20.20906	28.16901	2540	657	394	245	0.14493	0.41494	NA	Class A
947	64	M	20.97656	0	0	0	1	0	1	0	0	0	0	0	0	0	1	1	0	0	1	114	137	73	80	4.85	4.51	10.45265	11.31772	9.4	11	4.2	4.3	75	101	175	156	26.69039	34.82759	395	516	1130	1350	0.59259	0.93373	5.2	Class A
948	80	F	33.15648	1	0	0	0	0	0	0	1	0	0	0	0	0	1	1	0	0	0	134	104	76	50	1.33	1.14	29.83772	35.31876	10.8	10.1	4.1	3.6	78	66	14	16.1	19.5	18.03279	211	361	67	112	0.2615	0.09015	6.6	Class A
949	77	M	27.43902	1	0	0	0	1	1	0	0	1	1	0	0	0	0	0	0	0	0	161	156	85	80	2.34	2.15	22.00173	24.13744	10.6	11.6	3.99	4.15	56	108	75	51	18.79195	34.72669	1080	1100	223	233	0.23664	0.40161	6	Class A
950	60	M	29.26838	0	0	1	0	1	0	0	0	0	0	0	0	0	1	1	1	0	1	127	129	82	87	2.96	NA	18.27592	NA	10.5	NA	3	NA	58	81	47.5	48.9	27.7512	36.16071	538	265	440	409	3.52113	NA	NA	Class A
951	89	M	22.31962	0	0	1	0	0	1	0	1	0	0	0	0	0	1	1	0	0	1	114	NA	65	NA	1.51	2.05	34.08207	24.39316	9	10.4	NA	2.97	85	49	28.4	129	28.52349	20	1950	16000	16100	13500	NA	NA	5.4	Class A
952	74	F	16.56005	0	0	1	0	1	0	0	0	0	0	0	0	0	1	0	1	0	0	121	146	67	79	2.58	3.09	14.77967	12.13283	8.8	10.9	4.1	3.7	66	126	126	92.8	33.84615	65.28497	50	50	1140	2430	4.89224	8.63971	NA	Class B
953	74	F	18.29817	0	1	0	0	1	0	0	0	0	1	0	0	0	1	0	1	0	0	139	124	80	74	1.43	1.12	28.18636	36.8241	9.9	11.8	3.3	3.7	34	98	393	269	15.52511	40.83333	5630	5310	1480	1470	0.26316	0.09901	6	Class A
954	65	F	19.11111	0	0	0	1	0	0	1	0	0	0	0	0	1	1	1	0	0	1	116	111	58	64	1.38	1.35	30.41672	31.15695	10.4	12.2	3.9	3.8	45	55	66.8	31	17.64706	20.2952	467	172	120	115	0.09009	0.11958	NA	Class A

955	59	M	21.27833	0	0	1	0	0	0	1	0	1	1	0	0	0	0	0	0	0	0	NA	NA	NA	NA	2.09	NA	26.87368	NA	9	NA	4.5	NA	131	NA	58	NA	32.26601	NA	94	NA	1260	NA	0.70732	NA	5.9	Class A
956	78	F	22.9381	0	1	0	0	1	0	0	0	0	0	0	0	1	1	1	0	0	1	110	122	50	74	1.07	1.13	38.13021	35.78981	10.2	10.3	3.7	4	60	42	17.2	9.6	16.26016	10.63291	2040	799	74	96	0.07718	0.04488	5.8	Class A
957	70	F	19.27056	0	0	1	0	1	0	0	0	0	0	0	1	0	1	1	0	1	1	136	122	55	60	1.44	1.5	28.42199	27.18061	7.7	7.3	4.3	4	67	57	41.7	43.4	20.87227	18.93688	81	162	750	739	0.19333	0.1773	NA	Class A
958	72	M	29.01207	1	0	0	0	0	1	0	1	1	0	0	0	0	1	1	0	0	1	150	125	52	46	2.67	3.12	19.41529	16.37352	11	11	3.4	4.1	62	81	105	12.5	25.40984	25.39185	3150	2320	1950	876	7.00312	2.18157	7.4	Class A
959	80	M	19.33373	0	0	0	1	0	1	0	1	0	0	0	0	0	1	1	0	0	0	160	190	84	97	1.84	1.73	28.30768	30.28255	8.4	10.2	3.7	3.4	68	101	157	150	27.86885	46.33028	659	850	2660	1410	1.4	1.4	NA	Class A
960	35	M	21.89279	0	1	0	0	1	0	0	0	0	0	0	0	0	1	1	1	0	0	107	136	63	90	3.93	4.28	15.64547	14.25131	7.7	11.3	4.1	4.5	60	73	292	167	24.19355	27.54717	52	137	423	334	0.36232	1.07066	NA	Class A
961	70	M	26.44564	0	0	0	1	0	1	0	0	0	0	0	0	0	1	1	0	0	1	126	114	66	65	2.83	2.62	18.36556	19.98191	10.7	14	4.3	4.4	59	76	140	64.1	24.38017	27.63636	763	980	383	251	0.23392	0.17442	NA	Class A
962	81	F	22.97194	0	0	0	1	1	0	0	0	0	0	0	0	0	0	0	0	0	0	NA	131	NA	70	1.61	1.7	24.12367	22.73002	9.5	11.3	3.8	4	89	90	45.7	34.9	28.61736	23.56021	50	50	1600	490	0.7	0.72	NA	Class A
963	70	M	24.13268	1	0	0	0	0	1	0	1	1	0	0	1	0	1	1	0	0	0	148	164	83	76	4.56	4.48	10.89811	11.11119	11.7	10.6	3.7	3.4	66	63	97.6	65	29.72973	29.03226	5240	1300	843	1470	4.12009	7.01583	6.9	Class A
964	84	M	NA	0	1	0	0	0	0	1	0	0	0	0	0	0	1	1	0	1	1	100	134	NA	71	2.02	2.59	25.20456	19.20364	8.8	9.7	3.9	4	73	94	117	160	32.88288	37.15415	11800	7680	1850	1410	NA	NA	NA	Class C
965	67	M	27.15341	0	0	0	1	0	1	0	1	0	0	0	0	0	1	1	0	1	1	141	137	89	93	3.39	3.64	15.2644	14.06134	10.2	12.2	3.4	3.6	54	65	99.3	126	25.71429	28.88889	1100	524	90	66	2.42524	2.69126	5.6	Class A
966	75	F	20.63038	0	0	0	1	1	0	0	0	0	0	0	0	0	1	1	0	0	0	119	133	77	77	2.39	2.19	16.00797	17.61399	9.9	11	4	4	83	96	336	291	35.77586	40	1450	1090	313	357	0.06562	0.21569	5.5	Class A
967	64	F	34.12607	1	0	0	0	1	0	0	1	1	0	0	0	0	0	0	0	0	0	177	153	87	61	1.9	2.6	21.53357	15.27889	8	9.1	2.8	2.1	33	44	208	205	17.36842	30.55556	8680	16100	3330	8270	12.62857	2.8169	6.1	Class A
968	69	F	NA	0	0	1	0	1	0	0	0	0	0	0	0	0	1	1	0	0	1	NA	113	NA	71	1.24	1.19	33.47339	35.01504	11	14	3.6	3.5	58	61	7.5	63.9	16.47727	24.01575	1850	1850	67	42	0.5	0.88571	NA	Class A
969	86	M	25.25721	0	0	0	1	0	1	0	1	1	0	0	0	1	1	1	0	0	1	142	117	62	56	2.57	2.99	19.23684	16.30108	10.3	12	3.8	3.5	62	43	73.5	29.6	27.92793	18.53448	2460	509	1140	355	0.73038	0.1722	7.3	Class A
970	57	M	24.74048	0	0	0	1	0	0	1	1	1	0	0	0	1	1	1	0	0	0	148	158	74	79	2.7	2.8	20.50943	19.70946	9.4	12.1	3.5	3.9	90	107	261	243	30.10033	37.41259	80	155	360	1420	1.5873	NA	5.9	Class A
971	59	F	23.82305	0	0	0	1	0	1	0	0	1	0	0	0	0	0	0	0	0	0	96	100	60	60	2.5	2.11	16.32545	19.55923	9.9	10.3	4	3.7	90	80	88.3	85.8	28.03738	26.31579	644	821	91	56	2.97976	3.26792	NA	Class A
972	54	M	20.60606	0	0	1	0	1	0	0	0	0	0	0	0	0	1	1	0	0	0	119	123	71	70	2.12	2.28	27.13897	25.06248	11.1	11	3.7	3.8	115	96	8.9	29.9	41.51625	38.4	4100	5170	131	65	4.17431	7.67635	NA	Class B
973	81	M	20.8949	0	0	0	1	0	1	0	1	0	0	0	0	0	1	1	0	0	0	184	145	88	75	3.32	3.6	14.78909	13.53542	9.5	12.3	3.6	3.3	75	142	294	260	31.12033	61.73913	294	358	1440	1330	5.35885	5.14729	5	Class C
974	76	F	24.08975	0	1	0	0	1	0	0	0	1	0	0	0	0	1	1	0	0	0	137	120	69	46	1.43	1.63	27.97145	24.23926	10.6	13.4	3.9	4	61	72	161	117	20.89041	20.74928	1120	2500	111	93	2.36154	2.00784	6.1	Class A
975	78	M	28.67188	0	1	0	0	0	1	0	1	0	0	1	0	0	1	1	0	0	0	144	139	66	80	3.7	5.22	13.27877	9.07928	8.5	9.3	3.4	3.3	76	61	114	96.4	29.80392	25.52301	493	282	3290	4660	3.84724	2.33111	5.1	Class A
976	67	F	32	1	0	0	0	1	0	0	1	1	0	0	1	0	1	1	0	0	0	167	152	70	57	1.54	1.71	26.7433	23.74753	10	10	3.9	3.4	85	58	44.1	35.1	28.52349	18.83117	814	392	530	530	13.13632	10.49173	5.7	Class A
977	85	F	22.53906	0	1	0	0	1	0	0	1	0	0	0	0	1	0	0	0	0	0	173	120	90	65	4.52	4.49	7.69098	7.74721	8	7.7	3.6	3.9	40	89	211	184	24.09639	45.64103	4170	957	10800	1960	0.80488	0.97714	NA	Class A
978	68	M	25.9302	1	0	0	0	1	0	0	1	1	0	0	0	0	1	0	1	0	0	141	NA	75	NA	2.62	NA	20.14884	NA	10.9	NA	3.9	NA	87	NA	182	NA	38.83929	NA	167	NA	412	NA	NA	NA	5.7	Class B
979	64	F	25.36952	0	0	0	1	0	0	0	0	0	0	0	0	0	1	1	0	0	0	135	134	86	77	3	3.31	13.06477	11.73223	9.4	11.6	3.5	3.7	51	73	91.6	63.5	22.17391	28.85375	575	607	74	85	1.55792	1.70819	NA	Class A
980	77	F	24.88889	0	1	0	0	1	0	0	1	1	0	0	0	0	1	1	1	0	0	141	147	57	65	1.72	1.54	22.76959	25.6966	9.7	10.8	4.5	4.5	98	94	253	245	35.2518	33.33333	101	115	212	171	0.08226	0.23284	NA	Class A
981	89	M	29.80469	1	0	0	0	0	1	0	0	1	0	0	0	0	0	0	0	0	0	131	104	57	49	1.23	1.41	42.65506	36.73509	11.4	12.5	4.4	4.6	49	64	204	90.4	17.25352	19.21922	2160	1070	361	137	0.07576	0.03745	6.9	Class A
982	76	F	23.40192	0	1	0	0	1	0	0	0	0	0	0	0	0	1	1	0	0	1	118	111	64	71	2.15	2.42	17.90462	15.73108	9.3	10.1	3.7	3.9	80	51	45.2	68.9	29.85075	17.89474	758	291	279	235	1.97965	2.1624	6.1	Class A
983	63	F	18.50796	0	1	0	0	1	0	0	0	0	0	0	0	0	1	1	0	0	1	121	138	70	76	2.73	2.25	14.55035	17.97826	10.8	10.6	4.1	3.8	36	62	31.6	12.1	11.25	17.71429	967	578	341	469	1.13889	1.32203	5.3	Class A
984	86	M	NA	0	1	0	0	1	0	0	1	0	0	0	0	0	1	1	0	0	0	189	151	103	71	1.85	1.92	27.56226	26.46483	9	10	3.7	3.9	67	94	41.1	36.5	30.87558	35.74144	367	483	197	155	0.3178	NA	NA	Class A
985	69	F	22.69065	0	0	0	1	1	0	0	1	1	0	0	0	0	1	1	0	0	0	140	122	62	60	1.96	1.68	20.36894	24.11061	10.2	11.4	4.1	3.8	59	84	61.9	43.4	18.61199	27.45098	286	778	56	100	1.075	2.50455	7	Class A
986	65	F	20.16496	0	0	1	0	1	0	0	0	0	0	0	0	0	1	1	0	0	0	133	125	79	71	4.17	4.81	9.07217	7.76021	11	11.4	3.5	3.6	122	93	43.7	24.6	33.42466	25.47945	50	103	114	152	4.13823	18.95	NA	Class B
987	74	F	19.26589	0	0	1	0	1	0	0	0	0	0	0	0	0	1	1	0	0	0	126	109	63	52	1.96	2.33	19.88728	16.45948	10.6	9.8	4.1	3.6	NA	76	NA	116	NA	26.57343	NA	615	NA	264	0.79032	2.34167	NA	Class A
988	80	F	20.50355	0	1	0	0	0	1	0	0	0	0	0	0	0	1	0	1	1	1	118	140	69	80	1.4	1.34	28.20949	29.59421	11.1	11.3	3.6	3.5	89	120	65.3	80.8	38.52814	58.53659	7720	6760	2050	3190	0.04032	0.10345	5.5	Class B
989	48	M	25.58845	0	0	1	0	1	0	0	0	0	0	0	0	0	0	0	0	0	0	96	101	55	43	6.18	7.82	8.70851	6.69187	10.7	10.4	4.7	4.1	82	107	179	92.5	31.53846	43.49593	1000	332	206	668	1.45902	1.11628	5	Class C
990	80	M	22.23201	0	0	1	0	0	1	0	0	0	0	0	0	0	1	0	1	0	0	132	128	64	64	2.56	2.24	19.72424	22.82672	10.2	11.7	3.9	3.5	51	47	184	74.4	20.73171	18.65079	733	9810	559	445	1.17284	1.64013	5.6	Class B
991	70	F	22.82688	0	1	0	0	1	0	0	1	0	0	0	0	1	0	0	0	1	1	141	127	67	68	3.25	2.51	11.66548	15.47604	10.2	12.1	3.5	3.7	58	39	31.5	53.8	19.52862	14.77273	268	496	1310	1340	2.18462	0.35484	NA	Class A
992	73	M	20.87624	0	1	0	0	0	1	0	1	0	0	0	0	0	1	1	0	0	1	148	139	82	75	2.45	3	21.24616	17.02384	9.7	10.9	2.7	3.2	49	81	52.7	105	16.61017	29.56204	1420	97	817	790	3.93743	3.81897	5.9	Class B
993	60	F	19.23438	0	0	1	0	1	0	0	1	0	0	0	0	0	1	0	1	0	0	160	155	87	80	1.82	1.92	22.99316	21.68629	10.3	11.5	3.7	3.8	71	68	59.9	37.8	25.53957	21.65605	115	50	682	444	0.7027	0.52632	NA	Class B
994	74	M	22.49135	1	0	0	0	0	0	1	0	1	0	0	0	0	1	1	0	0	0	135	147	40	64	2.3	2.01	22.67785	26.28063	10.1	11.7	4.1	4.3	54	69	361	279	20.14925	25.46125	4640	428	343	131	0.03933	0.05222	6.2	Class A
995	78	F	18.85328	0	0	1	0	1	0	0	1	1	0	0	0	0	0	0	0	0	1	146	152	68	83	1.07	1.13	37.99105	35.78981	9.2	12.3	3.4	3.5	54	65	243	44.6	20.07435	19.34524	1610	1340	328	190	0.38363	0.1061	6.6	Class A
996	65	M	25.92251	0	0	0	1	0	1	0	0	0	0	0	0	0	1	1	0	1	1	126	NA	78	NA	4.97	6.45	10.08739	7.58463	6.9	12.1	2.5	3.5	21	86	13.6	117	7.636364	32.69962	9850	6390	2770	4360	0.93809	12.2549	NA	Class B
997	72	F	19.2637	0	1	0	0	1	0	0	1	0	0	0	0	0	0	0	0	1	1	157	136	77	68	2.44	2.67	15.83388	14.29122	10.9	11.7	4.3	4.3	84	119	38.1	57.1	28.28283	38.76221	234	117	363	228	0.17725	0.15726	5.3	Class A
998	78	M	22.57075	0	0	1	0	0	1	0	0	0	0	0	1	0	0	0	0	0	0	130	130	84	84	4.07	3.92	11.96394	12.46567	9.7	11	3.8	3.5	100	109	103	70.7	42.73504	44.30894	450	313	1610	1770	0.62713	0.81374	NA	Class A
999	43	M	24.32251	0	0	0	1	1	0	0	0	0	0	0	0	0	1	0	1	0	0	121	141	77	92	4.85	4.39	11.71642	13.06592	10.1	11.1	4	4	86	96	188	134	35.2459	38.24701	979	1900	78	72	1.41031	1.18839	NA	Class B
1000	54	F	18.4808	0	0	0	1	0	1	0	0	0	0	0	0	0	1	1	0	0	0	131	129	76	80	2.04	1.76	20.91769	24.58433	9.7	10.7	4.2	4.1	84	101	22.2	19.9	27.54098	33.22368	155	176	220	187	0.41463	1.27536	NA	Class A
1001	41	M	28.28753	1	0	0	0	1	0	0	0	1	0	0	0	0	1	1	0	0	0	122	NA	74	NA	5.15	7.63	11.12284	7.18534	10.7	14.2	3.4	3	70	108	287	132	23.72881	37.76224	918	188	344	686	7.43827	9.4434	6.4	Class C
1002	69	M	21.0812	1	0	0	0	0	1	0	0	1	0	0	0	0	1	1	0	1	1	123	104	59	76	5.63	11.41	8.68952	4.01219	10	10.8	3.5	2.8	67	35	65.5	115	24.27536	12.41135	135	9360	16100	98600	1.30955	NA	6.3	Class B
1003	65	F	17.36111	0	1	0	0	1	0	0	0	0	0	0	0	0	0	0	0	0	0	NA	NA	NA	NA	3.91	4.64	9.73416	8.07178	10.3	11.4	4.5	4.3	56	115	34.5	15.9	18.24104	32.76353	50	50	480	334	1.56863	1.40351	NA	Class A
1004	65	F	26.78252	1	0	0	0	1	0	0	1	1	0	0	0	0	1	1	0	0	0	166	133	63	59	1.05	NA	41.01653	NA	9.7	NA	3.6	NA	60	65	183	179	18.86792	22.10884	148	290	123	204	0.83333	NA	7.5	Class A
1005	90	F	18.49108	0	1	0	0	1	0	0	1	0	0	0	0	0	0	0	0	0	1	145	117	76	69	1.1	1	35.50545	39.40747	10	12.7	3.3	3.9	33	103	12	56.2	11.53846	36.14035	232	63	76	82	0.13468	0.15238	NA	Class A
1006	63	M	25.11226	1	0	0	0	0	1	0	0	1	0	0	0	0	1	1	0	0	0	128	162	70	90	5.51	6.62	9.13211	7.47089	11.1	11.1	3.7	3.3	94	NA	127	NA	35.20599	NA	325	NA	456	NA	4.94783	8.71875	6.1	Class A
1007	78	M	NA	0	0	1	0	0	0	0	0	0	0	0	0	0	0	0	0	0	0	127	125	45	70	1.79	1.48	29.3866	36.18297	10.2	10.9	3.7	3.5	108	67	111	70.3	41.06464	30.45455	1630	978	75	78	NA	0.27222	NA	Class A
1008	73	M	28.39458	0	1	0	0	1	0	0	0	0	0	0	0	0	1	1	0	0	0	136	138	73	71	2.83	3.04	18.1457	16.77893	9.9	10.1	4.3	4	64	70	98.4	26.5	23.7037	23.10231	1500	2270	43	34	1.26316	0.78889	NA	Class A
1009	49	M	17.92388	0	0	1	0	0	0	1	0	1	1	0	0	0	0	0	0	0	0	133	137	81	89	3.09	3.63	18.47995	15.4945	10.4	13.1	4.4	4.5	107	115	186	129	31.19534	30.58511	123	110	493	364	2.61538	3.33684	5.5	Class A
1010	50	F	31.99591	0	0	1	0	1	0	0	0	0	0	0	0	0	0	0	0	0	0	110	126	52	52	3.4	2.67	12.22936	15.93083	8.4	10.2	4.3	4.5	41	NA	94.4	NA	15.58935	NA	1020	NA	286	NA	1.81429	3.55	NA	Class B
1011	79	M	22.77319	1	0	0	0	0	1	0	1	1	0	0	0	0	0	0	0	0	0	151	151	75	64	3.63	5.27	13.50968	8.98508	10.2	10.7	2.7	3.1	65	46	129	117	31.70732	22.11538	3410	8660	443	847	10.42553	6.40835	6.8	Class C
1012	81	M	22.6034	1	0	0	0	0	1	0	0	1	0	0	0	0	1	1	0	0	0	139	149	63	66	2.8	3.18	17.81868	15.50284	9	10.5	3.7	3.2	78	51	250	221	30.95238	22.07792	946	20200	1210	4720	3.26744	3.21795	6	Class A
1013	79	F	21.67746	0	1	0	0	1	0	0	0	0	0	0	0	0	1	1	0	0	0	125	132	59	58	1.43	1.45	27.66238	27.14706	10.7	12.9	3.64	4.03	77	61	151	90.7	32.62712	22.26277	465	428	291	142	0.13855	0.5	NA	Class A
1014	84	F	27.00617	1	0	0	0	1	0	0	1	1	0	0	0	1	0	0	0	0	1	144	136	58	63	2.14	3.08	17.4866	11.74096	8.2	10.3	3.5	3.8	58	26	60.7	86.6	20.13889	10.19608	2820	819	4610	4690	1.29964	0.92424	6.8	Class A
1015	88	F	22.08889	0	0	1	0	1	0	0	0	1	0	0	0	0	1	1	0	0	1	128	137	50	65	0.88	0.9	45.61583	44.50803	10.2	13.4	4.1	4.1	42	100	11.4	60.7	9.545455	29.41176	676	482	772	620	0.35492	0.30275	8.6	Class A
1016	67	F	18.06535	0	1	0	0	0	1	0	0	0	0	0	0	0	0	0	0	1	1	120	132	57	71	3.1	2.8	12.43979	13.90503	8.7	11.4	4.4	3.9	83	63	110	39.6	27.39274	24.23077	178	124	351	264	1.91882	5.53903	NA	Class B
1017	73	F	29.87392	1	0	0	0	0	1	0	0	1	0	0	0	1	0	0	0	1	1	137	152	55	54	4.11	3.72	8.91517	9.94257	9.6	10.3	4.6	4.5	52	56	181	152	17.27575	20.89552	224	216	967	971	2.30769	2.28	5.3	Class A
1018	79	F	19.61672	0	0	0	1	1	0	0	0	0	0	0	0	0	1	1	0	0	0	122	133	54	56	1.5	1.48	26.25327	26.64164	9.9	10.3	3.1	3.7	47	83	226	165	21.75926	35.0211	20800	3370	764	540	0.34672	0.71429	5.1	Class A
1019	68	M	19.14702	1	0	0	0	0	0	1	1	1	0	0	0	0	1	1	0	0	0	141	140	61	57	3.59	3.99	14.27573	12.71766	10.3	10.1	3.7	3.8	86	78	39.5	18.3	21.85185	26.44068	802	474	6190	11500	2.6062	2.0903	6.3	Class A
1020	77	F	19.86397	0	0	0	1	1	0	0	1	0	0	0	0	0	1	1	0	0	0	146	127	74	82	1.53	1.54	25.8804	25.60161	9.8	11.3	3.6	3.7	52	60	85.2	45.2	21.66667	24.19355	1500	873	4580	2620	1.74954	NA	NA	Class A
1021	78	F	26.4	1	0	0	0	1	0	0	0	1	0	0	0	0	0	0	0	0	0	125	121	84	81	1.37	1.17	29.09663	34.45338	10.1	11	3.7	3.7	92	67	259	450	29.29936	24.72325	260	500	149	172	0.97647	0.71875	7.4	Class A
1022	81	M	28.09776	0	1	0	0	0	0	1	0	0	0	0	0	0	0	0	0	0	0	136	130	80	78	2.95	4.34	16.82989	11.03196	9	10.6	3.6	3.2	95	94	146	182	38.93443	38.05668	445	387	358	531	3.10638	7.81481	5.2	Class C
1023	82	M	22.23099	0	1	0	0	1	0	0	0	0	0	0	0	1	1	1	0	0	1	116	112	68	64	3.95	4.18	12.18597	11.45435	9.9	10.3	3.8	3.6	90	88	207	196	37.3444	34.92063	3090	1930	1990	1410	0.14706	0.09	NA	Class B
1024	67	M	20.72757	0	1	0	0	0	1	0	0	0	0	0	0	0	0	0	0	0	0	NA	NA	NA	NA	1.09	1.04	52.5929	55.36524	10.7	10.4	4.4	4.1	35	31	4.9	5.4	8.373206	7.451923	865	895	246	272	0.12346	0.32967	NA	Class A
1025	68	F	27.88762	1	0	0	0	0	1	0	0	1	0	0	0	0	1	1	0	1	1	128	132	58	68	3.1	3.2	12.38701	11.96416	10	11.8	3.9	3.7	60	46	152	96.2	24.19355	19.40928	117	220	162	252	2.24756	3.38462	7.7	Class A
1026	69	F	25.39063	0	1	0	0	0	0	1	0	0	0	0	0	0	0	0	0	0	0	118	124	68	98	1.95	1.6	20.48324	25.43251	9.8	10.7	4.5	4.5	94	NA	85	NA	31.54362	NA	194	NA	141	NA	0.35366	0.51908	5.4	Class A
1027	60	M	41.26623	0	0	1	0	0	1	0	0	1	0	0	0	0	1	1	0	0	0	130	145	84	82	5.89	6.43	8.60927	7.82149	10.6	11.1	3.7	3.9	71	71	116	125	27.73438	25.81818	914	1030	682	966	2.09559	2.43056	5.8	Class A
1028	62	M	28.04038	1	0	0	0	1	0	0	0	1	1	1	0	1	1	1	1	0	1	108	118	55	54	4.57	5.51	11.25737	9.17414	10.9	11.5	3.7	3.5	67	65	38.1	72.1	26.17188	30.09259	484	1640	705	638	3.83693	6.77596	6	Class C
1029	71	M	21.4908	1	0	0	0	0	1	0	0	1	0	0	0	0	1	1	0	0	0	114	160	66	80	3.81	4.91	13.2117	10.01031	10.4	12.1	3.3	2.4	67	68	75.5	54.7	26.5873	28.57143	158	140	1330	2590	7.19398	13.24082	5.7	Class A
1030	71	M	24.22145	0	0	0	1	0	1	0	1	0	0	0	0	0	1	1	0	0	0	140	140	70	80	5.86	5.83	8.24918	8.29563	10.6	10.7	4.5	4.3	65	79	97.1	65.9	19.63746	24.23313	1780	166	232	318	1.12703	2.00722	5.4	Class A
1031	59	M	21.18102	1	0	0	0	0	0	1	0	1	0	0	0	0	1	0	1	0	0	133	102	64	61	2.33	2.77	23.86052	19.65167	10.4	13.9	4.1	3.9	79	73	303	178	31.10236	27.34082	103	68	236	68	3.11615	2.55491	6.3	Class A
1032	79	M	24.81265	0	0	0	1	0	1	0	1	0	0	0	0	0	1	1	0	0	0	172	151	92	80	2.86	2.89	17.53551	17.33646	10.6	12	2.68	3.07	114	100	152	139	54.28571	41.49378	1720	1290	3000	625	NA	NA	NA	Class A
1033	87	F	20.64702	0	1	0	0	0	0	0	0	0	0	1	0	0	0	0	0	0	0	134	136	72	70	1.51	1.87	25.35148	20.06363	9.4	9.7	3.4	3.5	63	55	91.4	65.6	21.07023	17.08075	4900	3820	2420	1400	7.15457	3.25221	NA	Class B
1034	75	F	28.64583	0	1	0	0	1	0	0	0	1	0	0	0	0	1	1	0	0	0	133	151	52	48	2.51	2.73	15.17262	13.78767	9.5	10.4	4	4.1	60	56	79.9	48.3	23.62205	20.81784	388	304	1010	655	0.40643	0.38122	5.9	Class A
1035	88	M	23.84472	0	0	1	0	0	1	0	1	0	0	0	0	1	0	0	0	0	0	164	151	81	80	2.64	2.67	18.55669	18.32871	10.6	11.5	3.7	4	20	69	214	172	8.695652	30.53097	11100	330	976	1210	1.64706	1.27976	NA	Class A
1036	66	F	20.35219	0	1	0	0	1	0	0	1	0	0	0	0	0	1	1	0	0	0	142	149	88	81	1.86	1.89	21.8469	21.37536	10.5	12.6	4.1	4.2	70	80	79.5	39.4	34.14634	34.63203	15700	14900	332	639	1.02703	1.34752	6.3	Class A
1037	89	M	15.47565	0	0	0	1	0	0	0	0	0	0	0	0	0	0	0	0	0	0	110	110	55	50	1.62	1.66	31.55857	30.72759	10.4	10.7	4	4	86	80	108	103	33.07692	31.1284	670	1170	546	503	0.11215	0.10687	NA	Class A
1038	86	M	23.39209	0	1	0	0	0	1	0	0	0	0	0	0	0	0	0	0	0	0	103	117	63	66	3	NA	16.24165	NA	9.2	NA	4.1	NA	92	73	328	214	39.48498	29.08367	1240	4180	1340	1360	0.41944	NA	6.3	Class A
1039	76	M	23.14726	0	0	0	1	0	1	0	1	0	1	0	0	0	0	0	0	0	0	151	142	71	67	2.62	3.01	19.51581	16.76704	10.4	11.4	3.8	3.8	69	74	267	248	30.80357	33.78995	617	816	2680	1900	1.44056	3.53923	NA	Class B
1040	67	M	25.6367	0	1	0	0	0	1	0	1	0	0	0	1	0	1	1	0	0	1	165	112	85	49	2.03	1.79	26.74962	30.69708	8.6	10.8	2	3.6	60	158	38.4	18.5	34.28571	57.03971	368	95	450	181	4.15051	1.36237	5.3	Class A
1041	87	F	24.48531	0	1	0	0	1	0	0	0	0	0	0	0	0	0	0	0	0	0	112	168	48	48	2.46	1.67	14.8635	22.70661	9	10.1	3.9	3.6	47	62	64.1	37.3	21.46119	26.16034	168	182	321	887	0.20282	0.55541	NA	Class A
1042	76	M	20.18519	1	0	0	0	0	1	0	1	1	1	1	0	1	1	1	0	0	0	164	158	81	74	3.49	4.01	14.20783	12.20503	10.3	12.6	3.9	3.9	74	93	137	74.3	30.70539	34.57249	220	183	836	834	2.59947	3.87941	5.9	Class A
1043	72	M	19.60716	0	1	0	0	0	1	0	0	0	0	0	0	0	1	0	1	0	0	110	117	71	60	2.61	2.12	19.9041	24.98825	10.2	12	4.4	4.5	60	85	358	300	20.40816	27.6873	641	322	1400	1330	0.12853	0.001852	5.5	Class A
1044	63	M	20.74658	1	0	0	0	0	1	0	0	1	1	0	0	1	1	1	0	0	0	108	196	58	89	3.73	3.59	13.99399	14.5921	9.5	10.5	4.3	4.2	66	104	183	126	23.15789	36.36364	327	148	1320	2430	NA	3.94524	7.1	Class A
1045	57	F	19.27894	0	0	1	0	1	0	0	0	0	0	0	0	0	1	1	0	0	0	100	126	60	80	3.05	2.75	13.26435	14.85526	10.9	11.2	4.8	4.6	70	78	58.6	30.1	19.66292	21.25341	246	141	151	254	0.20833	NA	NA	Class A
1046	64	F	23.70791	0	0	0	1	1	0	0	0	1	1	1	0	1	1	0	1	0	1	133	138	53	65	0.94	0.81	46.50189	54.48255	9.9	12.6	3.8	4	40	69	6.4	24.8	9.111617	17.33668	50	50	376	303	0.19704	0.78431	6.7	Class A
1047	76	M	24.33748	0	0	0	1	0	1	0	1	0	0	0	1	0	1	1	0	0	0	140	137	72	62	1.81	1.86	29.24879	28.28341	11.3	11.6	4.3	4.3	56	64	331	308	24.77876	26.01626	1580	927	203	862	0.08411	0.26973	5.5	Class A
1048	81	F	22.95918	0	0	0	1	1	0	0	0	1	0	1	0	0	0	0	0	0	0	116	NA	70	NA	2.01	1.91	18.92407	19.94027	10.8	11.4	4.5	4.4	96	116	150	141	28.40237	33.91813	67	50	475	579	0.001724	0.000735	NA	Class A
1049	72	F	34.66667	0	1	0	0	0	1	0	1	1	0	0	0	0	1	1	0	0	0	148	160	80	102	2.67	2.76	14.3479	13.78218	10.4	10.8	4.2	4.2	37	46	184	151	14.34109	17.35849	3150	3160	411	339	2.48214	2.09524	5.5	Class A
1050	34	M	27.07363	1	0	0	0	0	0	1	0	1	0	1	0	0	0	0	0	0	0	127	102	77	66	2.73	3.47	23.50203	18.07785	11.2	10.9	2.7	2.7	60	43	150	175	24.79339	16.79688	5150	3930	2310	2980	5.40426	NA	5	Class B
1051	47	F	26.90868	1	0	0	0	0	1	0	0	1	0	1	0	0	1	1	0	1	1	125	136	66	83	2.11	2.46	20.97921	17.62978	10	12.3	2.8	2.6	121	137	66.3	29.4	46.89922	55.69106	50	177	338	780	11.41778	14.46825	8.7	Class A
1052	49	F	16.39958	0	0	1	0	1	0	0	1	0	0	0	0	0	0	0	0	0	0	142	NA	99	NA	2.77	2.9	15.39172	14.6385	8.6	10.4	3.4	3.5	76	55	65	53.6	33.48018	20.75472	2140	248	324	358	1.28866	2.40566	5.2	Class A
1053	79	M	26.6436	0	0	1	0	0	1	0	0	1	0	0	0	0	0	0	0	1	1	121	98	66	56	3.63	3.22	13.50968	15.4024	9.3	10	3.4	3.1	78	51	93.6	51.3	28.78229	19.31818	265	95	532	638	2.77654	2.15517	6.5	Class A
1054	64	M	21.45329	0	0	1	0	0	0	1	1	0	0	0	0	0	1	1	0	0	0	146	127	83	74	3.02	3.3	17.55094	15.92846	10.3	12.3	3.8	3.7	99	134	235	166	39.6	52.96443	314	118	281	134	0.29749	0.72884	NA	Class A
1055	56	F	22.59349	0	0	1	0	0	0	1	0	0	0	0	0	0	0	0	0	1	1	115	125	85	85	3.77	3.99	10.57301	9.93692	10.2	11.1	4.9	4.5	86	69	106	93.7	33.59375	28.63071	238	144	207	170	0.64553	0.47857	NA	Class A
1056	76	M	19.57759	0	1	0	0	0	1	0	1	0	1	0	0	0	1	1	0	0	0	145	151	71	91	4.44	4.36	10.95901	11.17918	8.6	11.4	3.4	4.5	45	83	40.2	46	17.11027	23.2493	536	1810	1030	506	0.93361	1.20209	5.4	Class B
1057	67	F	NA	0	0	1	0	1	0	0	0	0	0	0	1	0	0	0	0	0	1	115	136	67	81	2.34	2.28	16.92157	17.40933	9.3	9.1	NA	3.3	NA	71	NA	39	NA	23.98649	NA	3210	NA	3190	NA	0.18421	NA	Class A
1058	62	F	28.88889	0	0	0	1	1	0	0	0	0	0	0	0	0	1	1	0	0	0	117	136	69	77	2.86	2.65	13.89202	15.10076	10.1	10.6	3.8	3.5	59	86	50.6	24.9	21.69118	28.28947	537	392	343	454	1.04167	2.60377	NA	Class A
1059	74	M	22.26969	0	1	0	0	1	0	0	0	1	1	1	1	1	1	1	0	0	1	108	116	57	56	1.46	1.64	37.28466	32.83168	9.7	11.8	3.8	4	38	102	11.7	20.2	8.941176	27.0557	513	171	706	1470	0.07236	0.06547	6.1	Class A
1060	84	M	17.48555	0	0	0	1	1	0	0	0	0	0	0	0	0	1	1	1	0	0	128	140	68	66	3.54	4.35	13.64344	10.88996	9.5	9	3.3	3.1	63	42	40.3	24.1	22.58065	14.78873	540	4590	549	1180	3.6165	5.26542	NA	Class A
1061	62	M	27.3044	1	0	0	0	0	1	0	1	1	0	0	0	0	0	0	0	0	0	207	138	94	70	4	4.65	13.02362	11.04566	10	10.4	4.4	4	65	57	174	73.6	23.63636	21.83908	163	96	210	243	3.5127	2.36602	6.1	Class B
1062	57	M	21.71492	1	0	0	0	0	0	1	1	1	1	0	0	0	0	0	0	0	0	140	131	84	78	2.62	2.91	21.19552	18.89586	10	10.6	3.9	3.7	65	56	185	138	23.63636	22.48996	169	406	729	834	1.50855	2.08752	5.8	Class A
1063	90	M	23.4365	0	1	0	0	0	1	0	0	0	0	0	0	0	0	0	0	0	0	125	122	85	53	3.65	3.24	12.93557	14.73662	8.5	11	3.6	3.8	81	82	234	193	40.29851	44.08602	368	407	3410	3180	0.13139	NA	NA	Class A
1064	75	M	27.07214	0	0	1	0	0	1	0	1	0	0	0	0	0	1	0	1	1	1	147	167	66	77	4.84	4.38	10.0101	11.12335	8.8	10.1	3.5	3.6	79	112	30.8	59	27.8169	38.35616	574	1200	298	300	2.57056	2.46345	NA	Class C
1065	50	F	17.39585	0	0	0	1	0	0	1	1	0	0	0	0	0	0	0	0	0	0	168	131	99	89	1.95	2.39	22.46694	17.98351	9.9	10.2	3.1	3.5	26	23	21.3	20.8	10	8.333333	18400	9730	612	693	3.24742	2.5578	5.5	Class A
1066	89	F	27.39966	0	0	1	0	1	0	0	1	0	0	0	0	0	0	0	0	0	1	192	189	99	95	1.16	1.18	33.60889	32.9862	10.7	11.7	4.7	4.5	86	99	57.9	77.8	31.85185	41.42259	487	1010	237	313	1.67442	0.86207	NA	Class A
1067	63	F	21.64412	0	0	0	1	1	0	0	0	0	0	0	0	0	1	1	0	0	0	120	120	80	80	1.96	2.12	20.90775	19.18775	10.5	10.9	3.9	3.9	76	72	49.7	53.1	27.63636	27.48092	374	401	96	112	0.54237	0.27857	NA	Class A
1068	73	M	24.48118	1	0	0	0	1	0	0	1	1	1	0	0	0	1	1	0	0	0	140	NA	64	NA	3.96	NA	12.56463	NA	10.4	NA	3.3	NA	41	NA	363	NA	19.24883	NA	1320	NA	393	NA	2.21205	NA	NA	Class B
1069	78	M	22.08398	1	0	0	0	1	0	0	1	1	0	0	0	0	1	1	0	0	0	145	132	71	65	3.77	4.07	13.00928	11.96394	10.1	12.6	3.8	3.7	89	72	122	62	36.92946	26.47059	275	209	1080	603	1.25	1.20643	NA	Class A
1070	36	F	32.48775	1	0	0	0	0	1	0	0	1	0	0	0	1	1	1	0	1	1	106	129	61	77	1.9	1.62	25.39984	30.00287	9.7	11.7	2.6	2.2	48	55	65.6	78.9	22.42991	31.6092	1810	649	255	297	8.77143	12.62143	7.8	Class B
1071	83	M	23.99455	0	0	1	0	0	1	0	0	0	0	0	0	0	1	1	0	0	1	132	144	68	60	2.32	2.08	21.73614	24.4943	9.4	9.7	3.9	3.6	52	NA	61.3	NA	21.66667	NA	202	NA	725	NA	1.70883	1.27156	5.6	Class A
1072	79	F	23.96279	0	1	0	0	1	0	0	1	0	0	0	0	0	1	0	1	0	0	159	146	65	99	1.81	1.77	21.29899	21.82612	10.9	10.3	4.34	4.02	112	118	25.6	22.8	35.33123	38.06452	122	108	266	423	0.10711	0.26975	NA	Class A
1073	89	F	27.88398	0	0	1	0	1	0	0	1	1	1	0	0	0	0	0	0	0	0	145	143	63	60	1.12	1.14	34.92422	34.14481	10.5	11.1	3.2	3.6	28	62	34.4	9.1	9.180328	15.15892	42100	882	310	217	0.09615	0.07527	5.9	Class A
1074	80	M	26.77747	1	0	0	0	0	1	0	0	1	0	0	0	0	0	0	0	0	0	135	149	71	84	2.81	3.04	17.81272	16.34373	9.9	10.5	4.2	4.3	62	76	152	143	25.83333	30.89431	1250	751	4220	4450	0.90909	2.43939	6.1	Class A
1075	64	M	NA	1	0	0	0	0	1	0	0	1	0	1	0	0	1	1	0	0	0	126	142	76	83	1.6	1.62	35.16583	34.69115	9.3	11.1	2	2.5	61	61	125	93.3	33.88889	29.32692	708	153	952	683	8.07317	8.15	5.3	Class A
1076	85	M	27.17456	0	1	0	0	0	1	0	0	0	1	0	0	0	1	0	1	0	0	119	121	74	73	3.22	3.47	15.08218	13.89754	10.5	11.2	3.9	3.9	91	82	77	45.8	33.57934	28.3737	340	128	978	969	2.0159	2.13957	6.4	Class A
1077	67	M	21.62039	1	0	0	0	0	1	0	0	1	0	1	0	0	1	0	1	0	0	110	141	61	76	2.36	2.33	22.68572	23.00546	9.4	12.2	3.8	3.4	69	NA	266	NA	25.55556	NA	328	NA	267	NA	1.23514	3.24167	6.1	Class A
1078	77	F	31.49298	0	0	1	0	1	0	0	0	0	0	0	0	0	1	0	1	0	0	123	119	80	79	2.14	2.25	17.92878	16.9721	11	12.1	3.6	3.3	69	36	92.3	43.6	28.63071	12.99639	336	731	337	246	1.53563	1.54472	5.4	Class A
1079	80	M	NA	1	0	0	0	1	0	0	1	1	0	0	1	0	1	1	0	0	0	146	143	54	53	3.32	3.56	14.84191	13.70188	9.2	10.9	NA	3	71	89	288	227	31.83857	40.82569	997	511	2300	1780	NA	3.7	NA	Class A
1080	74	F	28.01022	0	0	1	0	1	0	0	0	1	0	0	0	0	1	1	0	0	0	133	125	56	53	3.24	3.45	11.51968	10.75481	9	9.9	3.9	4.3	41	NA	84.9	NA	16.94215	NA	6470	NA	937	NA	1.33727	0.80416	7.2	Class B
1081	54	F	19.7777	0	1	0	0	1	0	0	0	0	0	0	1	0	1	1	0	0	0	124	129	76	82	1.75	1.67	24.73805	25.90063	9.7	9.8	4.3	4.5	76	91	308	337	26.48084	33.95522	1090	348	32	107	1.86878	2.51256	NA	Class A
1082	74	M	26.11047	0	0	0	1	0	1	0	0	0	0	0	0	0	1	1	0	0	0	122	148	66	68	2.68	2.94	19.1846	17.26979	10	11.4	3.5	3.9	62	84	250	225	29.95169	36.84211	5800	424	472	412	2.4	3.32692	5.6	Class A
1083	64	F	21.77844	0	0	1	0	1	0	0	1	1	0	0	0	0	1	1	0	0	1	164	158	66	68	2.4	2.64	16.67713	14.9591	9.7	11.3	4.4	4.4	52	65	61.9	80	19.04762	23.55072	273	483	139	129	0.6383	1	NA	Class A
1084	66	M	22.26578	0	0	0	1	0	0	1	0	0	0	0	0	0	1	1	0	0	0	113	126	60	74	1.74	1.89	31.80034	29.04982	10.4	13.3	2.5	2.5	59	77	105	80.2	32.41758	34.0708	2050	4330	475	375	9.94468	12.23158	NA	Class A
1085	65	M	NA	0	0	1	0	0	1	0	0	0	0	0	0	0	0	0	0	0	0	NA	NA	NA	NA	1.75	2.06	31.74037	26.55369	10.5	12.5	3.3	3.1	109	116	221	54.1	34.16928	37.0607	415	1990	320	252	2.79798	3.85124	5.4	Class B
1086	82	M	23.61296	1	0	0	0	0	1	0	0	1	0	0	0	0	1	1	0	0	0	126	NA	46	NA	1.74	NA	29.8797	NA	8.9	NA	3.2	NA	52	71	56	52.8	19.77186	22.11838	197	961	403	605	0.68889	NA	6.9	Class A
1087	71	M	21.25285	0	0	0	1	0	1	0	1	0	0	0	1	0	1	0	1	0	0	170	166	90	85	3.19	3.7	16.04514	13.642	10.8	12.9	4	4.2	64	45	200	149	28.57143	18.44262	204	843	1660	1870	2.27626	NA	NA	Class A
1088	80	M	21.13554	0	0	0	1	0	1	0	0	1	0	0	0	0	0	0	0	0	0	NA	NA	NA	NA	1.89	0.72	27.48943	78.73082	8.9	12.8	3	2.8	22	48	187	135	13.83648	21.91781	39900	4080	2300	483	15.475	4.86957	NA	Class A
1089	72	M	22.85446	0	0	0	1	0	1	0	0	0	0	0	0	1	1	1	0	0	0	127	132	59	60	3.12	NA	16.37352	NA	10.8	NA	3.5	NA	84	44	75.7	121	31.81818	18.33333	2150	4940	1200	2630	3.05678	NA	5.6	Class A
1090	79	M	27.22656	0	0	1	0	0	1	0	0	1	0	0	0	0	0	0	0	0	1	129	149	70	87	2.49	2.48	20.40519	20.49522	9.2	11.8	3.6	3.8	63	77	8.8	53.5	20.93023	27.89855	275	189	172	64	0.25175	0.47321	NA	Class A
1091	58	M	NA	1	0	0	0	0	1	0	0	1	0	0	0	0	0	0	0	1	1	125	137	79	74	4.92	5.37	10.58493	9.61847	10	10.6	4.2	4	83	100	23	28.7	31.08614	37.59398	104	102	1320	1140	1.54846	3.2655	4.7	Class B
1092	60	M	NA	0	0	1	0	0	1	0	0	0	1	0	0	0	1	1	0	0	0	NA	115	NA	76	1.39	1.45	41.7844	39.89659	10.9	12.7	3.6	3.2	70	74	350	323	31.53153	33.94495	1630	6260	349	439	0.70455	1.4898	4.9	Class C
1093	77	F	21.20473	0	0	0	1	1	0	0	1	0	0	0	0	0	1	1	0	0	0	142	103	69	45	0.84	0.74	49.87257	57.07889	10.7	8.1	3.7	2.5	103	55	10.6	79.3	32.38994	20.75472	416	13900	111	186	0.83505	0.58333	5.4	Class A
1094	68	F	19.10422	0	1	0	0	1	0	0	0	0	0	0	0	0	0	0	0	1	1	128	128	68	68	1.38	1.07	30.02537	39.6616	9.6	11.2	3.9	3.8	54	36	22.4	43.7	16.46341	13.18681	1090	797	913	903	0.02899	0.11236	6.7	Class A
1095	48	M	26.89879	1	0	0	0	0	1	0	1	1	0	0	0	0	1	1	0	0	0	146	137	96	84	1.54	1.95	39.82335	30.5786	10.9	12.3	3	2.7	76	51	215	105	33.62832	22.66667	7230	7200	129	223	12.35867	18.11744	8.1	Class A
1096	82	M	22.19742	0	1	0	0	0	1	0	0	0	0	0	0	0	0	0	0	1	1	121	124	57	56	1.67	1.6	31.25254	32.7514	8.7	8.8	3.7	3.5	119	106	45.8	72.6	35	33.97436	3550	8770	1210	3440	0.4803	NA	NA	Class B
1097	70	M	22.58118	1	0	0	0	1	0	0	1	1	0	0	0	0	1	1	0	0	0	171	151	92	77	5.27	5.43	9.30246	9.00301	9.8	11.3	3.6	3.7	79	74	263	146	31.34921	29.36508	109	304	362	378	3.60465	3.35888	5.3	Class B
1098	52	M	39.67707	0	0	1	0	0	0	0	1	0	0	0	0	0	0	0	0	0	0	152	162	76	80	4.15	4.85	13.08549	11.034	8.9	11	3.5	3.5	118	108	613	502	44.19476	45.76271	27400	38600	772	746	3.94495	4.74699	NA	Class C
1099	73	F	16.66441	0	0	1	0	1	0	0	1	0	0	0	0	0	1	1	0	0	0	140	131	80	80	1.98	1.92	19.82078	20.49939	10.4	11.7	4	3.8	91	82	101	92.8	31.92982	31.06061	4220	16500	854	403	0.28436	0.33491	5.1	Class A
1100	77	F	19.84714	1	0	0	0	0	0	0	1	1	0	0	0	0	0	0	0	0	0	176	146	78	72	1.78	1.58	21.93127	24.8934	10.9	10.8	4	4	97	68	22.5	23.9	34.03509	24.81752	330	155	152	206	1.10599	1.03448	6.5	Class A
1101	59	F	21.31189	1	0	0	0	1	0	0	0	1	0	0	0	0	0	0	0	0	1	132	132	71	70	3.69	3.61	10.66313	10.86936	8.7	10.6	3.8	4	45	39	127	53.3	21.73913	15.85366	791	443	655	585	5.75472	5.05814	5.6	Class A
1102	68	F	20.02492	0	0	0	1	1	0	0	0	0	0	0	0	0	1	1	0	0	0	127	120	75	73	3.01	2.4	12.79276	16.38947	10.2	10.6	3.7	3.9	85	68	13.9	8.8	27.41935	20.42042	91	103	333	358	0.61702	NA	NA	Class A
1103	72	M	17.31341	0	0	1	0	0	1	0	0	0	0	0	0	0	1	0	1	0	0	123	141	79	70	2.28	1.26	23.07632	43.97671	9.5	10.6	3.5	3.4	61	115	197	132	22.18182	47.71784	704	6190	164	656	2.80612	16.60053	5.3	Class A
1104	71	F	27.40918	1	0	0	0	1	0	0	1	1	1	0	0	0	1	1	0	0	0	154	178	80	105	2.01	1.79	19.65344	22.22135	10.6	11.3	3.3	3.1	50	79	86.8	80.7	19.84127	33.76068	1700	1760	663	1010	4.42602	6.86515	NA	Class A
1105	52	M	20.97547	1	0	0	0	0	0	1	1	1	0	0	0	0	1	1	0	0	0	155	148	86	83	1.55	NA	38.64434	NA	10.7	NA	3.4	NA	83	93	67	56.7	33.06773	36.75889	50	151	205	198	4.48611	NA	NA	Class A
1106	73	M	22.23099	0	1	0	0	0	1	0	1	0	0	0	0	0	0	0	0	0	0	153	152	70	78	2.08	2.12	25.41363	24.88953	10.9	12.3	4.3	3.7	70	92	48.5	22.2	20.46784	26.21083	3910	551	553	172	4.08122	6.09594	NA	Class A
1107	79	F	20.99558	0	0	0	1	0	1	0	1	0	0	0	0	0	1	1	0	0	0	150	144	66	86	4.49	5.07	7.9117	6.92705	8	9	2.8	3.1	77	120	41.8	13.6	34.22222	45.28302	218	457	1520	4060	8.36111	8.71698	NA	Class A
1108	67	M	16.95502	0	1	0	0	0	1	0	0	0	0	0	0	0	0	0	0	0	0	132	134	68	70	1.4	1.33	40.16559	42.48392	10.1	10.5	4.3	4.3	35	67	53.5	63.8	11.86441	21.13565	7790	6310	228	292	1.43333	2.2	NA	Class A
1109	63	F	25.29218	0	0	1	0	1	0	0	1	0	0	0	0	0	0	0	0	0	0	148	137	78	83	2.64	2.84	15.09387	13.93494	8.8	12.5	4.3	4.3	45	88	81.4	223	21.12676	36.06557	328	56	1310	170	1.54355	1.5625	NA	Class A
1110	68	F	19.57285	1	0	0	0	1	0	0	0	1	0	0	0	0	0	0	0	0	0	130	139	66	65	2.69	2.05	14.46662	19.39268	9.8	10.9	4	3.9	64	44	148	101	27.23404	19.46903	810	2600	167	98	0.24419	0.12281	NA	Class A
1111	52	F	25.46029	1	0	0	0	0	0	1	0	1	0	1	0	0	1	1	0	0	0	130	150	76	90	4.77	5.35	8.34938	7.36435	10.6	11.5	4.2	4.7	47	76	180	193	19.58333	29.00763	413	211	653	679	1.85	1.42667	5.3	Class B
1112	65	F	21.03028	0	0	0	1	1	0	0	0	0	0	0	0	0	1	1	0	0	0	120	NA	72	NA	1.71	NA	24.05705	NA	10.7	NA	4.3	NA	145	115	234	173	52.15827	40.78014	198	6600	317	343	0.05208	NA	5.3	Class A
1113	67	M	20.75522	0	0	1	0	1	0	0	1	0	0	0	0	0	1	1	0	0	0	140	145	86	89	4.9	5.9	10.20102	8.2901	8.6	13.1	3.7	4.3	55	62	229	128	23.50427	23.57414	193	232	1330	780	3.94541	4.18848	NA	Class A
1114	60	M	NA	1	0	0	0	0	1	0	0	1	0	0	0	0	1	1	1	0	0	139	147	70	78	2.91	3.01	18.61973	17.85913	9.7	9.7	2.7	3.2	101	86	140	132	40.72581	34.67742	113	53	1230	770	NA	8.10811	6.1	Class A
1115	61	M	28.91488	0	1	0	0	1	0	0	0	0	0	0	0	0	0	0	0	0	0	120	NA	82	NA	3.35	3.65	15.88594	14.46318	10	11.4	3.6	3.6	54	78	105	93.7	21.09375	30.95238	537	1750	218	244	NA	3.70769	NA	Class A
1116	78	F	25.1747	1	0	0	0	1	0	0	0	1	0	0	0	1	0	0	0	0	0	132	123	75	70	3.75	4.33	9.66996	8.26223	10.4	11.4	4.3	4.3	60	86	22.4	18.2	18.98734	24.57143	2180	2400	355	342	2.12528	3.84462	6.8	Class A
1117	64	F	16.21771	0	1	0	0	1	0	0	0	0	0	0	0	0	0	0	0	0	0	121	128	74	79	3.82	4.22	10.02987	8.99458	10.1	11.1	4	4.1	49	83	40.4	32.7	17.01389	26.77419	353	414	311	291	0.69486	0.56738	NA	Class A
1118	60	M	20.80275	0	0	1	0	1	0	0	0	0	0	0	0	0	0	0	0	0	0	131	136	79	82	1.87	2.26	30.20491	24.43529	10.4	12.8	3.3	3.5	65	104	178	115	29.68037	39.54373	1180	140	306	130	3.96825	2.04959	NA	Class B
1119	61	M	24.56747	0	0	0	1	0	0	1	1	0	0	0	1	0	1	1	0	0	1	147	137	97	94	5.21	5.71	9.79922	8.86446	10.3	10.9	3.4	3.4	82	44	73.1	27.8	27.24252	15.49296	464	431	600	505	9.44752	2.89776	NA	Class B
1120	57	M	20.81032	0	0	1	0	1	0	0	0	0	0	0	0	0	0	0	0	0	0	110	120	70	80	3.22	NA	16.91497	NA	10	NA	4.1	NA	96	135	65.6	59.9	37.2093	48.91304	136	146	260	225	0.53125	NA	5	Class A
1121	57	F	24.56659	0	0	1	0	1	0	0	0	0	0	0	0	0	1	1	0	0	0	126	133	81	88	3.2	3.55	12.58566	11.17873	10.5	11.2	4	3.9	88	60	64.9	60.8	30.03413	20.76125	295	296	238	167	2.10541	2.50303	NA	Class A
1122	82	M	19.81405	0	1	0	0	0	1	0	0	1	1	1	0	0	0	0	0	0	0	123	107	62	46	2.38	NA	21.21104	NA	9.9	NA	4.1	NA	68	104	154	95.3	22.51656	34.09836	887	293	2350	3050	0.12676	NA	NA	Class B
1123	73	M	NA	0	0	1	0	0	1	0	0	1	1	0	1	0	0	0	0	0	1	124	151	52	75	3.46	3.16	14.56394	16.02044	9.6	11.4	3.6	4	68	80	105	50.8	26.5625	29.85075	40700	170	3740	1710	0.30556	NA	6.4	Class A
1124	78	M	24.97399	1	0	0	0	0	1	0	0	1	0	0	0	0	0	0	0	0	0	114	120	64	78	1.23	1.43	44.30109	37.43218	9.4	10.7	3.7	4	71	50	122	104	37.36842	25.12563	229	765	123	131	0.20668	0.48842	7	Class A
1125	69	M	22.78147	0	1	0	0	0	1	0	1	1	0	0	0	0	0	0	0	0	0	172	138	98	88	3.37	3.65	15.23439	13.90305	9.4	9.9	3.4	3.6	64	81	167	118	26.22951	35.84071	1630	801	11100	13500	NA	2.05811	NA	Class A
1126	89	F	23.96798	0	0	1	0	1	0	0	1	1	0	0	0	0	1	1	1	0	0	140	134	54	52	2.17	2.29	16.93886	15.9191	10.7	11.4	4	3.9	76	65	103	87.1	30.4	25.39063	117	538	987	742	0.05435	0.03947	6.5	Class A
1127	73	F	27.6243	0	0	1	0	1	0	0	0	0	0	0	0	0	1	1	0	0	0	133	138	71	88	3.49	3.86	10.66158	9.54874	10.1	10.8	4.1	4	101	107	105	81.9	45.90909	51.69082	537	1270	286	308	1.11072	0.72127	5.6	Class A
1128	76	M	22.51808	0	0	1	0	0	1	0	1	0	0	0	0	0	1	1	0	0	0	143	144	49	66	3	NA	16.8282	NA	10.2	NA	3.5	NA	58	NA	124	NA	26.85185	NA	13200	NA	1550	NA	0.96712	NA	NA	Class A
1129	75	F	21.17781	1	0	0	0	0	1	0	1	1	0	0	0	0	1	1	0	0	1	150	143	76	73	1.76	1.81	22.37241	21.69718	10.6	13.4	3.8	3.6	59	31	23.4	56.6	23.04688	13.41991	60	1780	219	287	4.8072	7.18523	5.6	Class A
1130	69	F	21.58837	0	0	0	1	1	0	0	1	0	0	0	0	0	1	1	1	0	0	145	150	85	82	3.65	3.87	10.31687	9.677	9.9	10.2	4.1	3.8	65	75	33	27.8	22.10884	25.86207	70	55	728	1100	2.94118	2.05405	NA	Class B
1131	53	M	27.34847	1	0	0	0	0	1	0	1	1	1	0	0	0	1	1	0	0	0	161	167	73	79	4.51	5.13	11.94718	10.37686	10.4	12	4.3	4.3	79	110	38.1	48.5	27.14777	39.71119	1290	214	646	865	2.9636	3.3222	5.9	Class A
1132	85	M	25.0724	0	1	0	0	0	1	0	1	1	0	0	0	0	0	0	0	0	0	194	158	84	76	2.61	2.7	18.97815	18.28717	10.8	11.9	3.7	3.5	92	85	349	268	40.17467	43.14721	265	245	925	1140	2.79355	1.85479	5.7	Class A
1133	62	M	25.98728	0	0	0	1	0	0	1	0	1	0	0	1	1	1	1	0	0	1	118	91	51	53	4.67	5.53	10.99392	9.09598	10.3	11	4.2	4.1	68	50	248	63.1	27.64228	18.11594	2810	628	2700	1200	1.57718	2.7533	5.6	Class A
1134	74	M	23.23862	0	1	0	0	0	1	0	1	0	1	0	0	0	1	1	0	1	1	142	120	80	56	3.7	2.57	13.48092	20.00748	9.7	11.7	4	3.5	48	35	26.6	44.1	14.11765	13.01115	2200	4150	924	1280	0.72449	0.34174	5.7	Class A
1135	86	F	19.96024	0	1	0	0	1	0	0	1	0	0	0	1	0	0	0	0	0	0	144	NA	77	NA	1.23	NA	31.83383	NA	10.8	NA	3.7	NA	29	NA	83.8	NA	12.66376	NA	11200	NA	347	NA	0.23566	NA	5.8	Class A
1136	72	F	23.23632	0	0	1	0	1	0	0	0	0	0	1	0	0	0	0	0	1	1	134	125	75	72	2.37	2	16.28163	19.60405	8.9	10.8	3.3	3.6	39	66	201	53.5	19.21182	26.4	12600	1160	549	987	1.69512	2.39726	NA	Class A
1137	58	F	17.10731	0	0	0	1	0	0	0	0	0	0	0	0	0	1	1	0	0	0	107	112	61	80	1.72	1.75	24.69871	24.23588	10.4	11.6	4.7	4.6	83	102	75.6	69	27.48344	33.44262	93	88	97	89	0.3527	1.26263	NA	Class A
1138	73	M	18.51694	0	0	0	1	0	0	1	0	0	0	0	0	0	1	1	0	0	1	92	112	58	64	2.21	2.27	23.7828	23.09595	9.9	13.2	3.2	3.2	52	NA	238	NA	19.54887	NA	930	NA	366	NA	2.46226	2.87263	5.7	Class A
1139	80	M	17.61719	1	0	0	0	1	0	0	0	1	0	0	0	0	0	0	0	0	0	136	140	70	70	1.03	0.87	53.40365	64.23635	10.9	12.3	4.1	4.5	68	68	21.5	17	19.65318	17.17172	320	349	51	38	0.45714	0.94444	7	Class A
1140	85	M	NA	0	0	1	0	0	1	0	0	0	1	0	1	0	0	0	0	0	0	131	NA	60	NA	3.24	3.5	14.98036	13.76728	8.5	9.4	NA	NA	67	63	233	168	25.96899	24.32432	148	126	813	785	0.62439	0.68226	NA	Class B
1141	83	M	18.73278	0	0	0	1	0	1	0	0	0	0	0	0	0	1	1	0	0	0	127	130	65	65	2.58	2.94	19.35149	16.77468	8.9	8.9	3.1	3.1	123	115	470	434	59.13462	53.73832	187	118	622	754	3.1668	2.72891	NA	Class A
1142	77	F	22.70451	1	0	0	0	1	0	0	1	1	0	0	0	0	1	1	0	0	0	198	119	102	71	3.4	6.16	10.80402	5.61841	9.5	11.5	4	4	61	28	48.3	31.3	18.37349	8.333333	1930	25200	613	856	5.04673	4.36141	5.6	Class B
1143	43	F	27.82931	1	0	0	0	0	0	1	1	1	0	0	0	0	1	1	0	1	1	161	131	90	82	2.69	3.42	16.5003	12.60523	10.6	13.8	2	2.3	57	76	16.4	64.9	23.84937	35.84906	5130	3380	219	245	21.15546	13.22581	8.2	Class A
1144	34	M	NA	0	0	1	0	0	1	0	0	0	0	0	0	0	1	1	0	0	0	NA	142	NA	78	2.84	2.87	22.50801	22.0664	11.7	12.3	4.1	4.4	160	76	184	72.7	61.53846	26.0274	438	1900	163	96	0.21186	0.66279	5.9	Class A
1145	58	F	26.29215	0	0	0	1	0	1	0	1	1	0	0	0	0	1	0	1	0	1	155	128	82	72	1.96	2.03	21.40988	20.60353	10.1	11.4	3.6	3.6	72	77	36.5	37.3	26.96629	29.84496	10400	2230	219	123	6.42424	4.16923	7.5	Class A

1146	61	M	17.81962	0	0	0	1	1	0	0	0	0	0	0	0	0	0	0	0	0	0	125	NA	85	NA	2.89	NA	18.67196	NA	9.5	NA	4	NA	70	37	219	108	20.89552	10.22099	851	4330	98	36	NA	NA	NA		Class A
1147	44	M	30.00949	0	0	1	0	1	0	0	0	0	0	0	0	0	1	0	1	0	0	133	132	89	92	6.11	6.25	9.04069	8.81938	10.1	11	4	3.9	61	98	63.4	46.8	20.89041	35.63636	5790	3000	510	713	1.47427	1.5807	NA		Class A
1148	84	M	21.21094	0	0	0	1	1	0	0	1	0	0	0	0	0	1	1	0	1	1	159	148	78	76	3.09	2.81	15.83139	17.56503	7.7	11.8	2.6	3.3	84	113	100	35	43.75	43.79845	1170	226	331	389	1.15544	5.74402	NA		Class B
1149	63	M	21.15529	0	0	1	0	0	1	0	1	0	0	0	0	0	1	1	0	0	0	150	169	90	104	3.07	4.2	17.31656	12.29012	9.8	12.5	2.2	1.9	56	54	383	288	26.66667	31.95266	510	469	1940	2780	7.92707	7.73994		5.6	Class A
1150	35	F	22.81186	1	0	0	0	1	0	0	0	1	0	0	0	0	1	1	0	0	0	109	100	68	60	1.38	1.36	36.33046	36.91536	9.6	10.3	3.3	3.5	56	79	51.9	48.4	25.45455	32.91667	260	181	179	193	1.13924	1.35976		8.5	Class A
1151	83	F	17.8536	0	0	0	1	1	0	0	1	0	0	0	0	0	0	0	0	0	0	140	135	102	69	2.81	2.58	12.98056	14.25168	10.8	9.2	4.5	4.2	13	27	11.8	10.9	3.376623	6.852792	499	70	519	570	1.47067	1.35771	NA		Class A
1152	67	M	25.9717	0	0	0	1	1	0	0	0	0	0	0	0	0	1	1	0	0	0	124	144	65	81	3.72	3.86	13.78937	13.18697	9.4	10.8	4.2	4.2	71	103	40.9	35.2	24.23208	31.69231	134	470	518	424	0.6561	NA	NA		Class B
1153	79	M	23.21799	0	1	0	0	0	1	0	1	0	0	0	1	0	0	0	0	0	0	156	167	56	62	1.95	1.74	26.66152	30.09221	8.6	10.5	4	4.1	43	47	168	77.2	18.29787	18.07692	6650	1500	150	130	1.08919	1.37572	NA		Class A
1154	77	M	21.10791	0	0	0	1	0	1	0	0	1	0	0	0	0	1	1	0	0	0	120	NA	52	NA	1.97	2.14	26.56029	24.26086	8.5	9.4	2.9	2.5	56	72	13.6	14.2	16.09195	23.45277	997	1690	1480	875	6.90909	NA		6.4	Class A
1155	80	M	25.63692	0	0	0	1	1	0	0	1	0	0	0	0	0	0	0	0	0	0	140	150	78	80	2.45	3.47	20.69508	14.14147	10.8	9.9	3.9	2.9	49	108	86.3	95.3	18.49057	42.68775	877	813	13000	17500	4.8764	10.65385	NA		Class A
1156	62	M	NA	0	0	1	0	0	0	1	0	0	0	0	0	0	0	0	0	0	0	NA	NA	NA	NA	1.28	1.6	45.29994	35.48772	11.1	10.7	3.8	4.5	134	61	243	95.6	37.11911	17.52874	4770	1290	167	426	0.39716	0.1005		4.6	Class A
1157	39	F	18.86717	0	0	1	0	1	0	0	1	0	0	0	0	0	1	1	0	1	1	166	161	89	97	3.22	3.24	13.8376	13.74418	8.8	11.2	4.25	4.19	18	61	37.9	23.8	6.338028	20.40134	19500	121	140	185	NA	0.77657	NA		Class A
1158	81	M	19.4674	1	0	0	0	0	0	0	0	1	1	0	0	0	1	1	0	0	0	132	132	60	52	1.79	NA	29.07002	NA	9.7	NA	3.7	NA	61	90	35.9	56.5	23.28244	32.96703	143	228	436	230	0.002083	NA		8.1	Class A
1159	56	F	26.86634	1	0	0	0	1	0	0	1	1	0	0	0	1	1	1	0	0	0	142	138	72	77	1.41	1.53	31.00777	28.35723	7.9	9.9	3.8	3.8	69	76	241	113	33.82353	32.75862	651	430	225	89	2.63735	2.13547		5.5	Class A
1160	90	M	18.63074	0	1	0	0	1	0	0	1	0	0	0	0	0	1	1	0	0	0	160	162	75	81	1.89	1.88	26.57571	26.64576	10.1	11	3.7	3.9	71	107	61.5	28.1	26.39405	35.90604	552	744	516	473	0.12	NA		5	Class A
1161	65	M	28.58131	0	0	1	0	0	0	0	0	0	0	0	0	0	0	0	0	0	0	134	128	78	76	5.12	5.1	9.80741	9.84949	10.6	11.3	4.1	4.3	90	93	131	84.5	31.91489	35.09434	474	339	110	180	1.77551	2.82813	NA		Class A
1162	77	M	23.1405	1	0	0	0	0	1	0	1	1	0	0	0	0	1	1	0	0	0	153	136	61	58	2.23	NA	23.19174	NA	9.1	NA	4	NA	76	61	202	182	32.75862	26.75439	5470	1960	2590	4710	3.25557	NA		4.8	Class B
1163	72	M	21.59136	0	0	1	0	0	1	0	0	0	1	0	0	0	1	1	0	0	1	118	119	74	73	1.29	1.26	42.85909	43.97671	10.9	11.6	4	3.9	78	82	113	16.4	20.3125	24.18879	124	50	193	204	1.08108	0.71336	NA		Class A
1164	69	M	24.80159	0	0	0	1	1	0	0	0	0	0	0	0	0	0	0	0	0	0	130	NA	64	NA	3	4.52	17.30141	11.00366	8.3	8.2	3.3	3.7	37	37	64.1	53.1	14.28571	14.56693	5000	4800	20700	13200	NA	NA		5.8	Class A
1165	73	M	23.58833	0	0	1	0	1	0	0	0	0	0	0	0	0	0	0	0	1	1	110	134	65	82	1.25	1.31	44.36173	41.97952	11.3	10.6	4.2	4.1	147	125	187	335	54.04412	50.40323	737	5110	413	240	NA	0.03	NA		Class A
1166	83	M	30.84628	1	0	0	0	1	0	0	1	1	0	0	0	0	0	0	0	0	0	157	126	69	64	1.6	NA	32.63766	NA	11.3	NA	3.9	NA	34	NA	23.8	NA	9.418283	NA	12400	NA	534	NA	1.24742	NA		7.6	Class A
1167	91	M	21.875	0	1	0	0	0	1	0	0	0	0	0	1	0	0	0	0	0	0	125	125	80	75	2.76	2.66	17.50662	18.1708	10.7	11.2	3.5	3.4	71	138	79	82	29.70711	58.22785	114	50	740	514	0.14851	0.45399		5.9	Class A
1168	80	M	NA	0	0	0	1	0	1	0	1	1	0	0	0	1	1	0	0	0	0	145	144	65	72	3.93	3.39	12.34097	14.50696	6.9	9.4	2.6	2.8	89	61	700	319	38.52814	24.4	940	640	1920	4600	1.85714	1.63415		4.8	Class A
1169	81	M	26.80163	0	1	0	0	1	0	0	0	0	0	0	0	0	0	0	0	0	0	108	NA	73	NA	1.18	1.23	45.85932	43.82383	8.8	13	3.2	4	47	113	84.4	43.2	20.61404	38.43537	4530	423	387	327	NA	0.02151	NA		Class A
1170	73	M	25.81663	0	0	1	0	0	1	0	0	1	0	0	1	1	1	1	0	0	1	111	123	85	54	2.06	2.18	25.68368	24.14108	10.6	13	3.9	4	57	88	41.2	54.9	17.37805	25.28736	828	816	105	74	0.36107	0.83333		6.4	Class A
1171	83	F	19.68796	1	0	0	0	1	0	0	1	1	1	1	0	0	0	0	0	0	0	164	134	74	60	1.84	2.24	20.69951	16.69166	8.9	11.2	3.6	4.1	64	78	384	288	27.58621	33.33333	1870	444	755	352	1.43556	0.68157		7	Class A
1172	86	M	27.24878	1	0	0	0	1	0	0	0	1	1	0	0	0	0	0	0	0	0	124	NA	68	NA	1.73	2.19	29.66048	22.84093	10.9	10.9	3.8	NA	69	NA	25.7	NA	22.84768	NA	927	NA	354	NA	NA	NA			Class A
1173	65	M	23.73866	1	0	0	0	0	0	1	0	1	0	0	0	0	1	1	0	0	0	134	137	70	74	3.14	3.95	16.74382	13.02622	9.1	11.3	4.2	3.3	48	76	126	78.9	16.49485	27.04626	1040	1520	641	927	2.01149	8.45035		6	Class A
1174	79	M	19.21565	0	1	0	0	0	0	1	0	0	0	0	0	0	1	1	0	0	0	120	141	62	74	3.77	4.09	12.9618	11.8138	7.9	7.6	3.1	3.3	46	44	188	98.3	26.13636	25.43353	13300	6640	12100	14800	7.03333	NA	NA		Class A
1175	68	M	22.13374	0	1	0	0	0	1	0	1	0	0	0	0	0	1	1	0	0	0	146	144	96	92	3.51	3.77	14.63207	13.53176	10.3	11.5	4	3.9	90	79	252	217	35.01946	32.64463	1100	2850	291	334	0.04253	3.34869	NA		Class B
1176	89	F	NA	1	0	0	0	1	0	0	0	1	1	0	0	0	0	0	0	0	1	NA	NA	NA	NA	1.52	NA	25.00543	NA	8.8	NA	4	NA	85	86	81	97.3	37.1179	37.88546	96	300	5240	7930	NA	NA			Class B
1177	78	F	17.84238	0	1	0	0	1	0	0	0	0	0	1	0	0	1	1	0	0	0	91	84	55	44	2.06	1.79	18.62277	21.7167	10.5	11.3	4.6	4.5	80	89	69.7	33	22.34637	20.74592	2880	139	1910	1790	0.0597	0.16949		6	Class B
1178	72	M	25.74448	1	0	0	0	0	1	0	1	1	0	0	0	0	1	0	1	0	0	153	145	83	80	3.65	4.71	13.7911	10.39305	10.2	11.3	4	3.9	88	78	58.8	50.5	27.07692	23.85321	321	1160	615	359	1.80392	1.52857		7.7	Class A
1179	74	M	25.90064	0	0	0	1	0	1	0	0	0	0	0	0	0	1	1	0	0	0	114	132	60	75	3.41	4.45	14.74005	11.01606	9.9	10.9	3.8	3.5	89	33	95.7	75	38.19742	15.34884	481	15100	380	381	1.62	2.22951		5.6	Class A
1180	66	M	23.98668	1	0	0	0	0	1	0	0	1	0	0	0	1	1	1	0	0	1	117	117	66	59	2.7	2.21	19.6644	24.37548	9.9	12.8	4	3.6	71	64	134	76.6	27.84314	26.66667	270	1070	237	559	0.42572	3.32975		6.4	Class A
1181	80	F	23.54006	1	0	0	0	0	1	0	0	1	0	0	0	1	1	1	0	1	1	136	121	66	50	1.34	1.62	29.59421	23.96081	9.1	10.2	4.3	4.4	153	120	235	206	59.76563	48.78049	77	134	309	287	0.60729	0.12305		7.3	Class A
1182	78	F	NA	0	0	1	0	1	0	0	0	0	0	0	0	0	1	1	0	0	0	NA	111	NA	57	0.83	NA	50.34352	NA	10.9	NA	3.5	NA	61	NA	20	NA	19.61415	NA	506	NA	387	NA	0.075	NA			Class A
1183	76	M	15.17578	0	1	0	0	1	0	0	0	0	0	0	0	0	0	0	0	0	0	138	126	68	64	2.89	NA	17.53016	NA	10.8	NA	3.9	NA	70	97	48.3	17.4	24.39024	34.51957	517	307	1220	1870	0.46512	NA		5.4	Class A
1184	61	F	22.52206	1	0	0	0	1	0	0	1	0	1	0	0	0	1	1	0	0	1	111	113	56	54	4.07	4.15	9.48768	9.28778	9.8	10.4	4.3	4.2	70	87	119	100	26.81992	34.11765	8550	830	4060	8740	3.1405	3.23336		8.3	Class A
1185	67	M	16.80547	0	0	0	1	0	1	0	0	1	0	0	0	0	1	1	0	0	0	125	155	73	88	1.75	2.58	31.4655	20.57815	10.8	11.7	3.4	3.9	51	62	48.3	32.1	19.69112	21.23288	2380	81	144	153	0.39286	0.56667		6.1	Class A
1186	77	M	29.72108	0	0	1	0	0	0	1	1	0	0	0	0	0	1	1	0	0	0	141	148	76	95	3.53	3.67	14.0318	13.44727	11.1	12	4.4	4.3	123	82	70.4	60	46.2406	30.59701	368	323	832	870	0.46689	0.43187		5.3	Class A
1187	64	M	19.0625	0	1	0	0	0	0	1	0	0	0	0	0	0	0	0	0	0	0	126	140	76	92	3.82	4.1	13.57222	12.50576	9.8	10.8	3.8	3.5	54	58	131	105	24.77064	26.00897	3170	2200	1640	5950	2.85965	4.69231	NA		Class A
1188	68	M	25.32958	1	0	0	0	0	0	1	0	1	0	0	1	0	1	1	0	0	0	139	140	83	70	2.85	2.93	18.37686	17.82865	11.1	12.7	3.7	3.4	67	50	56	35.1	22.40803	18.58736	1180	4060	166	137	2.22772	2.26512		6.8	Class A
1189	86	F	14.88447	0	1	0	0	0	1	0	0	0	0	0	0	0	0	0	0	0	1	105	110	62	60	1.88	1.94	20.01319	19.33704	10.9	11.9	4.1	4	53	47	56.9	39.4	22.26891	18.28794	116	115	427	450	1.95854	2.447	NA		Class A
1190	73	M	28.1473	0	0	0	1	1	0	0	0	1	0	0	0	1	1	1	0	0	0	104	102	51	52	1.45	1.98	37.71303	26.82109	10.6	12.8	4.1	4	91	112	139	90.6	29.07348	36.72131	593	919	126	168	0.08292	0.11566	NA		Class A
1191	84	F	19.95337	0	0	1	0	1	0	0	1	0	0	0	0	0	1	1	0	0	0	145	130	61	64	1.48	1.59	26.17651	24.20191	10.3	9.8	3.5	3.5	65	44	15.4	14.4	18.95044	13.83648	194	356	398	515	5.35135	4.27793	NA		Class A
1192	81	M	16.43918	0	0	1	0	1	0	0	1	0	0	0	0	0	1	1	0	0	0	143	149	62	64	1.69	NA	30.95697	NA	10.5	NA	4	NA	73	NA	103	NA	26.07143	NA	50	NA	103	NA	0.19231	NA			Class A
1193	67	M	23.26997	1	0	0	0	0	0	1	0	1	0	0	0	0	1	0	1	0	0	122	183	80	99	2.1	2.13	25.77569	25.37879	9.2	12.5	3	3	59	81	439	487	23.98374	34.76395	482	455	235	412	2.66547	7.16024		6.8	Class A
1194	69	F	23.66603	0	0	0	1	1	0	0	1	0	0	0	0	0	1	1	0	0	0	147	140	94	70	3.73	3.83	10.07505	9.74728	10.2	9.3	4.3	4	77	97	42	29.7	19.39547	22.66355	50	50	983	834	2.09917	2.59968	NA		Class C
1195	78	M	22.74642	1	0	0	0	0	0	0	1	1	0	0	0	0	1	1	0	0	0	144	NA	67	NA	4.42	6.8	10.93147	6.82348	8.9	10.4	3.2	2.2	65	NA	42.7	NA	27.54237	NA	678	NA	1150	NA	8.92857	NA	NA		Class B
1196	52	M	22.40163	0	0	0	1	0	1	0	1	0	0	0	0	1	0	1	0	0	0	164	164	68	94	5.8	7.27	9.1226	7.12509	11	11	4.2	3.7	46	30	24.6	15.3	12.16931	8.287293	540	8560	267	721	2.63456	3.77676	NA		Class B
1197	70	F	21.82921	0	0	1	0	1	0	0	0	0	0	0	0	0	1	1	0	0	0	131	121	76	67	1.49	1.65	27.38024	24.38976	10.7	11	4.2	4.2	77	53	41.2	28.2	24.75884	16.40867	97	75	116	116	0.35	0.13514	NA		Class A
1198	70	M	22.18783	0	0	1	0	1	0	0	1	1	0	0	0	0	1	1	0	0	0	171	140	86	60	3.2	2.7	16.05553	19.33511	9.4	12.8	3.4	3.5	59	NA	207	NA	30.72917	NA	161	NA	781	NA	4.57726	15.32143		5.4	Class A
1199	79	M	20.25888	0	1	0	0	0	1	0	1	0	1	0	0	0	1	1	0	0	0	149	135	75	60	2.05	2.12	25.24202	24.24396	10.3	12.4	3.7	3.9	102	100	184	106	41.12903	39.52569	134	1280	225	591	0.79186	2.17109	NA		Class A
1200	80	M	30.19059	0	0	1	0	0	1	0	1	0	0	0	0	0	0	0	0	0	1	140	124	70	60	1.95	2.23	26.56544	22.85709	10.2	12.5	4	4.3	28	37	17.2	20.8	9.427609	11.59875	1910	780	148	88	1.1	1.5	NA		Class A
1201	72	M	NA	1	0	0	0	1	0	0	1	1	0	0	0	0	0	0	0	0	0	153	136	80	75	1.66	2.16	32.65495	24.48245	10.2	10.4	3.9	3.8	27	29	12.2	15.8	6.75	7.837838	2470	27800	112	131	0.001493	0.20367		6.4	Class A
1202	76	M	22.48133	0	0	0	1	1	0	0	1	0	0	0	0	0	1	1	0	0	0	135	149	48	56	2.6	2.54	19.6801	20.18925	10.2	10.9	NA	3.4	NA	104	NA	40.5	NA	38.37638	NA	165	NA	488	2.68	6.72414		5.8	Class A
1203	65	F	38.47058	1	0	0	0	1	0	0	1	1	0	0	0	0	1	1	0	0	0	150	127	68	69	1.77	1.83	23.16633	22.33668	10.1	11	3.6	3.5	50	54	45	35.3	15.2439	16.36364	217	235	141	177	1.15385	1.3913		6	Class A
1204	78	M	26.74341	0	0	1	0	1	0	0	1	0	0	0	0	0	0	0	0	0	0	146	151	72	67	1.63	2.04	32.55647	25.47037	10	9.7	3.8	3.9	64	72	23	20.6	19.45289	20	129	86	520	240	1.60227	1.21053		5.9	Class A
1205	57	F	21.42486	0	0	1	0	1	0	0	0	0	0	0	0	0	1	1	0	0	0	105	120	75	75	2.07	1.95	20.26929	21.63776	10.7	11.1	4.2	3.9	64	94	112	83.8	20.91503	32.52595	149	174	53	111	0.04231	0.02258	NA		Class A
1206	75	M	25.03947	0	1	0	0	1	0	0	0	0	1	0	0	0	1	1	0	0	1	125	145	79	90	4.59	4.23	10.60806	11.59959	10	10.7	4.2	4.3	59	76	48.9	43.4	17.25146	21.7765	955	753	884	671	0.36029	0.28834		5.3	Class A
1207	62	F	27.17567	0	1	0	0	1	0	0	0	0	0	0	1	0	0	0	0	1	1	130	134	70	76	3.53	3.8	11.03479	10.17997	9.1	10.6	3.5	3.6	90	104	64.2	85	35.29412	42.10526	1350	1560	410	464	3.97297	4.64103	NA		Class B
1208	80	M	21.89164	0	1	0	0	1	0	0	0	0	0	0	0	0	1	1	0	0	0	135	135	60	60	2.91	3	17.14415	16.58228	10.7	10.1	4.1	3.7	62	68	107	110	24.40945	27.7551	404	890	531	581	1.09479	0.72674		5.6	Class A
1209	55	M	19.38512	1	0	0	0	0	1	0	0	1	0	0	1	0	1	0	1	0	0	124	127	63	67	1.53	1.51	38.57138	39.13063	9.5	11.3	3.4	3.7	36	106	75.2	85.7	13.04348	31.17647	9150	655	185	197	0.45492	0.68338		5.8	Class A
1210	72	F	19.18832	0	0	1	0	0	0	1	1	0	0	0	1	0	1	1	0	0	0	150	146	76	80	2.8	3.08	13.62074	12.27205	9.5	10.8	4.3	4.3	52	35	90.9	47.9	18.57143	11.62791	869	5490	650	463	0.28	0.32857	NA		Class A
1211	79	F	21.7502	0	0	1	0	1	0	0	0	0	0	0	0	0	1	1	0	0	0	104	134	60	80	1.35	1.49	29.46063	26.44609	10.9	10.6	4	3.9	55	58	34.9	23.5	22.17742	21.09091	2130	2690	874	434	0.20313	0.08333		5.2	Class A
1212	69	M	23.9913	0	0	1	0	0	1	0	0	0	0	0	0	0	0	0	0	1	1	120	136	67	76	1.52	1.48	36.40115	37.32435	11.1	11.9	3.8	4	97	84	44.1	64.5	33.21918	28.76712	176	397	130	199	1.0549	0.52314		5.4	Class A
1213	67	M	20.75805	1	0	0	0	0	0	1	0	1	0	0	0	0	0	0	0	0	1	124	NA	75	NA	6.4	NA	7.61653	NA	9.7	NA	3.5	NA	79	107	107	23.6	27.14777	28.45745	288	429	1510	1480	0.66	NA		5.7	Class C
1214	58	M	24.29887	0	1	0	0	0	0	1	0	1	0	0	0	0	1	1	0	0	0	115	117	69	64	2.31	2.2	24.20507	25.53216	10.4	12.3	4.1	4	61	81	184	129	25.63025	30.79848	264	173	59	53	0.21875	0.29268		6	Class A
1215	69	F	19.5649	0	0	1	0	1	0	0	0	0	0	0	0	0	1	1	0	0	0	109	124	61	69	2.05	1.97	19.39268	20.25585	10.1	10.9	4.4	4.5	56	72	44.6	35.8	22.95082	27.58621	1330	793	133	203	0.09524	0.05556	NA		Class A
1216	84	M	19.33478	0	0	0	1	0	1	0	0	0	0	0	0	1	1	1	0	0	0	103	98	49	51	2.74	3.05	18.05654	16.05867	10.1	11.9	4	3.8	131	78	539	273	61.79245	41.93548	1020	528	127	223	0.12957	0.07864	NA		Class A
1217	70	M	21.85849	1	0	0	0	0	0	1	0	1	1	0	0	0	0	0	0	0	0	108	101	47	53	2.21	1.99	24.07096	26.99686	8.7	12	3.8	3.7	36	47	198	152	14.81481	19.50207	42900	13300	280	169	NA	1.15849		5.4	Class A
1218	77	M	26.71304	1	0	0	0	0	1	0	0	1	0	0	0	1	1	1	0	0	0	130	NA	68	NA	2.28	2	22.63592	26.12475	9.8	9.6	3.8	3.4	30	53	26.9	56.2	9.375	17.90541	8700	15400	281	197	1.97981	0.42857		7.2	Class A
1219	67	F	29.19423	1	0	0	0	0	1	0	1	1	0	0	0	0	1	1	0	1	1	158	158	86	79	1.46	1.55	28.3505	26.5546	9.9	11.1	3.5	3.5	45	72	45.4	58.1	16.24549	24.4898	218	168	535	334	4.88223	3.45269		6.7	Class A
1220	78	F	22.98743	0	0	1	0	1	0	0	1	0	0	0	0	0	1	1	0	0	0	140	134	67	70	2.88	3.02	12.90743	12.25427	10.4	10.9	4.2	4.2	101	117	48.3	42.3	36.72727	39.93174	263	172	261	210	2.20879	3.1		5.6	Class A
1221	83	F	20.77564	1	0	0	0	1	0	0	1	1	1	0	0	0	1	1	0	0	0	150	154	60	55	3.1	NA	11.69825	NA	8.4	NA	3.8	NA	49	27	62.5	40.8	20.50209	9.9631	99	775	754	984	0.65389	NA		6.9	Class A
1222	82	M	20.08078	0	0	0	1	0	1	0	0	0	0	0	0	0	0	0	0	0	1	88	104	51	60	1.82	1.34	28.44586	39.6254	9.7	11.1	4	3.9	112	83	56.2	10.3	41.48148	26.43312	50	52	142	131	0.02687	0.02656		6.1	Class A
1223	57	F	22.50693	0	0	1	0	1	0	0	0	0	0	0	0	0	1	1	0	0	1	96	138	61	90	2.69	2.33	15.21812	17.71964	8.7	11	3.7	3.8	44	55	18.8	28.6	13.41463	17.85714	5680	13600	289	363	0.05297	0.42328	NA		Class A
1224	62	F	27.99427	1	0	0	0	0	0	1	1	1	0	0	0	0	1	1	0	0	0	148	138	68	70	1.9	1.74	21.73068	23.81631	10.1	12.7	4	4.2	86	70	177	112	30.82437	25	50	78	570	254	1.80702	2.23913		6.1	Class A
1225	69	M	22.30815	0	0	0	1	1	0	0	0	1	0	0	0	0	1	1	0	0	0	134	121	65	56	3.18	3.58	16.23292	14.25949	10.6	12.7	3.7	3.7	92	111	173	191	37.86008	43.70079	240	84	483	310	10.06165	8.82263		6.3	Class A
1226	90	F	21.97384	1	0	0	0	1	0	0	0	1	0	1	1	0	0	0	0	1	1	126	134	56	72	2.64	2.59	13.62523	13.91325	10.1	11.7	3.7	3.8	57	69	124	83.8	22.00772	25.46125	866	388	818	1560	1.8911	4.48485		5.3	Class A
1227	82	F	18.2615	0	0	1	0	1	0	0	0	0	0	0	0	0	1	1	0	1	1	120	120	65	65	3.19	3.36	11.37718	10.74896	9.9	11.7	4	4	38	70	6.7	29.7	10.46832	25.64103	225	491	567	1610	NA	0.8	NA		Class A
1228	76	M	23.52821	1	0	0	0	0	1	0	0	1	1	0	1	1	0	0	0	0	0	139	148	55	60	3.47	3.86	14.35119	12.72484	9.6	11.1	3.4	3.9	62	85	119	142	25.40984	31.0219	5650	3460	1410	1140	2.95019	2.67806		6.4	Class B
1229	72	F	25.52866	0	0	1	0	1	0	0	0	0	0	0	0	0	1	1	0	0	0	127	138	81	94	3.49	NA	10.70387	NA	9.4	NA	3.9	NA	72	94	91.6	71.5	25.17483	33.81295	661	433	206	166	1.07221	NA	NA		Class A
1230	55	F	19.85397	0	0	1	0	1	0	0	1	0	0	0	0	0	1	1	0	0	0	135	133	90	82	2.62	2.96	15.82488	13.84742	10.5	10.6	4.3	4.5	91	66	332	300	39.56522	27.16049	59	50	323	226	0.85492	0.90323	NA		Class A
1231	62	M	22.07274	0	0	1	0	1	0	0	0	0	0	0	0	0	1	1	0	0	0	135	150	78	75	6.62	7.21	7.47089	6.80471	9.3	10.7	3.79	3.82	76	109	150	76.7	28.04428	38.79004	159	154	268	219	1.85313	2.53804	NA		Class B
1232	63	M	27.40129	1	0	0	0	0	1	0	0	1	1	0	0	1	0	0	0	0	0	138	131	67	62	2.6	4.1	20.76874	12.61843	10.7	10.5	3.8	3.6	107	78	56.2	64.8	36.02694	24.07407	146	4350	214	309	4.70817	4.80672		6.8	Class A
1233	79	F	22.92509	0	1	0	0	0	1	0	0	0	0	0	0	0	1	1	0	0	0	111	125	60	73	2.11	1.55	18.07433	25.3282	7.5	10.3	4.4	4	69	35	73	8.5	25.65056	9.433962	112	322	670	387	0.13864	0.4569		5.4	Class A
1234	84	F	21.62065	0	1	0	0	1	0	0	1	0	0	0	0	1	1	1	0	0	0	151	146	49	57	1.51	1.83	25.60809	20.68147	10.5	10.9	3.9	3.9	89	76	18.1	13.9	28.34395	23.31288	222	265	297	279	1.84874	0.75829	NA		Class A
1235	85	M	21.67291	1	0	0	0	0	1	0	0	1	0	0	0	1	1	1	0	1	1	115	164	53	75	3.33	3.65	14.53799	13.14953	8.9	10.8	4.03	4.02	96	75	17.9	32.7	30	23.51097	66	332	1240	1060	2.77188	5.58648		8.6	Class C
1236	68	M	21.4554	0	0	1	0	0	1	0	1	0	0	0	0	1	1	0	0	0	1	156	153	75	79	3.75	3.98	13.61073	12.75263	8.9	10.2	4.2	4.6	113	95	270	174	34.98452	33.5689	162	253	87	111	1.80277	0.65556		6.2	Class A
1237	65	M	26.66337	1	0	0	0	0	0	1	1	1	0	0	0	0	1	1	0	0	0	142	160	70	86	3.07	3.09	17.16193	17.04045	9	10.6	3.1	3.3	41	44	152	147	18.14159	18.80342	4170	29000	515	355	4.01901	4.16484		3.7	Class A
1238	72	M	20.42372	1	0	0	0	0	1	0	1	1	1	0	0	1	0	0	0	0	0	158	188	82	98	2.29	4.31	22.9661	11.49818	9.8	12.4	3	2.9	61	70	184	206	22.76119	30.30303	367	3880	2090	5370	7.58962	13.65019		4.5	Class B
1239	61	M	27.61977	1	0	0	0	0	1	0	1	1	0	0	1	0	0	0	0	0	0	147	148	76	85	1.94	2.44	28.87733	22.36562	10.1	10.1	3.4	2.9	66	73	89.3	75	22.14765	27.34082	163	141	604	687	6.29959	NA		6.5	Class A
1240	48	F	NA	0	0	1	0	0	0	1	0	0	0	0	0	0	1	1	0	1	1	NA	NA	NA	NA	1.38	NA	33.18197	NA	11.9	NA	4	NA	219	61	13	14.4	66.36364	20.67797	356	459	105	61	0.06897	NA		5.2	Class A
1241	78	F	24.39388	0	1	0	0	1	0	0	0	1	1	1	0	0	1	1	0	0	0	115	103	52	60	1.74	2.56	22.40032	14.68252	9.5	9.9	4.2	3.4	65	60	112	104	22.49135	24.59016	353	3810	596	266	0.07619	0.08589		6.4	Class A
1242	69	F	23.33629	0	0	0	1	1	0	0	0	0	0	0	0	0	1	1	0	1	1	132	142	80	68	2.29	1.98	17.18053	20.06094	10.3	14.5	4	4.1	74	105	69.6	33.1	25.87413	36.45833	532	1580	687	769	2.28145	2.25191	NA		Class A
1243	66	M	20.27337	1	0	0	0	0	0	1	1	1	0	0	0	0	0	0	0	0	0	146	NA	60	NA	1.62	1.62	34.38612	34.23804	9.9	9.6	3.4	3.4	40	25	216	99.6	15.5642	8.928571	15300	37600	934	675	3.52174	0.7236		6.7	Class A
1244	75	M	21.30395	0	0	1	0	1	0	0	1	0	0	0	1	0	1	0	1	0	0	135	128	60	65	2.03	1.23	25.89754	44.63258	8.7	11.4	4.5	4.5	74	78	439	291	25.08475	25.74257	4330	1040	143	102	0.1	NA		6.3	Class A
1245	73	M	26.31006	1	0	0	0	0	0	0	0	1	0	0	0	1	1	1	0	0	1	123	120	55	60	2.09	2.12	25.28064	24.88953	9.4	10.3	3.6	3.8	70	106	87.3	87	22.87582	31.73653	3760	877	429	269	1.07843	1.57		5.9	Class A
1246	81	M	22.48133	0	1	0	0	0	1	0	0	0	0	0	0	0	1	1	0	0	0	137	125	79	73	2.85	3.16	17.47697	15.61021	9.7	10	4	3.8	89	57	21.1	12.9	26.4881	15.92179	92	181	6130	4530	3.16006	2.36549		6	Class A
1247	75	M	21.84642	0	0	1	0	1	0	0	1	0	0	0	0	0	0	0	0	1	1	145	143	84	83	2.42	2.39	21.36806	21.66167	10.1	11.1	4.4	4.1	50	51	65.4	57.3	17.48252	18.68132	768	1250	185	105	0.87391	2.53755		5.9	Class A
1248	85	F	27.43126	0	1	0	0	0	1	0	0	0	1	0	0	0	0	0	0	0	0	126	127	54	52	1.72	1.44	22.13272	26.88155	10.3	10	3.6	3.6	47	46	77.4	59.3	19.2623	21.59624	1990	1590	3210	5380	0.002089	0.33146		5.5	Class A
1249	51	M	28.05398	1	0	0	0	0	0	1	1	1	0	0	0	0	0	0	0	0	0	144	NA	82	NA	3.74	4.51	14.82544	12.07981	11.3	13.4	3.1	3	92	63	113	50.9	36.65339	25	1280	2740	389	319	2.12766	7.40664		7.7	Class B
1250	73	F	19.39619	0	0	1	0	1	0	0	0	0	0	0	0	0	0	0	0	0	1	119	150	64	75	4.74	5.64	7.6273	6.2817	8.9	9	4.53	4.04	63	60	80.7	116	21.72414	23.52941	835	13700	1150	2780	3.23846	3.91877	NA		Class A
1251	82	F	16.33579	1	0	0	0	0	1	0	0	1	1	1	1	0	1	0	1	0	0	96	141	47	66	1.91	1.33	19.94027	29.52413	8.9	10.8	2.4	2.7	54	45	120	57.4	36.9863	28.48101	1980	975	1750	4130	0.14061	1.361		7.2	Class A
1252	82	F	20.68941	0	0	1	0	1	0	0	0	0	0	0	0	0	0	0	0	1	1	121	142	77	67	2.03	1.73	18.65438	22.1436	9.6	10.7	3.4	3.8	42	69	85	96.8	20.09569	29.87013	9550	1130	1000	205	0.496	0.68868	NA		Class A
1253	71	F	21.2041	0	0	0	1	1	0	0	0	1	0	0	0	1	1	1	0	0	0	120	111	75	62	2.23	1.98	17.54242	19.97944	10	12.9	4.5	4.6	94	117	224	175	36.15385	40.34483	122	489	87	64	0.07403	0.30256	NA		Class A
1254	78	M	30.42306	0	1	0	0	0	0	0	1	0	0	0	0	0	1	1	0	1	1	92	128	55	73	4.2	NA	11.55941	NA	8.9	NA	3.7	NA	38	NA	15.3	NA	13.71841	NA	5190	NA	7880	NA	0.74176	NA		5.8	Class B
1255	66	M	20.14096	1	0	0	0	0	1	0	1	1	0	0	0	0	0	0	0	0	0	192	118	99	69	3.24	4.25	16.10855	11.97113	10	11.1	3.2	3	71	32	88.9	50.6	27.73438	13.9738	912	22700	1210	597	6.65494	3.5518		6.7	Class A
1256	76	M	23.88038	1	0	0	0	0	1	0	0	1	1	0	1	0	1	1	0	0	0	118	101	63	55	3	3.01	16.8282	16.76704	9.5	11	3.9	3.9	87	84	145	87.7	31.07143	28	177	167	606	533	0.15652	0.08333		7.5	Class A
1257	81	M	21.09731	0	0	0	1	1	0	0	1	0	0	0	0	0	0	0	0	0	0	141	133	87	88	4.53	4.56	10.52677	10.41429	9.6	10.1	4	4.1	71	70	287	262	28.28685	29.53586	1670	124	1140	2450	3.27835	0.59701	NA		Class C
1258	44	M	19.72656	0	0	1	0	0	0	0	0	0	0	0	0	0	1	1	0	0	0	NA	115	NA	75	3.88	4.09	14.85761	14.02509	9.9	10.6	3.8	4	28	108	128	124	11.29032	42.1875	8170	132	259	118	3.71429	2.08163	NA		Class B
1259	85	M	20.17325	1	0	0	0	0	0	1	1	1	1	0	0	0	0	0	0	0	1	155	134	76	78	4.5	4.35	10.45789	10.85304	10.6	10.6	4.5	4.8	47	83	10.7	31.9	13.39031	25.53846	50	54	4070	4130	0.20202	0.2635		5.7	Class A
1260	81	F	24.08136	0	1	0	0	0	1	0	1	0	0	0	0	0	1	1	0	1	1	165	131	79	67	1.31	1.41	30.22847	27.79303	10.2	12.3	3.9	4.2	83	91	216	219	31.32075	34.0824	514	465	509	339	0.11455	0.03478	NA		Class A
1261	72	M	22.11073	1	0	0	0	0	1	0	1	0	0	0	0	0	1	1	0	0	0	137	130	65	60	4.3	4.11	11.52744	12.11168	9	10.7	3.5	3.7	64	85	93.1	62	28.44444	34.55285	1490	662	930	1370	2.18	2.05		6.6	Class B
1262	79	F	NA	1	0	0	0	0	0	0	1	0	0	0	0	0	0	0	0	0	0	131	NA	59	NA	1.03	1.16	39.4653	34.65312	9.7	10.4	4	4	52	74	116	83.7	22.12766	29.95951	2160	2020	151	205	0.28302	0.22409		6.1	Class A
1263	58	F	23.44862	1	0	0	0	0	0	0	1	0	0	0	0	0	0	0	0	0	0	131	131	79	84	5.93	6.72	6.37706	5.53439	9.4	10.2	4.2	4.3	50	73	88.2	80.8	18.79699	27.54717	1660	1510	661	915	2.91525	4.50177	NA		Class C
1264	61	M	21.96206	0	0	1	0	1	0	0	1	0	0	0	0	0	0	0	0	0	0	100	118	72	78	2.84	3.22	19.03188	16.51167	10.7	11.8	4	3.2	66	65	534	350	24.81203	28.50877	1510	6260	80	56	0.25271	NA		5.8	Class A
1265	71	F	24.31031	0	1	0	0	1	0	0	1	0	0	0	0	0	1	1	0	0	1	140	140	80	86	1.45	1.63	28.09302	24.71734	10.5	11.6	4	4.1	78	102	25.3	30.2	25.24272	30.44776	92	101	204	62	1.92524	1.75097	NA		Class A
1266	74	M	21.98479	0	0	1	0	1	0	0	1	0	1	1	0	0	1	1	0	0	0	145	137	60	67	1.65	1.86	32.61405	28.60784	9.6	12.3	3.3	3.9	59	108	162	70.7	24.38017	33.75	304	188	809	802	0.50847	0.58072	NA		Class A
1267	84	F	21.45727	0	0	1	0	1	0	0	1	1	0	0	0	0	0	0	0	0	1	158	165	77	54	1.78	1.93	21.39038	19.57845	9.2	10.2	2.6	3	196	127	42.4	63.7	69.75089	44.09722	3980	1980	242	233	4.24446	1.98064		5.5	Class B
1268	72	M	25.57493	0	1	0	0	0	1	0	1	0	0	0	0	1	1	0	1	1	1	150	165	70	67	5.3	5.7	9.17043	8.43531	10.1	10.9	4.1	NA		50	NA	20.6	NA	16.50165	NA	6460	NA	3690	1.32554	1.64313	NA		Class A
1269	62	F	19.42015	0	0	0	1	0	0	1	1	0	0	0	0	0	0	0	0	0	1	155	151	87	102	2.26	1.85	17.9736	22.37401	8.1	9.2	3.7	3.5	83	130	8.6	20.6	25.15152	47.97048	2260	2880	369	538	0.78194	2.19688	NA		Class C
1270	63	M	23.4375	1	0	0	0	0	1	0	1	1	0	0	0	0	0	0	0	0	0	143	142	65	66	5.64	6.99	8.90208	7.03936	9.7	11.1	3.7	4	66	93	96.4	77.7	26.19048	34.44444	146	285	4900	5310	3.23214	2.11765		5.2	Class A
1271	78	M	25.1975	1	0	0	0	0	1	0	1	1	0	0	0	0	1	1	1	0	0	140	130	61	70	2.66	2.36	18.98289	21.638	10.7	10.7	4.4	4	73	62	29.2	38.8	23.47267	20.32787	270	897	289	268	1.42857	1.79372		6.6	Class A
1272	74	F	19.48052	0	0	0	1	1	0	0	1	0	0	0	0	0	0	0	0	0	1	150	111	70	55	2.15	2.2	18.04218	17.59407	10.2	11.1	4	3.9	67	68	22.7	60.1	26.8	30.35714	160	120	271	314	1.06304	0.51064	NA		Class A
1273	68	F	29.51389	1	0	0	0	1	0	0	1	1	0	0	1	0	1	1	0	1	1	148	158	78	72	3.82	4.02	9.85686	9.28268	8.5	12.2	3.5	3.8	59	48	91.1	75.8	24.58333	19.35484	2340	14600	3390	3960	2.09677	2.42024		6.7	Class A
1274	72	M	16.16162	0	0	1	0	1	0	0	0	0	0	0	0	0	1	1	0	0	1	96	NA	59	NA	1.21	NA	46.15089	NA	9.5	NA	2.8	NA	21	53	36.3	41.9	6.818182	17.43421	38400	20100	113	96	0.15789	NA		6.5	Class A
1275	52	F	17.59856	1	0	0	0	0	1	0	0	1	0	0	0	0	1	0	1	0	0	134	140	89	95	2.81	3.31	14.89596	12.45264	10.3	11.3	4	3.7	58	69	33	5.7	22.56809	18.54839	130	489	426	424	1.31579	NA		5.5	Class A
1276	64	M	23.072	1	0	0	0	0	1	0	1	1	0	0	0	0	1	1	0	0	0	142	135	95	NA	4.2	4.9	12.2347	10.33603	11.3	11.5	1.4	1.1	64	54	427	455	46.37681	39.41606	289	931	1090	1420	16.49555	19.88156		5	Class A
1277	69	M	16.47473	0	0	0	1	0	1	0	1	0	0	0	0	0	0	0	0	0	0	151	141	81	71	2.76	2.98	18.87574	17.35665	10.2	9.6	3.5	3.8	92	90	570	689	34.32836	35.01946	446	4930	931	769	4.3125	4.33613		5.1	Class A
1278	57	F	20.00633	0	0	1	0	1	0	0	1	0	0	0	0	0	1	1	0	0	0	143	156	87	89	2.53	2.78	16.27407	14.67997	10.7	10.5	3.4	3.7	51	81	27.7	29.8	17.28814	26.21359	645	455	276	300	NA	NA	NA		Class B
1279	69	M	17.53653	0	0	0	1	1	0	0	1	0	0	0	0	1	0	1	0	0	0	110	104	56	60	1.79	1.72	30.43903	31.6658	10.3	9.5	NA	3	75	51	202	189	29.18288	22.76786	3570	833	1160	801	0.001	0.001	NA		Class A
1280	76	F	23.29429	1	0	0	0	1	0	0	1	0	1	0	0	0	1	1	0	0	0	133	150	63	51	1.88	1.77	20.73595	22.14981	9.2	9.3	3.1	3.2	60	45	17.6	7.9	24.39024	14.90066	1300	476	3740	5890	0.95349	NA		7	Class A
1281	37	F	18.22068	0	0	1	0	1	0	0	0	0	0	0	0	0	1	1	0	0	0	114	106	59	60	2.32	2.9	20.25482	15.86751	9.5	10.7	3.5	3.6	82	94	104	50.6	31.29771	34.81481	335	340	294	181	4.71574	5.27914	NA		Class C
1282	76	M	22.53123	0	1	0	0	1	0	0	1	0	0	0	1	0	1	1	0	0	0	160	138	90	68	3.47	3.46	14.35119	14.34266	8.6	9.7	3.8	4	52	68	52	45.8	22.22222	26.77165	429	235	717	1080	2.16667	2.1	NA		Class A
1283	76	F	23.98961	0	0	1	0	1	0	0	1	1	0	0	1	1	1	0	0	0	1	118	130	68	72	0.96	0.98	43.25616	42.29133	9.3	11	3.7	3.7	129	102	179	49.1	46.07143	33.77483	1150	1530	621	1080	0.06905	0.05952		7	Class A
1284	78	M	22.7709	0	0	1	0	1	0	0	1	0	0	0	0	0	0	0	0	1	1	120	130	60	60	1.61	1.65	32.99917	32.125	10.8	11.8	3.7	4.1	91	88	51.6	70.6	32.38434	33.46008	4370	4770	310	304	0.23288	0.58788	NA		Class A
1285	87	M	21.22789	0	1	0	0	0	1	0	1	0	0	0	0	0	1	1	0	0	0	170	105	83	41	3.3	NA	14.58497	NA	9.9	NA	3.6	NA	111	78	31	31.6	40.21739	28.78229	714	3790	1120	2320	0.66144	NA		5.4	Class B
1286	80	M	22.60672	0	1	0	0	0	1	0	0	0	0	0	0	0	0	0	0	0	1	120	114	56	62	2.08	1.95	24.75447	26.56544	8.6	10.2	3.8	NA	312	109	174	109	93.9759	37.58621	1810	6110	3590	4300	NA	NA		6	Class A
1287	68	M	25.23396	1	0	0	0	0	0	1	1	1	0	0	0	1	1	0	0	0	0	190	179	74	75	3.31	3.46	15.60198	14.86355	10.8	10.8	2.9	3.2	57	77	149	81.7	22.17899	27.5	4270	1080	1080	926	6.18227	7.79221		6.2	Class C
1288	75	M	27.23922	0	1	0	0	0	1	0	1	1	0	1	0	1	1	0	0	0	0	116	105	64	67	1.74	1.63	30.65478	32.92501	10	10.7	3.9	3.8	80	73	101	90.2	24.39024	23.39744	2010	5790	1230	960	1.12727	NA		5.9	Class A
1289	53	F	17.69371	0	0	1	0	0	1	0	1	1	1	0	0	1	0	0	0	0	0	70	70	50	48	2.17	1.87	19.65586	23.13046	9.5	11.3	4.3	3.9	109	70	293	208	36.21262	27.66798	535	8510	9080	14300	0.07143	NA	NA		Class C
1290	64	M	25.77061	0	1	0	0	0	1	0	0	0	0	0	0	0	1	1	0	0	0	115	117	65	53	1.57	1.37	35.9016	41.6731	7.4	8.4	4.2	3.9	67	35	148	45.8	29.91071	14.83051	622	2610	3740	5440	0.11321	0.25		5	Class A
1291	67	M	21.46412	1	0	0	0	0	1	0	1	0	0	0	0	0	1	1	0	0	0	115	132	61	66	2.31	1.94	23.22345	28.11015	9	11	3.94	4.11	70	79	102	86.1	25.64103	25.23962	288	294	350	142	0.18401	0.77509		8.2	Class A
1292	75	F	21.91134	0	0	0	1	1	0	0	1	0	0	0	0	1	1	1	0	1	1	138	154	70	82	0.88	0.81	47.75728	52.29028	10.8	12.3	4.1	4.2	47	72	36	49.3	21.07623	33.02752	752	166	67	71	NA	0.09535		5.7	Class A
1293	84	M	24.19649	1	0	0	0	0	1	0	1	1	0	0	0	1	1	0	1	0	0	94	NA	60	NA	2.59	NA	19.20364	NA	10.3	NA	3.6	NA	54	NA	45.1	NA	17.47573	NA	1140	NA	400	NA	0.03916	NA		6.5	Class A
1294	72	F	19.97316	0	1	0	0	1	0	0	0	1	1	0	0	0	0	0	0	1	1	92	125	61	89	1.7	1.02	23.51152	41.11338	8.2	11	3.8	3.6	69	43	69.5	79.7	25.55556	19.11111	235	4820	2190	9780	0.001292	NA	NA		Class A
1295	75	F	28.65014	0	0	1	0	1	0	0	1	0	0	0	0	0	1	1	0	0	0	131	109	75	68	1.17	1.07	34.97102	38.56184	10.7	9	3.3	2.9	36	32	70.6	150	11.92053	13.06122	30400	64500	138	328	0.75107	0.30896		6.4	Class A
1296	80	M	25.31229	0	0	0	1	0	1	0	1	1	0	1	1	0	0	0	0	1	1	90	132	46	80	5.07	2.92	9.33977	17.01914	8.6	7.1	4.1	1.7	18	22	23.3	119	5.901639	20.37037	2830	84200	17000	28900	4.38152	2.07469		6.9	Class A
1297	71	M	19.86806	1	0	0	0	0	1	0	1	1	0	0	0	0	0	0	0	0	0	144	138	77	75	1.1	1.2	51.42887	46.75912	10.4	10.6	4	3.6	74	84	244	195	31.62393	39.43662	2060	499	445	288	0.18868	0.09091		7.1	Class A
1298	68	M	21.91659	0	0	1	0	0	1	0	1	0	0	0	0	0	0	0	0	0	0	142	164	64	70	1.29	1.29	43.74078	43.74078	9.9	11	3.3	NA	20	NA	133	NA	8.928571	NA	31400	NA	182	NA	0.08373	0.09259	NA		Class A
1299	77	F	24.17714	0	0	0	1	1	0	0	0	0	1	0	0	0	0	0	0	0	0	128	128	81	81	3.01	2.96	12.34445	12.57275	9.6	11.6	3.2	3.4	61	64	362	176	25.95745	24.90272	12300	4830	741	405	0.52778	0.32877		5.9	Class A
1300	50	F	33.9624	0	0	0	1	1	0	0	1	0	0	0	0	0	1	1	0	0	0	128	136	92	80	4.47	4.52	9.06578	8.95613	10.4	10.6	3.78	3.61	73	51	114	88	31.46552	22.17391	7890	11000	204	249	3.48571	4.48571	NA		Class A
1301	70	M	25.7367	0	0	0	1	0	1	0	1	0	0	0	0	0	1	1	0	0	0	143	148	70	81	4.75	4.86	10.42211	10.16432	9.2	11.7	4	3.9	70	89	221	88.6	27.55906	33.58491	1640	1130	436	671	1.87429	2.23971	NA		Class A
1302	80	F	NA	0	0	1	0	0	0	1	0	0	0	0	0	0	1	1	0	0	0	NA	NA	NA	NA	3.2	4.13	11.41893	8.60719	8.9	10.5	4	4	68	78	230	163	27.2	30.46875	2080	862	1070	693	1.70341	0.66384	NA		Class C
1303	76	M	22.30815	0	1	0	0	1	0	0	0	0	0	0	0	0	1	1	0	0	0	130	134	72	76	2.41	2.49	21.38364	20.55591	10.1	11.2	NA	NA	104	140	32.3	44.4	33.54839	42.81346	1420	1060	159	202	1.46769	1.76118	NA		Class B
1304	76	M	26.47032	0	1	0	0	0	1	0	0	0	0	0	0	0	1	1	0	0	1	123	140	60	68	1.94	1.23	27.11147	44.63258	10.4	10.8	4.2	4.2	115	90	31.4	34.9	32.03343	27.02703	939	862	216	220	0.000466	0.000717	NA		Class A
1305	61	F	32.364	1	0	0	0	0	0	1	0	1	0	0	0	0	1	1	0	0	0	132	130	65	67	2.81	3.29	14.22892	11.97414	9.2	9.6	3.9	3.6	56	48	140	174	19.44444	17.58242	554	13800	175	122	3.32877	2.13333		8.4	Class A
1306	50	M	28.29141	1	0	0	0	1	0	0	1	1	0	0	0	0	1	1	0	0	0	146	148	94	94	5.27	6.75	10.24558	7.81518	9.4	10.7	2.7	2.4	82	95	356	370	29.60289	40.42553	727	7830	18300	18800	13.71622	24.03825		5.2	Class B
1307	77	M	20.08628	0	0	1	0	1	0	0	0	0	0	0	0	0	1	1	0	0	1	114	103	70	63	1.72	1.64	30.81135	32.45934	10.4	10.9	3.9	4	61	100	28.2	57.5	21.03448	33.78378	50	50	132	119	0.08333	0.04762	NA		Class A
1308	44	M	25.54539	1	0	0	0	1	0	0	1	0	0	0	0	0	1	0	1	1	1	118	113	68	66	2.7	2.89	22.09118	20.37546	10	11.6	3.7	3.7	73	67	299	284	28.96825	26.5873	395	756	182	154	0.83651	2.18423		6.3	Class B
1309	66	M	20.50013	0	0	0	1	0	1	0	0	1	0	0	0	0	0	0	0	0	0	100	96	58	60	1.51	NA	37.13572	NA	10.9	NA	4.2	NA	77	NA	106	NA	31.17409	NA	1450	NA	309	NA	0.000355	NA			Class A
1310	81	F	23.55879	0	0	1	0	1	0	0	1	0	0	0	0	0	1	1	0	0	1	117	136	69	80	1.95	1.74	19.56199	22.159	10.1	12.6	3.9	4.5	42	60	13.3	46.7	13.77049	20.13423	285	523	103	110	0.03937	0.04473	NA		Class A
1311	80	F	25.66097	0	1	0	0	1	0	0	1	0	0	0	0	0	1	1	0	0	0	150	151	73	83	1.88	1.73	20.43293	22.37881	9.8	11	4	4.1	74	63	79.5	69.9	25.42955	23.33333	355	1980	761	642	1.61157	1.36986	NA		Class A
1312	45	M	23.14815	0	0	1	0	0	0	0	1	1	0	0	0	0	1	1	0	0	0	170	130	100	80	2.33	2.54	25.78947	23.46614	9.2	12.8	1.9	1.8	91	64	278	193	59.47712	40.76433	261	188	1140	823	4.56436	4.53184		5.2	Class A
1313	75	F	26.6655	0	0	1	0	1	0	0	0	0	0	0	0	0	1	1	0	0	0	139	140	72	70	2.63	2.35	14.4169	16.24443	9.2	9.9	4.2	4.1	83	101	111	119	33.2	38.40304	895	966	903	610	1.27077	2.76762		5.3	Class A
1314	74	M	21.70084	0	0	1	0	0	1	0	0	1	0	1	0	0	0	0	0	0	0	106	120	60	70	4.16	4.39	11.85889	11.18088	9.8	11.8	3.9	4.2	78	76	767	795	36.11111	35.02304	640	5590	1090	1260	1	0.20067		5.6	Class A
1315	69	F	22.31067	1	0	0	0	1	0	0	1	1	0	0	0	0	1	1	0	1	1	149	162	55	49	3.31	3.25	11.48166	11.66548	8.5	8.8	4.05	4.17	70	57	732	737	27.23735	25.22124	114	182	1760	2270	2.0598	2.53179		4.1	Class A
1316	74	M	24.87547	0	0	0	1	0	1	0	0	0	1	0	0	0	1	1	1	0	0	133	127	56	59	2.72	NA	18.87617	NA	10	NA	4	NA	70	68	157	128	27.23735	25.75758	2270	464	198	124	0.94737	NA		5.6	Class A
1317	70	F	27.84966	0	1	0	0	1	0	0	0	0	0	0	1	0	1	1	0	1	1	130	130	75	70	1.71	NA	23.55078	NA	10.5	NA	4.4	NA	64	72	240	178	24.61538	29.62963	1320	299	207	279	0.44474	NA		5.4	Class A
1318	79	M	NA	0	1	0	0	0	1	0	0	0	0	0	0	0	1	1	0	0	0	125	163	70	82	2.65	4.47	19.06127	10.75837	9.4	8.7	3.2	2.6	65	66	137	139	26.10442	29.72973	4470	2930	968	3980	3.27835	10.70667	NA		Class C
1319	86	F	19.90504	0	0	0	1	1	0	0	1	1	0	0	0	0	0	0	0	0	0	149	148	70	65	1.66	1.42	22.93226	27.20456	10.6	9.7	2.5	2.2	73	25	95.1	112	32.44444	13.81215	2240	19800	2450	3790	9.39885	NA		6.8	Class B
1320	64	F	24.16554	1	0	0	0	1	0	0	1	0	0	1	0	1	0	1	0	0	0	108	149	62	75	1.84	2.07	22.30292	19.60654	8.3	9.7	1.5	2.3	22	34	409	219	14.19355	17.98942	32200	1190	4040	3120	12.90766	13.83912		5.9	Class B
1321	67	M	23.88038	0	0	1	0	1	0	0	0	0	0	0	0	0	0	0	0	1	1	122	126	73	80	1.12	NA	51.27123	NA	10.4	NA	3.7	NA	23	58	6.7	13.5	5.437352	16.11111	1720	7820	50	39	0.02128	NA	NA		Class A
1322	61	M	NA	1	0	0	0	0	1	0	1	1	0	0	1	0	0	0	0	0	0	142	140	60	72	4.97	5.32	10.31806	9.57778	9.1	11	1.5	2	13	77	126	46.6	9.774436	41.62162	102000	476	1230	679	8.52968	11.28638		5.4	Class B
1323	66	M	22.17971	1	0	0	0	0	1	0	1	0	0	0	0	0	1	1	0	0	0	116	118	63	66	4.46	5.31	11.35587	9.38295	9.2	12.1	2.8	3.2	59	67	206	61.3	27.69953	27.68595	64	50	532	185	4.43165	3.79668		6.1	Class B
1324	46	F	18.03745	0	0	0	1	0	1	0	0	0	0	0	0	0	0	0	0	0	1	109	104	54	53	3.19	3.58	13.43036	11.83823	10.2	11.7	3.8	4.2	41	81	42.1	33.5	14.33566	25.71429	6780	1320	417	259	0.82258	1.02817		5.2	Class A
1325	74	M	21.92886	1	0	0	0	0	1	0	0	1	0	0	0	0	1	1	0	0	0	125	152	62	79	2.6	1.6	19.83131	33.73067	9.5	14.3	3	3.9	118	130	1380	74.5	49.16667	41.53355	1190	451	584	362	0.15	0.15786		8.3	Class A
1326	65	M	16.89624	0	0	0	1	1	0	0	1	0	0	0	0	0	1	1	0	0	1	164	153	102	108	2.1	2.41	26.00086	22.365	10.8	12.3	4.1	4.1	73	47	10.9	39	21.47059	17.603	50	73	190	254	2.17476	1.48848	NA		Class B
1327	77	M	22.95436	0	1	0	0	0	1	0	1	0	0	0	0	0	0	0	0	0	0	152	150	80	82	3.92	5.42	12.51192	8.74531	9.2	9.2	2.8	3.8	77	87	140	211	33.9207	37.99127	131	302	5450	11300	4.47	10.78788	NA		Class C
1328	66	M	27.28744	0	1	0	0	0	1	0	1	0	0	0	0	0	1	1	0	0	0	170	139	93	81	4.42	4.18	11.41895	12.13812	9.4	10.7	4.3	4.1	73	76	61.9	53.6	24.09241	26.29758	536	1630	290	170	1.81902	3.09244	NA		Class B
1329	68	M	26.83518	0	0	0	1	0	0	1	0	1	0	0	0	0	1	0	1	0	0	134	142	67	75	3.46	2.57	14.86355	20.57808	9.9	12	4.2	3.9	113	115	188	44.6	37.91946	35.06098	306	3970	378	239	NA	NA		7	Class A
1330	81	M	25.39343	0	1	0	0	0	1	0	0	0	1	0	0	0	1	0	1	0	1	92	NA	50	NA	2.34	NA	21.68425	NA	9.3	NA	3.8	NA	73	NA	11.7	NA	21.98795	NA	3390	NA	344	NA	0.15484	NA		5.7	Class A
1331	63	M	20.76125	0	0	1	0	1	0	0	0	0	0	0	0	0	0	0	0	0	0	57	80	31	54	2.15	2.04	25.56839	27.08043	10.2	11.1	4.1	3.9	75	54	123	52.6	31.38075	19.35484	205	6750	122	250	0.34602	0.2005	NA		Class A
1332	56	M	28.41706	1	0	0	0	0	1	0	1	1	0	0	1	0	1	1	0	0	0	154	124	73	63	2.85	2.89	19.42994	19.13593	10.2	10.7	3.3	3	95	75	203	139	35.05535	29.29688	148	424	595	1710	4.30387	0.01969		6.6	Class A
1333	80	F	21.51925	0	0	0	1	1	0	0	1	0	0	0	0	0	1	1	0	0	0	122	144	67	78	1.66	1.57	23.41322	24.79686	8.8	11	3.9	3.7	46	67	385	502	27.05882	40.60606	918	2770	925	860	2.78938	10.75544		5.7	Class A
1334	65	F	22.26792	0	0	0	1	0	1	0	1	0	1	0	0	0	1	1	0	1	1	140	197	85	90	2.27	2.06	17.64606	19.53738	7.7	10.7	3.5	3.7	33	34	37.9	53	9.322034	10.42945	2740	619	1920	721	0.76923	0.88496		5	Class A
1335	76	M	25.25209	0	0	0	1	1	0	0	1	0	0	0	0	0	0	0	0	1	1	188	155	76	68	4.75	5.8	10.17901	8.18122	9	9.7	3.4	3.5	48	48	34.5	31.1	20.08368	19.75309	421	542	1980	1400	NA	NA		5.9	Class A
1336	55	M	22.02722	0	0	1	0	0	1	0	0	0	0	0	1	0	0	0	0	0	0	93	116	71	80	2.39	2.38	23.67829	23.78715	8.7	9.1	3.3	3.7	35	71	308	336	13.94422	25.08834	29400	7380	538	692	0.47729	0.36941	NA		Class A

1337	74	M	21.41746	0	0	0	1	0	1	0	1	0	0	0	0	0	0	0	0	0	0	145	143	56	67	3.69	4.7	13.52089	10.37665	10.5	11.1	3.3	2.8	73	95	62.6	70.7	31.46552	44.39252	957	3060	931	1410	7.84936	9.01045	5.9	Class B
1338	71	M	24.90519	1	0	0	0	0	0	1	0	1	0	0	0	0	1	1	0	0	1	136	108	84	63	2.5	3.49	20.94808	14.54252	9.9	12	3.6	3.7	88	75	259	95.6	34.92063	28.51711	1290	737	403	342	4.12458	3.72919	7.1	Class A
1339	51	M	23.95188	1	0	0	0	0	1	0	1	1	0	0	0	0	1	1	0	0	0	154	146	82	72	6.14	6.86	8.61932	7.63467	10	11	4.2	3.9	83	71	131	130	37.72727	33.64929	79	7860	480	881	0.32841	0.77273	5.6	Class A
1340	68	M	21.61281	1	0	0	0	0	0	1	0	1	0	0	0	1	1	0	1	0	1	120	NA	60	NA	2.2	NA	24.39278	NA	7.9	NA	2.8	NA	25	53	49.7	22.5	13.08901	20.07576	4030	1100	504	564	0.78165	NA	8.6	Class A
1341	57	F	18.56261	0	0	1	0	1	0	0	1	0	1	0	0	0	1	0	1	0	0	151	121	102	81	4.44	NA	8.79575	NA	10.9	NA	3.5	NA	65	NA	75.3	NA	32.33831	NA	290	NA	645	NA	1.43902	NA	NA	Class A
1342	64	F	18.84444	1	0	0	0	0	1	0	0	0	1	0	0	0	1	1	0	1	1	100	110	60	50	0.91	0.96	48.1816	45.44308	9.6	10.8	4.2	4.1	88	68	46.3	47.1	30.76923	24.81752	62	55	86	142	0.11782	0.08743	7.3	Class A
1343	81	F	30.76438	0	1	0	0	0	0	1	0	0	1	1	1	1	0	0	0	0	0	114	114	44	44	2.82	2.46	13.06587	15.17148	10.7	10.2	4	3.9	45	NA	16.1	NA	10.61321	NA	1530	NA	502	NA	0.11527	NA	6.5	Class A
1344	70	F	23.99594	0	0	1	0	1	0	0	0	0	0	0	0	0	1	1	0	0	0	138	137	75	78	2.13	2.14	18.52063	18.42597	9.3	11.7	4.5	4.6	71	78	55.1	14	27.51938	23.78049	50	50	114	96	0.6	2	NA	Class A
1345	86	F	29.52622	0	1	0	0	1	0	0	0	0	0	0	0	0	0	0	0	0	0	139	176	86	86	1.86	2.26	20.24874	16.36253	10	12.2	2.5	2.3	69	58	83.6	47.1	29.87013	24.8927	1680	1200	506	931	13.22581	13.02839	5.6	Class A
1346	68	F	NA	0	0	1	0	1	0	0	0	0	0	0	0	0	0	0	0	0	0	129	121	74	71	2.04	2.17	19.57856	18.29908	9.7	9.7	4	NA	64	51	6.4	5.7	17.77778	13.45646	573	462	120	101	0.34118	0.40351	5.9	Class A
1347	66	M	24.07044	0	0	1	0	1	0	0	0	0	0	0	0	0	1	1	0	0	0	NA	NA	NA	NA	3.06	4.39	17.148	11.55411	9.7	12.1	3.5	3.6	49	71	169	117	24.37811	28.28685	606	390	724	152	0.58763	0.51389	5.6	Class A
1348	82	M	28.72288	1	0	0	0	1	0	0	1	1	0	1	0	0	1	1	0	1	1	171	137	63	64	2.24	2.61	22.66553	19.17487	8.9	11.4	2.8	3.8	24	51	71.7	41.8	14.11765	24.63768	3200	17300	8820	1310	0.76923	0.98558	8.7	Class B
1349	75	M	22.90047	0	0	0	1	0	1	0	0	0	0	1	1	1	0	0	0	0	0	118	133	63	67	1.48	NA	36.59256	NA	10.6	NA	3.6	NA	95	NA	53.6	NA	34.17266	NA	2700	NA	222	NA	2.47273	NA	6.1	Class A
1350	77	M	22.68118	0	0	0	1	1	0	0	0	0	0	0	0	0	1	1	0	0	0	127	138	70	81	3.02	2.79	16.64375	18.15047	10.6	11.6	3.9	4	40	NA	21.9	NA	13.51351	NA	683	NA	715	NA	0.30818	0.40244	NA	Class A
1351	58	F	NA	0	0	0	1	0	0	0	0	1	0	0	0	0	1	1	0	0	0	132	148	78	76	2.19	3.03	18.96254	13.29365	9.2	8.9	3.1	3.1	NA	43	NA	45.7	NA	17.7686	NA	8460	NA	1030	5.01724	5.61111	6.5	Class A
1352	68	M	17.82171	0	0	0	1	1	0	0	0	0	0	0	0	0	1	0	1	0	0	130	128	68	72	2.82	2.93	18.59085	17.82865	10.5	12.1	4.32	3.91	86	98	278	177	29.05405	36.70412	118	1260	360	314	0.79548	4.20386	NA	Class A
1353	79	M	20.66932	0	0	0	1	0	0	1	1	0	0	0	0	0	1	1	0	0	0	151	141	64	80	5.63	9.67	8.35847	4.62515	8.7	10	NA	2.8	89	83	170	131	37.23849	38.60465	50	195	504	2370	4	11.14315	NA	Class C
1354	85	M	19.15709	0	0	0	1	1	0	0	0	1	0	0	0	1	0	0	0	0	0	111	122	63	80	1.17	1.09	45.65235	49.33032	9.8	12	3.77	4.11	40	49	198	117	22.22222	23.00469	421	891	211	168	0.1109	0.3173	NA	Class A
1355	58	M	22.09085	0	0	1	0	0	1	0	0	0	0	0	0	0	1	1	0	0	0	110	122	70	90	3.81	4.04	14.00122	13.0673	9.8	12.9	3.7	3.7	71	95	128	85.7	26.10294	30.35144	63	79	144	150	1.70953	1.91176	NA	Class B
1356	85	M	21.38101	0	0	0	1	1	0	0	0	0	0	0	0	0	1	1	0	0	0	138	146	70	72	2.15	2.38	23.46231	20.99343	8.4	9.5	3.3	3.3	73	47	60.6	32.6	33.33333	20.88889	1310	2860	602	467	1.70564	2.68893	NA	Class B
1357	64	M	22.11085	0	0	1	0	1	0	0	0	1	0	0	0	0	1	1	0	1	1	134	112	87	72	2.05	2.26	26.81448	24.10091	10	9.3	3.2	2.8	120	91	109	87.3	40	29.26045	5610	6480	180	148	0.04545	NA	NA	Class A
1358	55	M	25.94447	0	0	0	1	0	1	0	0	1	0	0	0	1	1	1	0	0	1	113	112	71	66	2.96	3.06	18.6414	17.97598	11.4	13.5	4.5	4.3	99	102	44.9	26.6	34.02062	33.66337	112	354	32	36	0.65134	0.64782	6.4	Class A
1359	84	F	25.7986	1	0	0	0	0	1	0	0	1	0	0	0	0	1	0	1	0	0	115	149	48	69	1.92	1.73	19.69003	22.06762	10.7	14.2	3.5	4	66	93	230	71.8	29.59641	30	588	970	259	216	0.09259	0.85075	6.1	Class A
1360	72	F	23.73996	0	0	1	0	1	0	0	0	0	0	0	0	0	0	0	0	0	0	124	139	70	66	3.05	3.06	12.40417	12.35983	9	11.6	4	4	69	89	109	53.8	29.48718	33.71212	834	321	239	243	0.41791	0.42506	NA	Class A
1361	74	F	25.23926	0	1	0	0	1	0	0	1	1	0	0	0	0	1	1	0	0	0	172	135	104	75	1.42	1.29	28.40359	31.54943	10.3	11.2	3.9	3.6	70	70	62.8	56.3	20.11494	21.21212	1100	987	186	144	0.001953	0.10337	6.8	Class A
1362	74	M	30.44872	0	0	1	0	0	1	0	0	1	0	0	0	0	0	0	0	0	0	138	138	64	64	4.81	4.8	10.07842	10.10139	9.8	9.9	3.2	3.5	52	NA	364	NA	30.58824	NA	46600	NA	1100	NA	2.74499	5.2611	5.5	Class B
1363	80	M	17.81451	0	0	1	0	1	0	0	0	1	0	0	0	0	0	0	0	0	1	127	134	67	67	1.76	1.41	29.71831	37.87644	11	13.5	3.4	3.5	63	105	20.2	30	18.91892	31.34328	2490	3140	245	188	0.08651	5.79545	9.4	Class A
1364	80	F	25.42222	0	1	0	0	1	0	0	1	0	0	0	0	0	1	1	0	0	0	147	123	80	70	1	1	40.76236	40.76236	10.6	11.7	4.2	4.3	64	84	143	111	22.06897	32.18391	388	388	118	99	0.11321	0.08333	NA	Class A
1365	66	M	20.89844	0	0	0	1	0	1	0	0	0	0	0	0	0	0	0	0	0	1	124	134	72	74	4.18	4.45	12.19062	11.38379	9	11.9	4	4.1	57	109	61	30.1	25.22124	36.45485	697	95	1070	594	0.52113	0.69575	NA	Class A
1366	68	F	23.30905	0	0	0	1	0	1	0	1	0	0	0	0	0	1	1	0	0	0	164	NA	94	NA	2.96	3.32	13.02936	11.44383	9.8	10.7	3.5	3.8	91	NA	35.1	NA	38.88889	NA	225	NA	1030	NA	NA	NA	5.2	Class C
1367	58	F	21.88708	0	1	0	0	0	1	0	0	0	0	0	0	0	0	0	0	1	1	120	101	67	50	1.93	1.86	29.46444	30.52952	9.2	9.1	3.2	3.1	37	22	33.7	31.2	13.0742	7.857143	534	2430	263	346	0.72807	0.90071	NA	Class A
1368	72	M	20.7425	0	0	0	1	0	1	0	1	0	0	0	0	0	1	1	0	0	0	142	126	80	80	3.85	2.91	13.00928	17.60067	10.2	12.3	4.2	3.7	87	99	148	78.4	32.95455	34.98233	161	165	327	336	1.06615	4.2401	6.3	Class B
1369	66	F	24.1566	0	0	0	1	0	1	0	0	0	0	0	0	0	0	0	0	0	1	95	105	42	51	1.73	1.47	23.6491	28.26128	6.3	8.3	3.5	3.6	30	112	12.2	37.9	8.982036	40.87591	197	212	124	134	0.16056	0.1302	5.4	Class B
1370	74	F	21.6162	0	1	0	0	0	1	0	0	0	0	0	0	0	0	0	0	0	0	139	129	87	70	2.38	2.29	16.14364	16.83901	10.6	13.3	4.3	4.2	83	90	178	103	23.05556	22.72727	114	103	299	182	0.1278	0.07488	NA	Class A
1371	73	F	27.59024	1	0	0	0	0	0	1	1	1	0	0	0	0	0	0	0	0	0	147	85	52	32	2.5	NA	15.3577	NA	8.9	NA	3.5	NA	62	65	27.8	25.7	18.73112	20.50473	80	205	711	618	4.04082	NA	6	Class B
1372	75	M	25.51806	1	0	0	0	0	1	0	0	1	1	0	0	0	1	1	0	0	0	138	128	68	67	3.56	3.85	14.00789	12.85775	10.5	11.7	4.2	3.9	41	81	216	171	17.44681	32.92683	546	52	155	170	3.42424	4.54054	5.9	Class A
1373	83	F	24	1	0	0	0	1	0	0	0	1	0	0	0	0	1	1	0	0	1	134	128	70	80	0.99	0.84	40.77987	48.81005	9.8	12.8	4.3	4.1	70	70	70.7	85.9	25	25.08961	425	10400	1030	395	0.5	0.49133	6.3	Class A
1374	70	M	NA	0	1	0	0	0	1	0	0	1	1	0	1	0	1	0	1	0	1	120	120	70	65	3.32	3.81	15.42175	13.26559	11	12.2	4.6	4.5	62	61	14.3	29.9	17.22222	17.9941	687	744	4200	6740	0.30769	0.40506	5.4	Class A
1375	73	M	28.90625	1	0	0	0	0	1	0	0	1	1	0	0	1	1	0	1	0	0	131	124	67	54	2.14	1.83	24.63516	29.23523	10.3	11.1	3.8	3.9	78	70	136	138	27.85714	23.89078	1390	1760	929	1180	0.15952	0.2402	7.5	Class A
1376	82	M	21.26579	0	1	0	0	0	1	0	1	1	0	0	0	0	0	0	0	0	0	141	150	91	83	1.95	1.97	26.37784	26.08502	10.2	11.4	3.8	4.1	89	102	193	218	38.03419	41.12903	2150	1100	358	222	1.09311	1.11457	6	Class A
1377	68	M	23.83673	0	0	1	0	0	1	0	0	0	0	0	1	0	1	1	0	0	1	120	136	70	83	2	1.85	27.07353	29.48396	10	12.5	3.7	3.8	28	102	6.8	22.9	7.446809	31.28834	144	147	652	443	0.85137	1.40165	5.1	Class A
1378	92	M	20.9375	0	0	0	1	0	0	1	0	0	1	0	0	0	1	1	0	0	1	115	104	62	56	2.01	2.03	24.6887	24.42272	10.3	11.3	3.5	3.6	91	111	12.7	32.1	30.95238	38.54167	437	318	289	372	0.80303	0.89474	NA	Class A
1379	55	F	20.49854	0	0	1	0	0	1	0	0	0	0	0	0	0	1	0	1	0	0	130	101	55	52	0.85	0.94	54.22228	48.56912	8.9	8.7	3.7	3.8	29	38	4.8	4.6	7.073171	9.245742	727	873	93	100	0.10858	0.09036	5.7	Class A
1380	80	F	24.41589	0	0	1	0	1	0	0	0	0	0	0	0	0	0	0	0	0	0	124	114	69	69	1.28	1.28	31.11513	31.00439	10.8	11.7	3.8	3.5	142	47	14.6	22.8	46.1039	17.15328	3480	27400	43	32	17.9	0.06873	5.4	Class A
1381	71	M	19.43886	0	0	0	1	0	1	0	1	0	0	0	0	1	0	1	0	0	0	144	123	69	72	3.18	3.36	16.10035	15.15917	10.1	10.6	3.4	3.4	102	131	166	173	39.84375	53.68852	115	162	1200	1370	1.10828	1.52252	4.8	Class B
1382	85	F	22.51218	0	0	0	1	1	0	0	0	0	0	0	0	0	0	0	0	0	0	NA	NA	NA	NA	2.57	3.18	14.26383	11.2992	8.2	9.1	3.1	NA	35	NA	832	NA	23.80952	NA	21000	NA	12800	NA	0.65517	NA	NA	Class B
1383	61	F	18.7107	0	0	0	1	1	0	0	0	0	0	0	0	0	0	0	0	0	0	119	147	74	94	4.11	3.88	9.38671	9.99711	9.9	10.3	4.1	4.1	58	74	52.6	54.5	23.01587	27.71536	50	56	569	374	NA	NA	NA	Class A
1384	83	M	15.3356	0	0	0	1	1	0	0	0	1	0	0	0	0	1	1	0	0	0	126	139	49	57	1.38	1.36	38.3706	38.85456	10.2	12	3.5	3.8	65	65	135	98.6	25.89641	22.0339	1980	1210	321	204	0.37247	0.46684	6.6	Class A
1385	66	M	21.875	0	0	1	0	1	0	0	0	1	0	0	0	0	1	1	0	1	1	103	118	59	70	2.61	2.8	20.40741	18.816	10.9	13.3	3.9	3.8	28	44	10	21.4	9.302326	16.11722	679	1590	331	673	1.0929	1.875	NA	Class A
1386	87	F	20.76257	0	1	0	0	1	0	0	0	0	0	0	0	0	0	0	0	0	1	122	124	60	64	1.38	1.12	27.97541	35.15278	10	11.5	3.9	3.8	57	61	43.8	76.6	20.57762	25.95745	295	605	191	305	0.12168	0.24709	NA	Class A
1387	80	M	22.47078	0	1	0	0	0	1	0	0	0	1	0	0	0	0	0	0	0	0	132	158	72	80	3.66	6.75	13.34033	6.82899	9.9	10.9	3.5	3.9	81	95	153	135	34.91379	35.58052	295	549	917	1700	4.03461	6.74825	5.6	Class B
1388	66	F	29.87851	1	0	0	0	1	0	0	0	1	0	0	0	0	1	1	0	0	0	134	146	77	81	2.77	2.48	14.13072	15.87934	9.3	11.1	3.4	3.4	58	56	165	58.3	23.86831	23.62869	1000	925	469	331	4.55556	5.42529	6.5	Class A
1389	57	M	NA	1	0	0	0	0	0	0	1	1	0	0	0	0	0	0	0	0	0	146	156	80	84	4.21	4.56	12.6154	11.56001	10.5	12.4	4	4.1	36	79	74.2	51.3	14.4	28.72727	8970	750	236	144	0.75325	1.34118	NA	Class A
1390	75	M	27.35863	0	1	0	0	0	0	1	0	0	0	0	0	0	1	1	0	0	0	146	136	70	66	3	2.92	16.89229	17.39924	10.8	10.9	3.8	3.8	NA	NA	NA	NA	NA	NA	NA	NA	NA	NA	NA	0.74074	NA	Class A
1391	79	F	23.8054	1	0	0	0	1	0	0	1	1	0	0	1	0	1	1	0	0	0	145	156	43	51	2.72	3.26	13.69017	11.22967	9.3	9.1	3.6	3.3	49	58	51.5	64.1	21.97309	26.97674	1520	5170	4280	9500	8.35648	15.74176	5.9	Class C
1392	73	F	22.82688	0	0	1	0	1	0	0	1	0	0	0	0	0	1	1	0	0	0	147	131	78	64	1.65	1.56	24.19608	25.7273	9.5	9.4	3.9	3.9	31	48	5.1	4.4	7.888041	12.69841	232	129	295	482	0.04225	0.06557	6.1	Class A
1393	82	F	20.73688	0	1	0	0	1	0	0	1	0	0	0	0	0	1	1	0	1	1	162	127	84	54	1.74	1.42	22.0811	27.57898	8.3	11.4	4.5	NA	61	74	33.5	37.4	21.32867	24.58472	157	108	2240	2780	0.12587	0.17112	NA	Class B
1394	73	M	22.68731	0	1	0	0	0	1	0	0	0	0	0	0	0	1	1	0	0	0	107	134	61	72	2.85	2.68	18.00644	19.25966	8.9	11.3	4	3.7	64	87	174	113	29.3578	36.55462	197	580	216	243	0.45714	1.28947	NA	Class A
1395	68	F	NA	0	1	0	0	1	0	0	0	0	0	1	0	1	1	1	1	0	0	124	150	52	80	3.81	4.29	9.88517	8.68176	9.5	9.4	3.4	3.5	95	42	252	154	35.18519	18.26087	497	2110	8190	7920	1.31774	2.77826	NA	Class A
1396	60	M	21.61281	0	0	1	0	0	1	0	0	0	0	0	0	0	1	1	0	0	0	128	127	70	74	1.95	1.7	28.8519	33.52441	10.3	11.6	3.9	3.9	75	67	114	84.3	28.40909	25.57252	735	329	852	809	0.71486	0.67383	NA	Class A
1397	66	M	21.03806	1	0	0	0	0	1	0	1	1	0	0	0	0	1	1	0	0	0	174	182	101	88	2.08	4.39	26.15961	11.55411	9.6	9	3.52	3.42	69	37	692	265	26.33588	16.74208	400	636	2000	9200	9.78102	7.44235	5.6	Class C
1398	77	M	19.87395	1	0	0	0	1	0	0	1	1	0	0	0	0	1	1	0	0	0	139	158	70	77	3.7	4.33	13.32804	11.22176	9.8	10.2	3.5	3.4	63	70	45.4	67	20.79208	22.95082	730	2680	4200	3440	1.73925	2.59606	5.9	Class C
1399	71	M	22.14533	0	0	0	1	0	1	0	0	0	0	0	0	0	0	0	0	0	0	133	140	68	73	6.12	6.3	7.86655	7.621	10.6	11.3	4.3	4.2	68	99	103	68.2	22.51656	31.8328	199	205	668	213	1.43102	1.76073	NA	Class A
1400	44	M	19.29858	0	0	1	0	1	0	0	1	0	0	0	0	0	0	0	0	0	0	147	125	89	91	3.72	4.62	15.55811	12.27475	10	10.9	4.2	4.3	62	14	57.2	83.4	27.43363	6.511628	422	23000	472	293	0.3352	0.21322	NA	Class C
1401	79	F	17.45756	0	1	0	0	0	0	1	1	0	0	0	0	0	1	1	0	0	0	147	196	60	90	2.52	3.04	14.88315	12.1217	8.7	8.4	4.5	4.5	66	60	11	9	17.36842	13.98601	261	270	1610	1660	3.35714	3.48	NA	Class C
1402	81	F	21.84275	1	0	0	0	1	0	0	0	1	0	0	0	0	0	0	0	0	0	112	131	62	69	0.84	NA	49.15293	NA	8.6	NA	2.6	NA	15	24	184	92	7.009346	9.561753	106000	82500	771	525	0.39359	NA	6.4	Class A
1403	72	F	NA	0	0	1	0	1	0	0	0	0	0	0	0	0	1	1	0	0	0	NA	142	NA	74	4.92	5.7	7.3516	6.25842	8.9	9.5	4.2	4.1	106	95	152	93.5	36.17747	32.53425	236	312	225	198	NA	NA	NA	Class A
1404	61	M	NA	0	1	0	0	0	1	0	1	0	0	0	0	0	0	0	0	0	1	159	166	95	95	5.89	5.3	8.52863	9.57255	10.7	13	4	4.2	34	67	155	92.9	18.99441	34.53608	18600	361	242	145	NA	NA	NA	Class A
1405	69	F	19.319	0	0	0	1	1	0	0	1	0	0	0	0	0	1	1	0	1	1	136	142	72	70	0.9	0.99	47.72602	42.8231	10.8	11.5	3.8	3.9	70	95	70.7	41.5	25.83026	30.94463	156	115	472	406	0.20915	0.22302	NA	Class A
1406	62	F	22.04278	0	0	1	0	1	0	0	0	0	0	0	0	0	1	1	0	0	0	135	134	63	72	1.54	1.24	27.34526	34.65983	9.6	9.6	2.8	2	65	36	98.5	102	29.27928	19.25134	1390	88	399	474	1.68254	3.70968	5.5	Class A
1407	78	F	29.80621	1	0	0	0	1	0	0	0	1	0	0	0	0	1	1	1	0	1	126	113	41	59	2.27	3.56	16.74645	10.23596	10.6	11.9	3.8	4	29	114	22.9	28.4	9.863946	36.07595	43100	8230	6160	1610	0.89445	0.07593	9.2	Class A
1408	72	M	20.85587	1	0	0	0	0	0	1	1	1	0	0	1	0	1	1	0	0	0	140	128	58	71	4.02	3.93	12.40863	12.71985	9.3	11.1	4	3.6	59	58	130	44.4	21.53285	22.56809	361	450	237	430	3.41808	3.80336	6.1	Class A
1409	61	M	21.45737	0	0	1	0	0	0	1	1	0	0	0	0	0	1	1	0	0	0	150	161	84	98	5.73	6.84	8.83061	7.27546	10.2	9.5	4.3	3.9	68	96	30.1	28.7	20.8589	29.53846	1840	1810	336	586	1.02157	3.69603	NA	Class B
1410	85	M	24.4646	1	0	0	0	0	1	0	1	1	1	0	1	1	0	0	0	0	0	140	146	60	60	2	2.34	25.39403	21.31467	10.1	11.5	3	3.3	70	NA	266	NA	31.39013	NA	1530	NA	187	NA	2.58462	3.1746	5.6	Class A
1411	41	M	25.68956	0	0	1	0	0	1	0	0	0	0	0	0	0	1	1	0	0	0	112	119	73	64	3.48	3.84	17.07832	15.22898	10.2	11	4.1	4.1	92	67	173	201	38.33333	25.18797	1080	815	581	695	0.01265	0.49107	NA	Class A
1412	81	F	35.2095	0	0	1	0	1	0	0	0	0	0	0	0	0	0	0	0	0	1	118	173	63	97	1.33	1.27	29.73153	31.27157	9.8	11.6	3.5	3.5	22	289	13.6	53.3	6.128134	92.03822	4930	4460	301	348	0.14368	0.15676	NA	Class A
1413	85	F	19.49914	0	0	0	1	0	1	0	1	0	1	0	0	0	0	0	0	0	0	152	NA	76	NA	1.1	NA	36.0927	NA	8.3	NA	3.4	NA	68	NA	33.6	NA	24.63768	NA	396	NA	2260	NA	2.46	NA	5.9	Class A
1414	73	F	24.07093	1	0	0	0	1	0	0	1	1	0	0	0	0	0	0	0	0	0	164	110	70	67	1.17	1.27	35.24335	32.21894	9	9.6	3	3.6	42	53	445	211	15.73034	20	1090	225	199	204	1.95939	1.91156	7.1	Class A
1415	76	M	21.22701	0	1	0	0	0	1	0	1	0	0	0	0	0	1	1	0	0	0	146	132	68	64	3.06	3.29	16.46755	15.15537	10	10.8	3.8	3.8	72	44	283	207	29.03226	17.2549	249	265	908	819	0.6545	1.02873	NA	Class A
1416	61	F	22.0118	0	0	1	0	1	0	0	0	1	1	0	0	0	1	1	0	0	0	110	111	64	65	1.6	1.53	26.3481	27.66968	10.7	11.2	3.9	3.9	57	76	43.6	57.4	12.44541	16.66667	1340	1200	279	406	NA	0.10256	5.9	Class A
1417	71	M	28.58131	0	0	0	1	0	1	0	0	0	0	0	0	0	1	1	0	0	0	135	133	72	75	2.92	3.72	17.67509	13.50745	10.1	11.2	4.1	4.1	103	130	273	123	45.37445	52.41935	2080	2010	231	409	1.72747	3.06009	5.9	Class C
1418	41	M	30.79585	1	0	0	0	0	1	0	1	1	0	1	0	0	1	1	0	0	0	140	155	80	78	2.94	4.07	20.53812	14.38919	10.1	11.4	4	3.7	58	44	143	115	24.47257	18.64407	657	371	657	837	4.71021	5.12466	8.2	Class A
1419	86	M	33.71488	0	0	1	0	0	0	0	1	0	0	0	0	0	0	0	0	0	0	142	133	78	71	3.57	4.09	13.42708	11.53279	10.6	10.3	4.2	3.8	76	108	34.5	40.8	23.45679	34.83871	394	1200	595	839	4.33866	2.97279	5.3	Class A
1420	78	M	21.30395	0	1	0	0	0	1	0	0	1	1	0	0	1	1	1	0	1	1	128	140	48	57	5.28	7	8.99931	6.61048	7	9	3.8	3.5	73	93	122	133	34.43396	47.20812	150	89	2290	4530	1.38947	2.68293	5.9	Class C
1421	64	M	27.10925	1	0	0	0	0	1	0	0	1	0	0	0	0	1	1	0	0	0	122	150	80	62	3.86	4.69	13.41843	10.79525	10	9.6	3.5	3.2	82	68	93.7	57.1	30.71161	28.57143	652	1480	319	1350	5.87551	6.58076	6.2	Class B
1422	54	F	18.58725	0	0	1	0	1	0	0	0	0	0	1	0	0	0	0	0	0	1	71	78	45	52	1.1	NA	41.11171	NA	10.5	NA	3.3	NA	70	NA	56.6	NA	26.71756	NA	226	NA	263	NA	0.19653	NA	NA	Class A
1423	59	M	22.94801	0	0	0	1	0	1	0	0	0	0	0	0	0	1	1	0	0	1	116	119	73	72	3.52	4.17	15.19321	12.6223	9.5	10.4	3.1	3.6	135	103	218	356	63.08411	47.68519	126	1030	1740	3000	3.072	1.27289	NA	Class A
1424	67	F	17.49302	0	0	0	1	0	0	0	1	0	0	0	0	0	0	0	0	0	0	178	NA	88	NA	2.97	3.54	13.03668	10.71289	9.2	11.4	3	3.5	NA	62	NA	224	NA	21.98582	NA	3670	NA	2000	5.71429	1.53846	NA	Class A
1425	59	F	19.38002	0	1	0	0	1	0	0	0	0	0	0	0	0	0	0	0	0	0	120	120	73	74	1.85	1.29	22.69477	33.66871	10.4	11.1	3.9	3.7	57	54	60.3	37.5	18.93688	17.0347	798	877	164	144	0.15789	0.29825	NA	Class A
1426	73	F	23.14646	0	1	0	0	1	0	0	0	1	0	0	0	0	1	1	0	1	1	132	138	68	68	2.03	1.02	19.28732	40.79135	9.8	11.6	3.5	3.8	NA	NA	NA	NA	NA	NA	NA	NA	NA	NA	0.16219	0.27553	5.7	Class A
1427	84	M	22.28981	0	1	0	0	0	1	0	1	0	0	0	0	0	1	1	0	0	0	148	122	70	80	2	2.2	25.48043	22.95742	9.7	11.2	3.8	3.8	57	90	78.3	91.3	21.34831	36.88525	677	304	643	426	0.15537	0.12111	NA	Class A
1428	67	M	22.69896	1	0	0	0	0	1	0	1	1	0	0	0	0	1	0	1	0	1	150	146	68	80	3.31	3.77	15.66846	13.58942	9.7	11.5	3.7	3.6	55	58	106	59.4	21.65354	22.65625	144	301	636	401	3.83338	3.39636	6.3	Class A
1429	70	F	28.56113	1	0	0	0	1	0	0	0	1	0	0	0	0	1	1	0	1	1	120	126	78	82	4.49	6.43	8.19116	5.52994	8.8	9.6	3	2.9	53	NA	28	NA	19.34307	NA	450	NA	12500	NA	8.14528	8.23426	5.3	Class C
1430	50	M	26.96381	1	0	0	0	1	0	0	0	1	0	0	0	0	0	0	0	0	0	130	130	70	70	2.62	2.76	22.00775	20.78944	8.5	10.3	3.4	3.1	97	26	514	329	34.89209	9.454545	216	20100	88	210	NA	NA	6.3	Class B
1431	88	F	15.8819	0	0	1	0	1	0	0	1	0	0	0	0	0	1	1	0	0	0	148	120	76	61	1.04	1.21	37.99664	32.19678	9.4	10.7	3.5	3.3	62	88	125	74.2	20.39474	27.32919	397	545	1040	702	0.63291	0.32468	NA	Class A
1432	78	M	30.55244	0	1	0	0	1	0	0	0	0	0	0	0	0	1	1	0	1	1	119	116	53	62	2.06	1.88	25.19996	27.85107	9.4	11.4	4	4.2	35	68	4.9	27.8	10.80247	26.66667	1410	3270	178	175	0.11146	4.80519	NA	Class A
1433	83	M	24.12879	0	1	0	0	0	1	0	1	0	0	0	0	0	0	0	0	0	0	142	139	65	61	3.4	3.98	14.30831	12.04354	9.7	8.7	NA	3.6	62	63	156	131	25.10121	26.69492	452	1550	2480	7250	2.62903	5.44	NA	Class A
1434	79	M	21.07325	0	0	1	0	1	0	0	0	1	0	0	0	0	1	1	0	0	0	125	120	63	68	5.73	9.01	8.19901	4.99705	8.3	11	3.2	3.5	69	99	182	70.3	40.58824	47.59615	1110	1280	1280	2610	3.23438	4.42664	5.8	Class B
1435	32	F	22.3983	0	0	1	0	1	0	0	0	0	0	0	0	0	1	1	0	0	1	131	132	85	93	2.02	2.01	24.57048	24.70425	10.3	11.7	3.9	3.9	26	169	21.4	48.3	10.44177	68.69919	172	147	211	143	2.22642	2.52632	NA	Class A
1436	67	F	23.12467	0	0	0	1	1	0	0	0	0	0	0	0	0	1	1	0	0	1	106	109	62	65	2.08	1.74	19.2487	23.39924	9.4	8.4	4.2	4.2	97	117	34.9	56.1	30.3125	41.19718	843	492	129	136	0.40947	0.26911	5.3	Class A
1437	72	M	31.00833	1	0	0	0	0	0	1	1	1	1	1	1	0	1	1	0	0	0	168	157	77	79	3.14	3.06	16.25947	16.72507	10.3	11.5	3.8	3.9	64	54	110	71.9	27.11864	21.68675	1700	1420	867	729	NA	NA	6.1	Class A
1438	76	M	25.48173	0	1	0	0	0	1	0	1	1	1	0	0	0	1	1	0	0	0	142	123	75	69	2.14	2.18	24.35205	23.86365	10.3	11.5	3.8	3.9	94	140	1170	1460	41.40969	56.68016	166	169	393	248	0.75249	0.31938	6.2	Class A
1439	57	M	17.83667	0	0	0	1	1	0	0	0	0	0	0	0	0	1	1	0	1	1	118	133	74	89	2.04	2.08	27.73081	27.14792	8.9	10.2	3.3	3.4	21	15	54.3	55.3	7.420495	5.597015	52800	62600	784	867	0.95951	2.41893	NA	Class A
1440	72	M	21.69233	1	0	0	0	0	1	0	1	1	0	0	0	0	1	1	0	0	0	145	167	68	80	4.03	5.31	12.37495	9.15153	9.9	11.5	3.8	3.7	73	46	138	105	27.8626	17.89883	90	492	589	539	2.73077	4.80952	5.8	Class B
1441	43	F	NA	0	0	1	0	1	0	0	0	0	0	0	1	0	1	1	0	0	1	NA	NA	NA	NA	1.07	NA	45.23711	NA	9.9	NA	3.6	NA	14	NA	8.3	NA	3.825137	NA	227	NA	141	NA	0.08333	NA	NA	Class A
1442	51	F	17.08793	0	0	0	1	0	0	1	0	0	0	0	0	0	1	1	0	0	0	127	125	79	75	3.77	3.49	10.86066	11.81742	10.3	11.4	3.6	3.7	65	65	39.2	43.5	26.74897	26.10442	626	862	450	542	1.58491	2.7	NA	Class A
1443	54	F	27.05515	0	0	1	0	1	0	0	0	0	0	0	0	0	1	1	0	0	0	115	121	60	71	2.82	2.67	14.67833	15.58281	9.1	10.2	4.1	4	67	41	53.6	36	27.01613	15.5303	562	897	135	94	NA	0.64841	6.2	Class A
1444	89	M	19.85043	0	1	0	0	0	1	0	0	0	0	0	0	0	1	1	1	1	1	110	124	54	56	2.43	2.34	20.25226	21.10594	8.3	9.5	3.8	4	71	78	162	119	25.44803	26.62116	4430	644	2920	3000	0.09646	0.2	NA	Class A
1445	77	M	23.24219	1	0	0	0	0	1	0	1	1	0	0	0	0	0	0	0	0	1	188	170	88	60	3.86	4.6	12.72484	10.5032	8	9.9	2.7	2.9	56	84	295	111	23.04527	34.28571	3400	1120	13400	17800	9.62099	11.51834	5.3	Class C
1446	74	M	24.30717	1	0	0	0	1	0	0	0	1	1	1	0	0	0	0	0	1	1	132	143	73	76	3.21	3.14	15.74765	16.13211	10.1	11.3	3.5	3.7	92	102	23.2	13.8	27.05882	26.49351	636	237	1810	1410	1.98718	3.06818	5.7	Class A
1447	89	M	21.89386	0	0	0	1	1	0	0	0	0	1	1	0	0	1	0	1	1	1	117	171	40	56	2.46	1.75	19.98222	29.00302	9.3	12	3.2	2.8	72	NA	175	NA	28.23529	NA	13400	NA	14200	NA	0.95475	0.92807	5.9	Class A
1448	74	M	25.89532	1	0	0	0	0	0	1	0	1	0	0	0	1	1	1	0	0	1	127	NA	51	NA	2.33	NA	22.35861	NA	9.5	NA	3.5	NA	60	NA	49.2	NA	21.12676	NA	347	NA	153	NA	2.0396	NA	7	Class A
1449	79	M	21.66154	0	1	0	0	0	1	0	0	0	0	0	0	1	1	0	1	0	0	124	126	64	60	2.83	3.33	17.73897	14.79316	9.7	10.5	3.8	3.7	60	75	110	63.2	28.57143	34.09091	833	698	911	1330	0.40741	0.90909	NA	Class A
1450	81	F	22.23187	0	0	1	0	0	1	0	0	0	0	0	0	0	1	1	0	0	0	122	175	55	68	1.89	1.66	20.24239	23.24788	10	10.9	3.8	3.9	31	63	84.2	62.8	11.92308	22.1831	26400	11500	292	576	0.08282	0.0479	NA	Class A
1451	79	F	21.41804	0	1	0	0	1	0	0	1	0	0	0	0	0	1	1	0	0	0	140	139	79	76	3.61	3.72	10.04418	9.71971	10	11.9	4.2	4.3	40	73	83.5	47	14.76015	24.49664	4270	359	611	362	1.05714	0.84906	5.6	Class A
1452	78	M	25.64645	1	0	0	0	0	1	0	1	1	0	0	0	1	1	1	0	0	0	160	153	65	65	2.92	3.42	17.20449	14.41977	9.2	10.9	3.4	3.4	80	73	241	140	30.53435	29.79592	486	499	1040	755	7.62082	5.98131	5.8	Class C
1453	72	F	26.96291	0	1	0	0	1	0	0	0	1	0	0	0	0	0	0	0	0	1	132	165	79	90	2.27	2.63	17.13561	14.5868	8.5	11.1	3.5	3.9	56	NA	174	NA	24.45415	NA	5570	NA	2180	NA	1.70338	3.76829	NA	Class A
1454	67	F	17.73438	0	0	1	0	1	0	0	0	0	0	1	0	0	1	1	0	0	1	122	116	70	77	3.69	3.59	10.28101	10.59472	8.9	10.8	4.2	4.5	54	111	21.7	33.1	16.56442	33.84146	3100	419	1030	980	0.67146	1.52941	5.6	Class A
1455	57	M	22.22136	0	0	0	1	0	1	0	0	1	0	0	0	0	1	1	0	0	1	130	136	72	70	5.51	6.38	9.39822	8.00556	9.3	10.5	2.5	2.6	74	59	10.6	10.1	32.74336	25	6260	1540	21500	8860	14.39286	10.8	6.4	Class B
1456	75	M	15.78924	0	1	0	0	1	0	0	0	0	0	0	1	0	0	0	0	0	1	111	119	54	43	2.71	3.38	18.87951	14.76976	9.2	9.2	3.1	2.7	50	32	74.9	88.5	23.80952	16.24365	9300	29600	3350	34600	NA	3.78894	NA	Class A
1457	73	F	19.39619	0	0	0	1	1	0	0	0	0	0	0	0	0	1	1	0	0	1	132	111	85	72	1.33	1.35	30.51286	30.01868	9.8	12.1	4	4.1	95	80	33.4	35.3	29.32099	25.72347	122	132	199	124	0.81974	0.82034	5.3	Class A
1458	60	M	NA	0	0	1	0	0	1	0	1	0	0	0	0	0	1	1	0	0	0	148	NA	83	NA	4.57	4.88	11.36381	10.57648	10.6	10.1	4.1	4	44	67	206	194	17.81377	30.04484	709	883	209	289	NA	2.47357	NA	Class A
1459	77	F	24	0	1	0	0	0	1	0	1	1	1	0	0	0	1	1	0	0	0	180	130	82	71	3.69	3.29	9.87862	11.19982	9	9.4	4	3.5	68	47	209	89.2	17.21519	20.43478	4570	4420	767	711	NA	3.93651	5.8	Class A
1460	69	M	26.50212	0	1	0	0	0	0	1	1	1	0	0	0	0	0	0	0	0	0	157	NA	70	NA	3.22	3.71	16.01245	13.65726	9.3	10.3	3.9	4.3	49	54	239	252	20.08197	20.84942	4560	1920	903	2160	3.22314	1.54905	5.9	Class C
1461	82	M	19.55353	1	0	0	0	1	0	0	0	1	0	0	0	1	0	0	0	1	1	114	130	71	72	2.39	2.01	21.11397	25.51765	8.8	10.2	3.5	3.9	98	96	57.9	96.2	34.50704	31.89369	181	251	1570	1340	0.002494	0.5988	6.6	Class B
1462	77	M	NA	0	1	0	0	0	1	0	0	1	0	0	0	0	0	0	0	0	0	135	191	74	93	3.19	2.77	15.67588	18.29389	10	11.8	4	4	81	91	354	208	31.88976	37.44856	450	420	735	1260	0.02885	2.74074	6.6	Class A
1463	70	F	17.60153	0	0	0	1	1	0	0	0	0	0	0	0	0	0	0	0	0	0	128	145	78	88	2.89	2.85	13.26419	13.46799	10.5	10	4.1	2.9	54	29	103	144	18.68512	12.7193	50	9190	470	770	3	3.87755	5.5	Class A
1464	65	F	22.22656	0	0	1	0	1	0	0	0	0	0	0	0	0	1	1	0	0	0	120	NA	60	NA	2.62	2.29	15.08407	17.47753	10.5	11.3	4.2	4	88	86	141	122	36.97479	36.28692	414	690	279	217	0.42033	0.68274	NA	Class A
1465	66	F	20.59905	0	0	1	0	1	0	0	0	0	0	0	0	0	0	0	0	0	0	114	106	73	63	3.36	3.72	11.43989	10.23442	9.1	10.7	3.9	3.6	77	70	43.1	15.4	22.06304	18.86792	1020	243	556	149	0.93313	0.55046	NA	Class A
1466	79	M	22.22825	1	0	0	0	0	1	0	0	1	0	0	0	1	0	0	0	0	1	NA	139	NA	48	2.21	2.32	23.24971	22.04646	8.4	9.7	3.5	3.8	26	19	30.8	80.2	8.695652	7.169811	542	22300	1670	3040	4.94562	3.09609	6.8	Class B
1467	74	M	21.68115	0	1	0	0	0	1	0	0	0	0	0	0	0	0	0	0	0	0	127	117	60	58	2.95	NA	17.27217	NA	10.3	NA	3.4	NA	48	48	182	154	29.26829	27.42857	5990	12700	781	1070	0.63097	NA	5.9	Class A
1468	77	F	26.23797	0	1	0	0	1	0	0	1	1	0	0	0	0	1	1	0	0	0	164	152	86	72	3.34	3.59	11.01653	10.18005	8.3	10.1	3.4	3.7	60	63	130	111	28.30189	24.90119	534	585	652	669	1.6	2.89394	6.3	Class B
1469	71	M	19.73466	0	0	0	1	0	1	0	0	1	1	0	0	0	0	0	0	0	0	NA	NA	NA	NA	1.85	2.1	29.12089	25.24872	10.8	10.9	3.6	3.6	132	150	18.8	35.1	37.82235	42.01681	87	51	688	840	1.26087	0.59167	NA	Class A
1470	62	F	39.77438	1	0	0	0	1	0	0	1	1	1	0	0	1	1	1	0	0	1	156	133	83	73	2.26	2.33	17.9736	17.38371	10.7	11.6	4	3.9	54	52	25.7	50.1	16.82243	17.3913	2980	4110	274	651	1.25676	1.12879	7.1	Class C
1471	82	M	22.65292	0	1	0	0	0	1	0	1	0	0	0	0	0	1	1	0	1	1	143	133	62	60	15.7	3.16	2.68356	15.50132	9.4	11.2	3.4	3.5	79	84	91.5	107	34.64912	38.88889	1200	1010	1160	690	0.51208	1.00193	5.5	Class B
1472	69	M	23.31357	0	1	0	0	1	0	0	0	0	0	0	0	0	1	1	1	0	0	116	108	73	67	2.66	2.51	19.73475	21.02853	11	12	4.1	3.5	98	62	81.6	21.5	43.17181	20.59801	1040	1660	152	110	0.28814	0.33755	5.1	Class A
1473	73	M	NA	0	0	0	1	0	1	0	0	1	0	0	0	0	1	1	0	0	0	132	130	69	62	2.62	2.6	19.7427	19.9089	10.8	12	4.3	4.6	66	106	61	63.4	22.22222	30.54755	197	564	336	678	1.24051	2.01786	6	Class A
1474	68	M	16.38261	1	0	0	0	0	1	0	1	1	0	0	0	0	1	1	0	0	0	142	107	68	64	2.54	NA	20.84412	NA	8.2	NA	2.5	NA	94	116	754	593	49.47368	56.58537	378	785	1790	1210	11.02222	NA	5	Class A
1475	77	F	25.63117	0	1	0	0	1	0	0	0	0	0	0	0	0	1	1	0	0	0	NA	162	NA	88	1.47	1.26	27.03822	32.00501	10.6	11.9	4	4.3	77	98	23.2	10.6	26.01351	28.82353	298	141	646	495	0.06006	NA	6.3	Class A
1476	75	M	NA	0	0	1	0	0	1	0	0	0	0	0	0	0	0	0	0	0	0	128	124	70	73	1.86	1.66	28.49784	32.2746	9.7	11.6	3.5	3.6	54	65	396	310	27.41117	30.95238	19000	12000	231	110	0.07792	0.06667	NA	Class A
1477	68	F	29.27796	0	0	1	0	1	0	0	0	0	0	0	0	1	1	1	0	0	0	126	146	74	84	1.22	1.09	34.21518	38.70365	10.1	9.3	4.2	4	NA	40	NA	13.3	NA	11.33144	NA	447	NA	74	NA	NA	5.9	Class A
1478	70	M	24.91998	0	1	0	0	0	1	0	0	0	0	0	0	0	1	1	0	0	0	127	124	70	71	4.2	5.06	11.87561	9.68612	10.4	11.8	4.2	3.7	83	115	182	144	35.31915	51.33929	306	256	761	652	1.89531	2.6703	NA	Class B
1479	69	F	18.25632	1	0	0	0	0	0	1	0	1	0	0	1	0	0	0	0	0	0	137	141	47	54	3.51	NA	10.76789	NA	10.2	NA	NA	NA	60	55	69.1	50.9	23.52941	22.44898	318	459	1480	2780	13.35294	NA	6.5	Class B
1480	57	F	21.27101	1	0	0	0	1	0	0	1	1	0	0	0	0	1	1	0	0	1	156	142	80	86	1.56	1.66	27.62052	25.80547	9.4	10.4	3.8	3.8	70	74	148	105	24.73498	25.42955	114	145	274	180	2.93548	2.89916	6.2	Class A
1481	59	F	17.86398	0	0	1	0	1	0	0	0	0	0	0	0	0	1	0	1	0	1	130	124	84	61	1.58	1.53	26.96999	27.80125	10.8	11.1	4.4	4.1	89	70	103	110	38.86463	29.2887	101	94	130	102	0.23438	0.16667	NA	Class A
1482	57	F	21.92884	1	0	0	0	1	0	0	1	1	0	0	0	0	1	1	0	0	0	145	137	75	62	1.29	1.27	34.0036	34.58986	10.1	11.3	4.1	4.1	49	87	221	141	17.81818	30.96085	1120	505	136	61	1.09925	1.03015	7	Class A
1483	74	M	NA	1	0	0	0	0	1	0	0	1	1	0	1	1	1	1	0	1	1	130	134	80	84	1.89	1.98	28.11143	26.71656	10.6	12.4	NA	3.9	66	65	91.7	84.4	28.82096	24.16357	397	981	561	342	NA	0.91304	NA	Class A
1484	71	M	19.37432	0	0	0	1	0	1	0	1	1	0	0	0	0	1	1	0	0	0	160	120	80	60	2.15	NA	24.70603	NA	10.3	NA	3.9	NA	67	74	88.6	35.1	28.15126	26.33452	4250	483	756	351	0.07456	NA	NA	Class A
1485	95	F	21.56746	0	0	1	0	1	0	0	1	0	1	0	1	0	0	0	0	0	0	158	146	58	48	2.22	2.4	16.21546	14.88978	8.3	8.3	3.5	3.4	57	40	41.7	30.6	20.57762	12.30769	74	327	11000	3090	6.67153	4.81069	5.2	Class A
1486	76	M	18.81539	1	0	0	0	0	1	0	0	1	0	0	0	0	1	1	0	1	1	118	129	69	73	1.88	1.83	28.05948	28.89926	8.9	11.1	3.57	3.82	92	114	29.3	74.6	26.06232	37.2549	91	139	4040	1550	2.67775	1.34856	5.7	Class A
1487	55	F	27.27633	0	0	0	1	0	0	0	0	0	0	0	0	0	0	0	0	1	1	136	155	65	80	2.92	2.93	14.05508	14.00261	10.1	11.1	3.9	3.7	55	78	46	78.1	20.37037	30.46875	3710	3540	361	731	3.97928	2.53185	5.6	Class A
1488	78	M	22.21037	0	0	1	0	0	1	0	0	0	0	0	0	0	0	0	0	0	0	127	127	68	70	3.37	2.68	14.70766	18.82796	9.2	12.5	4.7	4.6	103	85	381	82.9	31.69231	24.28571	50	58	150	145	0.36999	0.18868	NA	Class A
1489	83	F	21.83281	0	0	1	0	1	0	0	0	0	0	0	0	0	0	0	0	0	0	139	144	68	80	1.25	NA	31.59739	NA	8	NA	3.3	NA	40	NA	139	NA	15.68627	NA	7440	NA	1350	NA	1.20913	NA	6.2	Class A
1490	80	M	24.73329	0	0	0	1	0	1	0	0	0	1	0	0	0	1	1	0	0	0	94	150	48	93	1.5	1.39	35.39738	38.33614	10.7	12.2	3.6	3.6	171	122	48.8	18.8	55.16129	34.95702	336	222	1370	1600	0.51899	2.25117	NA	Class A
1491	68	M	25.95738	0	0	0	1	0	1	0	1	0	0	0	0	1	1	1	0	0	0	142	142	79	68	3.29	3.4	15.70577	15.15074	10.9	12.3	3.8	3.6	96	91	114	42.3	34.65704	33.95522	845	293	327	268	2.92808	2.28018	NA	Class B
1492	80	M	25.99623	1	0	0	0	0	1	0	1	1	0	0	0	0	1	0	1	0	0	146	161	70	82	3.73	3.67	13.06669	13.25323	9.1	10.6	3.2	3.6	113	101	165	157	39.78873	36.07143	60	108	1160	2930	2.2272	3.55856	6.4	Class A
1493	63	F	25.02465	0	0	1	0	1	0	0	1	1	0	0	0	0	0	0	0	0	0	160	177	91	97	1.87	0.84	22.01105	52.82918	8.4	9.1	2.3	3.2	48	73	292	77.6	29.44785	32.01754	1890	2040	2550	852	4.46394	9.17801	5.8	Class A
1494	79	F	14.56816	0	0	0	1	1	0	0	1	0	0	0	0	0	0	0	0	0	0	146	130	88	86	0.75	0.82	56.04155	50.82938	9.9	10.3	3.3	2.8	57	99	57.1	46.5	17.43119	30.46154	7740	907	199	447	1.58333	7.40506	NA	Class A
1495	75	F	14.90418	0	0	1	0	1	0	0	0	1	0	0	0	0	1	1	0	0	0	113	132	61	68	1.03	0.9	40.20311	46.59746	9.4	12.2	2.9	3.1	119	104	750	323	46.3035	40.46693	1710	1540	881	655	0.53604	1.32075	6	Class A
1496	36	M	30.46112	1	0	0	0	1	0	0	1	1	0	0	0	0	0	0	0	0	0	170	167	90	110	2.64	4.54	23.98326	13.25329	9.6	9.3	3.1	2.1	52	39	172	158	16.61342	16.66667	1450	3790	809	1490	5.55712	4.20742	6.4	Class C
1497	63	M	22.81728	1	0	0	0	0	0	1	1	1	0	0	0	0	1	1	0	0	1	154	151	76	92	3.62	3.79	14.45985	13.68979	9.2	10.4	3	3.1	89	115	50.5	59.8	30.58419	43.56061	87	82	1710	2710	11.37255	12.68852	6	Class A
1498	80	M	22.22906	0	1	0	0	0	1	0	0	1	0	0	0	0	1	1	0	0	0	138	146	73	83	2.41	2.04	21.07115	25.28596	10.1	12.3	4.1	3.9	45	80	693	668	18.82845	31.62055	3010	408	243	183	0.24339	0.39752	6.3	Class A
1499	75	F	NA	0	0	1	0	1	0	0	0	0	0	0	0	0	1	1	0	0	0	NA	NA	NA	NA	0.94	NA	44.4326	NA	11.2	NA	3.9	NA	76	NA	32.4	NA	23.75	NA	115	NA	107	NA	0.30769	NA	6	Class A
1500	84	M	20.08766	0	1	0	0	1	0	0	0	0	0	0	0	0	1	1	0	1	1	110	99	42	39	1.54	1.72	33.91454	30.05145	8	10.8	3.5	3.7	147	155	451	441	72.05882	66.81034	155	171	853	424	0.13793	0.16	NA	Class A
1501	77	M	24.43918	0	0	0	1	0	1	0	1	0	0	0	0	0	1	1	0	0	0	197	132	70	72	2.24	2.44	23.0785	21.01718	10.6	10.9	2.6	2.7	50	70	192	134	27.02703	33.01887	188	188	23600	26100	6.51774	10.33731	NA	Class A
1502	47	M	24.22884	1	0	0	0	1	0	0	0	1	1	1	0	0	0	0	0	0	0	83	162	56	94	1.62	1.83	37.90532	33.17326	11.4	10.6	3.1	2.5	57	78	308	215	22.98387	39.39394	732	984	144	710	2.64498	11.84896	7.1	Class A
1503	55	M	17.857	0	0	1	0	1	0	0	0	0	0	0	0	0	0	0	0	0	1	130	124	84	79	3.95	4.58	13.66597	11.56338	10.5	12.3	3.8	4	62	58	26	25.3	22.46377	21.80451	294	146	565	790	3.35	3.68139	5.6	Class A
1504	51	F	16.5695	0	0	0	1	0	1	0	1	0	0	0	0	1	1	0	1	1	1	188	111	86	56	1.67	2.11	26.32094	20.37925	9.1	11.4	3.1	4.2	27	84	136	132	12.16216	25.60976	2850	295	1200	422	0.24831	0.68966	NA	Class A
1505	72	M	26.60648	0	0	0	1	0	1	0	0	1	1	1	0	1	1	1	0	0	0	117	100	53	64	1.8	2.08	29.88678	25.41363	10.8	12.6	3.8	4	39	81	206	143	14.94253	29.8893	3500	489	604	240	NA	0.7958	8.9	Class A
1506	50	M	29.61267	1	0	0	0	1	0	0	0	1	1	1	0	0	1	1	0	0	1	128	101	64	52	6.85	6.74	7.69045	7.7835	10.7	12.9	4.1	3.9	55	65	102	55.7	23.01255	26.10442	1900	478	992	1290	2.97391	3.63359	6.2	Class B
1507	63	M	NA	0	0	0	1	0	1	0	0	0	0	0	0	0	1	1	0	0	0	132	133	83	84	5.32	6.13	9.48951	8.12661	9.4	10.6	3.5	3.9	89	102	63.1	54.8	31.56028	36.42857	92	69	221	146	2.94118	3.15789	NA	Class A
1508	74	M	26.24501	1	0	0	0	0	1	0	0	1	1	0	0	0	1	0	1	0	1	124	135	64	69	2.13	2.11	24.66522	24.9211	9.4	11	3.6	3.5	84	NA	430	NA	32.43243	NA	186	NA	2680	NA	4.37548	5.52214	5.3	Class B
1509	79	M	22.60418	0	1	0	0	1	0	0	1	0	0	0	1	0	0	0	0	1	1	135	129	95	90	3.15	3.17	15.77724	15.66837	11.1	11.3	3.8	3.7	113	NA	28.7	NA	40.21352	NA	841	NA	231	NA	2.11827	NA	NA	Class A
1510	67	M	NA	1	0	0	0	0	0	1	0	1	0	0	0	0	1	0	1	0	0	110	120	60	80	2.85	2.49	18.37686	21.30245	9.8	10.8	4.3	4.2	67	50	140	75.5	28.75536	19.23077	83	339	6470	7010	0.90858	2.45877	6.5	Class A
1511	74	M	25.51323	1	0	0	0	1	0	0	1	1	0	0	1	0	1	1	0	0	0	177	178	83	98	4.77	5	10.21017	9.69748	10.3	11.9	4.2	4.3	94	77	263	178	47.95918	36.49289	2910	1760	590	691	0.55632	0.61219	6.2	Class A
1512	76	F	22.2859	1	0	0	0	1	0	0	1	1	1	1	0	0	0	0	0	0	0	150	148	78	70	2.41	2.87	15.80251	13.05359	9.2	8.9	3.2	3.8	49	48	149	38.8	18.63118	19.2	909	137	1270	450	2.76243	1.37738	9.6	Class C
1513	60	M	21.30644	1	0	0	0	0	1	0	0	1	0	0	0	0	0	0	0	0	0	128	118	83	91	3.14	NA	17.13292	NA	10.3	NA	3.9	NA	103	107	214	142	36.52482	36.27119	456	270	411	406	0.80488	NA	NA	Class A
1514	73	F	19.89688	0	0	1	0	1	0	0	0	0	0	0	0	0	1	1	0	0	0	115	108	70	60	1.48	1.7	27.25249	23.41862	9.3	10.7	3.8	3.7	114	73	148	87	40.5694	26.73993	50	3320	302	188	0.75235	0.58968	NA	Class A
1515	24	F	17.01323	0	0	1	0	1	0	0	0	0	0	0	0	0	1	1	0	1	1	119	117	80	73	2.11	NA	25.44254	NA	9.6	NA	4.4	NA	131	NA	37.9	NA	52.19124	NA	139	NA	92	NA	0.05882	NA	NA	Class A
1516	81	M	NA	0	1	0	0	0	0	1	1	0	0	0	0	1	1	1	0	0	0	154	141	80	62	1.71	NA	30.56109	NA	10.5	NA	3.8	NA	114	123	196	184	56.71642	61.80905	805	1160	516	600	0.34274	NA	5.9	Class A
1517	76	F	25.70486	0	1	0	0	1	0	0	1	0	0	0	0	0	1	1	0	0	0	153	166	82	92	1.39	1.44	28.85323	27.65507	10	10.4	4.03	3.94	55	72	91.8	76.1	20.52239	25.99278	374	638	256	261	0.06289	0.05744	NA	Class A
1518	28	M	23.22441	0	1	0	0	0	1	0	0	0	0	0	0	1	0	0	0	0	0	120	130	60	70	2.5	2.1	27.36031	33.11002	8.5	9.1	3.3	3	31	NA	197	NA	9.935897	NA	35700	NA	1560	NA	1.77745	3.81176	NA	Class A
1519	79	M	NA	0	1	0	0	0	0	0	1	0	0	0	1	0	1	1	0	0	0	140	128	76	76	3.69	4.25	13.26954	11.36908	9.6	11.3	4	3.9	89	76	113	82	30.68966	25.76271	208	14500	156	139	NA	3.06122	NA	Class A
1520	83	M	16.05166	0	1	0	0	0	1	0	0	0	1	0	0	1	0	0	0	0	1	105	132	59	67	5.1	5.14	9.18215	9.10401	7.7	10.5	3.5	3.3	64	97	68.9	186	23.18841	36.88213	3240	1840	9900	25200	0.51282	0.66116	5.5	Class C
1521	85	M	NA	0	0	1	0	0	1	0	0	1	0	0	0	0	1	1	0	0	0	123	NA	63	NA	2.79	2.92	17.6428	16.78532	10	13	3.4	3.8	107	103	81	64.8	45.53191	43.09623	2020	189	368	148	4.53704	4.09524	5.8	Class B
1522	41	M	36.71699	1	0	0	0	1	0	0	0	1	0	0	0	0	1	1	0	0	0	136	128	73	71	5.64	4.43	10.07009	13.11496	9.3	10.1	3.5	4.2	90	115	157	88	37.97468	44.0613	708	300	578	459	5.03662	3.09159	5.1	Class A
1523	50	M	27.8933	1	0	0	0	0	0	0	1	1	0	0	0	0	1	1	0	0	0	135	122	90	76	6.87	8.07	7.66596	6.42803	10.6	10.8	4.1	4.3	100	105	295	283	29.67359	32.50774	3960	2070	154	269	1.82451	1.92434	6.1	Class A
1524	56	F	25.5128	0	0	1	0	1	0	0	1	0	0	0	0	0	1	1	0	0	0	144	143	86	89	1.6	1.9	27.00281	22.37483	10.4	11.8	4.1	4	74	84	44.8	25.6	24.2623	26.25	342	291	177	114	0.18337	0.15817	NA	Class A
1525	50	M	21.98537	0	1	0	0	0	1	0	0	0	0	0	0	0	1	1	0	0	0	139	115	47	75	6.32	6.62	8.39872	7.93799	10.2	10.3	4.3	4.1	NA	92	NA	106	NA	39.14894	173	170	904	1570	0.76625	0.67083	NA	Class B
1526	72	M	25.02595	0	1	0	0	0	1	0	0	0	0	0	1	0	1	1	0	0	0	136	126	77	79	4.6	4.4	10.70755	11.24113	10.6	11.9	4.4	4.3	72	85	109	86.8	29.75207	36.63793	694	594	399	529	0.5	0.44118	5.6	Class A
1527	69	M	22.73322	0	0	0	1	0	1	0	0	1	0	0	0	0	1	1	0	0	0	110	125	67	80	5.84	5.84	8.34827	8.34827	10.1	12.2	3.9	4.1	54	131	96.7	46.7	20.14925	47.29242	1020	191	3320	2160	4.68555	4.95841	5.2	Class B

1528	49	F	20.1408	0	0	1	0	1	0	0	0	0	1	0	0	0	0	0	0	0	1	119	114	62	65	1.02	0.91	45.91465	51.71904	10.7	13.4	4.2	3.9	49	64	7.3	40	11.7506	20.44728	1840	2050	44	68	0.15385	0.08333	NA	Class A
1529	66	M	25.74074	1	0	0	0	0	0	1	0	1	1	0	0	0	0	0	0	0	0	117	118	63	69	3.57	NA	14.48684	NA	10.7	NA	3.3	NA	92	83	31	40.9	30.97643	26.86084	667	1150	9680	6640	2.9182	NA	7.4	Class A
1530	80	M	22.65625	1	0	0	0	1	0	0	1	1	1	0	0	0	1	1	1	0	1	140	167	80	71	2.45	2.86	20.69508	17.41013	9.2	11.2	4	4.2	78	71	40.5	45.4	22.41379	20.0565	389	279	624	516	1.43451	6.21053	5	Class A
1531	89	F	22.7747	0	1	0	0	0	0	1	0	0	0	0	1	0	1	1	0	1	1	119	104	66	49	1	1	39.53404	39.40747	9	11.8	2.9	3.2	70	55	32.2	39.3	28.80658	23.30508	16900	2010	2140	726	1.01852	NA	NA	Class A
1532	80	M	33.09042	0	0	1	0	1	0	0	0	1	0	0	0	0	0	0	0	0	0	128	140	74	82	2.63	2.36	19.15064	21.56002	10.6	10.6	3.6	3.3	58	52	89.2	93.6	23.57724	22.12766	2720	3850	349	520	0.22727	0.5	6.7	Class A
1533	67	M	23.10753	0	0	0	1	0	1	0	0	0	0	0	0	0	1	1	0	0	0	132	NA	76	NA	2.62	2.45	20.23469	21.77562	10.2	11.2	NA	3.7	49	NA	33.1	NA	19.9187	NA	121	NA	206	NA	0.83871	2.04211	6.1	Class A
1534	64	M	23.59091	1	0	0	0	0	1	0	0	1	0	0	0	0	0	0	0	0	0	131	128	68	75	3.57	3.26	14.61534	16.1424	9.8	10.3	3.7	3.8	91	58	130	61.8	30.95238	17.0088	171	595	273	216	1.87931	1.775	6.5	Class A
1535	74	F	NA	1	0	0	0	0	1	0	0	1	0	0	0	0	1	1	0	1	1	162	138	81	72	2.35	2.47	16.36924	15.50123	9.9	10.1	NA	3.9	NA	60	NA	206	NA	22.81369	NA	4370	NA	610	NA	NA	NA	Class B
1536	84	F	23.28342	0	1	0	0	1	0	0	1	0	0	0	0	0	1	1	1	0	0	151	133	87	82	1.6	1.73	24.03647	22.06762	10.6	11	3.7	3.9	79	103	50.2	49.1	32.64463	37.18412	527	440	365	401	2.1519	1.61739	NA	Class A
1537	75	M	30.22222	0	1	0	0	0	1	0	1	0	0	0	0	0	1	0	1	0	0	140	136	70	70	2.04	1.57	25.75869	34.17386	10.1	11.2	4.3	4.1	98	51	232	188	32.99663	20	152	10300	1290	4000	0.10435	0.15484	NA	Class A
1538	68	F	22.71884	0	0	0	1	1	0	0	0	0	0	0	0	0	1	1	0	0	0	132	122	66	64	3.15	2.81	12.17207	13.73447	9.4	12	3.99	4.09	60	93	107	52.7	20.27027	27.92793	338	286	72	120	1.3049	1.29091	NA	Class A
1539	46	M	25.12325	1	0	0	0	0	1	0	0	1	0	0	0	0	1	1	0	0	0	137	119	77	74	4.52	5.08	12.41277	10.92384	8.8	9.7	3.8	3.9	42	41	99	97.9	20.28986	18.06167	9930	2620	170	139	1.44954	1.40041	6.5	Class A
1540	69	M	24.57524	0	0	0	1	1	0	0	0	0	0	0	0	0	0	0	0	0	1	130	144	74	85	2.91	3.8	17.88764	13.35884	10.6	11.9	4.2	3.8	86	123	27.9	83.2	29.55326	44.72727	312	263	258	190	4.03338	7.36086	5.6	Class B
1541	71	M	21.80876	0	1	0	0	0	1	0	0	0	0	0	1	0	1	1	0	0	0	118	134	64	82	4.75	5.85	10.37977	8.2646	9.4	12.1	3.1	3.1	83	120	186	55.4	40.68627	51.94805	4310	1590	633	778	0.60486	6.38408	NA	Class C
1542	78	F	19.37439	0	0	0	1	1	0	0	0	0	0	0	0	0	0	0	0	0	1	109	138	60	65	1.6	1.5	24.55318	26.34943	5.4	8	2.4	3.2	9	65	4.2	17.5	2.848101	22.9682	7250	685	1100	273	0.11039	0.09197	4.8	Class A
1543	64	F	NA	1	0	0	0	1	0	0	0	1	1	1	0	0	0	0	0	0	1	NA	118	NA	68	3.54	4.19	10.90091	9.02481	9.7	10.4	3.4	3.4	74	57	107	106	27.81955	21.50943	2210	2230	317	335	8.53	6.91404	8.1	Class B
1544	58	M	NA	0	0	1	0	0	0	1	0	0	0	0	0	0	1	1	0	0	0	NA	NA	NA	NA	3.75	4.56	14.24648	11.44616	9.9	13.3	2.7	3.1	77	147	54.9	54.5	32.76596	51.94346	270	129	496	269	2.65385	2.34545	5.9	Class C
1545	72	M	NA	0	0	0	1	0	1	0	0	0	0	0	0	0	1	1	0	0	0	118	137	68	72	3.32	3.44	15.29756	14.71473	10.3	10.8	3.7	3.7	70	64	75.3	58.3	28.11245	24.06015	470	157	104	154	NA	1.88679	NA	Class A
1546	71	M	NA	1	0	0	0	0	1	0	0	1	0	0	0	0	1	1	0	0	0	132	134	64	68	2.53	3.16	20.67649	16.21186	10	11.2	3.4	3.5	97	109	71.4	66.2	37.5969	40.2214	930	585	202	215	7.14286	2.17391	NA	Class A
1547	71	M	26.85257	0	1	0	0	0	1	0	1	0	0	0	0	0	1	1	1	0	0	141	141	74	82	4.34	3.49	11.45716	14.54252	9.8	11.5	3.7	3.9	44	44	208	154	18.25726	18.80342	26900	10900	1140	792	0.43056	1.00775	NA	Class A
1548	68	F	29.21841	1	0	0	0	1	0	0	1	1	0	0	0	0	1	1	0	0	0	156	129	80	58	2.19	2.08	18.11633	19.16703	9.6	9.1	2.5	2.5	66	58	198	150	26.82927	23.48178	279	407	481	873	0.15072	8.36437	6.6	Class A
1549	70	M	21.97232	0	0	1	0	1	0	0	1	0	0	0	0	0	0	0	0	0	0	159	132	92	77	3.85	4.36	13.11489	11.44617	10.7	12.1	4.3	4	91	58	389	246	34.0824	23.48178	140	240	145	128	2	0.001	NA	Class A
1550	63	M	22.67373	0	1	0	0	0	1	0	0	0	0	0	0	0	0	0	0	0	0	132	144	74	73	4.52	NA	11.34148	NA	10.5	NA	3.5	NA	69	74	76.3	52.1	26.64093	29.01961	797	246	606	488	1.49849	NA	NA	Class B
1551	72	M	27.13058	1	0	0	0	0	1	0	0	1	0	0	0	1	1	1	0	0	0	115	133	59	59	1.8	1.65	29.88678	32.87153	10	10.7	4.3	4	79	71	120	73.7	28.21429	26.79245	1530	4950	104	169	0.47368	0.73239	6	Class A
1552	89	M	24.87772	0	0	1	0	0	1	0	1	0	1	0	1	0	1	1	0	0	0	172	151	83	70	4.96	5.07	9.27833	9.05833	8.4	8.9	3.2	3.2	71	69	152	123	31.41593	33.33333	212	53	3000	2030	5.86057	3.39674	NA	Class C
1553	83	M	23.3343	0	1	0	0	0	1	0	0	0	0	0	0	0	0	0	0	0	0	130	148	88	94	4.31	4.51	11.03845	10.46802	10.7	10.2	4	3.6	64	50	129	94.5	23.10469	19.23077	347	843	1480	1390	3.0703	4.83333	5.4	Class C
1554	74	F	25.4459	0	0	1	0	1	0	0	1	0	0	0	0	0	0	0	0	0	0	142	123	56	75	2.19	2.03	17.68198	19.21215	10.4	11.2	3.9	3.6	51	63	27.4	30.1	16.39871	20.32258	7880	8160	576	703	0.14286	0.16043	NA	Class B
1555	88	M	26.077	0	0	1	0	0	1	0	1	0	1	0	1	1	1	1	0	0	0	145	146	82	79	4.17	4.44	11.25399	10.50747	9.4	10.8	4	4.2	73	91	97.7	70.9	28.51563	33.95522	196	122	1840	1440	2.92876	1.71468	NA	Class B
1556	81	F	24.21424	0	0	1	0	1	0	0	1	0	0	0	0	0	1	0	1	0	1	140	NA	80	NA	2.11	2.21	17.9451	16.99873	10	10.8	3.7	3.7	110	94	22	61.7	42.80156	41.40969	377	564	4350	3630	0.92473	0.73803	NA	Class A
1557	76	M	19.44065	1	0	0	0	0	1	0	0	1	0	0	0	0	0	0	0	1	1	122	152	80	90	2.58	2.52	19.84706	20.36461	8.7	11	3.9	4.1	NA	NA	NA	NA	NA	NA	NA	NA	NA	NA	0.19499	0.74156	6.2	Class A
1558	85	F	20.22222	1	0	0	0	1	0	0	0	1	0	0	0	0	1	1	0	0	0	130	121	62	66	1.15	1.02	34.3795	39.06938	10.1	11	4.4	4.3	NA	91	NA	49.2	NA	28.98089	NA	2590	NA	528	NA	0.13043	6.2	Class A
1559	89	F	23.46101	0	0	1	0	0	0	0	0	0	0	0	0	0	1	1	0	1	1	110	120	70	58	2.09	2.3	17.64945	15.8434	10.1	11.5	3.2	3.1	53	48	131	144	20.94862	20.08368	664	1170	4760	4400	3.63636	3.6646	NA	Class A
1560	74	M	22.2146	0	1	0	0	0	1	0	0	0	0	0	1	0	0	0	0	0	0	125	106	63	57	2.59	2.86	19.91509	17.86766	8.2	10.7	3.6	3.8	53	64	397	227	28.80435	28.57143	1310	389	415	200	0.03656	0.000634	4.7	Class A
1561	62	M	26.38443	1	0	0	0	0	1	0	1	1	0	0	0	0	1	1	0	0	0	155	148	80	72	2.82	3.07	19.0903	17.39626	8.9	12.1	4.3	4.2	80	91	301	266	26.40264	29.93421	825	451	111	48	1.23389	1.39286	6.3	Class A
1562	51	M	20.20202	0	0	0	1	1	0	0	1	0	1	0	0	0	1	1	0	0	0	139	157	95	95	4.93	5.17	10.95859	10.40328	10.4	11.6	3.8	3.7	49	84	38.5	14.3	19.44444	29.37063	357	250	777	475	1.54039	1.49731	NA	Class A
1563	55	M	28.93325	1	0	0	0	0	1	0	0	1	0	0	0	0	1	1	0	0	0	113	146	64	82	2.36	2.91	24.00777	19.09056	8.4	11.1	3.8	3.9	45	77	160	79.7	18.14516	29.72973	397	189	5810	2120	6.82563	5.01047	7.8	Class C
1564	49	F	37.53239	1	0	0	0	0	1	0	0	1	0	0	0	0	1	1	0	0	0	125	140	60	67	1.9	1.88	23.24896	23.38369	9.7	9.6	4	3.4	63	75	79.5	54.5	22.1831	26.97842	1780	5090	189	186	4.35644	4.5082	6.7	Class A
1565	57	M	21.06122	1	0	0	0	1	0	0	0	1	0	0	0	0	1	1	0	0	0	122	NA	81	NA	4.48	5	11.78603	10.45183	8.3	10.6	3.1	4	44	119	487	319	20.65728	42.34875	4490	294	255	279	4.18973	NA	NA	Class C
1566	64	F	21.48438	0	0	0	1	1	0	0	1	1	0	0	0	0	1	1	0	0	0	200	138	80	74	1.23	NA	34.65104	NA	8.3	NA	2.9	NA	54	66	277	328	26.21359	30.84112	590	311	3250	1080	16.75676	NA	5	Class B
1567	76	M	17.79257	0	1	0	0	0	1	0	1	0	0	0	0	0	0	0	0	0	0	129	NA	93	NA	3.16	3.11	15.8983	16.17813	9.7	10.2	4.1	3.8	86	107	259	270	35.83333	44.39834	59	183	1190	1640	0.43373	3.31034	5.4	Class A
1568	78	M	21.98989	0	0	0	1	0	1	0	0	0	0	0	0	0	1	1	0	0	0	126	113	75	73	2.63	2.81	19.2903	17.94262	9.9	11.9	3.9	3.8	98	128	76.3	60.9	48.27586	59.53488	479	920	275	311	0.52812	0.6215	NA	Class A
1569	55	M	NA	0	0	0	1	0	0	1	0	0	0	0	0	0	1	1	0	0	0	NA	109	NA	74	2.87	NA	19.38183	NA	11.4	NA	3.7	NA	72	NA	131	NA	25.35211	NA	251	NA	55	NA	0.53623	NA	NA	Class A
1570	68	F	20.07775	0	0	0	1	1	0	0	0	0	0	0	0	0	1	1	0	0	0	132	NA	62	NA	2.11	2.29	18.8691	17.25266	9.5	12.1	3.6	3.8	90	96	36	8.8	32.3741	29.62963	384	444	445	308	2.64095	2.36975	NA	Class A
1571	74	M	22.45051	0	0	1	0	0	0	1	0	1	0	0	0	0	1	1	0	0	0	139	117	71	59	1.62	NA	33.27536	NA	10.1	NA	4.3	NA	49	25	12.4	62.5	13.3515	8.250825	256	53800	238	336	0.10526	NA	6	Class A
1572	83	F	28.4671	0	0	1	0	1	0	0	0	0	0	0	0	0	0	0	0	0	1	128	128	73	65	2.96	2.87	12.30489	12.72764	10.5	11.1	4	4.05	72	97	117	134	28.125	37.89063	3600	812	343	335	1.32048	1.341	5.2	Class A
1573	73	M	23.27021	1	0	0	0	0	1	0	0	1	0	0	0	0	1	1	0	1	1	129	151	53	72	5.07	4.91	9.58847	9.93082	9.3	10.3	4	3.8	84	101	497	411	35.74468	44.29825	314	225	550	779	2.05184	2.28758	6.3	Class B
1574	75	M	24.42742	0	0	0	1	0	1	0	1	0	0	0	0	0	0	0	0	0	0	140	125	73	77	2.72	2.67	18.80359	19.18915	8.4	10.2	4.3	4.2	83	78	290	236	30.85502	30.11583	1610	450	1100	219	3.74408	2.95331	NA	Class B
1575	76	M	23.93102	0	0	0	1	0	1	0	0	0	0	0	0	0	0	0	0	0	0	122	126	56	64	2.19	2.11	23.74446	24.73109	10.9	11	3.6	3.5	62	41	33.4	36.4	22.87823	15.01832	5240	1980	71	64	NA	1.89216	5.7	Class A
1576	75	F	26.89618	0	1	0	0	1	0	0	1	0	0	0	0	0	0	0	0	0	0	172	140	75	90	4.18	4.87	8.68434	7.34762	8.8	10.6	4.2	3.5	93	120	149	306	38.91213	80	341	306	713	605	5.80552	NA	NA	Class A
1577	72	M	19.99679	1	0	0	0	0	0	1	1	1	0	0	0	0	1	1	1	0	0	181	142	71	60	3.28	3.76	15.50177	13.35032	9.2	10.7	3.5	3.9	82	91	203	125	31.53846	31.48789	382	636	404	1190	5.80409	7.99564	6.5	Class A
1578	80	F	25.91037	0	0	0	1	1	0	0	1	0	0	0	0	0	1	1	0	0	0	156	132	83	78	1.84	1.88	20.91937	20.43293	10.5	12.9	3.7	3.8	71	116	128	52.8	23.50993	35.15152	396	295	323	274	0.99432	2.21729	NA	Class A
1579	76	F	18.07238	0	0	0	1	1	0	0	0	0	0	1	0	0	1	0	1	0	1	120	128	60	83	0.98	1.18	42.29133	34.38622	10.6	11.4	4.2	4.2	63	83	45	56.8	16.8	23.71429	85	99	2410	1660	0.28571	0.000714	6	Class A
1580	80	M	29.14063	0	1	0	0	0	1	0	0	1	1	0	0	0	1	1	0	0	0	132	NA	74	NA	3.19	NA	15.50486	NA	10.1	NA	3.7	NA	61	NA	147	NA	17.03911	NA	254	NA	4010	NA	0.43682	NA	6.3	Class A
1581	62	M	23.87543	0	0	1	0	1	0	0	0	0	0	0	0	1	1	1	0	0	0	105	98	59	59	1.86	1.83	30.09804	30.49787	10	12	4.2	4.2	77	105	344	236	29.38931	37.23404	128	114	112	96	0.13995	0.08872	NA	Class A
1582	48	M	23.67125	0	0	1	0	0	1	0	1	0	0	0	0	0	1	0	1	0	0	136	144	94	100	4.68	5.38	11.8042	10.13467	10	12.9	4.2	4.2	91	110	162	39.1	31.92982	34.16149	961	483	163	433	0.58486	0.71269	5.3	Class B
1583	77	F	26.41727	0	0	1	0	1	0	0	1	0	0	0	1	0	1	1	0	0	0	140	130	83	78	3.35	3.26	10.98056	11.31262	9.2	10.9	4.34	4.66	82	64	65.1	71.1	33.33333	24.52107	659	1480	107	142	1	2.91817	NA	Class B
1584	39	M	24.99599	1	0	0	0	1	0	0	1	1	0	0	0	0	1	1	0	0	0	154	138	88	92	3.06	2.99	19.94284	20.45417	9.4	11.7	3.7	3.5	90	91	283	102	33.45725	35.40856	113	50	113	180	4	2	6.2	Class A
1585	83	M	18.02596	1	0	0	0	1	0	0	1	1	1	0	1	1	0	0	0	0	0	135	109	90	53	2.95	2.87	16.71249	17.22279	9.5	9.8	3	3.3	39	48	12.4	12.5	12.5	13.7931	477	903	2000	2150	NA	0.05102	6	Class A
1586	49	M	21.45329	1	0	0	0	0	1	0	1	1	0	0	0	0	1	1	0	0	0	156	144	70	70	4.74	NA	11.57215	NA	9.6	NA	3.9	NA	68	NA	124	NA	25.75758	NA	264	NA	542	NA	3.49943	NA	4.9	Class C
1587	81	M	22.26451	0	1	0	0	0	1	0	0	0	0	0	0	0	1	1	0	0	0	127	112	83	78	3.25	3.62	15.0847	13.40633	10.4	11.5	4.1	4.4	77	108	330	298	31.30081	44.6281	159	1270	294	434	1.58108	1.82716	NA	Class A
1588	80	M	20.82094	0	0	0	1	0	1	0	1	1	0	0	1	1	1	1	0	1	1	140	110	61	61	1.31	1.34	41.05066	40.04629	9.6	14.1	4.2	4.6	36	97	11.6	36.6	10.11236	27.09497	2200	1110	182	132	0.18271	0.34024	6	Class A
1589	75	M	NA	1	0	0	0	0	1	0	0	1	0	0	0	0	1	1	0	0	0	NA	NA	NA	NA	2.61	NA	19.67227	NA	8.1	NA	NA	NA	13	NA	9.7	NA	3.834808	NA	2030	NA	673	NA	1.87382	NA	NA	Class A
1590	73	M	20.86112	0	0	0	1	0	1	0	0	0	0	0	0	0	1	1	0	0	0	139	142	79	82	2.3	NA	22.67785	NA	11.3	NA	3.8	NA	84	NA	89.3	NA	34.14634	NA	141	NA	349	NA	0.17778	NA	5.2	Class A
1591	26	F	NA	0	0	0	1	1	0	0	1	0	0	0	0	0	1	1	0	0	0	139	161	94	107	2.7	3.15	18.98615	16.03973	8.8	9.3	3.5	3.5	50	51	7.1	8	16.66667	17.05686	50	50	617	1480	2.14286	2.91262	NA	Class A
1592	61	M	19.09286	0	0	1	0	0	1	0	0	0	0	0	0	0	0	0	0	1	1	130	171	77	102	2.68	4.14	20.27835	12.60125	7.7	9.9	2.4	2.7	49	79	70.1	167	23.44498	32.2449	22900	4820	1050	2510	2.15869	2.57194	6.1	Class A
1593	85	F	24.65303	0	1	0	0	1	0	0	1	0	0	0	0	0	0	0	0	0	0	140	150	80	60	2.64	2.89	13.85059	12.54529	10.7	12.1	4.3	4.3	62	67	293	207	28.3105	31.60377	191	180	1720	1060	5.23333	3.425	NA	Class B
1594	63	F	14.28324	0	0	0	1	0	1	0	0	0	0	0	0	0	0	0	0	0	0	115	125	56	58	2.55	2.74	15.67763	14.49227	10.1	10.9	3.4	3.4	53	71	202	192	23.34802	31.98198	77	100	465	504	0.95556	0.6	NA	Class A
1595	89	M	22.6697	1	0	0	0	0	1	0	0	1	0	0	0	1	1	1	0	0	0	113	155	51	74	2.71	2.59	17.97457	18.8876	9.8	11.1	3.2	3.6	44	86	127	64.3	20.37037	38.56502	1070	297	540	503	4.15584	8.70796	NA	Class A
1596	54	M	24.30368	0	0	0	1	0	1	0	0	0	0	0	0	0	1	1	1	0	0	134	136	78	82	4.31	4.47	12.48782	11.99964	11.2	9.9	4	4	93	50	105	84	33.81818	18.18182	688	844	929	773	0.8913	0.67105	5.6	Class B
1597	66	F	21.28565	0	0	0	1	1	0	0	1	1	0	0	0	0	0	0	0	0	0	153	139	90	80	2.81	3.06	13.91081	12.67237	10.7	10.9	3.8	3.8	NA	98	NA	28.8	NA	29.08012	NA	157	NA	901	4.97674	2.97938	6.3	Class A
1598	75	F	23.68609	0	0	0	1	1	0	0	1	1	1	0	0	1	0	0	0	0	1	153	124	72	58	2.92	3.39	12.85804	10.92107	9.9	9.8	3.4	3.2	58	56	133	101	27.48815	26.04651	2160	286	3780	4080	7.79964	6.83258	NA	Class A
1599	71	M	22.93858	0	1	0	0	0	1	0	0	0	1	0	0	1	1	1	0	0	0	129	138	73	80	2.15	2.26	24.70603	23.39354	10.6	11.9	3.5	3.5	93	89	118	72.9	35.76923	33.33333	462	164	252	147	6.01134	2.43836	NA	Class B
1600	61	M	18.93878	0	0	0	1	1	0	0	0	0	0	0	0	1	1	1	0	0	1	132	112	73	69	2.92	4.61	18.46219	11.20271	8.7	14.5	3.5	4.5	58	104	41.4	28.8	25.77778	35.86207	2760	820	1330	95	0.26393	0.44479	4.8	Class A
1601	88	M	23.03152	1	0	0	0	0	0	0	0	1	0	0	0	0	0	0	0	0	0	NA	NA	NA	NA	1.18	1.85	44.78126	27.29235	10.7	10.9	3.4	3.4	62	88	236	220	27.92793	39.11111	603	477	804	590	NA	NA	5.5	Class A
1602	74	F	20.34329	0	0	1	0	1	0	0	0	0	0	1	0	0	1	1	0	0	0	104	100	59	64	2.98	2.84	12.62362	13.30597	10.1	11.9	4.6	4.5	42	53	140	74.5	14.33447	17.54967	140	74	918	836	0.7963	0.56757	NA	Class A
1603	41	M	27.01686	1	0	0	0	1	0	0	0	1	0	0	0	0	1	1	0	1	1	132	132	73	74	3.94	4.26	14.90939	13.68859	7.5	9.9	3.2	2.6	72	59	520	395	30	31.21693	796	1270	1700	1280	9.94975	12.23479	4.6	Class B
1604	78	F	26.62164	0	1	0	0	1	0	0	0	0	0	0	0	0	1	0	1	0	0	123	160	63	83	2.52	2.52	14.93767	14.93767	9.7	10.7	4.2	4	60	85	56.8	33.6	22.38806	34	175	154	185	209	0.72761	1.80107	NA	Class A
1605	72	M	22.37568	0	0	0	1	0	1	0	0	0	0	0	1	0	1	1	0	0	0	129	118	74	80	3.63	3.05	13.87425	16.78507	9.2	10.8	4	3.9	56	65	229	94.2	20.66421	23.38129	409	406	311	326	0.22947	0.23184	5.9	Class A
1606	69	F	18.32628	0	1	0	0	1	0	0	1	0	0	0	0	0	1	1	0	0	1	156	156	70	80	2.58	2.32	15.07942	16.93763	9.8	11.3	3.9	NA	77	61	52.5	43.6	26.73611	21.70819	50	110	963	563	0.93541	0.84586	NA	Class A
1607	79	M	25.73203	0	0	0	1	1	0	0	0	0	0	0	0	0	1	1	0	0	0	132	132	70	72	3.36	3.07	14.7017	16.22757	8.8	9.7	3.5	3.8	61	82	204	161	34.07821	45.55556	767	310	1890	2040	1.76471	1.59447	5.7	Class B
1608	73	M	19.60519	1	0	0	0	0	1	0	1	1	1	1	0	0	0	0	0	0	1	147	140	83	55	2.48	2.77	20.96515	18.57613	10.3	9.1	4	3.6	60	19	63.8	69	20.13423	7.169811	285	18700	7530	9760	0.75949	0.53061	7.2	Class A
1609	88	M	23.32414	0	1	0	0	0	1	0	0	1	0	0	0	0	0	0	0	0	0	111	90	53	48	3.49	3.86	13.67364	12.24641	8.9	11.4	2.9	2.9	51	71	150	74.5	25.12315	33.97129	1810	4060	1410	1580	1.71105	2.89032	NA	Class A
1610	74	M	23.62949	0	1	0	0	0	1	0	0	0	0	0	0	0	1	1	0	0	0	131	131	76	84	2.14	1.91	24.53915	27.68271	10.8	11.7	4.1	4.1	68	70	144	86.5	25.37313	25.17986	839	1580	150	90	0.03423	0.04742	6	Class A
1611	73	M	26.71222	0	1	0	0	1	0	0	1	0	0	0	0	0	1	1	0	0	0	147	140	69	70	3.6	3.5	13.94547	14.3259	9.8	11.8	4	3.9	85	NA	130	NA	33.86454	NA	100	NA	429	NA	0.45608	5.06375	NA	Class B
1612	68	M	20.27046	0	0	1	0	0	1	0	0	1	1	1	0	0	1	0	1	0	0	120	124	60	62	2.69	2.81	19.57594	18.66324	8.3	9.1	4	NA	57	55	157	67.7	24.05063	20.75472	311	250	875	771	0.14248	NA	6	Class A
1613	51	F	16.44908	0	0	1	0	1	0	0	0	0	0	0	0	0	0	0	0	0	0	110	110	80	80	3.87	3.85	10.55402	10.55502	9.6	11.8	4.5	4.5	92	52	85.6	41.1	32.97491	13.54167	1390	3160	348	436	0.40248	0.50909	NA	Class A
1614	73	M	18.125	0	0	0	1	1	0	0	0	0	1	0	0	0	1	0	1	0	0	116	134	52	56	3.14	3.02	16.19523	16.90054	10.6	13.4	4.2	4.7	93	162	145	139	29.0625	42.97082	194	303	173	215	0.78261	NA	5.9	Class A
1615	70	M	19.83516	1	0	0	0	0	1	0	1	1	0	0	0	0	1	1	0	0	0	158	175	77	82	2.2	1.9	24.19069	28.28356	9.7	12.7	3.9	4.2	67	76	104	44.6	29.00433	26.66667	633	393	342	84	0.29359	0.31187	5.7	Class A
1616	82	F	21.80323	0	1	0	0	0	0	0	0	0	0	0	0	0	0	0	0	0	0	118	146	68	78	2.4	NA	15.53211	NA	10.9	NA	4	NA	86	56	123	257	31.85185	22.76423	194	1250	916	844	2.05788	NA	NA	Class A
1617	60	F	26.09232	1	0	0	0	1	0	0	0	1	0	0	0	0	1	1	0	0	0	132	NA	73	NA	1.83	NA	22.85574	NA	10.3	NA	4	NA	74	NA	173	NA	26.90909	NA	125	NA	945	NA	9.54545	NA	5.6	Class A
1618	51	F	26	0	0	0	1	1	0	0	0	0	0	0	0	0	0	0	0	0	0	117	121	72	74	2.41	2.38	17.71931	17.96381	9.2	10.8	3.7	3.6	71	87	56.9	41.9	27.95276	32.58427	304	215	207	126	1.27928	1.05063	NA	Class A
1619	51	M	41.86812	1	0	0	0	0	0	1	0	1	0	0	0	0	1	1	1	0	0	138	148	87	95	2.52	2.62	22.83478	21.76142	11	11.6	3.5	3.6	77	69	179	130	33.33333	29.61373	5020	7130	81	62	2.94811	3.29472	5.7	Class B
1620	73	M	22.31262	0	0	0	1	1	0	0	0	0	0	0	0	0	1	1	0	0	1	122	103	64	66	2.83	3.75	18.1457	13.33638	11	11.9	3.5	3.3	88	98	53.7	59.1	35.34137	37.69231	96	264	312	463	4.27097	5.44118	NA	Class B
1621	79	M	20.3125	1	0	0	0	0	1	0	0	1	0	0	0	1	1	1	1	1	1	110	116	49	57	3.04	2.41	16.40284	21.14736	9.6	11.1	3.3	3.1	80	117	43.2	71.1	27.30375	42.54545	58	50	306	311	2.84091	6.03499	6.9	Class A
1622	68	F	NA	0	1	0	0	1	0	0	0	0	0	0	1	1	0	0	0	0	1	128	196	78	98	2.13	1.77	18.67535	22.86827	10.5	9.6	3.8	3.7	60	95	26.7	45.8	19.10828	35.98485	100	158	220	1410	1.57273	2.925	NA	Class A
1623	68	M	NA	0	0	1	0	0	1	0	0	0	0	0	0	0	1	1	0	0	0	NA	NA	NA	NA	2.47	2.61	21.49123	20.23331	11	11	NA	NA	76	69	44.2	42.8	32.75862	28.27869	179	172	363	580	NA	NA	5.4	Class A
1624	74	F	NA	1	0	0	0	1	0	0	0	1	0	0	0	0	0	0	0	0	1	115	161	61	70	2.23	2.12	17.33529	18.32168	9.1	12.7	2.9	3.6	34	79	48.5	33.8	14.04959	27.05479	40700	659	462	294	2.42609	2.375	6.6	Class A
1625	26	M	20.22505	0	0	1	0	1	0	0	1	0	0	0	0	0	1	0	1	0	0	140	110	82	70	4.28	5.29	15.52047	12.17699	9.8	12.9	3.7	3.9	37	83	132	63.4	17.37089	38.42593	774	100	411	124	2.01863	1.62521	NA	Class A
